# Multiplicative Stereocontrol in the Light-Driven Deracemization of Benzylic Nitriles

**DOI:** 10.1021/jacs.6c15890

**Published:** 2026-09-24

**Authors:** Y. Emily Du, Alexander S. Hurlburt, Justin Y. Wang, Qiaolin Yan, Robert R. Knowles

**Affiliations:** Department of Chemistry, 6740 Princeton University, Princeton, New Jersey 08544, United States

## Abstract

Light-driven deracemization has emerged as a powerful strategy for the optical enrichment of chiral organic molecules. In numerous cases, a single chiral catalyst promotes both stereoselective cleavage and re-formation of a C–H bond at the stereogenic center. However, methods to establish the mechanisms of these processes and quantify their individual contributions to overall deracemization enantioselectivity remain limited. Here we report a light-driven deracemization of benzylic nitriles with up to 99% yield and 98% ee enabled by cooperative photoredox and chiral phosphate catalysis that achieves high overall enantioselectivity through the combination of two moderately stereoselective steps. The reaction proceeds through a stereoablative sequence of oxidation and deprotonation, followed by stereoselective C–H bond formation to establish the α-nitrile stereocenter. Competition between back electron transfer and deprotonation of a key arene radical cation intermediate results in selective processing of the minor enantiomer in the reaction. The deprotonation step exhibits a primary kinetic isotope effect, enabling the extent of deracemization to be improved upon solvent isotopic substitution. The stereoselectivity of the C–H bond-forming step correlates with the ground-state reduction potential of the photocatalyst, consistent with the involvement of an enantioselective reductive proton-coupled electron transfer. The participation of a single chiral catalyst in two cooperative stereoselective steps produces a non-classical nonlinear effect that is shown to arise from the proposed mechanistic model. More broadly, these studies provide a set of diagnostic tools for analyzing the origins of enantioselectivity in deracemization reactions.

## Graphical abstract

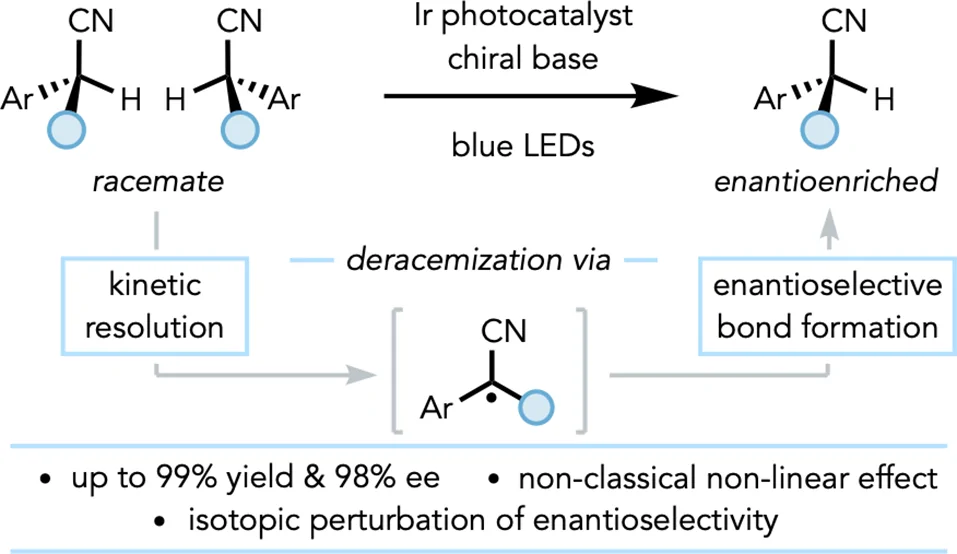

## Introduction

Light-driven methods for deracemization, wherein a racemic mixture is optically enriched by the selective conversion of one enantiomer into the other, have garnered significant interest over the past decade.^1–3^ To overcome the kinetic constraints imposed by microscopic reversibility, photochemical deracemization reactions must involve non-degenerate mechanisms of bond cleavage and formation, either or both of which may be stereoselective.^4^ In a kinetic resolution-based deracemization, selective processing of one enantiomer through a stereoablative pathway followed by non-selective bond re-formation results in accumulation of the slower reacting enantiomer (Figure 1A, kinetic resolution). Alternatively, deracemization may be accomplished via non-selective conversion of both enantiomers to an achiral intermediate followed by stereoselective bond formation (Figure 1A, enantioselective bond formation). If both bond cleavage and bond formation are selective and biased towards enrichment of the same enantiomer, the overall extent of deracemization can be expressed as the product of the selectivity of each step. In such cases, high levels of deracemization have been observed despite modest levels of stepwise enantiodiscrimination or enantioinduction.^3b,3f^

**Figure 1:**
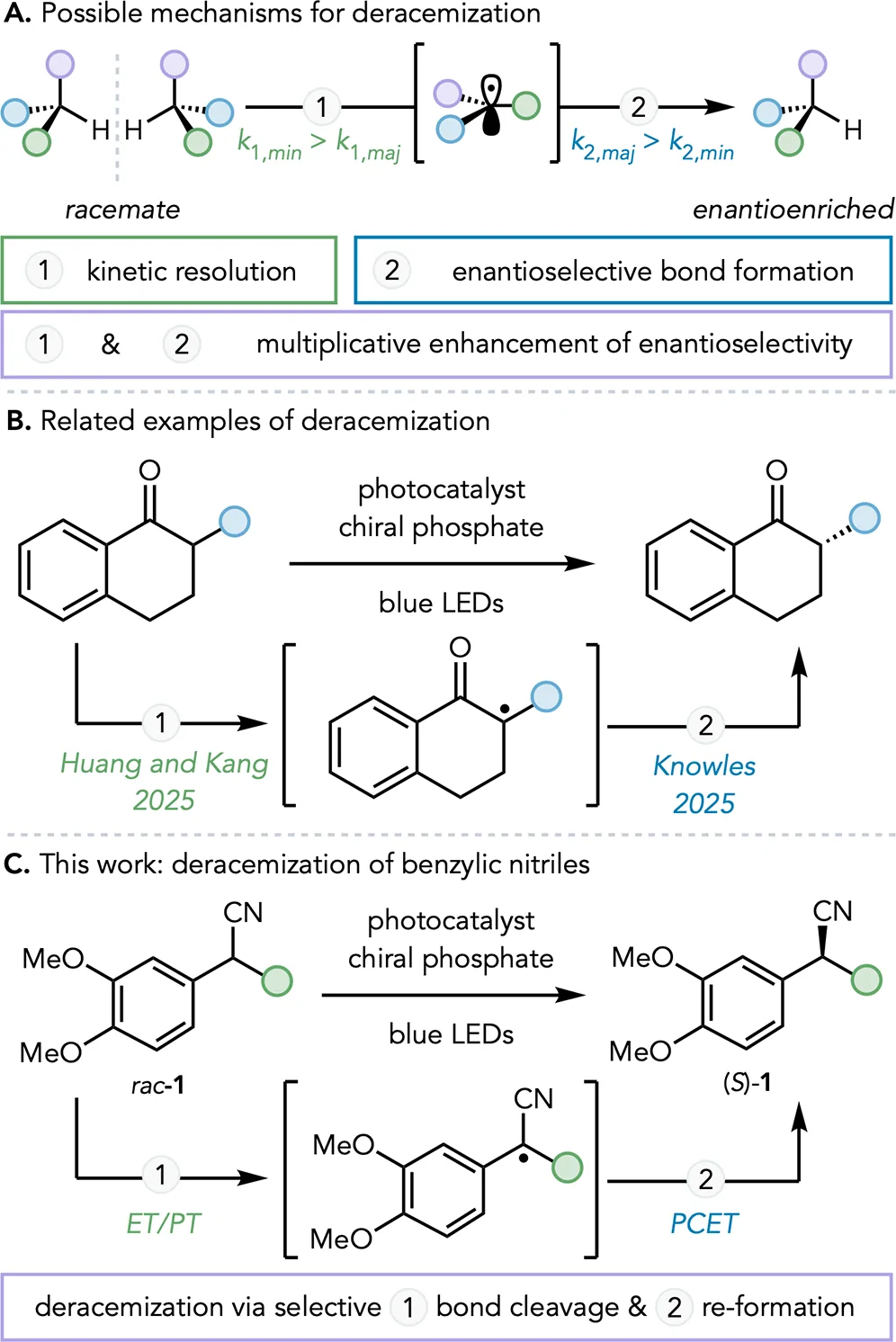
(A) Possible mechanisms for deracemization. (B) Related examples of deracemization. (C) This work: deracemization of benzylic nitriles via multiple selective steps.

However, direct determination of the mechanistic origins of stereoselectivity in a deracemization reaction can be challenging as the reactants and products differ only in stereochemical configuration.^5^ In cases where a single chiral catalyst participates in multiple elementary steps, subtle changes to catalyst structure or reaction conditions may significantly affect how selectivity is partitioned across bond cleavage and bond formation. For example, differing origins of selectivity were proposed in simultaneous reports from our laboratory and from Huang and Kang on the deracemization of cyclic α-aryl carbonyls under photoredox and chiral phosphate co-catalysis (Figure 1B).^6,7^ In the former case, stoichiometric generation of a prochiral intermediate suggested high stereoselectivity in the bond-forming step; in the latter case, deuterium incorporation experiments implicated a stereoselective kinetic resolution of starting material.

Given these precedents, we questioned whether analogous photoredox and chiral phosphate deracemization chemistry could be extended to acyclic benzylic nitriles, and how selectivity might be partitioned across stereoablative and stereoregenerative steps. Chiral nitriles commonly appear in pharmaceuticals and natural products and are versatile precursors to a range of important functional groups, including chiral amides, imines, amines, and *N*-heterocycles.^8^ We note that while the enantioselective synthesis of chiral nitriles by radical-rebound copper catalysis has been studied extensively,^9^ the complementary catalytic deracemization of α-chiral nitriles has yet to be reported.

Herein, we report a protocol for light-driven deracemization of benzylic nitriles through cooperative photoredox and chiral phosphate co-catalysis (Figure 1C). Mechanistic studies reveal a multiplicative enhancement of selectivity affording highly enantioenriched products under the action of a single chiral catalyst, despite the modest selectivity of the component kinetic resolution and C–H bond forming steps. Moreover, we observed a strong correlation between ground-state photocatalyst reduction potential and the extent of deracemization,^6^ suggesting an enantioselective reductive proton-coupled electron-transfer (PCET) mechanism for C–H bond formation as opposed to a stepwise electron-transfer/proton-transfer (ET/PT) sequence. The analyses presented may find further use in the study of the origins of enantioselectivity in light-driven deracemization reactions.

## Results & Discussion

Drawing on our prior work,^6^ we hypothesized that the following sequence of elementary steps could enable the photochemical deracemization of benzylic nitriles (Figure 2). Under blue light irradiation, the excited state iridium photocatalyst ^*^ **Ir** ^**III**^ is sufficiently oxidizing to generate arene radical cation **1-INT-1** from **1**. The benzylic C–H bond of **1-INT-1** is markedly acidified (p*K*_a_ ≈ −11 in DMSO, see Supporting Information for details), and may undergo stereoablative deprotonation by a chiral phosphate base (**CPB**, p*K*_aH_ ≈ 4 in DMSO)^10^ to yield the corresponding chiral phosphoric acid (**CPA**) and the neutral benzylic radical **1-INT-2**. However, **1-INT-1** may also undergo unproductive back electron transfer (BET) with the ground-state **Ir** ^**II**^ species to regenerate **Ir** ^**III**^ and **1**. **1-INT-2** may then undergo reduction by **Ir** ^**II**^ to the prochiral keteniminate **1-INT-3**, and subsequent protonation by the **CPA** to reform the α-nitrile stereocenter of **1**. Alternatively, **1-INT-2** may undergo reductive PCET, in which a proton and electron are simultaneously transferred from the **CPA** and **Ir** ^**II**^, respectively, to reform the C–H bond of **1** directly (Figure 2, purple arrow).^6,11^ Importantly, neither ET/PT nor reductive PCET to the neutral radical **1-INT-2** are the microscopic reverse of ET/PT from **1**. As such, the **CPB** co-catalyst could impart stereoselectivity in either the stereoablative C–H bond-breaking step, the C–H bond-forming step, or both steps of the deracemization reaction.

**Figure 2:**
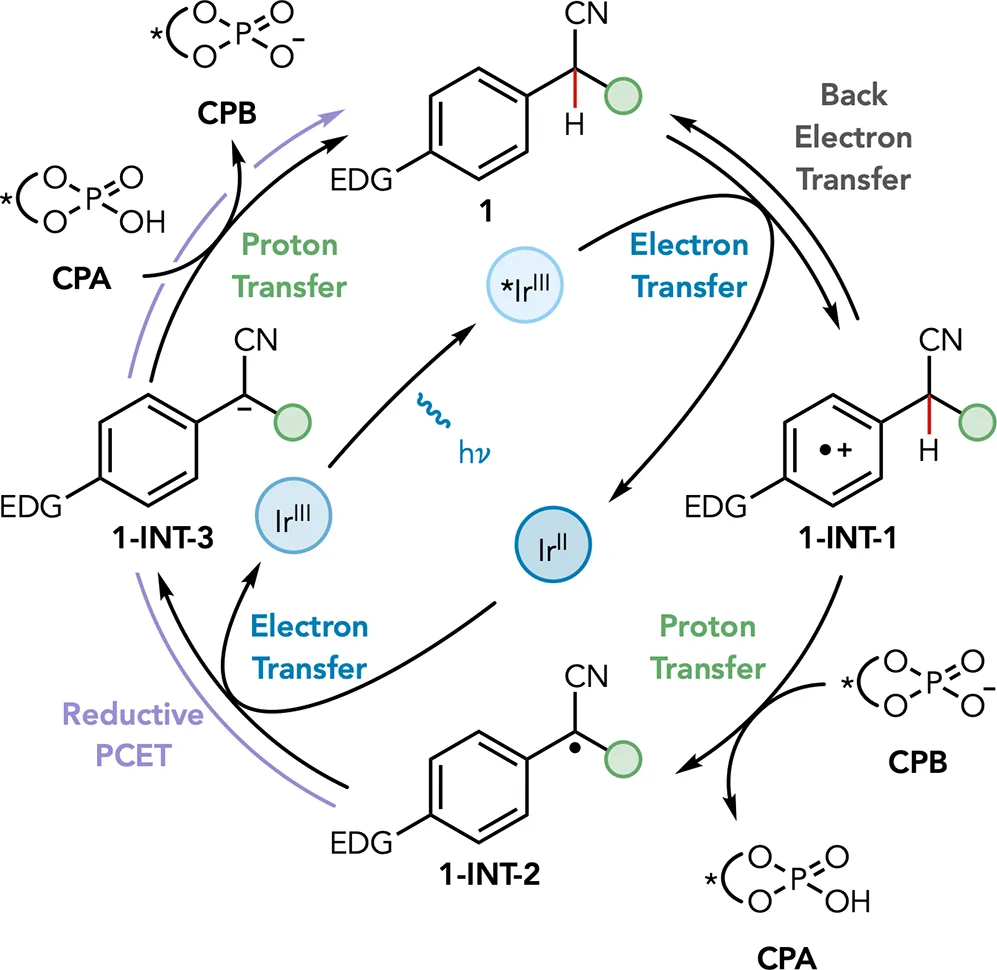
Proposed catalytic cycle.

From this mechanistic hypothesis, we began our study of the light-driven deracemization of electron-rich benzylic nitrile **1a** (*E*_p/2_ = +1.04 V vs Fc^+^/Fc in MeCN). A solution of **1a** in the presence of 2 mol % of [Ir­(dCF_3_(CF_3_)­ppy)_2_(5,5′-dCF_3_bpy)]­PF_6_ (**Ir-1**, *E*_1/2_(*Ir^III^/Ir^II^) = +1.43 V vs Fc^+^/Fc in MeCN)^12^ and 2.5 mol % of chiral BINOL-derived phosphate base (**CPB-1**) in toluene was irradiated with 456 nm light at 30 °C for 2 h, returning enantioenriched **1a** in 90% yield and 46% ee (Table 1, entry 1). A survey of phosphate bases revealed a strong countercation effect on selectivity, wherein exchanging tetrabutylammonium phosphate **CPB-1** for tetramethylphosphonium phosphate **CPB-2** increased the enantioselectivity of the reaction to 72% ee (entry 2). The reaction rate was further accelerated by the addition of water (see Supporting Information), returning **1a** in 86% ee in 2 h (entry 3). While increasing the concentration of **CPB-2** to 7 mol % afforded deracemized product in 90% ee (entry 4), we also observed improved solubility of the photocatalyst, presumably upon *in situ* ion-pairing with the base. Substituting ion-paired **Ir-CPB** as the photocatalyst afforded (*S*)-**1a** in 92% ee (entry 5).^7^ Addition of **CPA-1** (2.5 mol %) further improved the selectivity of deracemization to 94% ee (entry 6). Finally, cooling the reaction to 12 °C resulted in formation of enantioenriched (*S*)-**1a** in 96% yield and 97% ee (entry 7).^13^ No deracemization was observed in the absence of either photocatalyst or irradiation (entries 8–9).

**Table 1:**
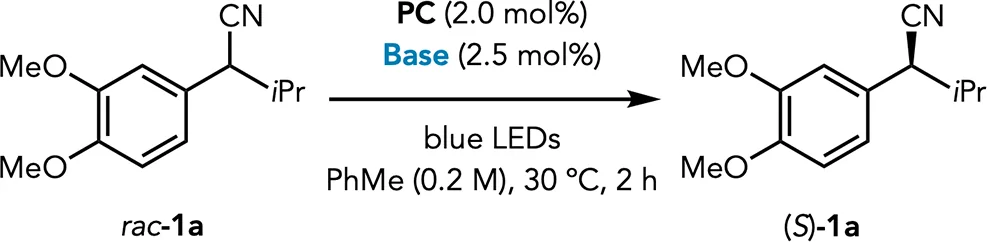

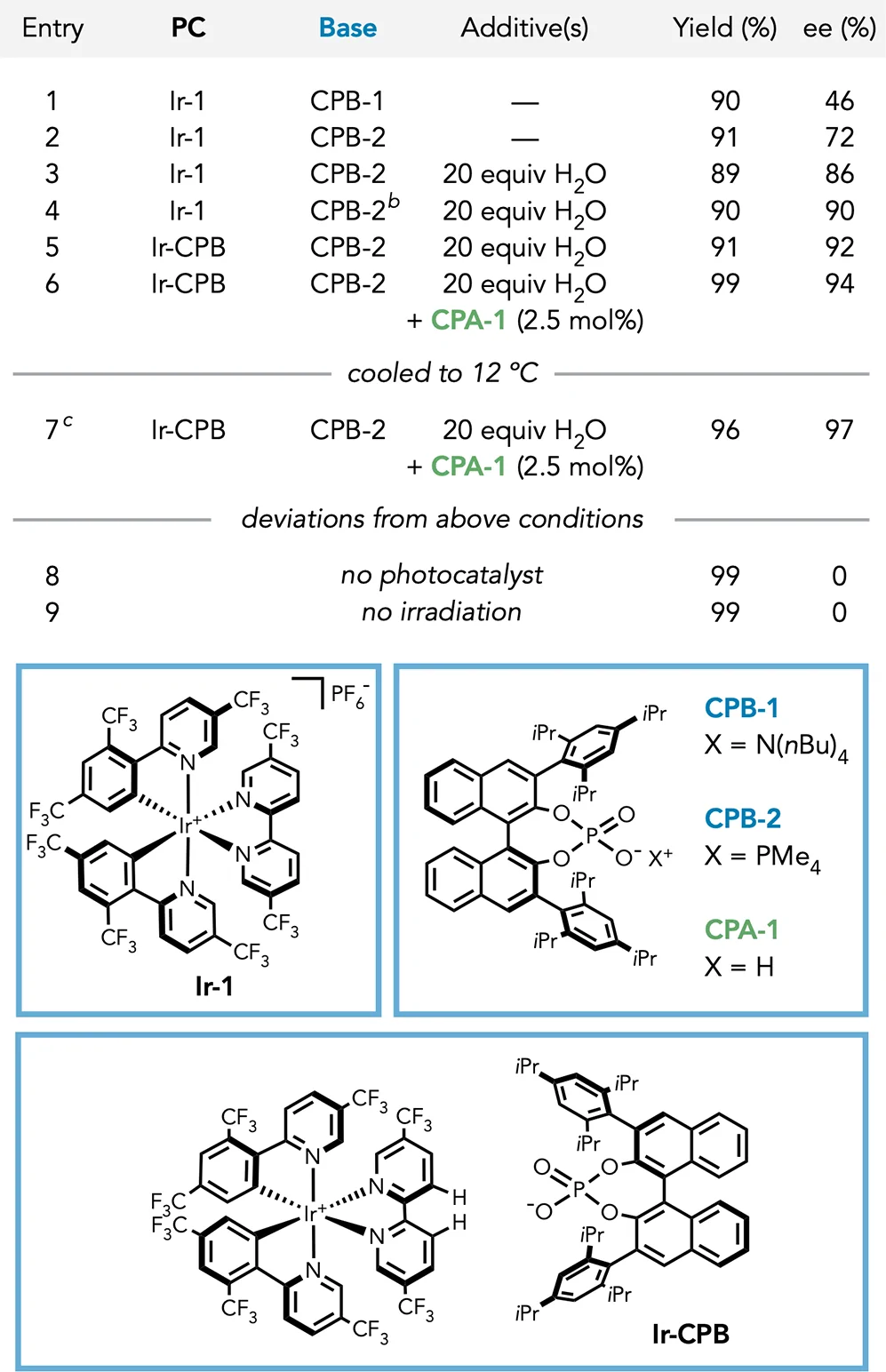
Optimization of Reaction Conditions^a^

a Reactions were performed on 0.05 mmol scale with respect to *rac* **-1a**. Yields were determined by ^1^H NMR spectroscopy with piperonal as an internal standard. Enantiomeric excess was determined by LC-SFC.

b Reaction performed with 7 mol% **CPB-2**.

c Reactions performed on 0.5 mmol scale, yield and enantioselectivity refer to isolated material.

With optimized conditions in hand, we then assessed the generality of the reaction (Table 2). Acyclic benzylic nitriles bearing α-alkyl substituents of varied steric profiles were successfully enantioenriched to 90–97% ee (**1a**–**d**). Additionally, substrates bearing cyclic aliphatic moieties underwent deracemization in 76–92% ee (**1e**–**g**). Notably, this method was amenable to the deracemization of cyclopropane **1e** without evidence for the formation of ring opened byproducts.^14^ Arenes bearing isopropoxy and benzyloxy groups also underwent deracemization in 95% and 70% ee, respectively (**1h** and **1i**).

**Table 2:**
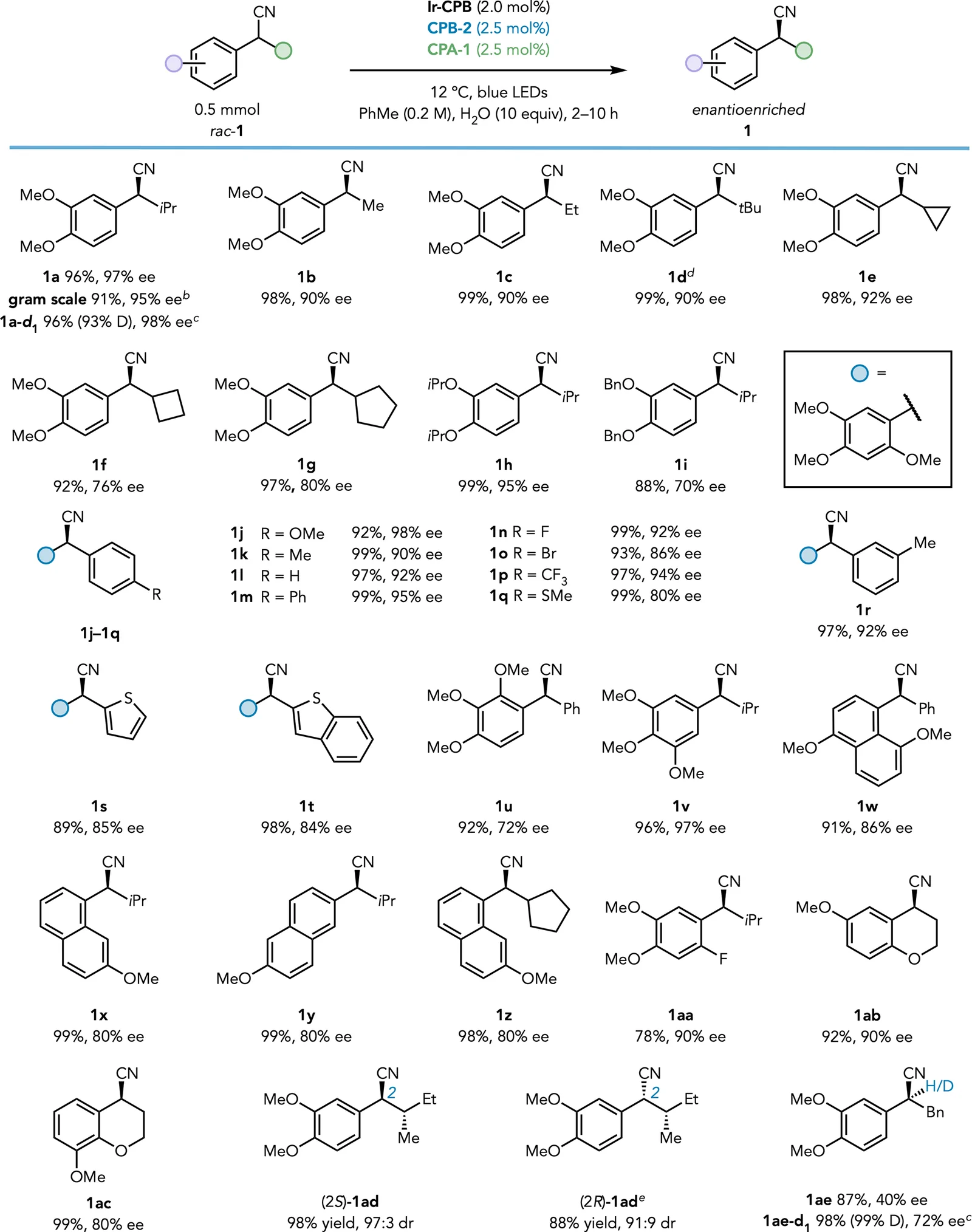
Substrate Scope for the Deracemization of Benzylic Nitriles^a^

a All reactions were performed on 0.5 mmol scale unless otherwise noted. Yields and ee values are reported as averages of two experiments and are for isolated compounds.

b Reaction performed on 1.0 g scale with 1 mol% **Ir-CPB**, 1.25 mol% **CPB-2**, and 1.25 mol% **CPA-1**.

c Reaction performed with D_2_O (10 equiv) instead of H_2_O.

d Irradiated for 22 h.

e Performed with **Ir**-(*R*)-**CPB**, (*R*)-**CPB 2**, and (*R*)-**CPA-1**.

We then investigated the deracemization of α,α-diarylacetonitriles. Chiral 1,1-diaryl motifs are found in a variety of pharmaceutical compounds, but general access to these compounds is challenging due to minimal steric differentiation between aryl groups at the stereogenic center.^15^ We observed high enantioselectivity in the deracemization of 2,4,5-trimethoxybenzene-derived substrates (**1j**–**1q**). These reactions were largely insensitive to the electronic effects of the arene *para*-substituent; the presence of an electron-donating methoxy or an electron-withdrawing trifluoromethyl group resulted in deracemized products with 98% and 94% ee, respectively (**1j** and **1p**). Thiophene and benzothiophene derivatives were also tolerated in this protocol, yielding products in 85% and 84% ee, respectively (**1s** and **1t**).

Modification of the electron rich α-aryl moiety to trimethoxybenzene and methoxynaphthyl isomers provided substrates that could be enantioenriched in 72% to 97% ee (**1u–z**). Additionally, fluorination at the *ortho*-position on the electron-rich arene resulted in only a moderate decrease in stereoselectivity, yielding enantioenriched product in 90% ee (**1aa**). Fused ring systems were also tolerated, enabling the deracemization of chromane derivatives to 90% and 80% ee (**1ab** and **1ac**). We demonstrated that the selectivity of epimerization is catalyst-controlled by subjecting substrate **1ad** (1:1 dr, >99% ee) bearing a homobenzylic stereocenter to both (*S*) and (*R*) configurations of the catalyst mixture. In the matched case, where catalyst and substrate chirality favor the same major diastereomer, the deracemized product was obtained in 97:3 dr ((2*S*)-**1ad**), while the mismatched case resulted in a dr of 91:9 ((2*R*)-**1ad**). We also observed that this transformation is amenable to hydrogen isotope exchange (HIE), wherein substitution of H_2_O for D_2_O furnishes isotopologue **1a-*d*_1_** in 98% ee with 93% deuterium incorporation. Interestingly, this ee was higher than the 97% ee of **1a** observed under the model conditions with H_2_O. This phenomenon was general for other substrates, and we leveraged HIE to improve the selectivity of the deracemization of substrate *rac*-**1ae**, which under otherwise identical reaction conditions reached 40% ee in the presence of H_2_O (**1ae**) and 72% ee in the presence of D_2_O (**1ae-*d*_1_**) with 99% deuterium incorporation. The origin of this effect is discussed below.

In all cases, reactions proceeded with high levels of recovery (78–99%), with an average yield of 95%. The absolute configuration of (*S*)-**1t** was determined by X-ray crystallography (see Supporting Information); the configurations of all other substrates were assigned by analogy. Finally, 1.0 g (4.6 mmol) scale-up of the model reaction with **1a** at reduced photocatalyst and buffered phosphate co-catalyst loadings afforded **1a** in 91% yield and 95% ee after 3 h of irradiation.

### Mechanistic Studies

In our proposed catalytic cycle, we hypothesized that stereoselectivity could arise from either a kinetic resolution in the stereoablative deprotonation of radical cation **1-INT-1** or a stereoselective C–H bond forming step, or a combination of both. To guide our mechanistic studies, we defined the kinetic scheme shown in Figure 3A and derived corresponding steady-state expressions that relate catalyst concentrations and elementary rate constants to the concentrations of (*R*) and (*S*) configurations of **1**.^6^ Under this treatment, the enantiomeric ratio at the photostationary state of the reaction can be expressed as the product of three terms (Figure 3B). Term A describes the competition between deprotonation of **1-INT-1** and BET from the reduced photocatalyst **Ir** ^**II**^ for each enantiomer, while Term B directly relates the rates of deprotonation of each enantiomer of radical cation **1-INT-1** by the **CPB**. Together, Terms A and B describe the selectivity of a kinetic resolution in the C–H bond cleavage. Notably, as the rate of BET increases, the contribution to selectivity from differential rates of **1-INT-1** deprotonation also increases. Finally, Term C is the enantioselectivity of the C–H bond-forming step from the prochiral radical **1-INT-2**. This analysis indicates that BET, deprotonation, and C–H bond formation may each influence the enantioselectivity of the deracemization reaction. We therefore designed experiments to determine the mechanisms of C–H bond cleavage and re-formation, and to determine how each contributes to the overall selectivity of deracemization (Figure 3C).

**Figure 3:**
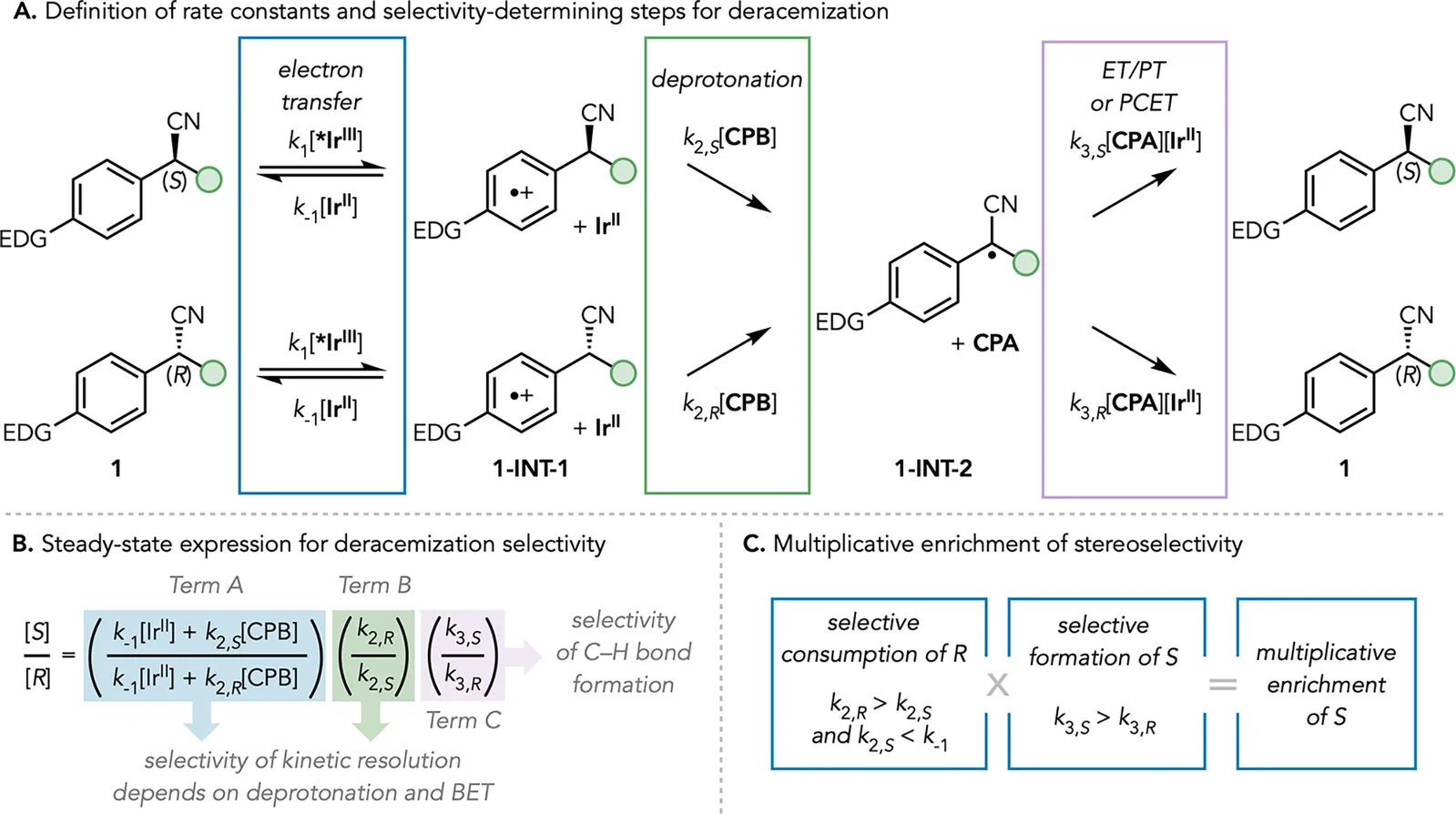
(A) Definition of rate constants and selectivity-determining steps for deracemization. (B) Expression for the extent of deracemization at the photostationary state. (C) Relative rate constants required for multiplicative enrichment of stereochemistry.

### Dependence of Enantioselectivity on Photocatalyst

Our initial studies focused on determining the mechanism of C–H bond formation and its contribution to the enantioselectivity of the deracemization reaction. In our previous work on the deracemization of α-aryl ketones, a strong correlation between ground-state photocatalyst reduction potential and the ee of C–H bond formation was observed.^6^ This was taken as evidence against a stepwise ET/PT mechanism involving stereoselective protonation of a discrete carbanion intermediate by the **CPA**, in which the stereoselectivity would not be expected to vary with redox properties of the photocatalyst. Instead, we proposed a stereoselective reductive PCET mechanism, in which a proton and electron are delivered simultaneously to the substrate radical to form a new C–H bond. This mechanism would involve the reduced photocatalyst in the stereodetermining transition state assembly, and the driving force for C–H bond formation would vary directly with the reducing power of the catalyst. We hypothesized that the observation of a similar trend may shed light on the operative mechanisms of C–H bond formation in the deracemization of benzylic nitriles.

To this end, we measured the ee at the photostationary state (ee_PS_) of the reaction with a range of photocatalysts **Ir-1–Ir-10** exhibiting varying ground-state reduction potentials (−1.77 V to −1.11 V vs Fc^+^/Fc in MeCN, see Supporting Information for photocatalyst structures). In parallel experiments, *rac*-**1a** and enantiopure (*S*)-**1a** were each subjected to the deracemization conditions until the enantioselectivity for both reactions converged to a common value, which was taken as ee_PS_. Plotting ee_PS_ for **1a** against the Ir^III^/Ir^II^ reduction potential revealed two distinct regimes (Figure 4). For photocatalysts more reducing than the reduction potential of substrate **1a**-derived radical **1-INT-2** (*E*° = −1.44 V vs Fc^+^/Fc in MeCN), the value of ee_PS_ was largely constant. However, above this threshold (*E*_1/2_(Ir^III^/Ir^II^) > −1.44 V), the value of ee_PS_ increased with increasingly positive ground-state photocatalyst reduction potential. Notably, no clear trend was observed when the ee_PS_ of **1a** was plotted against the excited-state reduction potential of the same photocatalysts (+0.85 V to +1.43 V vs Fc^+^/Fc in MeCN, see Supporting Information).

**Figure 4:**
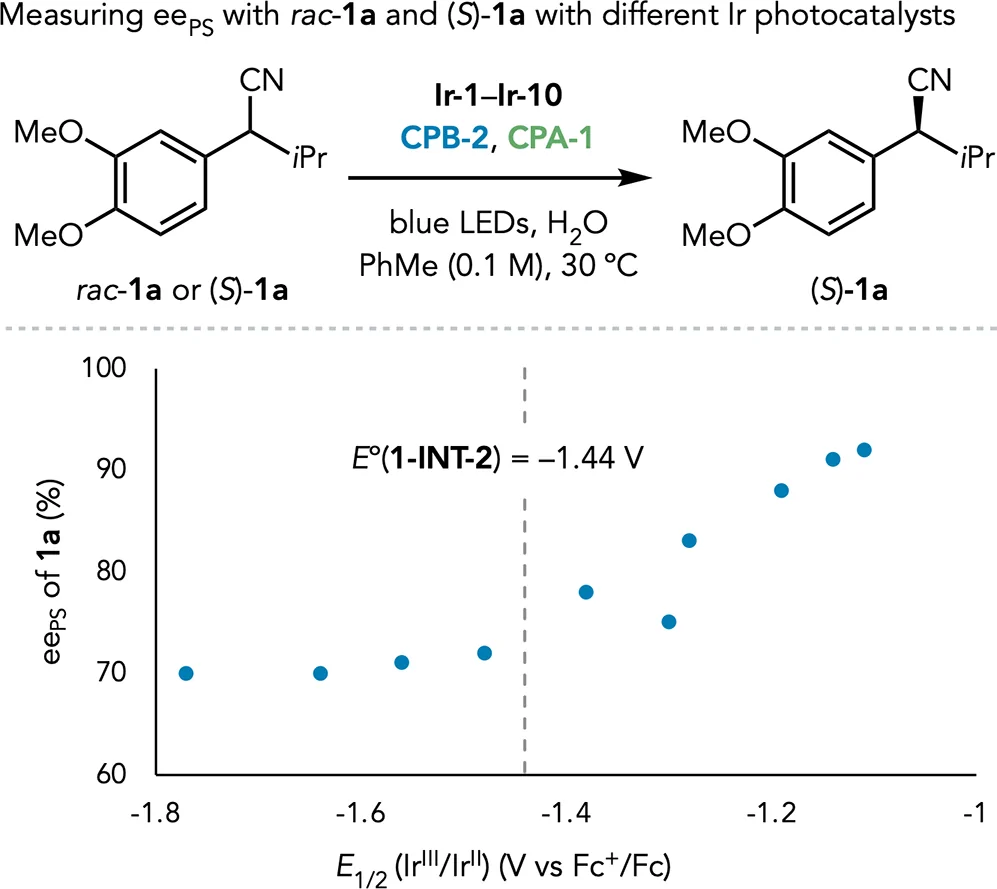
Effect of the ground-state reduction potential of the photocatalyst on ee_PS_ in the deracemization of **1a**. In all cases, yield was >80%. See Supporting Information for structures of **Ir-1**–**Ir-10**.

Although a dependence of the enantioselectivity of C–H bond formation (Figure 3B, Term C) on photocatalyst reduction potential is consistent with a reductive PCET mechanism, we recognized that the selectivity of a kinetic resolution could also be sensitive to the redox properties of the photocatalyst. Specifically, the rate of BET may vary with photocatalyst,^16^ as reduction potential effectively modulates the lifetime of the key radical cation intermediate (Figure 3B, Term A). To further decouple these effects, we aimed to quantify the influence of photocatalyst reduction potential on the enantioselectivity of C–H bond formation in isolation.

To this end, we sought to generate a structurally analogous radical intermediate to **1-INT-2** that could undergo productive C–H bond formation under catalytically relevant conditions, and the product of which would not undergo deracemization. Building on our previous work,^6^ we reasoned that cyanoether **2** could generate all three key intermediates involved in C–H bond formation—α-cyano radical **2-INT-2**, **Ir** ^**II**^, and **CPA-1**—under the standard reaction conditions for deracemization (Figure 5A). Upon blue light irradiation, oxidation of the electron-rich arene of **2** (*E*_p/2_ = +1.01 V vs Fc^+^/Fc in MeCN) by an excited-state photocatalyst ^*^ **Ir** ^**III**^ followed by deprotonation of the acidified benzylic C–H bond by **CPB-2** yields radical **2-INT-1** and **CPA-1**. This intermediate fragments via β-scission to generate benzylic α-cyano radical **2-INT-2** and aldehyde **4**. Finally, ET/PT or reductive PCET to **2-INT-2** generates product nitrile **3**. Due to its significantly higher oxidation potential (*E*_p/2_ = +2.09 V vs Fc^+^/Fc in MeCN), we reasoned that **3** would be configurationally stable under these conditions. Subjection of an authentic sample of *rac*-**3** to the standard reaction conditions with D_2_O instead of H_2_O resulted in no deracemization and no deuterium incorporation, confirming that the substrate does not react with the most oxidizing photocatalyst (**Ir-1**) employed in this study (see Supporting Information). Moreover, the formation of **2-INT-2** occurs by a catalytic oxidation and deprotonation sequence that closely mirrors the conditions and concentrations under which **1-INT-2** is formed. Notably, subjecting *rac*-**1a** to the standard deracemization conditions with an equivalent of aldehyde **4** demonstrated that this stoichiometric byproduct does not significantly affect enantioselectivity (see Supporting Information). Thus, we propose that the ee of product **3** should report solely on the selectivity of the C–H bond forming step.

**Figure 5:**
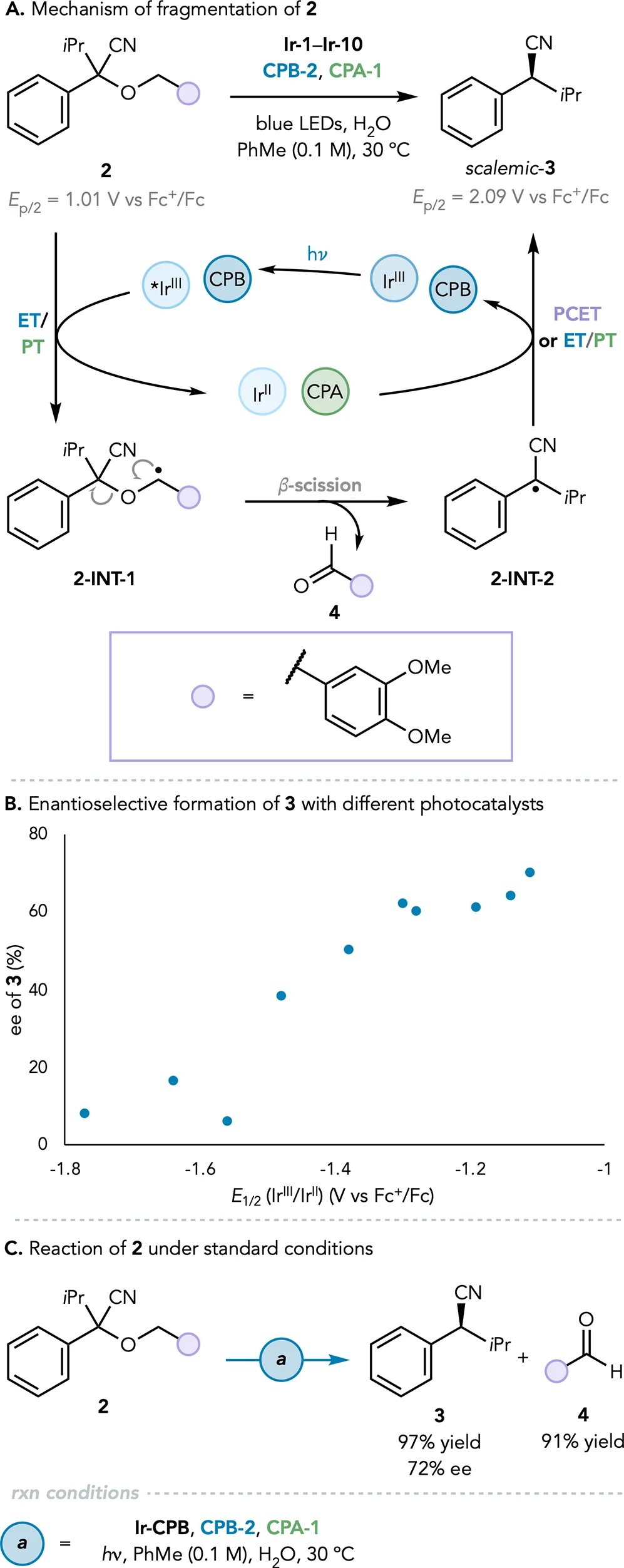
(A) Mechanism of fragmentation of cyanoether **2** to form nitrile **3**. (B) Enantioselectivity in the C–H bond formation of **3** varies with photocatalyst ground-state reduction potential. (C) Reaction of **2** under standard conditions.

The enantioselectivity of the formation of **3** was then evaluated by subjecting cyanoether **2** to standard reaction conditions for deracemization with the same panel of photocatalysts **Ir-1**–**Ir-10** (Figure 5B). In all cases, products **3** and **4** were formed in nearly equal yield with quantitative mass balance. The measured ee values varied widely between photocatalysts, from 6 to 70% ee. A strong correlation was observed between ground-state reduction potential and the ee of product **3**, wherein increased stereoselectivity was observed for photocatalysts with more positive ground-state reduction potentials.^17^ Again, these data are inconsistent with an exclusively stepwise ET/PT mechanism for C–H bond formation, which would exhibit photocatalyst-independent enantioselectivity.

To directly assess whether stereoselective protonation of a keteniminate could account for the observed enantioselectivity, the tetramethylphosphonium keteniminate derived from **1a** was generated independently and protonated with **CPA-1** (see Supporting Information). Under these stoichiometric conditions, protonation afforded racemic product. Thus, protonation of an equilibrated keteniminate is not stereoselective and cannot account for the levels of enantioinduction observed in the deracemization reaction. Together with the redox-potential dependence observed in Figures 4 and 5, these observations suggest a mechanism of C–H bond formation that is distinct from stepwise ET/PT.

To account for the results above, we propose that asymmetric C–H bond formation occurs via reductive PCET, with two possible explanations for the dependence of enantioselectivity on ground-state photocatalyst redox potential. In one scenario, the less reducing photocatalysts favor a concerted and stereoselective reductive PCET pathway, while more reducing photocatalysts progressively shift the mechanism toward a stepwise and nonselective ET/PT pathway; intermediate selectivity values across the photocatalyst series would then reflect competition between these two mechanisms. Alternatively, as the reaction becomes more exergonic, the transition state of reductive PCET shifts to a more reactant-like geometry, degrading stereocontrol without any change in mechanism.^18^ Given the onset of a potential-independent regime around the threshold for direct reduction of **1-INT-2**, we favor the former explanation.

Further analysis of the results with cyanoether **2** shed light on other notable features of the reaction. Upon subjecting **2** to the optimal conditions for deracemization, nitrile **3** was formed in 97% yield and 72% ee, along with aldehyde **4** in 91% yield (Figure 5C). The selectivity for this transformation is markedly lower than the selectivity observed in the deracemization of **1a** (94% ee) under analogous conditions. While the attenuation of electron density on the α-cyano arene may be a factor in this diminished stereoselectivity, the significant difference in ee indicates that stereoselective C–H bond formation alone cannot account for the overall selectivity of deracemization, suggesting the participation of an additional stereoselective step.

We also observed that the most reducing photocatalyst **Ir-10** ([Ir­(dF­(CF_3_)­ppy)_2_(4,4′-dtbbpy)]­PF_6_, *E*_1/2_(Ir^III^/Ir^II^) = −1.77 V vs Fc^+^/Fc in MeCN)^19^ formed **3** from **2** in only 8% ee (Figure 5B). In contrast, the ee_PS_ in the deracemization of **1a** with the same photocatalyst was measured to be 70%. From the low stereoselectivity of the C–H bond-forming step with **Ir-10**, we propose that the ee_PS_ values obtained for deracemization with this photocatalyst must largely reflect the selectivity of a stereoablative kinetic resolution step. As such, we reasoned that photocatalyst **Ir-10** could be used to study the selectivity of the kinetic resolution component of the deracemization in isolation (*vide infra*).

### Low-Conversion Deuterium Incorporation Studies

With evidence suggesting that deracemization occurs via multiple stereoselective steps, we next sought to quantify the contribution of each step under catalytically relevant conditions. Recently, Wendlandt and co-workers reported a low conversion deuterium incorporation strategy for estimating the facial diastereoselectivity of hydrogen atom donation (HAD) in the epimerization of carbohydrates.^20^ We envisioned that this framework could be adapted to the analysis of deracemization reactions. Starting with the minor enantiomer (*R*)-**1a**, HIE epimerization reactions were conducted in the presence of D_2_O to low conversion (<5 min irradiation; <15% conversion) to form (*S*)-**1a**-***d***_**1**_. Subsequent chromatographic separation of enantiomers and analysis of deuterium incorporation in each enantiomer by ^1^H NMR spectroscopy enabled the measurement of the selectivity of the C–H bond-forming step (see Supporting Information). Our kinetic model indicates that the observed enantiomeric ratio (Figure 6, er_obs_) in deracemization is a direct product of the selectivity of the kinetic resolution (er_1_) and the selectivity of C–H bond formation (er_2_). Using the experimental er_obs_ and the calculated er_2_ for C–H bond formation from the deuterium incorporation experiments, we could then calculate the selectivity er_1_ of the kinetic resolution.

**Figure 6:**
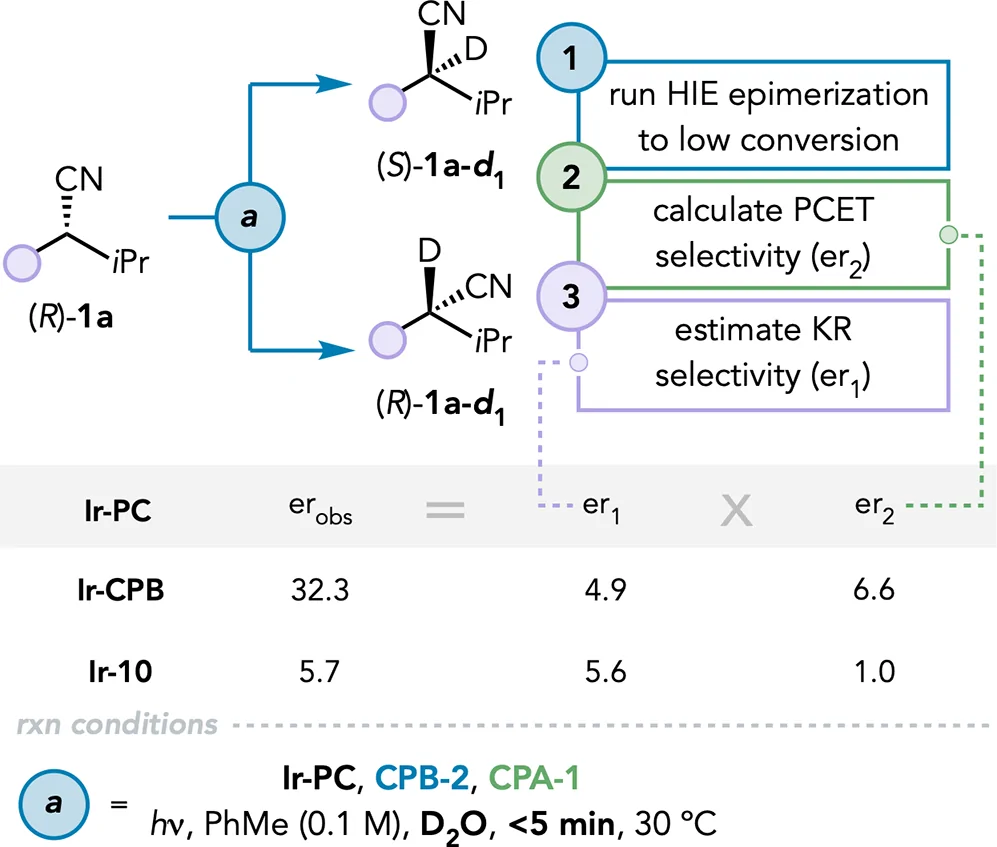
Estimated selectivity of C–H bond formation (er_2_) and KR (er_1_) based on low-conversion deuterium incorporation experiments and observed er in deracemization (er_obs_).

When low-conversion deuterium incorporation experiments were performed with **Ir-CPB** as photocatalyst (Figure 6), the enantioselectivity of the C–H/D bond-forming step was estimated to be 6.6:1 (74% ee). This value is in excellent agreement with the 6.1:1 er (72% ee) experimentally observed in the formation of **3** from cyanoether **2** (Figure 5C), indicating that **2** is a reliable probe for the selectivity of the C–H bond-forming step. Given an overall er of 32.3:1 (94% ee) in the deracemization of **1a** under these conditions, the selectivity of the kinetic resolution was estimated to be 4.9:1.

Moreover, when analogous low-conversion deuterium incorporation experiments were conducted with **Ir-10** as photocatalyst, we determined the selectivity of C–H/D bond formation to be 1.0:1. This value agrees well with the experimentally observed 1.2:1 er (8% ee) in the formation of **3** from cyanoether **2** with **Ir-10** as photocatalyst (Figure 5B) and is consistent with the lack of enantioselectivity observed in the stoichiometric protonation of pre-formed keteniminate. These data further support the hypothesis that deracemization with **Ir-10** as photocatalyst occurs largely by kinetic resolution. From the overall stereoselectivity of deracemization of **1a** with **Ir-10** measured to be 5.7:1 (70% ee), the er of the kinetic resolution was estimated to be 5.6:1.

Notably, the selectivity values obtained from cyanoether fragmentation, low-conversion deuterium incorporation studies with **Ir-CPB** and **Ir-10**, and stoichiometric protonation experiments are consistent, providing a coherent picture of selectivity partitioning across the deracemization network. These experiments demonstrate that the high enantioselectivity observed in the deracemization reflects the multiplicative combination of two moderately selective steps for C–H bond cleavage and re-formation that are stereochemically matched and mediated by the same chiral catalyst.

### Hydrogen Isotope Exchange

With strategies in hand to study the component steps of the reaction independently, we next investigated the origins of the isotopic perturbation of enantioselectivity in the deracemization reaction. As previously noted, we observed that exchanging H_2_O for D_2_O under otherwise standard conditions led to a large difference in er (Figure 7A, entry **1a-*d*_1_**). Similar cases of isotope-dependent enantioselectivity have been observed previously.^21^

**Figure 7:**
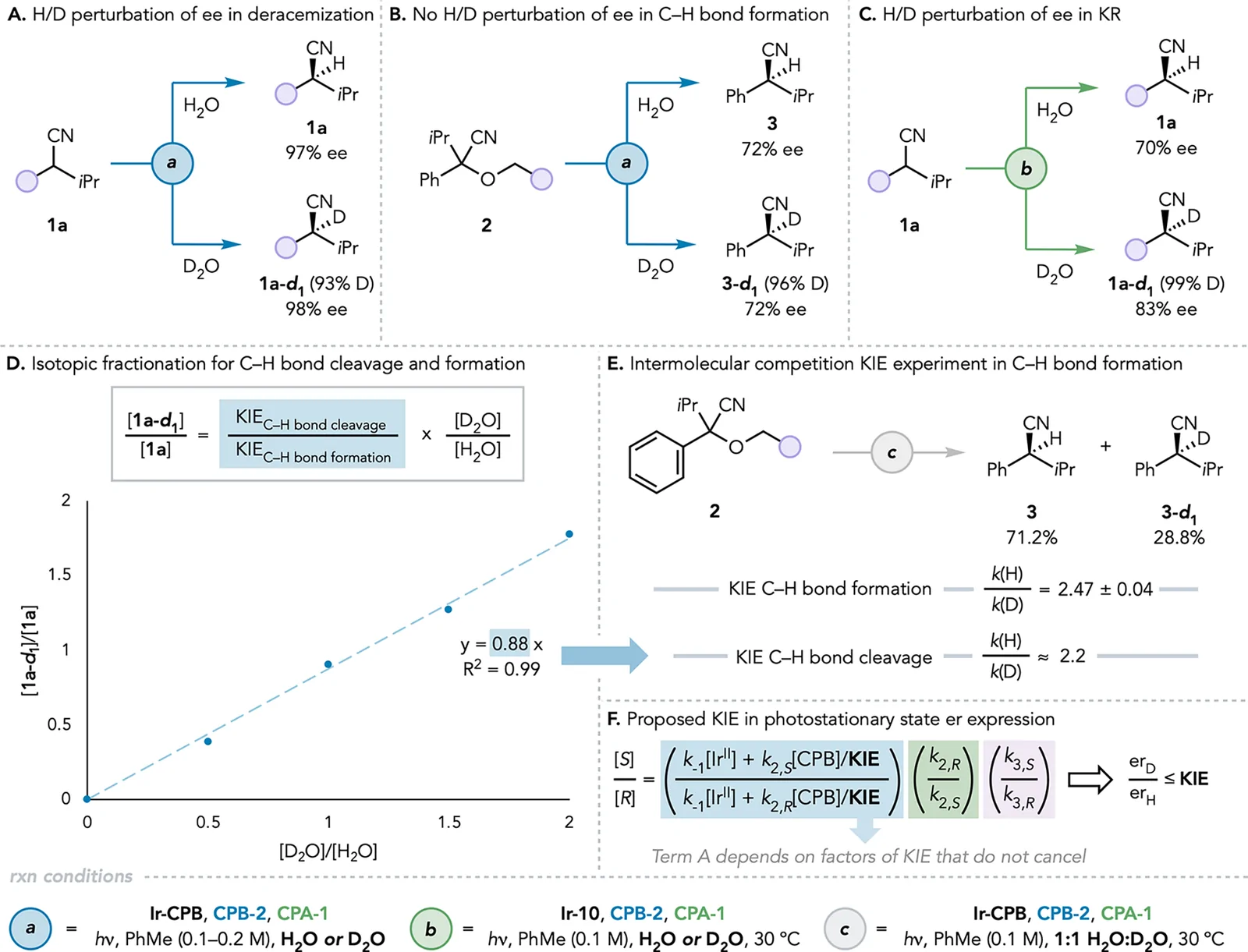
(A) Example of isotopic perturbation of enantioselectivity with D_2_O. (B) Parallel experiments with H_2_O and D_2_O with mechanistic probe **2**. (C) Parallel experiments with H_2_O and D_2_O with **Ir-10** and **1a**. (D) Isotopic fractionation experiments in deracemization of **1a** with 2 mol % **Ir-CPB**, 2.5 mol % **CPB-2**, 2.5 mol % **CPA-1**, and varying ratios of D_2_O:H_2_O. (E) Competition KIE experiment with **2** and 1:1 H_2_O:D_2_O. (F) Proposed KIE in the photostationary state er expression.

Initially, we questioned whether the selectivity of the C–H bond forming step was sensitive to isotopic substitution in the **CPA** proton donor (O–H vs O–D). However, subjecting cyanoether **2** to the standard reaction conditions with either H_2_O or D_2_O as cosolvent resulted in identical levels of enantioselectivity in the formation of **3** (Figure 7B). This result suggests that isotopic perturbation of enantioselectivity does not occur in the C–H bond-forming step of deracemization. Thus, we questioned whether the selectivity of the kinetic resolution step could improve upon substrate deuterium incorporation. Consistent with this hypothesis, we observed significantly greater deracemization of **1a** with **Ir-10** in the presence of D_2_O (83% ee) than with H_2_O (70% ee).

We reasoned that the origin of this effect could be traced back to the relative rates of BET and deprotonation, which would be affected by a primary KIE in the deprotonation of radical cation **1-INT-1**. Thus, we looked to experimentally evaluate the magnitude of the KIE in the deprotonation of **1-INT-1**. While challenging to measure directly, we reasoned that an estimate could be obtained from a fractionation protocol previously developed by our lab to study photoredox-based hydrogen isotope exchange reactions.^22,23^ In this approach, the slope of a plot of substrate deuterium incorporation ([**1a-*d*_1_**]/[**1a**]) against solvent deuterium content ([D_2_O]/[H_2_O]) gives the equilibrium isotope effect, equal to the ratio of the KIEs for C–H bond cleavage and bond formation. When isotopic fractionation experiments were performed with **1a** with varying D_2_O/H_2_O cosolvent ratios, the fractionation slope was determined to be 0.88 (*R* ^2^ = 0.99), indicating that the KIE for C–H cleavage is smaller than that of bond formation (Figure 7D). To estimate the KIE of the C–H bond-forming step, cyanoether **2** was subjected to an intermolecular competition experiment with 1:1 H_2_O/D_2_O as cosolvent (Figure 7E). From the isotopic composition of the products, the KIE for the formation of **3** was measured to be 2.47 ± 0.04. Assuming a similar magnitude KIE for C–H bond formation in the deracemization reaction, these data suggest a primary KIE of ∼2.2 for the deprotonation of **1-INT-1**.

While this value is only an approximation, a primary KIE of similar magnitude would be sufficient to impart significant isotopic perturbation of enantioselectivity in the deracemization reaction. Following initial substrate processing that exchanges hydrogen for deuterium at the α-position of **1**, a higher barrier to the deprotonation of **1-INT-1-*d*_1_** manifests as non-cancellable KIE factors in Term A of the expression for the photostationary state er (Figure 7F, bold). Indeed, it can be shown that the magnitude of the improvement in er upon H-to-D substitution is bounded by the KIE (see Supporting Information for derivation). While the possibility of non-degenerate KIEs for the deprotonation of **1-INT-1** enantiomers cannot be ruled out as an origin of the isotopic perturbation of enantioselectivity, this analysis provides a plausible rationale for these effects that follows directly from the proposed mechanistic framework for deracemization.^24^

### Non-Classical Nonlinear Effects

Finally, seeking further experimental evidence for our proposed mechanism, we questioned whether deracemization reactions with sequential stereoselective steps and a single chiral catalyst might exhibit a non-classical nonlinear effect (NLE). Fu^25^ and Blackmond^26^ have demonstrated that multi-step reactions featuring a single chiral catalyst can give rise to NLEs without invoking higher-order catalyst species or dual catalyst activation.^27^ Indeed, upon subjecting *rac*-**1a** to modified deracemization conditions (omitting **CPA-1**) with scalemic catalyst mixtures, a modest but reproducible positive nonlinear effect was observed (Figure 8A).^28^

**Figure 8:**
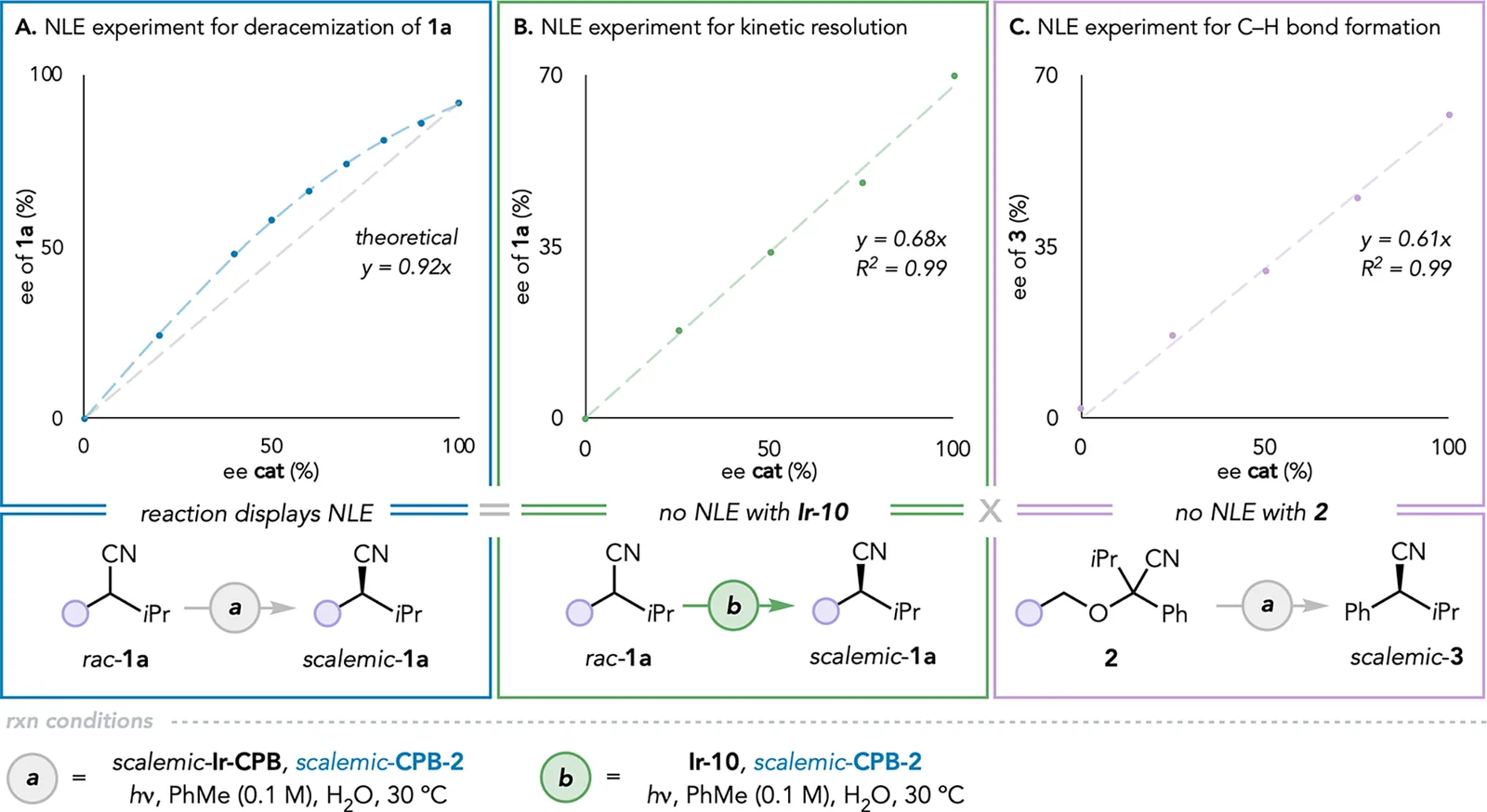
(A) Observation of nonlinear effect in deracemization of **1a**. (B) Linear dependence of product ee on catalyst ee in deracemization of **1a** with **Ir-10**. (C) Linear dependence of product ee on catalyst ee in the reaction of **2**.

To better understand the origins of the observed NLE, we examined whether the C–H bond cleavage or formation steps individually exhibit a positive NLE. To study the kinetic resolution step, **Ir-10** was used as photocatalyst; while to study the C–H bond forming step, cyanoether **2** was used as the substrate (*vide supra*). Subjecting *rac*-**1a** to deracemization with **Ir-10** and scalemic **CPB-2** resulted in a linear relationship between the ee of **1a** and the ee of the co-catalyst **CPB-2** (Figure 8B). Similarly, the reaction of cyanoether **2** to give nitrile **3** with scalemic **Ir-CPB** and **CPB-2** resulted in a linear dependence of the ee of **3** on the ee of the catalysts (Figure 8C). These results demonstrate that no NLE is present in either the kinetic resolution step or the C–H bond-forming step in isolation. These observations are therefore inconsistent with aggregation or higher-order involvement of the chiral phosphate co-catalyst in the deracemization reaction, in either protonation state.^29^

To rationalize the observed nonlinear behavior, we note that the observed er of multistep deracemization reactions can be represented by the product of the er of each individual step (eq 1).^3b^ Assuming a linear response of product ee to catalyst ee for both terms in eq 1 results in eq 2, which features nonlinear dependence on the ee of the chiral catalyst in the absence of aggregation effects (see Supporting Information for derivation).

1 $$er_{obs}=er_{1}\times er_{2}$$

2 $$ee_{NLE}=\frac{ee_{cat}\left(ee_{1}+ee_{2}\right)}{1+ee_{1}\times ee_{2}\times ee_{cat}^{2}}$$

In addition to qualitatively accounting for the observed NLE, we believe this analysis could be useful for the study of deracemization reactions that proceed with a single chiral catalyst, particularly in cases where selectivity of individual steps cannot be independently measured by other means.^30^ In the limiting case where only a single stereoselective step contributes to deracemization, eq 2 collapses to a linear relationship and no NLE is predicted. Alternatively, observation of a non-classical nonlinear effect can be taken as support for multiplicative stereocontrol when appropriate controls are conducted to rule out aggregation-based effects.

## Conclusions

We have developed a light-driven deracemization of benzylic nitriles that proceeds with high yield and stereoselectivity through photoredox and chiral phosphate co-catalysis. We observed that the overall enantioselectivity of the reaction exhibits a dependence on the ground-state reduction potential of the photocatalyst. Independent generation of a key radical intermediate isolated this dependency to the C–H bond forming step of catalysis, consistent with an enantioselective reductive PCET mechanism for C–H bond formation. Subsequent low conversion deuterium incorporation experiments demonstrated that a stereochemically matched kinetic resolution also contributes to the high selectivity observed in the deracemization reaction. Additionally, this system displays a pronounced isotopic perturbation of enantioselectivity, wherein higher levels of enantioenrichment are obtained when D_2_O is used in place of H_2_O as an additive. The origin of this effect was attributed to a primary KIE in the deprotonation of an arene radical cation intermediate, which increases the selectivity of the kinetic resolution of the substrate. Finally, we observed a non-classical nonlinear effect that was attributed to the participation of a single chiral catalyst in two distinct and sequential stereoselective steps in the absence of aggregation effects.

Several features of this work may find broader application. Enantioselective reductive PCET to carbon-centered radicals could offer a novel manifold for the construction of stereogenic methine centers. In such reactions, the ground-state potential of the photocatalyst represents an additional variable for optimization that is independent from the structure of the chiral proton donor. Moreover, we hope that the mechanistic experiments and analyses disclosed herein may prove to be general towards the study and development of new photoredox-mediated deracemization reactions. Particularly, methods to determine how stereoselectivity is partitioned across bond-breaking and bond-forming events could facilitate the rational design of better catalysts for these unique reactions.

## Supplementary Material



## Notes

Experimental details, crystallographic and computational information, NMR spectra, and HPLC traces (PDF)
