# Supplementary material for: Multiplicative Stereocontrol in the Light-Driven Deracemization of Benzylic Nitriles
Source: J Am Chem Soc. 2026 Sep 24;148(39):42695–706. doi: 10.1021/jacs.6c15890 (PMC13651525; doi:10.1021/jacs.6c15890)
Supplement: Supplementary file 1 [file ja6c15890_si_001.pdf]

Supporting Information for:

Multiplicative Stereocontrol in the Light-Driven Deracemization of Benzylic Nitriles

Y. Emily Du,[#] Alexander S. Hurlburt,[#] Justin Y. Wang, Qiaolin Yan, Robert R. Knowles[*]

Department of Chemistry, Princeton University, Princeton, NJ 08544

Corresponding Author:

*rknowles@princeton.edu

1. General Information 2

2. Synthesis of Catalysts 3

3. Synthesis of Racemic Benzylic Nitriles 8

4. Cyclic Voltammetry Experiments 30

5. Deracemization of Benzylic Nitriles 33

6. Determination of Photostationary State ee with Photocatalysts **Ir-1**–**Ir-10** 90

7. Synthesis of Mechanistic Probe **2** 92

8. Control Experiments with Nitrile **3** 94

9. Mechanistic Experiments with Cyanoether **2** 96

10. Stoichiometric Protonation Experiments with **CPA-1** 102

11. Low-conversion Deuterium Incorporation Experiments 106

12. Determination of KIE for Deprotonation of **1-INT-1** 118

13. Non-linear Effects 122

14. Determination of Initial Rates and Catalyst Order 132

15. Photocatalyst Excited-State Lifetime Measurements 136

16. Stern-Volmer Luminescence Quenching Experiments and Photocatalyst-Substrate Association 139

17. X-Ray Crystallography Data 142

18. Computational Details 148

19. Unsuccessful Substrates 167

20. NMR spectra of catalysts, substrates, and deracemized products 168

21. References 247

1. General Information

Reagents were purchased from commercial vendors and were used as received unless otherwise noted. Anhydrous solvents were either used directly from a Sigma-Aldrich SureSeal™ container or purified according to the method of Grubbs.[1] Chromatographic purification of compounds was accomplished by automated flash chromatography using Biotage® Selekt or Biotage® Enkel instruments with 25 or 50 g silica gel columns (Silicycle F60 silica gel) according to the method of Still.[2] Preparative supercritical fluid chromatography (SFC) was performed by Lotus Separations, LLC and was conducted using a Berger Multi-gram II SFC instrument equipped with two Varian SD-1 pumps, a Knauer K-2501 spectrophotometer, and a 6-ton bulk CO_2 tank with a built-in chiller, heater, and G700 compressor. Thin-layer chromatography (TLC) was performed on Silicycle 250 μm silica gel plates. Visualization of the developed chromatogram was performed by irradiation with UV light or treatment with a solution of potassium permanganate stain followed by heating. Yields refer to purified compounds unless otherwise noted.

^{1}H, ^{13}C, ^{19}F, and ^{31}P NMR spectra were recorded on Bruker 500 instruments, unless otherwise noted, and are internally referenced to residual solvent signals by the method recommended by IUPAC.[3] Data for ^{1}H NMR are reported as follows: chemical shift (δ ppm), integration, multiplicity (s = singlet, d = doublet, t = triplet, q = quartet, p = pentet, sext = sextet, hept = septet, m = multiplet, brs = broad singlet), and coupling constant (J in Hz). Data for ^{13}C NMR are reported as follows: chemical shift (δ ppm), multiplicity (s = singlet, d = doublet, t = triplet, q = quartet, p = pentet, sext = sextet, hept = septet, m = multiplet, brs = broad singlet), and coupling constant (J in Hz), and no special nomenclature is used for equivalent carbons. ^{19}F NMR are referenced to fluorobenzene (PhF referenced at δ = -113.15 ppm). ^{31}P NMR spectra are referenced to triphenylphosphine (PPh_3 referenced at δ = -6.00 ppm). IR spectra were recorded on a Thermo Scientific Nicolet 6700 FT-IR spectrometer and are reported in terms of wavenumber (cm^{-1}). High resolution mass spectra were obtained at Princeton University mass spectrometry facilities using an Agilent 6230 TOF LC/MS. Enantiomeric ratio (er) was determined by high-performance liquid chromatography (HPLC) performed on an Agilent 1260 Infinity Series LC using commercial ChiralPak®/ChiralCel® columns from Daicel Chemical Industries, notably ChiralPak® AD-H (5 μm particle size, 4.6 mm × 250 mm), ChiralCel® OZ-H (5 μm particle size, 4.6 mm × 250 mm), and ChiralCel® OD-H (5 μm particle size, 4.6 mm × 250 mm), ChiralCel® AS-H (5 μm particle size, 4.6 mm × 250 mm), and ChiralCel® OJ-H (5 μm particle size, 4.6 mm × 250 mm). Tandem LC-SFC analyses were performed with a custom-built instrument: the first dimension is comprised of an Agilent 1290 Infinity III Series LC equipped with an Agilent SB-C18 (1.8 μm particle size, 2.1 × 50 mm) column with a SB-C18 (1.8 μm particle size, 2.1 × 5 mm) pre-column, UV-vis diode array detector, and ASM 2D-LC valve plumbed to a trapping loop; the second dimension is comprised of an Agilent 1290 Infinity III Series SFC equipped with a Daicel ChiralPak® AD-3 (3 μm particle size, 4.6 × 250 mm) chiral column and a UV-vis diode array detector. Optical rotations were measured on a Jasco P-2000 polarimeter at the sodium D-line (589 nm) using a cell of 100 mm path length. The concentration values (c) are reported in g/100 mL.

2. Synthesis of Catalysts

Ir-1 [Ir(dCF$_3$(CF$_3$)ppy)$_2$(5,5'-dCF$_3$-bpy)]PF$_6$

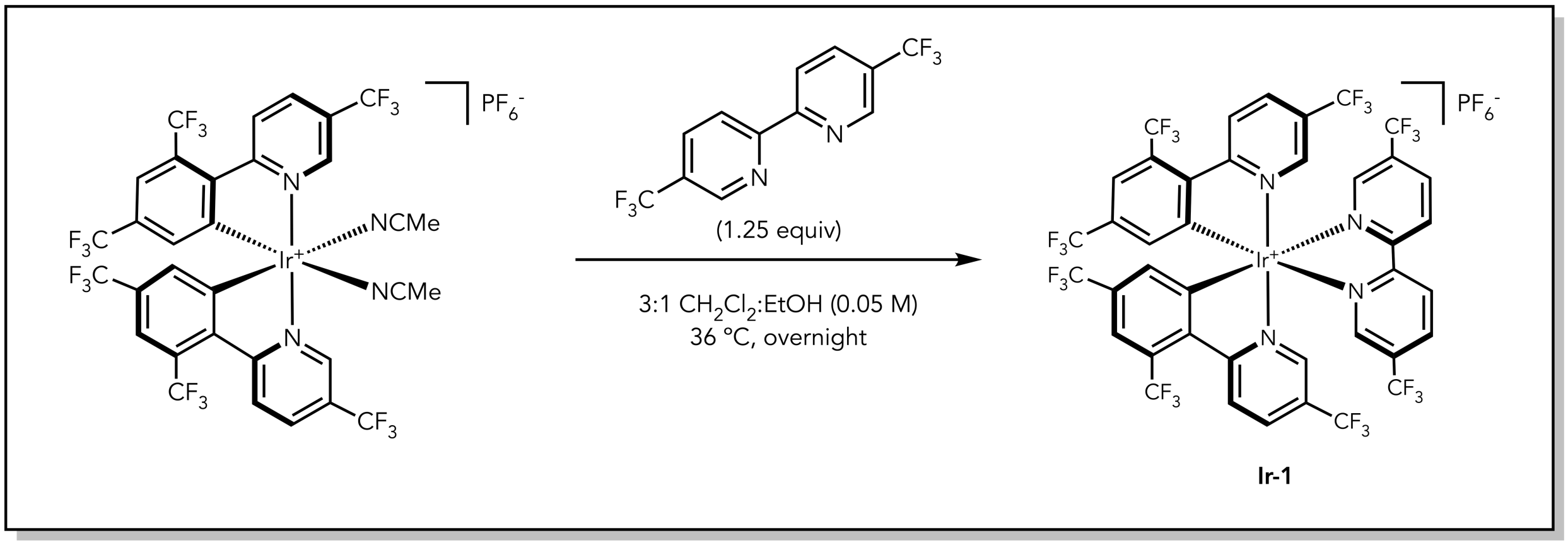

The title compound was prepared according to a modified literature procedure.[4] To a 250 mL round-bottom flask equipped with a magnetic stir bar was added [Ir(dCF$_3$(CF$_3$)ppy)$_2$(MeCN)$_2$]PF$_6$ (3.16 g, 2.78 mmol, 1.00 equiv.), 5,5'-bis(trifluoromethyl)-2,2'-bipyridine (1.02 g, 3.48 mmol, 1.25 equiv.), CH_2Cl_2 (42 mL) and ethanol (14 mL). The reaction mixture was sparged with N_2 for 30 minutes, then heated to 36 °C and stirred overnight. The reaction mixture was cooled to room temperature and filtered to afford the title compound in 74% yield as a bright yellow powder (2.78 g, 2.07 mmol). Characterization data was consistent with reported literature values.[4]

Ir-CPB [Ir(dCF$_3$(CF$_3$)ppy)$_2$(5,5'-dCF$_3$-bpy)][(*S*)-4-Hydroxy-2,6-bis(2,4,6-tri-*iso*-propylphenyl)-dinaphtho[2,1-d:1',2'-f][1,3,2]dioxaphosphepin-4-olate 4-oxide]

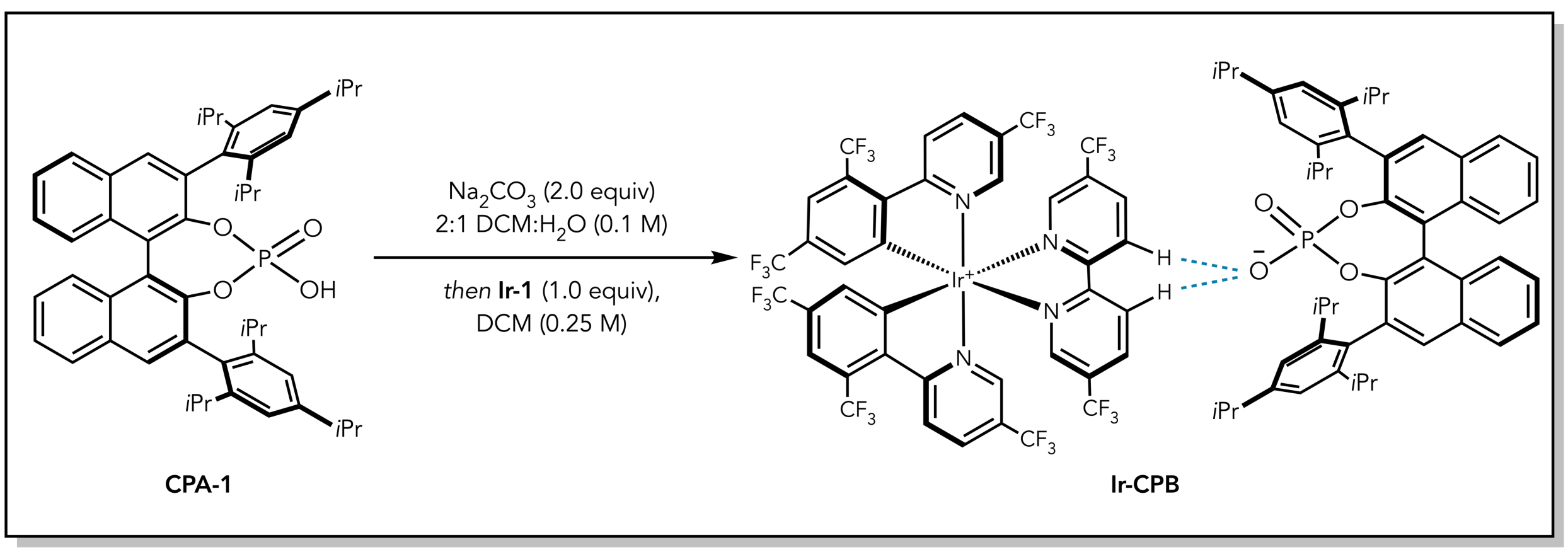

To a 20 mL scintillation vial equipped with a magnetic stir bar was added **CPA-1** (200 mg, 0.27 mmol, 1.0 equiv), Na_2CO_3 (56 mg, 0.53 mmol, 2.0 equiv), CH_2Cl_2 (2.0 mL), and H_2O (1.0 mL). The reaction mixture was stirred for 30 minutes, then concentrated under reduced pressure. The crude material was filtered, washed with water (3 × 10 mL), then dissolved in CH_2Cl_2 (2 mL). **Ir-1** (360 mg, 0.27 mmol, 1.0 equiv) was added, and the reaction mixture was stirred in the dark for 2 h at room temperature. The reaction was transferred to a separatory funnel, diluted with H_2O (5 mL) and the aqueous phase extracted with CH_2Cl_2 (2 × 20 mL). The combined organic layers were

dried over Na_2SO_4, filtered, and concentrated under reduced pressure to afford the title compound in 99% yield (520 mg, 0.27 mmol) as an orange solid. **^{1}H NMR** (500 MHz, $CDCl_3$) δ 10.01 (d, J = 8.6 Hz, 1H), 9.76 (d, *J* = 8.5 Hz, 1H), 8.61 (dd, *J* = 9.0, 4.9 Hz, 2H), 8.26 (td, *J* = 8.6, 2.1 Hz, 2H), 8.13 (dd, *J* = 8.6, 2.1 Hz, 1H), 8.09 (dd, *J* = 8.6, 2.1 Hz, 1H), 7.89 – 7.84 (m, 2H), 7.78 (d, *J* = 10.9 Hz, 3H), 7.58 (t, *J* = 2.8 Hz, 2H), 7.39 (ddd, *J* = 8.0, 6.7, 1.2 Hz, 2H), 7.31 (d, *J* = 8.5 Hz, 2H), 7.27 – 7.20 (m, 5H), 6.95 (d, *J* = 1.8 Hz, 2H), 6.88 (d, *J* = 1.9 Hz, 2H), 6.23 (s, 2H), 3.07 (p, *J* = 6.8 Hz, 2H), 2.80 (p, *J* = 6.8 Hz, 2H), 2.71 (p, *J* = 6.9 Hz, 2H). **^{13}C NMR** (126 MHz, $CDCl_3$) δ 167.03, 158.42, 157.92, 152.80, 152.61, 149.10, 149.02, 148.32, 147.66, 147.24, 146.85, 145.13, 143.01, 139.93, 139.80, 137.28, 134.01, 133.43, 133.09, 132.80, 131.76, 131.56, 131.49, 131.39, 130.64, 130.30, 129.36, 129.07, 128.02, 127.49, 126.23, 125.41, 124.35, 124.12, 123.10, 121.93, 121.22, 120.96, 120.74, 120.40, 119.77, 34.14, 30.91, 30.80, 26.53, 25.05, 24.11, 23.65, 23.60, 23.33. **^{19}F NMR** (470 MHz, $CDCl_3$) δ -57.37 (s, 3F), -57.43 (s, 3F), -62.92 (s, 3F), -62.99 (m, 6F), -63.14 – -63.20 (m, 6F), -64.05 (d, *J* = 6.3 Hz, 3F). **^{31}P NMR** (203 MHz, $CDCl_3$) δ 4.63. **HRMS** m/z M^+ calcd. for $C_{40}H_{16}F_{24}IrN_4^+$: 1201.06155, found 1201.05935 (-1.83 ppm). m/z $[M+H]^+$calcd. for $C_{50}H_{58}O_4P$: 753.40672, found 753.40422 (-3.32 ppm).

Ir-CPB is protonated to form **CPA-1** in 1:1 MeCN/H_2O + 0.1% formic acid solution used for HRMS.

CPB-2 (*S*)-4-Hydroxy-2,6-bis(2,4,6-tri-*iso*-propylphenyl)dinaphtho[2,1-d:1',2'-f][1,3,2] dioxaphosphepin-4-olate 4-oxide tetramethylphosphonium salt

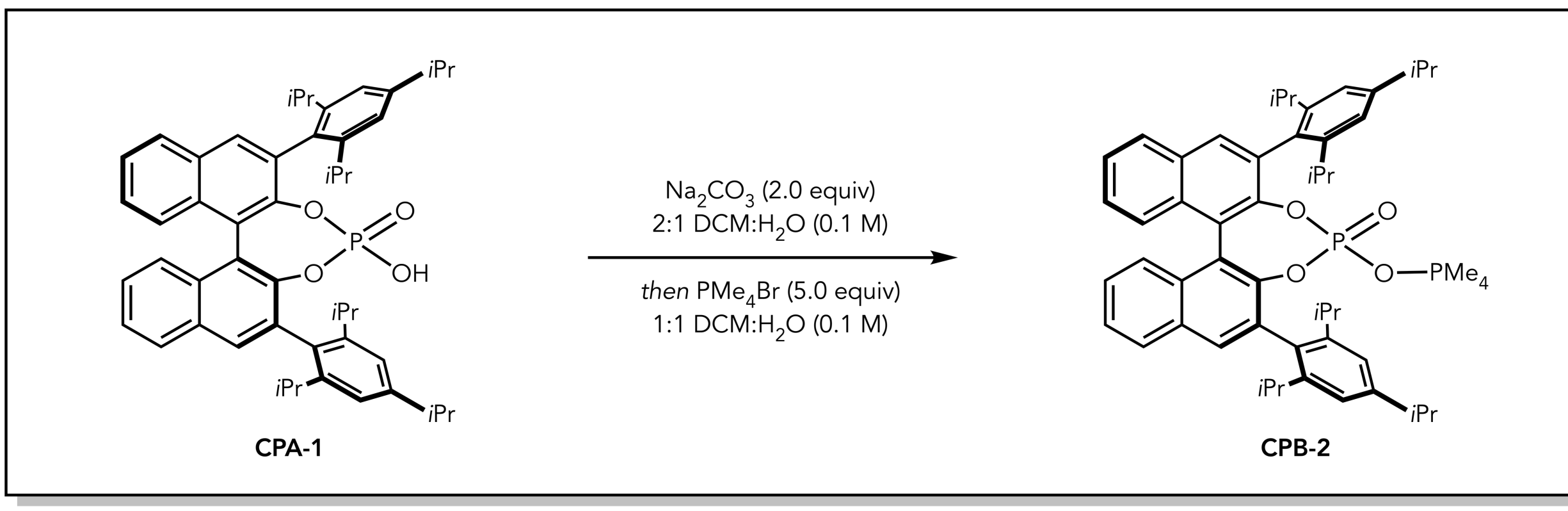

CPA-1 was prepared according to a literature procedure[5] or purchased from Ambeed.

To a 20 mL scintillation vial equipped with a magnetic stir bar was added **CPA-1** (200 mg, 0.27 mmol, 1.0 equiv), Na_2CO_3 (56 mg, 0.53 mmol, 2.0 equiv), CH_2Cl_2 (1.8 mL), and H_2O (0.9 mL). The reaction mixture was stirred for 30 minutes at room temperature, then concentrated under reduced pressure. The resulting white suspension was filtered and washed with water (3 x 10 mL), then suspended in CH_2Cl_2 (1 mL) and H_2O (1 mL). PMe_4Br (230 mg, 1.3 mmol, 5.0 equiv) was added to the reaction mixture and stirred for 30 minutes at room temperature. The mixture was concentrated under reduced pressure and the crude material was filtered and washed with water (3 × 10 mL) to afford the title compound as a white solid in 98% yield (220 mg, 0.26 mmol). **^{1}H NMR** (500 MHz, $CDCl_3$) δ 7.81 (d, *J* = 8.1 Hz, 1H), 7.73 (s, 1H), 7.36 (ddd, *J* = 8.1, 6.4, 1.5 Hz, 1H), 7.25 – 7.17 (m, 2H), 7.07 (d, *J* = 1.8 Hz, 1H), 6.93 (d, *J* = 1.7 Hz, 1H), 3.07 (hept, *J* = 6.7 Hz, 1H), 2.88 (hept, *J* = 6.9 Hz, 1H), 2.66 (p, *J* = 6.8 Hz, 1H), 1.39 – 1.32 (m, 6H), 1.28 – 1.15 (m, 13H), 1.02 (d, *J* = 6.8 Hz, 3H), 0.87 (d, *J* = 6.8 Hz, 3H). **^{13}C NMR** (126 MHz, $CDCl_3$) δ

148.85, 148.78, 147.52, 146.97, 134.51, 133.16, 133.05, 131.43, 130.26, 128.03, 127.15, 125.51, 124.23, 123.01, 121.04, 119.40, 34.33, 30.96, 30.55, 26.89, 25.19, 24.56, 24.48, 23.45, 23.24, 9.85, 9.41. **^{31}P NMR** (162 MHz, $CDCl_3$) δ 22.16 (tt, *J* = 29.6, 14.8 Hz), 3.87. **HRMS** m/z $[M+H]^+$_ calcd. for $C_{50}H_{58}O_4P$: 753.40672, found 753.40470 (-2.68 ppm).

Catalysts **Ir-2–Ir-10** were synthesized according to a literature procedure.[6]

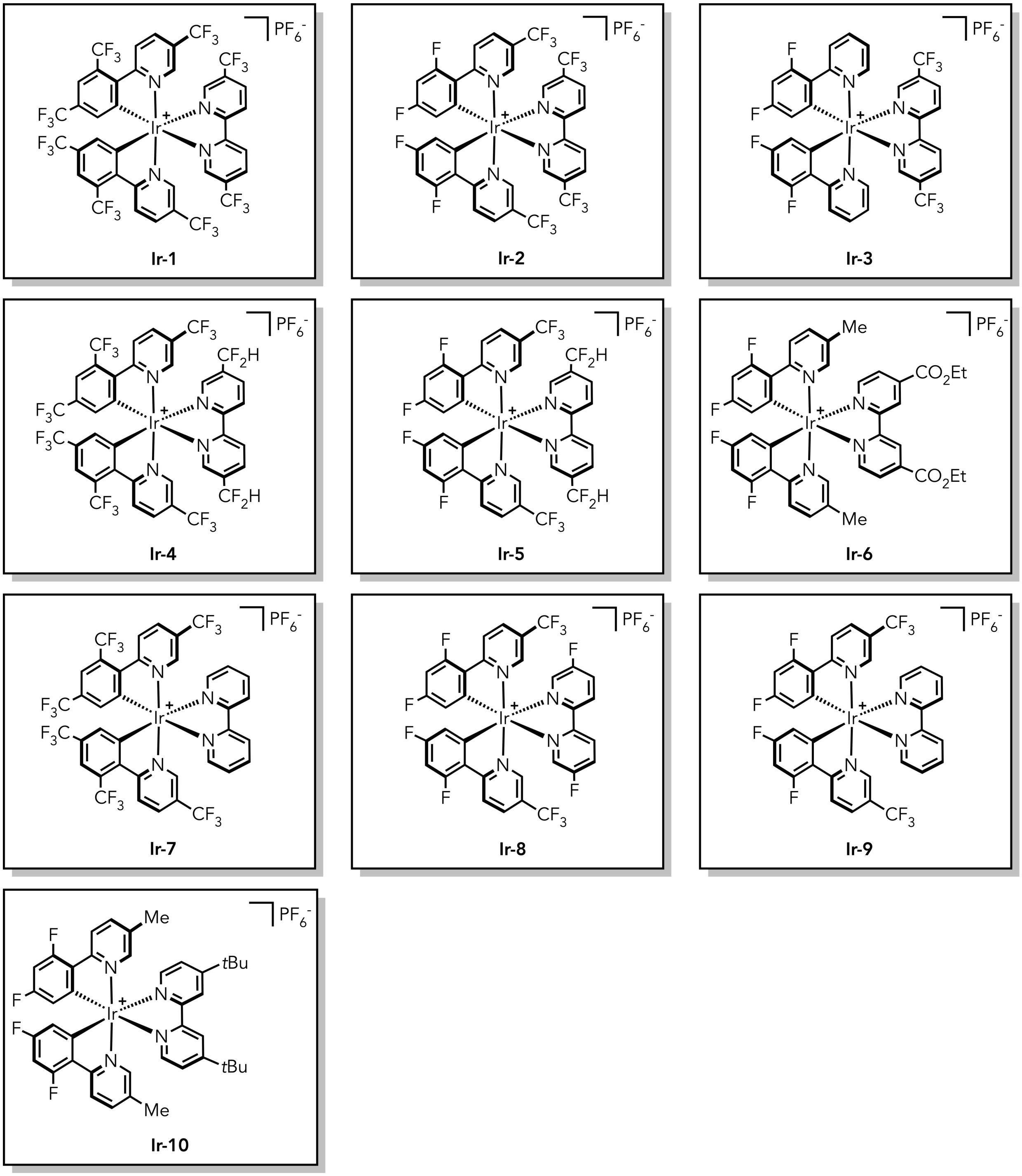

Figure S2.1 Photocatalysts used in this study.

Table S2.1 Photocatalyst redox potentials.

Photocatalyst	$E_{1/2}(Ir^{III}/Ir^{II})$ (V vs Fc^+/Fc)	$E_{1/2}(^*Ir^{III}/Ir^{II})$ (V vs Fc^+/Fc)
**Ir-1**	-1.11	1.43
**Ir-2**	-1.14	1.28
**Ir-3**	-1.19	1.10
**Ir-4**	-1.28	1.28
**Ir-5**	-1.30	1.19
**Ir-6**	-1.38	0.85
**Ir-7**	-1.48	1.09
**Ir-8**	-1.56	1.21
**Ir-9**	-1.64	1.04
**Ir-10**	-1.77	0.98

3. Synthesis of Racemic Benzylic Nitriles

3.1 General Procedure A

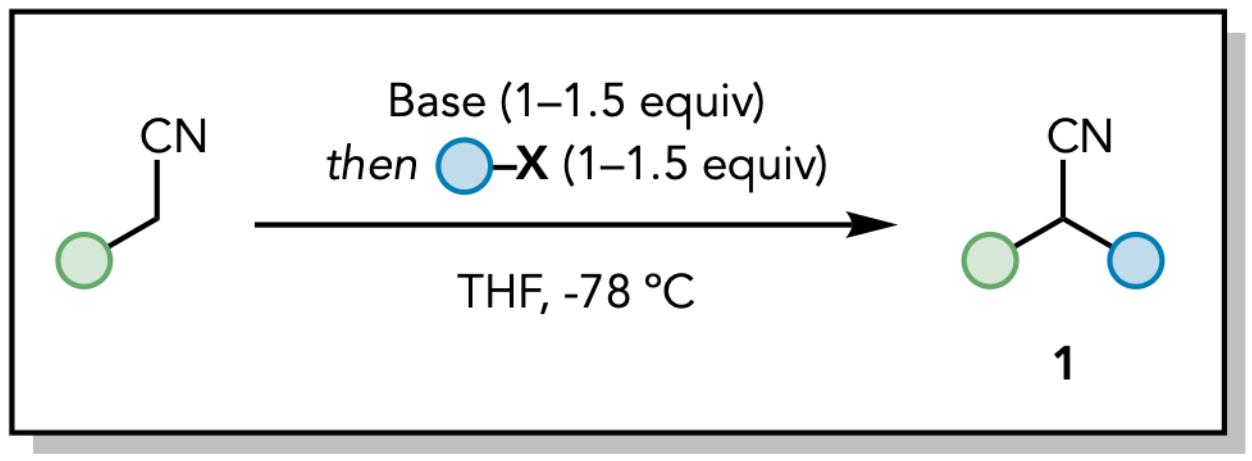

To a flame-dried 100 mL round-bottom flask equipped with a magnetic stir bar was charged KO^{t}Bu or LiHMDS (1.0–1.5 equiv) and aryl acetonitrile (1.0 equiv). The flask was evacuated and backfilled with N_2 three times, then cooled to -78 ºC in an acetone-dry ice bath. THF (0.2 M) was added dropwise under constant stirring. The reaction was stirred for 30 min, then **R-X** (1.0–1.5 equiv) was added as a single portion. The reaction was stirred for an additional 2 h, warming to room temperature. Upon completion, monitoring by TLC, the reaction was quenched by the addition of saturated aqueous $NaHCO_3$ solution. The reaction was transferred to a separatory funnel, and the aqueous phase extracted with EtOAc (3 × 25 mL). The combined organic layers were washed with brine (1 × 25 mL), dried over Na_2SO_4, filtered, and concentrated under reduced pressure to afford crude **1**. The crude material was further purified using flash column chromatography on silica.

3.2 General Procedure B

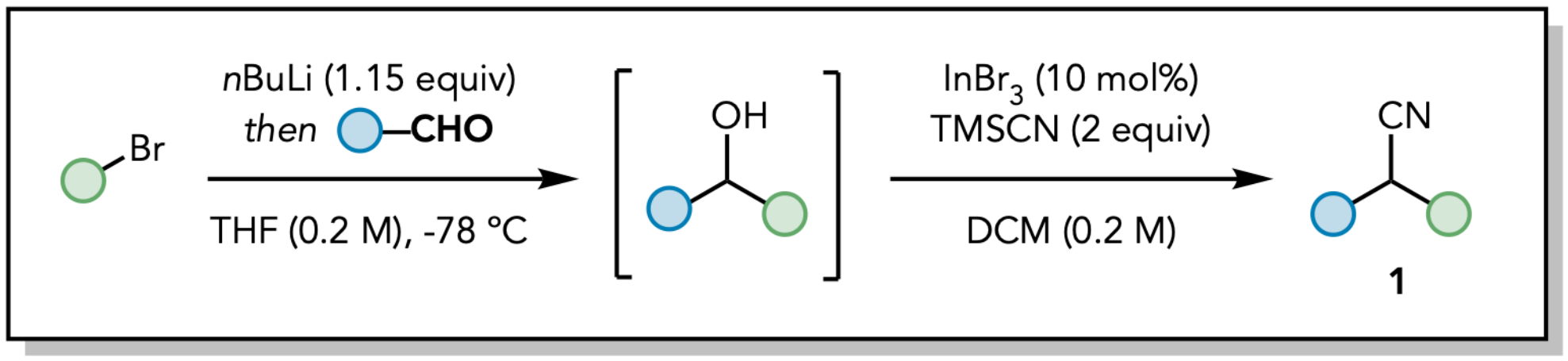

Lithiation–Addition

To a flame-dried 100 mL round-bottom flask equipped with a magnetic stir bar was charged aryl or alkyl bromide (1.1 equiv) and THF (0.2 M) under an N_2 atmosphere. The solution was cooled to -78 ºC in an acetone-dry ice bath, then charged with *n*-BuLi (1.2 equiv). The reaction was stirred for 30 min, after which aldehyde (1.0 equiv) was charged as a solid in a single portion. The reaction was stirred for an additional 2 hours, warming to room temperature. Upon full consumption of aldehyde, monitored by TLC (1:4 EtOAc/hexanes), the reaction was quenched by the addition of saturated aqueous NH_4Cl solution (20 mL), then diluted with H_2O and EtOAc and transferred to a separatory funnel. The aqueous phase was extracted with EtOAc (3 x 20 mL). The combined organic layers were washed with brine (1 x 50 mL). The organic phase was then dried over Na_2SO_4, filtered, and concentrated under reduced pressure to afford the crude alcohol, which was carried forward in the cyanation procedure without further purification.

Cyanation

Cyanation reactions were conducted according to a modified literature procedure.[7] To a flame-dried 40 mL vial equipped with a magnetic stir bar was charged $InBr_3$ (10 mol%) and CH_2Cl_2 (0.40 M) under an N_2 atmosphere. The vial was then charged with TMSCN (2.0 equiv) in a single portion. A 20 mL scintillation vial containing the crude alcohol was charged with CH_2Cl_2 (0.40 M) under an inert atmosphere. The solution was then transferred dropwise via syringe to the solution containing $InBr_3$ and TMSCN. The reaction was stirred for 0.5–1 h, with conversion monitored by TLC (1:4 EtOAc/hexanes). Upon completion, the reaction was quenched by the addition of saturated aqueous $NaHCO_3$ solution. The mixture was then transferred to a separatory funnel. The aqueous layer was extracted with DCM (3 × 15 mL). The combined organic phase was then washed with saturated aqueous $NaHCO_3$ solution (1 × 15 mL) and brine (1 × 15 mL), dried over Na_2SO_4, filtered, and concentrated under reduced pressure to afford crude **1**. The material was further purified using flash column chromatography on silica.

3.3 General Procedure C

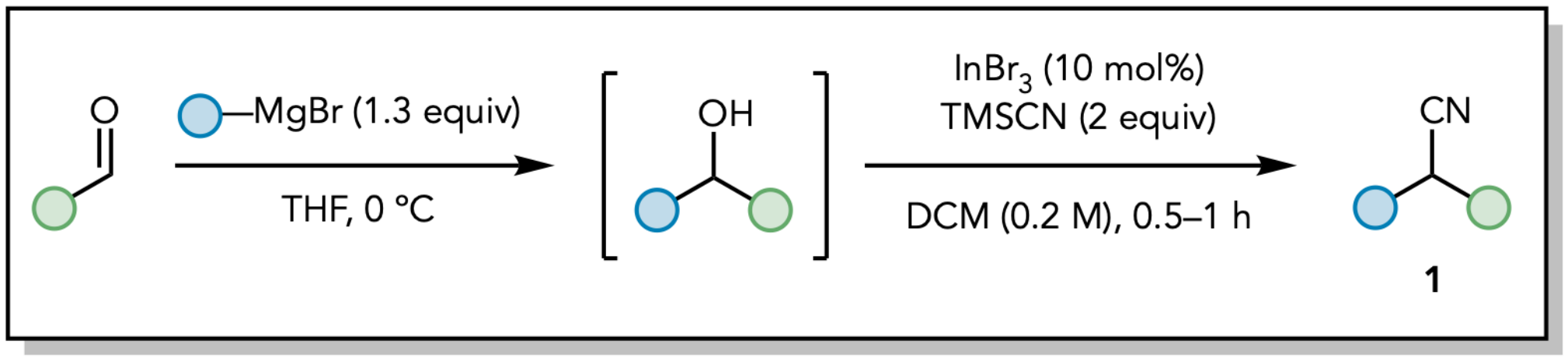

Grignard Addition

To a flame-dried 100 mL round-bottom flask equipped with a magnetic stir bar was charged aldehyde (1.0 equiv) and THF (1.0 M) under an N_2 atmosphere. The solution was cooled to 0 ºC, after which point a solution of Grignard reagents (1.3 equiv, solution in THF or hexanes) was charged to the reaction vessel. The stirring mixture was allowed to equilibrate to room temperature over the course of 2 h. Upon full consumption of aldehyde as monitored by TLC (1:4 EtOAc/hexanes), the reaction was quenched by the addition of saturated aqueous NH_4Cl solution (20 mL), then diluted with H_2O and EtOAc and transferred to a separatory funnel. The aqueous phase was extracted with EtOAc (3 × 20 mL). The combined organic layers were combined and washed with brine (1 × 50 mL). The organic phase was then dried over Na_2SO_4, filtered, and concentrated to afford crude alcohol, which was carried forward in the cyanation procedure without further purification.

Cyanation

Cyanation reactions were conducted according to a modified literature procedure.[7] To a flame-dried 40 mL vial equipped with a magnetic stir bar was charged $InBr_3$ (10 mol%) and CH_2Cl_2 (0.40 M) under an N_2 atmosphere. The vial was then charged with TMSCN (2.0 equiv) in a single portion. A 20 mL scintillation vial containing the crude alcohol was charged with CH_2Cl_2 (0.40 M) under an inert atmosphere. The solution was then transferred dropwise via syringe to the solution containing $InBr_3$ and TMSCN. The reaction was stirred for 0.5–1 h, with conversion monitored by TLC (1:4 EtOAc/hexanes). Upon completion, the reaction was quenched by the addition of saturated aqueous $NaHCO_3$ solution. The mixture was then transferred to a separatory funnel. The aqueous layer was extracted with DCM (3 × 15 mL). The combined organic phase was then washed with saturated aqueous $NaHCO_3$ solution (1 × 15 mL) and brine (1 × 15 mL), dried over Na_2SO_4, filtered, and concentrated under reduced pressure to afford crude **1**. The material was further purified using flash column chromatography on silica.

3.4 General Procedure D

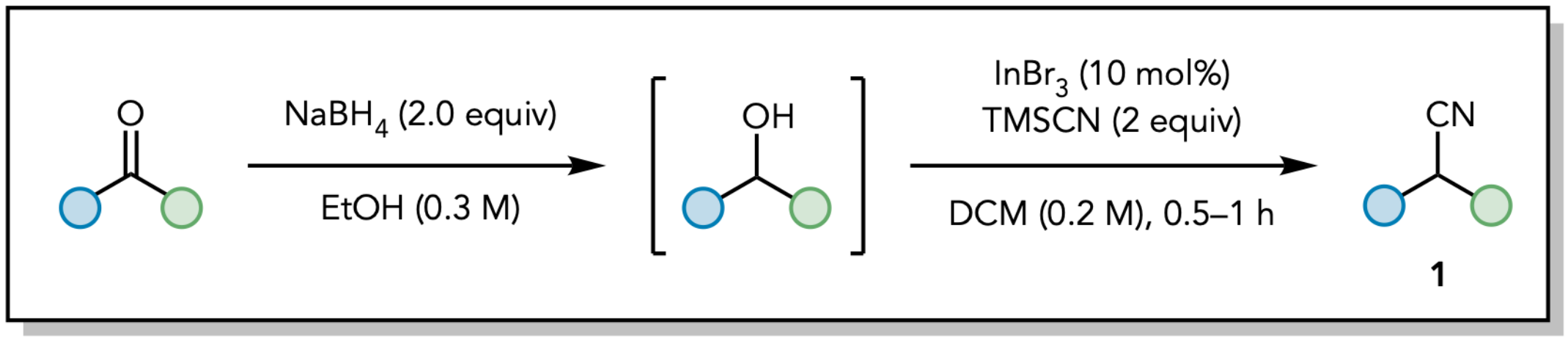

Reduction

To a flame-dried 100 mL round-bottom flask equipped with a magnetic stir bar was charged ketone (1.0 equiv) and EtOH (0.3 M) under an N_2 atmosphere. The solution was cooled to 0 ºC and then charged with sodium borohydride (2.0 equiv). The stirring mixture was allowed to equilibrate to room temperature over the course of 1 h. Upon full consumption of ketone, monitored by TLC (1:4 EtOAc/hexanes), the reaction was quenched by the addition of 10% aqueous NaOH solution (20 mL), then diluted with H_2O and EtOAc and transferred to a separatory funnel. The aqueous phase was extracted with EtOAc (3 × 20 mL). The combined organic layers were combined and washed with brine (1 × 50 mL). The organic phase was then dried over Na_2SO_4, filtered, and concentrated to afford crude alcohol, which was carried forward in the cyanation procedure without further purification.

Cyanation

Cyanation reactions were conducted according to a modified literature procedure.[7] To a flame-dried 40 mL vial equipped with a magnetic stir bar was charged $InBr_3$ (10 mol%) and CH_2Cl_2 (0.40 M) under an N_2 atmosphere. The vial was then charged with TMSCN (2.0 equiv) in a single portion. A 20 mL scintillation vial containing the crude alcohol was charged with CH_2Cl_2 (0.40 M) under an inert atmosphere. The solution was then transferred dropwise via syringe to the solution containing $InBr_3$ and TMSCN. The reaction was stirred for 0.5–1 h, with conversion monitored by TLC (1:4 EtOAc/hexanes). Upon completion, the reaction was quenched by the addition of saturated aqueous $NaHCO_3$ solution. The mixture was then transferred to a separatory funnel. The aqueous layer was extracted with DCM (3 × 15 mL). The combined organic phase was then washed with saturated aqueous $NaHCO_3$ solution (1 × 15 mL) and brine (1 × 15 mL), dried over Na_2SO_4, filtered, and concentrated under reduced pressure to afford crude **1**. The material was further purified using flash column chromatography on silica.

rac-**1a** 2-(3,4-dimethoxyphenyl)-3-methylbutanenitrile

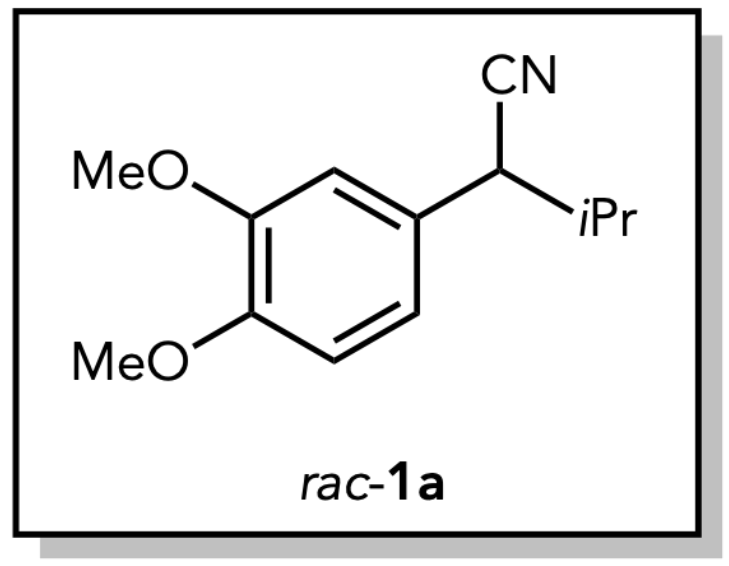

The tile compound was synthesized according to **General Procedure A** from 2-(3,4-dimethoxyphenyl)acetonitrile (5.0 g, 28 mmol, 1.0 equiv), KO^{t}Bu (3.2 g, 28 mmol, 1.0 equiv), and 2-iodopropane (2.8 mL, 28 mmol, 1.0 equiv) in THF (140 mL). The crude material was purified by silica gel column chromatography (0–30% EtOAc/hexanes) to afford the title compound as a white solid in 56% yield (3.4 g, 15 mmol). **^{1}H NMR** (500 MHz, $CDCl_3$) δ 6.83 (d, *J* = 1.5 Hz, 2H), 6.78 (d, *J* = 1.6 Hz, 1H), 3.88 (s, 3H), 3.58 (s, 3H), 2.14 – 2.04 (m, *J* = 6.7 Hz, 1H), 1.03 (d, *J* = 6.9 Hz, 6H). **^{13}C NMR** (126 MHz, $CDCl_3$) δ 149.28, 148.86, 127.49, 120.38, 120.18, 111.33, 110.94, 56.11, 56.06, 44.87, 33.94, 20.85, 19.06. **HRMS**: m/z $[M+Na]^+$ calcd. for $C_{13}H_{17}NO_2Na$: 242.11514, found: 242.11406 (-4.49 ppm). **IR**: 2963, 1979, 1700, 1684, 1652, 1593, 1558, 1516, 1464, 1419, 1258, 1143, 1025, 805, 763.

rac-**1b** 2-(3,4-dimethoxyphenyl)propanenitrile

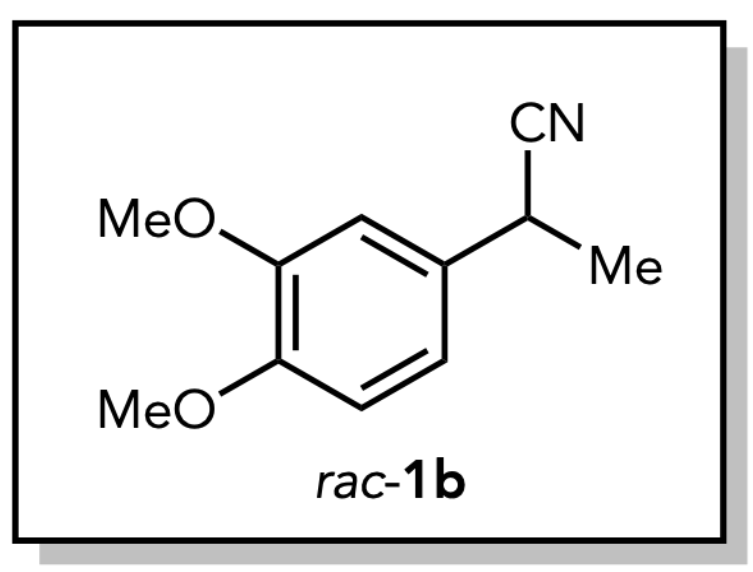

The title compound was synthesized according to General Procedure A from 2-(3,4-dimethoxyphenyl)acetonitrile (1.1 g, 6.0 mmol, 1.0 equiv), KO^{t}Bu (670 mg, 6.0 mmol, 1.0 equiv), and iodomethane (560 µL, 9.0 mmol, 1.5 equiv). The crude material was purified by silica gel column chromatography (0–30% EtOAc/hexanes) to afford the title compound as a white solid in 27% yield (310 mg, 1.6 mmol). **^{1}H NMR** (500 MHz, $CDCl_3$) δ 6.91 – 6.82 (m, 3H), 3.91 (s, 3H), 3.88 (s, 3H), 3.85 (q, *J* = 7.3 Hz, 1H), 1.64 (d, *J* = 7.3 Hz, 3H). **^{13}C NMR** (126 MHz, $CDCl_3$) δ 149.56, 148.96, 129.64, 121.94, 119.07, 111.61, 109.95, 56.14, 56.14, 31.01, 21.68. **HRMS**: m/z M^+ calcd. for $C_{11}H_{13}NO_2$: 191.09408, found: 191.09350 (-3.03 ppm). **IR**: 2937, 2836, 2238, 1593, 1515, 1451, 1418, 1339, 1251, 1232, 1160, 1142, 1023, 919, 852, 807, 763, 604.

rac-**1c** 2-(3,4-dimethoxyphenyl)butanenitrile

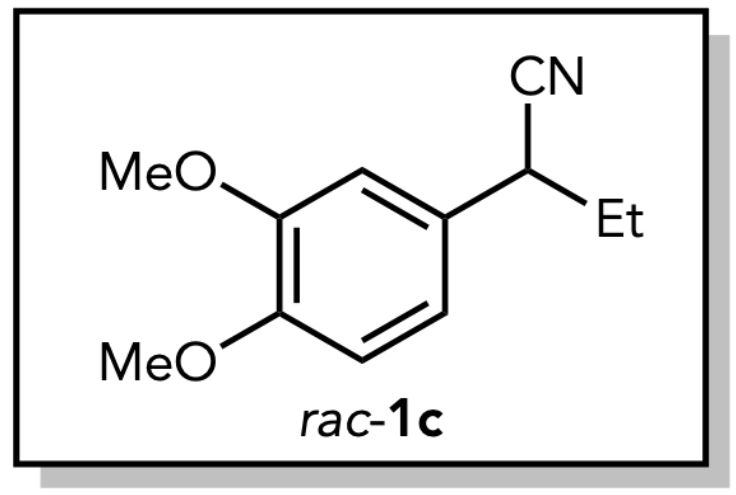

The title compound was synthesized according to **General Procedure A** from 2-(3,4-dimethoxyphenyl)acetonitrile (1.1 g, 6.0 mmol, 1.0 equiv), KO^{t}Bu (670 mg, 6.0 mmol, 1.0 equiv), and ethyl iodide (720 µL, 9.0 mmol, 1.5 equiv). The crude material was purified by silica gel column chromatography (0–30% EtOAc/hexanes) to afford the title compound as a translucent oil in 48% yield (600 mg, 2.9 mmol). **^{1}H NMR** (500 MHz, $CDCl_3$) δ 6.88 – 6.82 (m, 2H), 6.81 (s, 1H), 3.89 (s, 3H), 3.87 (s, 3H), 3.67 (t, *J* = 7.2 Hz, 1H), 1.98 – 1.87 (m, 2H), 1.06 (t, *J* = 7.3 Hz, 3H). **^{13}C NMR** (126 MHz, $CDCl_3$) δ 149.45, 148.90, 128.28, 121.10, 119.75, 111.49, 110.39, 56.11, 56.09, 38.65, 29.38, 11.63. **HRMS**: m/z $[M+H]^+$ calcd. for $C_{12}H_{16}NO_2$: 206.11755, found: 206.11777 (+1.07 ppm). **IR**: 2934, 2835, 2239, 1592, 1515, 1456, 1419, 1238, 1142, 1023, 848, 807, 763, 638.

rac-**1d** 2-(3,4-dimethoxyphenyl)-3,3-dimethylbutanenitrile

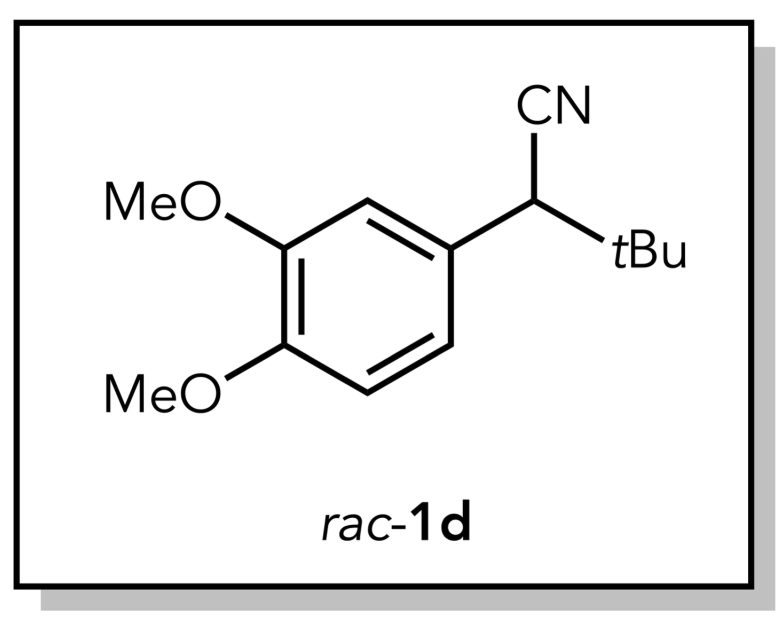

The title compound was synthesized according to **General Procedure C** from 3,4-dimethoxybenzaldehyde (1.5 g, 9.0 mmol, 1.0 equiv) and *tert*-butyl(chloro)magnesium (12 mL, 12 mmol, 1.3 equiv, 1.0 M in THF). The crude material was purified by silica gel chromatography (0–15% EtOAc/hexanes) to afford the title compound as a colorless oil in 37% yield (770 mg, 3.3 mmol) . **^{1}H NMR** (500 MHz, $CDCl_3$) δ 6.86 – 6.80 (m, 2H), 6.78 (d, *J* = 2.1 Hz, 1H), 3.89 (s, 3H), 3.89 (s, 3H), 1.06 (s, 9H). **^{13}C NMR** (126 MHz, CDCl3) δ 148.99, 148.73, 125.91, 122.03, 120.71, 112.58, 110.89, 56.16, 56.05, 49.43, 35.30, 27.58. **HRMS**: m/z $[M+Na]^+$ $C_{14}H_{19}NO_2Na$ calcd. 256.1308; found: 256.12963 (-4.51 ppm). **IR**: 2952, 2931, 2852, 2235, 1652, 1592, 1514, 1456, 1419, 1382, 1238, 1143, 1025, 854, 802, 762, 618.

rac-**1e** 2-cyclopropyl-2-(3,4-dimethoxyphenyl)acetonitrile

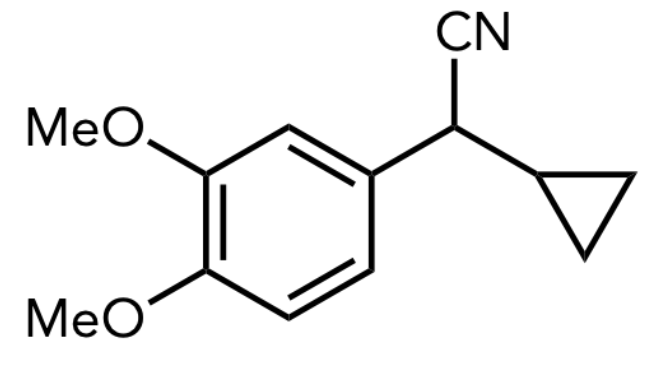

rac-**1e**

The title compound was synthesized according to **General Procedure B** from bromocyclopropane (530 µL, 6.6 mmol, 1.10 equiv) and 3,4-dimethoxybenzaldehyde (1.0 g, 6.0 mmol, 1.0 equiv). The crude material was purified via silica gel chromatography (0–30% EtOAc/hexanes) to afford the title compound as an off-white solid in 51% yield (670 mg, 3.1 mmol) . **^{1}H NMR** (500 MHz, $CDCl_3$) δ 6.96 – 6.83 (m, 3H), 3.91 (s, 3H), 3.89 (s, 3H), 3.46 (d, *J* = 7.5 Hz, 1H), 1.35 – 1.24 (m, 1H), 0.78 – 0.64 (m, 2H), 0.59 – 0.43 (m, 2H). **^{13}C NMR** (126 MHz, $CDCl_3$) δ 149.44, 149.09, 128.09, 120.17, 119.79, 111.49, 110.57, 56.15, 56.12, 40.75, 15.69, 4.87, 4.02. **HRMS**: m/z M^+ calcd. for $C_{13}H_{15}NO_2$: 217.10973, found: 217.10913 (-2.76 ppm). **IR**: 2934, 2835, 2237, 1652, 1592, 1514, 1463, 1419, 1336, 1261, 1143, 1023, 807, 763, 635.

rac-**1f** 2-cyclobutyl-2-(3,4-dimethoxyphenyl)acetonitrile

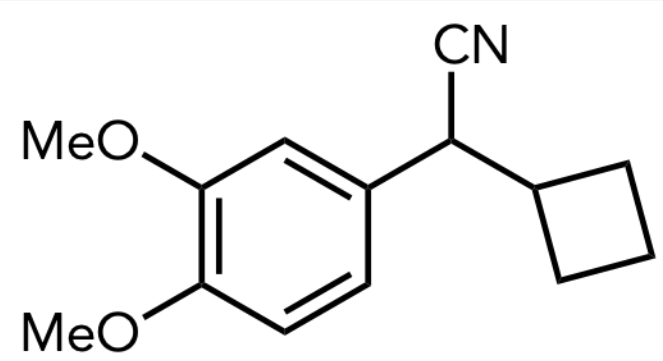

rac-**1f**

The title compound was synthesized according to **General Procedure A** from 2-(3,4-dimethoxyphenyl)acetonitrile (1.1 g, 6.0 mmol, 1.0 equiv), LiHMDS (1.1 g, 6.6 mmol, 1.1 equiv), and bromocyclobutane (850 µL, 9.9 mmol, 1.5 equiv). The crude material was purified by silica gel column chromatography (0–30% EtOAc/hexanes) to afford the title compound as a white solid in 57% yield (800 mg, 3.5 mmol). **^{1}H NMR** (500 MHz, $CDCl_3$) δ 6.85 – 6.79 (m, 2H), 6.77 (d, *J* = 1.9 Hz, 1H), 3.89 (s, 3H), 3.87 (s, 3H), 3.70 (d, *J* = 7.8 Hz, 1H), 2.78 – 2.68 (m, 1H), 2.17 – 2.08 (m, 1H), 2.07 – 1.96 (m, 2H), 1.95 – 1.83 (m, 3H). **^{13}C NMR** (126 MHz, $CDCl_3$) δ 149.41, 148.93, 127.24, 120.21, 119.78, 111.45, 110.44, 56.12, 56.08, 42.86, 39.90, 26.71, 26.19, 17.55. **HRMS**: m/z $[M+H]^+$ calcd. for $C_{14}H_{17}NO_2$: 232.13320, found: 232.13227 (-4.01 ppm). **IR**: 2935, 2238, 1653, 1592, 1513, 1463, 1419, 1335, 1238, 1142, 1024, 851, 811, 761, 666.

rac-**1g** 2-cyclopentyl-2-(3,4-dimethoxyphenyl)acetonitrile

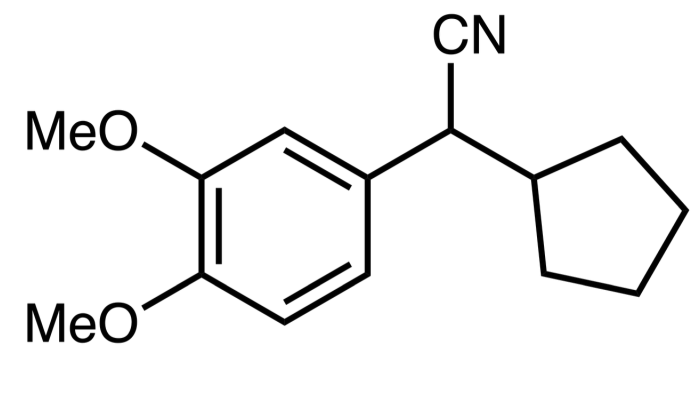

rac-**1g**

The title compound was synthesized according to **General Procedure A** from (3,4-dimethoxyphenyl)acetonitrile (1.0 g, 5.6 mmol, 1.0 equiv), NaO^{t}Bu (530 mg, 5.5 mmol, 1.0 equiv), and bromocyclopentane (0.59 mL, 5.5 mmol, 1.0 equiv) in THF (30 mL). The crude material was purified by silica gel column chromatography (0–20% EtOAc/hexanes) to afford the title compound in 66% yield (920 mg, 3.7 mmol) as a white solid. **^{1}H NMR** (500 MHz, $CDCl_3$) δ 6.87 – 6.84 (m, 2H), 6.83 – 6.81 (m, 1H), 3.90 (s, 3H), 3.88 (s, 3H), 3.64 (d, *J* = 7.8 Hz, 1H), 2.35 – 2.24 (m, 1H), 1.92 – 1.82 (m, 1H), 1.77 – 1.63 (m, 3H), 1.63 – 1.44 (m, 3H), 1.38 – 1.27 (m, 1H). **^{13}C NMR** (126 MHz, $CDCl_3$) δ 149.37, 148.86, 128.46, 120.90, 120.09, 111.38, 110.68, 56.14, 56.09, 45.45, 42.28, 31.10, 30.48, 25.06, 25.02. **HRMS:** m/z M^+ $C_{15}H_{19}NO_2$ calcd. For 245.14158; found: 245.14086 (-2.93 ppm). **IR**: 3003, 2938, 2864, 2837, 2228, 2014, 1605, 1592, 1516, 1464, 1448, 1419, 1338, 1294, 1233, 1185, 1147, 1137, 1022, 850, 775, 745, 648, 636, 598.

rac-**1h** 2-(3,4-diisopropoxyphenyl)-3-methylbutanenitrile

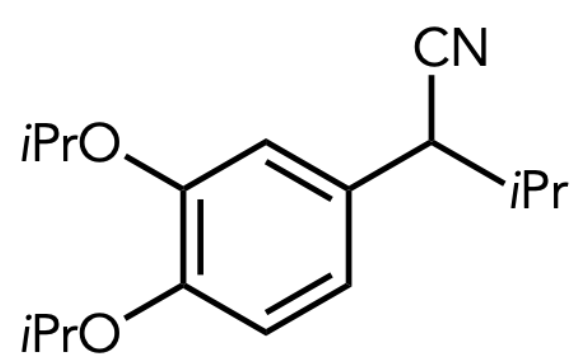

rac-**1h**

The title compound was synthesized according to **General Procedure C** from 3,4-diisopropoxybenzaldehyde (670 mg, 3.0 mmol, 1.0 equiv) and isopropylmagnesium chloride (2.0 M solution in THF, 3.0 mL, 6.0 mmol, 2.0 equiv). The crude material was purified by silica gel column chromotagraphy (0–20% EtOAc/hexanes) to afford the title compound in 61% yield as a yellow oil (509 mg, 1.8 mmol). **^{1}H NMR** (500 MHz, $CDCl_3$) δ 6.89 – 6.80 (m, 3H), 4.51 – 4.43 (m, *J* = 6.1 Hz, 2H), 3.55 (d, *J* = 6.4 Hz, 1H), 2.12 – 2.03 (m, *J* = 6.7 Hz, 1H), 1.33 (d, *J* = 6.1 Hz, 12H), 1.03 (d, *J* = 6.7 Hz, 6H). **^{13}C NMR** (126 MHz, $CDCl_3$) δ 149.18, 149.11, 128.19, 121.39, 120.27, 118.22, 117.96, 72.61, 72.28, 44.73, 33.95, 22.35, 22.34, 20.86, 19.09. **HRMS**: m/z M^+ calcd. for $C_{17}H_{25}NO_2$: 275.18798, found: 275.18656 (-5.16 ppm). **IR**: 2974, 2930, 2150, 1700, 1684, 1653, 1576, 1503, 1465, 1425, 1371, 1260, 1176, 1135, 1105, 993, 930, 755.

rac-**1i** 2-(3,4-bis(benzyloxy)phenyl)-3-methylbutanenitrile

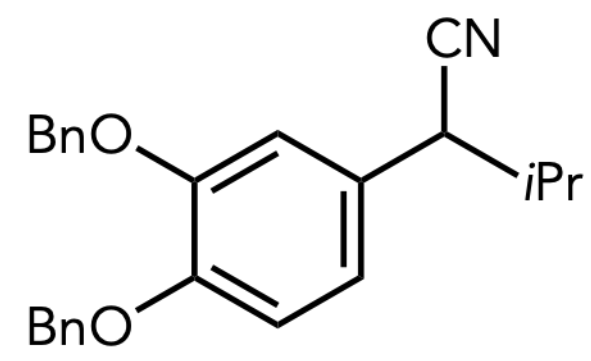

rac-**1i**

The title compound was synthesized according to **General Procedure C** from 3,4-dibenzyloxybenzaldehyde (1.9 g, 6.0 mmol, 1.0 equiv) and isopropylmagnesium chloride (2.0 M solution in THF, 6.0 mL, 12.0 mmol, 2.0 equiv). The crude material was purified via silica gel chromatography (0–30% EtOAc/hexanes) to afford the title compound as a white solid in 32% yield (720 mg, 1.9 mmol) . **^{1}H NMR** (500 MHz, $CDCl_3$) δ 7.46 – 7.40 (m, 4H), 7.39 – 7.27 (m, 6H), 6.91 (d, *J* = 8.3 Hz, 1H), 6.85 (d, *J* = 2.2 Hz, 1H), 6.79 (dd, *J* = 8.2, 2.2 Hz, 1H), 5.18 – 5.15 (m, 4H), 3.52 (d, *J* = 6.3 Hz, 1H), 2.05 – 1.97 (m, 1H), 0.97 (d, *J* = 6.8 Hz, 3H), 0.96 (d, *J* = 6.8 Hz, 3H). **^{13}C NMR** (126 MHz, $CDCl_3$) δ 148.96, 148.93, 137.19, 137.05, 128.68, 128.66, 128.10, 128.07, 128.04, 127.61, 127.43, 121.19, 120.13, 115.23, 115.05, 71.63, 71.43, 44.74, 33.90, 20.79, 18.99. **HRMS**: m/z M^+ calcd. for $C_{25}H_{25}NO_2$: 371.18798, found: 371.18649 (-4.01 ppm). **IR**: 3019, 2961, 2690, 2173, 1653, 1591, 1507, 1454, 1426, 1371, 1257, 1215, 1139, 1012, 854, 804, 748, 696.

rac-**1j** 2-(4-methoxyphenyl)-2-(2,4,5-trimethoxyphenyl)acetonitrile

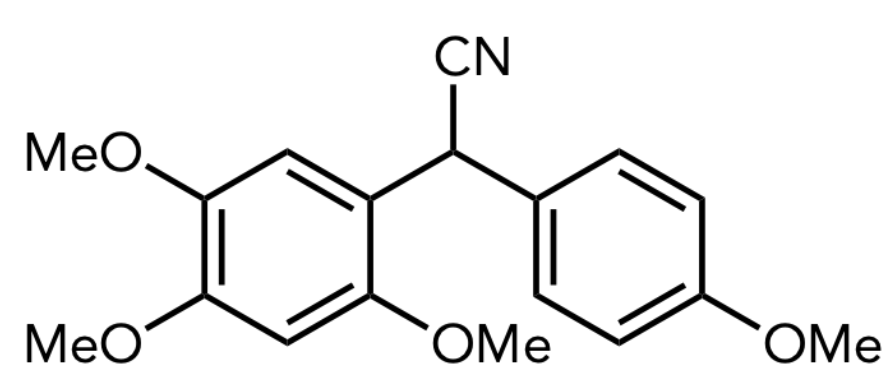

rac-**1j**

The title compound was synthesized according to **General Procedure C** from 2,4,5-trimethoxybenzaldehyde (1.0 g, 5.1 mmol, 1.0 equiv) and (4-methoxyphenyl)magnesium bromide (0.5 M solution in THF, 11.2 mL, 5.6 mmol, 1.1 equiv). The crude material was purified via silica gel chromatography (10–30% EtOAc/hexanes) to afford the title compound as an orange oil in 60 % yield (980 mg, 3.1 mmol) . **^{1}H NMR** (500 MHz, $CDCl_3$) δ 7.27 (d, *J* = 8.8 Hz, 2H), 6.86 (d, *J* = 8.8 Hz, 2H), 6.82 (s, 1H), 6.53 (s, 1H), 5.47 (s, 1H), 3.89 (s, 3H), 3.82 (s, 3H), 3.80 (s, 3H), 3.79 (s, 3H). **^{13}C NMR** (126 MHz, $CDCl_3$) δ 159.29, 150.68, 149.98, 143.57, 128.78, 128.09, 120.54, 116.07, 114.40, 112.56, 97.68, 56.85, 56.62, 56.34, 55.46, 35.16. **HRMS**: m/z $[M+H]^+$ calcd. for $C_{18}H_{20}NO_4$: 314.13868, found: 314.13755 (-3.60 ppm). **IR**: 2935, 2833, 2235, 1653, 1610, 1507, 1457, 1397, 1304, 1248, 1204, 1176, 1107, 1028, 844, 753.

rac-**1k** 2-(*p*-tolyl)-2-(2,4,5-trimethoxyphenyl)acetonitrile

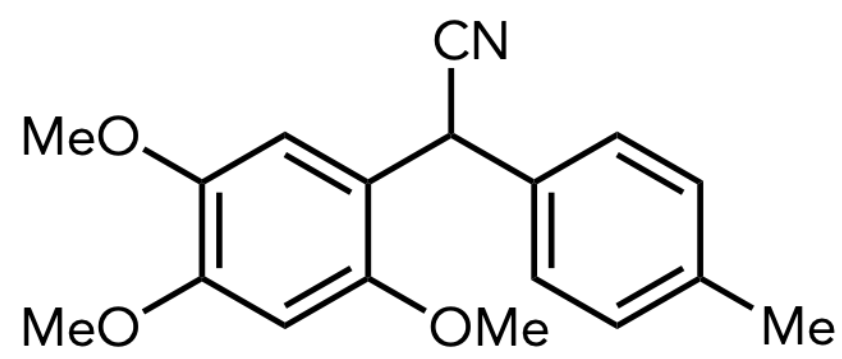

rac-**1k**

The title compound was synthesized according to **General Procedure B** from 2,4,5-trimethoxybenzaldehyde (1.2 g, 6.0 mmol, 1.0 equiv) and 1-bromo-4-methyl-benzene (1.1 g, 6.6 mmol, 1.1 equiv). The crude material was purified via silica gel column chromatography (0–30% EtOAc/hexanes) to afford the title compound as a yellow oil in 73% yield (1.3 g, 4.4 mmol). **^{1}H NMR** (500 MHz, $CDCl_3$) δ 7.24 (d, *J* = 8.0 Hz, 2H), 7.15 (d, *J* = 7.9 Hz, 2H), 6.82 (s, 1H), 6.53 (s, 1H), 5.50 (s, 1H), 3.89 (s, 3H), 3.83 (s, 3H), 3.80 (s, 3H), 2.33 (s, 3H). **^{13}C NMR** (126 MHz, $CDCl_3$) δ 150.67, 149.96, 143.56, 137.72, 133.06, 129.69, 127.47, 120.45, 115.93, 112.60, 97.64, 56.81, 56.59, 56.30, 35.48, 21.18. **HRMS**: m/z M^+ calcd. for $C_{18}H_{19}NO_3$: 297.13594 found: 297.13542 (-1.75 ppm). **IR**: 2931, 2244, 1609, 1509, 1454, 1439, 1398, 1306, 1270, 1238, 1203, 1176, 1111, 1029, 879, 859, 843, 748, 704, 681, 634, 574, 503, 466, 430.

rac-**1l** 2-phenyl-2-(2,4,5-trimethoxyphenyl)acetonitrile

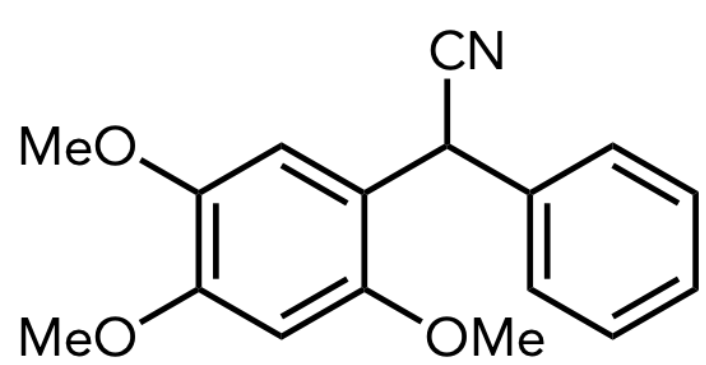

rac-**1l**

The title compound was synthesized according to **General Procedure C** from 2,4,5-trimethoxybenzaldehyde (1.2 g, 6.0 mmol, 1.0 equiv) and bromo(phenyl)magnesium (3.0 M solution in Et_2O, 2.3 mL, 6.9 mmol, 1.2 equiv). The crude material was purified via silica gel column chromatography (0–30% EtOAc/hexanes) to afford the title compound as a light pink solid in 76% yield (1.3 g, 4.6 mmol). **^{1}H NMR** (500 MHz, $CDCl_3$) δ 7.39 – 7.32 (m, 4H), 7.31 – 7.27 (m, 1H), 6.82 (s, 1H), 6.54 (s, 1H), 5.54 (s, 1H), 3.89 (s, 3H), 3.83 (s, 3H), 3.80 (s, 3H). **^{13}C NMR** (126 MHz, $CDCl_3$) δ 150.71, 150.04, 143.58, 136.07, 129.02, 127.95, 127.61, 120.29, 115.68, 112.62, 97.62, 56.81, 56.58, 56.31, 35.85. **HRMS**: m/z M^+ calcd. for $C_{17}H_{17}NO_3$: 283.12029, found: 283.11878 (-5.33 ppm). **IR**: 2932, 2832, 2185, 1653, 1600, 1507, 1452, 1397, 1308, 1204, 1110, 1028, 860, 826, 752, 699.

rac-**1m** 2-([1,1'-biphenyl]-4-yl)-2-(2,4,5-trimethoxyphenyl)acetonitrile

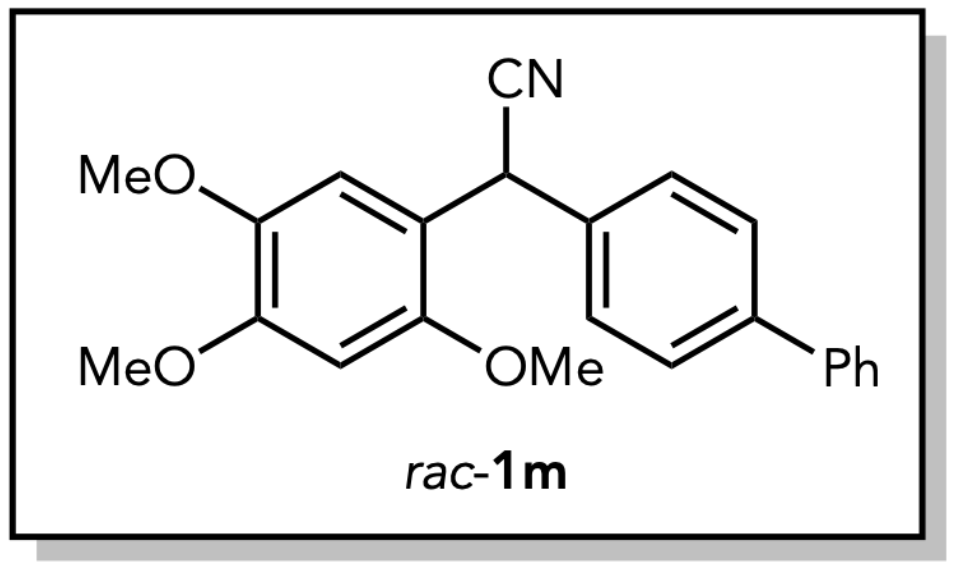

The title compound was synthesized according to **General Procedure B** from 2,4,5-trimethoxybenzaldehyde (1.2 g, 6.0 mmol, 1.0 equiv) and 1-bromo-4-phenyl-benzene (1.5 g, 6.6 mmol, 1.1 equiv). The crude material was purified via silica gel chromatography (0–35% EtOAc/hexanes) to afford the title compound as a foamy white solid in 34% yield (750 mg, 2.1 mmol). **^{1}H NMR** (500 MHz, $CDCl_3$) δ 7.59 – 7.54 (m, 4H), 7.46 – 7.41 (m, 4H), 7.38 – 7.32 (m, 1H), 6.88 (s, 1H), 6.55 (s, 1H), 5.58 (s, 1H), 3.90 (s, 3H), 3.86 (s, 3H), 3.82 (s, 3H). **^{13}C NMR** (126 MHz, $CDCl_3$) δ 150.74, 150.13, 143.66, 140.95, 140.49, 135.10, 128.98, 128.03, 127.75, 127.68, 127.22, 120.29, 115.60, 112.64, 97.67, 56.89, 56.63, 56.34, 35.62. **HRMS**: m/z M^+ calcd. for $C_{23}H_{21}NO_3$: 359.15159 found: 359.15074 (-2.36 ppm). **IR**: 3012, 2933, 2166, 1653, 1600, 1507, 1486, 1464, 1437, 1398, 1308, 1205, 1109, 1031, 1008, 848, 749, 697, 666.

rac-**1n** 2-(4-fluorophenyl)-2-(2,4,5-trimethoxyphenyl)acetonitrile

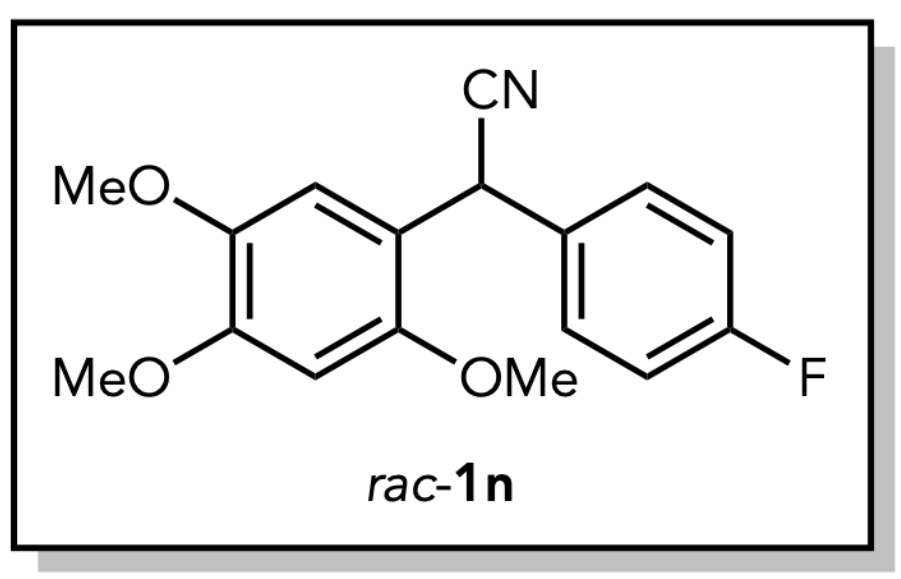

The title compound was synthesized according to **General Procedure B** from 2,4,5-trimethoxybenzaldehyde (780 mg, 4.0 mmol, 1.0 equiv) and 1-bromo-4-fluoro-benzene (0.48 mL, 4.4 mmol, 1.1 equiv). The crude material was purified via silica gel chromatography (0–35% EtOAc/hexanes) to afford the title compound as a colorless oil in 78% yield (950 mg, 3.2 mmol). **^{1}H NMR** (500 MHz, $CDCl_3$) δ 7.37 – 7.28 (m, 2H), 7.05 – 6.98 (m, 2H), 6.82 (s, 1H), 6.53 (s, 1H), 5.49 (s, 1H), 3.89 (s, 3H), 3.82 (s, 3H), 3.81 (s, 3H). **^{13}C NMR** (126 MHz, $CDCl_3$) δ 162.38 (d, J = 247.0 Hz), 150.66, 150.20, 143.63, 131.95 (d, J = 3.2 Hz), 129.31 (d, J = 8.2 Hz), 120.12, 115.96 (d, J = 21.8 Hz), 115.39, 112.48, 97.64, 56.87, 56.55, 56.33, 35.33. **^{19}F NMR** (471 MHz, $CDCl_3$) δ -114.31 (tt, J = 9.0, 4.8 Hz). **HRMS**: m/z M^+ calcd. for $C_{17}H_{16}FNO_3$: 301.11087 found: 301.10985 (-3.39 ppm). **IR**: 2935, 2832, 2160, 1602, 1505, 1456, 1397, 1306, 1204, 1110, 1029, 845, 754, 578.

rac-**1o** 2-(4-bromophenyl)-2-(2,4,5-trimethoxyphenyl)acetonitrile

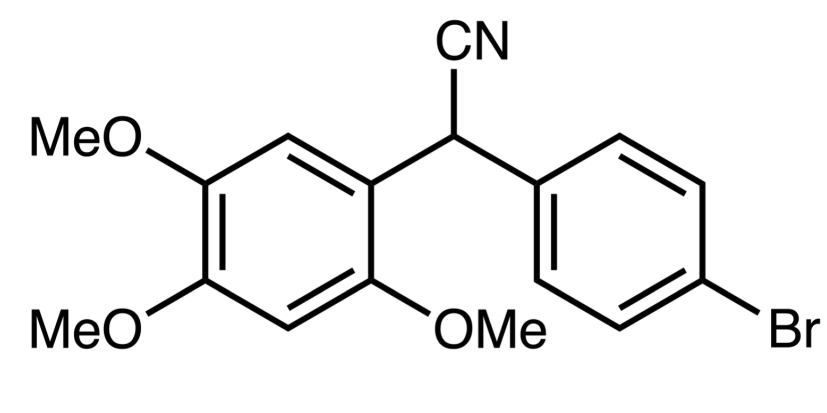

rac-**1o**

The title compound was synthesized according to **General Procedure B** from 2,4,5-trimethoxybenzaldehyde (2.9 g, 15 mmol, 1.0 equiv) and 1,4-dibromobenzene (3.9 g, 2.1 mL, 17 mmol, 1.1 equiv). The crude material was purified via silica gel chromatography (0–30% EtOAc/hexanes) to afford the title compound as a white solid in 22% yield (1.2 g, 3.3 mmol). **^{1}H NMR** (500 MHz, $CDCl_3$) δ 7.50 – 7.43 (m, 2H), 7.25 – 7.21 (m, 2H), 6.82 (s, 1H), 6.53 (s, 1H), 5.47 (s, 1H), 3.90 (s, 3H), 3.82 (s, 3H), 3.81 (s, 3H). **^{13}C NMR** (126 MHz, $CDCl_3$) δ 150.52, 150.15, 143.53, 135.11, 132.02, 129.16, 121.93, 119.65, 114.83, 112.32, 97.47, 56.75, 56.40, 56.20, 35.42. **HRMS:** m/z M^+ calcd. for $C_{17}H_{16}BrNO_3$: 361.03136; found: 361.02993 (-2.44 ppm). **IR**: 2920, 2183, 2029, 1609, 1511, 1487, 1463, 1398, 1307, 1207, 1073, 1030, 1012, 818, 752.

rac-**1p** 2-(4-(trifluoromethyl)phenyl)-2-(2,4,5-trimethoxyphenyl)acetonitrile

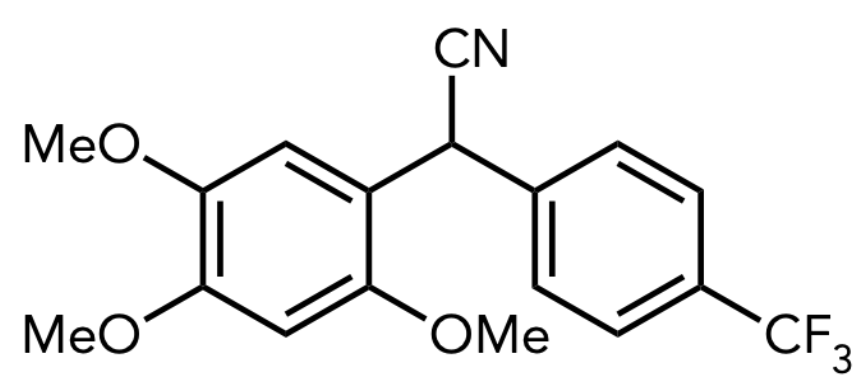

rac-**1p**

The title compound was synthesized according to **General Procedure B** from 2,4,5-trimethoxybenzaldehyde (1.2 g, 6.0 mmol, 1.0 equiv) and 1-bromo-4-(trifluoromethyl)benzene (920 µL, 6.6 mmol, 1.1 equiv). The crude material was purified via silica gel chromatography (10–30% EtOAc/hexanes) to obtain the title compound as a pale yellow oil in 52% yield (1.1 g, 3.2 mmol) . **^{1}H NMR** (500 MHz, $CDCl_3$) δ 7.60 (d, J = 8.1 Hz, 2H), 7.49 (d, J = 8.1 Hz, 2H), 6.85 (s, 1H), 6.54 (s, 1H), 5.57 (s, 1H) 3.90 (s, 3H), 3.83 (s, 3H), 3.82 (s, 3H).**^{13}C NMR** (126 MHz, $CDCl_3$) δ 150.68, 150.44, 143.72, 140.17, 127.99, 129.98-130.72 (q, J = 32.9 Hz), 125.99-126.08 (q, J = 3.8 Hz), 119.56, 114.54, 112.47, 97.58, 56.91, 56.51, 56.34, 35.92.**^{19}F NMR** (471 MHz, $CDCl_3$) δ -62.66. **HRMS**: m/z M^+ calcd. for $C_{18}H_{16}F_3NO_3$: 351.10768 found: 351.10643 (-3.56 ppm). **IR**: 3030, 2184, 1617, 1510, 1465, 1439, 1399, 1324, 1207, 1166, 1143, 1068, 1031, 1019, 851, 784, 667.

rac-**1q** 2-(4-(methylthio)phenyl)-2-(2,4,5-trimethoxyphenyl)acetonitrile

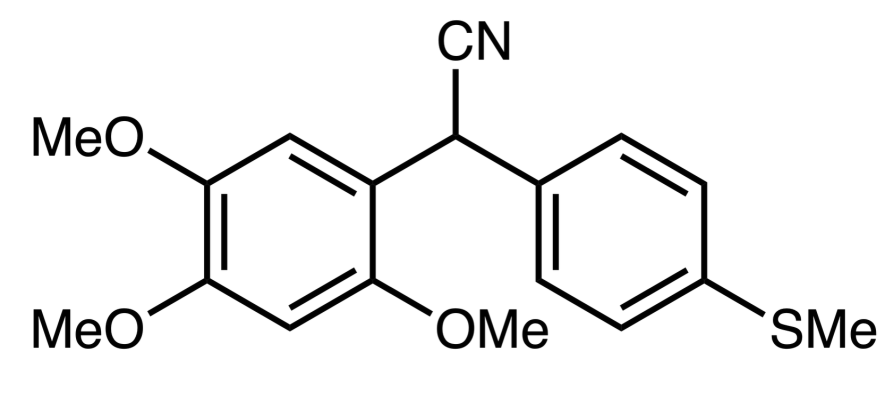

rac-**1q**

The title compound was synthesized according to **General Procedure B** from 2,4,5-trimethoxybenzaldehyde (1.2 g, 6.0 mmol, 1.0 equiv) and 4-bromothioanisole (1.2 g, 820 µL, 6.0 mmol, 1.0 equiv). The crude material was purified via silica gel chromatography (0–30% EtOAc/hexanes) to afford the title compound as a pale orange solid in 51% yield (1.0 g, 3.1 mmol). ^{1}H NMR (500 MHz, $CDCl_3$) δ 7.27 (d, *J* = 7.3 Hz, 1H), 7.24 – 7.18 (m, 2H), 6.82 (s, 1H), 6.53 (s, 1H), 5.48 (s, 1H), 3.89 (s, 3H), 3.82 (s, 3H), 3.81 (s, 3H), 2.47 (s, 3H). **^{13}C NMR** (126 MHz, $CDCl_3$) δ 150.68, 150.10, 143.61, 138.53, 132.79, 128.05, 126.91, 120.17, 115.54, 112.54, 97.63, 56.87, 56.58, 56.33, 35.45, 15.81. **HRMS**: m/z $[M+Na]^+$ $C_{18}H_{19}NO_3SNa$ calcd. 352.09779; found: 352.0965 (-3.66 ppm). **IR**: 2919, 2847, 2009, 1598, 1510, 1457, 1437, 1397, 1307, 1204, 1094, 1030, 818, 750.

rac-**1r** 2-(*m*-tolyl)-2-(2,4,5-trimethoxyphenyl)acetonitrile

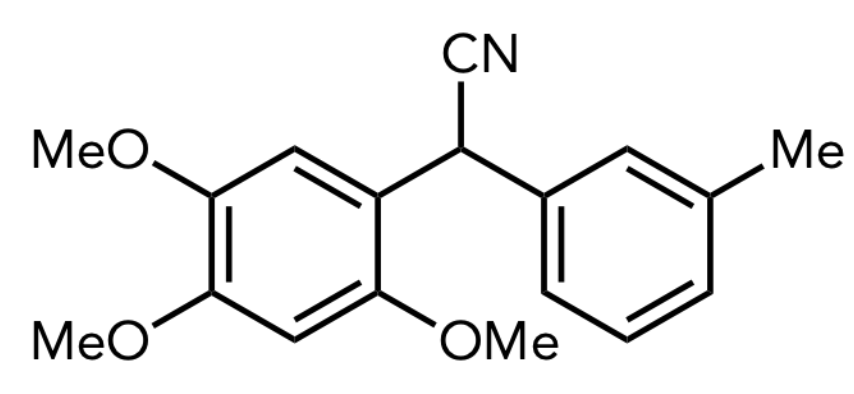

rac-**1r**

The title compound was synthesized according to **General Procedure B** from 2,4,5-trimethoxybenzaldehyde (590 mg, 3.0 mmol, 1.0 equiv) and 3-bromotoluene (400 µL, 3.3 mmol, 1.1 equiv). The crude material was purified via silica gel chromatography (0–30% EtOAc/hexanes) to afford the title compound as a white solid in 63% yield (570 mg, 1.9 mmol). **^{1}H NMR** (500 MHz, $CDCl_3$) δ 7.25 – 7.08 (m, 4H), 6.81 (s, 1H), 6.54 (s, 1H), 5.50 (s, 1H), 3.89 (s, 3H), 3.84 (s, 3H), 3.80 (s, 3H), 2.34 (s, 3H). **^{13}C NMR** (126 MHz, $CDCl_3$) δ 150.74, 150.01, 143.58, 138.84, 135.94, 128.89, 128.74, 128.26, 124.70, 120.42, 115.84, 112.72, 97.66, 56.86, 56.63, 56.32, 35.71, 21.58. **HRMS**: m/z M^+ calcd. for $C_{18}H_{19}NO_3$: 297.13594 found: 297.13516 (-2.63 ppm). **IR**: 2960, 2240, 1607, 1513, 1463, 1415, 1242, 1206, 1170, 1143, 1065, 1026, 814, 751.

rac-**1s** 2-(thiophen-2-yl)-2-(2,4,5-trimethoxyphenyl)acetonitrile

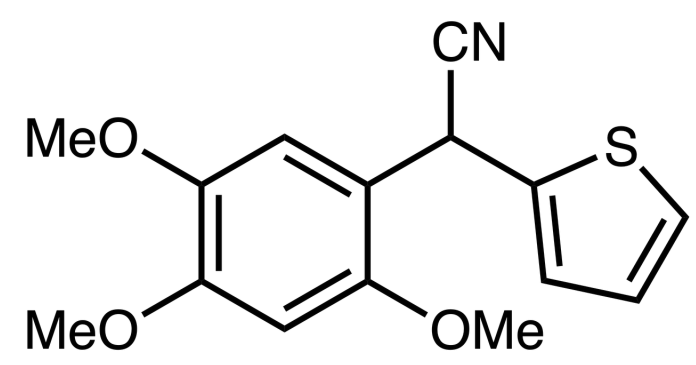

rac-**1s**

The title compound was synthesized according to **General Procedure B** from 2,4,5-trimethoxybenzaldehyde (1.2 g, 6.0 mmol, 1.0 equiv) and 2-bromothiophene (980 mg, 580 µL, 6.0 mmol, 1.0 equiv). The crude material was purified via silica gel chromatography (0–30% EtOAc/hexanes) to afford the title compound as a colorless oil in 25% yield (393 mg, 1.5 mmol). **^{1}H NMR** (500 MHz, $CDCl_3$) δ 7.25 (dd, J = 5.2, 1.3 Hz, 1H), 7.13 (dt, J = 3.7, 1.2 Hz, 1H), 6.98 (dd, J = 5.2, 3.5 Hz, 1H), 6.94 (s, 1H), 6.56 (s, 1H), 5.76 (s, 1H), 3.93 (s, 3H), 3.88 (s, 3H), 3.85 (s, 3H). **^{13}C NMR** (126 MHz, $CDCl_3$) δ 150.47, 150.35, 143.63, 138.93, 127.00, 126.35, 125.99, 119.52, 115.27, 112.17, 97.50, 56.82, 56.58, 56.33, 31.47. **HRMS**: m/z $[M+Na]^+$ calcd. for $C_{15}H_{15}NO_3SNa$: 312.067036 found: 312.06547 (-5.01 ppm). **IR:** 2918, 2832, 2166, 2157, 1979, 1610, 1509, 1456, 1437, 1307, 1205, 1109, 1028, 860, 755, 700.

rac-**1t** 2-(benzo[b]thiophen-2-yl)-2-(2,4,5-trimethoxyphenyl)acetonitrile

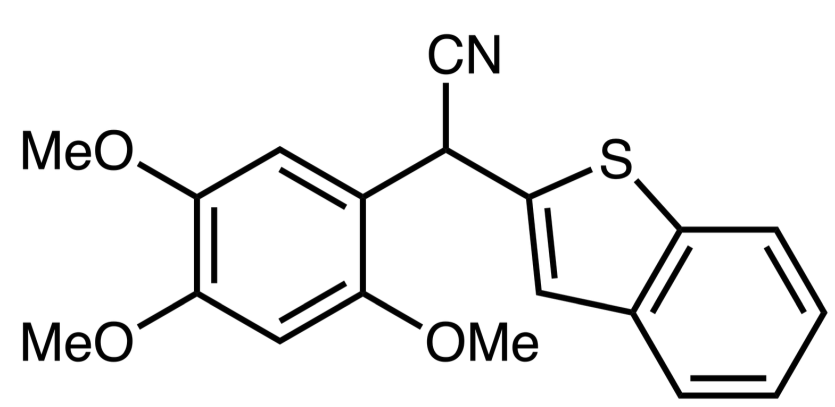

rac-**1t**

The title compound was synthesized according to **General Procedure B** from 2,4,5-trimethoxybenzaldehyde (1.2 g, 6.0 mmol, 1.0 equiv) and 2-bromobenzothiophene (1.3 g, 6.0 mmol, 1.0 equiv). The crude material was purified via silica gel chromatography (0–30% EtOAc/hexanes) to afford the title compound as a gum-like white solid in 56% yield (1.1 g, 3.4 mmol). **^{1}H NMR** (500 MHz, $CDCl_3$) δ 7.76 – 7.70 (m, 2H), 7.38 – 7.28 (m, 2H), 6.96 (s, 1H), 6.56 (s, 1H), 5.81 (s, 1H), 3.91 (s, 3H), 3.88 (s, 3H), 3.83 (s, 3H). **^{13}C NMR** (126 MHz, $CDCl_3$) δ 150.55, 143.71, 140.14, 139.74, 139.20, 124.82, 124.76, 123.86, 122.93, 122.37, 119.06, 114.48, 112.22, 97.45, 56.84, 56.61, 56.33, 32.21. **HRMS:** m/z $[M+Na]^+$ calcd. for $C_{19}H_{17}NO_3SNa$: 362.0822133; found: 362.08082 (-3.84 ppm). **IR:** 2930, 2200, 1609, 1509, 1457, 1436, 1398, 1305, 1204, 1028, 855, 753, 726.

rac-**1u** 2-phenyl-2-(2,3,4-trimethoxyphenyl)acetonitrile

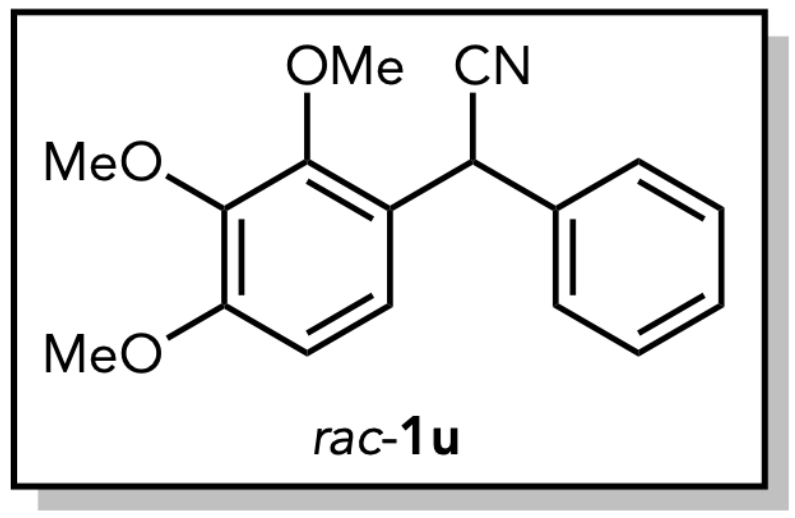

The title compound was synthesized according to **General Procedure C** from 2,3,4-trimethoxybenzaldehyde (1.2 g, 6.0 mmol, 1.0 equiv) and bromo(phenyl)magnesium (1.0 M solution in THF, 8.0 mL, 8.0 mmol, 1.3 equiv). The crude material was purified via silica gel chromatography (0–30% EtOAc/hexanes) to afford the title compound as a translucent oil in 321% yield (540 mg, 1.9 mmol). **^{1}H NMR** (500 MHz, $CDCl_3$) δ 7.37 – 7.34 (m, 4H), 7.32 – 7.27 (m, 1H), 7.01 (d, *J* = 8.6 Hz, 1H), 6.66 (d, *J* = 8.7 Hz, 1H), 5.42 (s, 1H), 3.85 (s, 3H), 3.85 (s, 3H), 3.81 (s, 3H). **^{13}C NMR** (126 MHz, $CDCl_3$) δ 154.26, 151.11, 142.34, 136.19, 129.08, 128.06, 127.80, 123.31, 122.27, 120.24, 107.36, 61.02, 60.89, 56.16, 36.78. **HRMS**: m/z M^+ calcd. for $C_{17}H_{17}NO_3$: 283.12029; found: 283.11905 (-4.38 ppm). **IR**: 2935, 2211, 1601, 1559, 1539, 1517, 1492, 1465, 1417, 1313, 1258, 1170, 1144, 1095, 1027, 796, 750, 698, 667.

rac-**1v** 3-methyl-2-(3,4,5-trimethoxyphenyl)butanenitrile

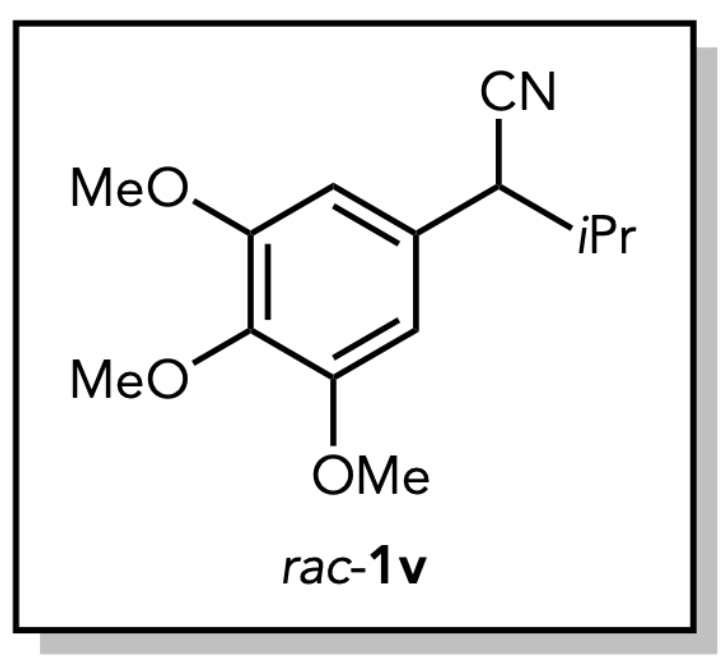

The title compound was synthesized according to **General Procedure A** from 2-(3,4,5-trimethoxyphenyl)acetonitrile (1.2 g, 6.0 mmol, 1.0 equiv), KO^tBu (670 mg, 6.0 mmol, 1.0 equiv), and 2-iodopropane (1.0 g, 600 µL, 6.0 mmol, 1.0 equiv). The crude material was purified via silica gel chromatography (0–15% EtOAc/hexanes) to afford the title compound as a colorless oil in 83% yield (1.2 g, 5.0 mmol). **^{1}H NMR** (500 MHz, $CDCl_3$) δ 6.50 (s, 2H), 3.87 (s, 3H), 3.87 (s, 3H), 3.85 (s, 3H), 3.58 (dd, J = 6.5, 1.6 Hz, 1H), 2.17 – 2.05 (m, 1H), 1.09 – 1.03 (m, 6H). **^{13}C NMR** δ 153.58, 137.81, 130.71, 119.95, 105.09, 61.04, 56.39, 45.57, 33.99, 21.04, 19.10. **HRMS**: m/z M^+ calcd. for $C_{14}H_{19}NO_3$: 249.136494; found: 249.13588 (-2.46 ppm). **IR**: 2919, 2848, 1964, 1692, 1591, 1507, 1457, 1422, 1322, 1237, 1124, 1005, 750, 700, 649.

rac-**1w** 2-(4,8-dimethoxynaphthalen-1-yl)-2-phenylacetonitrile

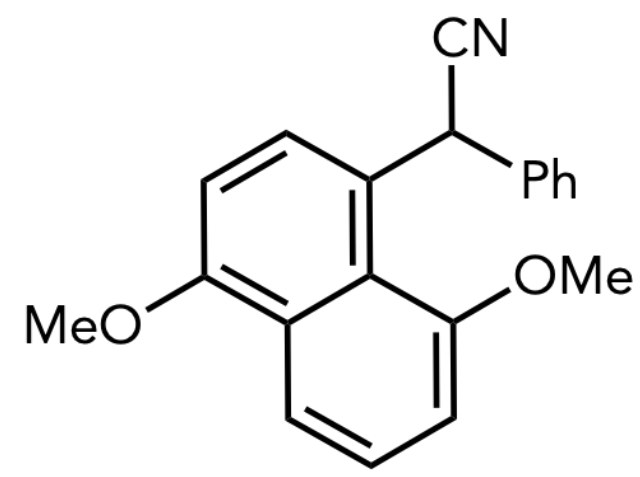

rac-**1w**

The title compound was synthesized according to **General Procedure C** from 4,8-dimethoxynaphthalene-2-carbaldehyde (650 mg, 3.0 mmol, 1.0 equiv) and phenylmagnesium bromide (3.0 M solution in Et_2O, 1.1 mL, 1.1 equiv). The crude material was purified via silica gel chromatography (0–30% EtOAc/hexanes) to afford the title compound as a white solid in 62% yield (570 mg, 1.9 mmol). **^{1}H NMR** (500 MHz, $CDCl_3$) δ 7.95 (d, *J* = 8.4 Hz, 1H), 7.43 – 7.37 (m, 2H), 7.35 – 7.23 (m, 5H), 6.90 (d, *J* = 7.7 Hz, 1H), 6.80 (d, *J* = 8.1 Hz, 1H), 6.71 (bs, 1H), 4.00 (s, 3H), 3.81 (s, 3H). **^{13}C NMR** (126 MHz, $CDCl_3$) δ 156.31, 155.62, 137.99, 128.86, 128.32, 127.77, 127.49, 125.84, 124.09, 121.49, 115.44, 107.46, 104.01, 55.81, 55.42. **^{13}C NMR** (126 MHz, DMSO-d_6)[27] δ 155.72, 154.87, 145.72, 137.97, 128.79, 128.07, 127.46, 127.32, 126.70, 126.22, 126.19, 123.02, 122.46, 121.30, 114.41, 107.62, 104.48, 74.25, 55.82, 55.15. **HRMS**: m/z M^+ calcd. for $C_{20}H_{17}NO_2$: 303.12538; found: 303.12423 (-3.79 ppm). **IR**: 2935, 2235, 1598, 1515, 1492, 1464, 1410, 1383, 1270, 1095, 1066, 868, 791, 752, 698.

rac-**1x** 2-(7-methoxynaphthalen-1-yl)-3-methylbutanenitrile

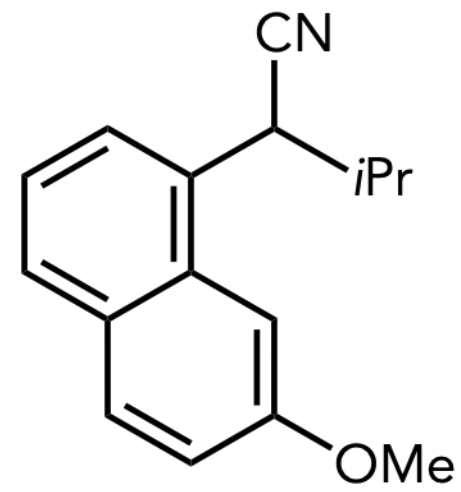

rac-**1x**

The title compound was synthesized according to **General Procedure A** from 2-(7-methoxy-1-naphthyl)acetonitrile (1.2 g, 6.0 mmol, 1.0 equiv), KO^{t}Bu (1.0 g, 9.0 mmol, 1.5 equiv), and 2-iodopropane (1.2 mL, 12.0 mmol, 2.0 equiv). The crude material was purified via silica gel chromatography (0–30% EtOAc/hexanes) to afford the title compound as a white solid in 34% yield (500 mg, 2.0 mmol). **^{1}H NMR** (500 MHz, $CDCl_3$) δ 7.81 (d, *J* = 9.0 Hz, 1H), 7.77 (d, *J* = 8.2 Hz, 1H), 7.62 (d, *J* = 7.1 Hz, 1H), 7.35 (dd, *J* = 8.2, 7.2 Hz, 1H), 7.20 (dd, *J* = 8.9, 2.4 Hz, 1H), 7.12 (d, *J* = 2.4 Hz, 1H), 4.35 (d, *J* = 5.4 Hz, 1H), 3.95 (s, 3H), 2.42 – 2.32 (m, 1H), 1.18 (d, *J* = 6.8 Hz, 3H), 1.08 (d, *J* = 6.6 Hz, 3H). **^{13}C NMR** (126 MHz, $CDCl_3$) δ 158.36, 131.54, 131.02, 129.64, 129.57, 128.73, 127.09, 123.15, 119.97, 118.18, 101.81, 55.50, 42.59, 32.01, 21.72, 18.89. **HRMS**: m/z M^+ calcd. for $C_{16}H_{17}NO$: 239.13046; found: 239.12904 (-5.93 ppm). **IR**: 2929, 2234, 1626, 1598, 1513, 1492, 1450, 1410, 1383, 1327, 1214, 1096, 1066, 1030, 829, 792, 745, 700.

rac-**1y** 2-(6-methoxynaphthalen-2-yl)-3-methylbutanenitrile

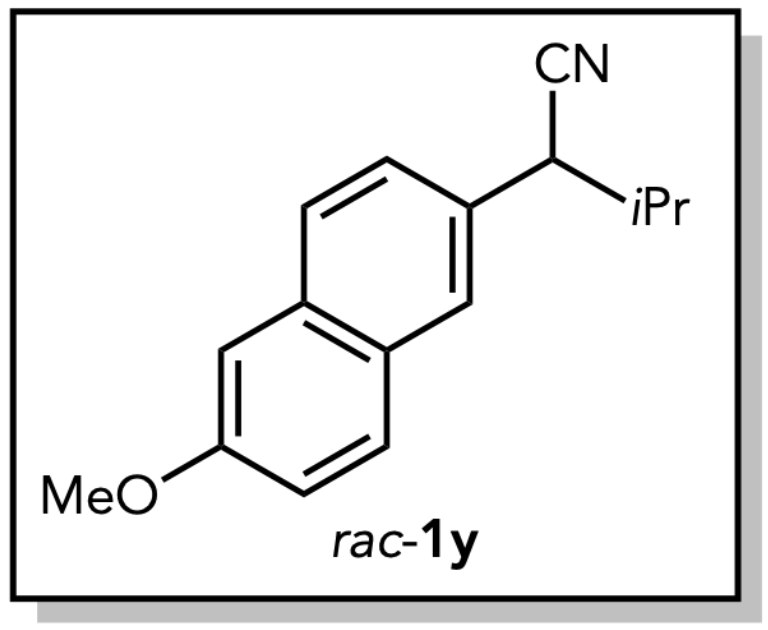

The title compound was synthesized according to **General Procedure C** from 6-methoxynaphthalene-2-carbaldehyde (1.1 g, 6.0 mmol, 1.0 equiv) and isopropylmagnesium chloride (2.0 M solution in THF, 6.0 mL, 12.0 mmol, 2.0 equiv). The crude material was purified via silica gel chromatography (0–30% EtOAc/hexanes) to afford the title compound as a translucent oil in 24% yield (350 mg, 1.5 mmol). **^{1}H NMR** (500 MHz, $CDCl_3$) δ 7.74 (dd, *J* = 8.7, 2.7 Hz, 2H), 7.71 (s, 1H), 7.34 (dd, *J* = 8.5, 2.0 Hz, 1H), 7.19 (dd, *J* = 9.0, 2.5 Hz, 1H), 7.14 (d, *J* = 2.5 Hz, 1H), 3.93 (s, 3H), 3.79 (d, *J* = 6.3 Hz, 1H), 2.26 – 2.17 (m, *J* = 6.7 Hz, 1H), 1.09 (d, *J* = 6.8 Hz, 3H), 1.06 (d, *J* = 6.7 Hz, 3H). **^{13}C NMR** (126 MHz, $CDCl_3$) δ 158.23, 134.14, 130.09, 129.50, 128.78, 127.65, 126.94, 126.03, 120.16, 119.66, 105.76, 55.51, 45.29, 33.90, 21.04, 19.01. **HRMS**: m/z $[M+H]^+$ calcd. for $C_{16}H_{20}NO$: 240.13828 found: 240.13706 (-5.08 ppm). **IR**: 2963, 2239, 1631, 1605, 1507, 1482, 1462, 1419, 1389, 1325, 1264, 1229, 1220, 1164, 1125, 1068, 1029, 890, 851, 810, 756, 668, 475.

rac-**1z** 2-cyclopentyl-2-(7-methoxynaphthalen-1-yl)acetonitrile

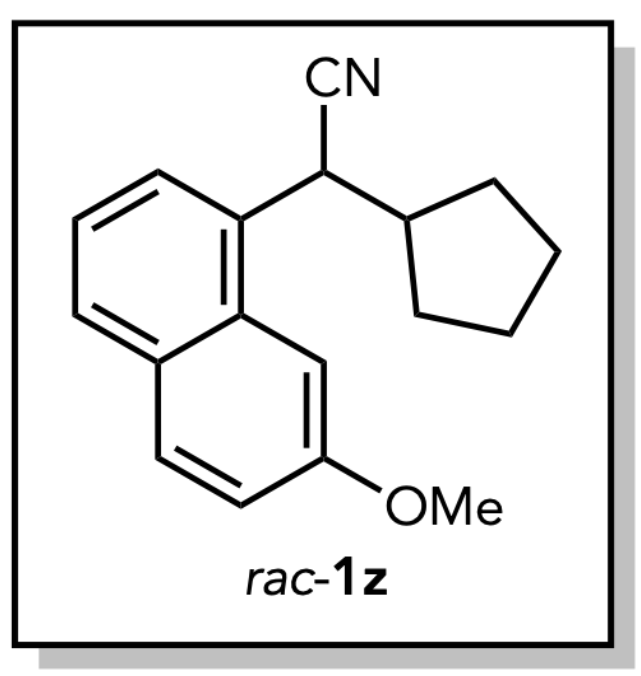

The title compound was synthesized according to **General Procedure A** from 2-(7-methoxy-1-naphthyl)acetonitrile (1.2 g, 6.0 mmol, 1.0 equiv), KO^{t}Bu (810 mg, 7.2 mmol, 1.2 equiv) and bromocyclopentane (1.3 mL, 12.0 mmol, 2.0 equiv). The crude material was purified via silica gel chromatography (0–30% EtOAc/hexanes) to afford the title compound as a white solid in 59% yield (943 mg, 3.55 mmol) . **^{1}H NMR** (500 MHz, $CDCl_3$) δ 7.81 (d, *J* = 9.8 Hz, 1H), 7.76 (d, *J* = 8.1 Hz, 1H), 7.61 (d, *J* = 7.2 Hz, 1H), 7.37 – 7.31 (m, 1H), 7.23 – 7.19 (m, 2H), 4.42 (d, *J* = 6.8 Hz, 1H), 3.96 (s, 3H), 2.64 – 2.53 (m, 1H), 1.84 – 1.71 (m, 4H), 1.70 – 1.41 (m, 4H). **^{13}C NMR** (126 MHz, $CDCl_3$) δ 158.35, 131.52, 130.96, 130.25, 129.60, 128.67, 126.66, 123.26, 120.69, 118.24, 101.82, 55.56, 43.40, 39.57, 31.48, 30.40, 25.22, 25.05. **HRMS**: m/z $[M+H]^+$ calcd. for $C_{18}H_{20}NO$: 266.15393 found: 266.15283 (-4.13 ppm). **IR**: 3018, 2955, 2160, 1627, 1602, 1512, 1470, 1449, 1387, 1259, 1213, 1026, 902, 829, 745, 703, 667, 647, 516.

rac-**1aa** 2-(2-fluoro-4,5-dimethoxyphenyl)-3-methylbutanenitrile

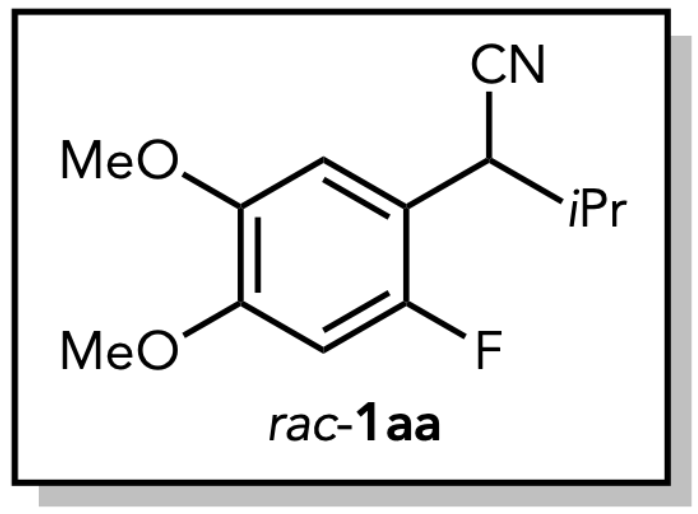

The title compound was synthesized according to **General Procedure C** from 2-fluoro-4,5-dimethoxy-benzaldehyde (1.1 g, 6.0 mmol, 1.0 equiv) and isopropylmagnesium chloride (2.0 M solution in THF, 6.0 mL, 12 mmol, 2.0 equiv). The crude material was purified via silica gel chromatography (0–30% EtOAc/hexanes) to afford the title compound as a translucent oil in 49% yield (710 mg, 3.0 mmol). **^{1}H NMR** (500 MHz, $CDCl_3$) δ 6.84 (d, *J* = 7.0 Hz, 1H), 6.63 (d, *J* = 11.1 Hz, 1H), 3.90 (d, *J* = 6.9 Hz, 1H), 3.88 (s, 3H), 3.86 (s, 3H), 2.18 – 2.08 (m, 1H), 1.08 (d, *J* = 6.7 Hz, 3H), 1.02 (d, *J* = 6.7 Hz, 3H). **^{13}C NMR** (126 MHz, $CDCl_3$) δ 154.19 (d, *J* = 240.3 Hz), 149.84 (d, *J* = 9.9 Hz), 145.68 (d, *J* = 2.5 Hz), 119.67, 112.70 (d, *J* = 15.5 Hz), 111.26 (d, *J* = 4.8 Hz), 100.20 (d, *J* = 28.0 Hz), 56.68, 56.34, 38.20 (d, *J* = 1.9 Hz), 32.98 (d, *J* = 1.2 Hz), 20.54, 19.36. **^{19}F NMR** (471 MHz, $CDCl_3$) δ -125.16 (dd, *J* = 11.3, 7.0 Hz). **HRMS**: m/z M^+ calcd. for $C_{13}H_{16}FNO_2$: 237.11596, found: 237.11496 (-4.21 ppm). **IR**: 2961, 2244, 1624, 1508, 1443, 1404, 1371, 1304, 1266, 1211, 1192, 1131, 1102, 1027, 1001, 919, 860, 830, 755, 700.

rac-**1ab** 6-methoxychromane-4-carbonitrile

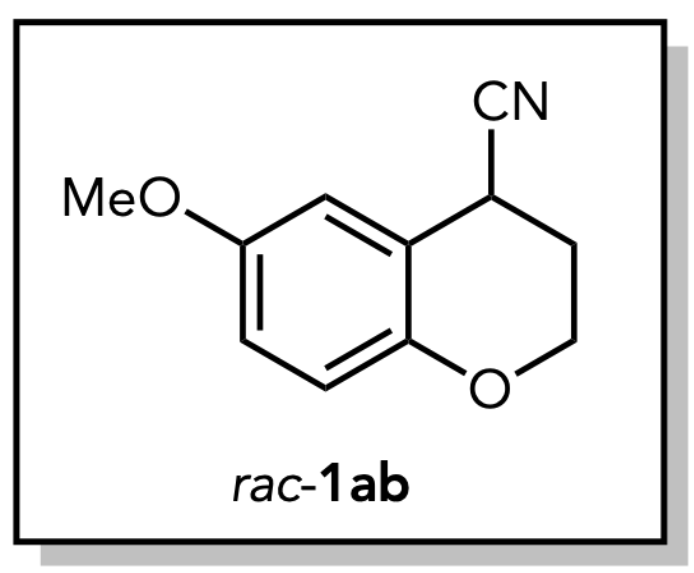

The title compound was synthesized according to **General Procedure D** from 6-methoxychroman-4-one (1.1 g, 6.0 mmol, 1.0 equiv) and $NaBH_4$ (450 mg, 12 mmol, 2.0 equiv). The crude material was purified via silica gel chromatography (0–30% EtOAc/hexanes) to afford the title compound as a translucent oil in 77% yield (870 mg, 4.6 mmol). **^{1}H NMR** (500 MHz, $CDCl_3$) δ 6.83 – 6.76 (m, 3H), 4.28 (ddd, *J* = 11.0, 7.0, 3.7 Hz, 1H), 4.18 (ddd, *J* = 11.4, 6.3, 3.8 Hz, 1H), 4.00 (t, *J* = 6.1 Hz, 1H), 3.77 (s, 3H), 2.37 – 2.26 (m, 2H). **^{13}C NMR** (126 MHz, $CDCl_3$) δ 153.97, 148.11, 120.58, 118.68, 116.85, 115.44, 113.23, 63.63, 55.93, 27.21, 26.37. **HRMS**: m/z $[M+H]^+$ calcd. for $C_{11}H_{12}NO_2$: 190.08625 found: 190.08547 (-4.10 ppm). **IR**: 2935, 2238, 1653, 1624, 1496, 1465, 1266, 1209, 1064, 1034, 817, 754.

rac-**1ac** 8-methoxychromane-4-carbonitrile

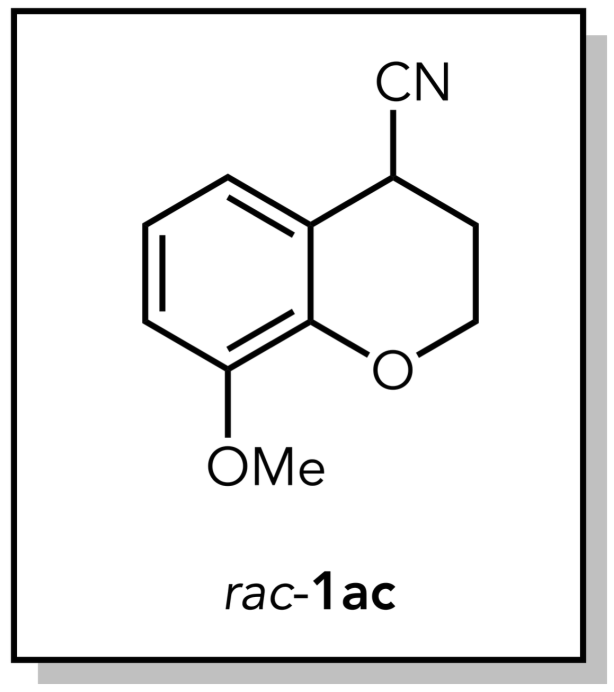

The title compound was synthesized according to **General Procedure D** from 8-methoxychroman-4-one (1.00 g, 5.61 mmol, 1.00 equiv) and $NaBH_4$ (425 mg, 11.22 mmol, 2.00 equiv). The crude material was purified via silica gel chromatography (0–30% EtOAc/hexanes) to afford the title compound as a white solid in 67% yield (720 mg, 3.8 mmol). **^{1}H NMR** (500 MHz, $CDCl_3$) δ 6.93 – 6.89 (m, 2H), 6.86 – 6.81 (m, 1H), 4.41 (ddd, *J* = 11.2, 6.9, 4.1 Hz, 1H), 4.33 (dtd, *J* = 10.7, 4.0, 1.9 Hz, 1H), 4.03 (t, *J* = 6.0 Hz, 1H), 3.87 (s, 3H), 2.34 (dtt, *J* = 7.7, 6.0, 4.2 Hz, 2H). **^{13}C NMR** (126 MHz, $CDCl_3$) δ 148.88, 143.80, 121.02, 120.93, 120.50, 115.93, 111.43, 64.12, 56.12, 26.84, 26.06. **HRMS**: m/z $[M+Na]^+$ calcd. for $C_{11}H_{11}NO_2Na$: 212.06820; found: 212.06823 (0.14 ppm) **IR**: 2938, 2242, 1583, 1478, 1456, 1435, 1333, 1267, 1248, 1214, 1183, 1098, 1078, 1054, 1016, 852, 798, 782, 743, 700, 619, 557, 515.

rac-**1ad** (3*S*)-2-(3,4-dimethoxyphenyl)-3-methylpentanenitrile

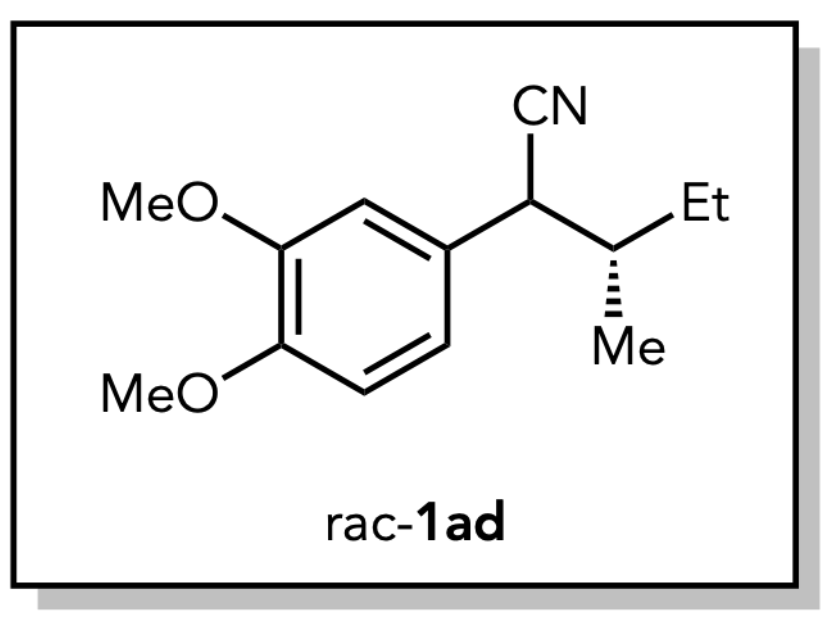

The title compound was synthesized according to **General Procedure A** from 2-(3,4-dimethoxyphenyl)acetonitrile (2.0 g, 11 mmol, 1.0 equiv), KO^tBu (1.2 g, 11.0 mmol, 1.0 equiv) and 2-bromobutane (1.6 g, 1.2 mL, 11 mmol, 1.0 equiv). The crude material was purified via silica gel chromatography (0–15% EtOAc/hexanes) to afford the title compound as a clear oil in 64% yield (1.69 g, 7.24 mmol). The oil was submitted to LOTUS Separations and separated into four diastereomers (ColumnTek Enantiocel® C2-5 (500 x 30 mm) 85 mL/min @ 15% methanol / 85% CO_2 (100 bar backpressure); 220 nm and ColumnTek Enantiocel® C9-5 (500 x 30 mm) 85 mL/min @ 15% methanol / 85% CO_2 (100 bar backpressure); 220 nm). Diastereomers were analyzed by HPLC and two were recombined to obtain the title compound (1:1 dr, >99% ee) as a white solid. **^{1}H NMR** (500 MHz, CDCl3) δ 6.86 – 6.83 (m, 2H), 6.79 (s, 1H), 3.90 (s, 3H), 3.88 (s, 3H), 3.78 (d, J = 5.7 Hz, 1H), 3.62 (d, J = 6.7 Hz, 1H), 1.92 – 1.78 (m, 1H), 1.68 – 1.51 (m, 1H), 1.42 – 1.24 (m, 1H), 0.99 – 0.90 (m, 6H).**^{13}C NMR** (126 MHz, $CDCl_3$) δ 149.30, 148.88, 148.80, 127.59,

127.26, 120.63, 120.29, 119.94, 111.35, 111.30, 111.10, 110.90, 56.15, 56.09, 43.75, 43.04, 40.51, 40.04, 27.84, 26.01, 17.00, 15.68. **HRMS**: m/z $[M+H]^+$ calcd. for $C_{14}H_{20}NO_2$: 234.14885; found: 234.14847 (-1.62 ppm). **IR**: 2962, 2931, 2235, 1653, 1592, 1515, 1457, 1419, 1383, 1238, 1144, 1026, 855, 802, 763, 618.

rac-**1ae** 2-(3,4-dimethoxyphenyl)-3-phenylpropanenitrile

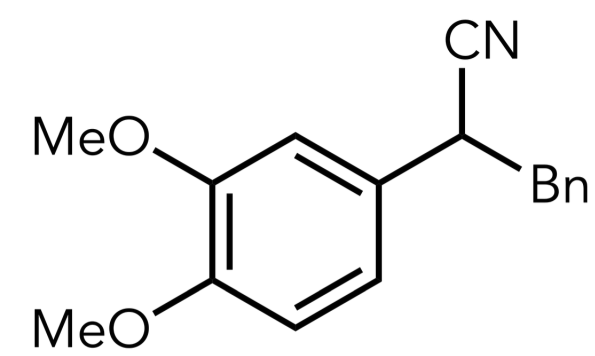

rac-**1ae**

The title compound was synthesized according to **General Procedure A** from 2-(3,4-dimethoxyphenyl)acetonitrile (1.1 g, 6.0 mmol, 1.0 equiv), LiHMDS (1.0 g, 6.0 mmol, 1.0 equiv), and benzyl bromide (1.0 g, 710 µL, 6.0 mmol, 1.0 equiv). The crude material was purified by silica gel chromatography (0–30% EtOAc/hexanes) to afford the title compound as a white solid in 31% yield (500 mg, 1.9 mmol). ^{1}**H NMR** (500 MHz, $CDCl_3$) δ 7.36 – 7.25 (m, 3H), 7.17 – 7.11 (m, 2H), 6.88 – 6.79 (m, 2H), 6.66 (d, J = 1.9 Hz, 1H), 3.97 (dd, J = 8.0, 6.6 Hz, 1H), 3.90 (s, 3H), 3.83 (s, 3H), 3.22 (dd, J = 13.5, 7.9 Hz, 1H), 3.14 (dd, J = 13.5, 6.6 Hz, 1H). ^{13}**C NMR** (126 MHz, $CDCl_3$) δ 149.27, 149.01, 136.44, 129.46, 128.74, 128.25, 127.60, 127.48, 120.73, 119.92, 111.43, 110.73, 56.09, 56.05, 42.39, 39.42. **HRMS**: m/z M^+ calcd. for $C_{17}H_{17}NO_2$: 267.12593; found: 267.12478 (-4.30 ppm). **IR**: 2835, 2239, 1591, 1518, 1441, 1415, 1332, 1264, 1239, 1167, 1141, 1027, 892, 871, 798, 750, 736, 699.6, 633, 620, 563, 483.

rac-**3** 3-methyl-2-phenylbutanenitrile

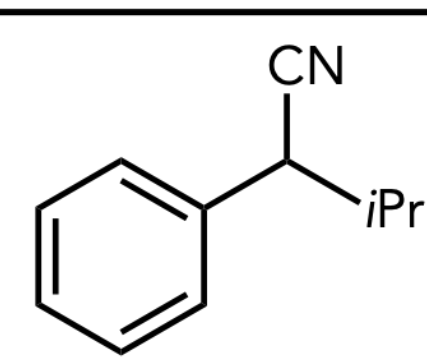

rac-**3**

The title compound was synthesized according to **General Procedure A** from phenylacetonitrile (1.3 g, 11 mmol, 1.0 equiv), KO^tBu (1.2 g, 11.0 mmol, 1.0 equiv), and 2-iodopropane (1.9 g, 1.1 mL, 11 mmol, 1.0 equiv). The crude material was purified by silica gel chromatography (0–30% EtOAc/hexanes) to afford the title compound as a clear colorless oil in 68% yield (1.2 g, 7.7 mmol). These characterization data were consistent with those reported in the literature.[8]

rac-**6** 2-(3,4-dimethoxyphenyl)-3-methylbutanamide

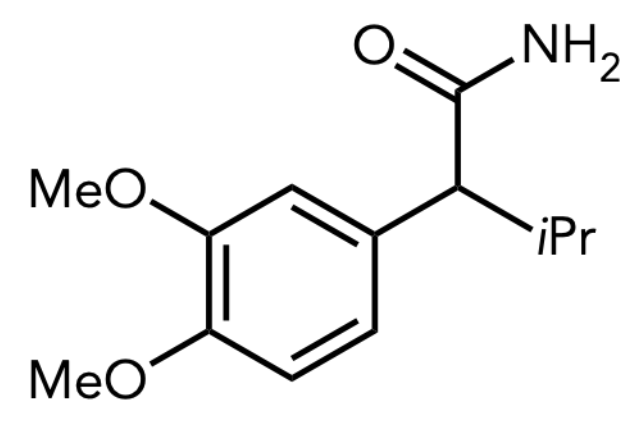

rac-**6**

[(DPPF)-Pt(PMe_2OH)Cl])Tf was prepared by literature procedure.[9]

A 2-dram vial equipped with a magnetic stir bar was charged with **1a** (150 mg, 0.684 mmol, 1.00 equiv), [(DPPF)-Pt(PMe_2OH)Cl])Tf (5.5 mg, 5.5 µmol, 0.8 mol%), silver triflate (1.4 mg, 5.5 µmol, 0.8 mol%), water (0.5 mL), and THF (0.5 mL). The vial was stirred at 40 ºC under air for 20 h. The reaction mixture was poured into brine (30 mL) and transferred to a separatory funnel. The aqueous phase extracted with EtOAc (3 × 30 mL). The combined organic layers were dried over Na_2SO_4, filtered, and concentrated *in vacuo*. The crude residue was purified by silica gel chromatography (25–100% EtOAc/hexanes) to afford the title compound as a white solid in 93% yield (151 mg, 0.632 mmol). **^{1}H NMR** (500 MHz, $CDCl_3$) δ 6.91 (d, J = 1.8 Hz, 1H), 6.84 – 6.76 (m, 2H), 5.45 (s, 1H), 5.34 (s, 1H), 3.87 (s, 3H), 3.86 (s, 3H), 2.85 (d, J = 10.1 Hz, 1H), 2.34 (dp, J = 10.1, 6.6 Hz, 1H), 1.06 (d, J = 6.5 Hz, 3H), 0.72 (d, J = 6.7 Hz, 3H). **^{13}C NMR** (126 MHz, $CDCl_3$) δ 175.95, 149.19, 148.39, 131.98, 120.70, 111.08, 111.02, 60.74, 56.04, 55.99, 31.41, 21.72, 20.44. **HRMS**: m/z $[M+H]^+$ calcd. for $C_{13}H_{20}NO_3$: 238.14377; found: 238.1441 (1.39 ppm). **IR**: 3405, 3133, 2962, 1676, 1512, 1465, 1397, 1268, 1229, 1187, 1125, 1024, 888, 845, 819, 794, 761, 698, 672, 613, 574, 459.

4. Cyclic Voltammetry Experiments

Cyclic voltammetry experiments were performed on a CH Instruments 600E potentiostat using CHI600E software. Experiments were run at a scan rate of 0.1 V/s in a 25 mL three-neck round-bottom flask equipped a glassy carbon working electrode, a platinum mesh counter electrode, and an $AgNO_3$/Ag pseudo-reference electrode or AgCl/Ag pseudo-reference electrode. All measurements against each pseudo-reference electrode were then referenced to the Fc^+/Fc redox couple. Experiments were carried out as follows: a flame-dried round-bottom flask was charged with 1.0 mmol $N(^nBu)_4PF_6$ electrolyte and 10 mL dry solvent. The solution was sparged with nitrogen for 5 minutes, and a blank voltammogram was collected. The analyte (0.01 mmol) was then dissolved in the electrolyte solution, and the solution was sparged with N_2 for 5 minutes prior to recording the second voltammogram with analyte. Between each measurement, the Ag^+/Ag and platinum mesh electrodes were rinsed with MeCN and water, and the glassy carbon electrode was polished on CH Instruments MicroPolish alumina powder (0.05 μm).

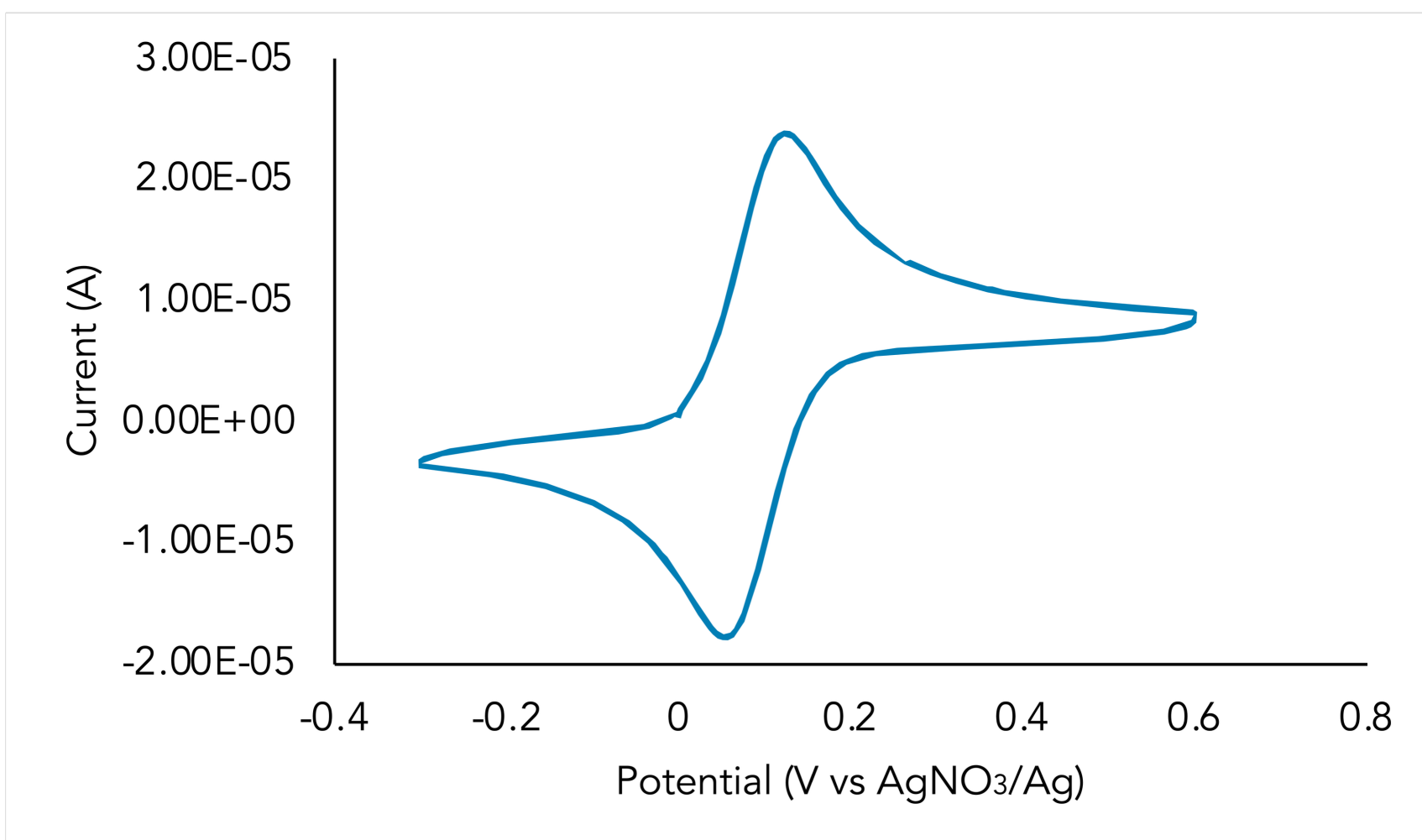

$E_{1/2}$ = +0.09 V vs $AgNO_3$/Ag

Figure S4.1. Cyclic voltammogram of ferrocene in MeCN with NBu_4PF_6 electrolyte.

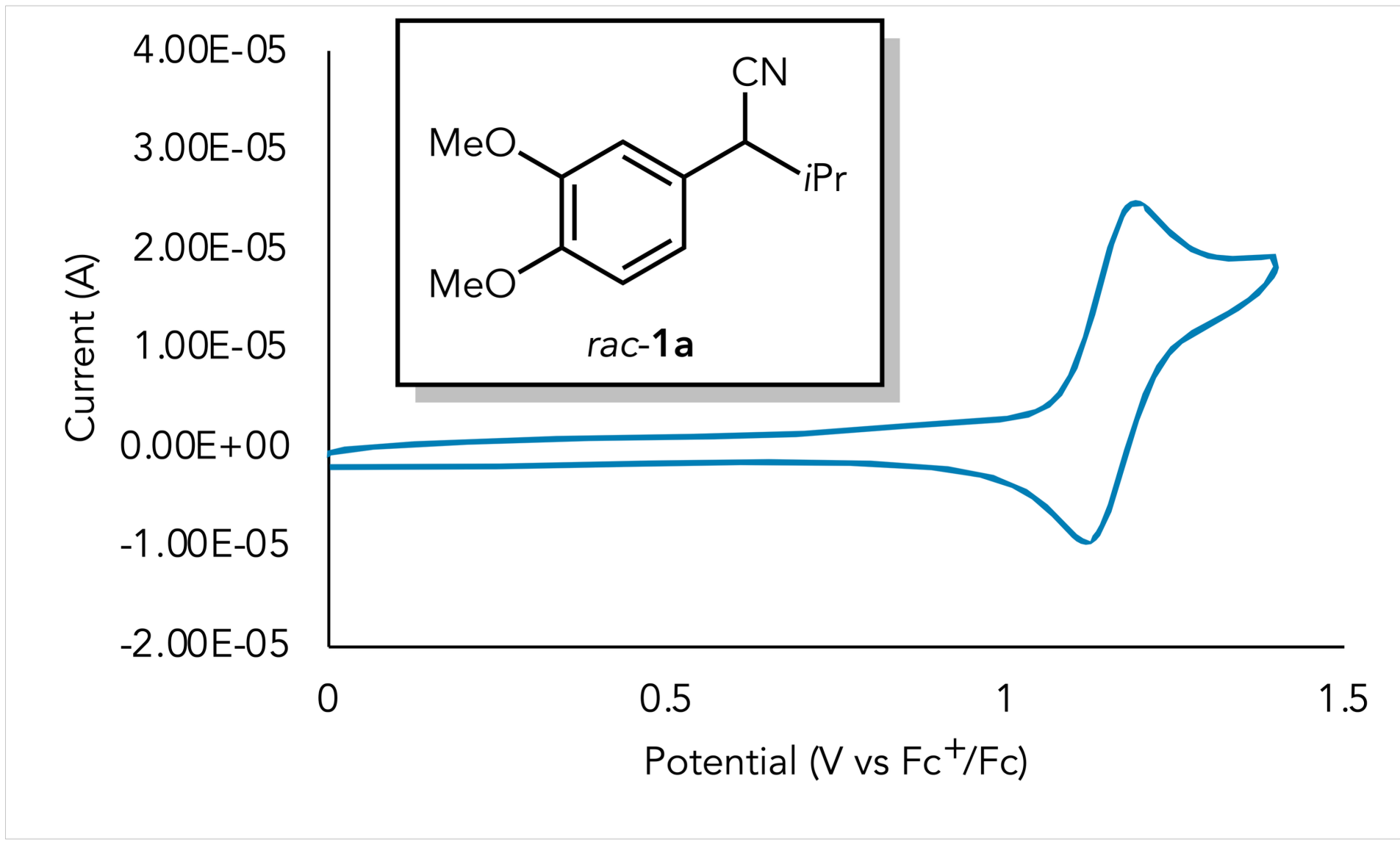

$E_{p/2}$ = +1.04 V vs Fc$^+$/Fc

Figure S4.2. Cyclic voltammogram of **1a** in MeCN with NBu_4PF_6 electrolyte.

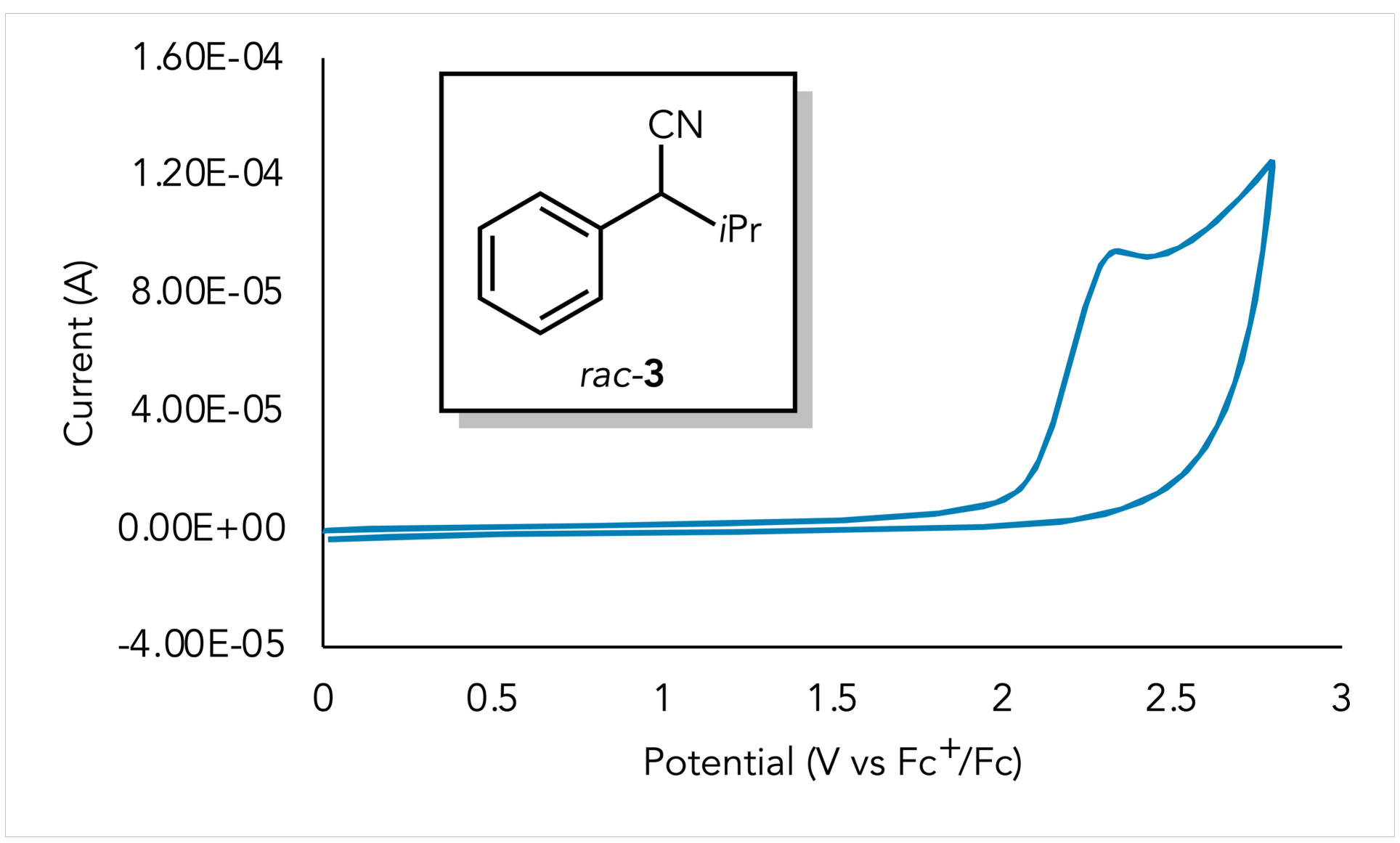

$E_{p/2}$ of **3** = +2.09 V vs Fc$^+$/Fc

Figure S4.3. Cyclic voltammogram of **3** in MeCN with NBu_4PF_6 electrolyte.

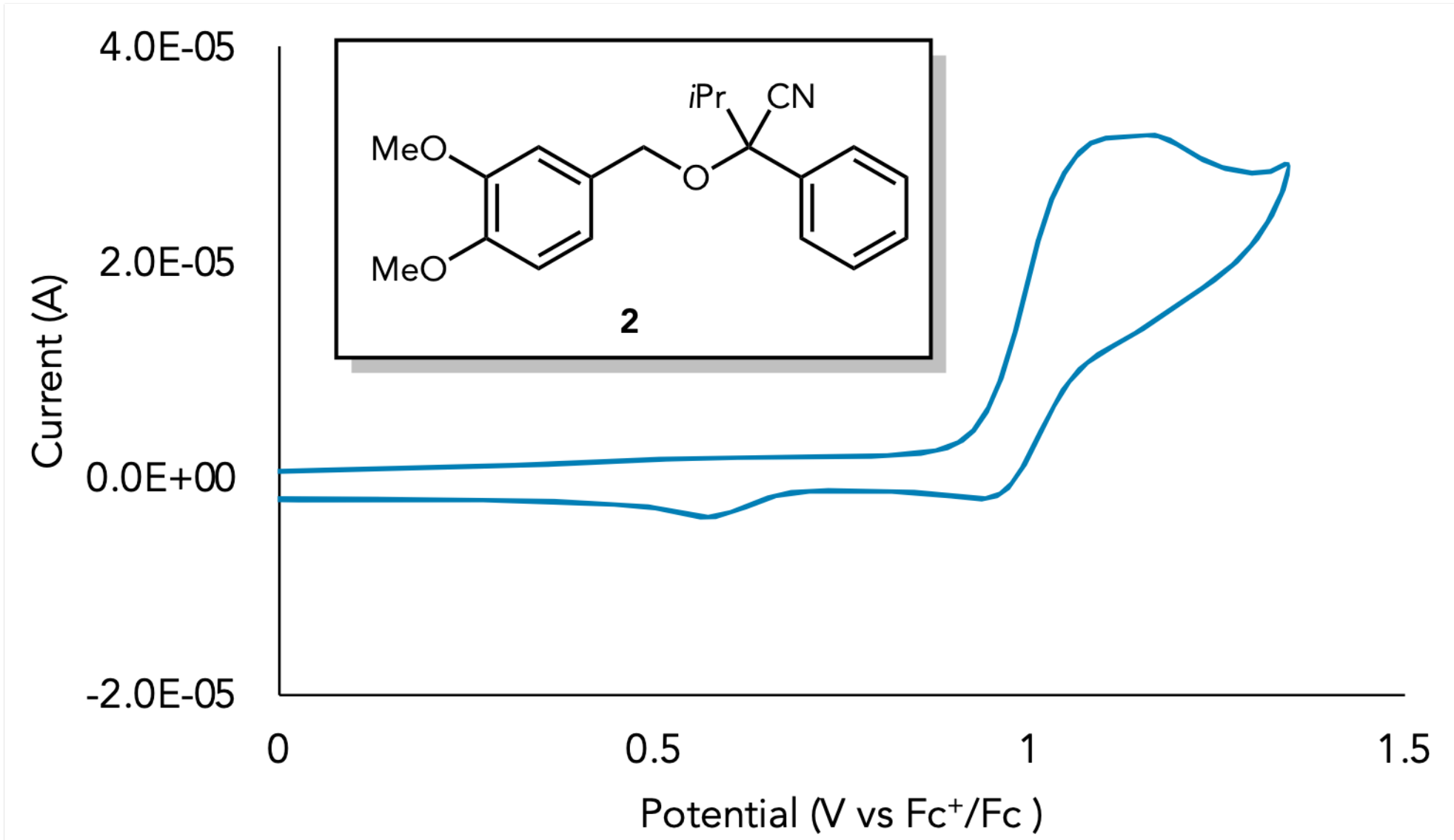

$E_{p/2}$ = +1.01 V vs Fc^{+}/Fc in MeCN

Figure S4.4. Cyclic voltammogram of **2** in MeCN with NBu_4PF_6 electrolyte.

5. Deracemization of Benzylic Nitriles

5.1 General Procedure for Deracemization of Benzylic Nitriles

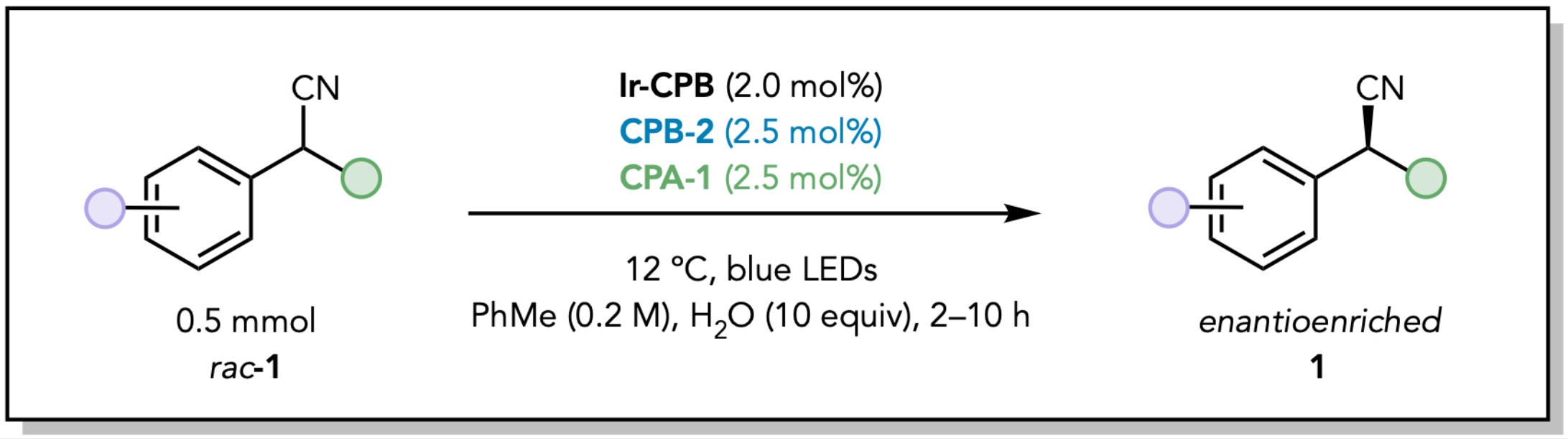

An oven-dried 2-dram vial equipped with a magnetic stir bar was charged with racemic nitrile (0.50 mmol, 1.00 equiv), **Ir-CPB** (10.0 µmol, 2.00 mol%), **CPB-2** (12.5 µmol, 2.50 mol%), and **CPA-1** (12.5 µmol, 2.50 mol%). The vial was imported into an N_2 atmosphere glovebox and charged with toluene (2.50 mL, 0.20 M). The vial was sealed with a Teflon septum cap and the junction between the cap and vial wrapped with electrical tape. The reaction mixture was exported from the glovebox and charged with degassed H_2O or D_2O (100 µL, 10 equiv) via Hamilton syringe, then the top of the vial cap was sealed with electrical tape. The reaction was then placed in a photoreactor equipped with a recirculating ice-water bath and 456 nm Kessil lamps (PR160L) set to 100% intensity and irradiated for 2 h unless otherwise noted. Upon completion, the reaction mixture was opened to air and transferred directly to a silica gel column. The reaction vial was then washed with toluene (2 × 1 mL). The product was isolated using flash column chromatography on silica (EtOAc/hexanes).

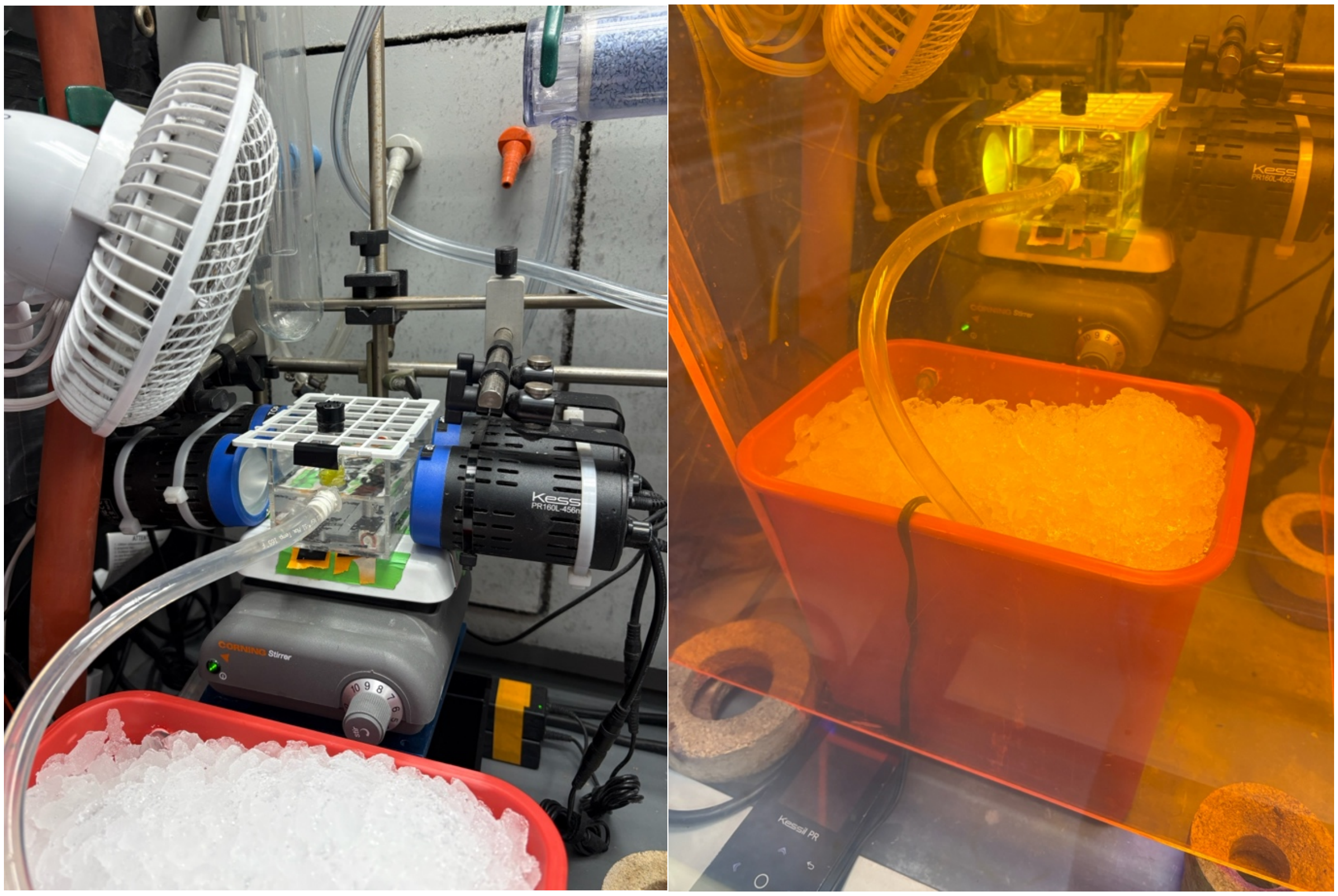

Figure S5.1. Standard photoreactor setup for 0.5 mmol scale reaction.

5.2 General Procedure for Gram-Scale Deracemization of Benzylic Nitriles

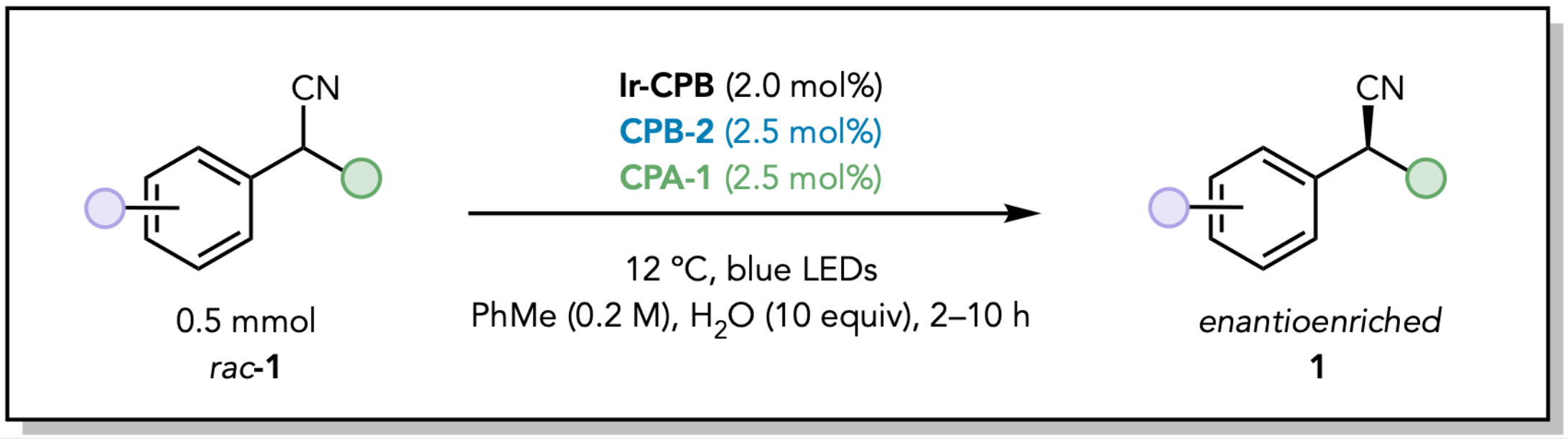

An oven-dried 40 mL vial equipped with a magnetic stir bar was charged with racemic nitrile (4.56 mmol, 1.00 equiv), **Ir-CPB** (45.6 µmol, 1.00 mol%), **CPB-2** (51.5 µmol, 1.25 mol%), and **CPA-1** (51.5 µmol, 1.25 mol%). The vial was imported into an N_2 atmosphere glovebox and charged with toluene (22.8 mL, 0.20 M). The vial was sealed with a Teflon septum cap and the junction between the cap and vial wrapped with electrical tape. The reaction mixture was exported from the glovebox and charged with degassed H_2O (822 µL, 10.0 equiv) via Hamilton syringe, then the top of the vial cap was sealed with electrical tape. The reaction was then placed in a photoreactor equipped with a recirculating ice-water bath and 456 nm Kessil lamps (PR160L) set to 100% intensity and irradiated for 2 h unless otherwise noted. Upon completion, the reaction mixture was opened to air and transferred directly to a silica gel column. The reaction vial was then washed with toluene (2 × 1 mL). The product was isolated using flash column chromatography on silica (EtOAc/hexanes).

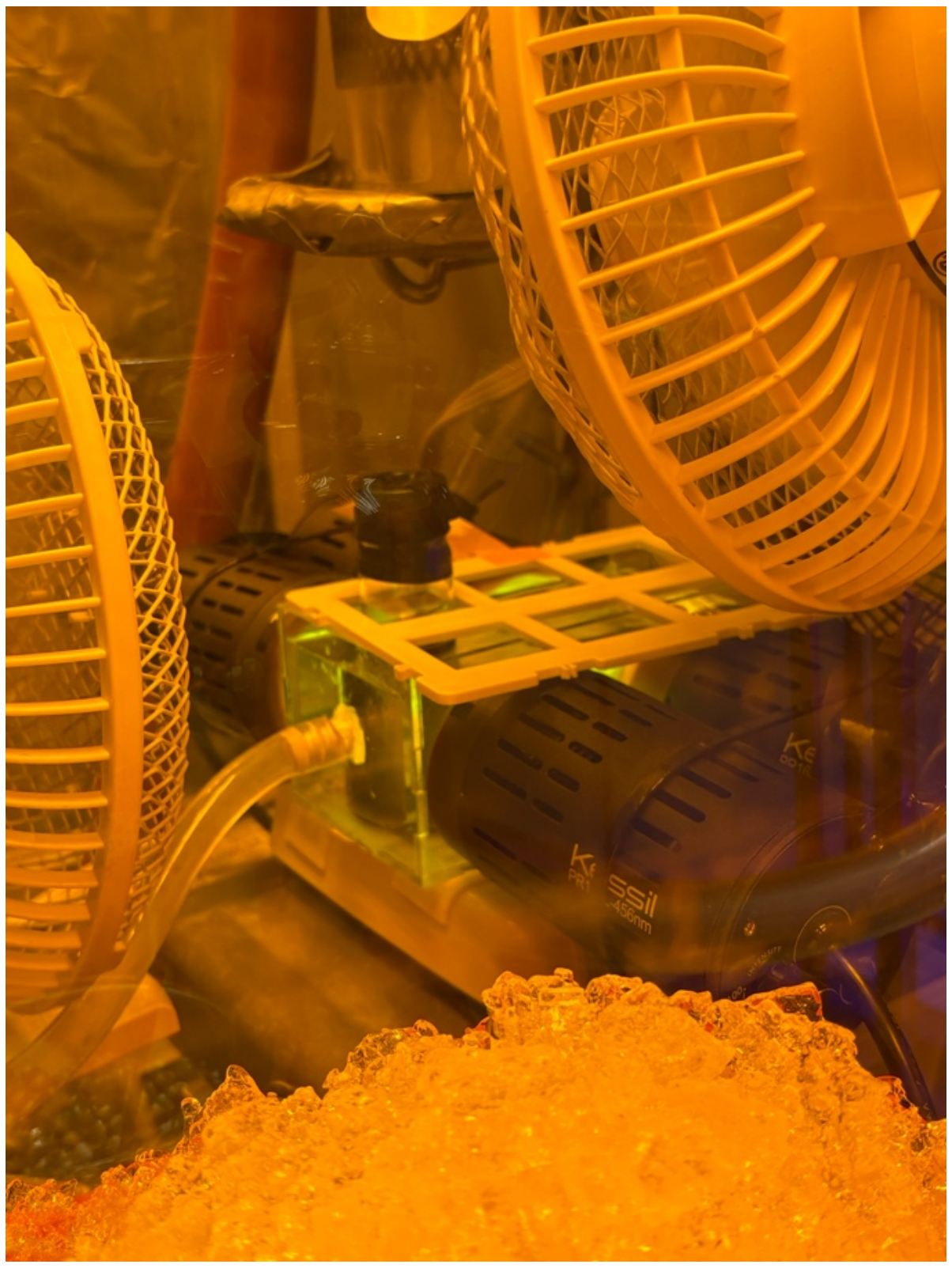

Figure S5.2. Photoreactor setup for 1 gram reaction.

(*S*)-**1a**

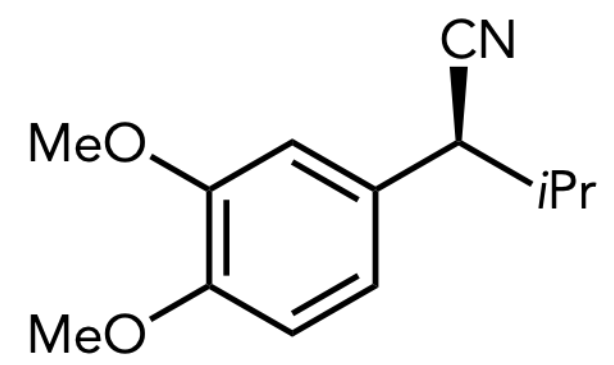

(*S*)-**1a**

(*S*)-**1a** was prepared according to the **General Deracemization Procedure** using (*rac*)-**1a** with **Ir-CPB** (19.5 mg, 10.0 μmol, 2.00 mol%), **CPB-2** (10.5 mg, 12.5 μmol, 2.50 mol%), **CPA-1** (9.40 mg, 12.5 μmol. 2.5 mol%), and toluene (0.20 M) as solvent. After 2 h of irradiation, the reaction was stopped and silica gel chromatography (0–30% EtOAc/hexanes) was performed to obtain the title compound in 96% yield and 97% ee as an average of two experiments. **HPLC** (10% isopropyl alcohol in hexanes, ChiralPak® AD-H, 1.0 mL/min, 230 nm detection): t_R(major) = 7.7 min, t_R(minor) = 8.9 min. **$[\alpha]_D$ =** -26.7º (c = 0.20, $CHCl_3$).

(*S*)-**1a** was prepared according to the **General Gram-Scale Deracemization Procedure** using (*rac*)-**1a** with **Ir-CPB** (89.1 mg, 45.6 μmol, 1.00 mol%), **CPB-2** (43.6 mg, 51.5 μmol, 1.25 mol%), **CPA-1** (38.8 mg, 51.5 μmol, 1.25 mol%), and toluene (0.20 M) as solvent. After 3 h of irradiation, the reaction was stopped and silica gel chromatography (0–30% EtOAc/hexanes) was performed to obtain the title compound in 91% yield and 95% ee.

(*S*)-**1a-*d*$_1$** (*S*)-2-(3,4-dimethoxyphenyl)-3-methylbutanenitrile-2-*d*

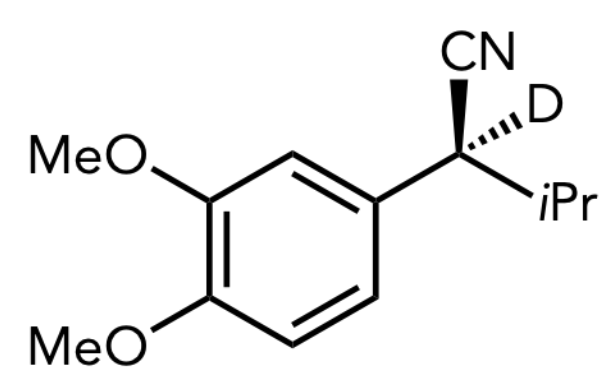

(*S*)-**1a-*d*$_1$**

(*S*)-**1a-*d*$_1$** was prepared according to the **General Deracemization Procedure** using (*rac*)-**1a** with **Ir-CPB** (19.5 mg, 10.0 μmol, 2.0 mol%), **CPB-2** (10.5 mg, 12.5 μmol, 2.5 mol%), **CPA-1** (9.4 mg, 12.5 μmol. 2.5 mol%), D_2O (100 μL) and toluene (0.2 M) as solvent. After 2 h of irradiation, the reaction was stopped and silica gel chromatography (0–30% EtOAc/hexanes) was performed to obtain the title compound in 96% yield, 93% D, and 98% ee as an average of two experiments. **HPLC** (10% isopropyl alcohol in hexanes, ChiralPak AD-H, 1.0 mL/min, 230 nm detection): t_R(major) = 7.7 min, t_R(minor) = 8.9 min. **$[\alpha]_D$ =** -29.0º (c = 0.23, $CHCl_3$). **^{1}H NMR** (500 MHz, $CDCl_3$) δ 6.85 – 6.76 (m, 3H), 3.89 (s, 3H), 3.88 (s, 3H), 2.14 – 2.05 (m, 1H), 1.05 (s, 3H), 1.03 (s, 3H). **^{13}C NMR** (126 MHz, $CDCl_3$) δ 149.29, 148.87, 127.51, 127.47, 120.39, 120.21, 111.32, 110.92, 56.14, 56.09, 44.90-44.41 (m), 33.98, 33.89, 20.89, 20.86, 19.09, 19.07. **HRMS** m/z M^+ calcd. for $C_{13}H_{17}DNO_2$: 220.13166, found: 220.13095 (-3.22 ppm).

(*S*)-**1b**

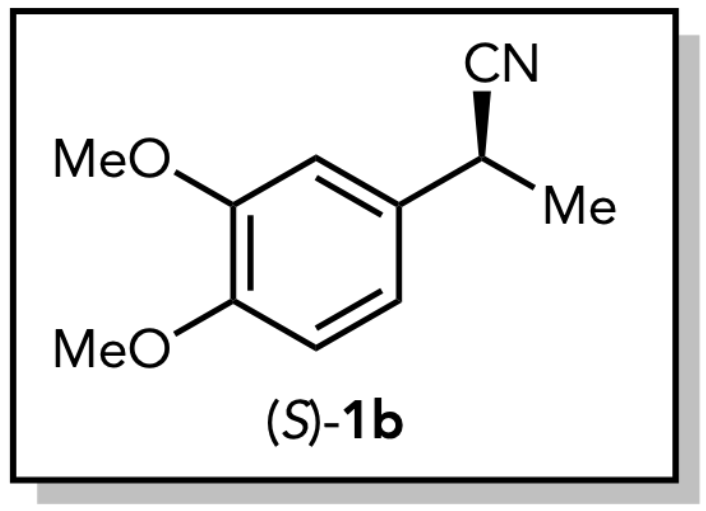

(*S*)-**1b** was prepared according to the **General Deracemization Procedure** using (*rac*)-**1b** with **Ir-CPB** (19.5 mg, 10.0 µmol, 2.0 mol%), **CPB-2** (10.5 mg, 12.5 µmol, 2.50 mol%), **CPA-1** (9.40 mg, 12.5 µmol. 2.50 mol%), and toluene (0.20 M) as solvent. After 3 h of irradiation, the reaction was stopped and silica gel chromatography (0–30% EtOAc/hexanes) was performed to obtain the title compound in 98% yield and 90% ee as an average of two experiments. **HPLC** (8% isopropyl alcohol in hexanes, ChiralPak AD-H, 1.0 mL/min, 230 nm detection): t_R(major) = 9.5 min and t_R(minor) = 10.9 min. **[α]$_D$ =** -21.1º (c = 0.12, $CHCl_3$).

(*S*)-**1c**

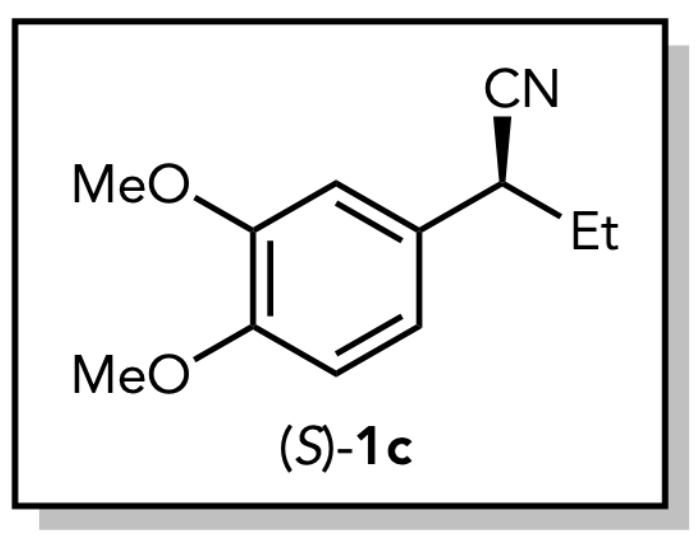

(*S*)-**1c** was prepared according to the **General Deracemization Procedure** using (*rac*)-**1c** with **Ir-CPB** (19.5 mg, 10.0 µmol, 2.0 mol%), **CPB-2** (10.5 mg, 12.5 µmol, 2.5 mol%), **CPA-1** (9.40 mg, 12.5 µmol. 2.50 mol%), and toluene (0.20 M) as solvent. After 3 h of irradiation, the reaction was stopped and silica gel chromatography (0–30% EtOAc/hexanes) was performed to obtain the title compound in 99% yield and 90% ee as an average of two experiments. **HPLC** (8% isopropyl alcohol in hexanes, ChiralPak AD-H, 1.0 mL/min, 230 nm detection): t_R(major) = 8.6 min and t_R(minor) = 10.4 min. **[α]$_D$ =** -30.8º (c = 0.10, $CHCl_3$).

(*S*)-**1d**

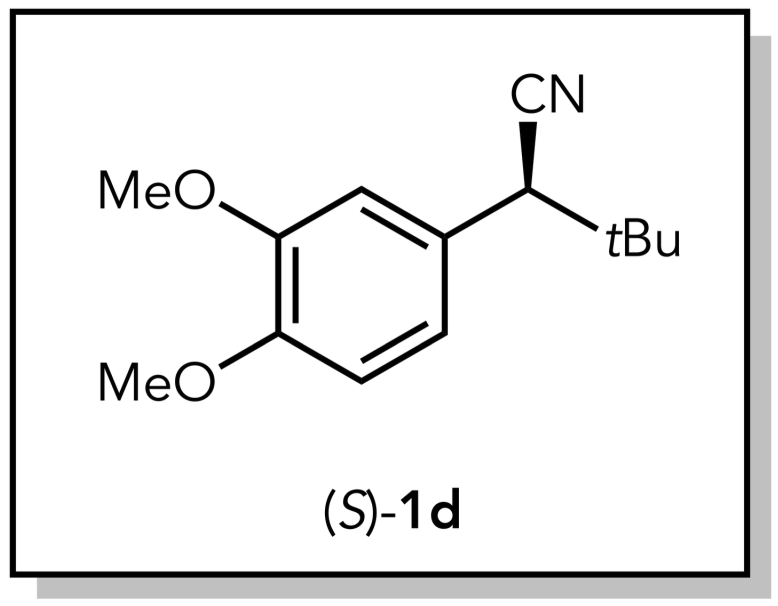

(*S*)-**1d** was prepared according to the **General Deracemization Procedure** using (*rac*)-**1d** with **Ir-CPB** (19.5 mg, 10.0 µmol, 2.0 mol%), **CPB-2** (10.5 mg, 12.5 µmol, 2.50 mol%), **CPA-1** (9.40 mg, 12.5 µmol. 2.50 mol%), and toluene (0.20 M). After 22 h of irradiation, the reaction was stopped and silica gel chromatography (0–15% EtOAc/hexanes) was performed to obtain the title compound in 99% yield and 90% ee as an average of two experiments.

Room Temperature Conditions:

(*S*)-**1d** was prepared according to the **General Deracemization Procedure** using (*rac*)-**1d** with **Ir-CPB** (19.5 mg, 10.0 µmol, 2.0 mol%), **CPB-2** (10.5 mg, 12.5 µmol, 2.50 mol%), **CPA-1** (9.40 mg, 12.5 µmol. 2.50 mol%), and toluene (0.20 M). After irradiation for 2 hours at 30 ºC, the reaction was stopped and silica gel chromatography (0–15% EtOAc/hexanes) was performed to obtain the title compound in 95% yield and 87% ee as an average of two experiments. **HPLC** (10% isopropyl alcohol in hexanes, ChiralPak AD-H, 1.0 mL/min, 230 nm detection): t_R(major) = 7.6 min and t_R(minor) = 9.3 min. **$[\alpha]_D$ =** –21.6º (c = 0.1, $CHCl_3$).

(*S*)-**1e**

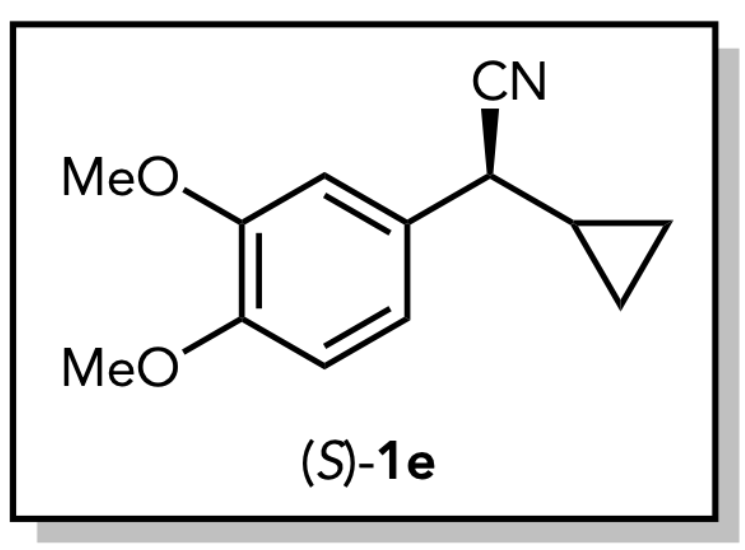

(*S*)-**1e** was prepared according to the **General Deracemization Procedure** using (*rac*)-**1e** with **Ir-CPB** (19.5 mg, 10.0 µmol, 2.0 mol%), **CPB-2** (10.5 mg, 12.5 µmol, 2.50 mol%), **CPA-1** (9.40 mg, 12.5 µmol. 2.50 mol%), and toluene (0.20 M). After 4 h of irradiation, the reaction was stopped and silica gel chromatography (0–30% EtOAc/hexanes) was performed to obtain the title compound in 98% yield and 92% ee as an average of two experiments. HPLC (8% isopropyl alcohol in hexanes, ChiralPak AD-H, 1.0 mL/min, 230 nm detection): t_R(major) = 10.2 min and t_R(minor) = 12.3 min.

$[\alpha]_D$ = –31.8º (c = 0.115, $CHCl_3$).

(*S*)-**1f**

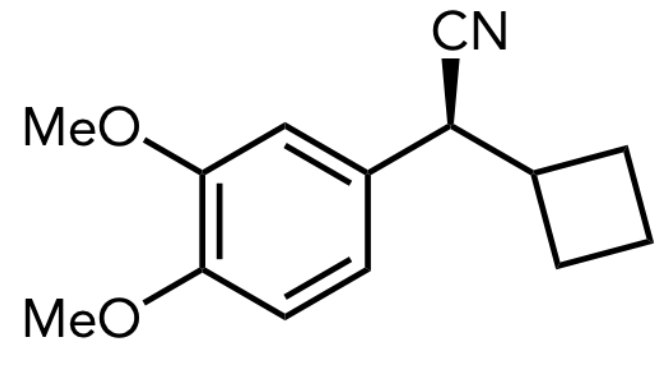

(*S*)-**1f**

(*S*)-**1f** was prepared according to the **General Deracemization Procedure** using (*rac*)-**1f** with **Ir-CPB** (19.5 mg, 10.0 µmol, 2.0 mol%), **CPB-2** (10.5 mg, 12.5 µmol, 2.50 mol%), **CPA-1** (9.40 mg, 12.5 µmol. 2.50 mol%), and toluene (0.20 M). After 4 h of irradiation, the reaction was stopped and silica gel chromatography (0–30% EtOAc/hexanes) was performed to obtain the title compound in 92% yield and 76% ee as an average of two experiments. HPLC (8% isopropyl alcohol in hexanes, ChiralPak AD-H, 1.0 mL/min, 230 nm detection): t_R(major) = 9.5 min and t_R(minor) = 10.9 min. **$[\alpha]_D$ =** –46.8º (c = 0.06, $CHCl_3$).

(*S*)-**1g**

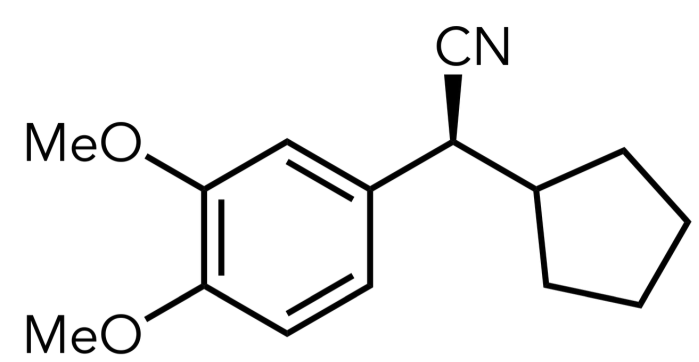

(*S*)-**1g**

(*S*)-**1g** was prepared according to the **General Deracemization Procedure** using (*rac*)-**1g** with **Ir-CPB** (19.5 mg, 10.0 µmol, 2.0 mol%), **CPB-2** (10.5 mg, 12.5 µmol, 2.50 mol%), **CPA-1** (9.40 mg, 12.5 µmol. 2.50 mol%), and toluene (0.20 M). After 3 h of irradiation, the reaction was stopped and silica gel chromatography (0–30% EtOAc/hexanes) was performed to obtain the title compound in 97% yield and 80% ee as an average of two experiments. **HPLC** (10% isopropyl alcohol in hexanes, ChiralPak AD-H, 1.0 mL/min, 280 nm detection): t_R(major) = 8.1 min and t_R(minor) = 8.9 min. **$[\alpha]_D$ =** –43.3º (c = 0.1, $CHCl_3$).

(*S*)-**1h**

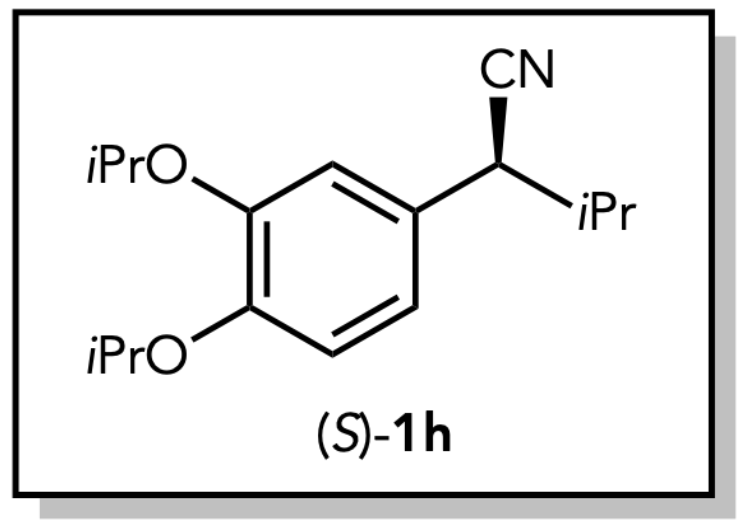

(*S*)-**1h** was prepared according to the **General Deracemization Procedure** using (*rac*)-**1h** with **Ir-CPB** (19.5 mg, 10.0 µmol, 2.0 mol%), **CPB-2** (10.5 mg, 12.5 µmol, 2.50 mol%), **CPA-1** (9.40 mg, 12.5 µmol. 2.50 mol%), and toluene (0.20 M). After 2 h of irradiation, the reaction was stopped and silica gel chromatography (0–30% EtOAc/hexanes) was performed to obtain the title compound in 99% yield and 95% ee as an average of two experiments. **HPLC** (8% isopropyl alcohol in hexanes, ChiralPak AD-H, 1.0 mL/min, 230 nm detection): t_R(major) = 7.1 min and t_R(minor) = 11.0 min. **$[\alpha]_D$ =** -23.1º (c = 0.105, $CHCl_3$).

(*S*)-**1i**

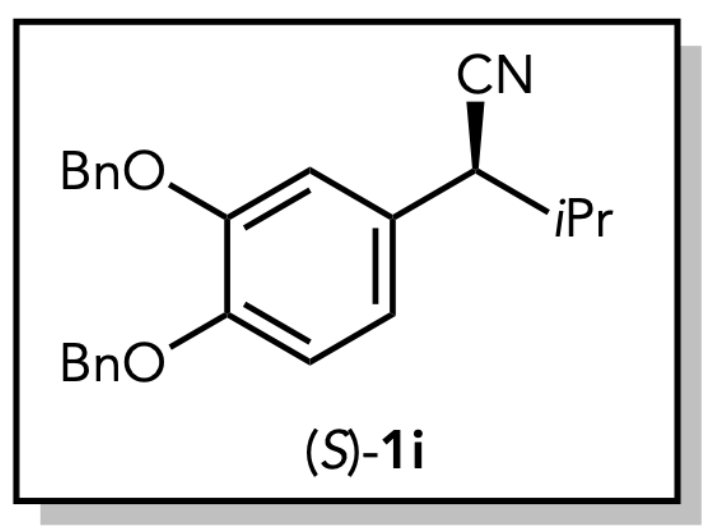

(*S*)-**1i** was prepared according to the **General Deracemization Procedure** using (*rac*)-**1i** with **Ir-CPB** (19.5 mg, 10.0 µmol, 2.0 mol%), **CPB-2** (10.5 mg, 12.5 µmol, 2.50 mol%), **CPA-1** (9.40 mg, 12.5 µmol. 2.50 mol%), and toluene (0.20 M). After 2 h of irradiation, the reaction was stopped and silica gel chromatography (0–30% EtOAc/hexanes) was performed to obtain the title compound in 88% yield and 70% ee as an average of two experiments. **HPLC** (20% isopropyl alcohol in hexanes, ChiralPak AD-H, 1.0 mL/min, 230 nm detection): t_R(major) = 8.0 and t_R(minor) = 13.0 min. **$[\alpha]_D$ =** –21.7º (c = 0.08, $CHCl_3$).

(*S*)-**1j**

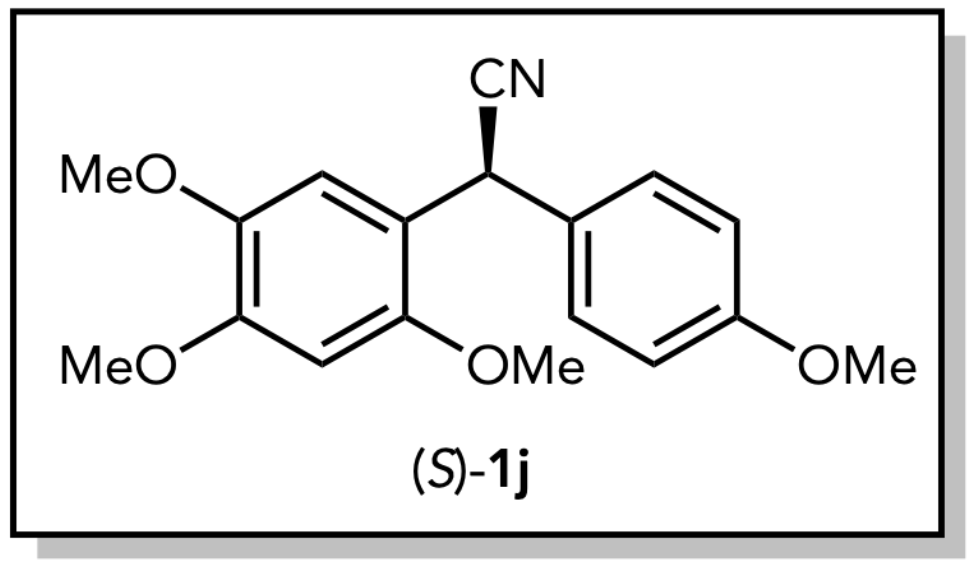

(*S*)-**1j** was prepared according to the **General Deracemization Procedure** using (*rac*)-**1j** with **Ir-CPB** (19.5 mg, 10.0 µmol, 2.0 mol%), **CPB-2** (10.5 mg, 12.5 µmol, 2.50 mol%), **CPA-1** (9.40 mg, 12.5 µmol. 2.50 mol%), and toluene (0.20 M). After 2 h of irradiation, the reaction was stopped and silica gel chromatography (0–30% EtOAc/hexanes) was performed to obtain the title compound in 92% yield and 98% ee as an average of two experiments. **HPLC** (10% isopropyl alcohol in hexanes, ChiralPak AD-H, 1.0 mL/min, 230 nm detection): t_R(major) = 14.5 min and t_R(minor) = 17.9 min. **$[\alpha]_D$ =** -53.1º (c = 0.085, $CHCl_3$).

(*S*)-**1k**

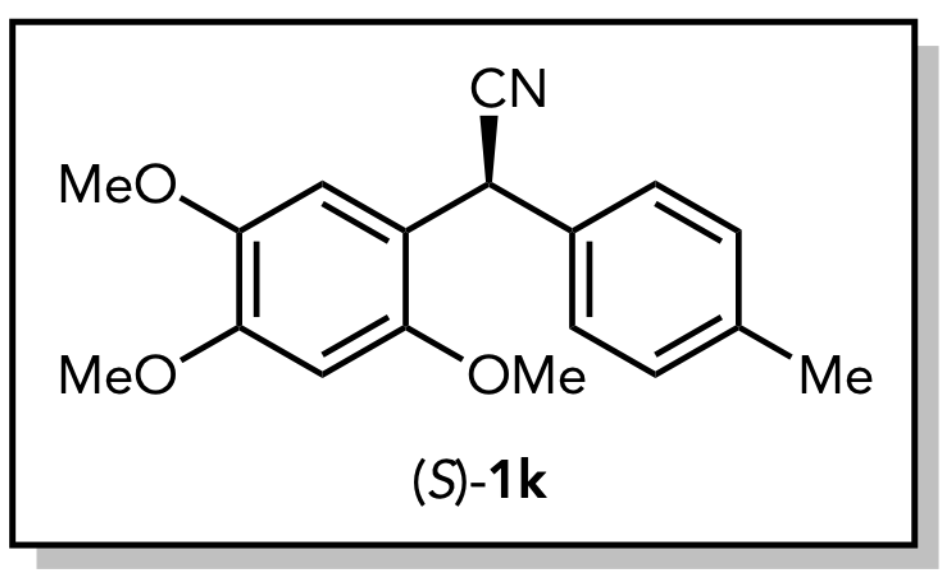

(*S*)-**1k** was prepared according to the **General Deracemization Procedure** using (*rac*)-**1k** with **Ir-CPB** (19.5 mg, 10.0 µmol, 2.0 mol%), **CPB-2** (10.5 mg, 12.5 µmol, 2.50 mol%), **CPA-1** (9.40 mg, 12.5 µmol. 2.50 mol%), and toluene (0.20 M). After 2 h of irradiation, the reaction was stopped and silica gel chromatography (0–30% EtOAc/hexanes) was performed to obtain the title compound in> 99% yield and 90% ee as an average of two experiments. **HPLC** (10% isopropyl alcohol in hexanes, ChiralPak AD-H, 1.0 mL/min, 230 nm detection): t_R(major) = 10.7 min and t_R(minor) = 12.3 min. **$[\alpha]_D$ =** -45.4º (c = 0.21, $CHCl_3$).

(*S*)-**1l**

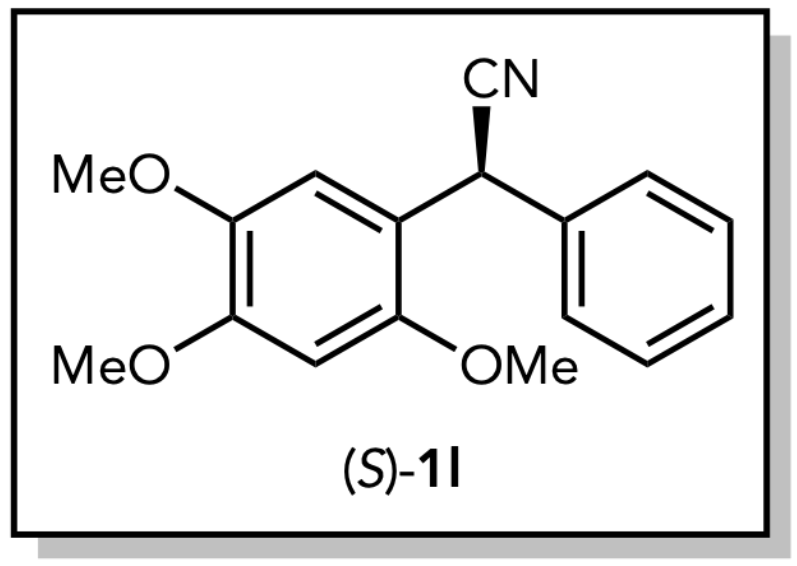

(*S*)-**1l** was prepared according to the **General Deracemization Procedure** using (*rac*)-**1l** with **Ir-CPB** (19.5 mg, 10.0 µmol, 2.0 mol%), **CPB-2** (10.5 mg, 12.5 µmol, 2.50 mol%), **CPA-1** (9.40 mg, 12.5 µmol. 2.50 mol%), and toluene (0.20 M). After 2 h of irradiation, the reaction was stopped and silica gel chromatography (0–30% EtOAc/hexanes) was performed to obtain the title compound in 97% yield and 92% ee as an average of two experiments. **HPLC** (10% isopropyl alcohol in hexanes, ChiralPak AD-H, 1.0 mL/min, 230 nm detection): t_R(major) = 10.0 min and t_R(minor) = 11.8 min. **$[\alpha]_D$ =** -62.8º (c = 0.10, $CHCl_3$).

(*S*)-**1m**

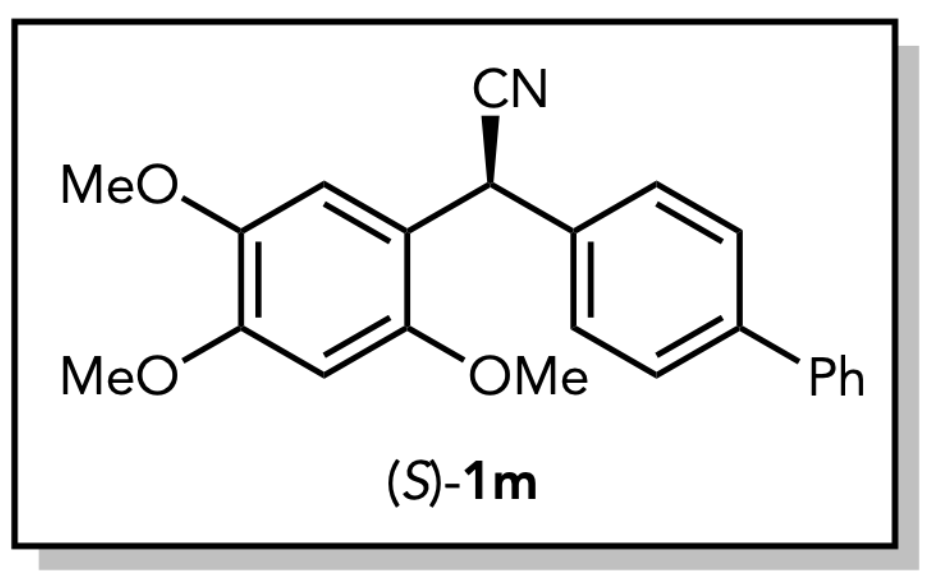

(*S*)-**1m** was prepared according to the **General Deracemization Procedure** using (*rac*)-**1m** with **Ir-CPB** (19.5 mg, 10.0 µmol, 2.0 mol%), **CPB-2** (10.5 mg, 12.5 µmol, 2.50 mol%), **CPA-1** (9.40 mg, 12.5 µmol. 2.50 mol%), and toluene (0.20 M). After 2 h of irradiation, the reaction was stopped and silica gel chromatography (0–30% EtOAc/hexanes) was performed to obtain the title compound in >99% yield and 95% ee as an average of two experiments. **HPLC** (20% isopropyl alcohol in hexanes, ChiralPak AD-H, 1.0 mL/min, 230 nm detection): t_R(major) = 13.12 min and t_R(minor) = 10.86 min. **$[\alpha]_D$ =** -57.0º (c = 0.115, $CHCl_3$).

(*S*)-**1n**

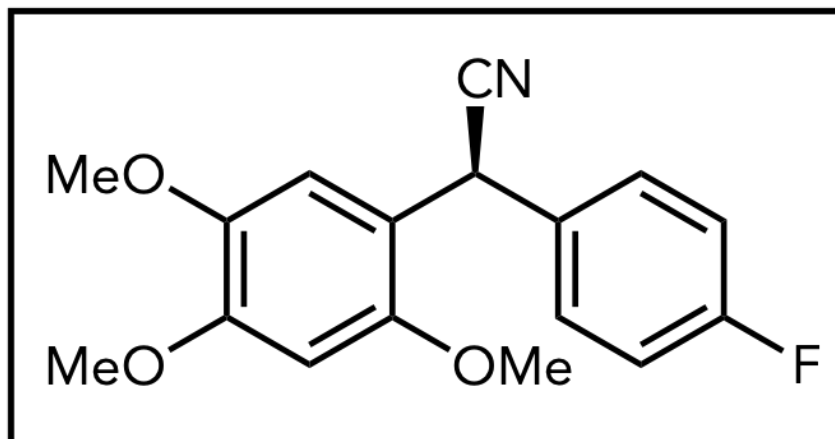

(*S*)-**1n**

(*S*)-**1n** was prepared according to the **General Deracemization Procedure** using (*rac*)-**1n** with **Ir-CPB** (19.5 mg, 10.0 µmol, 2.0 mol%), **CPB-2** (10.5 mg, 12.5 µmol, 2.50 mol%), **CPA-1** (9.40 mg, 12.5 µmol. 2.50 mol%), and toluene (0.20 M). After 2 h of irradiation, the reaction was stopped and silica gel chromatography (0–30% EtOAc/hexanes) was performed to obtain the title compound in >99% yield and 92% ee as an average of two experiments. HPLC (10% isopropyl alcohol in hexanes, ChiralPak AD-H, 1.0 mL/min, 230 nm detection): t_R(major) = 10.30 min and t_R(minor) = 12.37 min. **$[\alpha]_D$** = -52.7º (c = 0.085, $CHCl_3$).

(*S*)-**1o**

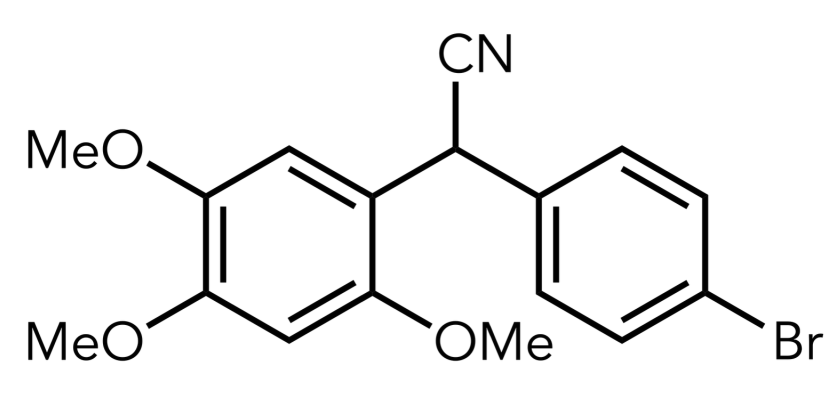

(*S*)-**1o**

(*S*)-**1o** was prepared according to the **General Deracemization Procedure** using (*rac*)-**1o** with **Ir-CPB** (19.5 mg, 10.0 µmol, 2.0 mol%), **CPB-2** (10.5 mg, 12.5 µmol, 2.50 mol%), **CPA-1** (9.40 mg, 12.5 µmol. 2.50 mol%), and toluene (0.20 M). After 2 h of irradiation, the reaction was stopped and silica gel chromatography (0–20% EtOAc/hexanes) was performed to obtain the title compound in 93% yield and 86% ee as an average of two experiments. **HPLC** (10% isopropyl alcohol in hexanes, ChiralPak AD-H, 1.0 mL/min, 280 nm detection): t_R(major) = 12.0 and t_R(minor) = 13.5 min. **$[\alpha]_D$** = –52.4º (c = 0.1, $CHCl_3$).

(*S*)-**1p**

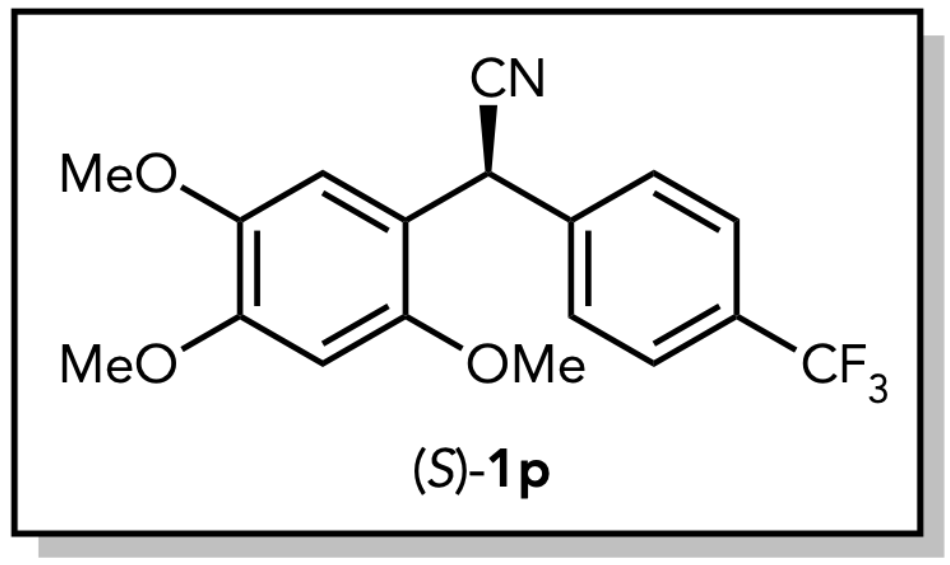

(*S*)-**1p** was prepared according to the **General Deracemization Procedure** using (*rac*)-**1p** with **Ir-CPB** (19.5 mg, 10.0 µmol, 2.0 mol%), **CPB-2** (10.5 mg, 12.5 µmol, 2.50 mol%), **CPA-1** (9.40 mg, 12.5 µmol. 2.50 mol%), and toluene (0.20 M). After 2 h of irradiation, the reaction was stopped and silica gel chromatography (0–20% EtOAc/hexanes) was performed to obtain the title compound in 97% yield and 94% ee as an average of two experiments. HPLC (5% isopropyl alcohol in hexanes, ChiralPak AD-H, 1.0 mL/min, 230 nm detection): t_R(major) = 13.6 and t_R(minor) = 14.6 min. **$[\alpha]_D$ =** –59.1º (c = 0.10, $CHCl_3$).

(*S*)-**1q**

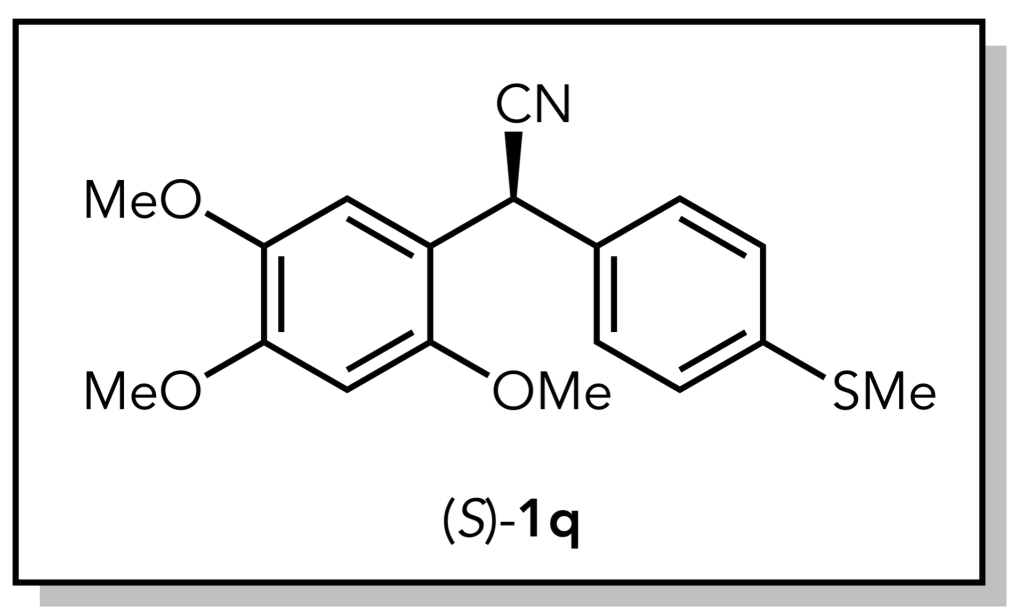

(*S*)-**1q** was prepared according to **the General Deracemization Procedure** using (*rac*)-**1q** with **Ir-CPB** (19.5 mg, 10.0 µmol, 2.0 mol%), **CPB-2** (10.5 mg, 12.5 µmol, 2.50 mol%), **CPA-1** (9.40 mg, 12.5 µmol. 2.50 mol%), and toluene (0.20 M). After 2 h of irradiation, the reaction was stopped and silica gel chromatography (0–30% EtOAc/hexanes) was performed to obtain the title compound in >99% yield and 80% ee as an average of two experiments. HPLC (15% isopropyl alcohol in hexanes, ChiralPak AD-H, 1.0 mL/min, 280 nm detection): t_R(major) = 12.1 min and t_R(minor) = 13.9 min. **$[\alpha]_D$ =** –52.4º (c = 0.10, $CHCl_3$).

(*S*)-**1r**

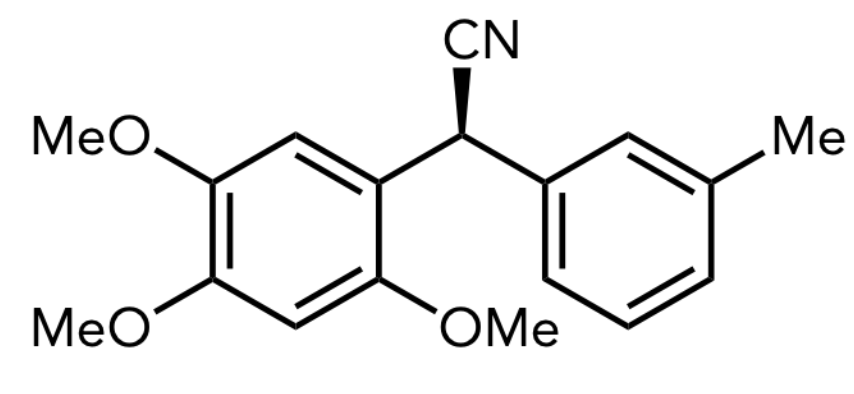

(*S*)-**1r**

(*S*)-**1r** was prepared according to the **General Deracemization Procedure** using (*rac*)-**1r** with **Ir-CPB** (19.5 mg, 10.0 µmol, 2.0 mol%), **CPB-2** (10.5 mg, 12.5 µmol, 2.50 mol%), **CPA-1** (9.40 mg, 12.5 µmol. 2.50 mol%), and toluene (0.20 M). After 2 h of irradiation, the reaction was stopped and silica gel chromatography (0–30% EtOAc/hexanes) was performed to obtain the title compound in 97% yield and 92% ee as an average of two experiments. **HPLC** (10% isopropyl alcohol in hexanes, ChiralPak AD-H, 1.0 mL/min, 230 nm detection): t_R(major) = 8.4 min and t_R(minor) = 10.1 min. **$[\alpha]_D$ =** –65.1º (c = 0.08, $CHCl_3$).

(*S*)-**1s**

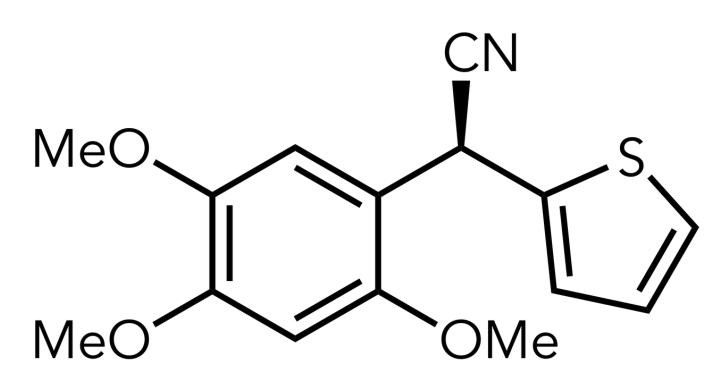

(*S*)-**1s**

(*S*)-**1s** was prepared according to the **General Deracemization Procedure** using (*rac*)-**1s** with **Ir-CPB** (19.5 mg, 10.0 µmol, 2.0 mol%), **CPB-2** (10.5 mg, 12.5 µmol, 2.50 mol%), **CPA-1** (9.40 mg, 12.5 µmol. 2.50 mol%), and toluene (0.20 M). After 8 h of irradiation, the reaction was stopped and silica gel chromatography (0–30% EtOAc/hexanes) was performed to obtain the title compound in 89% yield and 85% ee as an average of two experiments. HPLC (10% isopropyl alcohol in hexanes, ChiralPak AD-H, 1.0 mL/min, 230 nm detection): t_R(major) = 10.9 min and t_R(minor) = 13.1 min. **$[\alpha]_D$ =** –34.5º (c = 0.16, $CHCl_3$).

(*S*)-**1t**

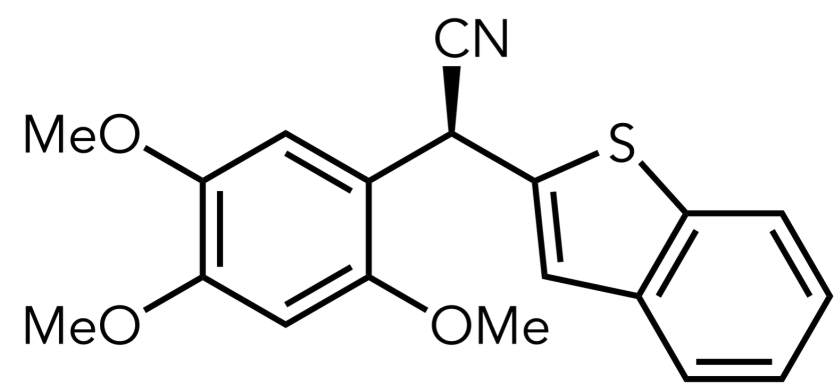

(*S*)-**1t**

(*S*)-**1t** was prepared according to the **General Deracemization Procedure** using (*rac*)-**1t** with **Ir-CPB** (19.5 mg, 10.0 µmol, 2.0 mol%), **CPB-2** (10.5 mg, 12.5 µmol, 2.50 mol%), **CPA-1** (9.40 mg, 12.5 µmol. 2.50 mol%), and toluene (0.20 M). After 2 h of irradiation, the reaction was stopped and silica gel chromatography (0–30% EtOAc/hexanes) was performed to obtain the title compound in 98% yield and 84% ee as an average of two experiments. **HPLC** (20% isopropyl alcohol in hexanes, ChiralCel AS-H, 1.0 mL/min, 280 nm detection): t_R(major) = 10.2 min and t_R(minor) = 23.2 min. **$[\alpha]_D$** = –69.6º (c = 0.10, $CHCl_3$).

(*S*)-**1u**

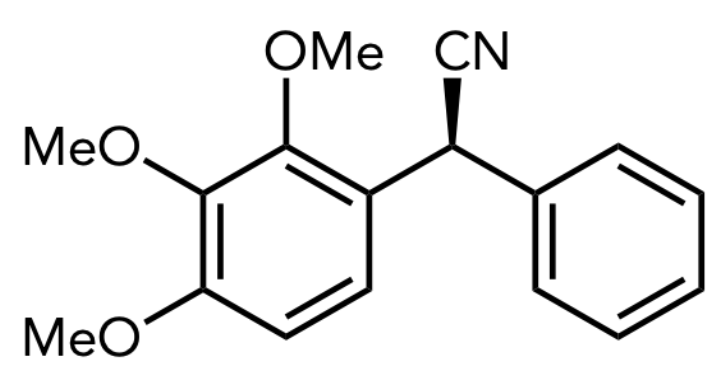

(*S*)-**1u**

(*S*)-**1q** was prepared according to the **General Deracemization Procedure** using (*rac*)-**1q** with **Ir-CPB** (19.5 mg, 10.0 µmol, 2.0 mol%), **CPB-2** (10.5 mg, 12.5 µmol, 2.50 mol%), **CPA-1** (9.40 mg, 12.5 µmol. 2.50 mol%), and toluene (0.20 M). After 2 h of irradiation, the reaction was stopped and silica gel chromatography (0–30% EtOAc/hexanes) was performed to obtain the title compound in 92% yield and 72% ee as an average of two experiments. **HPLC** (10% isopropyl alcohol in hexanes, ChiralPak OD-H, 1.0 mL/min, 230 nm detection): t_R(major) = 7.4 and t_R(minor) = 8.7 min. **$[\alpha]_D$** = –25.9º (c = 0.135, $CHCl_3$).

(*S*)-**1v**

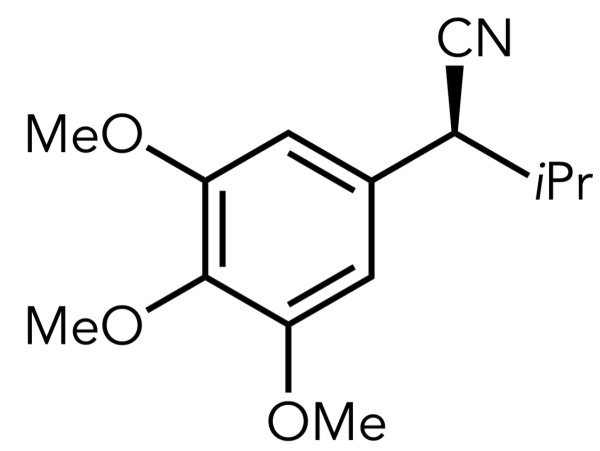

(*S*)-**1v**

(*S*)-**1v** was prepared according to the General Deracemization Procedure using (*rac*)-**1v** with **Ir-CPB** (19.5 mg, 10.0 µmol, 2.0 mol%), **CPB-2** (10.5 mg, 12.5 µmol, 2.50 mol%), **CPA-1** (9.40 mg, 12.5 µmol. 2.50 mol%), and toluene (0.20 M). After 2 h of irradiation, silica gel chromatography (0–15% EtOAc/hexanes) was performed to obtain the title compound in 96% yield and 97% ee as an average of two experiments. HPLC (10% isopropyl alcohol in hexanes, ChiralCel AS-H, 1.0 mL/min, 230 nm detection): t_R(major) = 8.7 and t_R(minor) = 16.0 min. **[α]$_D$ =** –26.6º (c = 0.10, $CHCl_3$).

(*S*)-**1w**

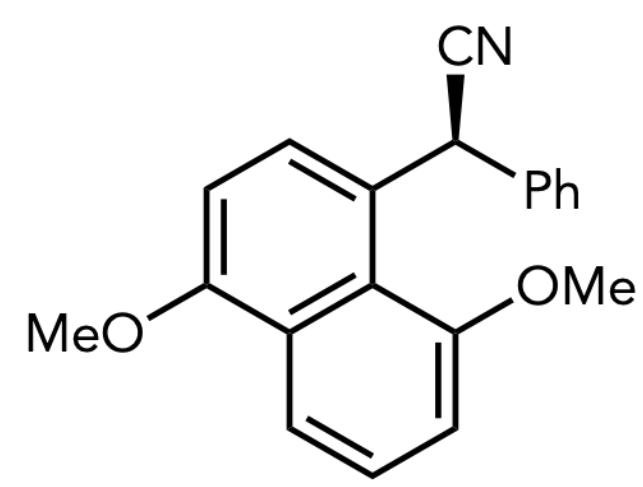

(*S*)-**1w**

(*S*)-**1w** was prepared according to the General Deracemization Procedure using (*rac*)-**1w** with **Ir-CPB** (19.5 mg, 10.0 µmol, 2.0 mol%), **CPB-2** (10.5 mg, 12.5 µmol, 2.50 mol%), **CPA-1** (9.40 mg, 12.5 µmol. 2.50 mol%), and toluene (3.5 mL, 0.14 M). After 3 h of irradiation, silica gel chromatography (0–30% EtOAc/hexanes) was performed to obtain the title compound in 91% yield and 86% ee as an average of two experiments. **HPLC** (10% isopropyl alcohol in hexanes, ChiralCel AS-H, 1.0 mL/min, 230 nm detection): t_R(major) = 24.2 and t_R(minor) = 16.7 min**. [α]$_D$ =** –58.9º (c = 0.085, $CHCl_3$).

(*S*)-**1x**

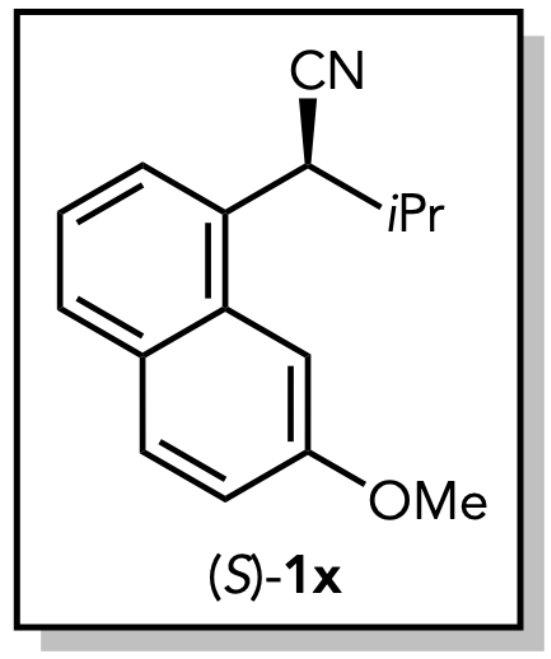

(*S*)-**1x** was prepared according to the General Deracemization Procedure using (*rac*)-**1x** with **Ir-CPB** (19.5 mg, 10.0 µmol, 2.0 mol%), **CPB-2** (10.5 mg, 12.5 µmol, 2.50 mol%), **CPA-1** (9.40 mg, 12.5 µmol. 2.50 mol%), and toluene (0.20 M). After 3 h of irradiation, the reaction was stopped and silica gel chromatography (0–30% EtOAc/hexanes) was performed to obtain the title compound in 99% yield and 80% ee as an average of two experiments. **HPLC** (5% isopropyl alcohol in hexanes, ChiralPak AD-H, 1.0 mL/min, 230 nm detection): t_R(major) = 11.3 and t_R(minor) = 10.7 min. **$[\alpha]_D$** = –55.5º (c = 0.09, $CHCl_3$).

(*S*)-**1y**

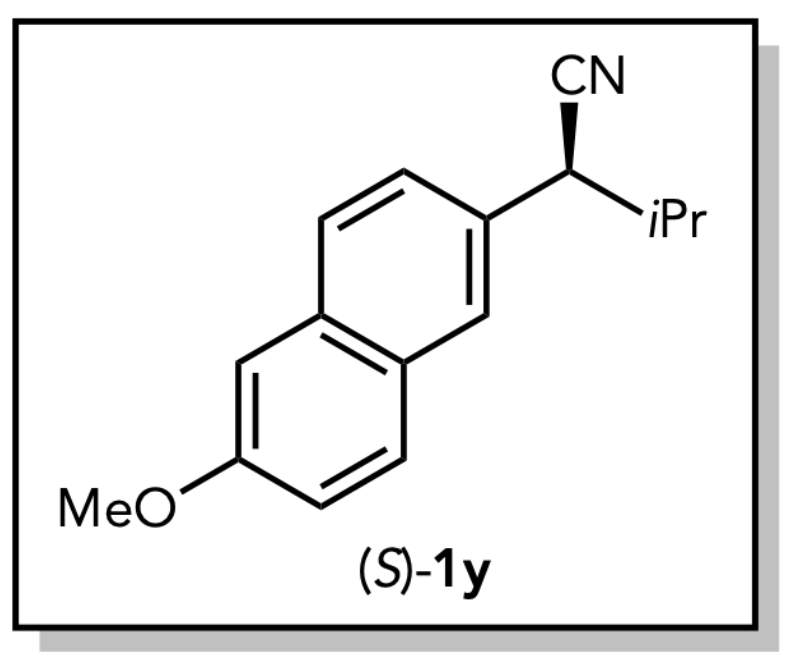

(*S*)-**1y** was prepared according to the General Deracemization Procedure using (*rac*)-**1y** with **Ir-CPB** (19.5 mg, 10.0 µmol, 2.0 mol%), **CPB-2** (10.5 mg, 12.5 µmol, 2.50 mol%), **CPA-1** (9.40 mg, 12.5 µmol. 2.50 mol%), and toluene (0.20 M). After 8 h of irradiation, the reaction was stopped and silica gel chromatography (0–30% EtOAc/hexanes) was performed to obtain the title compound in 99% yield and 80% ee as an average of two experiments. **HPLC** (10% isopropyl alcohol in hexanes, ChiralPak OJ-H, 1.0 mL/min, 230 nm detection): t_R(major) = 16.4 and t_R(minor) = 23.3 min. **$[\alpha]_D$** = –37.6º (c = 0.11, $CHCl_3$).

(S)-**1z**

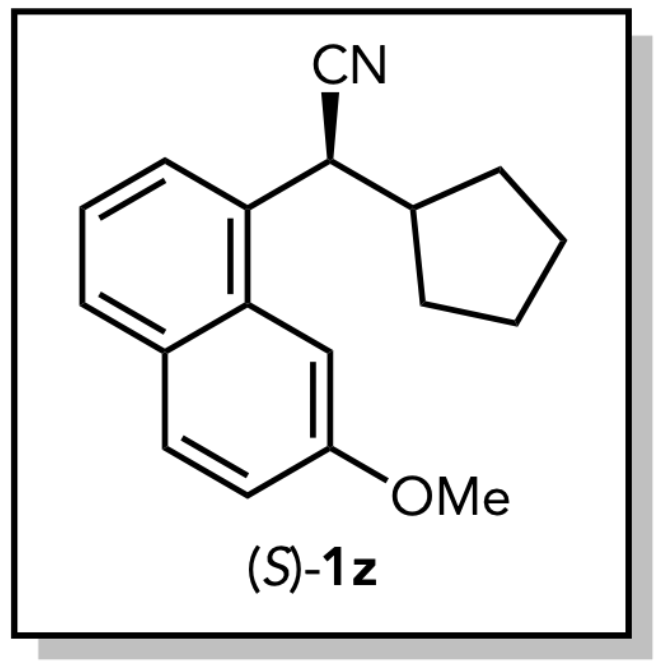

(*S*)-**1z** was prepared according to the **General Deracemization Procedure** using (*rac*)-**1z** with **Ir-CPB** (19.5 mg, 10.0 µmol, 2.0 mol%), **CPB-2** (10.5 mg, 12.5 µmol, 2.50 mol%), **CPA-1** (9.40 mg, 12.5 µmol. 2.50 mol%), and toluene (0.20 M). After 7 h of irradiation, the reaction was stopped and silica gel chromatography (0–30% EtOAc/hexanes) was performed to obtain the title compound in 98% yield and 80% ee as an average of two experiments. HPLC (10% isopropyl alcohol in hexanes, ChiralPak AD-H, 1.0 mL/min, 230 nm detection): t_R(major) = 9.2 and t_R(minor) = 8.4 min. **$[\alpha]_D$ =** –78.6º (c = 0.135, $CHCl_3$).

(*S*)-**1aa**

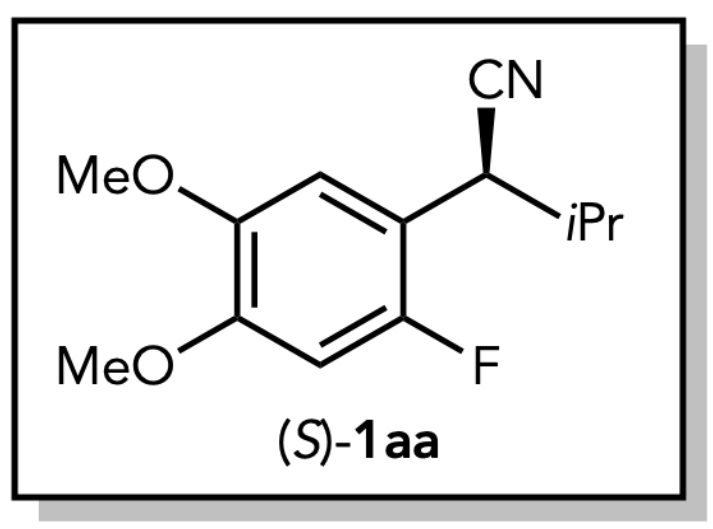

(*S*)-**1aa** was prepared according to the **General Deracemization Procedure** using (*rac*)-**1aa** with **Ir-CPB** (19.5 mg, 10.0 µmol, 2.0 mol%), **CPB-2** (10.5 mg, 12.5 µmol, 2.50 mol%), **CPA-1** (9.40 mg, 12.5 µmol. 2.50 mol%), and toluene (0.20 M). After 2 h of irradiation, the reaction was stopped and silica gel chromatography (0–30% EtOAc/hexanes) was performed to obtain the title compound in 78% yield and 90% ee as an average of two experiments. **HPLC** (8% isopropyl alcohol in hexanes, ChiralPak AD-H, 1.0 mL/min, 230 nm detection): t_R(major) = 6.5 and t_R(minor) = 8.3 min. **$[\alpha]_D$ =** –32.8º (c = 0.13, $CHCl_3$).

(*S*)-**1ab**

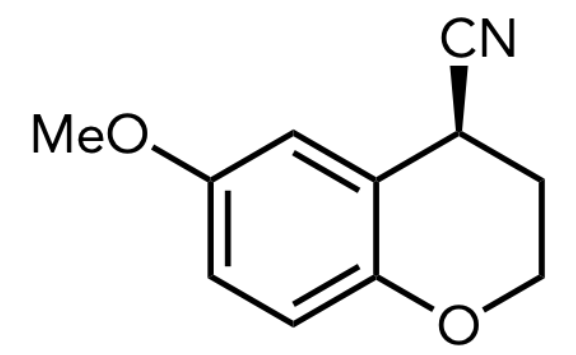

(*S*)-**1ab**

(*S*)-**1ab** was prepared according to the **General Deracemization Procedure** using (*rac*)-**1ab** with **Ir-CPB** (19.5 mg, 10.0 µmol, 2.0 mol%), **CPB-2** (10.5 mg, 12.5 µmol, 2.50 mol%), **CPA-1** (9.40 mg, 12.5 µmol. 2.50 mol%), and toluene (0.20 M). After 4 h of irradiation, the reaction was stopped and silica gel chromatography (0–30% EtOAc/hexanes) was performed to obtain the title compound in 92% yield and 90% ee as an average of two experiments. **HPLC** (5% isopropyl alcohol in hexanes, ChiralPak OD-H, 1.0 mL/min, 230 nm detection): t_R(major) = 14.3 and t_R(minor) = 23.9 min. **[α]$_D$ =** –45.8º (c = 0.195, $CHCl_3$)

(*S*)-**1ac**

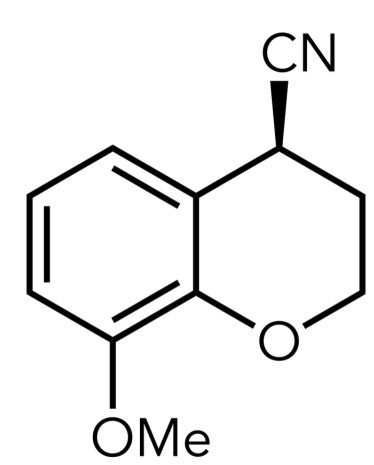

(*S*)-**1ac**

(*S*)-**1ac** was prepared according to the **General Deracemization Procedure** using (*rac*)-**1ac** with **Ir-CPB** (19.5 mg, 10.0 µmol, 2.0 mol%), **CPB-2** (10.5 mg, 12.5 µmol, 2.50 mol%), **CPA-1** (9.40 mg, 12.5 µmol. 2.50 mol%), and toluene (0.20 M). After 2 h of irradiation, the reaction was stopped and silica gel chromatography (0–30% EtOAc/hexanes) was performed to obtain the title compound in 99% yield and 80% ee as an average of two experiments. HPLC (10% isopropyl alcohol in hexanes, OZ-H, 1.0 mL/min, 280 nm detection): t_R(major) = 13.4 and t_R(minor) = 16.5 min. **[α]$_D$ =** -5.5º (c = 0.16, $CHCl_3$)

(2*S*)-**1ad** (2*S*, 3*S*)-2-(3,4-dimethoxyphenyl)-3-methylpentanenitrile

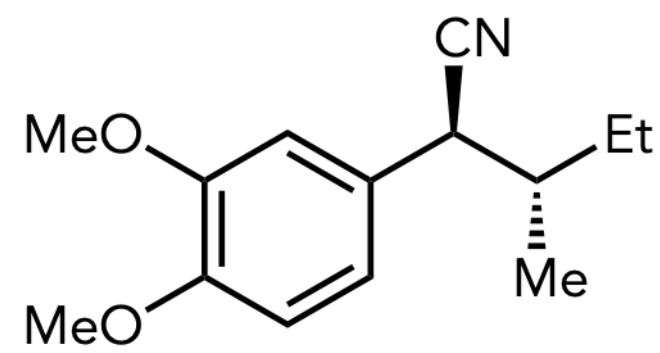

(2*S*)-**1ad**

(2*S*)-**1ad** was prepared according to the **General Deracemization Procedure** using (*rac*)-**1ad** with **Ir-CPB** (19.5 mg, 10.0 µmol, 2.0 mol%), **CPB-2** (10.5 mg, 12.5 µmol, 2.50 mol%), **CPA-1** (9.40 mg, 12.5 µmol. 2.50 mol%), and toluene (0.20 M). After 2 h of irradiation, the reaction was stopped and silica gel chromatography (0–15% EtOAc/hexanes) was performed to obtain the title compound in 98% yield and 97:3 dr as an average of two experiments. (Reported as a mixture of diastereomers) **^{1}H NMR** (500 MHz, $CDCl_3$) δ 6.84 (d, *J* = 1.9 Hz, 2H), 6.79 (d, *J* = 1.6 Hz, 1H), 3.90 (s, 3H), 3.88 (s, 3H), 3.78 (d, *J* = 5.7 Hz, 0.03H), 3.62 (d, *J* = 6.7 Hz, 0.97H), 1.88 (dpd, *J* = 10.4, 6.7, 3.7 Hz, 1H), 1.63 (dqd, *J* = 14.9, 7.5, 3.7 Hz, 1H), 1.40 – 1.22 (m, 1H), 0.98 (d, *J* = 6.8 Hz, 3H), 0.92 (t, *J* = 7.4 Hz, 3H). **^{13}C NMR** (126 MHz, $CDCl_3$) δ 149.30, 148.89, 127.26, 120.63, 111.30, 111.11, 56.15, 56.09, 43.76, 40.05, 26.02, 17.01, 11.44. HPLC (5% isopropyl alcohol in hexanes, ChiralCel AS-H, 1.0 mL/min, 280 nm detection): t_R(major) = 13.1 and t_R(minor) = 18.3 min. **[α]$_D$ =** –27.5° (c = 0.10, $CHCl_3$).

(2*R*)-**1ad** (2*R*, 3*S*)-2-(3,4-dimethoxyphenyl)-3-methylpentanenitrile

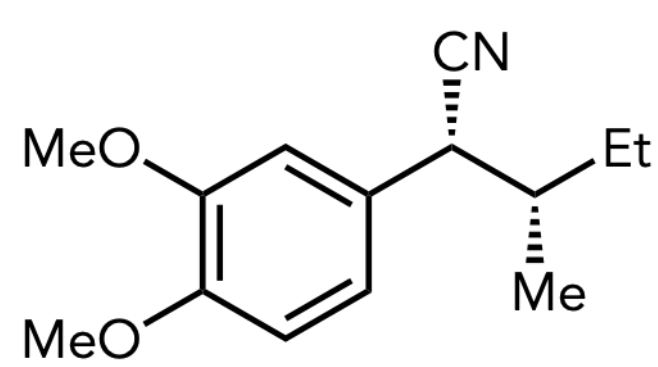

(2*R*)-**1ad**

(2*R*)-**1ad** was prepared according to the **General Deracemization Procedure** using (*rac*)-**1ad** with (*R*)-**Ir-CPB** (19.5 mg, 10.0 µmol, 2.0 mol%), (*R*)-**CPB-2** (10.5 mg, 12.5 µmol, 2.50 mol%), (*R*)-**CPA-1** (9.40 mg, 12.5 µmol. 2.50 mol%), and toluene (0.20 M). After 2 h of irradiation, the reaction was stopped and silica gel chromatography (0–15% EtOAc/hexanes) was performed to obtain the title compound in 88% yield and 91:9 dr as an average of two experiments. (Reported as a mixture of diastereomers) **^{1}H NMR** (500 MHz, $CDCl_3$) δ 6.88 (d, *J* = 1.3 Hz, 2H), 6.81 (d, *J* = 1.4 Hz, 1H), 3.92 (s, 3H), 3.91 (s, 3H), 3.80 (d, *J* = 5.7 Hz, 1H), 3.64 (d, *J* = 6.8 Hz, 0.09H), 1.97 – 1.78 (m, 0.91H), 1.57 (hd, *J* = 7.4, 5.6 Hz, 2H), 1.46 – 1.26 (m, 1H), 1.06 – 0.90 (m, 6H). **^{13}C NMR** (126 MHz, $CDCl_3$) δ 149.30, 148.80, 127.59, 127.26, 120.63, 120.29, 119.94, 111.35, 111.30, 111.10, 110.90, 56.15, 56.10, 43.75, 43.04, 40.51, 40.04, 27.84, 26.01, 17.00, 15.68, 11.68,

11.44. **HPLC** (5% isopropyl alcohol in hexanes, ChiralCel AS-H, 1.0 mL/min, 280 nm detection): t_R(major) = 17.5 min and t_R(minor) = 12.6 min. **[α]$_D$ =** 21.2º (c = 0.10, $CHCl_3$).

(*S*)-**1ae**

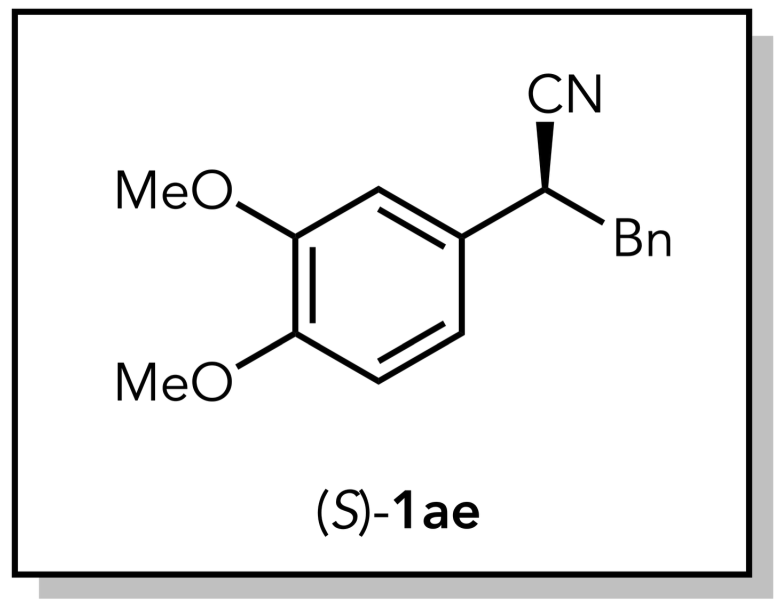

(*S*)-**1ae** was prepared according to the **General Deracemization Procedure** using (*rac*)-**1ae** with **Ir-CPB** (19.5 mg, 10.0 µmol, 2.0 mol%), **CPB-2** (10.5 mg, 12.5 µmol, 2.50 mol%), **CPA-1** (9.40 mg, 12.5 µmol. 2.50 mol%), and toluene (0.20 M). After 2 h of irradiation, the reaction was stopped and silica gel chromatography (0–30% EtOAc/hexanes) was performed to obtain the title compound in 87% yield and 40% ee as an average of two experiments. **HPLC** (5% isopropyl alcohol in hexanes, ChiralCel AS-H, 1.0 mL/min, 230 nm detection): t_R(major) = 9.7 min and t_R(minor) = 11.0 min. **[α]$_D$ =** 12.9º (c = 0.10, $CHCl_3$).

(*S*)-1ae-*d*$_1$ 2-(3,4-dimethoxyphenyl)-3-phenylpropanenitrile-2-*d*

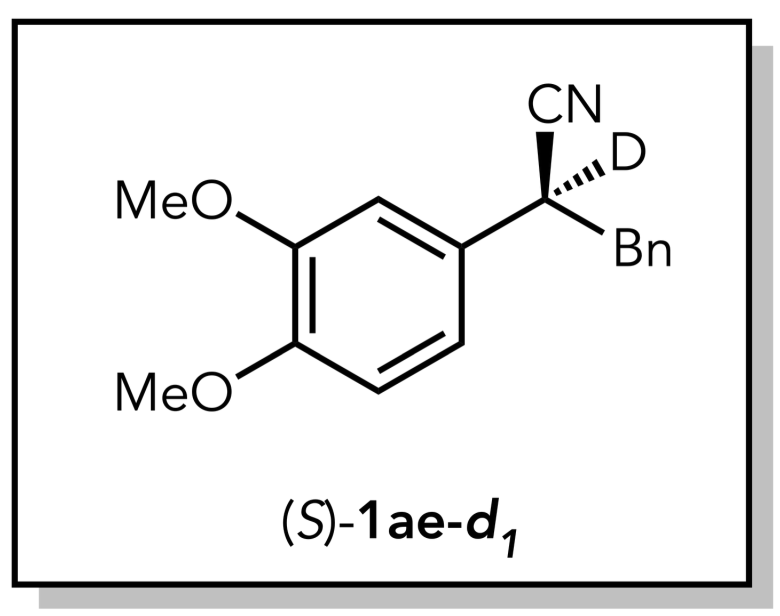

(*S*)-**1ae-*d*$_1$** was prepared according to the **General Deracemization Procedure** using (*rac*)-**1ae** with **Ir-CPB** (19.5 mg, 10.0 µmol, 2.0 mol%), **CPB-2** (10.5 mg, 12.5 µmol, 2.50 mol%), **CPA-1** (9.40 mg, 12.5 µmol. 2.50 mol%), D_2O (10 equiv), and toluene (0.20 M). After 2 h the reaction was stopped and silica gel chromatography (0–30% EtOAc/hexanes) was performed to obtain the title compound in 98% yield, 72% ee, and 99% D incorporation as an average of two experiments. **^{1}H NMR** (500 MHz, $CDCl_3$) δ 7.32 – 7.26 (m, 5H), 7.14 – 7.10 (m, 2H), 6.85 – 6.77 (m, 2H), 6.64 (d, *J* = 1.9 Hz, 1H), 3.88 (s, 3H), 3.80 (s, 3H), 3.19 (d, *J* = 13.5 Hz, 1H), 3.11 (d, *J* = 13.6 Hz, 1H). **^{13}C NMR** (126 MHz, $CDCl_3$) δ 149.28, 149.02, 136.44, 129.46, 128.75, 127.57, 127.49, 120.73, 119.92, 111.43, 110.72, 56.09, 56.06, 42.31. **HRMS**: m/z $[M+Na]^+$ calcd. For $C_{17}H_{16}DNO_2Na$: 291.121975; found: 291.12206 (0.29 ppm). **HPLC** (5% isopropyl alcohol in hexanes, ChiralCel AS-H, 1.0 mL/min, 230 nm detection): t_R(major) = 9.7 min and t_R(minor) = 11.0 min. **[α]$_D$ =** 13.3º (c = 0.10, $CHCl_3$).

(*R*)-**6**

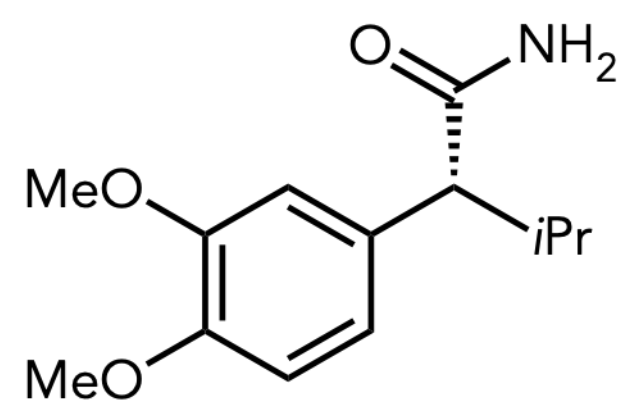

(*R*)-**6**

A 2-dram vial equipped with a magnetic stir bar was charged with (*S*)-**1a** (89% ee) (150 mg, 0.684 mmol, 1.00 equiv), [(DPPF)-Pt(PMe_2OH)Cl])Tf (5.5 mg, 5.5 µmol, 0.8 mol%), silver triflate (1.4 mg, 5.4 µmol, 0.8 mol%), water (0.5 mL), and THF (0.5 mL). The vial was stirred at 40 ºC under air for 20 h. The reaction mixture was poured into brine (30 mL) and transferred to a separatory funnel. The aqueous phase extracted with EtOAc (3 × 30 mL). The combined organic layers were dried over Na_2SO_4, filtered, and concentrated *in vacuo*. The crude residue was purified by silica gel chromatography (25–100% EtOAc/hexanes) to afford the title compound as a white solid in 92% yield and 89% ee (150 mg, 0.632 mmol). **HPLC** (15% isopropyl alcohol in hexanes, ChiralCel OD-H, 1.0 mL/min, 280 nm detection): t_R(major) = 16.9 min and t_R(minor) = 12.9 min. **$[\alpha]_D$ =** 20.0º (c = 0.20, $CHCl_3$)

5.2 HPLC Chromatograms

1a and racemic standard (10% isopropyl alcohol in hexanes, AD-H, 15 min)

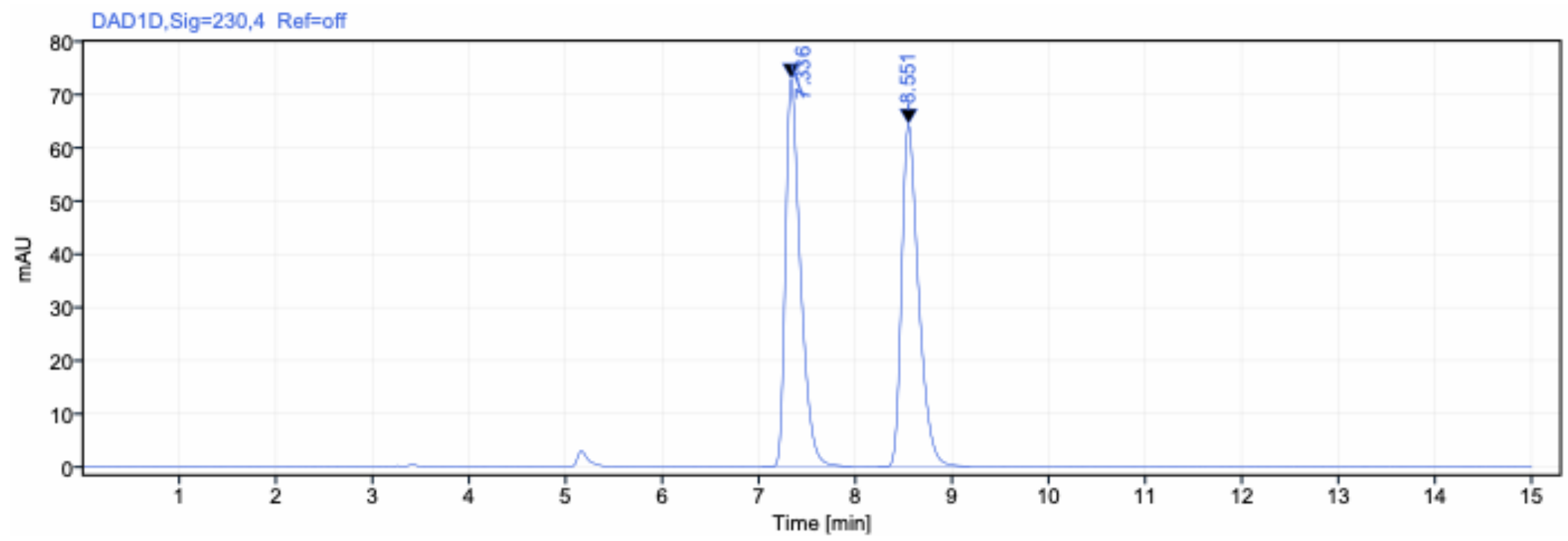

Signal: DAD1D,Sig=230,4 Ref=off

RT [min]	Type	Width [min]	Area	Height	Area%	Name
7.336	MM m	0.94	784.43	72.80	50.05	
8.551	MM m	0.96	782.90	64.27	49.95	

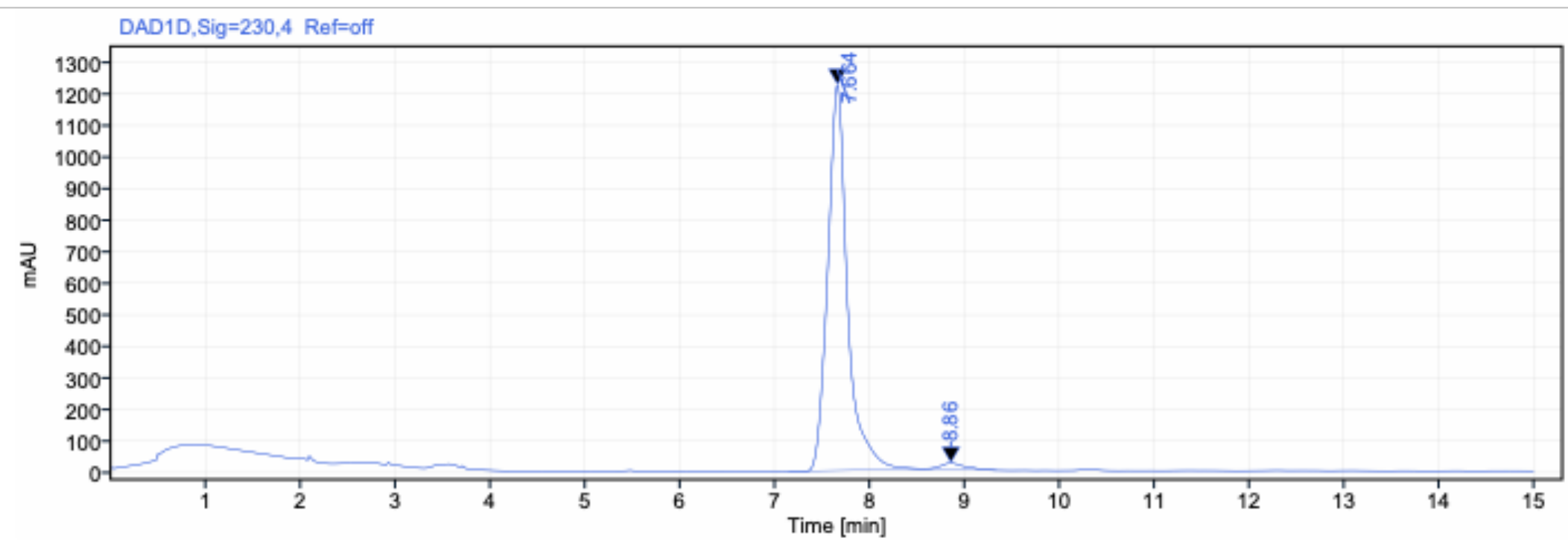

Signal: DAD1D,Sig=230,4 Ref=off

RT [min]	Type	Width [min]	Area	Height	Area%	Name
7.664	MM m	1.40	17663.00	1221.87	98.09	
8.860	MM m	0.78	344.21	19.94	1.91	
		**Sum**	**18007.21**			

1a-d_1 and racemic standard (10% isopropyl alcohol in hexanes, AD-H, 15 min)

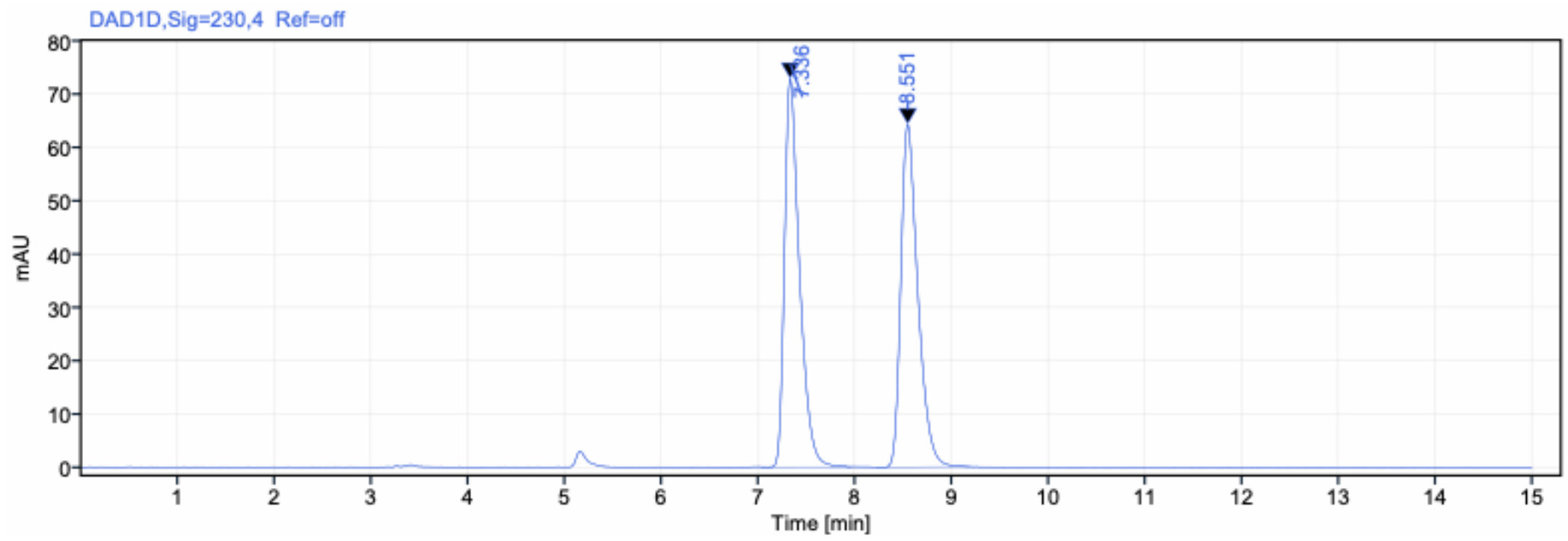

Signal: DAD1D,Sig=230,4 Ref=off

RT [min]	Type	Width [min]	Area	Height	Area%	Name
7.336	MM m	0.94	784.43	72.80	50.05	
8.551	MM m	0.96	782.90	64.27	49.95	

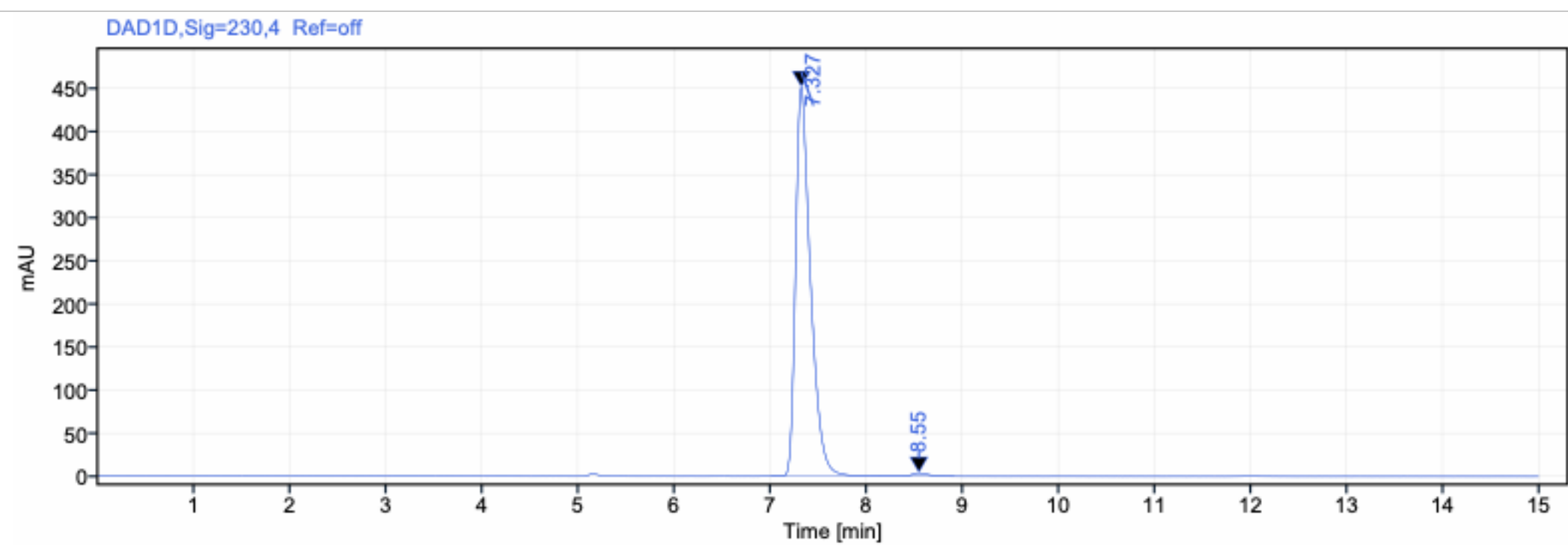

Signal: DAD1D,Sig=230,4 Ref=off

RT [min]	Type	Width [min]	Area	Height	Area%	Name
7.327	MM m	1.29	4870.50	451.30	99.05	
8.550	MM m	0.62	46.47	3.93	0.95	

1b and racemic standard (8% isopropyl alcohol in hexanes, AD-H, 20 min)

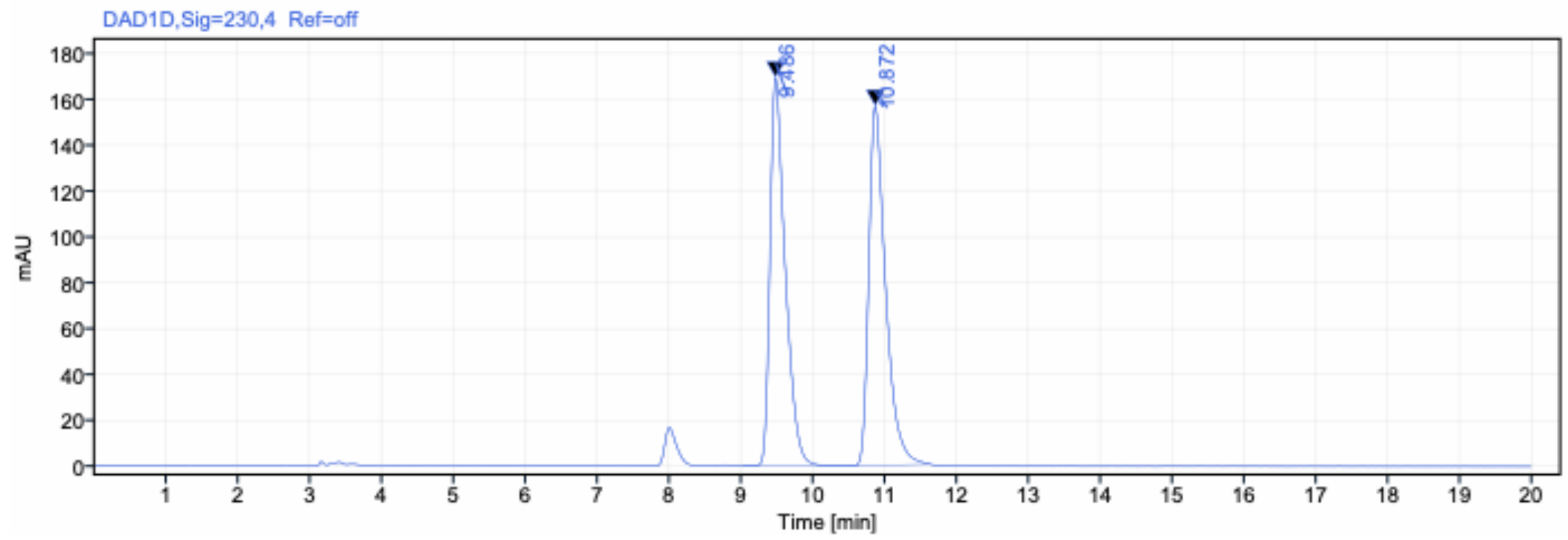

Signal: DAD1D,Sig=230,4 Ref=off

RT [min]	Type	Width [min]	Area	Height	Area%	Name
9.486	MM m	1.41	2490.24	169.42	49.54	
10.872	MM m	1.19	2536.99	156.79	50.46	

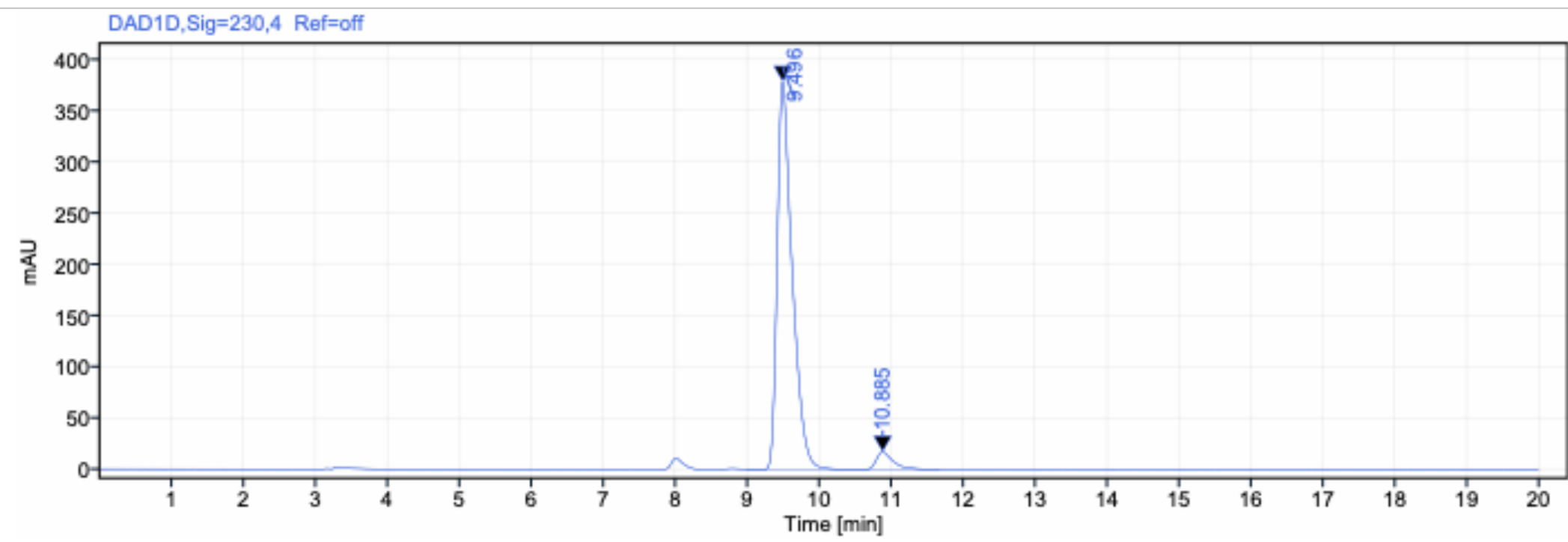

Signal: DAD1D,Sig=230,4 Ref=off

RT [min]	Type	Width [min]	Area	Height	Area%	Name
9.496	MM m	1.44	5473.41	378.30	95.07	
10.885	MM m	1.25	283.92	17.10	4.93	

1c and racemic standard (8% isopropyl alcohol in hexanes, AD-H, 20 min)

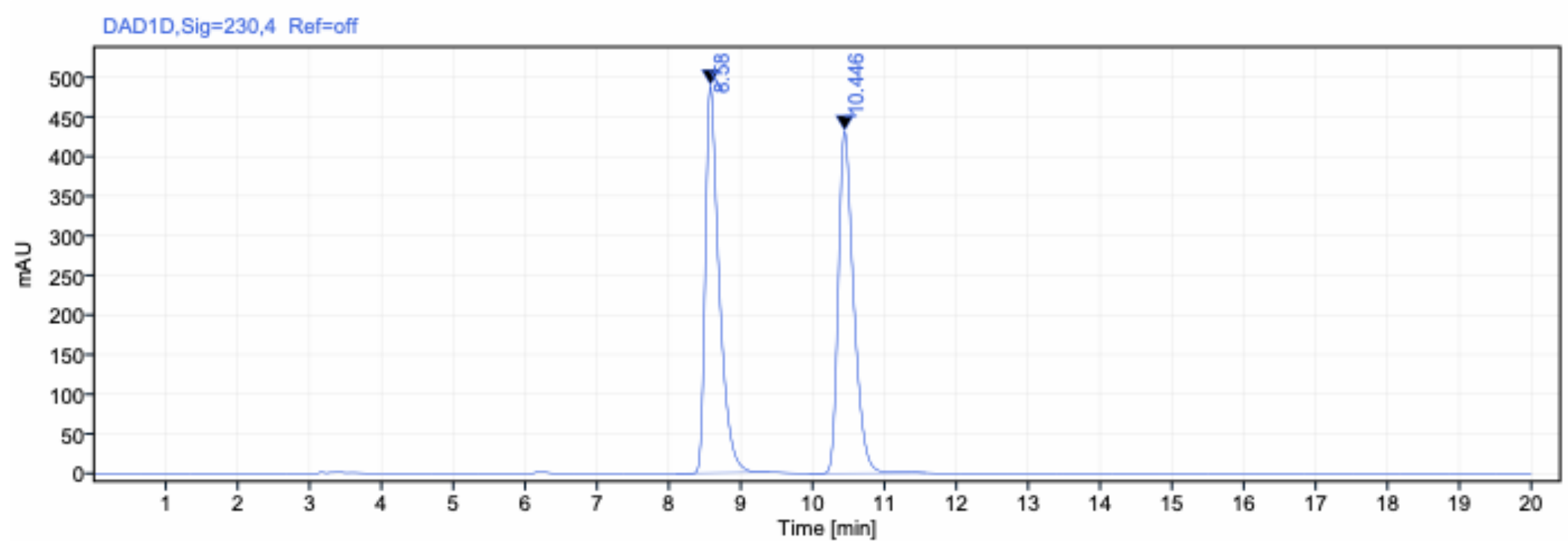

Signal: DAD1D,Sig=230,4 Ref=off

RT [min]	Type	Width [min]	Area	Height	Area%	Name
8.580	MM m	1.31	6507.24	488.76	50.43	
10.446	MM m	1.63	6396.61	431.31	49.57	

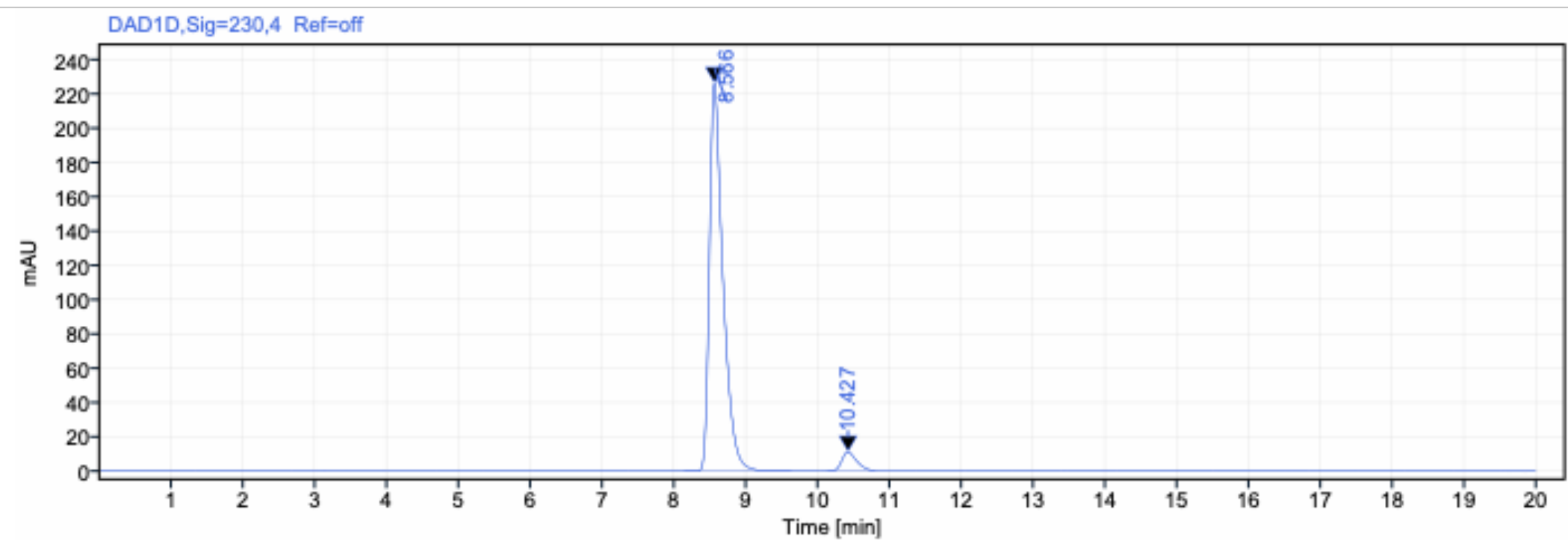

Signal: DAD1D,Sig=230,4 Ref=off

RT [min]	Type	Width [min]	Area	Height	Area%	Name
8.566	MM m	1.49	2952.54	226.20	94.89	
10.427	MM m	0.67	158.84	11.11	5.11	

1d and racemic standard (10% isopropyl alcohol in hexanes, AD-H, 15 min)

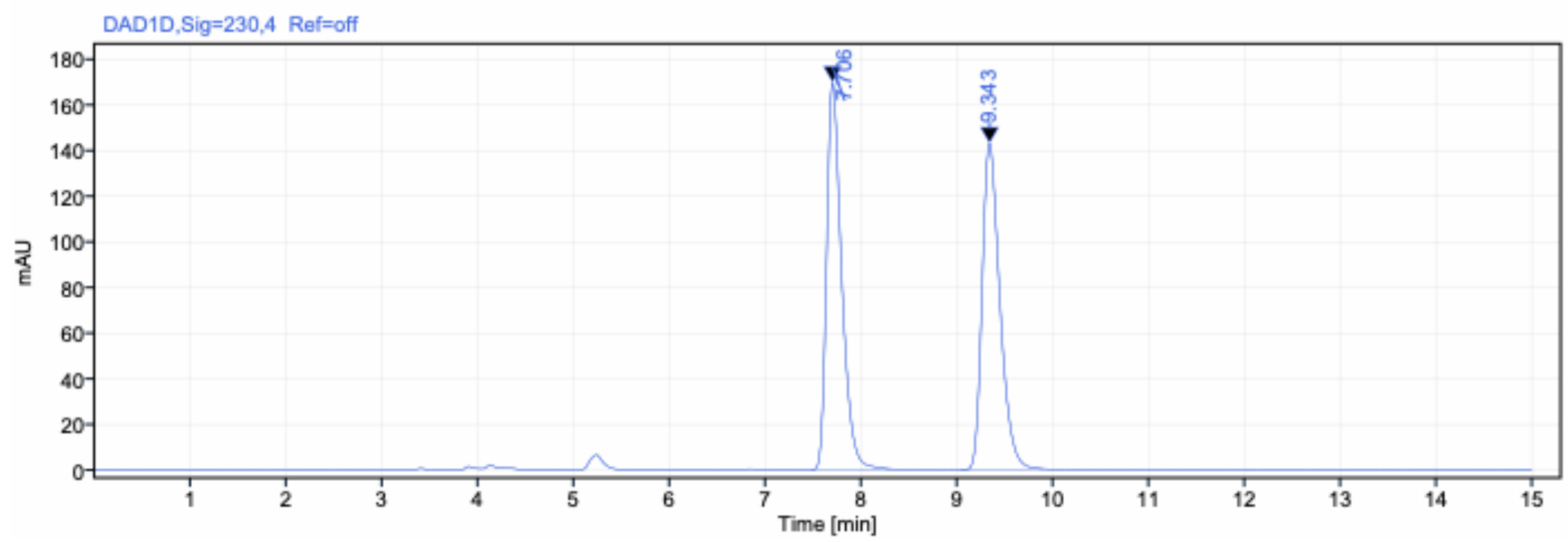

Signal: DAD1D,Sig=230,4 Ref=off

RT [min]	Type	Width [min]	Area	Height	Area%	Name
7.706	MM m	0.82	1842.59	169.61	50.08	
9.343	MM m	0.86	1836.68	142.94	49.92	

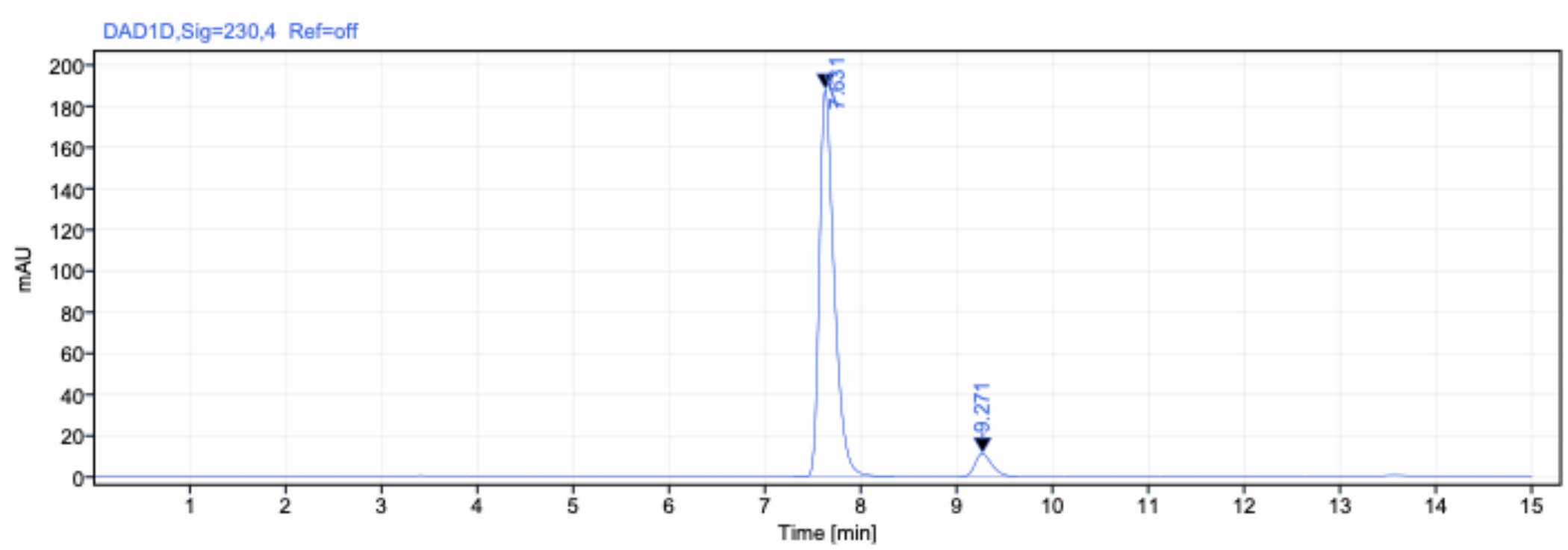

Signal: DAD1D,Sig=230,4 Ref=off

RT [min]	Type	Width [min]	Area	Height	Area%	Name
7.631	MM m	1.05	2012.69	187.88	93.64	
9.271	MM m	0.61	136.72	11.16	6.36	

1e and racemic standard (8% isopropyl alcohol in hexanes, AD-H, 20 min)

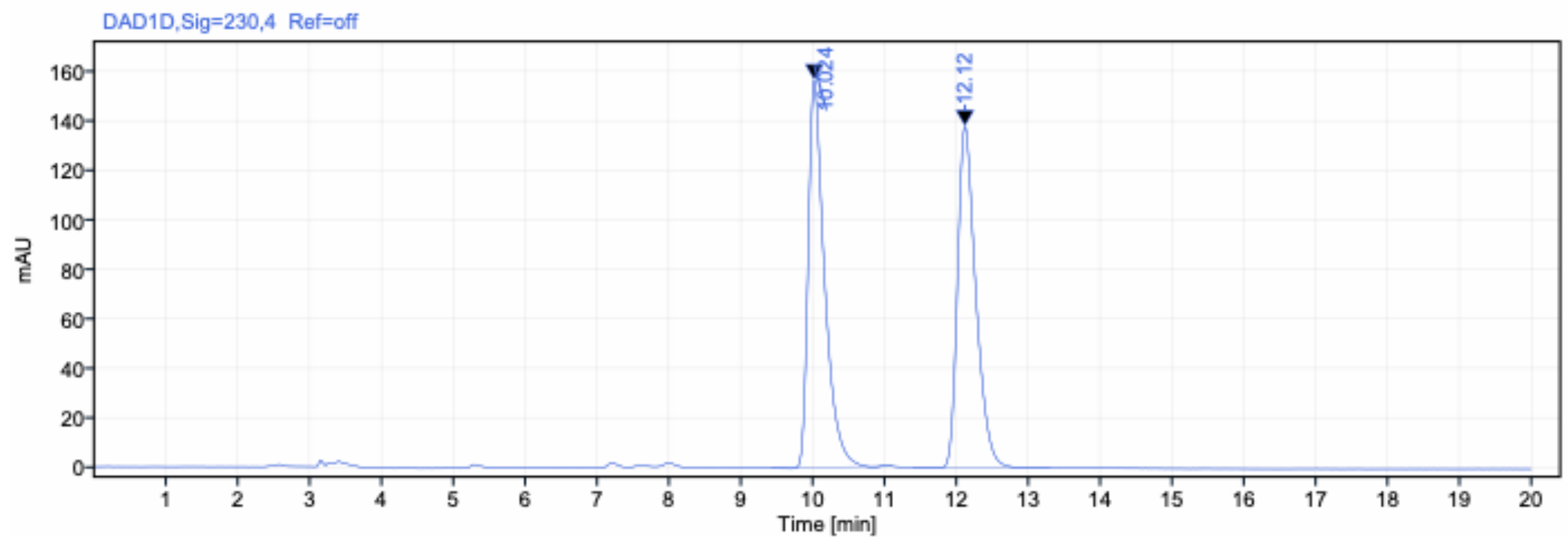

Signal: DAD1D,Sig=230,4 Ref=off

RT [min]	Type	Width [min]	Area	Height	Area%	Name
10.024	MM m	1.69	2470.00	156.41	50.37	
12.120	MM m	1.91	2434.09	138.02	49.63	

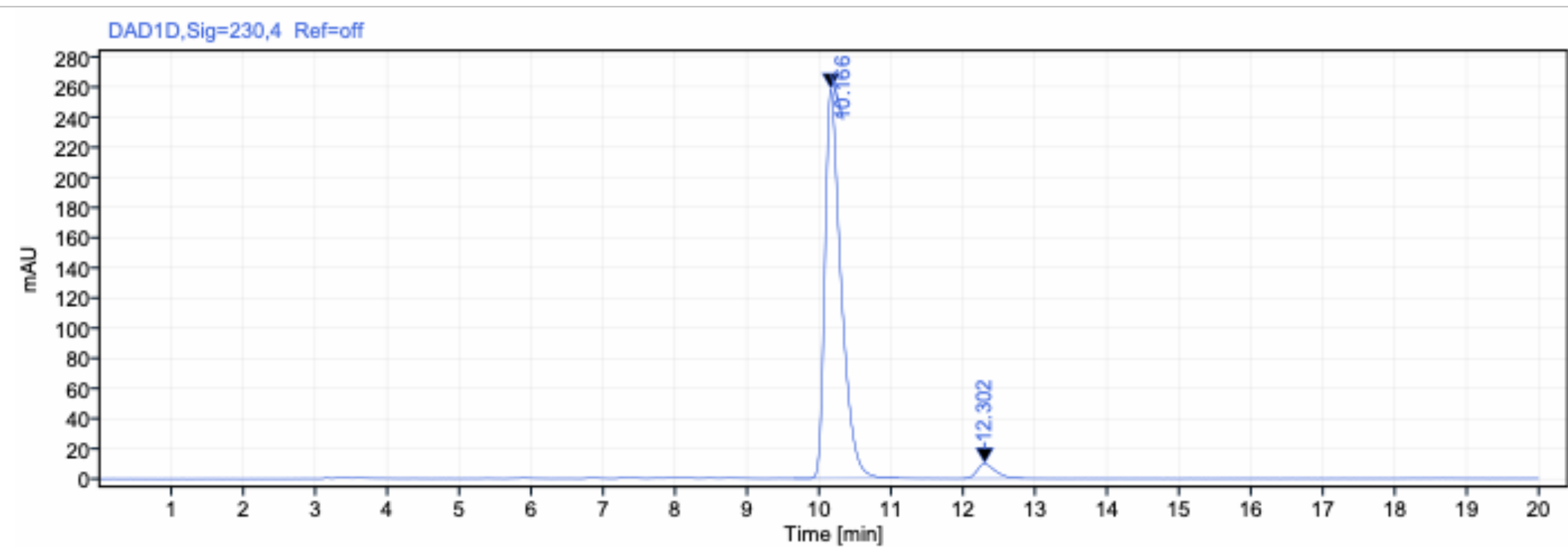

Signal: DAD1D,Sig=230,4 Ref=off

RT [min]	Type	Width [min]	Area	Height	Area%	Name
10.166	MM m	1.46	4026.75	258.16	95.95	
12.302	BB	0.83	169.90	9.70	4.05	

1f and racemic standard (8% isopropyl alcohol in hexanes, AD-H, 20 min)

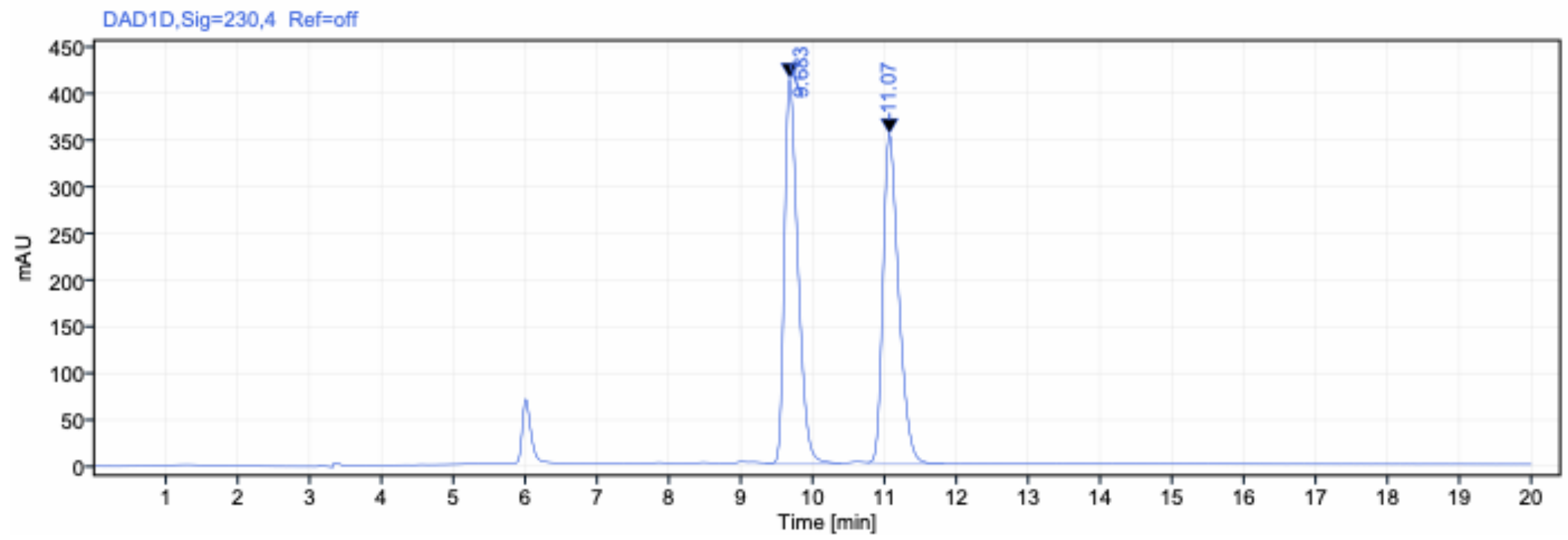

Signal: DAD1D,Sig=230,4 Ref=off

RT [min]	Type	Width [min]	Area	Height	Area%	Name
9.683	MM m	1.05	5343.42	411.46	50.09	
11.070	MM m	1.07	5325.00	352.01	49.91	

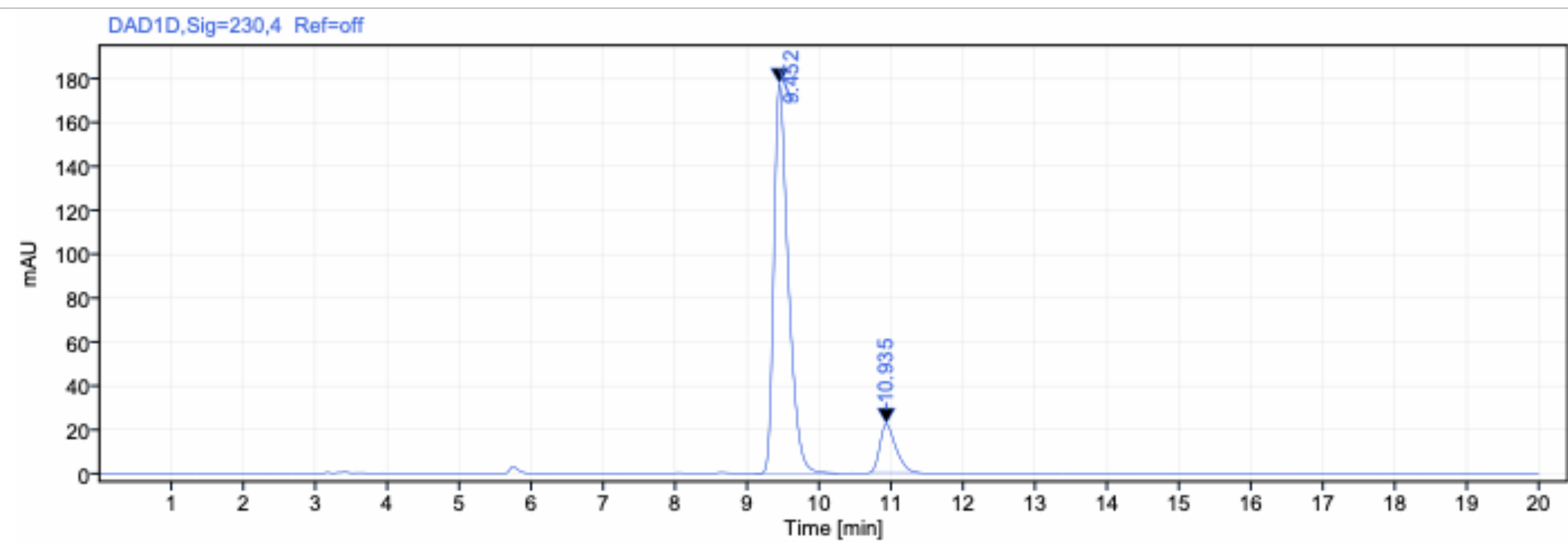

Signal: DAD1D,Sig=230,4 Ref=off

RT [min]	Type	Width [min]	Area	Height	Area%	Name
9.452	MM m	1.16	2467.32	177.26	87.85	
10.935	MM m	0.70	341.21	22.11	12.15	

1g and racemic standard (10% isopropyl alcohol in hexanes, AD-H, 15 min)

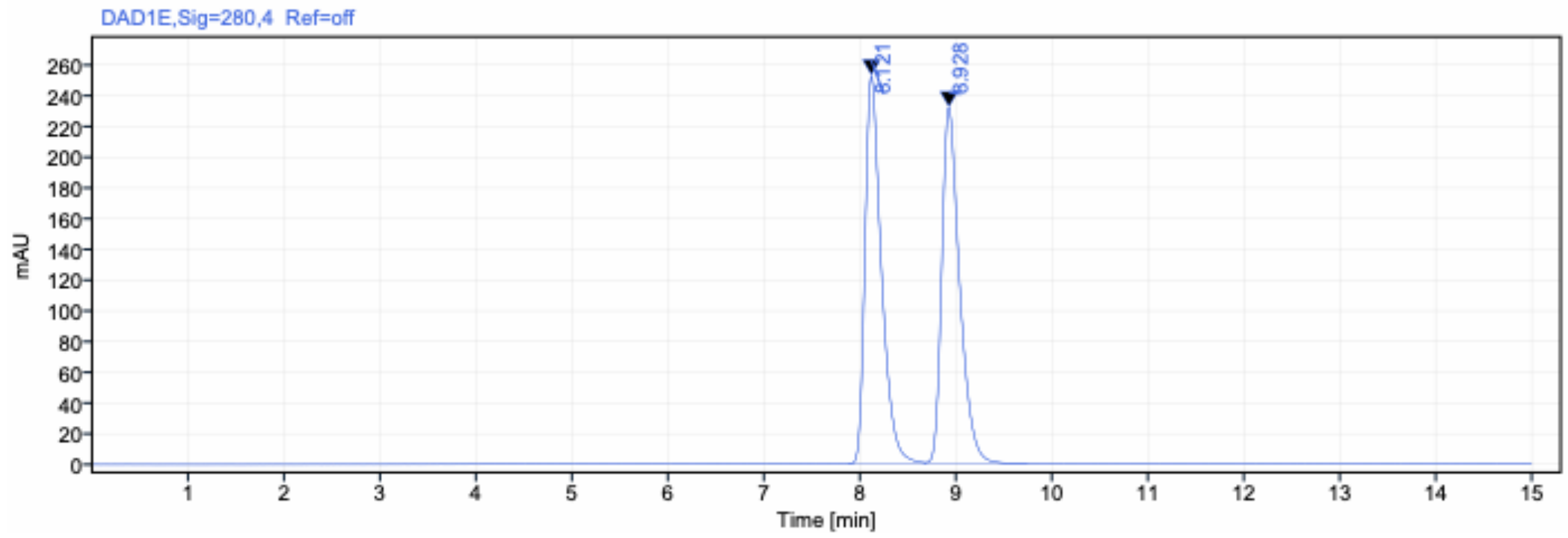

Signal: DAD1E,Sig=280,4 Ref=off

RT [min]	Type	Width [min]	Area	Height	Area%	Name
8.121	BV	0.81	3007.05	252.77	50.08	
8.928	VB	1.06	2997.89	231.90	49.92	

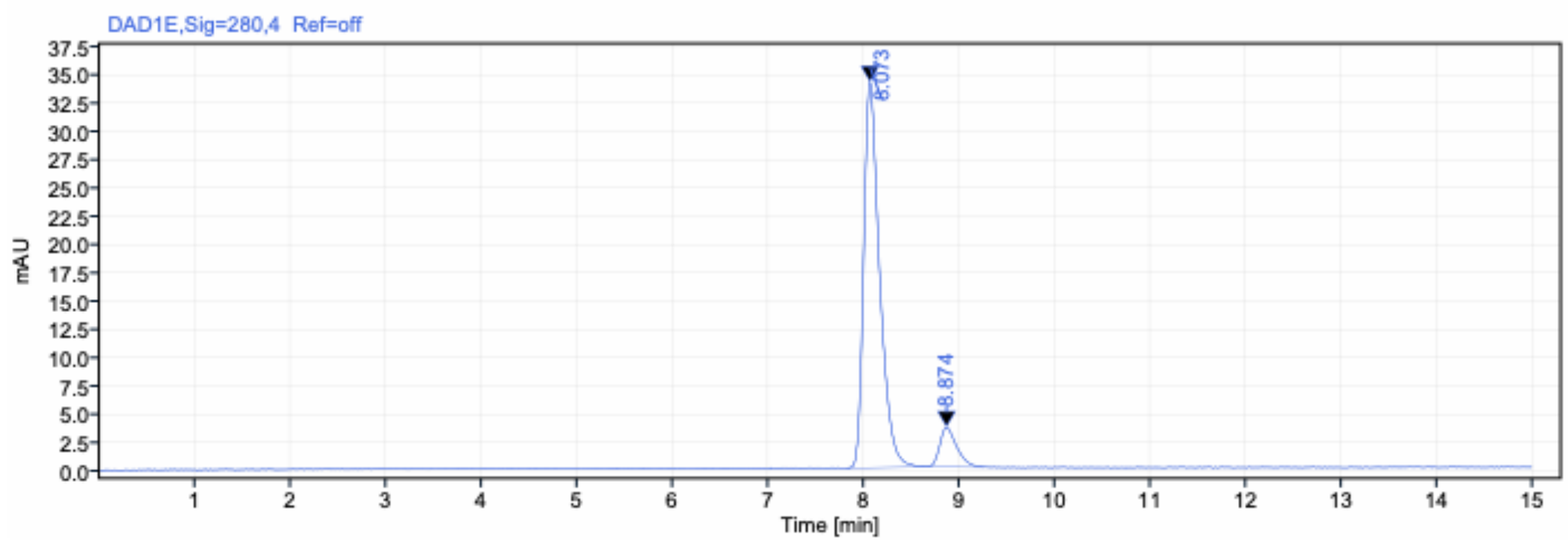

Signal: DAD1E,Sig=280,4 Ref=off

RT [min]	Type	Width [min]	Area	Height	Area%	Name
8.073	BB	0.81	394.71	34.08	90.16	
8.874	MM m	0.62	43.07	3.51	9.84	

1h and racemic standard (8% isopropyl alcohol in hexanes, AD-H, 20 min)

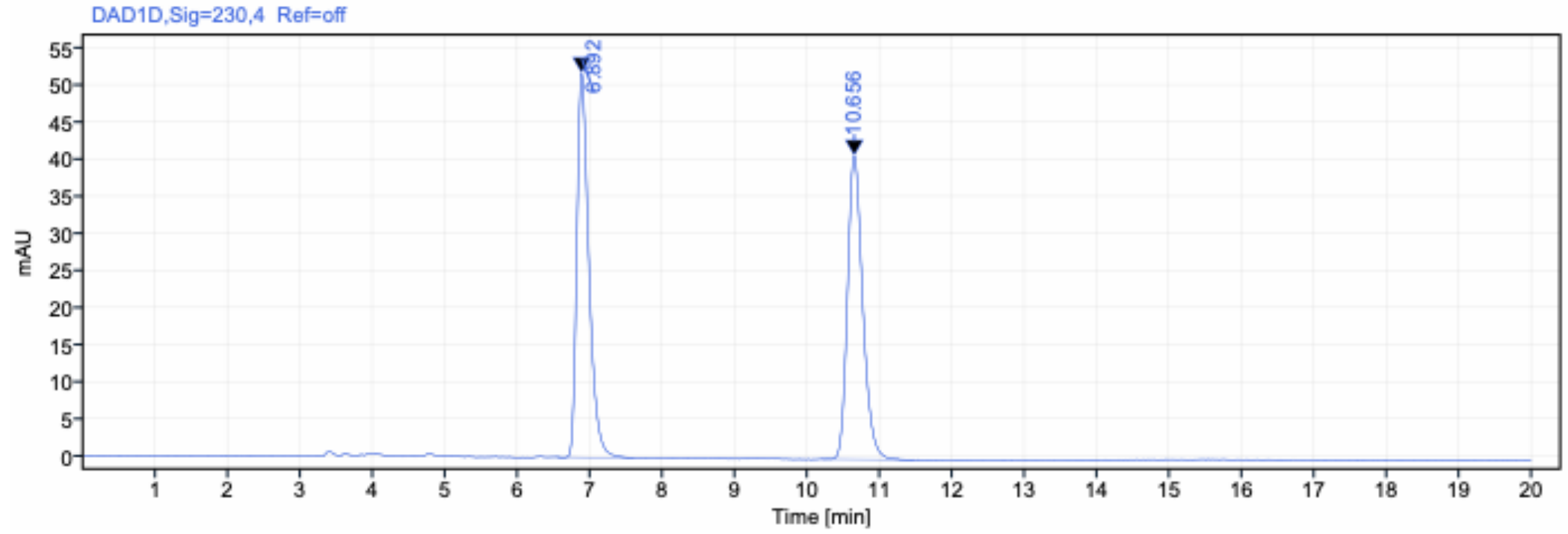

Signal: DAD1D,Sig=230,4 Ref=off

RT [min]	Type	Width [min]	Area	Height	Area%	Name
6.892	MM m	1.07	597.33	51.74	50.04	
10.656	MM m	1.28	596.48	40.89	49.96	

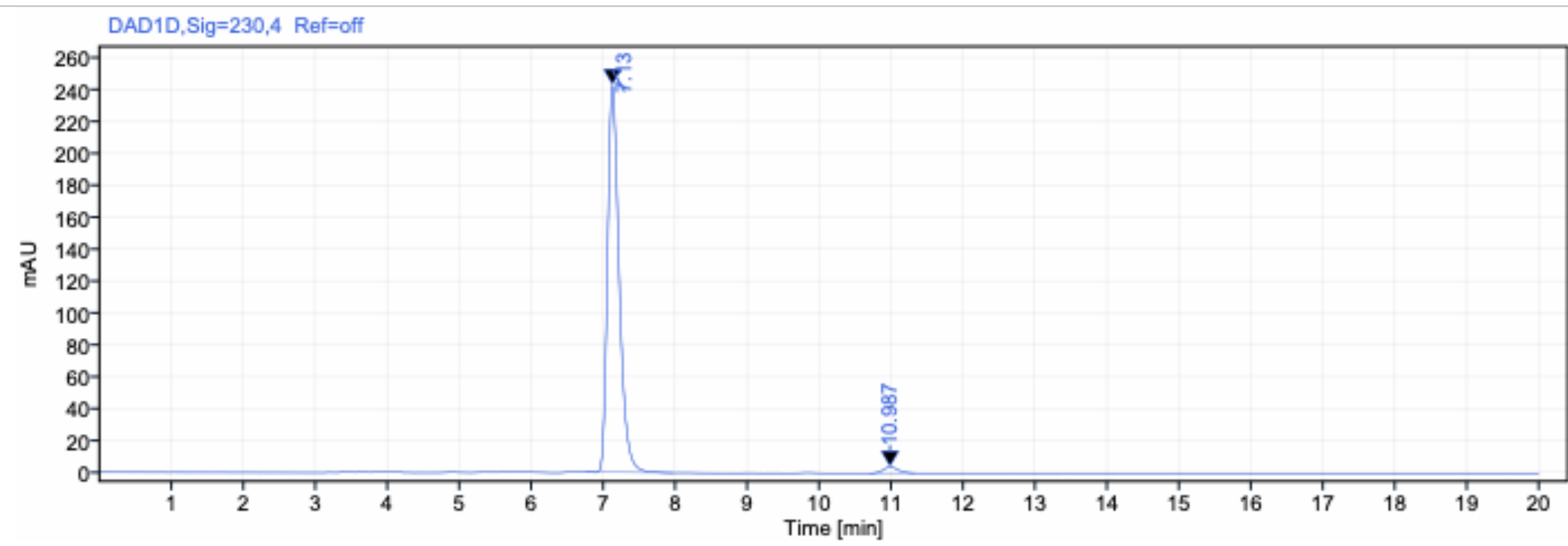

Signal: DAD1D,Sig=230,4 Ref=off

RT [min]	Type	Width [min]	Area	Height	Area%	Name
7.130	MM m	1.22	2721.25	242.54	98.07	
10.987	MM m	0.63	53.54	3.85	1.93	

1i and racemic standard (20% isopropyl alcohol in hexanes, AD-H, 30 min)

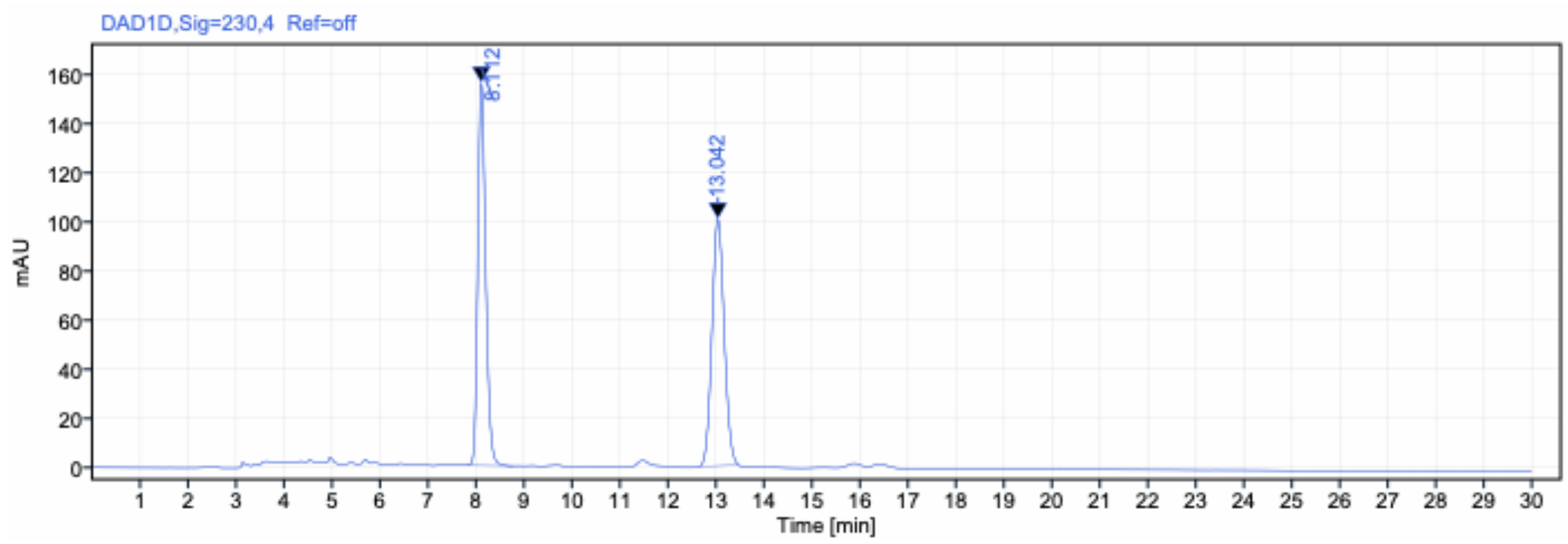

Signal: DAD1D,Sig=230,4 Ref=off

RT [min]	Type	Width [min]	Area	Height	Area%	Name
8.112	MM m	0.98	1774.32	155.77	49.98	
13.042	MM m	0.81	1775.85	100.49	50.02	

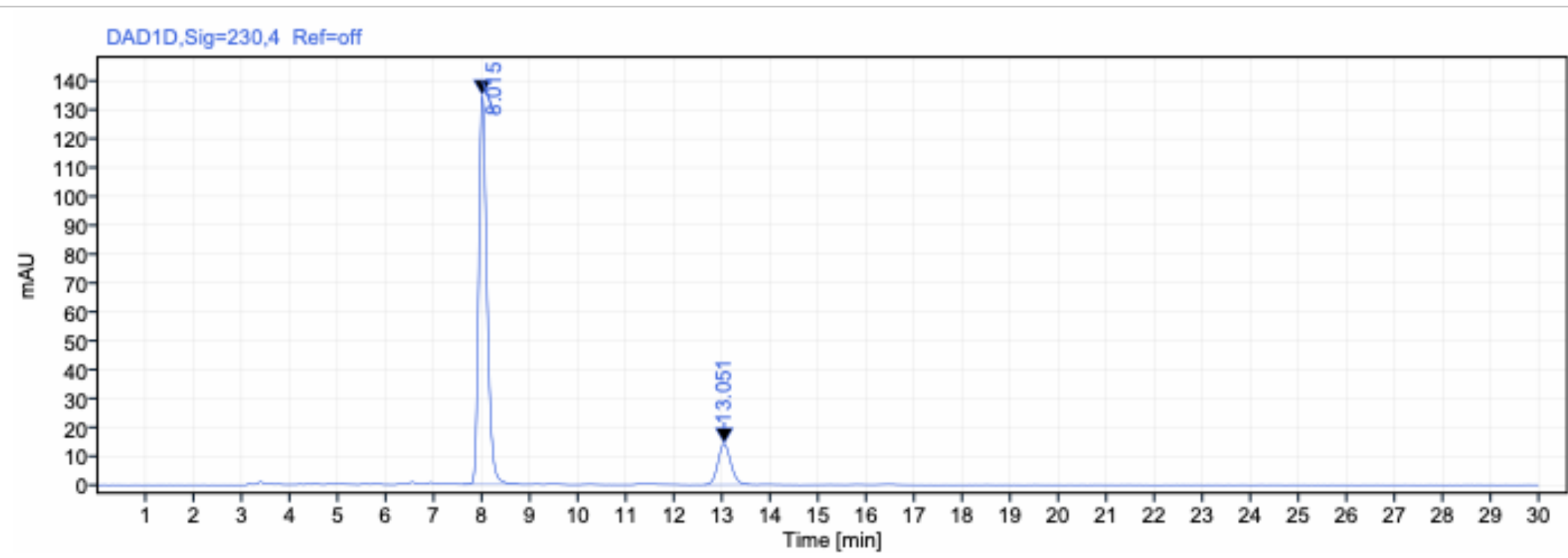

Signal: DAD1D,Sig=230,4 Ref=off

RT [min]	Type	Width [min]	Area	Height	Area%	Name
8.015	MM m	1.15	1605.42	134.54	85.96	
13.051	BB	0.93	262.15	14.09	14.04	

1j and racemic standard (10% isopropyl alcohol in hexanes, AD-H, 30 min)

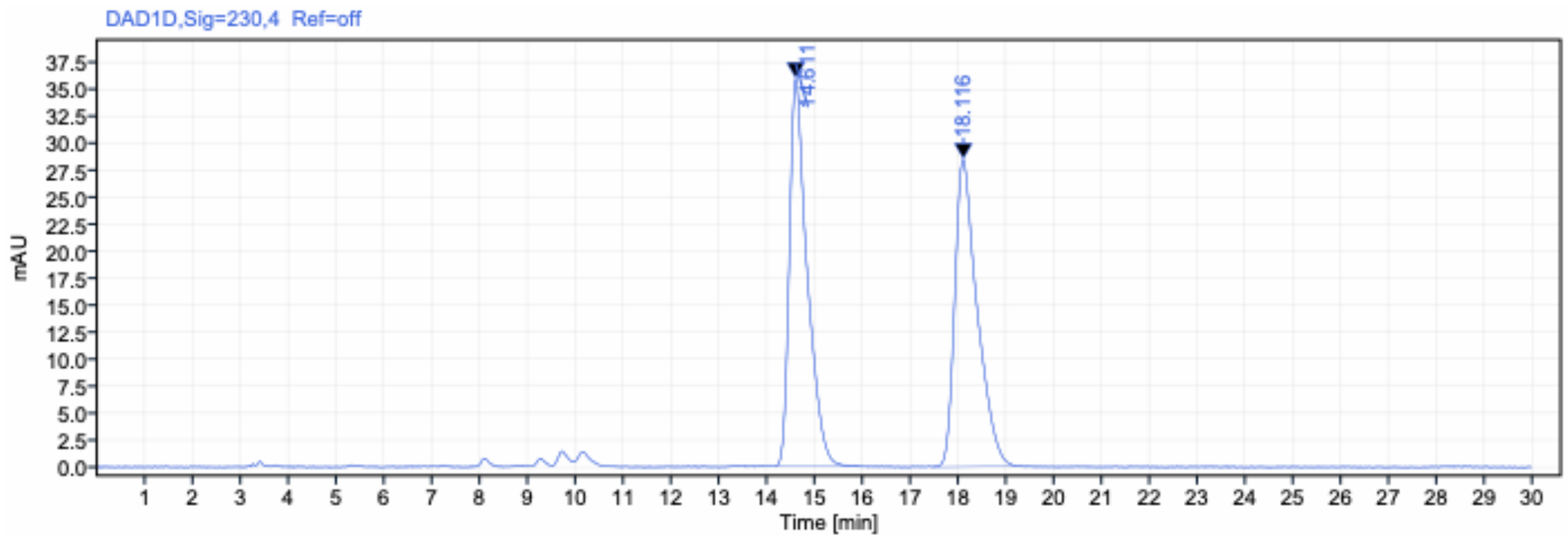

Signal: DAD1D,Sig=230,4 Ref=off

RT [min]	Type	Width [min]	Area	Height	Area%	Name
14.611	BM m	1.73	929.08	35.90	50.02	
18.116	BB	1.75	928.26	28.47	49.98	

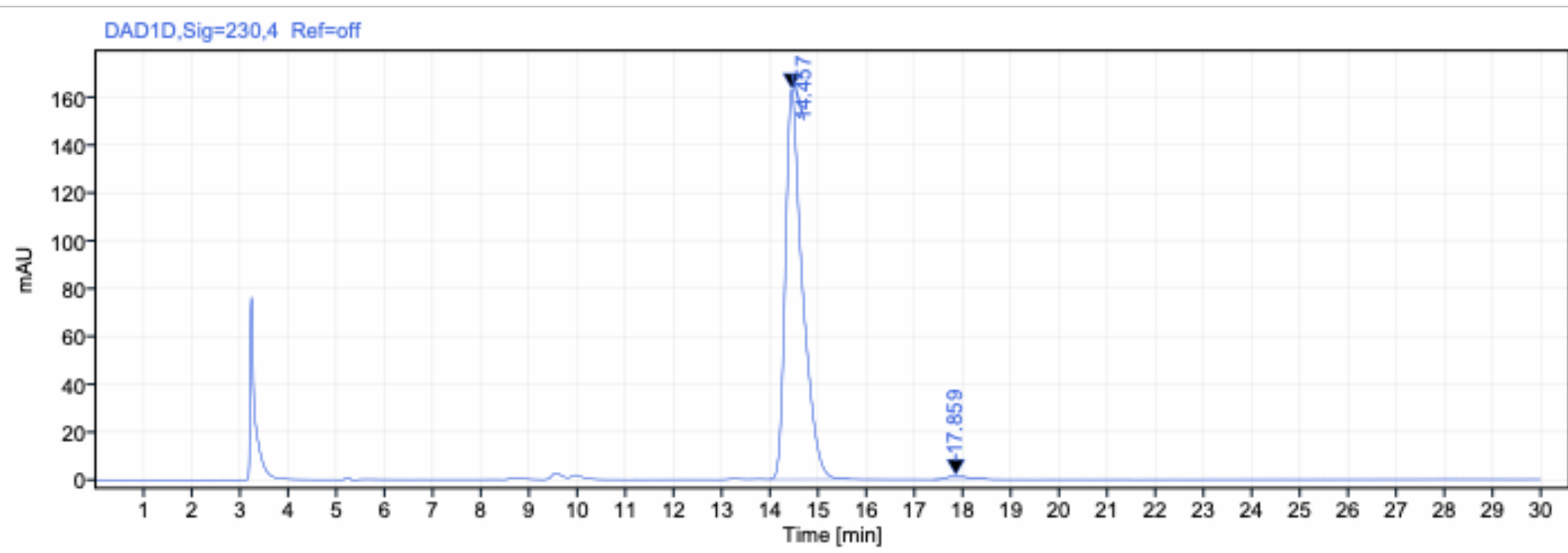

Signal: DAD1D,Sig=230,4 Ref=off

RT [min]	Type	Width [min]	Area	Height	Area%	Name
14.457	BB	1.66	4014.50	162.96	98.96	
17.859	MM m	1.11	42.37	1.55	1.04	

1k and racemic standard (10% isopropyl alcohol in hexanes, AD-H, 15 min)

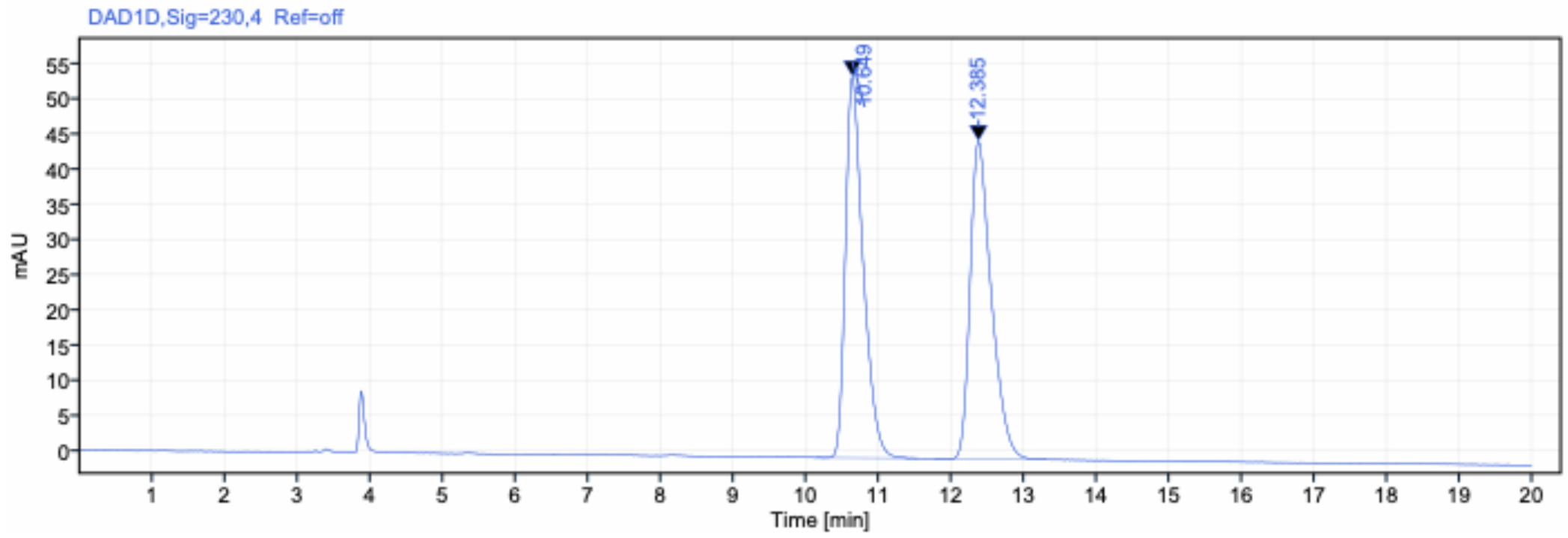

Signal: DAD1D,Sig=230,4 Ref=off

RT [min]	Type	Width [min]	Area	Height	Area%	Name
10.649	MM m	1.40	917.56	54.12	50.09	
12.385	MM m	1.53	914.22	45.12	49.91	

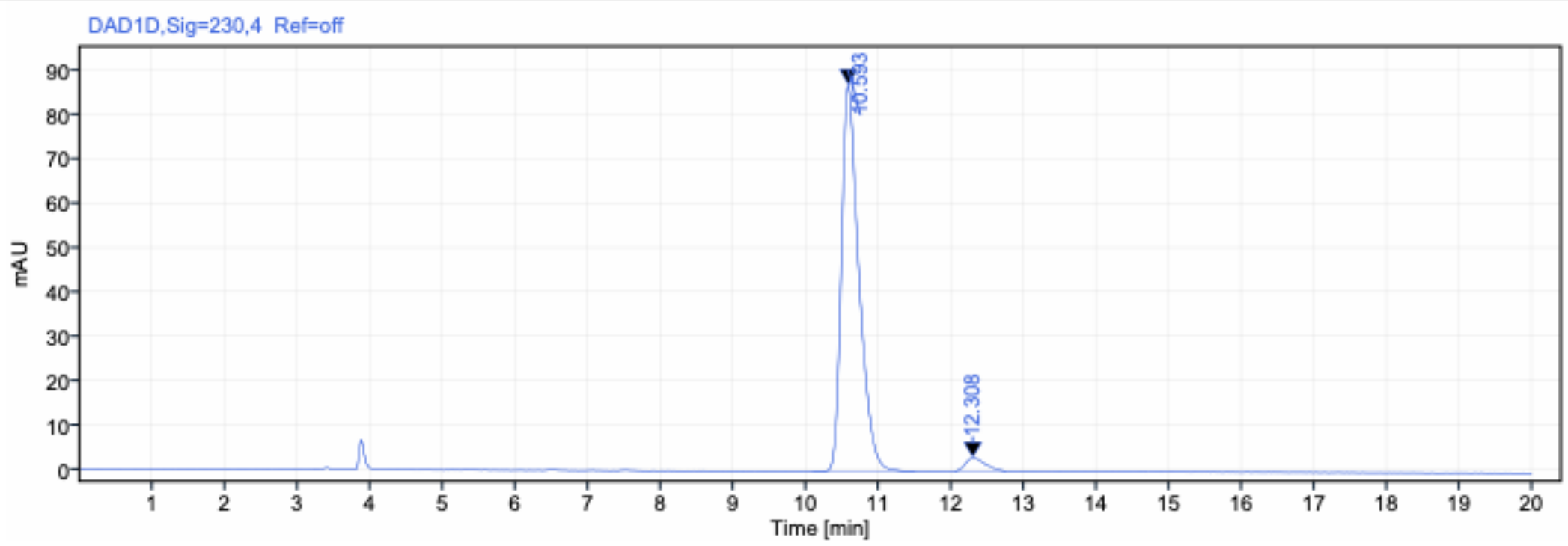

Signal: DAD1D,Sig=230,4 Ref=off

RT [min]	Type	Width [min]	Area	Height	Area%	Name
10.593	MM m	1.41	1466.57	87.04	96.32	
12.308	MM m	0.71	55.98	2.99	3.68	

1l and racemic standard (10% isopropyl alcohol in hexanes, AD-H, 15 min)

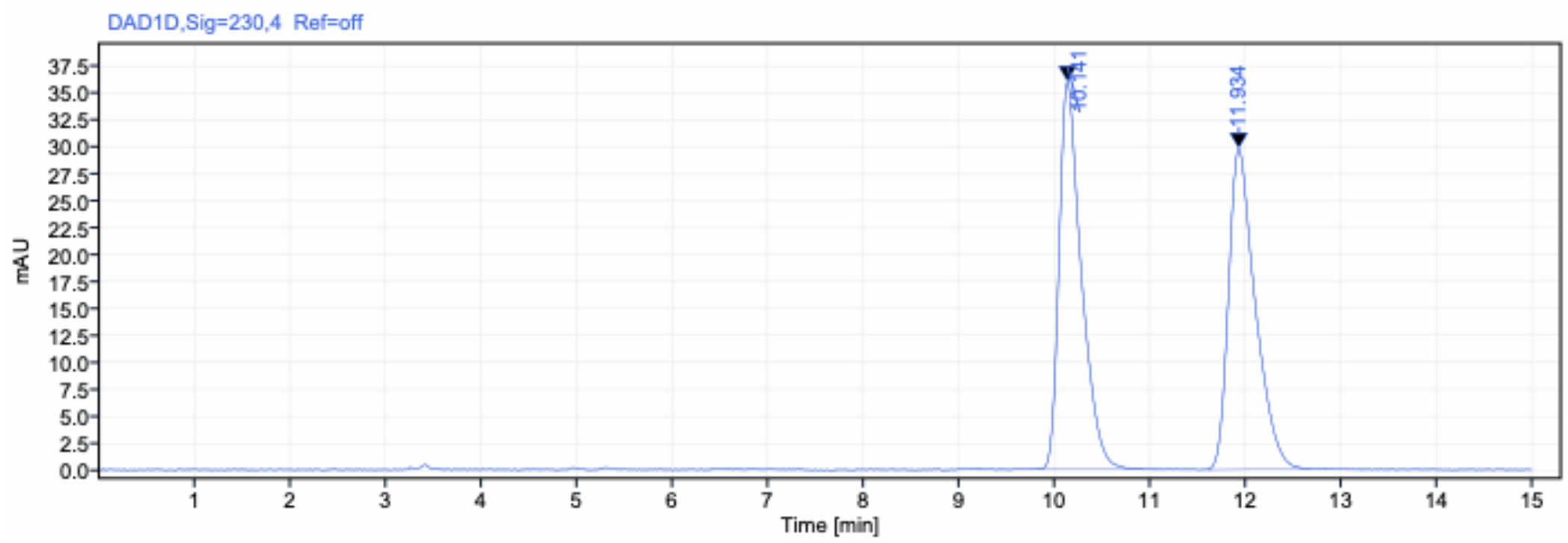

Signal: DAD1D,Sig=230,4 Ref=off

RT [min]	Type	Width [min]	Area	Height	Area%	Name
10.141	MM m	1.12	580.04	35.87	50.08	
11.934	BM m	1.09	578.12	29.72	49.92	

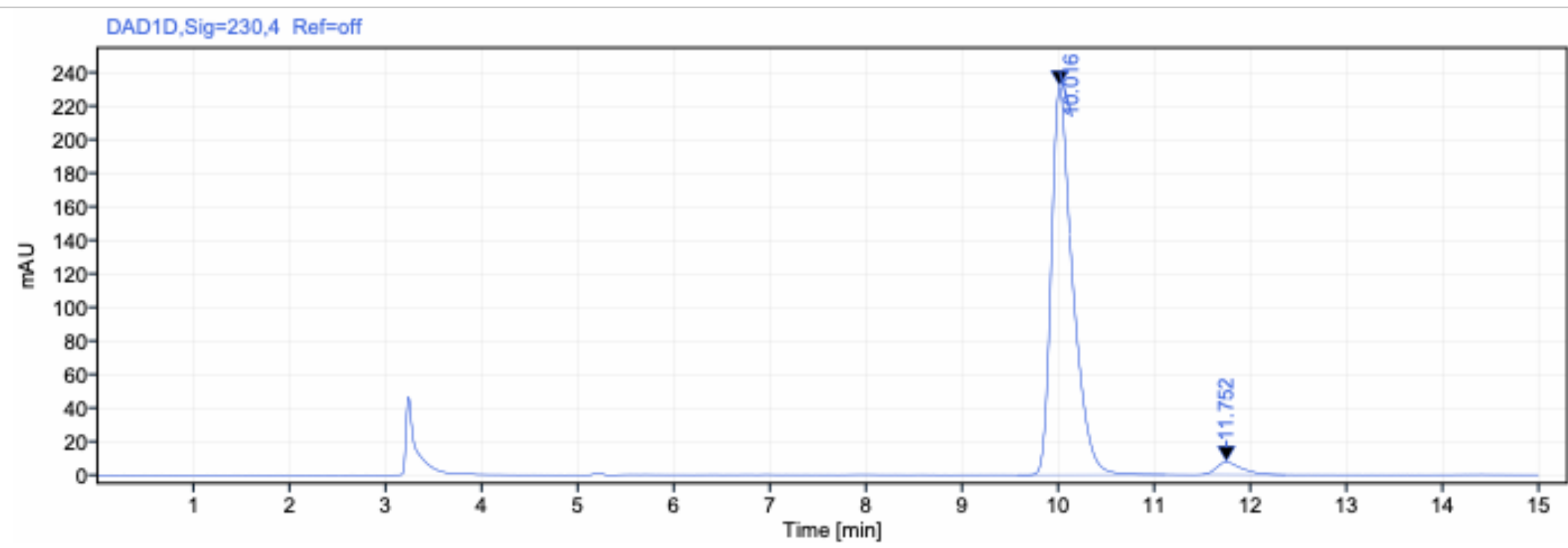

Signal: DAD1D,Sig=230,4 Ref=off

RT [min]	Type	Width [min]	Area	Height	Area%	Name
10.016	MM m	1.54	3573.14	231.43	96.36	
11.752	BB	0.86	134.83	7.46	3.64	

1m and racemic standard (20% isopropyl alcohol in hexanes, AD-H, 30 min)

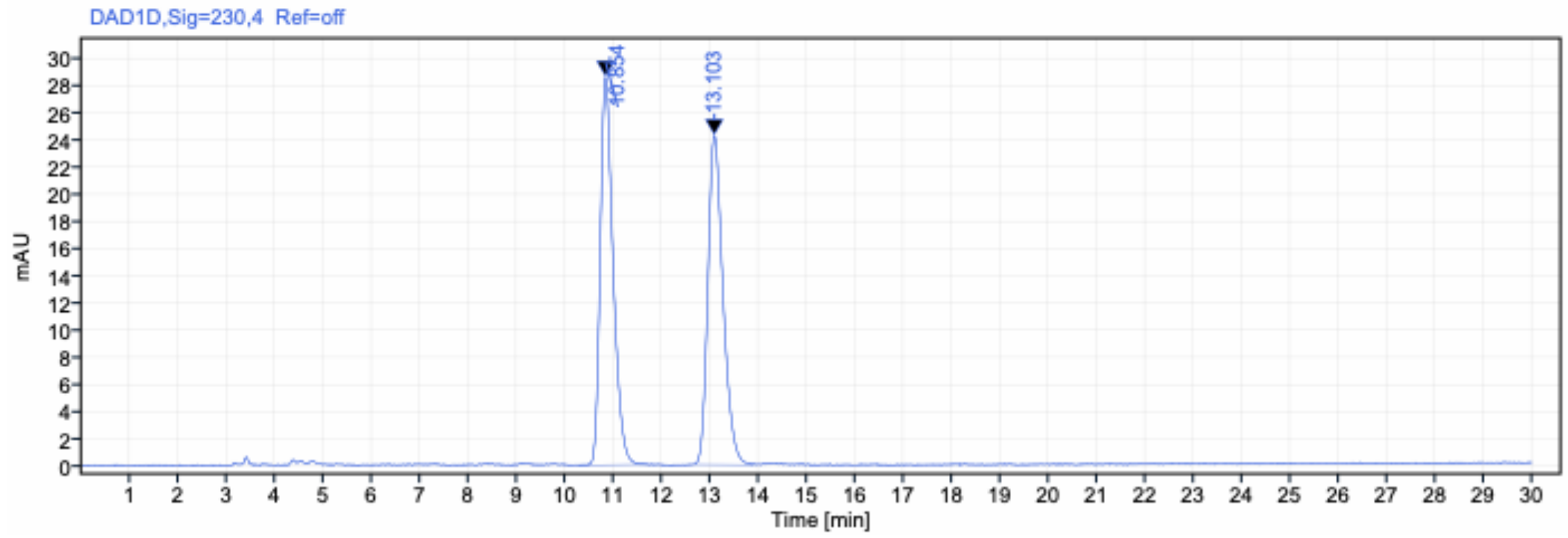

Signal: DAD1D,Sig=230,4 Ref=off

RT [min]	Type	Width [min]	Area	Height	Area%	Name
10.854	MM m	1.44	526.01	28.54	50.00	
13.103	BB R	1.20	526.00	24.23	50.00	

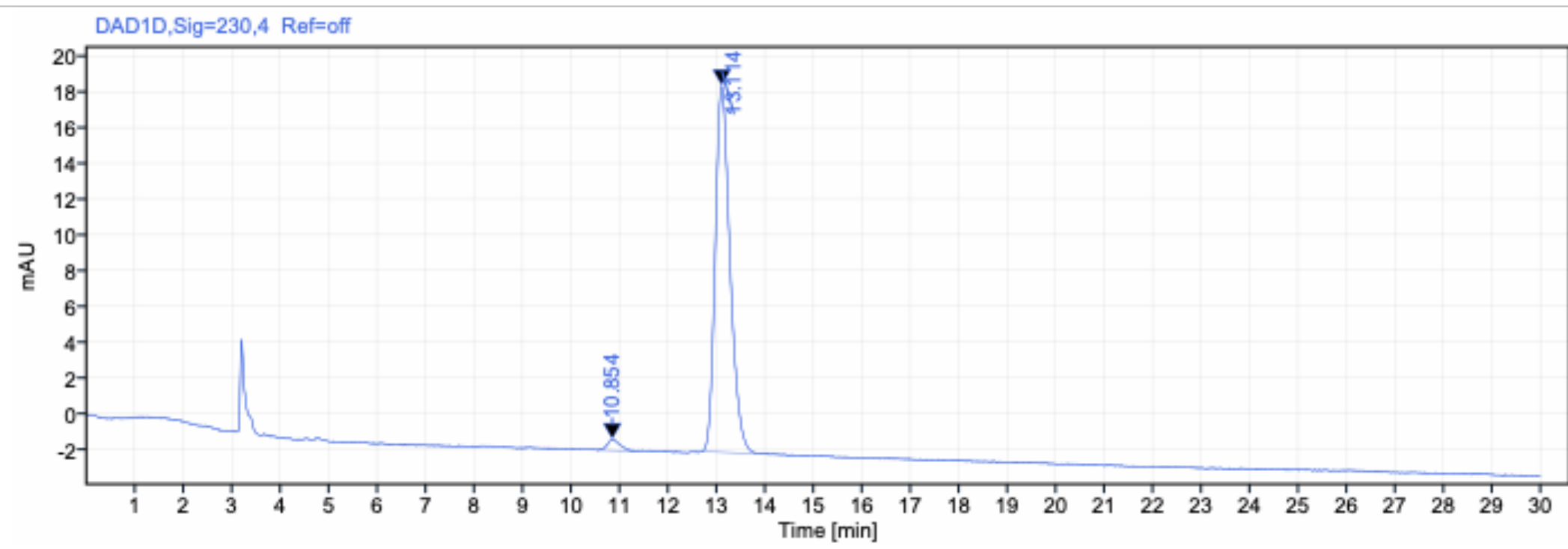

Signal: DAD1D,Sig=230,4 Ref=off

RT [min]	Type	Width [min]	Area	Height	Area%	Name
10.854	MM m	0.79	10.90	0.61	2.44	
13.114	BB	1.08	434.92	20.50	97.56	

1n and racemic standard (10% isopropyl alcohol in hexanes, AD-H, 15 min)

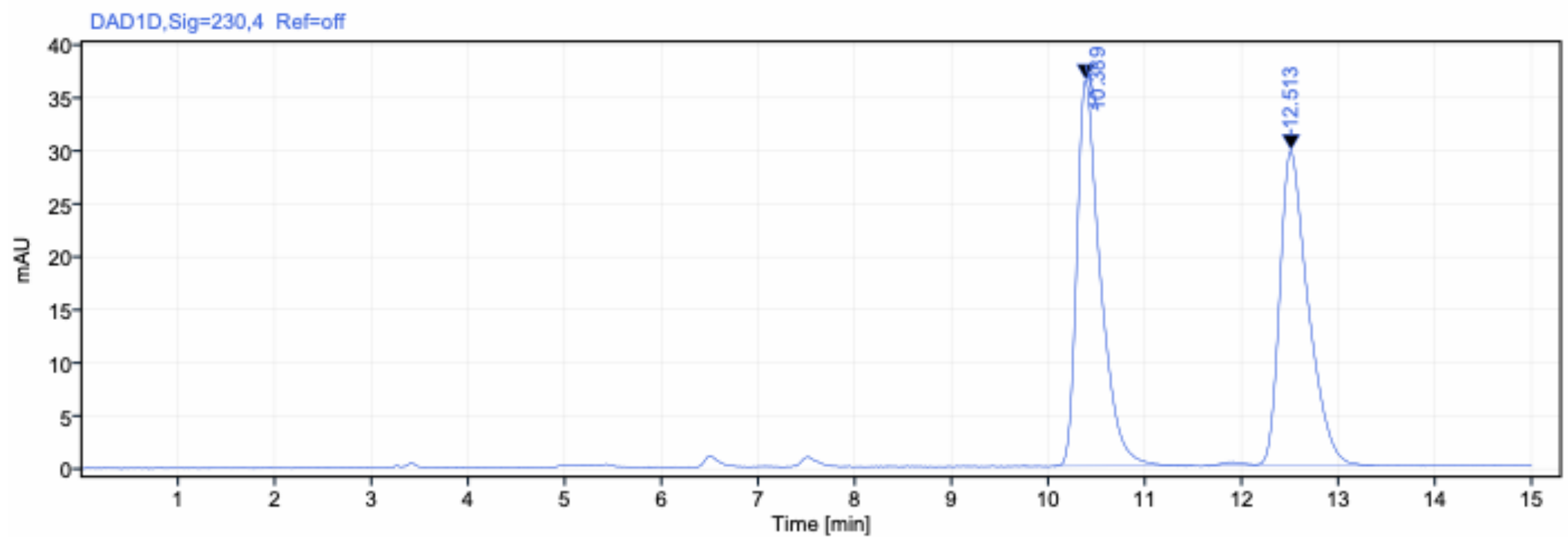

Signal: DAD1D,Sig=230,4 Ref=off

RT [min]	Type	Width [min]	Area	Height	Area%	Name
10.389	MM m	1.44	613.90	36.41	50.15	
12.513	MM m	1.75	610.17	29.72	49.85	

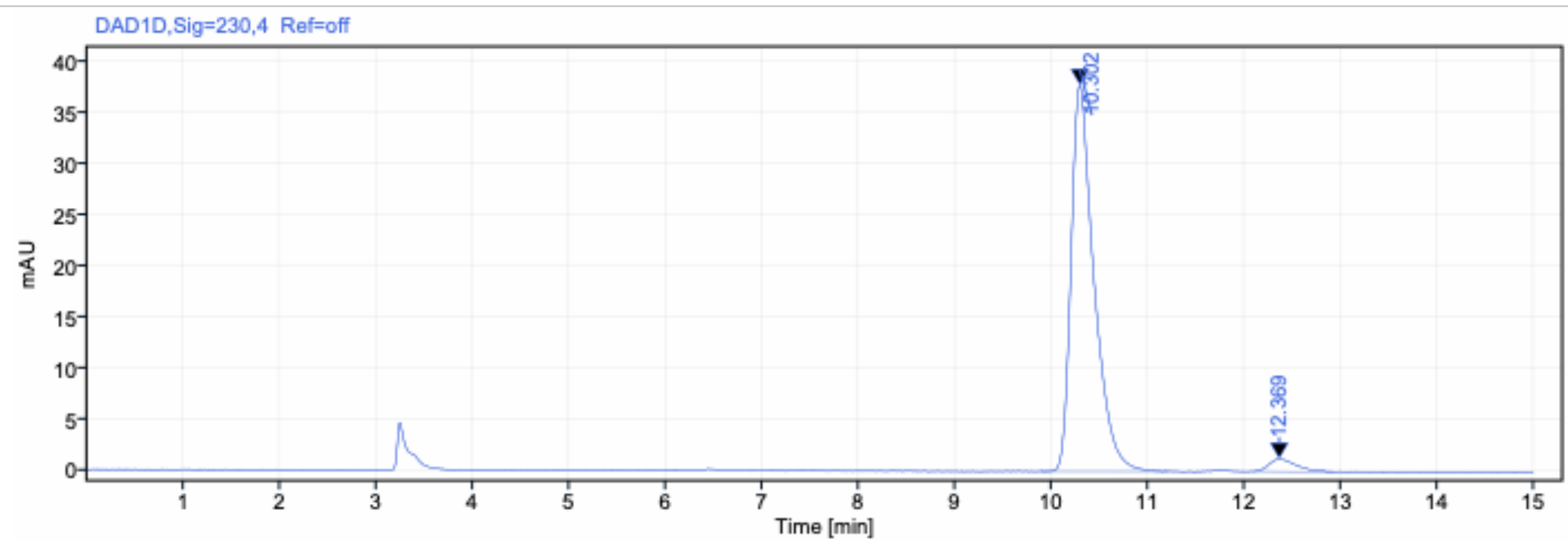

Signal: DAD1D,Sig=230,4 Ref=off

RT [min]	Type	Width [min]	Area	Height	Area%	Name
10.302	MM m	1.32	622.58	37.78	96.23	
12.369	MM m	0.83	24.37	1.24	3.77	

1o and racemic standard (10% isopropyl alcohol in hexanes, AD-H, 20 min)

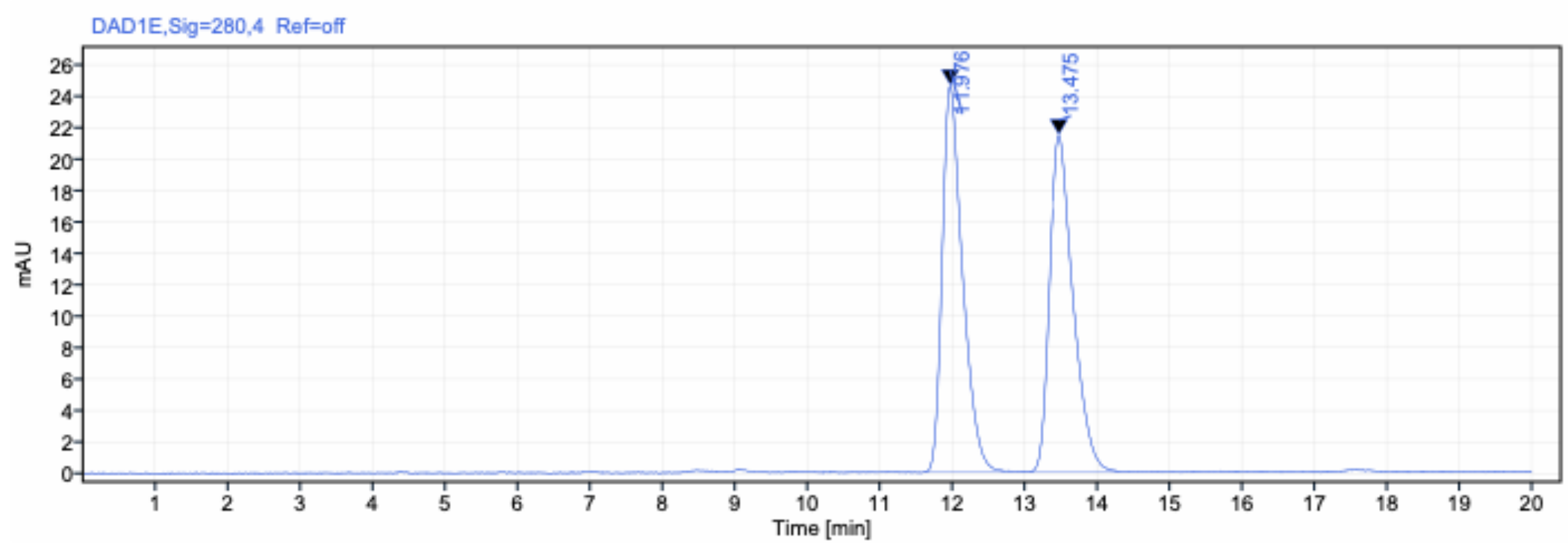

Signal: DAD1E,Sig=280,4 Ref=off

RT [min]	Type	Width [min]	Area	Height	Area%	Name
11.976	BB	1.09	479.30	24.59	50.04	
13.475	BB	1.22	478.46	21.42	49.96	

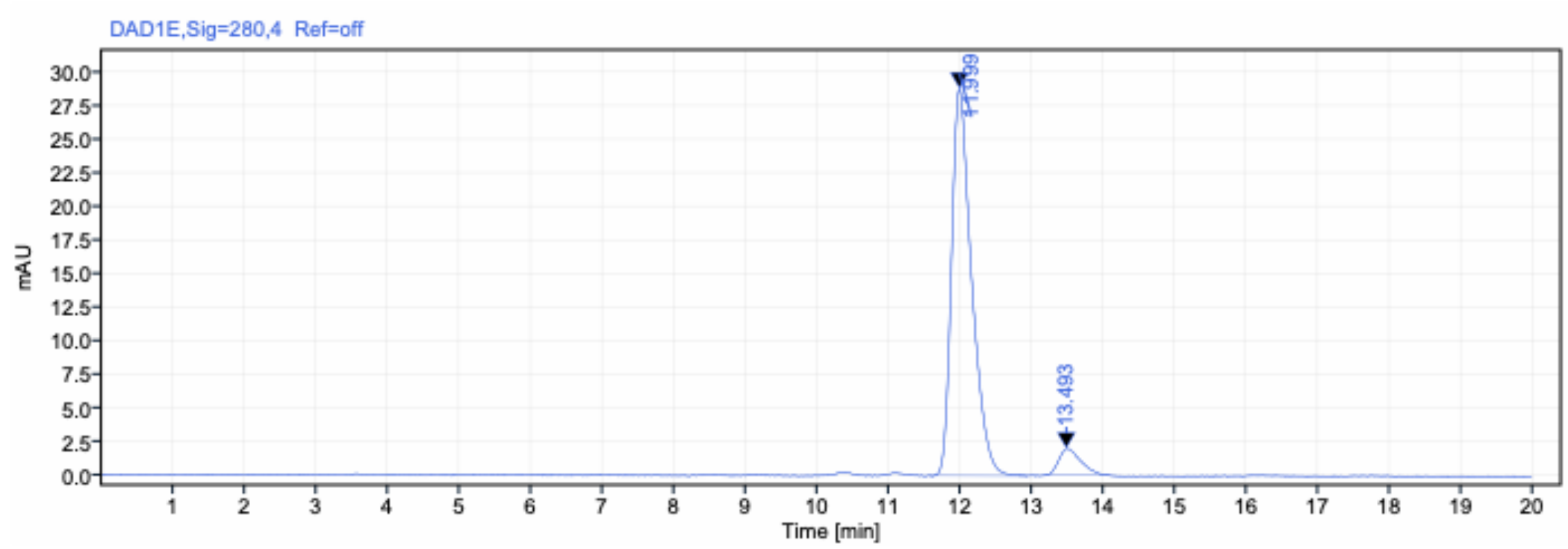

Signal: DAD1E,Sig=280,4 Ref=off

RT [min]	Type	Width [min]	Area	Height	Area%	Name
11.999	MM m	1.37	561.54	28.83	92.90	
13.493	MM m	0.92	42.93	2.00	7.10	

1p and racemic standard (5% isopropyl alcohol in hexanes, AD-H, 20 min)

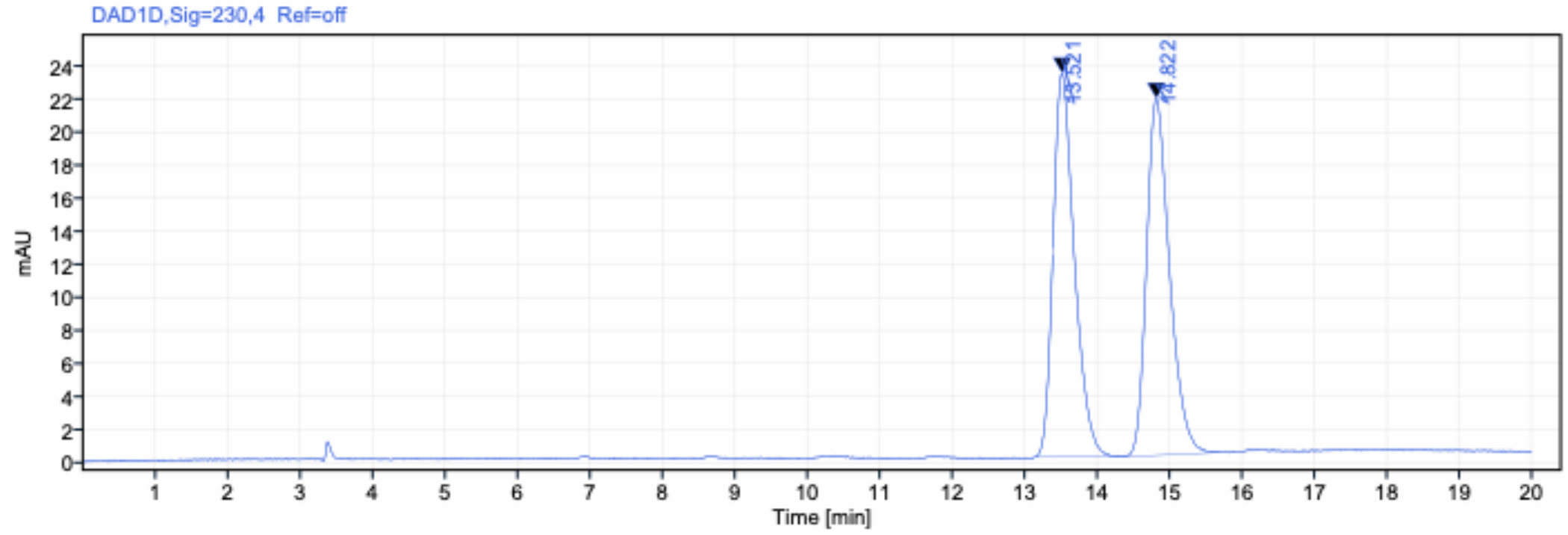

Signal: DAD1D,Sig=230,4 Ref=off

RT [min]	Type	Width [min]	Area	Height	Area%	Name
13.521	BM m	1.23	475.38	23.20	49.94	
14.822	BM m	1.31	476.52	21.56	50.06	

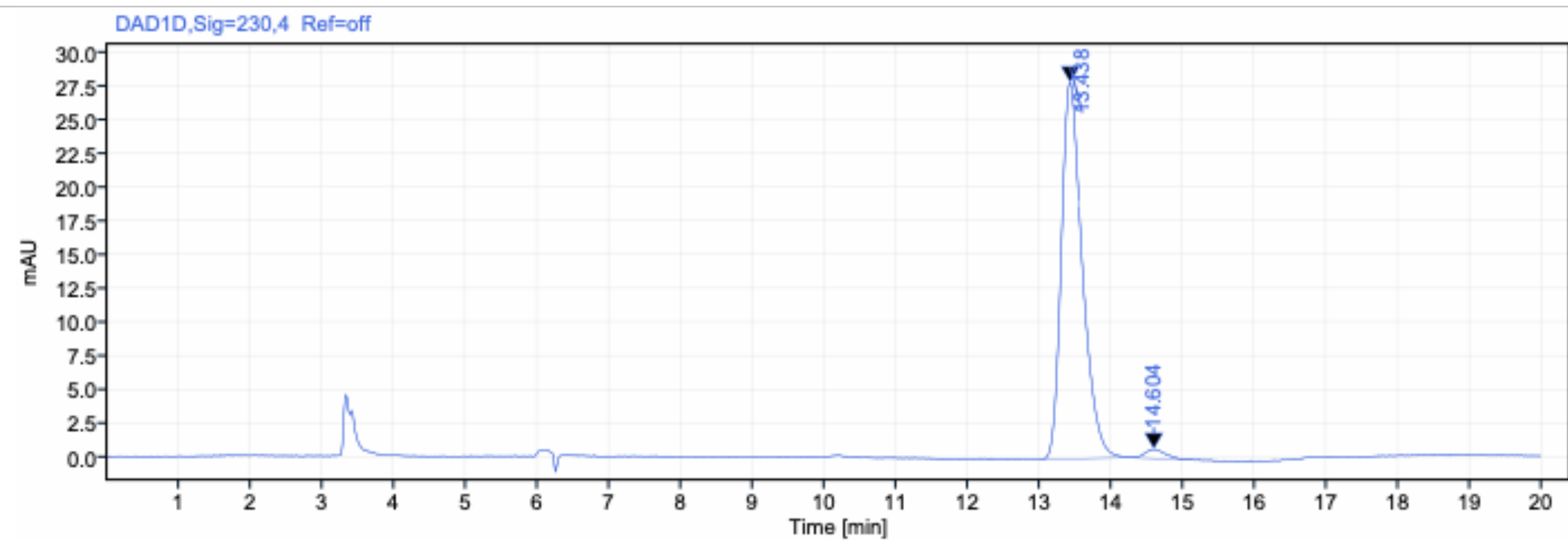

Signal: DAD1D,Sig=230,4 Ref=off

RT [min]	Type	Width [min]	Area	Height	Area%	Name
13.438	BM m	1.19	565.74	27.89	97.70	
14.604	MM m	0.90	13.29	0.69	2.30	

1q and racemic standard (15% isopropyl alcohol in hexanes, AD-H, 30 min)

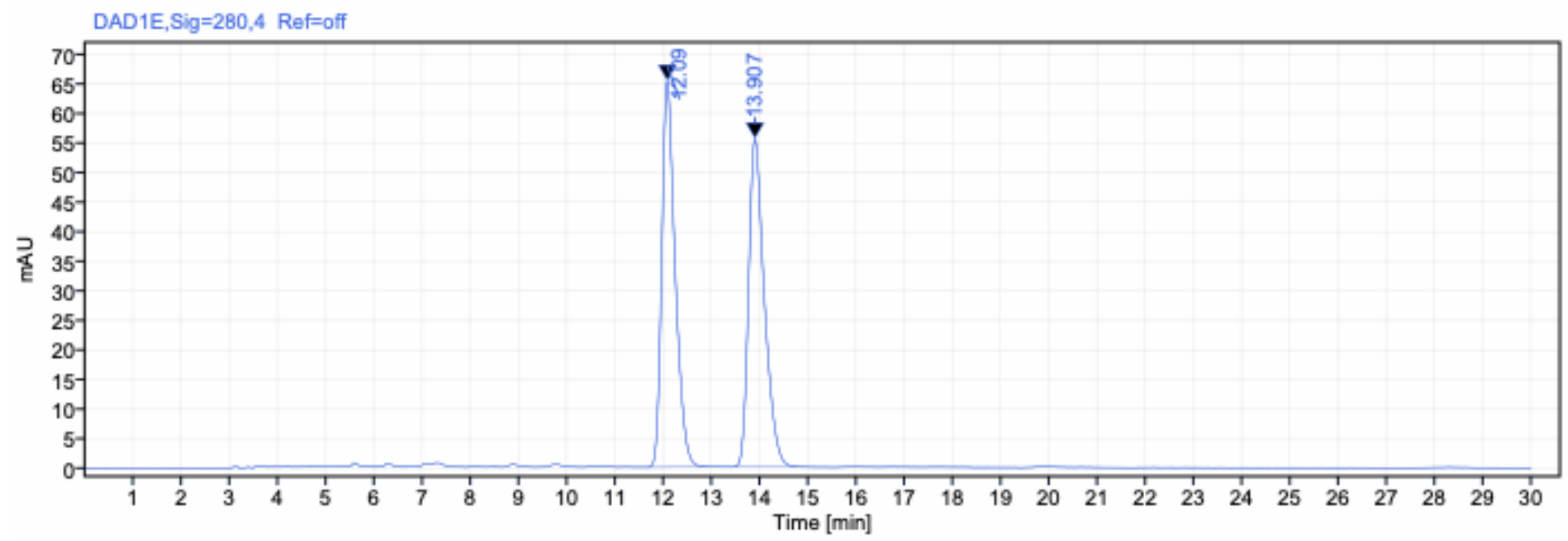

Signal: DAD1E,Sig=280,4 Ref=off

RT [min]	Type	Width [min]	Area	Height	Area%	Name
12.090	BM m	1.23	1257.50	65.32	50.00	
13.907	BB	1.32	1257.38	55.53	50.00	

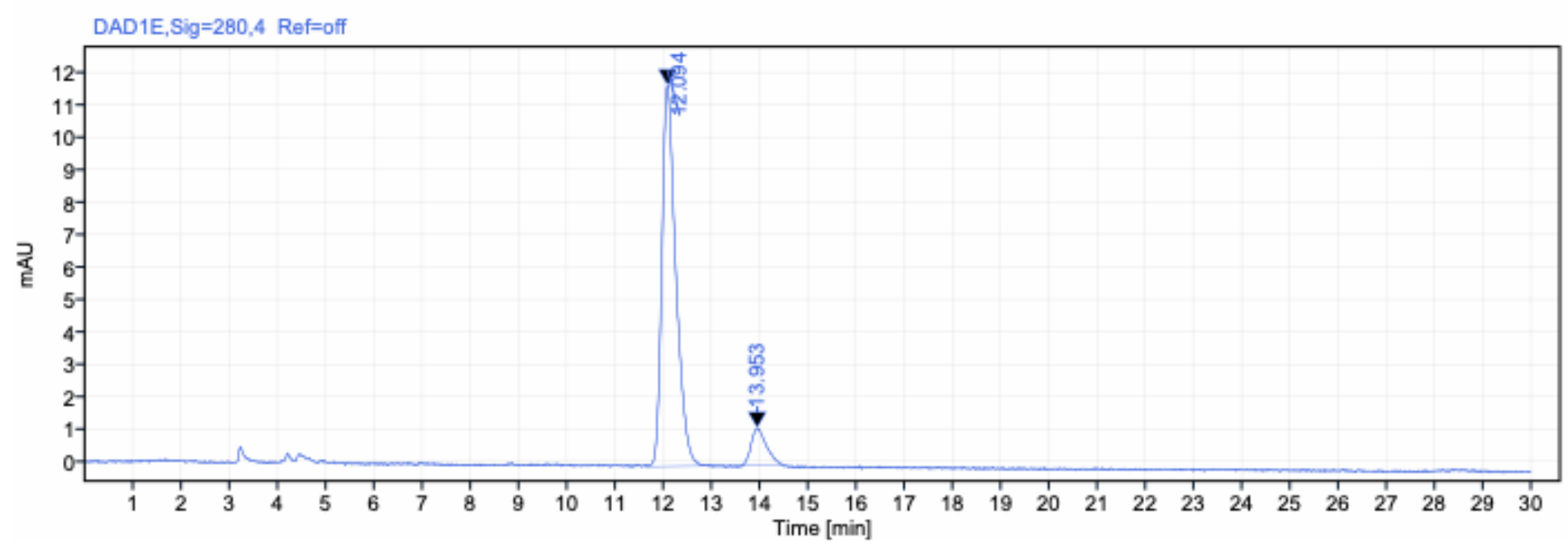

Signal: DAD1E,Sig=280,4 Ref=off

RT [min]	Type	Width [min]	Area	Height	Area%	Name
12.094	MM m	1.35	236.16	11.75	90.24	
13.953	MM m	1.03	25.55	1.16	9.76	

1r and racemic standard (10% isopropyl alcohol in hexanes, AD-H, 20 min)

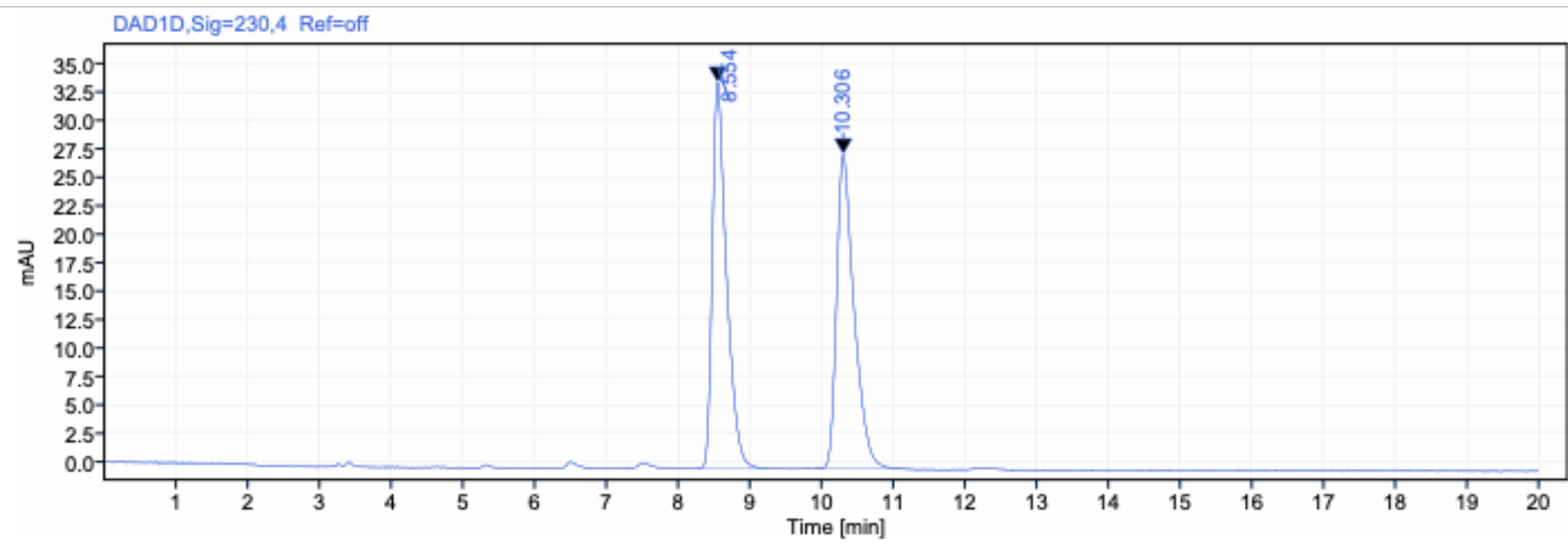

Signal:	DAD1D,Sig=230,4 Ref=off					
RT [min]	Type	Width [min]	Area	Height	Area%	Name
8.554	MM m	0.99	463.88	33.89	49.98	
10.306	MM m	1.20	464.23	27.53	50.02	

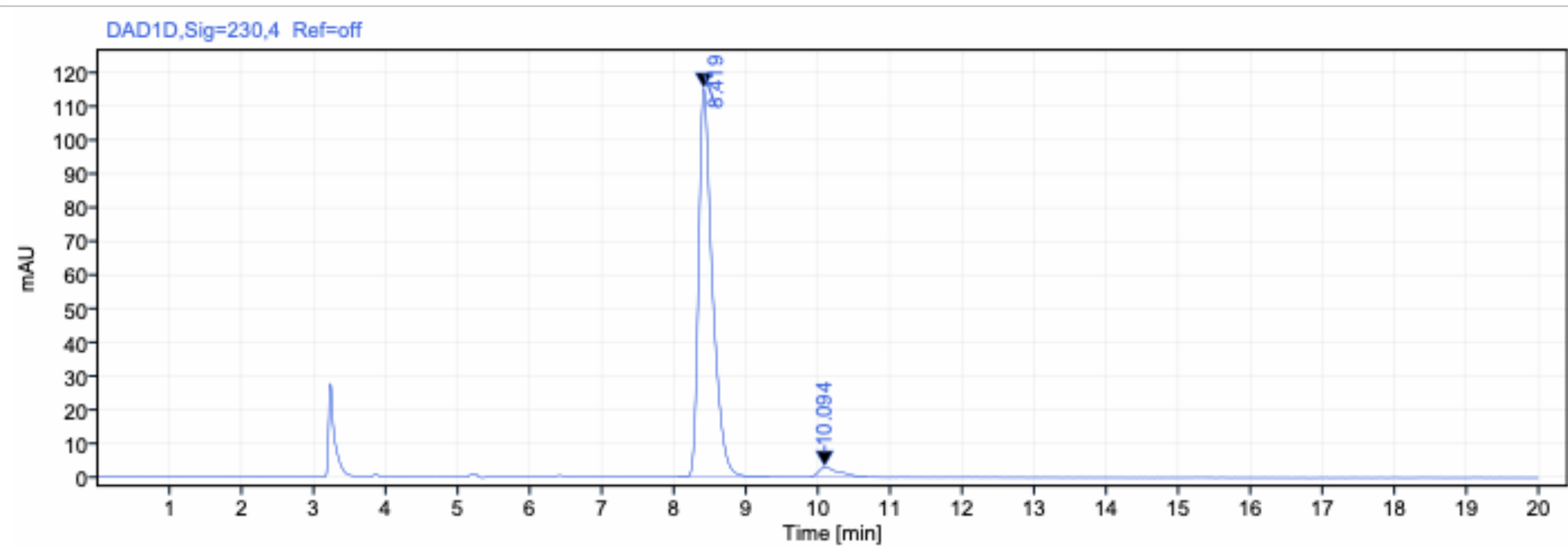

Signal:	DAD1D,Sig=230,4 Ref=off					
RT [min]	Type	Width [min]	Area	Height	Area%	Name
8.419	MM m	1.28	1528.41	115.22	96.47	
10.094	MM m	0.80	55.90	2.97	3.53	

1s and racemic standard (10% isopropyl alcohol in hexanes, AD-H, 20 min)

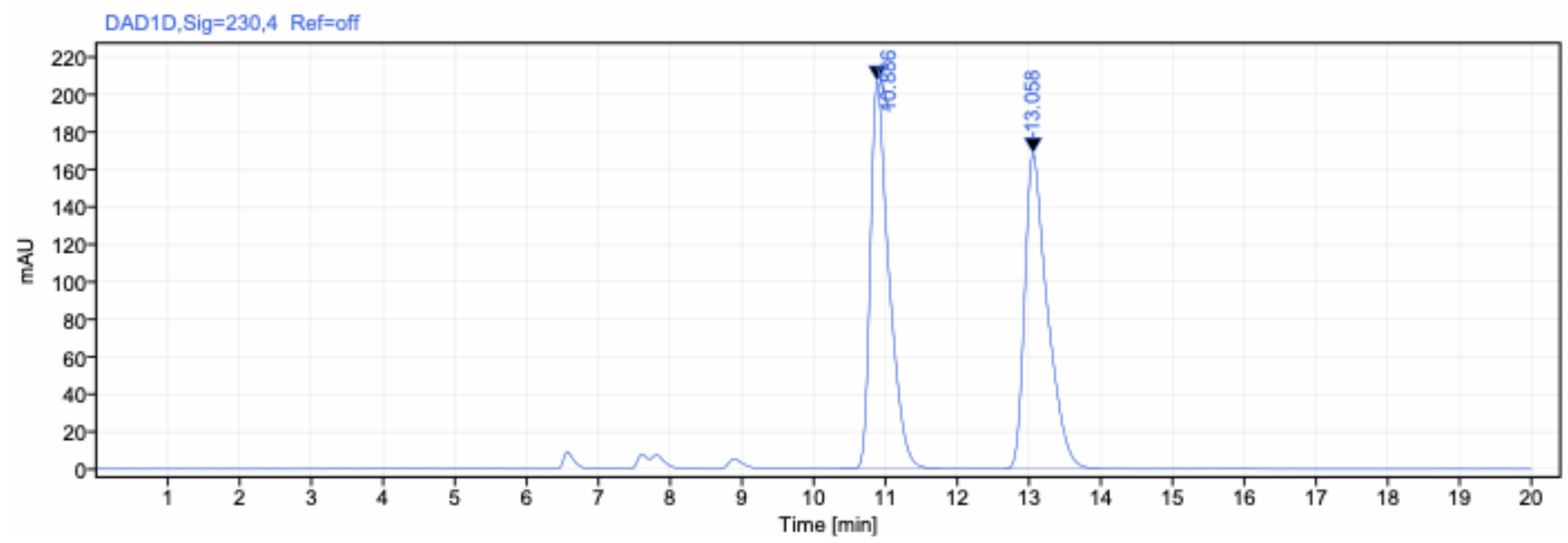

Signal: DAD1D,Sig=230,4 Ref=off

RT [min]	Type	Width [min]	Area	Height	Area%	Name
10.886	MM m	1.42	3703.27	206.56	50.02	
13.058	BM m	1.32	3700.95	168.22	49.98	

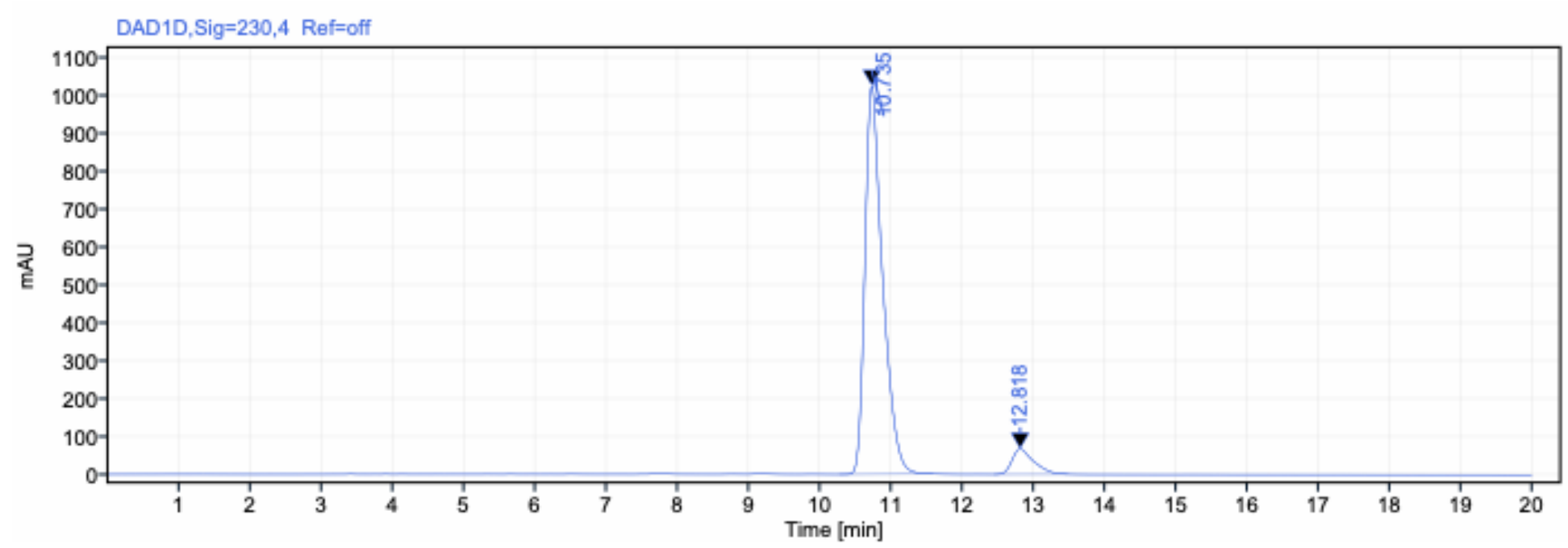

Signal: DAD1D,Sig=230,4 Ref=off

RT [min]	Type	Width [min]	Area	Height	Area%	Name
10.735	MM m	1.31	17653.63	1024.24	92.63	
12.818	BM m	1.04	1405.22	66.71	7.37	

1t and racemic standard (20% isopropyl alcohol in hexanes, AS-H, 30 min)

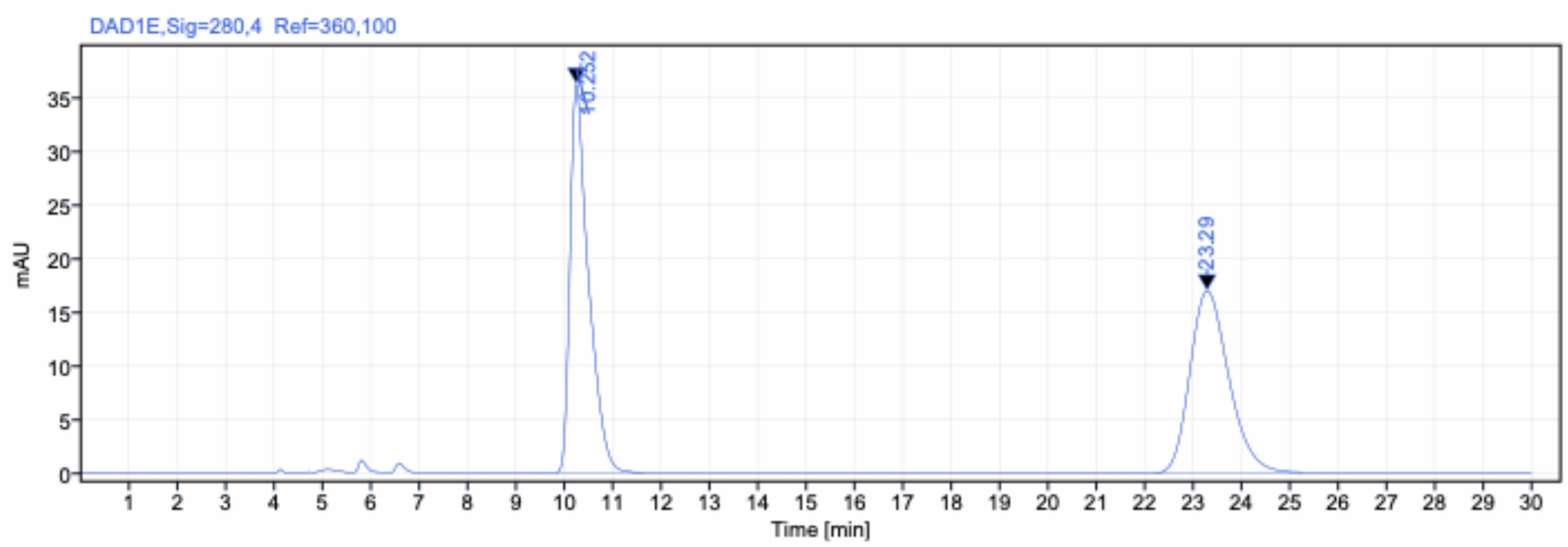

Signal: DAD1E,Sig=280,4 Ref=360,100

RT [min]	Type	Width [min]	Area	Height	Area%	Name
10.252	BB	1.80	957.06	36.21	50.27	
23.290	BB	3.07	946.86	16.99	49.73	

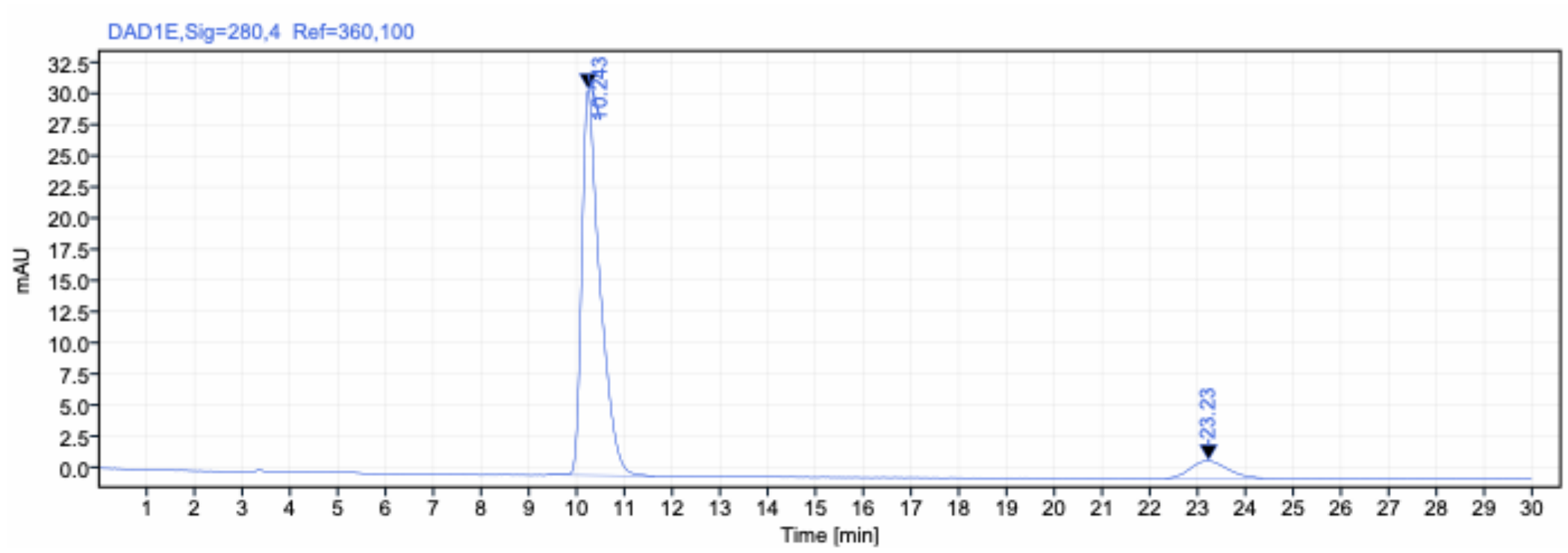

Signal: DAD1E,Sig=280,4 Ref=360,100

RT [min]	Type	Width [min]	Area	Height	Area%	Name
10.243	MB m	2.23	815.13	30.94	91.89	
23.230	MM m	2.04	71.99	1.39	8.11	

1u and racemic standard (10% isopropyl alcohol in hexanes, OD-H, 20 min)

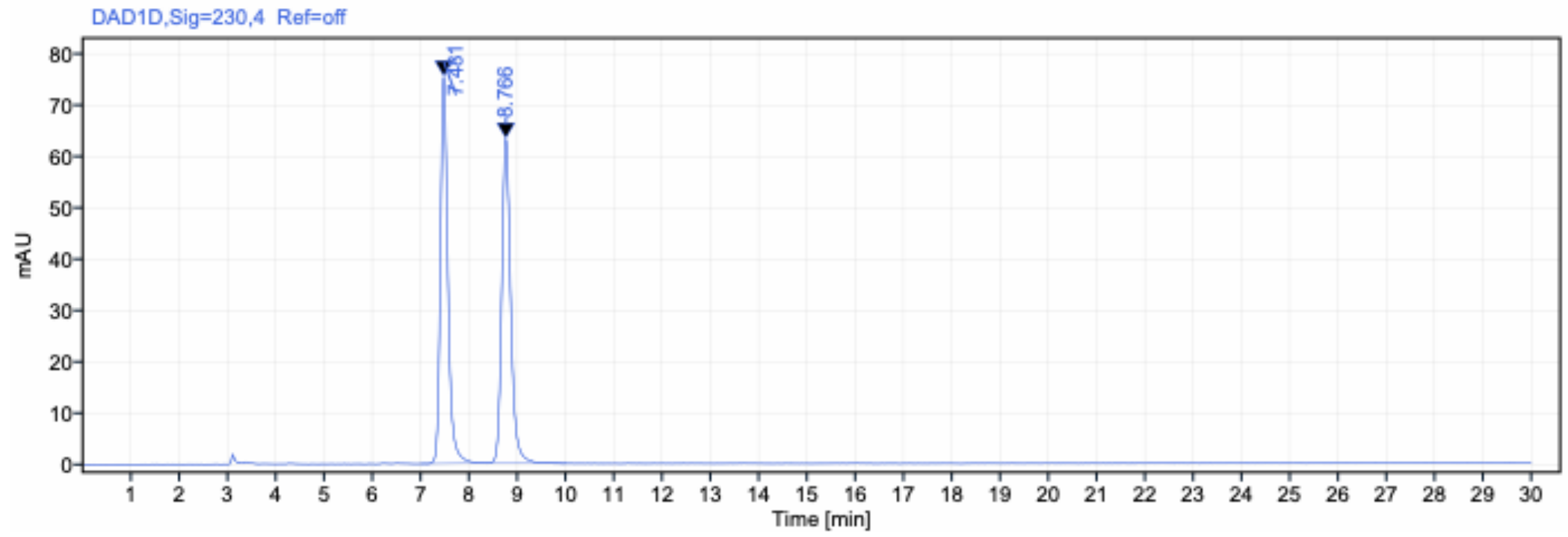

Signal: DAD1D,Sig=230,4 Ref=off

RT [min]	Type	Width [min]	Area	Height	Area%	Name
7.481	MM m	1.33	816.54	75.34	50.02	
8.766	MM m	1.69	815.86	63.17	49.98	

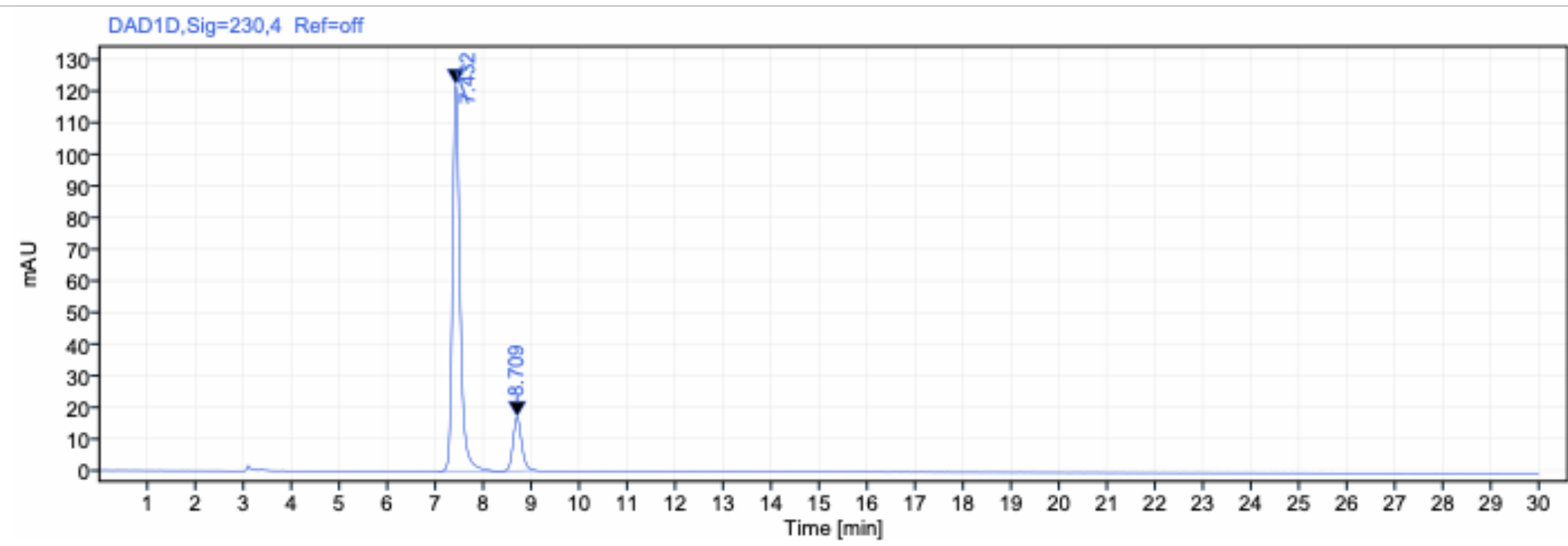

Signal: DAD1D,Sig=230,4 Ref=off

RT [min]	Type	Width [min]	Area	Height	Area%	Name
7.432	MM m	1.29	1324.01	122.08	86.26	
8.709	MM m	0.75	210.97	16.92	13.74	

1v and racemic standard (10% isopropyl alcohol in hexanes, AS-H, 20 min)

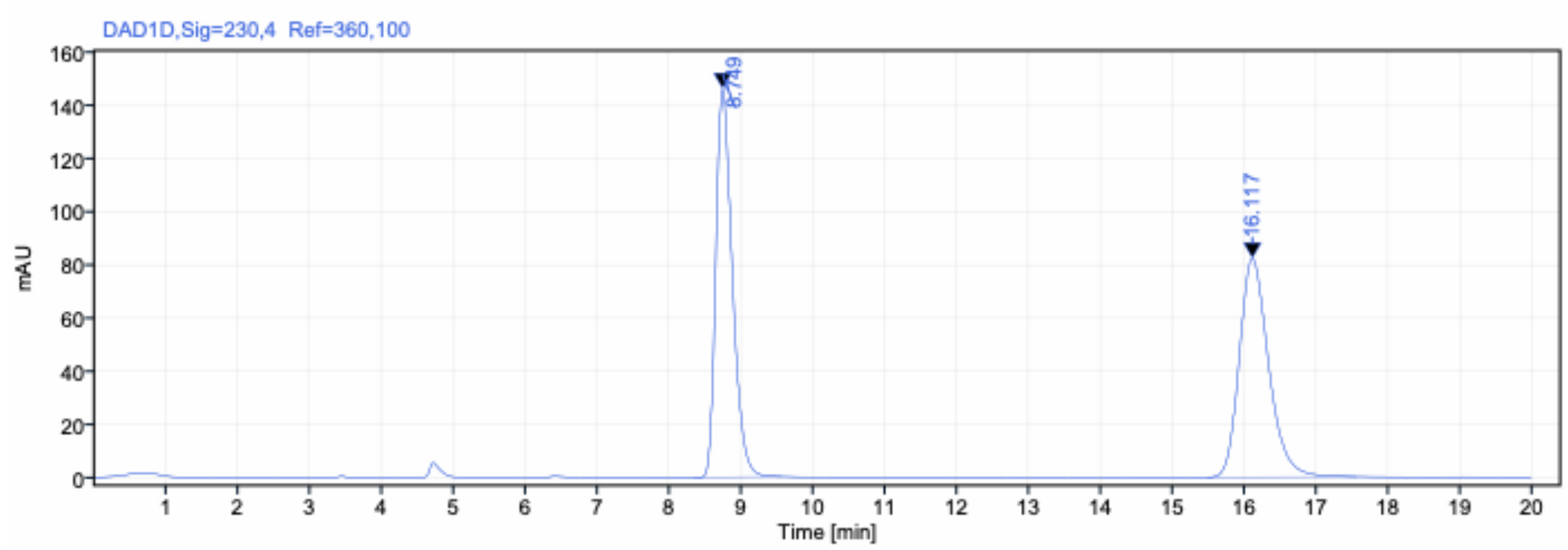

Signal: DAD1D,Sig=230,4 Ref=360,100

RT [min]	Type	Width [min]	Area	Height	Area%	Name
8.749	MM m	1.22	2326.85	145.88	50.03	
16.117	BB	2.08	2324.10	82.13	49.97	

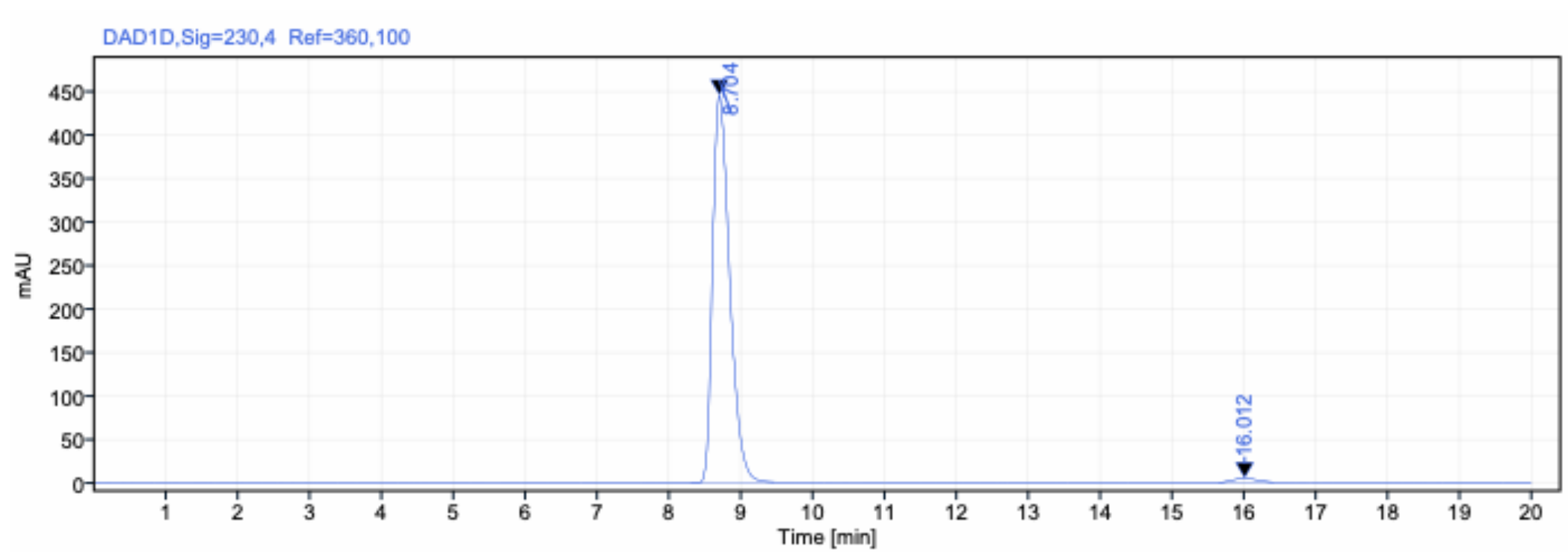

Signal: DAD1D,Sig=230,4 Ref=360,100

RT [min]	Type	Width [min]	Area	Height	Area%	Name
8.704	MM m	1.16	7356.63	444.83	98.57	
16.012	MM m	0.76	106.45	4.54	1.43	

1w and racemic standard (10% isopropyl alcohol in hexanes, AS-H, 30 min)

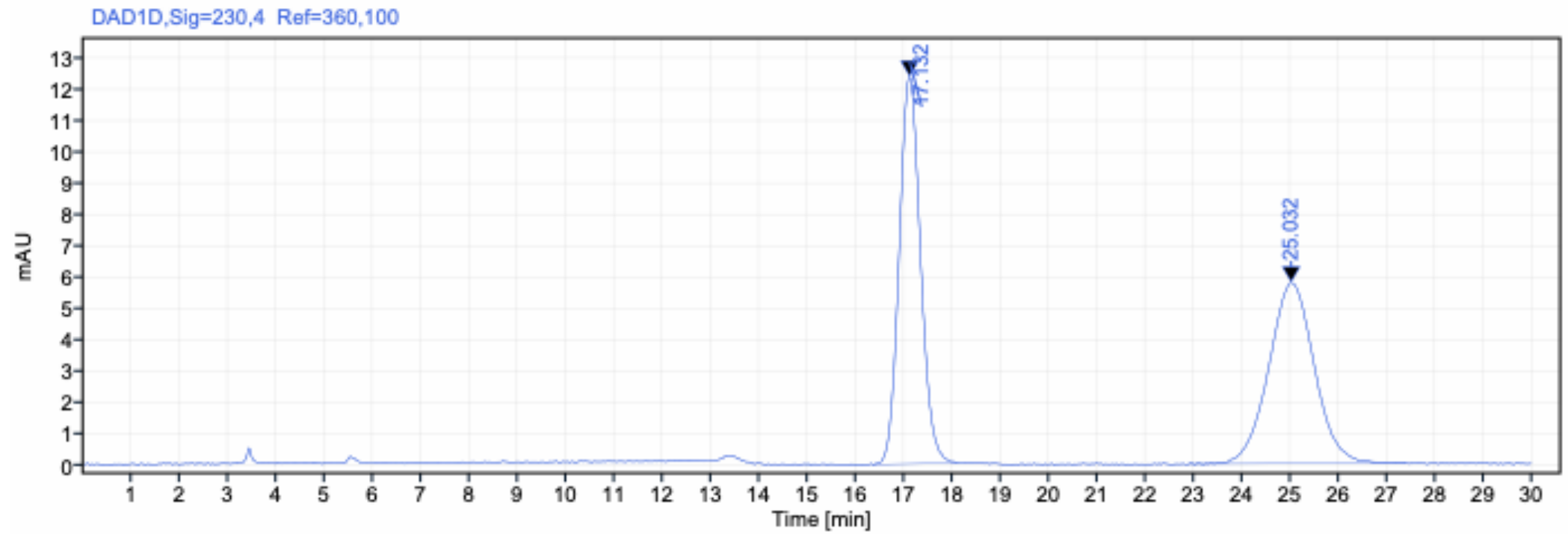

Signal: DAD1D,Sig=230,4 Ref=360,100

RT [min]	Type	Width [min]	Area	Height	Area%	Name
17.132	BB	1.77	377.94	12.36	50.07	
25.032	MM m	4.49	376.85	5.78	49.93	

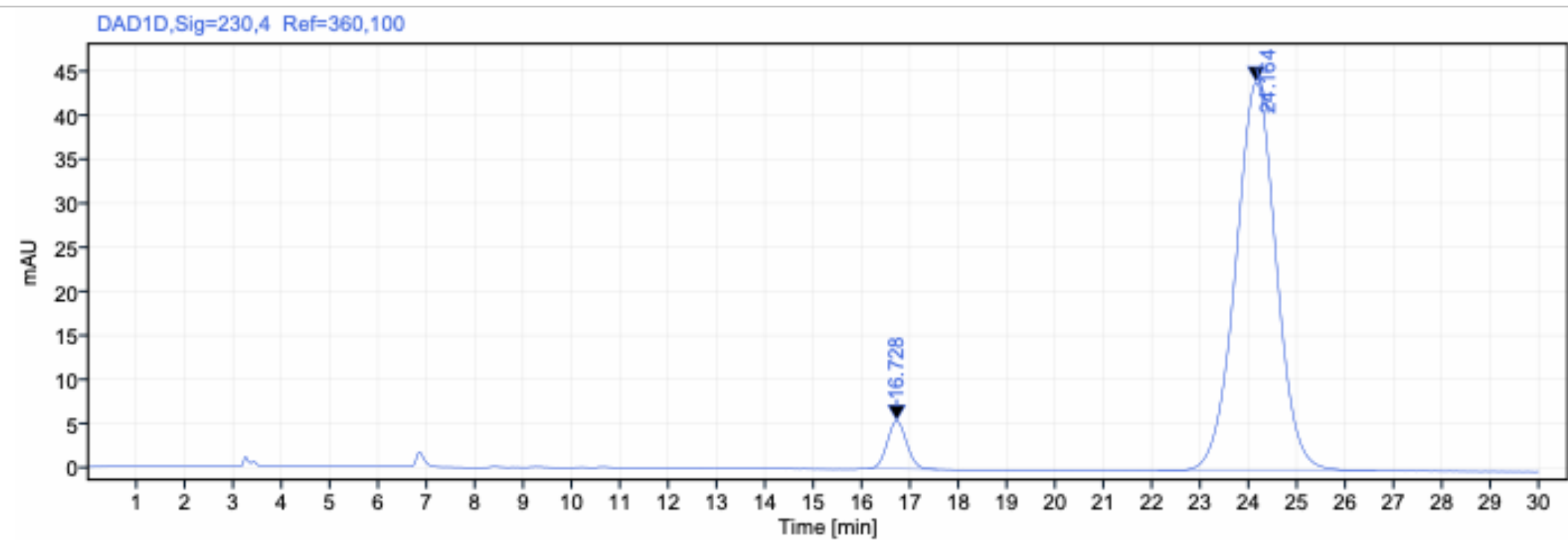

Signal: DAD1D,Sig=230,4 Ref=360,100

RT [min]	Type	Width [min]	Area	Height	Area%	Name
16.728	BB	1.44	151.41	5.43	5.59	
24.164	MM m	4.51	2557.37	44.04	94.41	

1x and racemic standard (5% isopropyl alcohol in hexanes, AD-H, 20 min)

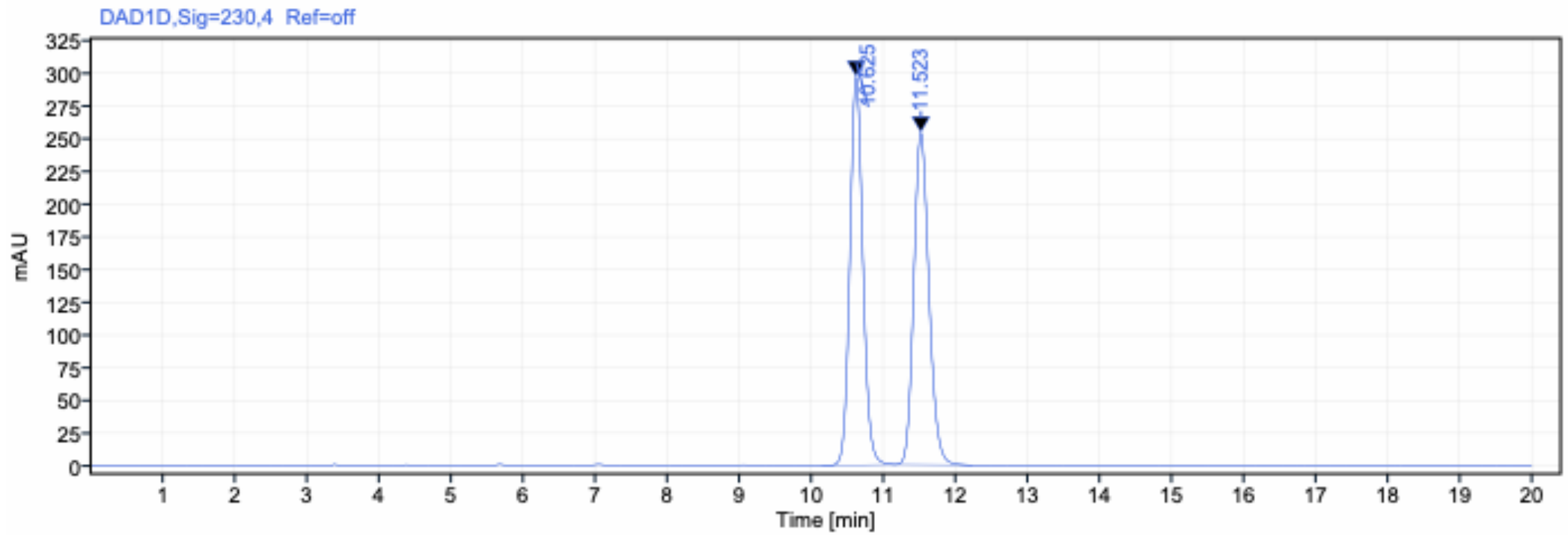

Signal: DAD1D,Sig=230,4 Ref=off

RT [min]	Type	Width [min]	Area	Height	Area%	Name
10.625	MM m	1.04	3719.93	296.54	50.03	
11.523	MM m	1.05	3715.43	253.60	49.97	

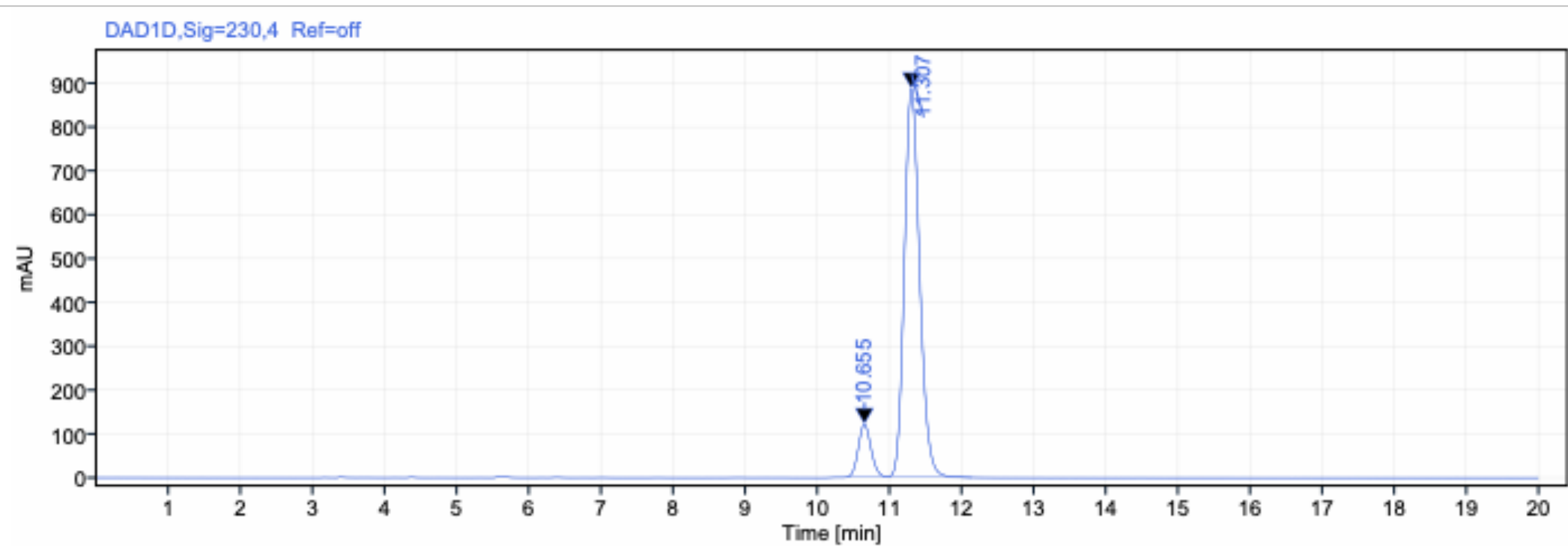

Signal: DAD1D,Sig=230,4 Ref=off

RT [min]	Type	Width [min]	Area	Height	Area%	Name
10.655	MM m	0.52	1452.61	119.73	10.02	
11.307	MM m	1.20	13037.35	885.16	89.98	

1y and racemic standard (10% isopropyl alcohol in hexanes, OJ-H, 20 min)

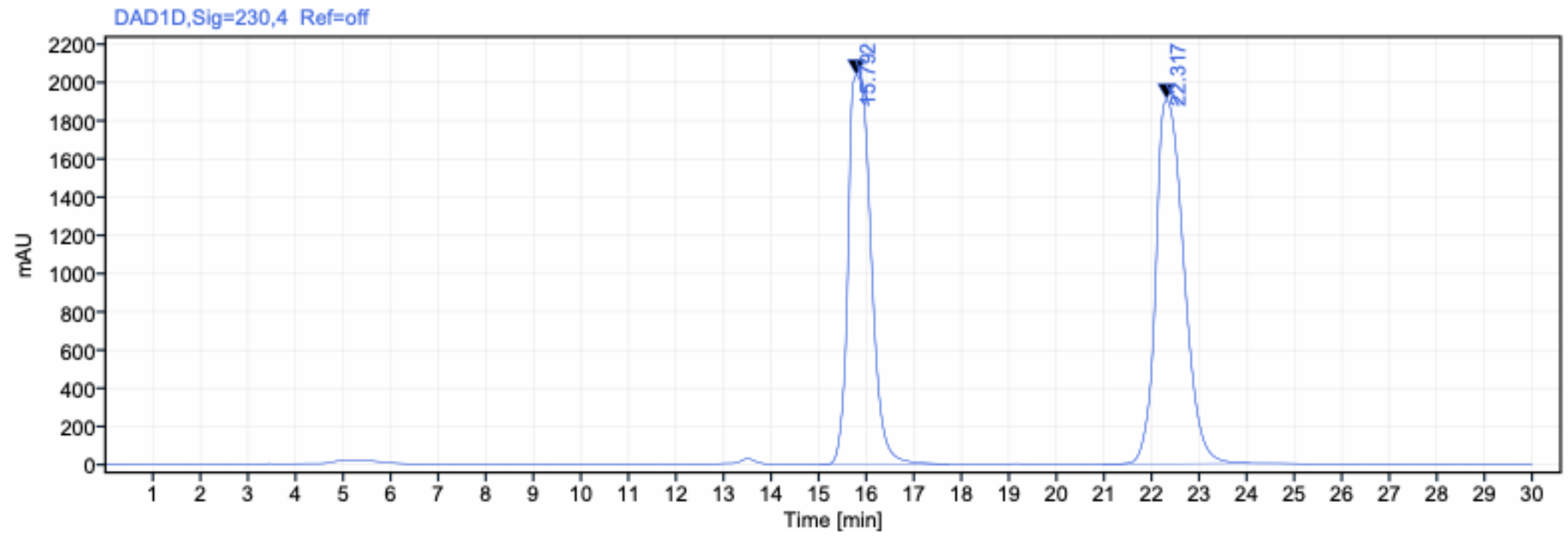

Signal: DAD1D,Sig=230,4 Ref=off

RT [min]	Type	Width [min]	Area	Height	Area%	Name
15.792	BB	2.73	67929.45	2033.34	46.56	
22.317	BBA	3.12	77974.08	1905.08	53.44	

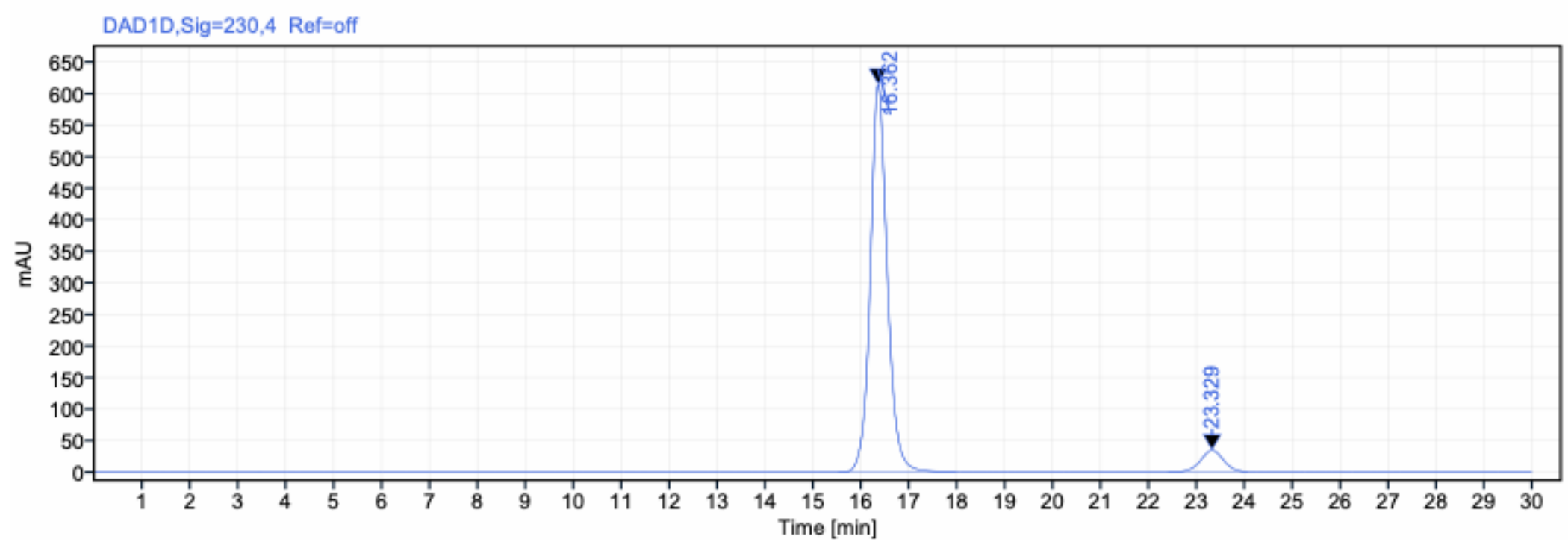

Signal: DAD1D,Sig=230,4 Ref=off

RT [min]	Type	Width [min]	Area	Height	Area%	Name
16.362	BB	2.45	15136.77	614.31	92.93	
23.329	BBA	1.66	1151.44	34.26	7.07	

1z and racemic standard (10% isopropyl alcohol in hexanes, AD-H, 30 min)

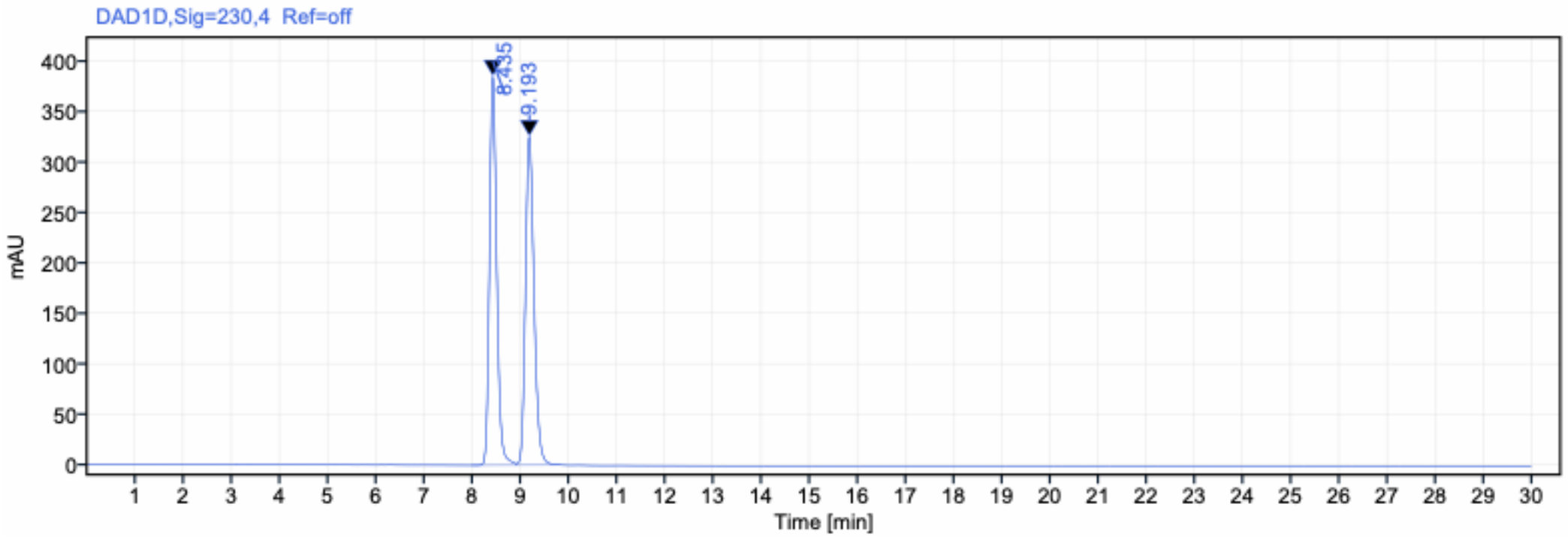

Signal: DAD1D,Sig=230,4 Ref=off

RT [min]	Type	Width [min]	Area	Height	Area%	Name
8.435	MM m	0.93	4047.99	385.60	50.10	
9.193	MM m	0.91	4031.33	325.80	49.90	

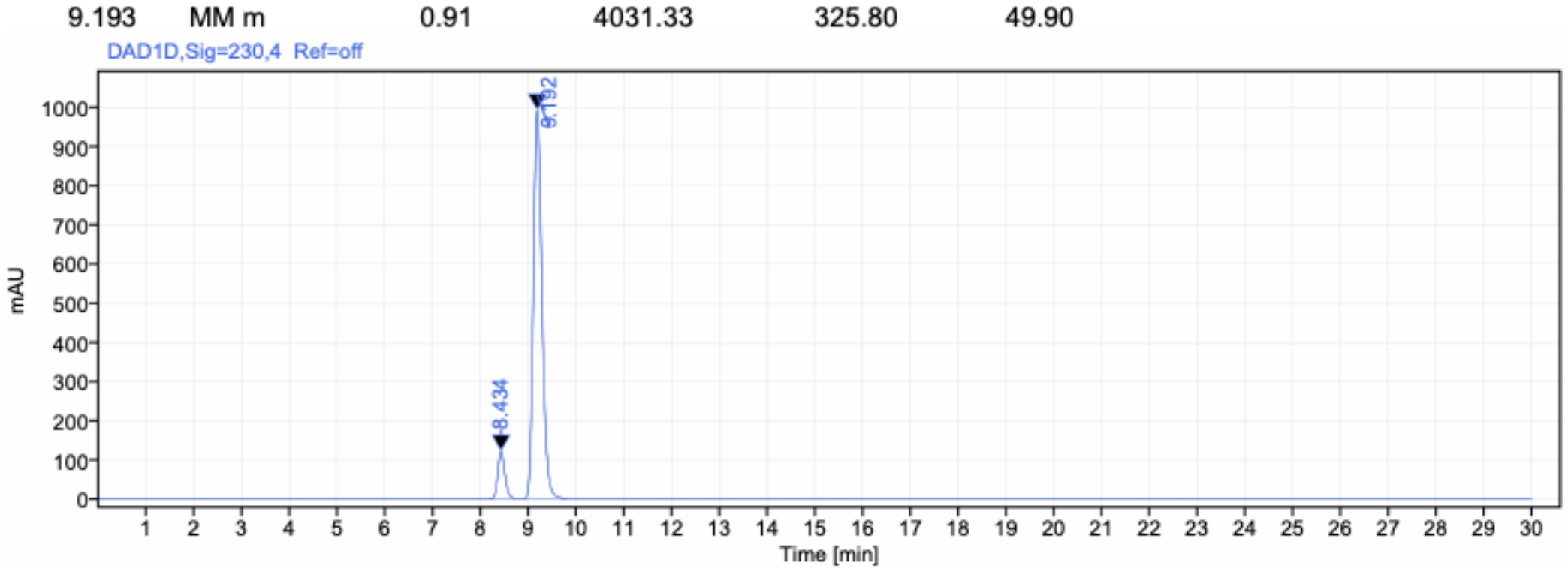

Signal: DAD1D,Sig=230,4 Ref=off

RT [min]	Type	Width [min]	Area	Height	Area%	Name
8.434	MM m	0.52	1234.80	121.73	9.08	
9.192	MM m	0.90	12369.00	992.23	90.92	

1aa and racemic standard (8% isopropyl alcohol in hexanes, AD-H, 20 min)

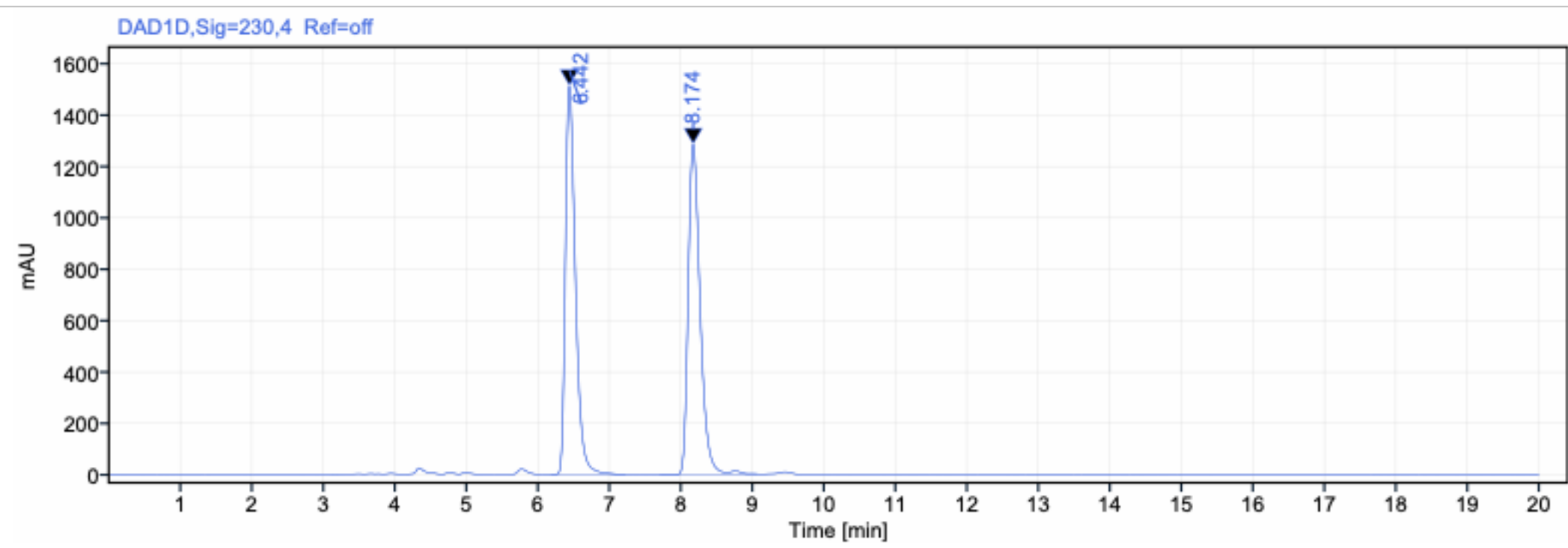

Signal: DAD1D,Sig=230,4 Ref=off

RT [min]	Type	Width [min]	Area	Height	Area%	Name
6.442	MM m	1.04	14700.30	1512.17	49.61	
8.174	MM m	1.35	14933.97	1284.62	50.39	

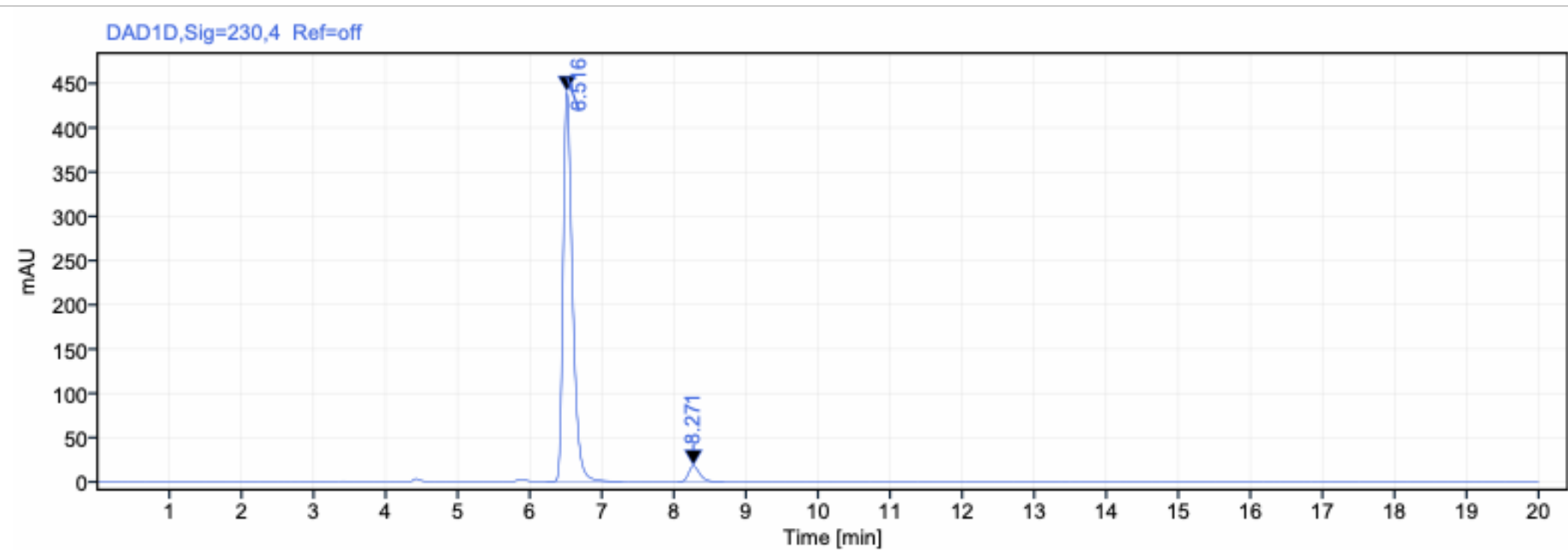

Signal: DAD1D,Sig=230,4 Ref=off

RT [min]	Type	Width [min]	Area	Height	Area%	Name
6.516	MM m	1.05	3972.78	439.85	95.48	
8.271	MM m	0.65	187.97	17.71	4.52	

1ab and racemic standard (5% isopropyl alcohol in hexanes, OD-H, 30 min)

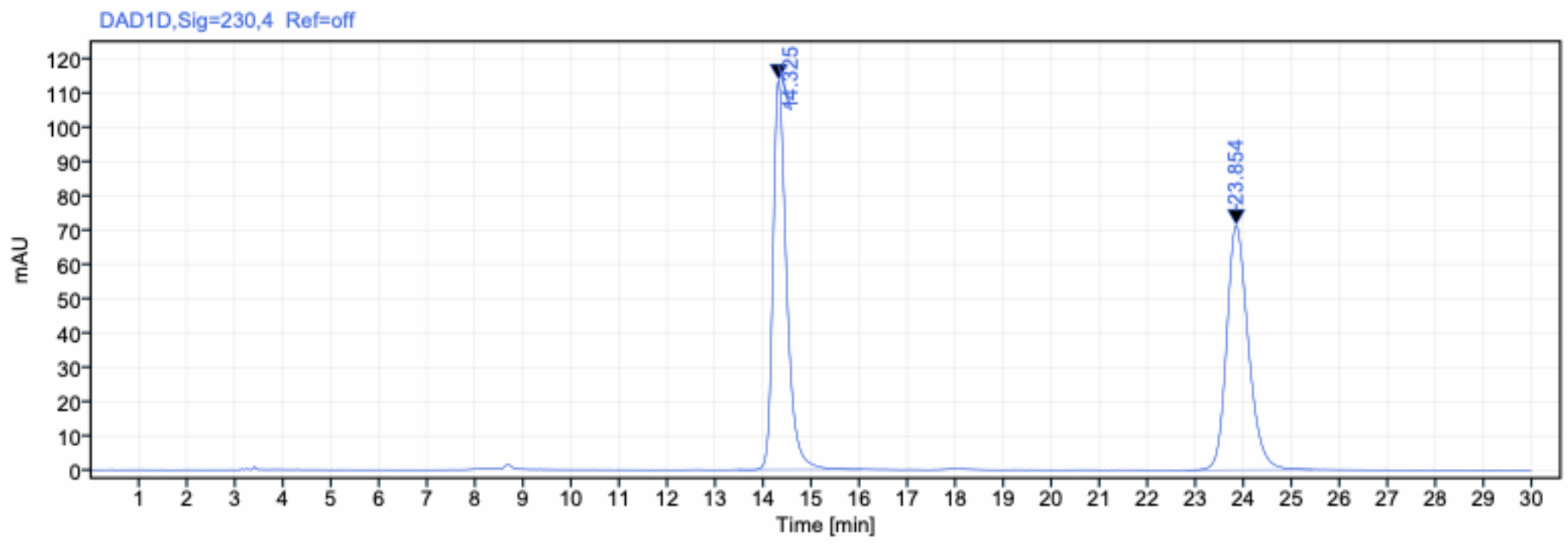

Signal: DAD1D,Sig=230,4 Ref=off

RT [min]	Type	Width [min]	Area	Height	Area%	Name
14.325	MM m	2.57	2232.41	113.57	50.05	
23.854	MM m	2.64	2227.75	71.29	49.95	

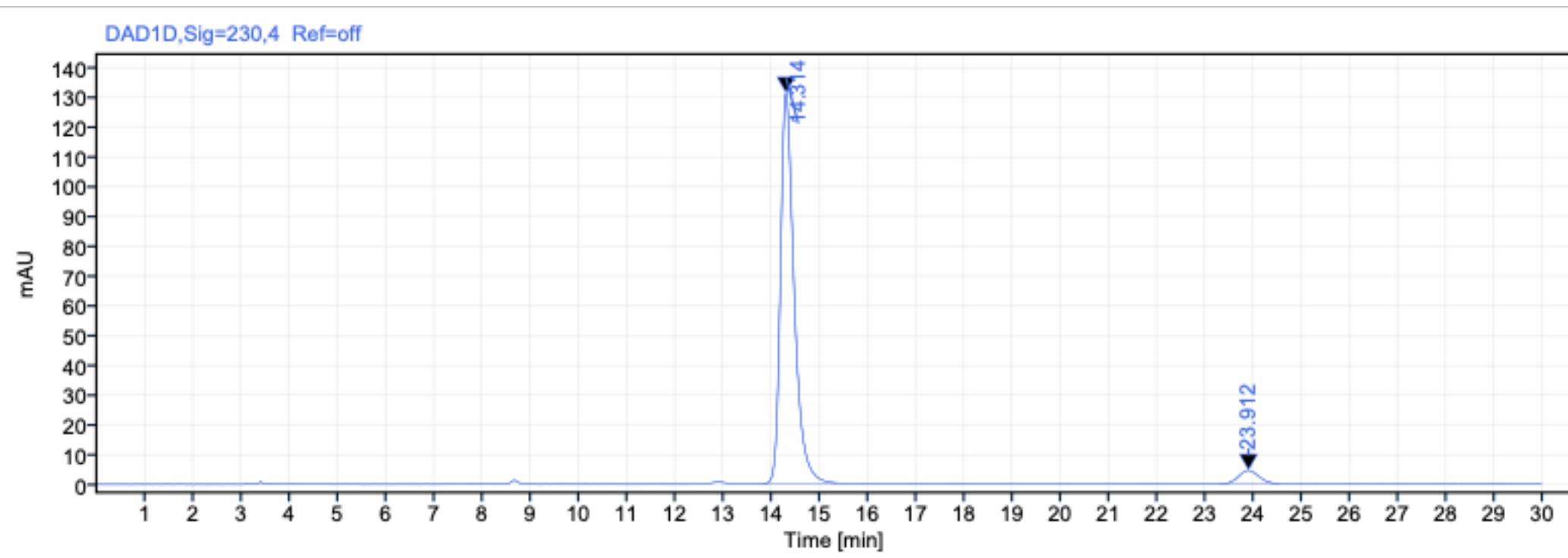

Signal: DAD1D,Sig=230,4 Ref=off

RT [min]	Type	Width [min]	Area	Height	Area%	Name
14.314	BB	1.52	2584.54	131.17	95.08	
23.912	MM m	1.24	133.78	4.58	4.92	

1ac and racemic standard (10% isopropyl alcohol in hexanes, OZ-H, 20 min)

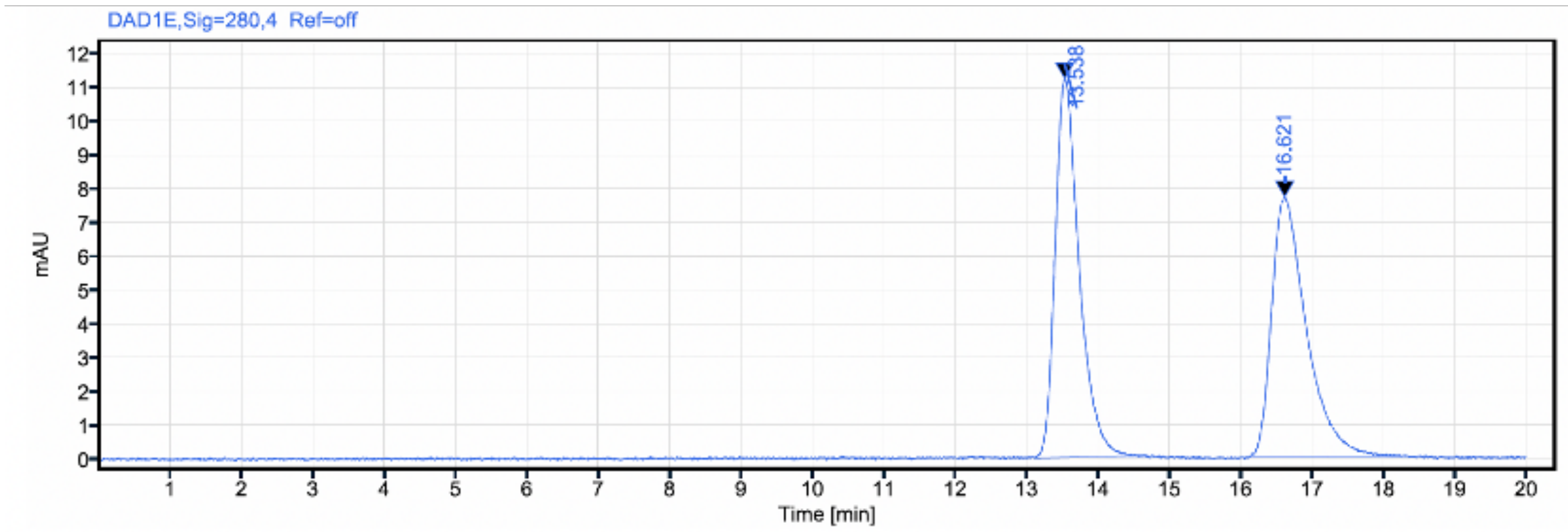

Signal: DAD1E,Sig=280,4 Ref=off

RT [min]	Type	Width [min]	Area	Height	Area%	Name
13.538	BM m	1.55	261.99	11.20	50.24	
16.621	MM m	2.12	259.47	7.67	49.76	
		**Sum**	**521.45**			

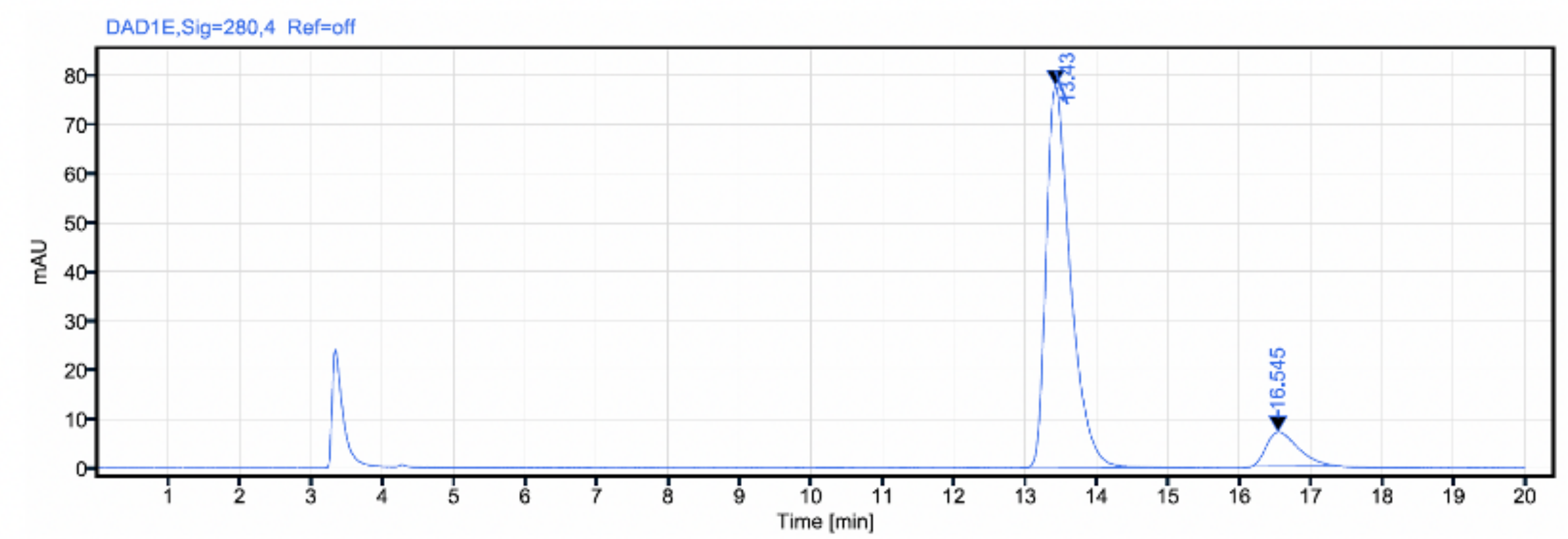

Signal: DAD1E,Sig=280,4 Ref=off

RT [min]	Type	Width [min]	Area	Height	Area%	Name
13.430	BM m	1.77	1844.07	77.78	89.69	
16.545	MM m	1.16	212.00	6.94	10.31	
		**Sum**	**2056.08**			

(2*S*)-**1ad** and racemic standard (5% isopropyl alcohol in hexanes, AS-H, 30 min)

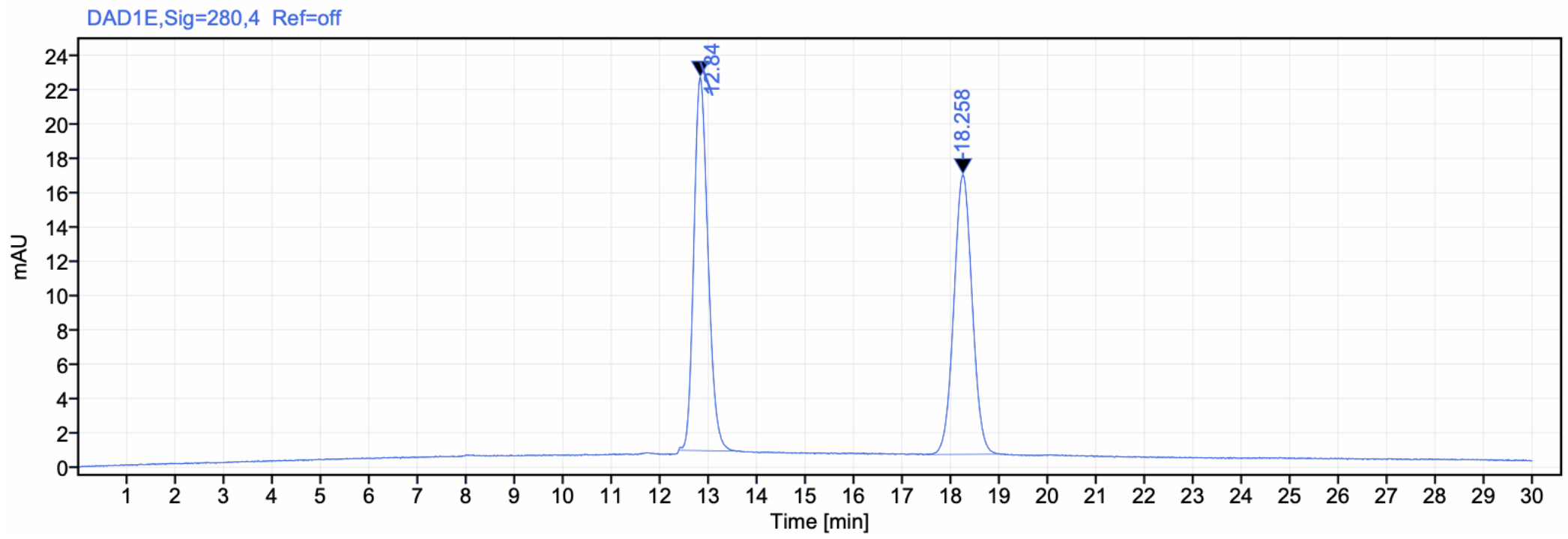

Signal: DAD1E,Sig=280,4 Ref=off

RT [min]	Type	Width [min]	Area	Height	Area%	Name
12.840	MB m	1.12	440.06	21.76	50.66	
18.258	MM m	1.51	428.62	16.30	49.34	

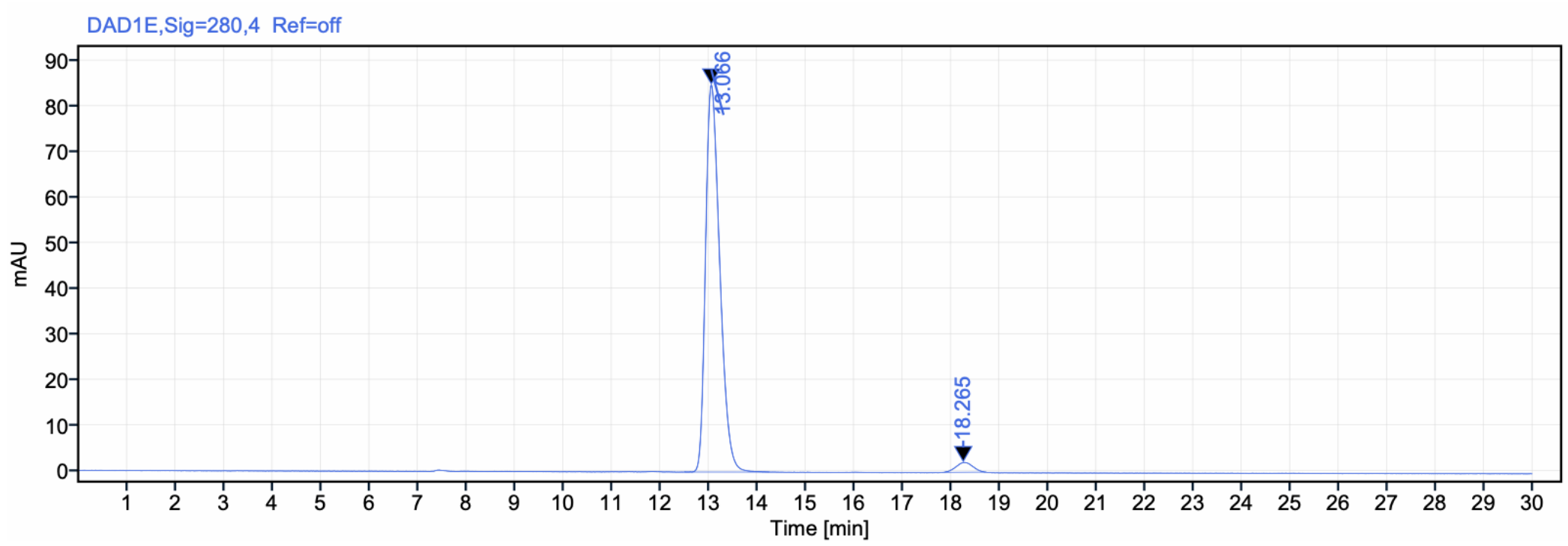

Signal: DAD1E,Sig=280,4 Ref=off

RT [min]	Type	Width [min]	Area	Height	Area%	Name
13.066	MM m	1.75	1735.69	84.93	97.11	
18.265	MM m	0.90	51.71	2.16	2.89	

(2*R*)**-1ad** and racemic standard (5% isopropyl alcohol in hexanes, AS-H, 30 min)

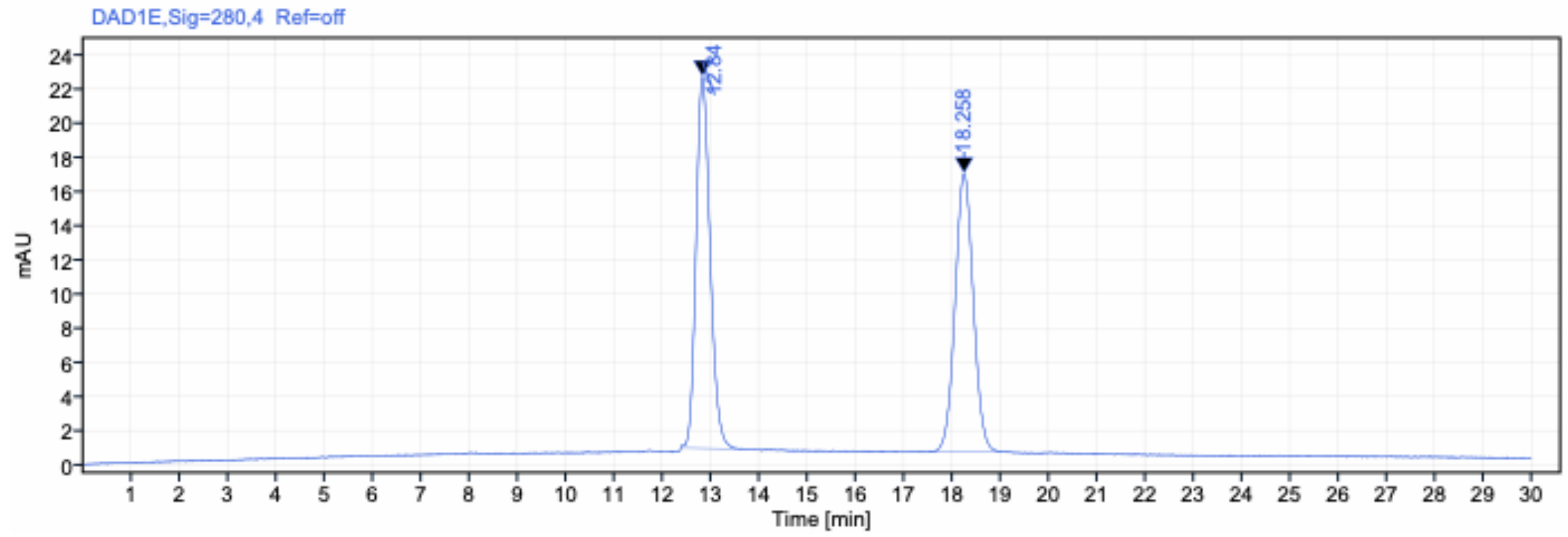

Signal: DAD1E,Sig=280,4 Ref=off

RT [min]	Type	Width [min]	Area	Height	Area%	Name
12.840	MB m	1.12	440.06	21.76	50.66	
18.258	MM m	1.51	428.62	16.30	49.34	

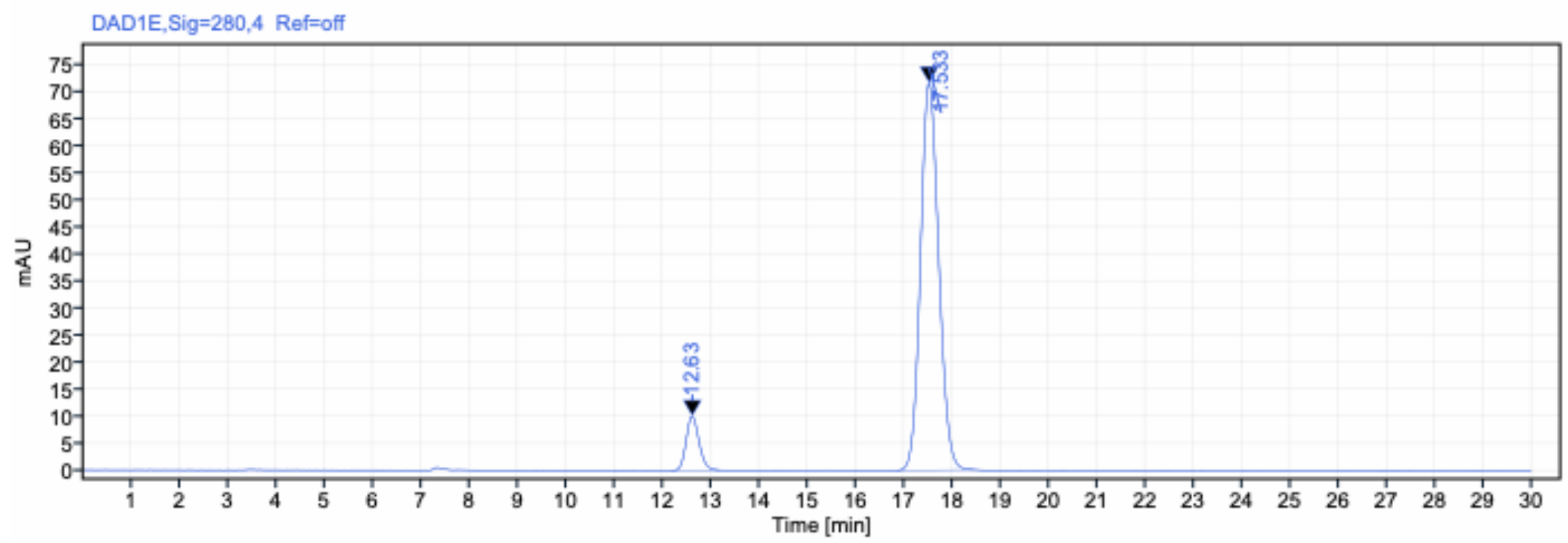

Signal: DAD1E,Sig=280,4 Ref=off

RT [min]	Type	Width [min]	Area	Height	Area%	Name
12.630	BB	0.99	186.31	10.03	9.02	
17.533	BB	1.61	1878.59	71.62	90.98	

1ae and racemic standard (5% isopropyl alcohol in hexanes, AS-H, 15 min)

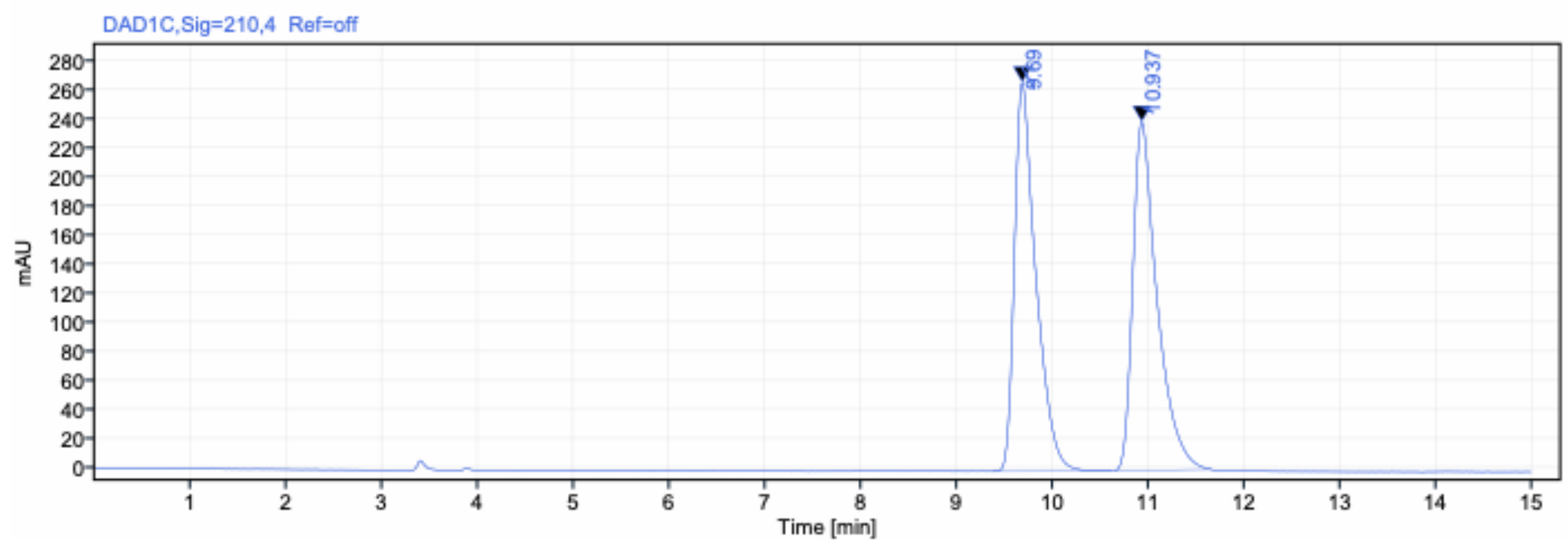

Signal: DAD1C,Sig=210,4 Ref=off

RT [min]	Type	Width [min]	Area	Height	Area%	Name
9.690	BM m	1.25	4167.55	267.60	49.61	
10.937	MM m	0.99	4233.01	239.71	50.39	

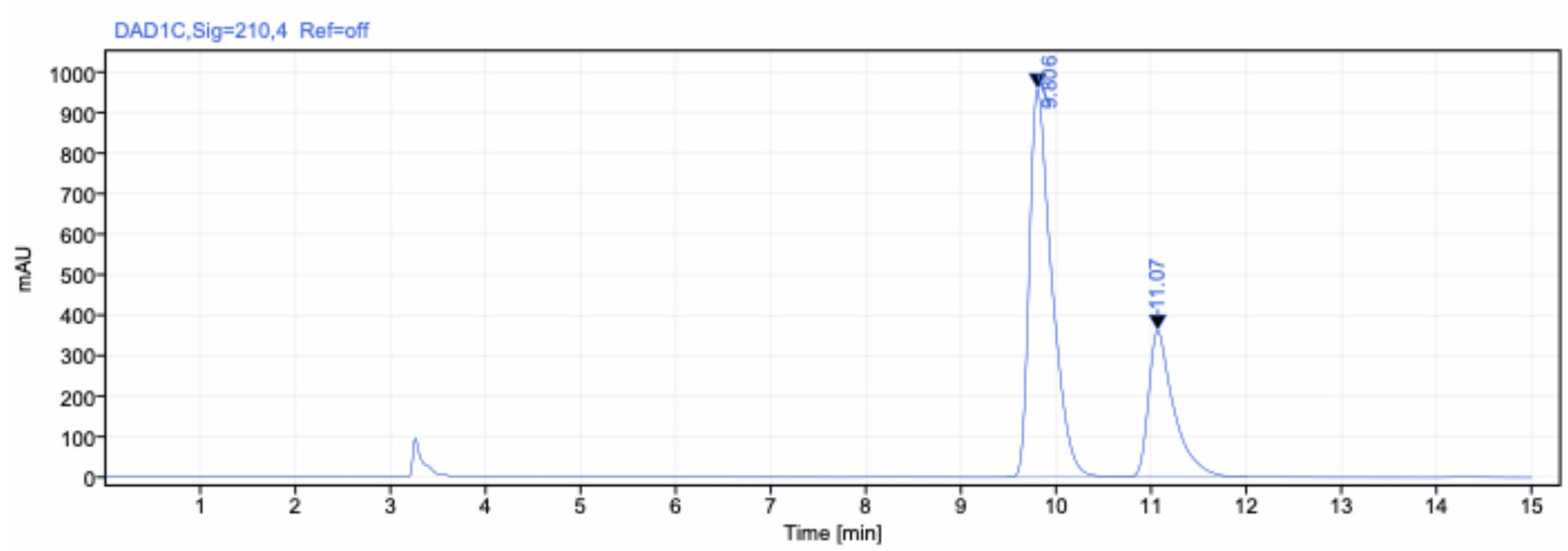

Signal: DAD1C,Sig=210,4 Ref=off

RT [min]	Type	Width [min]	Area	Height	Area%	Name
9.806	BB	1.25	15617.77	957.96	70.06	
11.070	BB R	1.27	6673.29	361.27	29.94	

1ae-d_1 and racemic standard (5% isopropyl alcohol in hexanes, AS-H, 15 min)

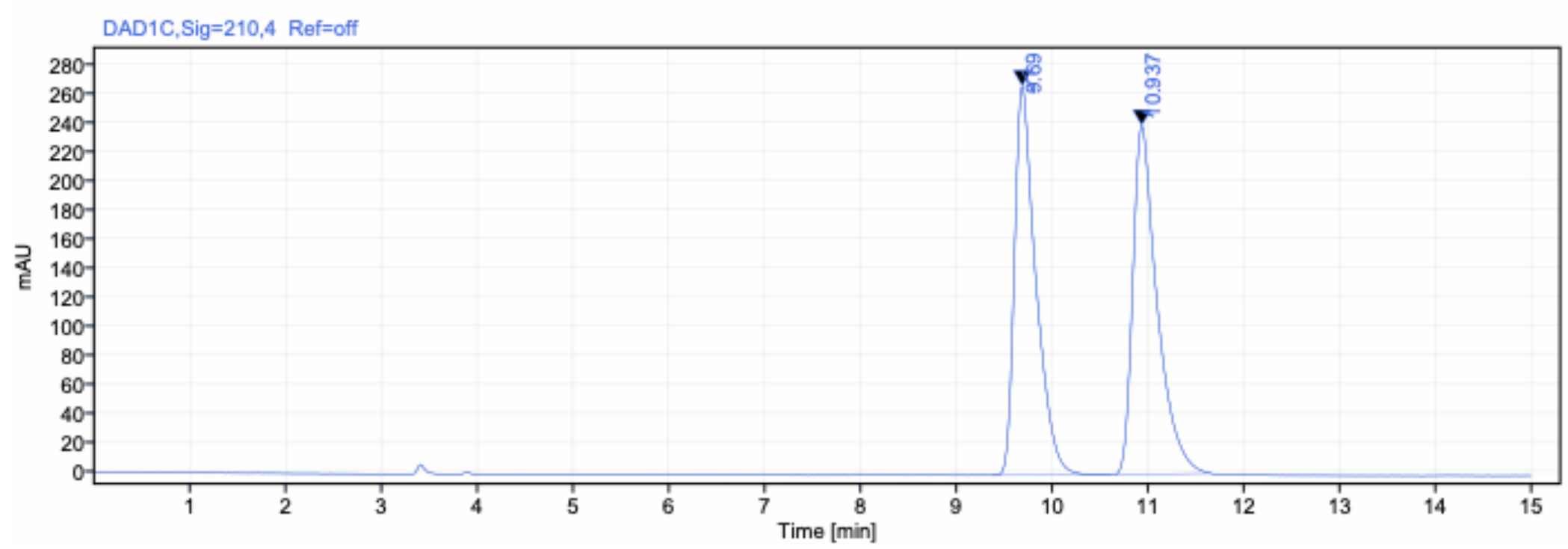

Signal: DAD1C,Sig=210,4 Ref=off

RT [min]	Type	Width [min]	Area	Height	Area%	Name
9.690	BM m	1.25	4167.55	267.60	49.61	
10.937	MM m	0.99	4233.01	239.71	50.39	

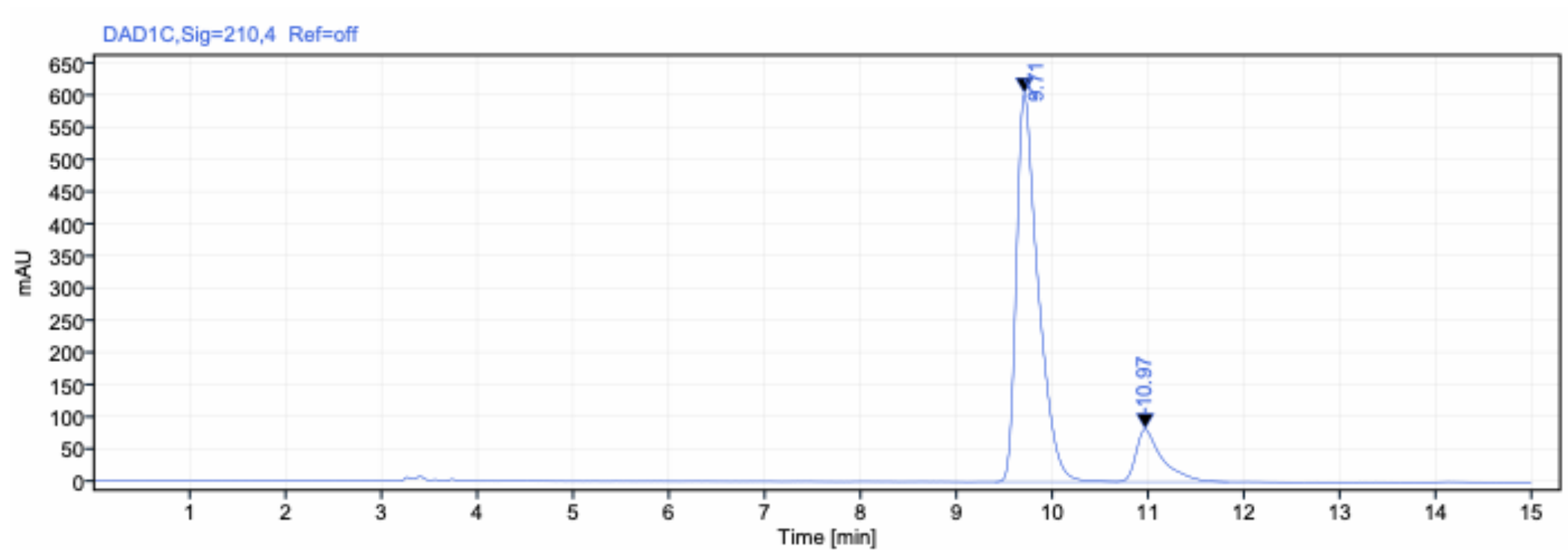

Signal: DAD1C,Sig=210,4 Ref=off

RT [min]	Type	Width [min]	Area	Height	Area%	Name
9.710	BV	1.26	9512.06	603.22	86.02	
10.970	VB R	1.17	1545.41	81.44	13.98	

6 and racemic standard (15% isopropyl alcohol in hexanes, OD-H, 20 min)

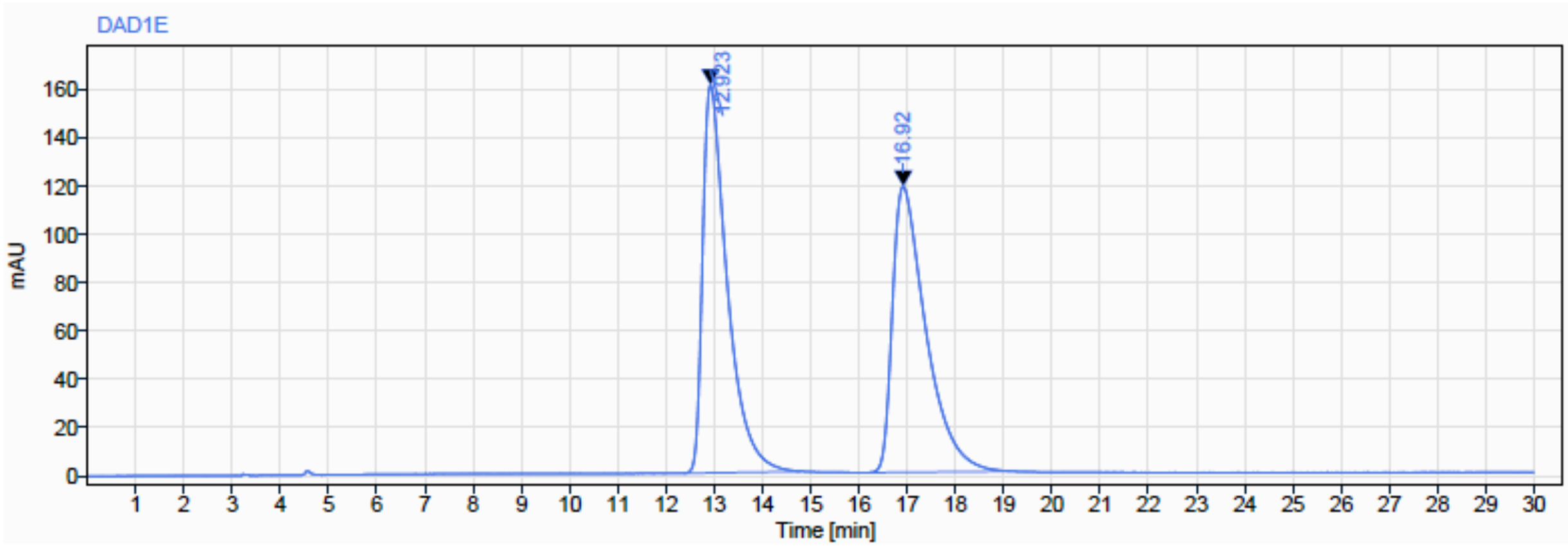

Signal:	DAD1E					
RT [min]	Type	Width [min]	Area	Height	Area%	Name
12.923	BB	2.45	5692.11	160.37	49.99	
16.920	BV	2.92	5693.53	118.18	50.01	
		**Sum**	**11385.63**			

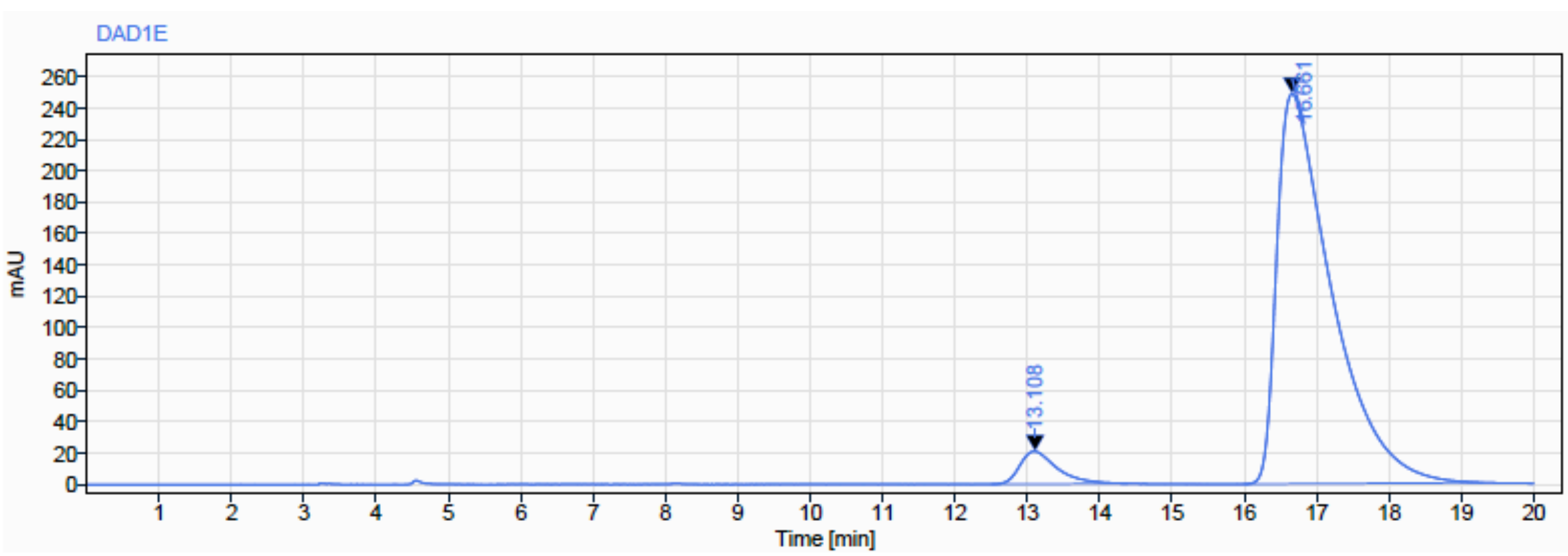

Signal:	DAD1E					
RT [min]	Type	Width [min]	Area	Height	Area%	Name
13.108	BB	1.88	748.12	20.85	5.41	
16.661	BB	3.31	13079.39	248.69	94.59	
		**Sum**	**13827.51**			

6. Determination of Photostationary State ee with Photocatalysts Ir-1–Ir-10

General Procedure

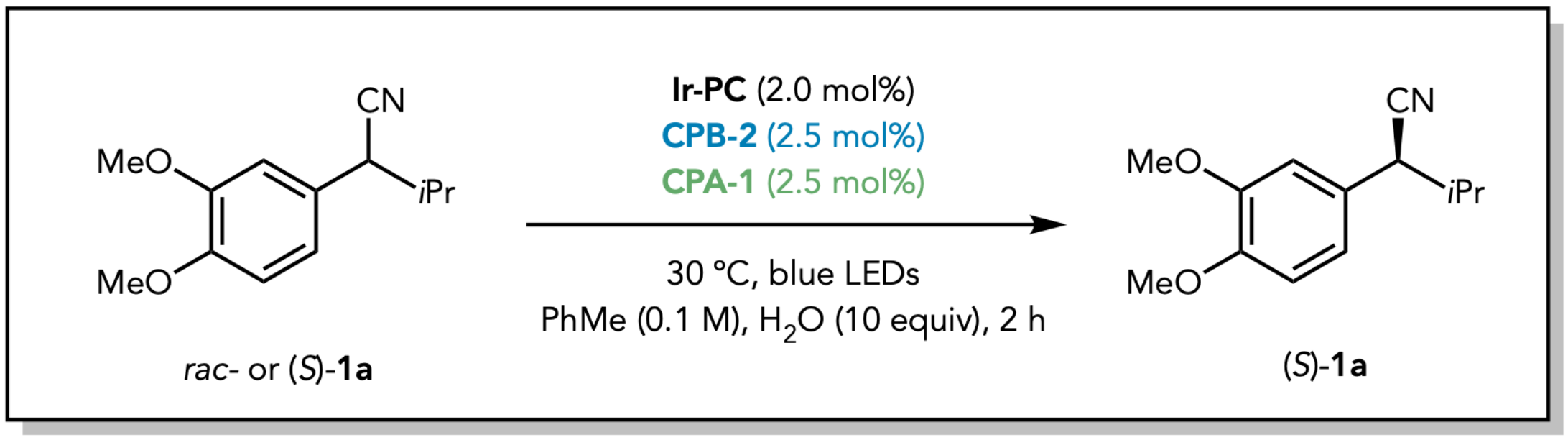

An oven-dried 2-dram vial equipped with a magnetic stir bar was charged with either racemic nitrile **1a** or enantiopure (*S*)-**1a** (0.05 mmol, 1.00 equiv), photocatalyst (1.0 µmol, 2.0 mol%), chiral phosphate base **CPB-2** (1.25 µmol, 2.50 mol%), and chiral phosphoric acid **CPA-1** (1.25 µmol, 2.50 mol%). The vial was imported into an N_2 atmosphere glovebox and charged with toluene (0.5 mL, 0.10 M). The vial was sealed with a Teflon septum cap and the junction between the cap and vial wrapped with vinyl electrical tape. The reaction mixture was exported from the glovebox and charged with degassed H_2O (10 µL, 10 equiv) via Hamilton syringe, then the top of the Teflon septum cap was taped over with vinyl electrical tape. The reaction was then placed in a photoreactor equipped with 456 nm Kessil lamps (PR160L) set to 100% intensity and irradiated for 2 h. After completion of irradiation, the reaction mixture was concentrated under reduced pressure. Piperonal (7.5 mg, 0.05 mmol, 1 equiv) was added as an internal NMR standard via $CDCl_3$ stock solution and an aliquot was removed for mass balance analysis by ^1H NMR spectroscopy. Another aliquot of the reaction was diluted in acetonitrile for optical purity analysis by 2D LC-SFC (**LC**: Agilent SB-C18 (1.8 µm particle size, 2.1 × 50 mm), 0–100% water/acetonitrile, 5.50 min, 1.0 mL/min flow rate, 210 nm detection, t_r **1a** = 3.12–3.25 min; collected onto the SFC; **SFC**: Daicel ChiralPak® AD-3 (3 µm particle size, 4.6 × 250 mm); 10% isopropyl alcohol /hexanes; 0.5 mL/min flow rate): t_R(major) = 6.11 min; t_R(minor) = 6.52 min).

ee_{PS} is determined as the average ee at the end of each reaction starting from *rac*-**1a** and (*S*)-**1a**.

Table S6.1. ee_{PS} of **1a** with varying photocatalysts

PC	$E_{1/2}(Ir^{III}/Ir^{II})$ (V vs Fc^+/Fc)	$E_{1/2}(^*Ir^{III}/Ir^{II})$ (V vs Fc^+/Fc)	from *rac*-**1a** Yield (%)	from *rac*-**1a** er	from *rac*-**1a** ee	from (*S*)-**1a** Yield (%)	from (*S*)-**1a** er	from (*S*)-**1a** ee	ee_{PS}
**Ir-1**	-1.11	1.43	96	95:5	90	91	97:3	94	92
**Ir-2**	-1.14	1.28	96	95.5:4.5	91	84	95.5:4.5	91	91
**Ir-3**	-1.19	1.10	92	93:7	86	97	95:5	90	88
**Ir-4**	-1.28	1.28	85	90:10	80	83	93:7	88	84
**Ir-5**	-1.30	1.19	81	88:12	76	87	87:13	74	75
**Ir-6**	-1.38	0.85	84	91:9	82	83	88:12	76	78
**Ir-7**	-1.48	1.09	84	85:15	70	83	86:14	72	71
**Ir-8**	-1.56	1.21	87	86:14	72	77	85:15	70	71
**Ir-9**	-1.64	1.04	83	84:16	68	86	86:14	70	70
**Ir-10**	-1.77	0.98	87	85:15	70	83	85:15	70	70

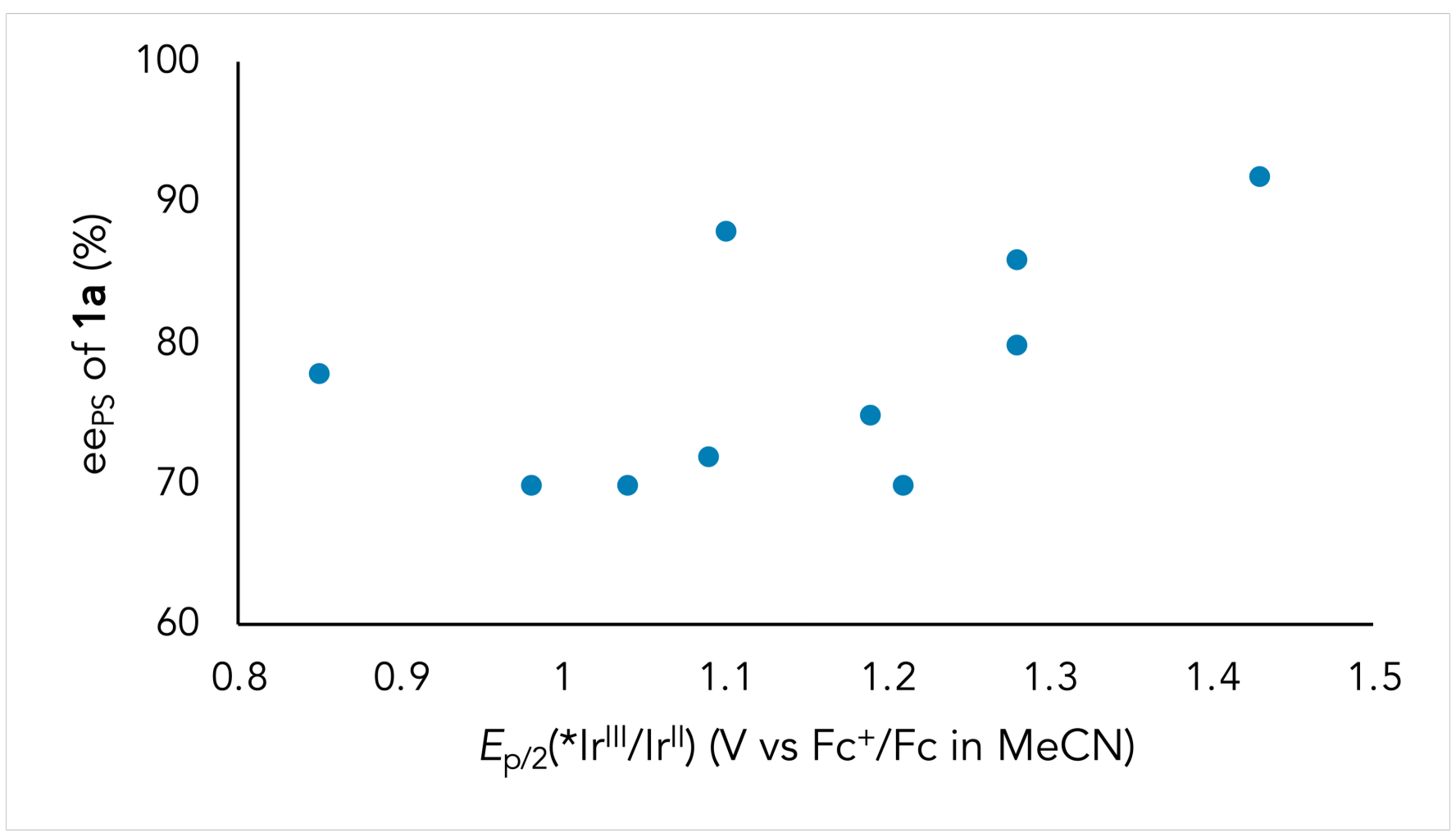

Figure S6.1. ee_{PS} of **1a** plotted against excited-state reduction potential of photocatalysts.

7. Synthesis of Mechanistic Probe 2

5 3,4-dimethoxybenzyl 2,2,2-trichloroacetimidate

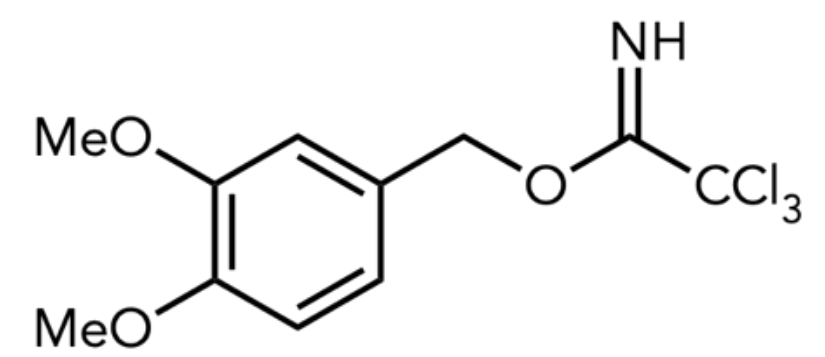

5

A flame-dried 100 mL round-bottom flask equipped with a magnetic stir bar was charged with (3,4-dimethoxyphenyl)methanol (5.0 g, 30 mmol, 1.0 equiv). The flask was evacuated and backfilled with N_2 three times, charged with CH_2Cl_2 (65 mL, 0.4 M solution), and cooled to 0 ºC in an ice bath. 1,8-Diazabicycloundec-7-ene (DBU) (890 µL, 6.0 mmol, 20 mol%) and trichloroacetonitrile (4.5 mL, 45 mmol, 1.5 equiv) were added sequentially in portions, and the reaction stirred for 1 h, monitoring by TLC (1:4 EtOAc/hexanes). Upon completion, the crude reaction mixture was filtered through a short silica plug and concentrated under reduce pressure to afford the title compound as a light yellow oil in 99% yield (9.2 g, 29 mmol).

Characterization data were consistent with those reported in the literature.[10]

2 2-((3,4-dimethoxybenzyl)oxy)-3-methyl-2-phenylbutanenitrile

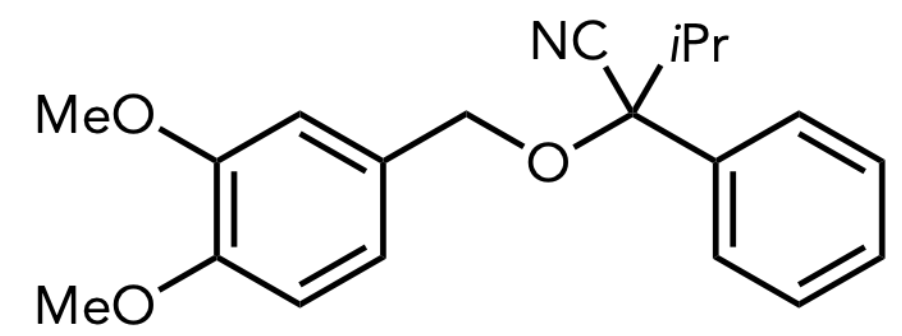

2

A flame-dried 100 mL round-bottom flask was charged with **5** (9.2 g, 29 mmol, 1.0 equiv) and a stir bar and placed under an N_2 atmosphere. The reactant was dissolved in CH_2Cl_2 (20 mL, 1.2 M) and cooled to 0 ºC in an ice bath. In a separate 2-dram vial was prepared a solution of pyridinium *p*-toluenesulfonate (PPTS, 370 mg, 1.5 mmol, 5.0 mol%) in CH_2Cl_2 (4 mL). The solution of PPTS was added as a single portion, followed by dropwise addition of mandelonitrile (3.5 mL, 29 mmol, 1.0 equiv). The reaction was stirred overnight, warming to room temperature. Upon completion, the reaction was quenched by the addition of 15% aqueous NaOH solution and transferred to a separatory funnel. The organic layer was washed with 15% aqueous NaOH solution (2 × 25 mL) and brine (1 × 25 mL), dried over Na_2SO_4, and concentrated *in vacuo*. The crude residue was purified by silica gel chromatography (0–40% EtOAc/hexanes) to afford 2-((3,4-dimethoxybenzyl)oxy)-2-phenylacetonitrile as a yellow oil in 24% yield (2.1 g, 7.3 mmol). The material was carried forward in the subsequent step without further purification.

A flame-dried 100 mL round-bottom flask was imported into an N_2 atmosphere glovebox and charged with potassium bis(trimethylsilyl)amide (700 mg, 3.5 mmol, 1.1 equiv). The flask was sealed with a rubber septum and exported from the glovebox. The reaction was placed under an N_2 atmosphere, dissolved in THF (30 mL, 0.1 M), and cooled to –78 ºC. In separate scintillation vials were prepared solutions of 18-crown-6 (1.3 g, 4.8 mmol, 1.5 equiv) and **5** (900 mg, 3.2 mmol, 1.0 equiv) in THF. The solution of 18-crown-6 was added to the reaction mixture as a single portion, followed by dropwise addition of the solution of **5**. After stirring for 30 minutes at –78 ºC, 2-iodopropane (0.64 mL, 6.4 mmol, 2.0 equiv) was added as a single portion. The reaction was stirred for 2 h, warming to room temperature. Upon completion, the reaction was quenched by the addition of saturated aqueous $NaHCO_3$. The reaction mixture was transferred to a separatory funnel and the aqueous phase extracted with EtOAc (3 × 20 mL). The combined organic layers were washed with brine (1 × 25 mL), dried over Na_2SO_4, filtered, and concentrated *in vacuo*. The crude residue was purified by silica gel chromatography (030% EtOAc/hexanes) to afford the title compound as an off-white solid in 38% yield (400 mg, 1.2 mmol).

^{1}H NMR (500 MHz, $CDCl_3$) δ 7.57 – 7.53 (m, 2H), 7.48 – 7.39 (m, 3H), 6.86 – 6.81 (m, 3H), 4.53 (d, *J* = 10.5 Hz, 1H), 4.14 (d, *J* = 10.4 Hz, 1H), 3.88 (s, 3H), 3.88 (s, 3H), 2.26 (hept, *J* = 6.8 Hz, 1H), 1.27 (d, *J* = 6.5 Hz, 3H), 0.81 (d, *J* = 6.8 Hz, 3H). **^{13}C NMR** (126 MHz, $CDCl_3$) δ 149.07, 148.97, 136.86, 129.76, 129.31, 128.89, 126.66, 120.56, 117.69, 111.43, 111.13, 86.72, 69.11, 56.07, 55.99, 40.07, 18.10, 17.74. **HRMS**: m/z $[M+Na]^+$ calcd. for $C_{20}H_{23}NO_3Na$: 348.15707, found: 348.15737 (+0.87 ppm). **IR**: 2875.57, 2155.80, 2037.29, 1982.98, 1980.39, 1594.57, 1517.73, 1491.84, 1467.79, 1448.82, 1418.12, 1381.19, 1261.89, 1239.65, 1211.80, 1160.25, 1140.75, 1055.78, 1039.83, 1024.62, 1000.89, 974.94, 936.75, 915.66, 854.17, 811.42, 776.46, 760.00, 750.56, 699.77, 655.10, 609.84, 556.89, 523.23, 457.75, 439.17, 430.13, 412.19, 410.08, 407.06.

8. Control Experiments with Nitrile 3

General Procedure

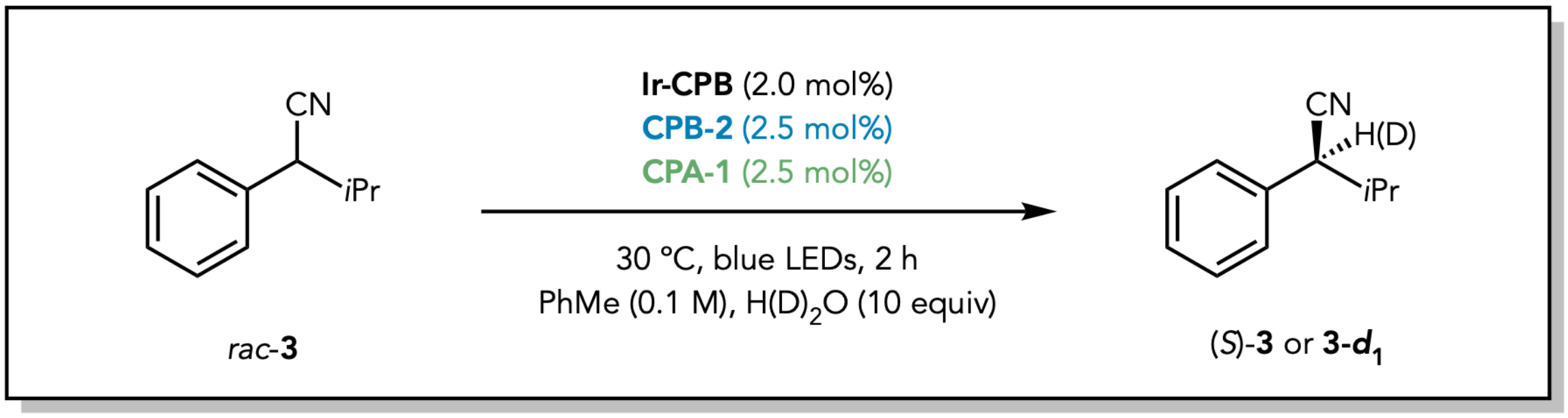

An oven-dried 2-dram vial equipped with a magnetic stir bar was charged with **3** (0.05 mmol, 8.0 mg, 1.00 equiv), photocatalyst (1.0 µmol, 2.0 mol%), chiral phosphate base **CPB-2** (1.25 µmol, 2.50 mol%), and chiral phosphoric acid **CPA-1** (1.25 µmol, 2.50 mol%). The vial was imported into an N_2 atmosphere glovebox and charged with toluene (0.5 mL, 0.1 M). The vial was sealed with a Teflon septum cap and the junction between the cap and vial wrapped with vinyl electrical tape. The reaction mixture was exported from the glovebox and charged with degassed H_2O or D_2O (10 µL, 10 equiv) via Hamilton syringe, then the top of the Teflon septum cap was taped over with vinyl electrical tape. The reaction was then placed in a photoreactor equipped with 456 nm Kessil lamps (PR160L) set to 100% intensity, and irradiated for 2 h. After completion of irradiation, the reaction mixture was concentrated under reduced pressure. Piperonal (7.5 mg, 0.05 mmol, 1.0 equiv) was added as an internal NMR standard via $CDCl_3$ stock solution and an aliquot was removed for mass balance analysis by ^1H NMR spectroscopy. Another aliquot of the reaction was purified by preparative TLC (15% EtOAc/hexanes) and analyzed by chiral HPLC (ChiralCel OJ-H, 10% isopropyl alcohol, 10 min, 210 nm detection) t_R = 6.7 and t_R = 7.6 min.

No deuterium incorporation or change in enantiomeric ratio was observed.

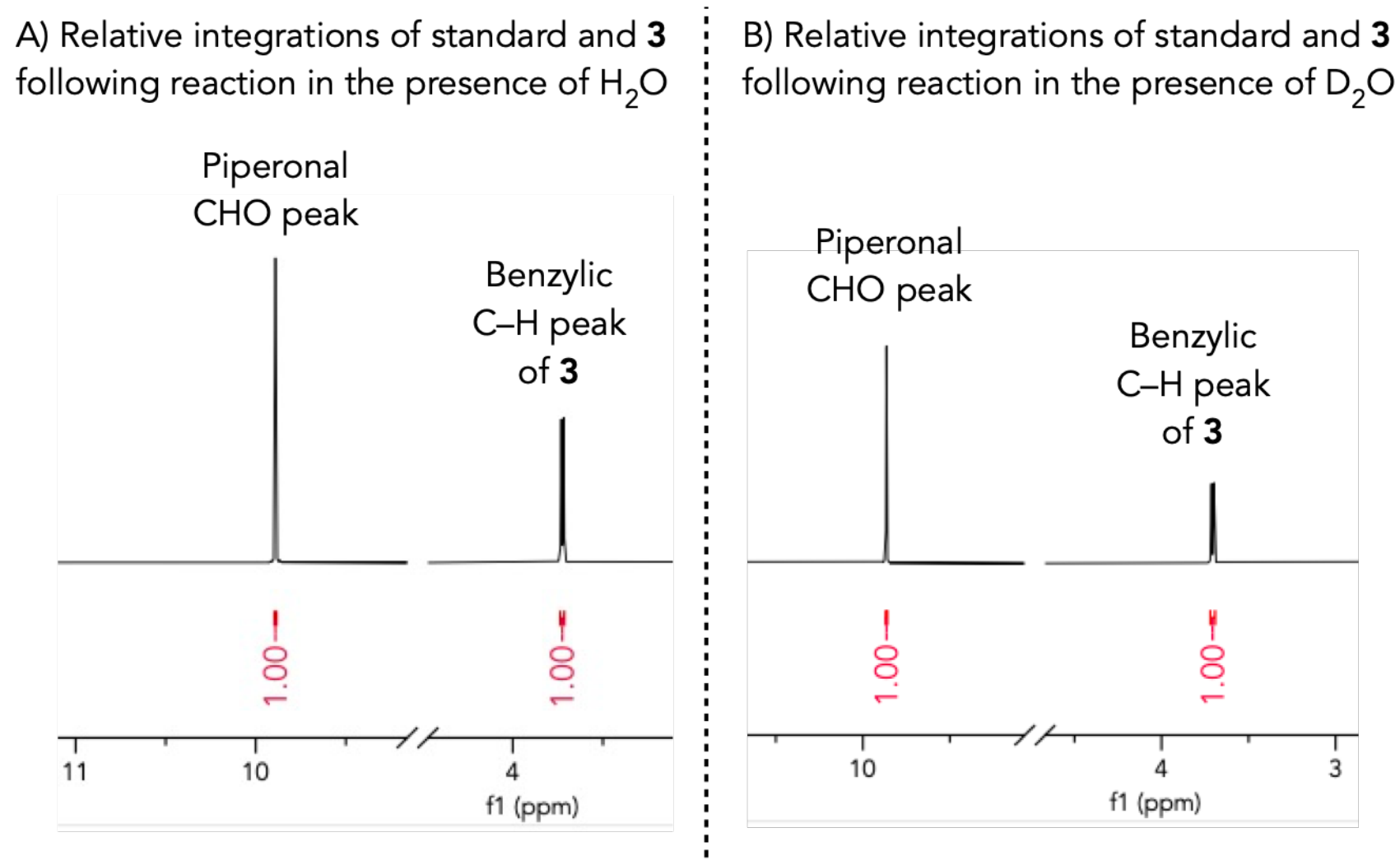

Figure S8.1 A) Relative integrations of piperonal internal standard and benzylic C–H peak of **3** under reaction conditions with H_2O. B) Relative integrations of piperonal internal standard and benzylic C–H peak of **3** under reaction conditions with D_2O. No deuterium incorporation observed.

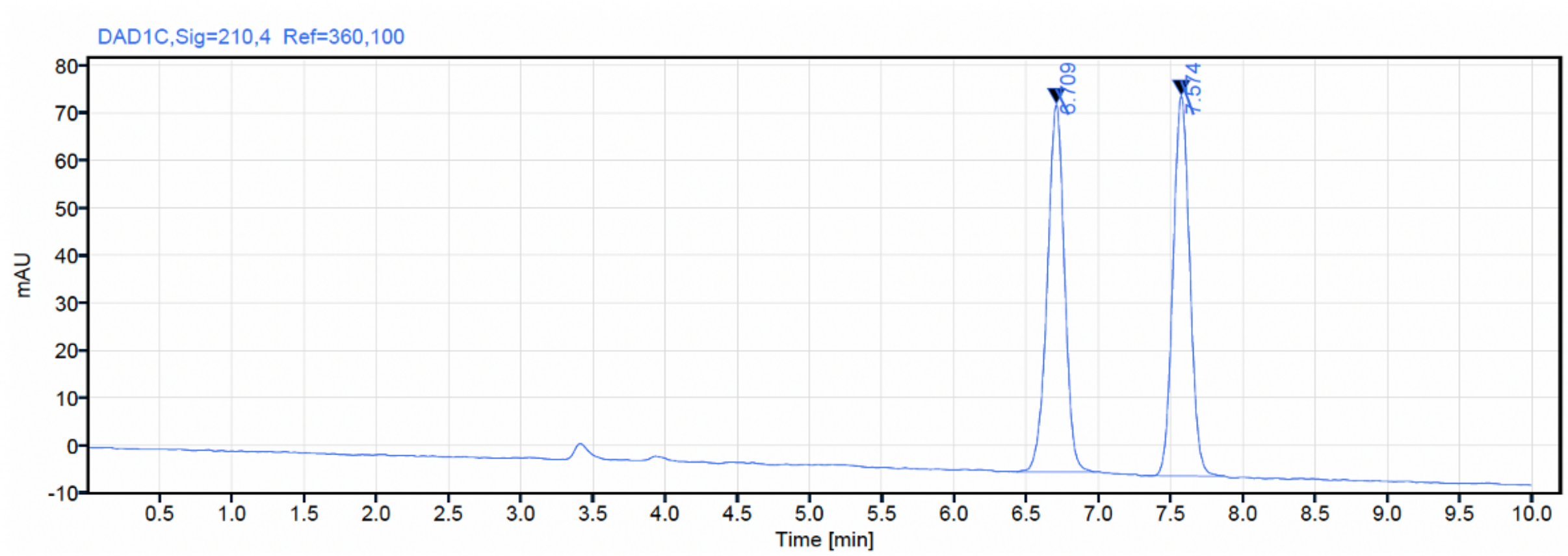

Signal: DAD1C,Sig=210,4 Ref=360,100

RT [min]	Type	Width [min]	Area	Height	Area%	Name
6.709	MM m	0.54	640.69	77.33	49.92	
7.574	MM m	0.56	642.66	79.95	50.08	
		**Sum**	**1283.35**			

Figure S8.2 HPLC trace of **3** following subjection to deracemization conditions. t_R = 6.71 min and t_R = 7.57 min.

9. Mechanistic Experiments with Cyanoether 2

9.1 Determination of C–H Bond Formation Selectivity from Cyanoether 2 with Photocatalysts Ir-1–Ir-10

General Procedure

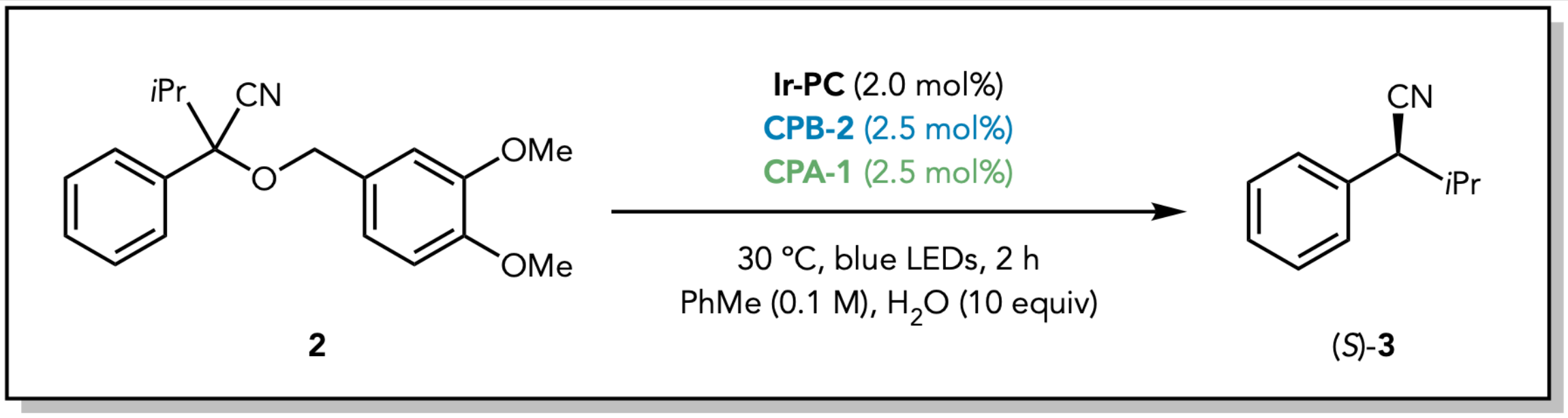

An oven-dried 2-dram vial equipped with a magnetic stir bar was charged with **2** (0.05 mmol, 16.3 mg, 1.00 equiv), photocatalyst (1.0 µmol, 2.0 mol%), chiral phosphate base **CPB-2** (1.25 µmol, 2.50 mol%), and chiral phosphoric acid **CPA-1** (1.25 µmol, 2.50 mol%). The vial was imported into an N_2 atmosphere glovebox and charged with toluene (0.5 mL, 0.1 M). The vial was sealed with a Teflon septum cap and the junction between the cap and vial wrapped with vinyl electrical tape. The reaction mixture was exported from the glovebox and charged with degassed H_2O (10 µL, 10 equiv) via Hamilton syringe, then the top of the Teflon septum cap was taped over with vinyl electrical tape. The reaction was then placed in a photoreactor equipped with 456 nm Kessil lamps (PR160L) set to 100% intensity and irradiated for 2 h. After completion of irradiation, the reaction mixture was concentrated under reduced pressure. Piperonal (7.5 mg, 0.05 mmol, 1.0 equiv) was added as an internal NMR standard via $CDCl_3$ stock solution and an aliquot was removed for mass balance analysis by ^1H NMR spectroscopy. Another aliquot of the reaction was purified by preparative TLC (15% EtOAc/hexanes) and analyzed by chiral HPLC (ChiralCel OJ-H, 10% isopropyl alcohol, 10 min, 210 nm detection) t_R(major) = 7.3 and t_R(minor) = 6.6 min.

Table S9.1. C–H bond formation selectivity vs. photocatalyst reduction potential.

PC	$E_{1/2}$ Ir^{III}/Ir^{II} (V vs Fc^+/Fc)	Yield **3** (%)	Yield **4** (%)	**2** (%)	er **3**	ee **3** (%)
**Ir-1**	-1.11	99	95	nd	85:15	70
**Ir-2**	-1.14	99	94	nd	82:18	64
**Ir-3**	-1.19	99	96	nd	81:19	61
**Ir-4**	-1.28	98	99	nd	80:20	60
**Ir-5**	-1.30	96	96	nd	81:19	62
**Ir-6**	-1.38	69	70	30	75:25	50
**Ir-7**	-1.48	99	98	nd	69:31	38
**Ir-8**	-1.56	99	96	nd	53:47	6
**Ir-9**	-1.64	96	96	nd	58:42	16
**Ir-10**	-1.77	98	94	nd	54:46	8

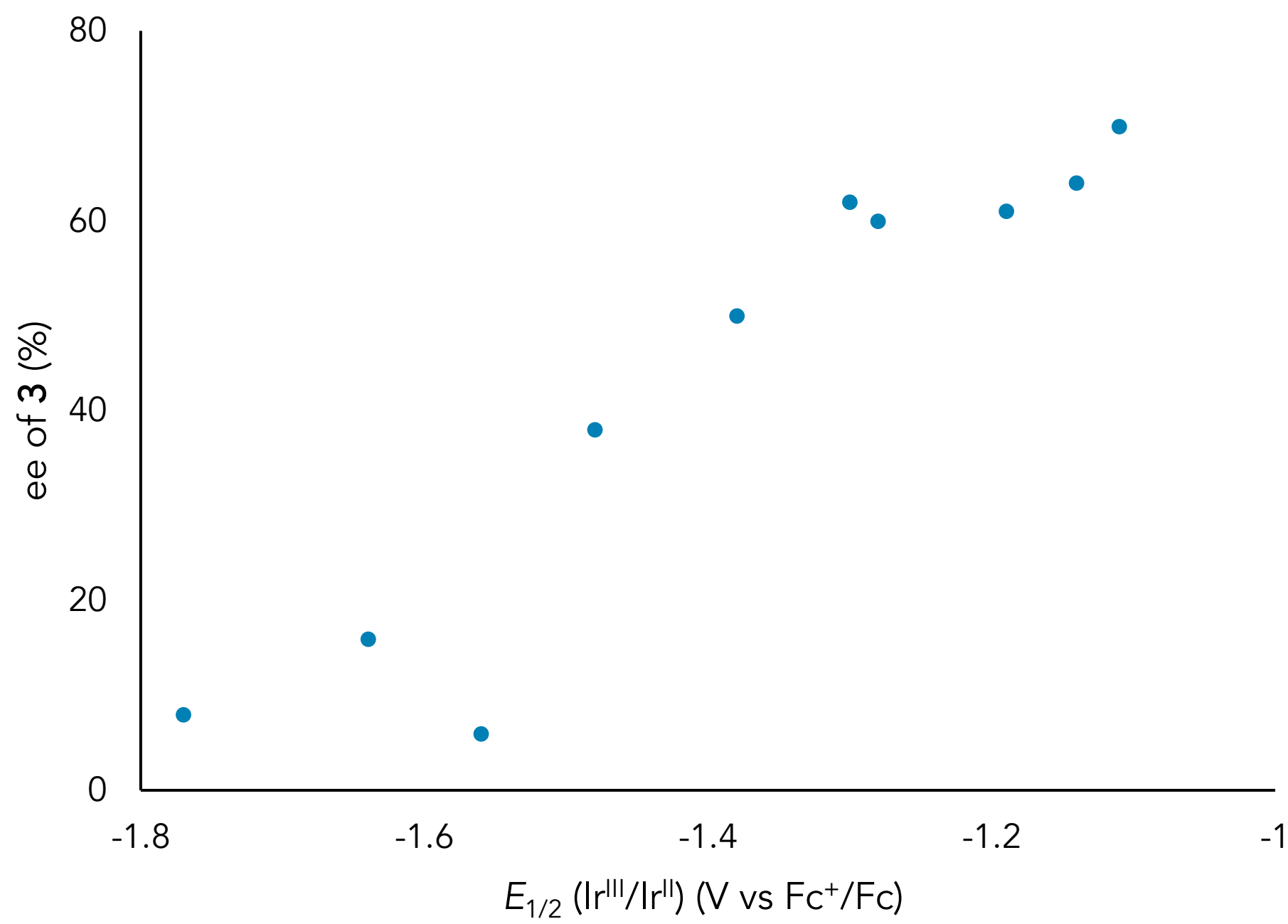

Figure S9.1 C–H bond formation selectivity vs ground-state photocatalyst reduction potential.

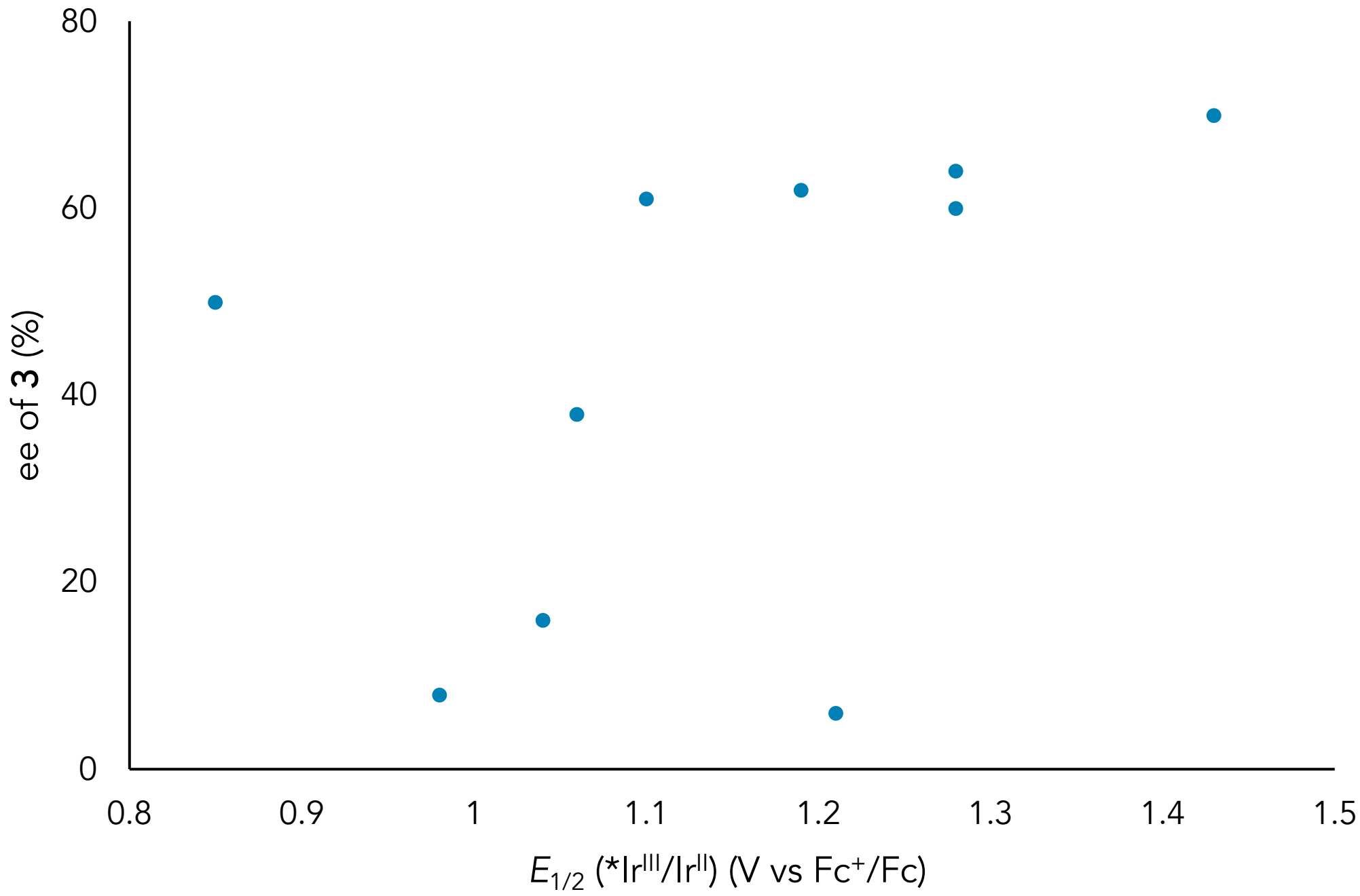

Figure S9.2 C–H bond formation selectivity vs. excited-state photocatalyst reduction potential.

9.2 Determination of Solvent Isotope Effect and Origin of Isotopic Perturbation of ee

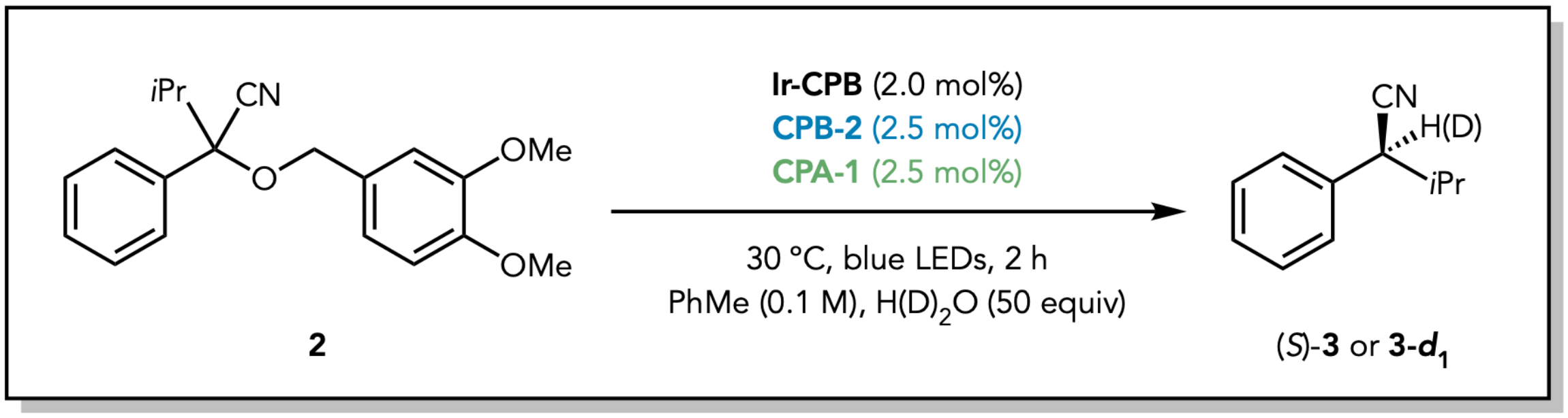

An oven-dried 2-dram vial equipped with a magnetic stir bar was charged with **2** (0.05 mmol, 16.3 mg, 1.00 equiv)**, Ir-CPB** (1.0 µmol, 2.0 mol%), chiral phosphate base **CPB-2** (1.25 µmol, 2.50 mol%), and chiral phosphoric acid **CPA-1** (1.25 µmol, 2.50 mol%). The vial was imported into an N_2 atmosphere glovebox and charged with toluene (0.5 mL, 0.1 M). The vial was sealed with a Teflon septum cap and the junction between the cap and vial wrapped with vinyl electrical tape. The reaction mixture was exported from the glovebox and charged with degassed H_2O or D_2O (50 µL, 50 equiv) via Hamilton syringe, then the top of the Teflon septum cap was taped over with vinyl electrical tape. The reaction was then placed in a photoreactor equipped with 456 nm Kessil lamps (PR160L) set to 100% intensity, and irradiated for 2 h. After completion of irradiation, the reaction mixture was concentrated under reduced pressure. Piperonal (7.5 mg, 0.05 mmol, 1.0 equiv) was added as an internal NMR standard via $CDCl_3$ stock solution and an aliquot was removed for mass balance analysis by ^{1}H NMR spectroscopy. Another aliquot of the reaction was purified by preparative TLC (15% EtOAc/hexanes) and analyzed by chiral HPLC (ChiralCel OJ-H, 10% isopropyl alcohol, 10 min, 210 nm detection) t_R(major) = 7.3 and t_R(minor) = 6.6 min.

Table S9.2. C–H bond formation selectivity is insensitive to solvent isotopic composition.

entry	additive (50 equiv)	Yield **3** (%)	%D **3**	er
**A**	H_2O	97	-	86:14
**B**	D_2O	91	96	86:14

9.3 Determination of C–H Bond Formation KIE with Varying Photocatalysts

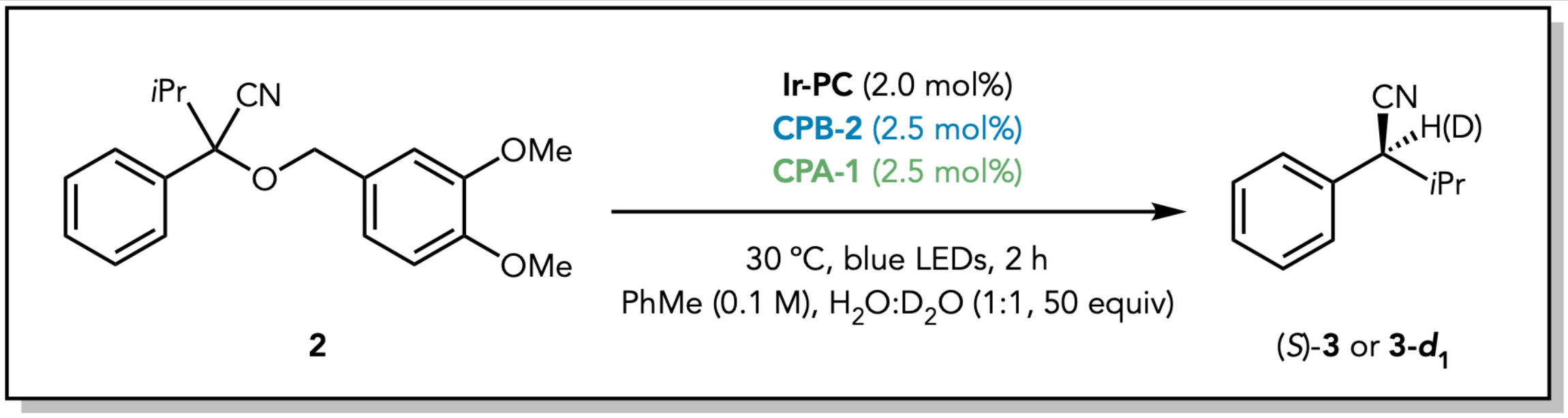

An oven-dried 2-dram vial equipped with a magnetic stir bar was charged with **2** (0.05 mmol, 16.3 mg, 1.00 equiv), photocatalyst (1.0 µmol, 2.0 mol%), chiral phosphate base **CPB-2** (1.25 µmol, 2.50 mol%), and chiral phosphoric acid **CPA-1** (1.25 µmol, 2.50 mol%). The vial was imported into an N_2 atmosphere glovebox and charged with toluene (0.5 mL, 0.1 M). The vial was sealed with a Teflon septum cap and the junction between the cap and vial wrapped with vinyl electrical tape. The reaction mixture was exported from the glovebox and charged with a degassed mixture of 1:1 H_2O:D_2O (50 µL, 50 equiv) via Hamilton syringe, then the top of the Teflon septum cap was taped over with vinyl electrical tape. The reaction was then placed in a photoreactor equipped with 456 nm Kessil lamps (PR160L) set to 100% intensity, and irradiated for 2 h. After completion of irradiation, the reaction mixture was purified by preparative TLC (15% EtOAc/hexanes) and deuterium content analyzed by ^{1}H NMR spectroscopy.

Table S9.3. C–H bond formation KIE with varying photocatalysts.

entry	Ir-PC	%H **3**	KIE	Avg.
A	Ir-CPB	71.0	2.45	
B	Ir-CPB	71.3	2.48	2.47±0.04
C	Ir-3	71.6	2.52	
D	Ir-3	71.2	2.47	2.50±0.04
E	Ir-6	68.1	2.13	
F	Ir-6	68.6	2.18	2.16±0.04
G	Ir-7	70.7	2.41	
H	Ir-7	70.9	2.43	2.42±0.02
I	Ir-9	70.8	2.42	
J	Ir-9	70.6	2.40	2.41±0.03
K	Ir-10	70.6	2.40	
L	Ir-10	70.4	2.38	2.39±0.03

9.4 Control experiment for aldehyde effects on enantioselectivity

General Procedure

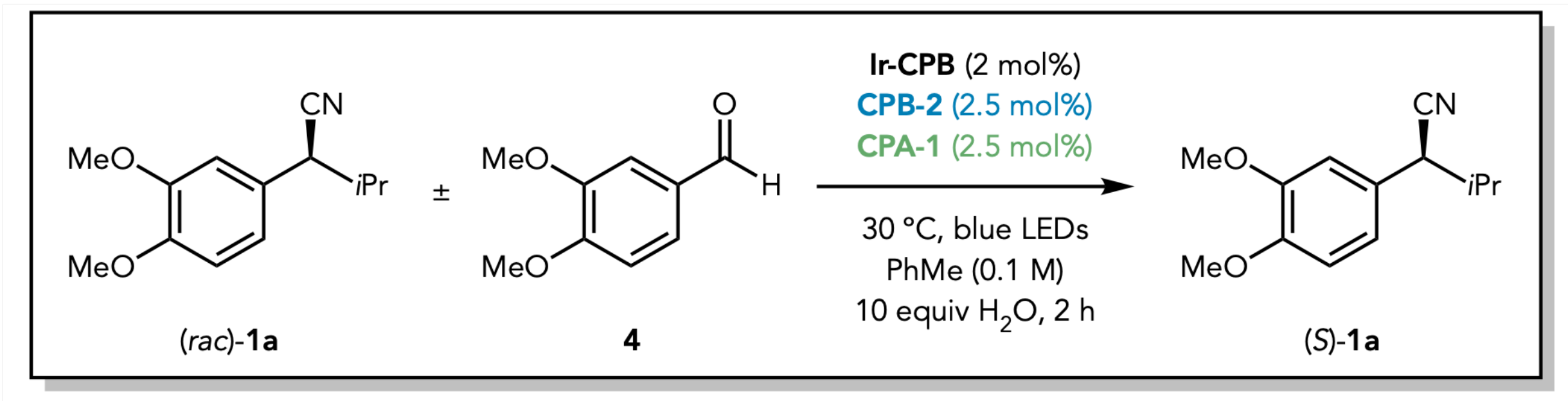

An oven-dried 2-dram vial equipped with a magnetic stir bar was charged with racemic nitrile (0.05 mmol, 1.00 equiv), aldehyde **4** (0.0 mmol, 0 equiv or 0.05 mmol, 1.00 equiv), **Ir-CPB** (1.0 µmol, 2.00 mol%), **CPB-2** (1.25 µmol, 2.50 mol%), and **CPA-1** (1.25 µmol, 2.50 mol%). The vial was imported into an N_2 atmosphere glovebox and charged with toluene (0.25 mL, 0.20 M). The vial was sealed with a Teflon septum cap and the junction between the cap and vial wrapped with electrical tape. The reaction mixture was exported from the glovebox and charged with degassed H_2O (10 µL, 10 equiv) via Hamilton syringe, then the top of the vial cap was sealed with electrical tape. The reaction was then placed in a photoreactor equipped with a recirculating ice-water bath and 456 nm Kessil lamps (PR160L) set to 100% intensity and irradiated for 2 h. Upon completion, the reaction mixture was opened to air and concentrated *in vacuo*. The crude material was redissolved in 500 µL of $CDCl_3$ with dibromomethane (0.1 M) as an internal standard. The other half of the solution was purified by preparative thin layer chromatography (15% EtOAc/hexanes) and subsequently analyzed for enantiomeric excess via HPLC (AD-H, 10% isopropyl alcohol in hexanes, 15 min, 230 nm detection).

Table S9.4. Deracemization selectivity in the presence and absence of external aldehyde.

Entry	Aldehyde equivalents	Yield (%)	1a ee (%)
A	0	>99	94
B	1	98	93

Extrapolating, the selectivity of C–H bond formation is likely not affected by the *in situ* formation of aldehyde **4**.

eb

10. Stoichiometric Protonation Experiments with CPA-1

10.1 Synthesis of Keteniminate 1a⁻

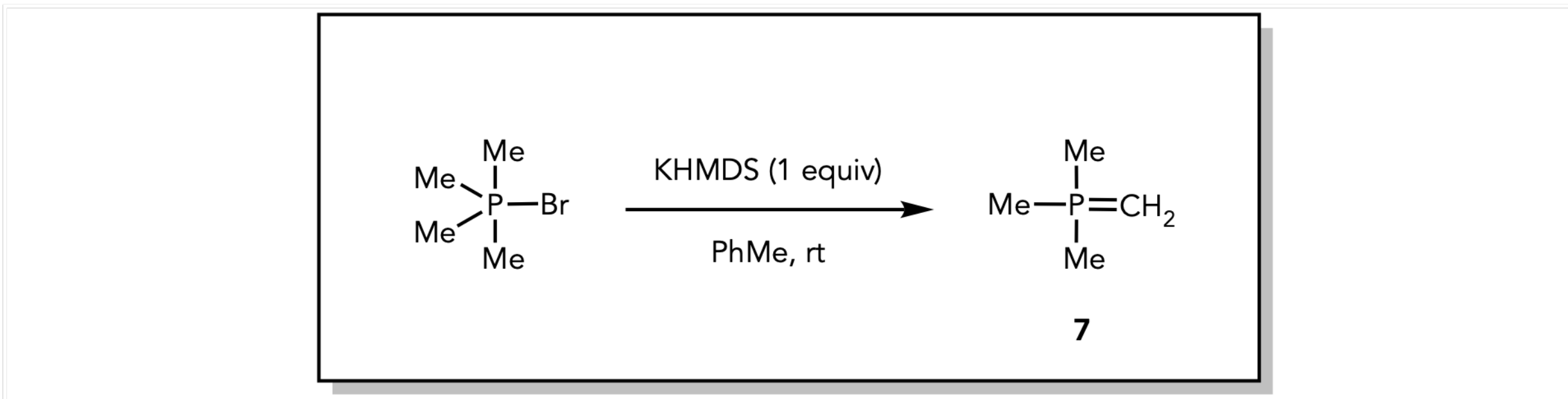

Ylide **7** was synthesized according to a modified literature procedure.[11] In an oven-dried 2-dram vial, vacuum-dried tetramethylphosphonium bromide (56 mg, 0.33 mmol, 1.0 equiv) was suspended in anhydrous toluene (1.5 mL) in an N_2 atmosphere glovebox. KHMDS (1.1 M solution in toluene, 3.0 mL, 0.33 mmol, 1.0 equiv) was added to the suspension and stirred at room temperature for 4 h. The mixture was filtered through a PTFE syringe (0.2 µm pore size) and used immediately without further purification.

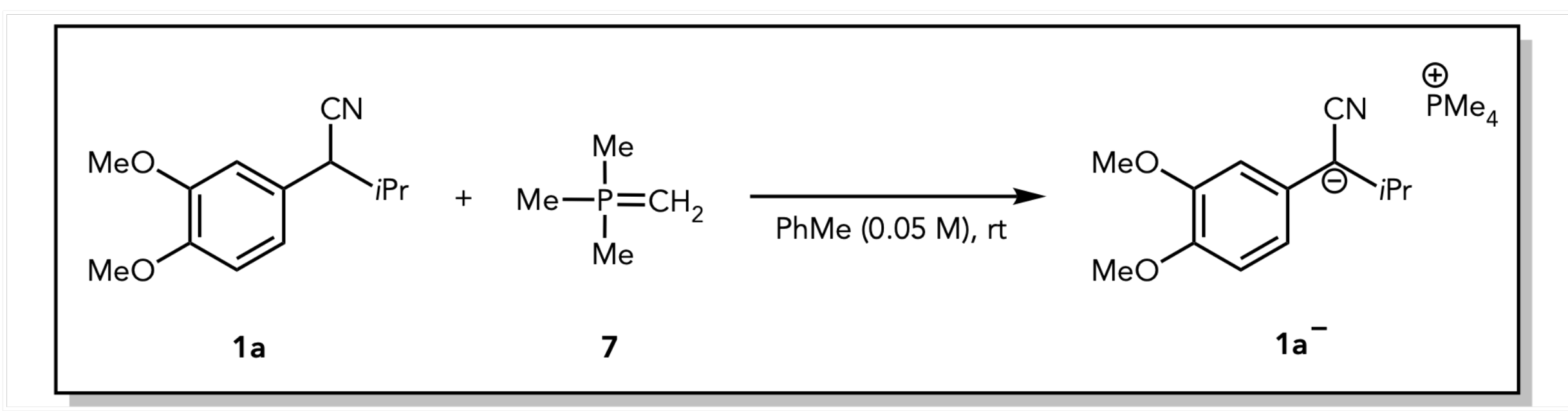

To an oven-dried 2-dram vial was charged with nitrile **1a** (1.0 equiv, 33 mg, 0.15 mmol) and anhydrous toluene (1.0 mL) in a N_2 atmosphere glovebox. Ylide **7** (0.072 M solution in toluene, 2.1 mL, 0.15 mmol, 1.0 equiv) was added dropwise to the solution and stirred at room temperature for 30 minutes. The solution was used immediately without further purification.

10.2 Deuterium Incorporation Experiment to Determine Extent of Deprotonation

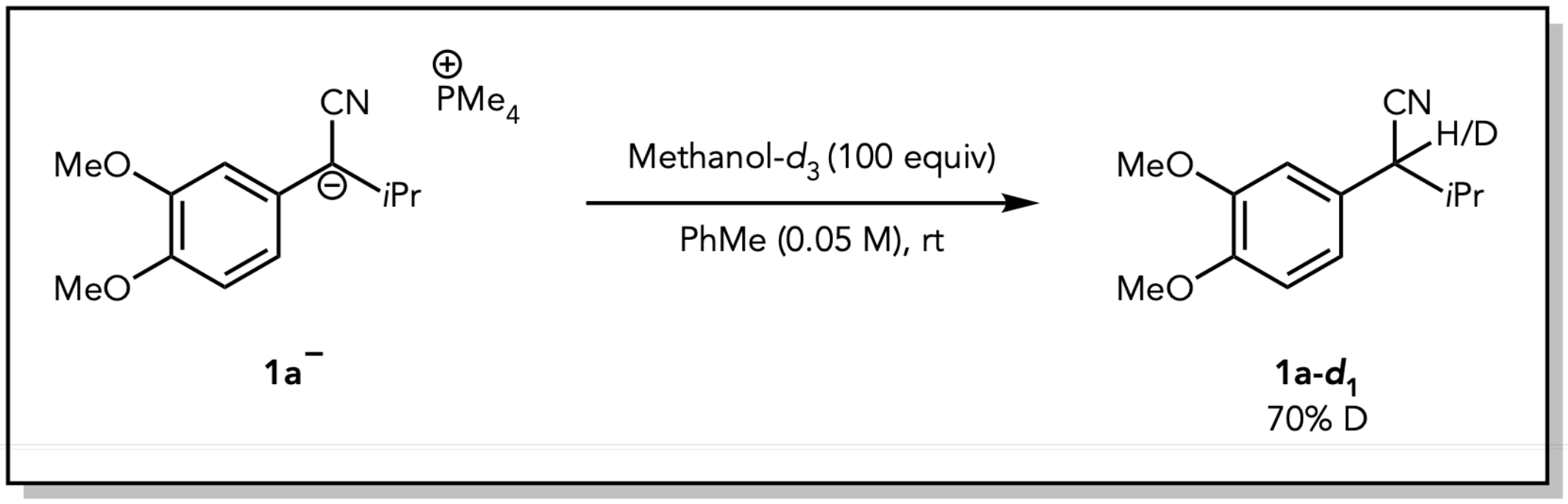

In an N_2 atmosphere glovebox, 0.2 mL anhydrous methanol-d_4 (100 equiv) was added to **1a**$^-$ (0.05 M solution in toluene, 11 mg, 0.05 mmol, 1.0 equiv). The reaction mixture was allowed to stir for 10 minutes at room temperature, then passed through a plug of silica gel. The crude material was concentrated under reduced pressure and an aliquot was removed to analyze deuterium incorporation by ^{1}H NMR.

The benzylic proton had undergone deuterium exchange in 70% of the material.

10.3 Stoichiometric Protonation with Excess CPA-1

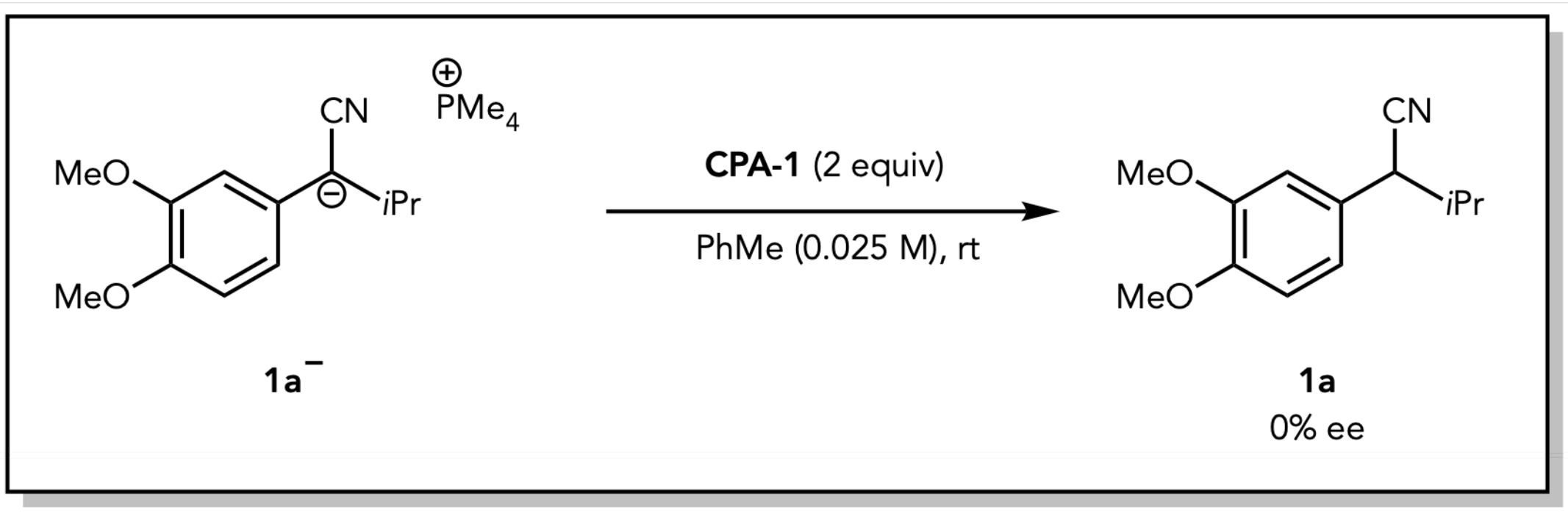

In an N_2 atmosphere glovebox, **1a**$^-$ (0.05 M solution in toluene, 11 mg, 0.05 mmol, 1.0 equiv) was added dropwise to a solution of **CPA-1** (75 mg, 0.10 mmol, 2.0 equiv) in toluene (1 mL). The reaction mixture was allowed to stir for 10 minutes at rt, then passed through a plug of silica gel. The crude material was concentrated under reduced pressure and then an aliquot of the material was purified by preparative thin layer chromatography (15% EtOAc/hexanes). The enantiomeric excess of the scalemic material was analyzed by HPLC (AD-H, 10% isopropyl alcohol/hexanes, 10 min, 230 nm detection).

The optical purity of **1a** was determined to be 0% ee.

10.4 Stoichiometric Protonation with Excess CPA-1 and Water

Water was included in this experiment to mirror the relative concentrations of water that would be present in the standard conditions of the deracemization reaction. The overall toluene:water ratio was 25:1 to mirror the 100 µL of water and 2.5 mL toluene.

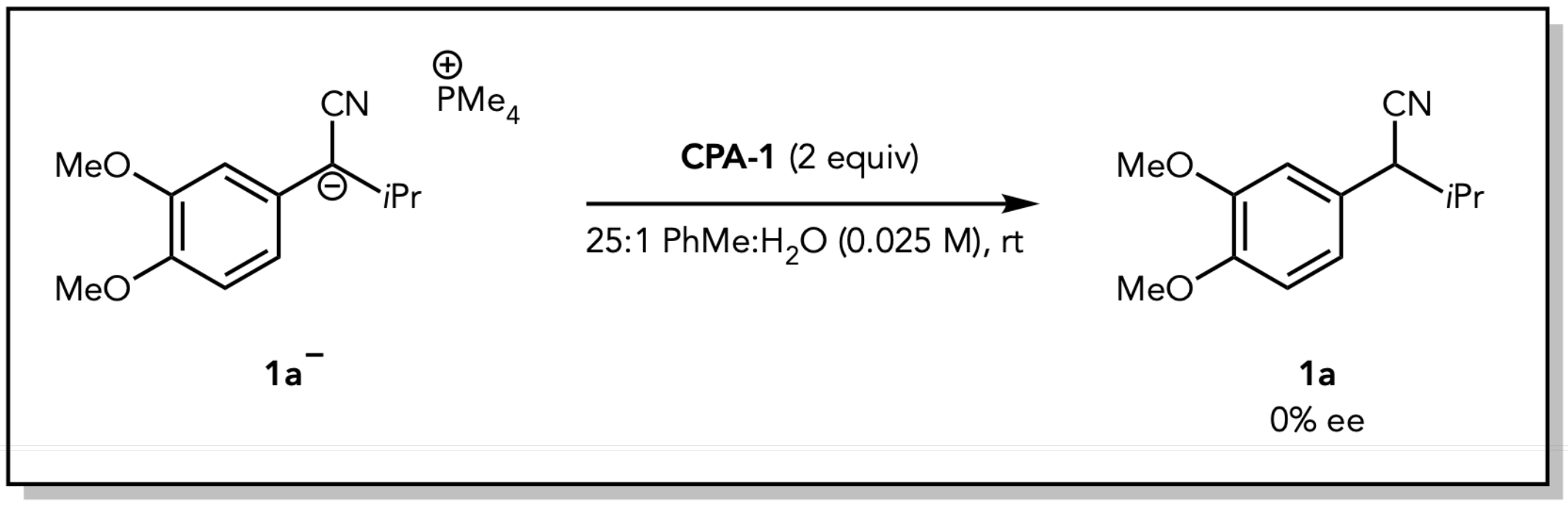

In an N_2 atmosphere glovebox, **1a**$^-$ (0.05 M solution in toluene, 11 mg, 0.05 mmol, 1.0 equiv) was added dropwise to a solution of **CPA-1** (75 mg, 0.10 mmol, 2.0 equiv) in 12.5:1 toluene:water (1 mL). The reaction mixture was allowed to stir for 10 minutes at room temperature, then passed through a plug of silica gel. The crude material was concentrated under reduced pressure and then an aliquot of the material was purified by preparative thin layer chromatography (15% EtOAc/hexanes). The enantiomeric excess of the scalemic material was analyzed by HPLC (AD-H, 10% isopropyl alcohol/hexanes, 10 min, 230 nm detection).

The optical purity of **1a** was determined to be 0% ee.

10.5 Configurational Stability of 1a in Keteniminate Protonation Conditions

In an N_2 atmosphere glovebox, **1a**$^-$ (0.05 M solution in toluene, 11 mg, 0.05 mmol, 1.0 equiv) was added dropwise to a solution of **CPA-1** (75 mg, 0.10 mmol, 2.0 equiv) and (*S*)-**1a** (11 mg, 0.05 mmol, 1.0 equiv) in toluene (1 mL). The reaction mixture was allowed to stir for 10 minutes at room temperature, then passed through a plug of silica gel. The crude material was concentrated under reduced pressure and then an aliquot of the material was purified by preparative thin layer chromatography (15% EtOAc/hexanes). The enantiomeric excess of the scalemic material was analyzed by HPLC (AD-H, 10% isopropyl alcohol/hexanes, 10 min, 230 nm detection).

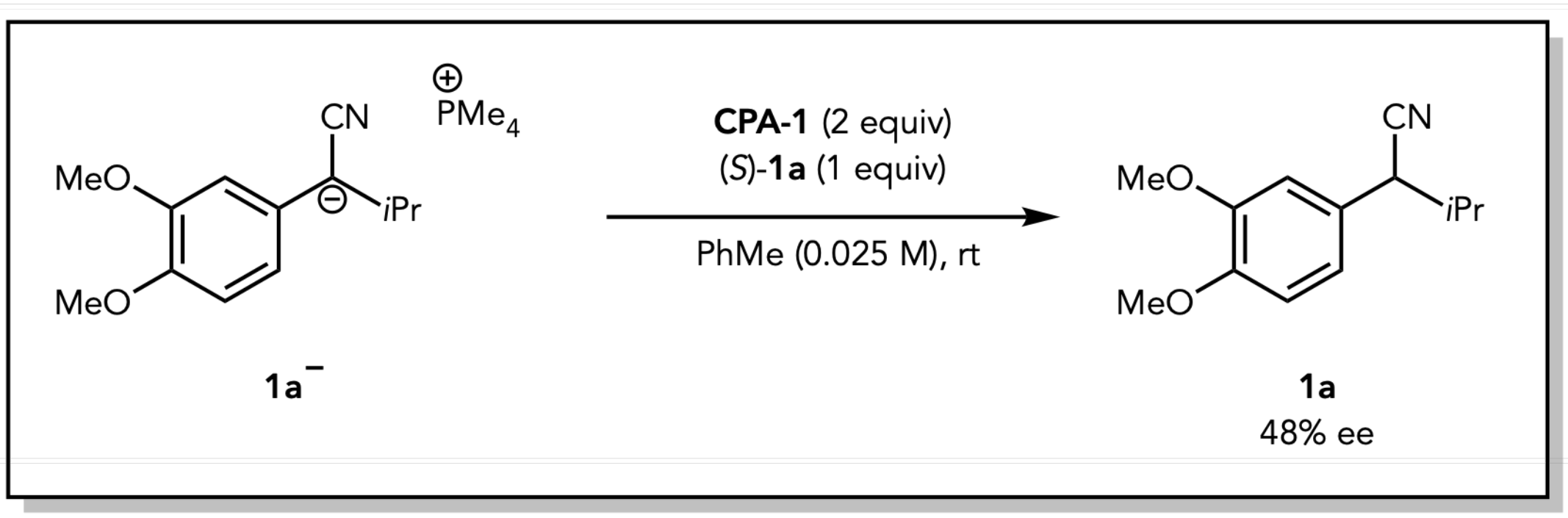

The optical purity of the product **1a** was determined to be 48% ee.

Assuming the enantiopure (*S*)-**1a** added did not undergo racemization in the presence of acid, phosphate, or keteniminate **1a**$^-$ during reaction, the theoretical enantiomeric ratio is (50+100):50 er, which simplifies to 75:25 er or 50% ee.

The optical purity of **1a** was 48% ee, and therefore there no significant racemization was observed during the reaction to erode the optical purity of **1a** formed during the reaction or that of (*S*)-**1a** added at the start of reaction.

11. Low-conversion Deuterium Incorporation Experiments

11.1 General Approach

In this section, we estimate the enantioselectivity of C–H bond formation with model substrate **1a** using the method of low-conversion isotope exchange developed by Wendlandt and coworkers.[12] This method has not been previously reported for the study of deracemization reactions, and therefore the general derivation is recapitulated below.

To simplify this analysis, we make the following assumptions about the system: (a) negligible isotopic fractionation, (b) the products are not processed again in low-conversion regimes, (c) perfect mass balance of enantiomers (no background kinetic resolution), and (d) substrate that undergoes unproductive BET is considered 'unprocessed.'

11.2 Definitions and Equations

We define the following sets of variables,

(*S*)-H := mole fraction protiated (*S*)-**1a**

(*S*)-D := mole fraction deuterated (*S*)-**1a**

(*S*)-Tot := total mole fraction (*S*)-**1a**

(*R*)-H := mole fraction protiated (*R*)-**1a**

(*R*)-D := mole fraction deuterated (*R*)-**1a**

(*R*)-Tot := total mole fraction (*R*)-**1a**

u := unprocessed substrate

d := efficiency of deuteration in back-end

p := % substrate processed

s := back-end selectivity

The following equations are adapted from the report by Wendlandt and coworkers.[12]

d **– Deuterium Incorporation Efficiency**

Any non-deuterated (*S*)-**1a** is the result of incomplete deuterium exchange between the phosphate base and solvent.

$$d = \frac{(S)-D}{(S)-Tot}$$

***p* – Fraction of Substrate Processed**

The fraction of substrate processed p may be determined by looking at total (S)-**1a** plus deuterated (R)-**1a**, corrected by a factor of $1/d$ to account for processed, but protiated (R)-**1a**,

$$p = (S) - Tot + \frac{(R)-D}{d}$$

***s* – C–H Bond Formation Selectivity**

The selectivity of the step that reforms the C–H or C–D bond may be calculated by considering the fraction of (S)-**1a** formed relative to the total material processed,

$$s = \frac{S-Tot}{p}$$

This value, the percent selectivity for forming (S)-**1a** is readily converted to C–H bond formation selectivity er_2,

$$er_2 = \frac{s}{1-s}$$

Finally, we posit that the selectivity of the stereoablative step, er_1, may be directly extracted, provided the overall selectivity of the reaction, er_{obs},

$$er_1 = \frac{er_{obs}}{er_2}$$

11.3 General Procedures for Low-Conversion Deuterium Incorporation Experiments

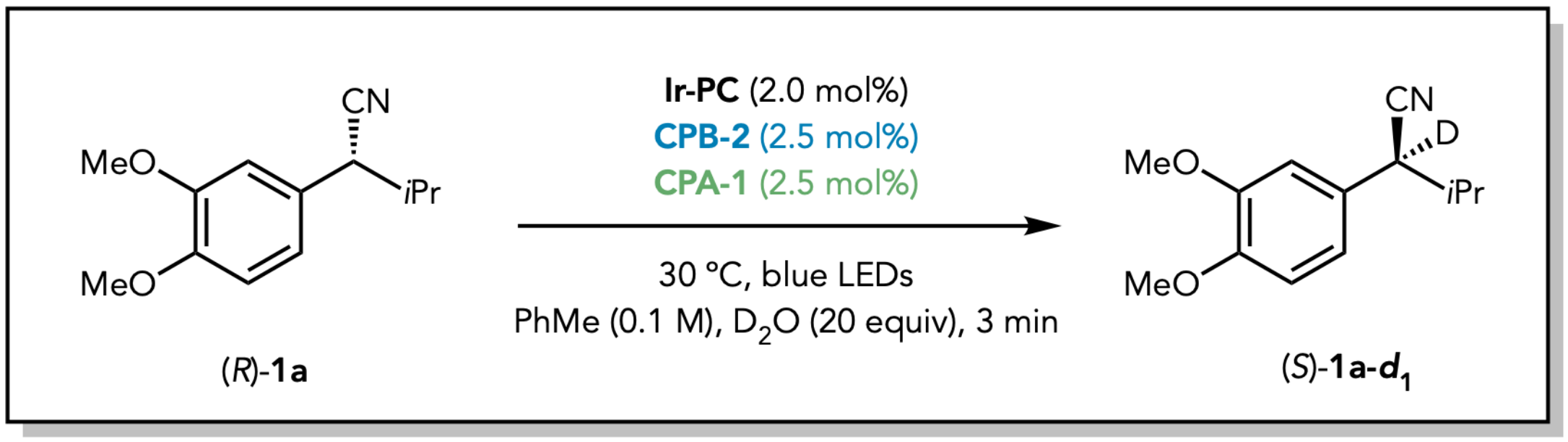

An oven-dried 2-dram vial equipped with a magnetic stir bar was charged with (*R*)-**1a** (0.25 mmol, 1.00 equiv), photocatalyst (5.0 µmol, 2.0 mol%), **CPB-2** (6.3 µmol, 2.5 mol%), and chiral **CPA-1** (6.3 µmol, 2.5 mol%). The vial was imported into an N_2 atmosphere glovebox and charged with toluene (1.3 mL, 0.20 M). The vial was sealed with a Teflon septum cap and the junction between the cap and vial wrapped with vinyl electrical tape. The reaction mixture was exported from the glovebox and charged with degassed D_2O (100 µL, 20 equiv) via Hamilton syringe. The reaction was then placed in a photoreactor equipped with 456 nm Kessil lamps (PR160L) set to 100% intensity and irradiated for 3 minutes unless otherwise noted. Upon completion, the reaction mixture was opened to air and concentrated *in vacuo*. The product was purified by preparative thin layer chromatography (15% EtOAc/hexanes). The enantiomeric excess of the scalemic material was analyzed by HPLC (AD-H, 10% isopropyl alcohol in hexanes, 15 min, 230 nm detection), and the enantiomers were separated by LOTUS Separations (ColumnTek Enantiocel® A6-5 (250 × 4.6 mm) 2.0 mL/min @ 15% methanol / 85% CO_2 (100 bar backpressure); 220 nm detection).

HIE A: with **Ir-CPB** as photocatalyst.

HIE B: with **Ir-10** as photocatalyst.

Deuterium incorporation was calculated relative to the integration of the isopropyl methine C–H ($C_{ref}H$). To account for potential fractionation in the starting material, the final deuterium incorporation for the alpha-nitrile C–H ($C_\alpha H$) was,

$$D = 1 - \frac{C_\alpha H_f}{C_{ref} H_f} \div \frac{C_\alpha H_0}{C_{ref} H_0}$$

Where C_iH_0 denotes the relative integrations of protons in starting material that have not been subjected to deracemization.

11.4 Low-Conversion Isotope Exchange Study of HIE A

Representative spectra for **HIE A** are provided below. Replicate scans and additional experimental data are provided in the following tables.

^{1}H NMR (500 MHz, $CDCl_3$) Spectrum for Unprocessed (*R*)-1a

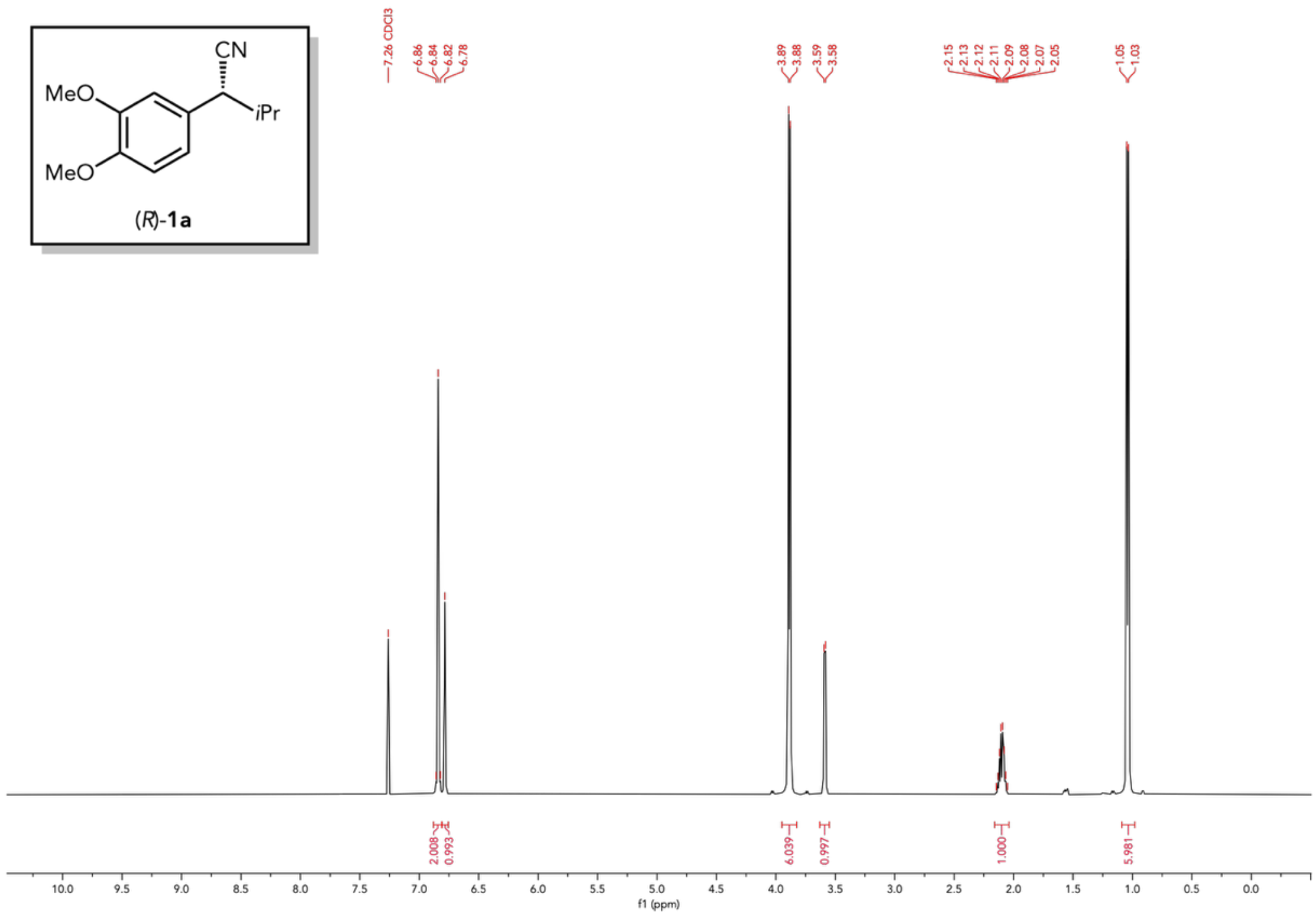

^{1}H NMR (500 MHz, $CDCl_3$) spectrum for partially deuterated (*R*)-1a

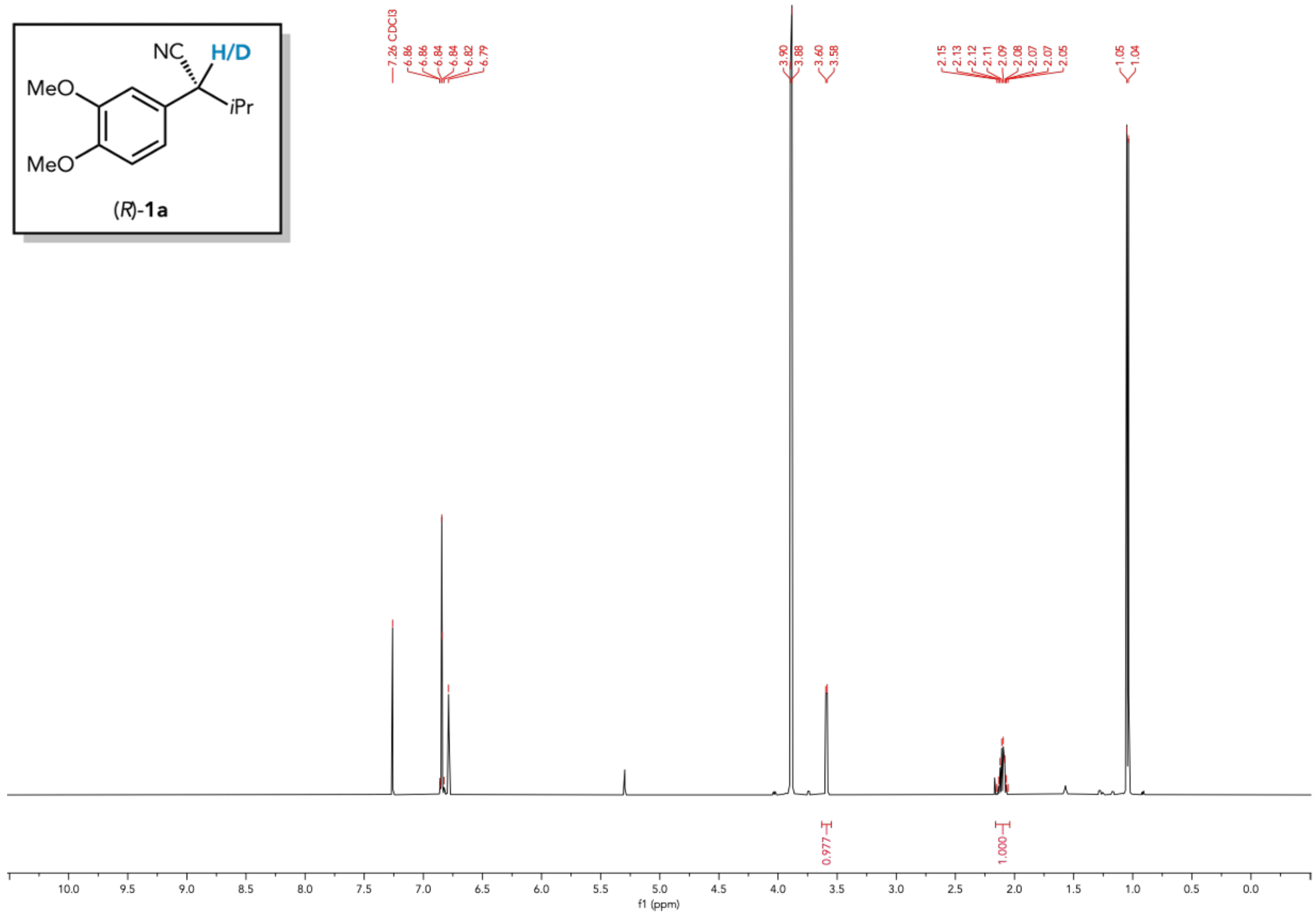

^{1}H NMR (500 MHz, $CDCl_3$) spectrum for partially deuterated (*S*)-1a

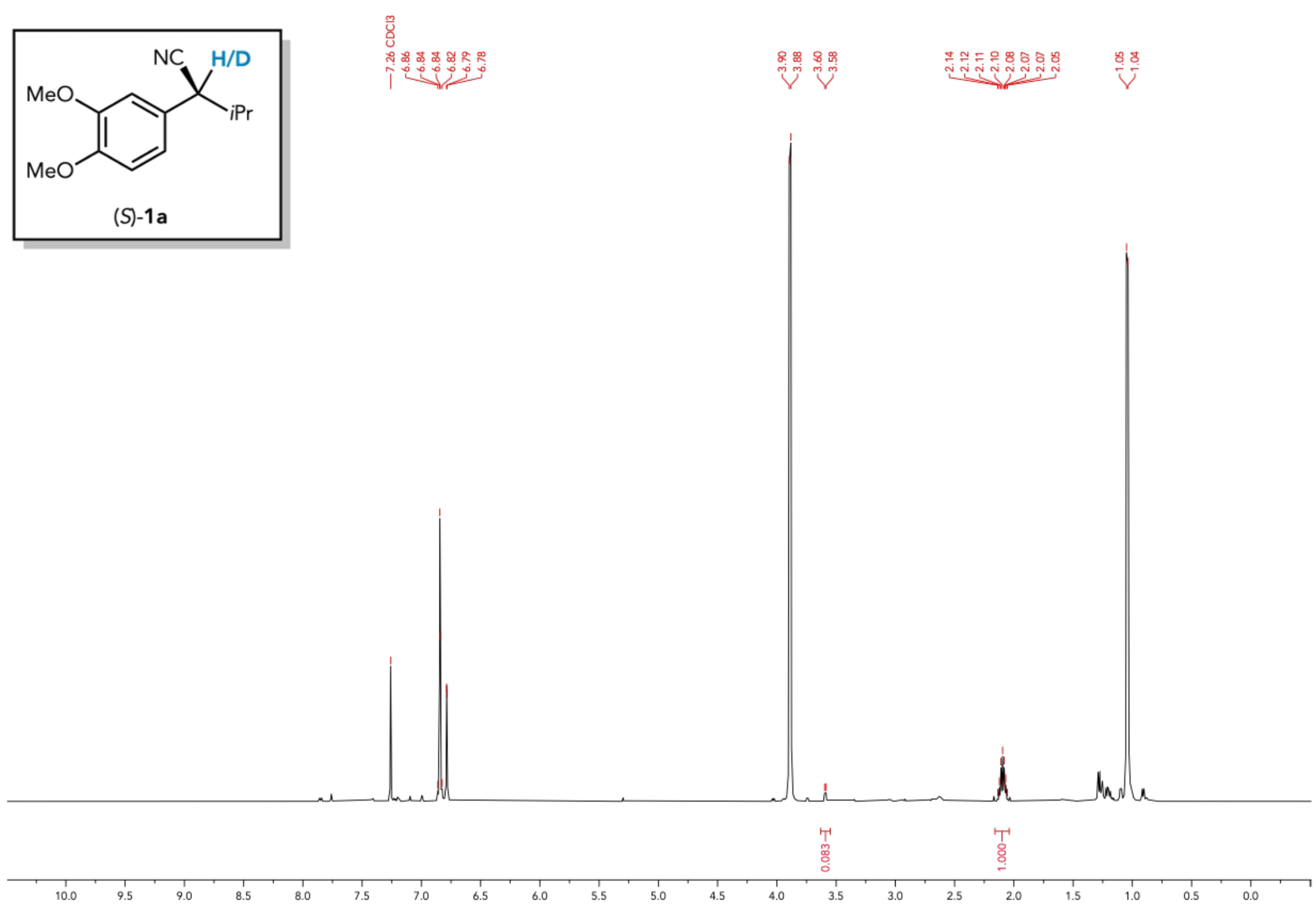

Table S11.1 Integrations for unprocessed (*R*)-**1a**

Trial	C_{ref}–H (2.04 - 2.16 ppm)	C_{α}-H (3.55 - 3.63 ppm)
**1**	1.000	0.997
**2**	1.000	0.997
**3**	1.000	0.997
**Avg**	1.000	0.997
**Std Dev**	0.000	0.000

Table S11.2 Integrations for partially deuterated (*S*)-**1a** and (*R*)-**1a**

	(*S*)-**1a**		(*R*)-**1a**	
**Trial**	C_{ref}–H (2.04 - 2.16 ppm)	C_{α}-H (3.55 - 3.63 ppm)	C_{ref}–H (2.04 - 2.16 ppm)	C_{ref}–H (2.04 - 2.16 ppm)
**1**	1.000	0.083	1.000	0.977
**2**	1.000	0.082	1.000	0.977
**3**	1.000	0.083	1.000	0.976
**Avg**	1.000	0.083	1.000	0.977
**Std Dev**	0.000	0.001	0.000	0.001

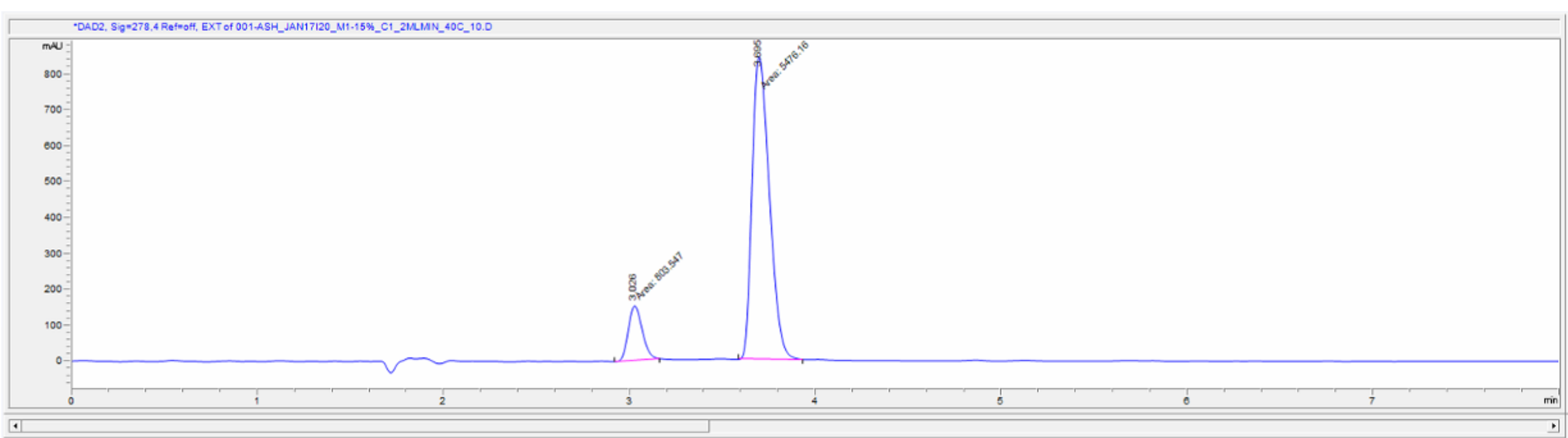

#	Time	Type	Area	Height	Width	Area%	Symmetry
1	3.026	MM	803.5	152.9	0.0876	12.796	0.799
2	3.695	MM	5476.2	845.6	0.1079	87.204	0.657

Figure S11.1. HPLC trace of partially deuterated (*S*)-**1a** and (*R*)-**1a**. t_R (*S*)-**1a** = 3.026 min and t_R (*R*)-**1a** = 3.695 min

Table S11.3 Calculated mole fractions of partially deuterated (*S*)-**1a** and (*R*)-**1a**.

Trial	(*S*)–D	(*S*)–H	(*S*)–Tot	(*R*)–D	(*R*)–H	(*R*)–Tot
**1**	0.1173	0.0107		0.0175	0.8545	
**2**	0.1174	0.0105	**0.1280**	0.0175	0.8545	**0.8720**
**3**	0.1173	0.0107		0.0184	0.8537	
**Avg**	0.1173	0.0106	-	0.0178	0.8542	-
**Std Dev**	7.41E-05	7.41E-05	-	0.0005	0.0005	-

Table S11.4 Calculated selectivity parameters for **HIE A**.

Trial	*d*	*p*	*s*	er_2	er_1	er_{obs}
**1**	0.9168	0.1470	0.8702	6.7059	4.8217	
**2**	0.9178	0.1470	0.8704	6.7131	4.8164	**32.3333**
**3**	0.9168	0.1480	0.8646	6.3865	5.0627	
**Avg**	0.9171	0.1473	0.8684	6.6019	4.9003	-
**Std Dev**	0.0006	0.0006	0.0033	0.1865	0.1407	-

The selectivity of C–H bond formation under standard conditions was estimated to be 6.6 ± 0.38 and selectivity of C–H bond cleavage to be 4.9 ± 0.28.

11.5 Low-Conversion Isotope Exchange Study of HIE B

Representative spectra for **HIE B** are provided below. Replicate scans and additional experimental data are provided in the tables following this section.

^{1}H NMR (800 MHz, $CDCl_3$) Spectrum for Partially Deuterated (*R*)-1a

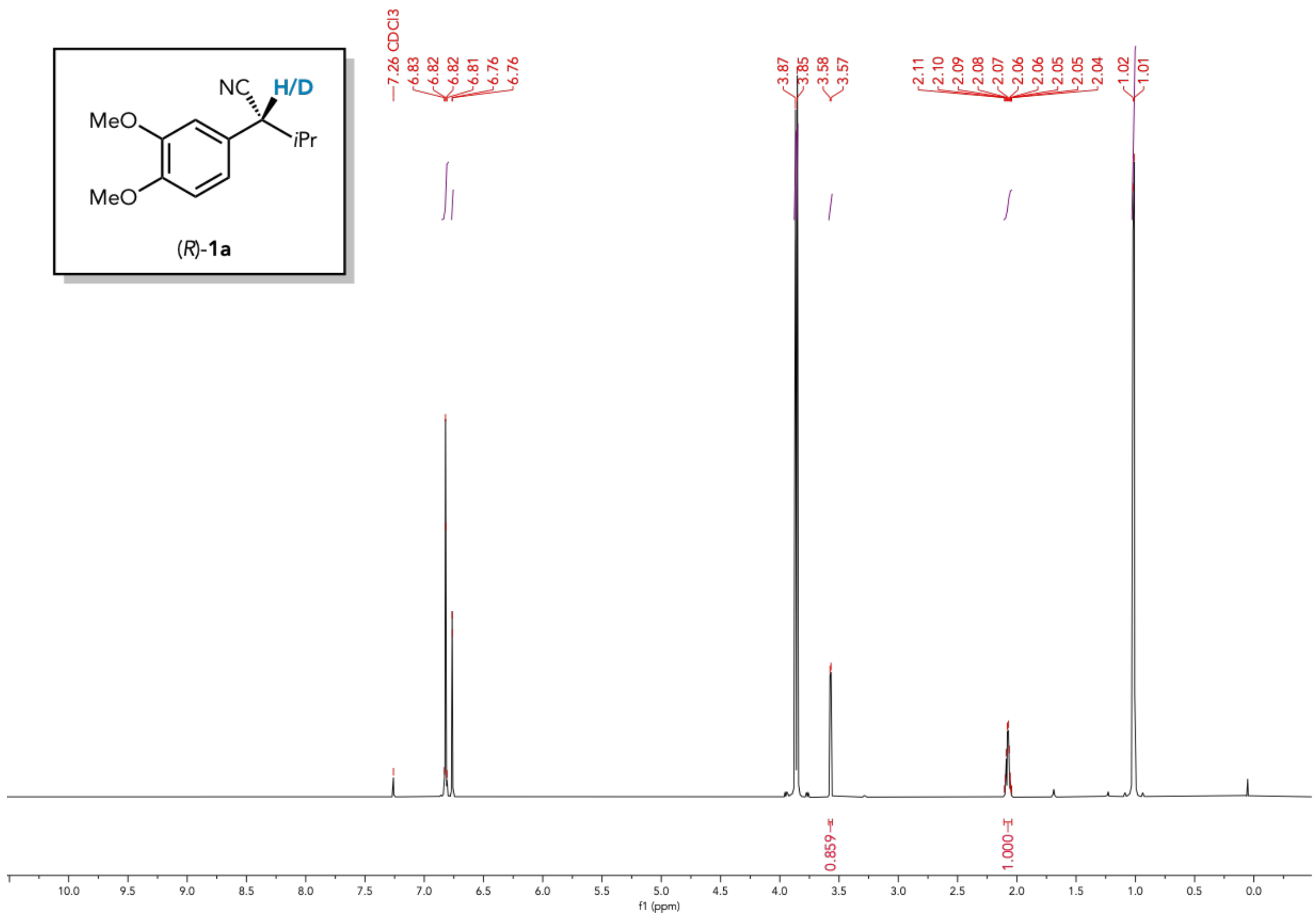

^{1}H NMR (500 MHz, $CDCl_3$) Spectrum for Partially Deuterated (*S*)-1a

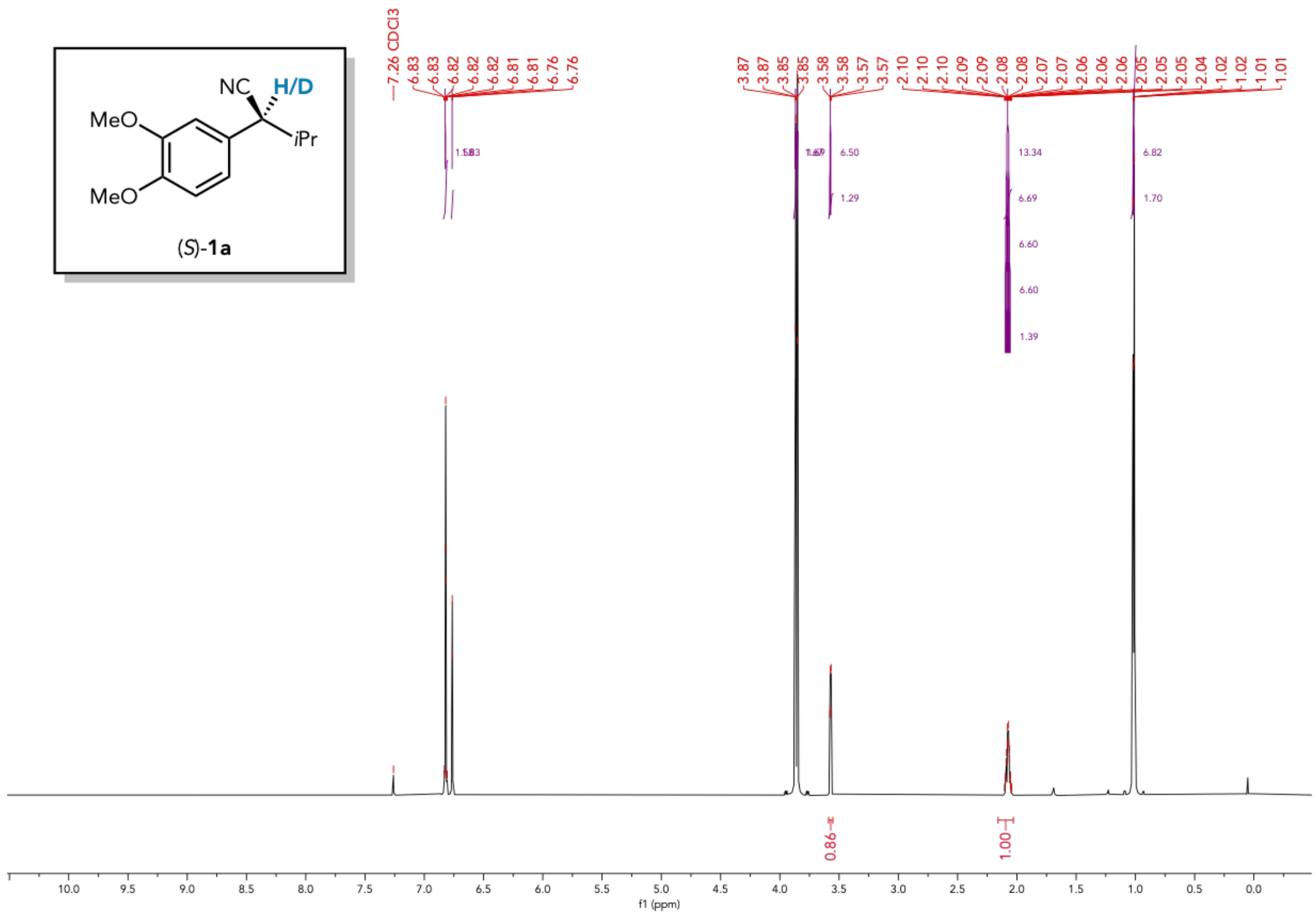

Table S11.5 Integrations for partially deuterated (*S*)-**1a** and (*R*)-**1a** – Set 1 of 2.

Data from Set 1 was acquired following irradiation of sample for 5 minutes.

	**(*S*)-1a**		**(*R*)-1a**	
**Trial**	C_{ref}–H (2.04 - 2.16 ppm)	C_{α}-H (3.55 - 3.63 ppm)	C_{ref}–H (2.04 - 2.16 ppm)	C_{ref}–H (2.04 - 2.16 ppm)
**1**	1.000	0.092	1.000	0.859
**2**	1.000	0.088	1.000	0.853
**Avg**	1.000	0.090	1.000	0.856
**Std Dev**	0.000	0.003	0.000	0.004

Table S11.6 Integrations for partially deuterated (*S*)-**1a** and (*R*)-**1a** – Set 2 of 2.

Data from Set 2 was acquired following irradiation of sample for 3 minutes.

	**(*S*)-1a**		**(*R*)-1a**	
**Trial**	C_{ref}–H (2.04 - 2.16 ppm)	C_{α}-H (3.55 - 3.63 ppm)	C_{ref}–H (2.04 - 2.16 ppm)	C_{ref}–H (2.04 - 2.16 ppm)
**1**	1.000	0.071	1.000	0.926
**2**	1.000	0.071	1.000	0.926
**Avg**	1.000	0.071	1.000	0.926
**Std Dev**	0.000	0.000	0.000	0.000

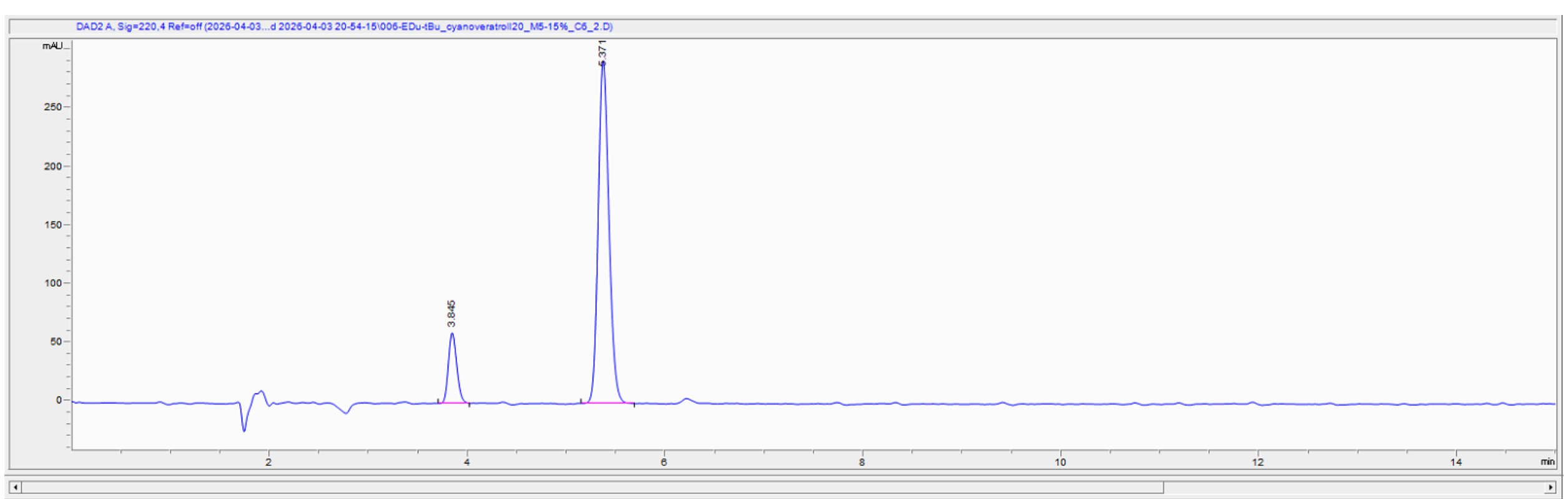

#	Time	Type	Area	Height	Width	Area%	Symmetry
1	3.845	BB	353.1	60.4	0.0902	13.732	0.804
2	5.371	BB	2218.5	293.9	0.1161	86.268	0.804

Figure S11.2. HPLC trace of partially deuterated (*S*)-**1a** and (*R*)-**1a**. t_R (*S*)-**1a** = 3.85 min and t_R (*R*)-**1a** = 5.37 min – Set 1 of 2

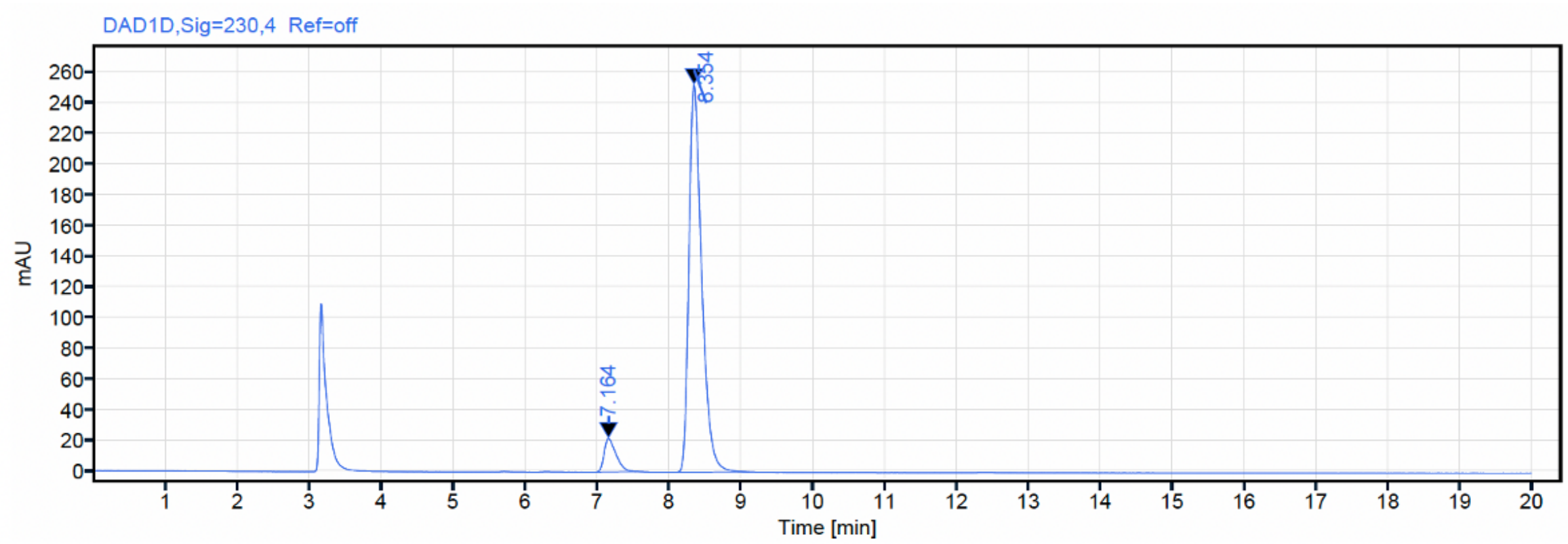

Signal:	DAD1D,Sig=230,4 Ref=off					
**RT [min]**	**Type**	**Width [min]**	**Area**	**Height**	**Area%**	**Name**
7.164	MM m	0.65	250.27	21.85	7.43	
8.354	MM m	1.02	3118.49	252.33	92.57	
		**Sum**	**3368.76**			

Figure S11.3. HPLC trace of partially deuterated (*S*)-**1a** and (*R*)-**1a**. t_R (*S*)-**1a** = 3.845 min and t_R (*R*)-**1a** = 5.371 min – Set 2 of 2.

Table S11.7 Calculated mole fractions of partially deuterated (*S*)-**1a** and (*R*)-**1a** – Set 1 of 2.

Trial	(*S*)–D	(*S*)–H	(*S*)–Tot	(*R*)–D	(*S*)–H	(*R*)–Tot
**1**	0.1246	0.0127		0.1220	0.7433	
**2**	0.1252	0.0121	**0.1373**	0.1185	0.7467	**0.8653**
**Avg**	0.1249	0.0124	-	0.1203	0.7450	-
**Std Dev**	3.90E-04	3.90E-04	-	2.40E-03	2.40E-03	-

Table S11.8 Calculated mole fractions of partially deuterated (*S*)-**1a** and (*R*)-**1a** – Set 2 of 2.

Trial	(*S*)–D	(*S*)–H	(*S*)–Tot	(*R*)–D	(*S*)–H	(*R*)–Tot
**1**	0.0687	0.0053		0.0687	0.8601	
**2**	0.0687	0.0053	**0.0370**	0.0687	0.8601	**0.9288**
**Avg**	0.0687	0.0053	-	0.0687	0.8601	-
**Std Dev**	0.0000	0.0000	-	0.0000	0.0000	-

Table S11.9 Calculated selectivity parameters for **HIE B** – Set 1 of 2.

Trial	*d*	*p*	*s*	$er_{back\text{-}end}$	$er_{front\text{-}end}$	er_f
**1**	0.9077	0.2717	0.5054	1.0217	5.5464	-
**2**	0.9117	0.2673	0.5137	1.0561	5.3654	**5.6667**
**Avg**	0.9097	0.2699	0.5095	1.0389	5.4559	-
**Std Dev**	0.0003	0.0003	0.0006	0.0244	0.1280	-

Table S11.10 Calculated selectivity parameters for **HIE B** – Set 2 of 2.

Trial	*d*	*p*	*s*	$er_{back\text{-}end}$	$er_{front\text{-}end}$	er_f
**1**	0.9288	0.1480	0.5000	1.0000	5.6667	-
**2**	0.9288	0.1480	0.5000	1.0000	5.6667	**5.6667**
**Avg**	0.9288	0.1480	0.5000	1.0000	5.6667	-
**Std Dev**	0.0000	0.0000	0.0000	0.0000	0.0000	-

Table S11.11 Average of calculated selectivity parameters for HIE B.

Set	$er_{back\text{-}end}$	$er_{front\text{-}end}$	er_f
**1**	1.0389	5.4559	-
**2**	1.0000	5.6667	**5.6667**
**Avg**	1.0195	5.5613	-
**Std Dev**	0.0275	0.1491	-

The selectivity of C–H bond formation under standard conditions was estimated to be 1.02 ± 0.06 and the selectivity of C–H bond cleavage under standard conditions was estimated to be 5.6 ± 0.30.

12. Determination of KIE for Deprotonation of 1-INT-1

12.1 Isotopic Fractionation Studies

To estimate the KIE of deprotonation for arene radical cation **1-INT-1**, isotopic fraction studies were conducted with **1a** with varied ratios of H_2O/D_2O cosolvent.[12]

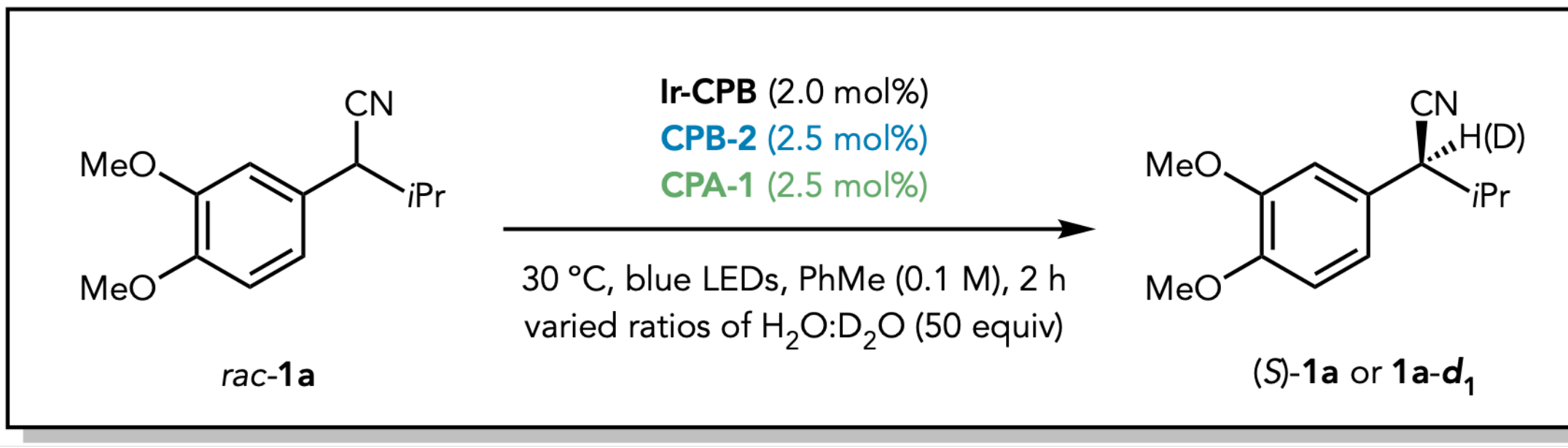

An oven-dried 2-dram vial equipped with a magnetic stir bar was charged with *rac*-**1a** (0.05 mmol, 1.0 equiv), **Ir-CPB** (1.0 µmol, 2.0 mol%), **CPB-2** (1.3 µmol, 2.5 mol%), and **CPA-1** (1.3 µmol, 2.5 mol%). The vial was imported into an N_2 atmosphere glovebox and charged with toluene (0.5 mL, 0.1 M). The vial was sealed with a Teflon septum cap and the junction between the cap and vial wrapped with vinyl electrical tape. The reaction mixture was exported from the glovebox and charged with degassed H_2O:D_2O solution (50 µL, 50 equiv) via Hamilton syringe, then the top of the Teflon septum cap was taped over with vinyl electrical tape. The reaction was then placed in a photoreactor equipped with a recirculating ice-water bath and 456 nm Kessil lamps (PR160L) set to 100% intensity and irradiated for 2 h. Upon completion, the reaction mixture was opened to air and concentrated under reduced pressure. Piperonal (7.5 mg, 0.05 mmol, 1.0 equiv) was added as an internal NMR standard via $CDCl_3$ stock solution and an aliquot was removed for mass balance analysis by ^{1}H NMR spectroscopy. Another aliquot of the reaction was purified by preparative TLC (15% EtOAc/hexanes) and analyzed by ^{1}H NMR spectroscopy for deuterium incorporation. Each experiment was repeated in duplicate, except for the zero point. The data are summarized in **Table S12.1** and visualized in **Figure S12.1**.

Table S12.1. Isotopic fractionation experiments with **1a**.

$[D_2O]/[H_2O]$	Trial 1 [**1a**-d_1]/[**1a**]	Trial 2 [**1a**-d_1]/[**1a**]	Avg. [**1a**-d_1]/[**1a**]
0	0	0	0
0.5	0.37	0.41	0.39
1.0	0.96	0.85	0.91
1.5	1.32	1.22	1.27
2.0	1.86	1.70	1.78

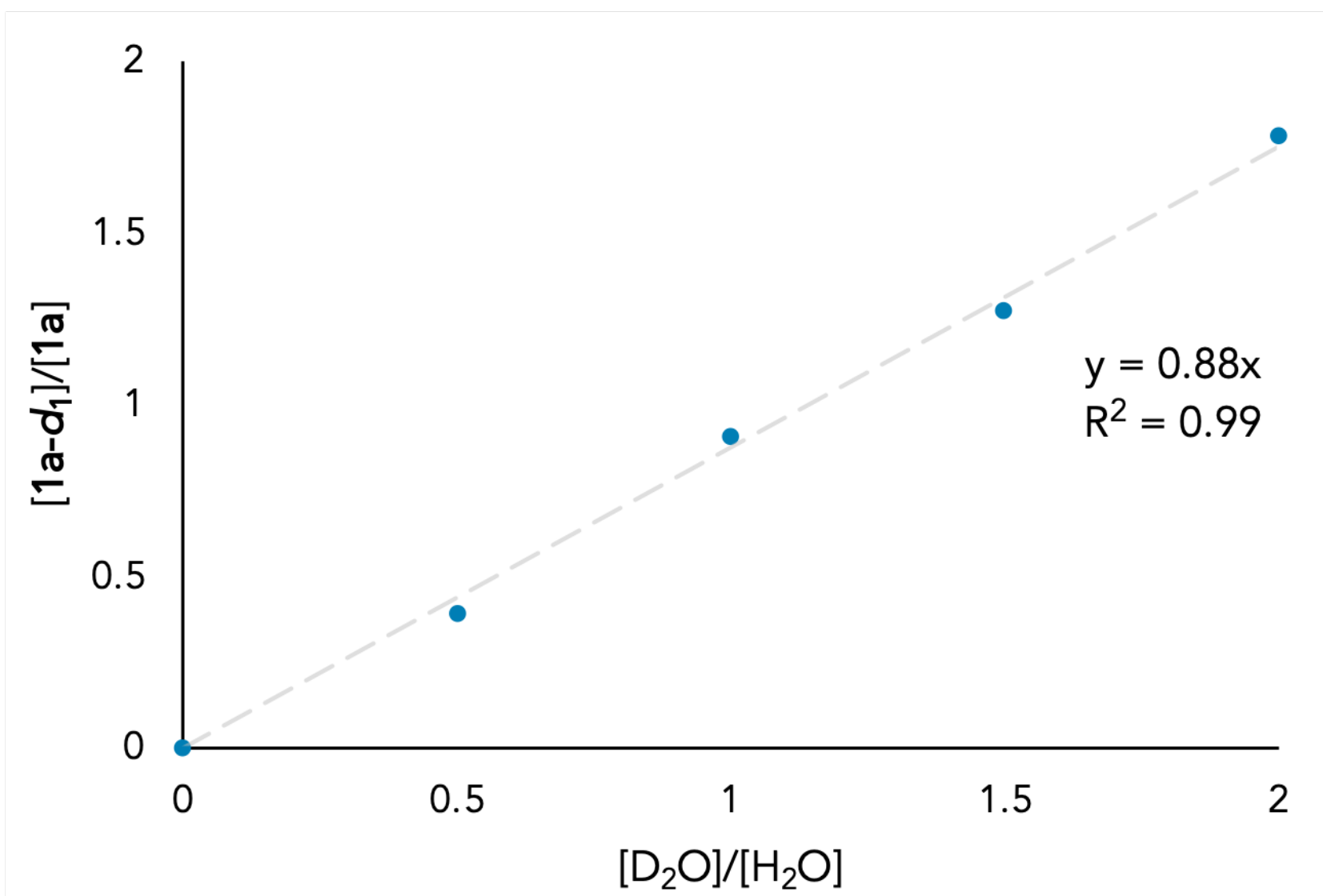

Figure S12.1. Isotopic fractionation plot for **1a**. Fractionation slope determined to be 0.88 (R^2 = 0.99).

12.2 Derivation of KIE-Bounded Enantioselectivity Perturbation

We demonstrate that the magnitude of the isotopic perturbation of enantiomeric ratio is bounded by the kinetic isotope effect (KIE) for deprotonation of **1-INT-1**. For the purpose of the derivation, we assume quantitative isotopic substitution at the benzylic position of **1** when D_2O is used as a cosolvent.

The ratio of the er when D_2O is used as cosolvent (er_D) versus er when H_2O is used as cosolvent (er_H) can be expressed in terms of the photostationary state equation previously derived.[6]

$$\frac{er_D}{er_H} = \frac{k_{-1}[Ir^{II}] + k_{2,S}[CPB]/KIE}{k_{-1}[Ir^{II}] + k_{2,R}[CPB]/KIE} \Big/ \frac{k_{-1}[Ir^{II}] + k_{2,S}[CPB]}{k_{-1}[Ir^{II}] + k_{2,R}[CPB]}$$

Multiplying through by KIE, this expression can be rewritten as,

$$\frac{er_D}{er_H} = \frac{KIE \times k_{-1}[Ir^{II}] + k_{2,S}[CPB]}{KIE \times k_{-1}[Ir^{II}] + k_{2,R}[CPB]} \Big/ \frac{k_{-1}[Ir^{II}] + k_{2,S}[CPB]}{k_{-1}[Ir^{II}] + k_{2,R}[CPB]}$$

Because all of these terms must be positive, demonstrating boundedness is straightforward. We first make the following assumptions, which are reasonable given that KIE ≥ 1,

$$KIE \times k_{-1}[Ir^{II}] + k_{2,S}[CPB] \;\leq\; KIE \times \left(k_{-1}[Ir^{II}] + k_{2,S}[CPB]\right)$$

with equality in the limit of k_{-1}[**Ir^{II}**] >> $k_{2,S}$[**CPB**], and

$$KIE \times k_{-1}[Ir^{II}] + k_{2,R}[CPB] \;\geq\; k_{-1}[Ir^{II}] + k_{2,R}[CPB]$$

with equality in the limit of $k_{2,R}$[**CPB**] >> KIE x k_{-1}[**Ir^{II}**]. Then replacing the numerator with an upper bound and denominator with a lower bound yields the following inequality,

$$\frac{KIE \times k_{-1}[Ir^{II}] + k_{2,S}[CPB]}{KIE \times k_{-1}[Ir^{II}] + k_{2,R}[CPB]} \leq \frac{KIE \times \left(k_{-1}[Ir^{II}] + k_{2,S}[CPB]\right)}{k_{-1}[Ir^{II}] + k_{2,R}[CPB]}$$

and therefore

$$\frac{er_D}{er_H} \leq \frac{KIE \times \left(k_{-1}[Ir^{II}] + k_{2,S}[CPB]\right)}{k_{-1}[Ir^{II}] + k_{2,R}[CPB]} / \frac{k_{-1}[Ir^{II}] + k_{2,S}[CPB]}{k_{-1}[Ir^{II}] + k_{2,R}[CPB]}$$

Cancelling terms yields the following expression, demonstrating that the perturbation of enantioselectivity is bounded by the KIE,

$$\frac{er_D}{er_H} \leq KIE$$

13. Non-linear Effects

13.1 Non-linear Effects with 1a; Standard Conditions

General Procedure

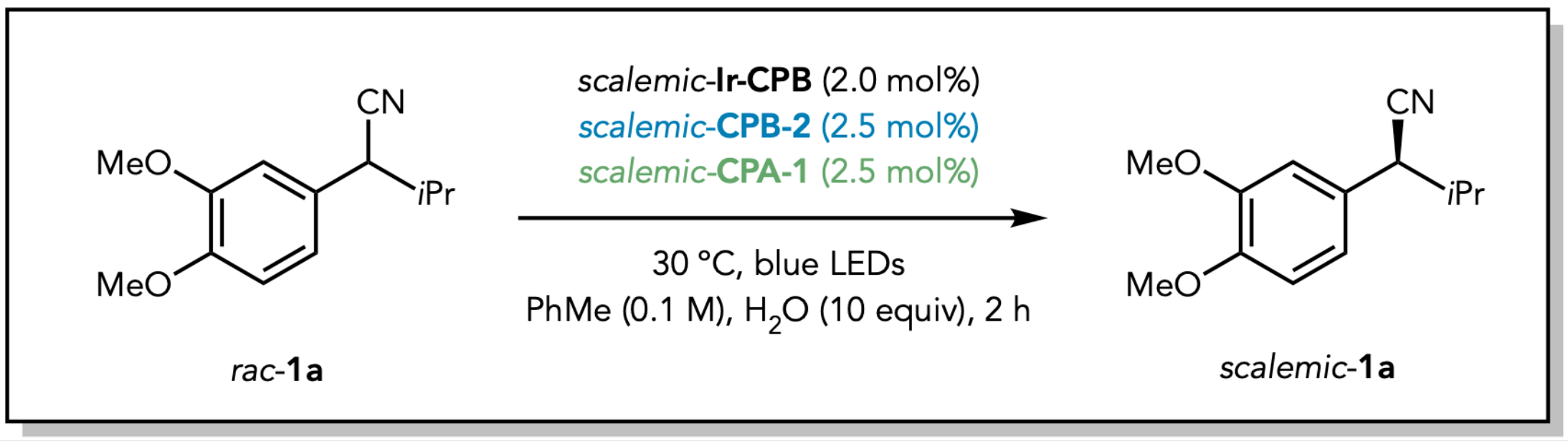

In an oven-dried volumetric flask, a stock solution of **Ir-CPB** (2.0 mM), (*S*)-**CPB-2** (2.5 mM), and (*S)*-**CPA-1** (2.5 mM) in toluene was prepared. An analogous stock solution of the (*R*)-enantiomers of each component was prepared. An oven-dried 2-dram vial equipped with a magnetic stir bar was charged with *rac*-**1a** (11 mg, 0.05 mmol, 1.0 equiv). The vial was imported into an N_2 atmosphere glovebox and charged with the desired ratio of (*S*)- and (*R*)-stock solutions, totaling 500 µL, to afford scalemic catalyst of known ee. The vial was sealed with a Teflon septum cap and the junction between the cap and vial wrapped with vinyl electrical tape. The reaction mixture was exported from the glovebox and charged with degassed H_2O (10 µL, 10 equiv) via Hamilton syringe, then the top of the Teflon septum cap was taped over with vinyl electrical tape. The reaction was then placed in a photoreactor equipped with 456 nm Kessil lamps (PR160L) set to 100% intensity and irradiated for 2 h. Upon completion, the reaction mixture was opened to air and concentrated *in vacuo*. The crude material was redissolved in 500 µL of $CDCl_3$ with piperonal (0.1 M) as an internal standard. Half of the solution was characterized by quantitative ^{1}H NMR, then chiral shift reagent (*R*)-(+)-1-(2-naphthyl)ethylamine (1.0 mg, 5.8 µmol, 12 mol%) and 2.0 M aqueous HCl (50 µL) was added and analyzed by quantitative ^{31}P NMR for catalyst ee.[13] The other half of the solution was purified by preparative thin layer chromatography (15% EtOAc/hexanes) and subsequently analyzed for enantiomeric excess via HPLC (AD-H, 10% isopropyl alcohol in hexanes, 15 min, 230 nm detection).

The following reactions were performed according to the general procedure. The enantiomeric excess of all three chiral catalyst components is reported as a single value obtained from chiral shift quantitative ^{31}P NMR, acknowledging that all chiral phosphate bound ion pairs exist in equilibrium in solution. Catalyst in the (*S*)-configuration is defined to have positive ee.

Table S13.1. ee of **1a** (%) vs. ee of catalyst (%) under standard conditions.

Entry	ee of **catalyst** (%)	ee of **1a** (%)
**A**	2	0
**B**	22	30
**C**	52	62
**D**	82	82
**E**	90	88
**F**	>99	94

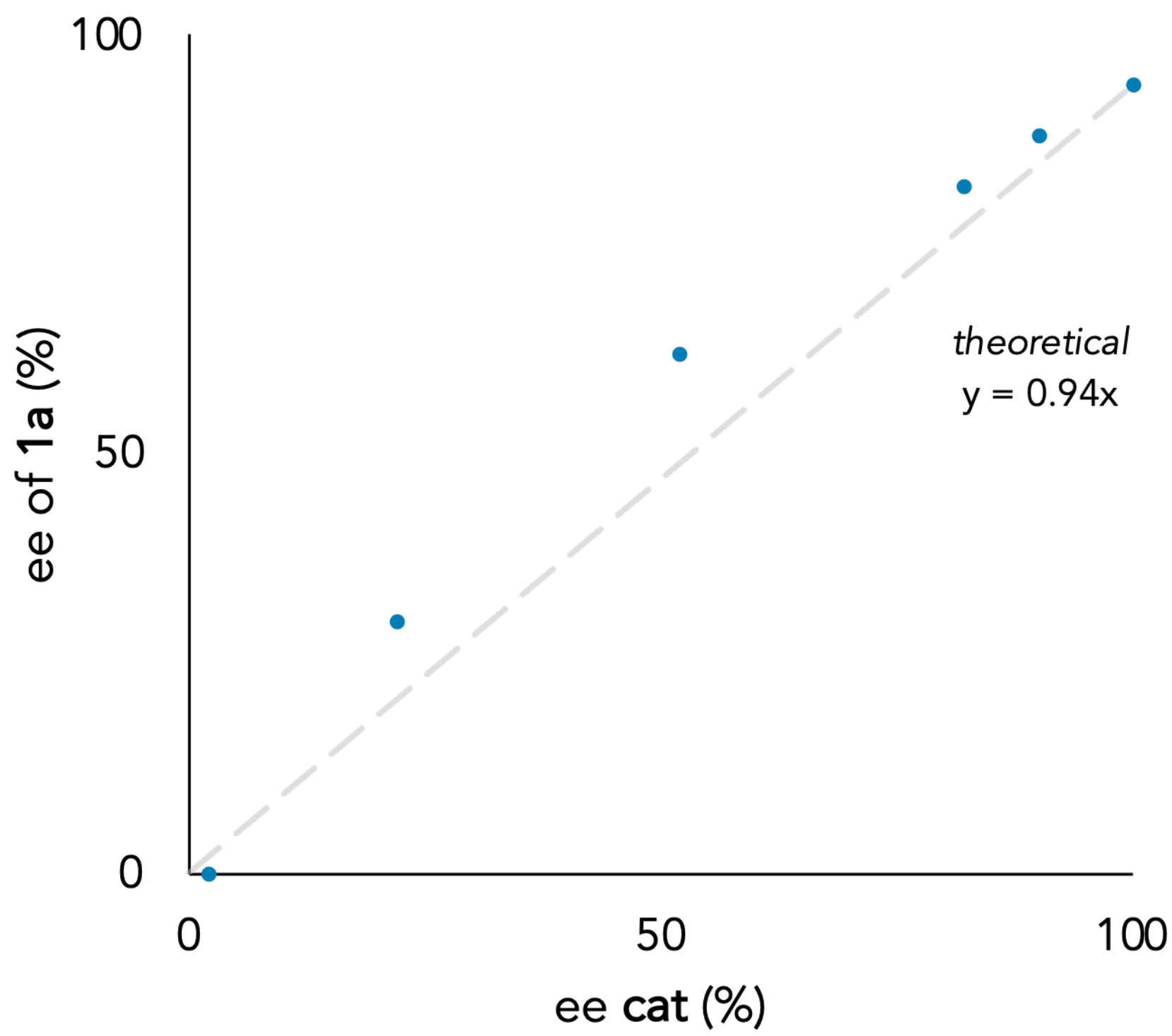

Figure S13.1. ee of **1a** (%) vs. ee of catalyst (%) under standard conditions.

13.2 Non-linear Effects with 1a; Modified Conditions

Given the known dimerization of phosphoric acids in nonpolar solvents, the non-linear effect was investigated in the absence of exogenous **CPA-1**.[15]

General Procedure

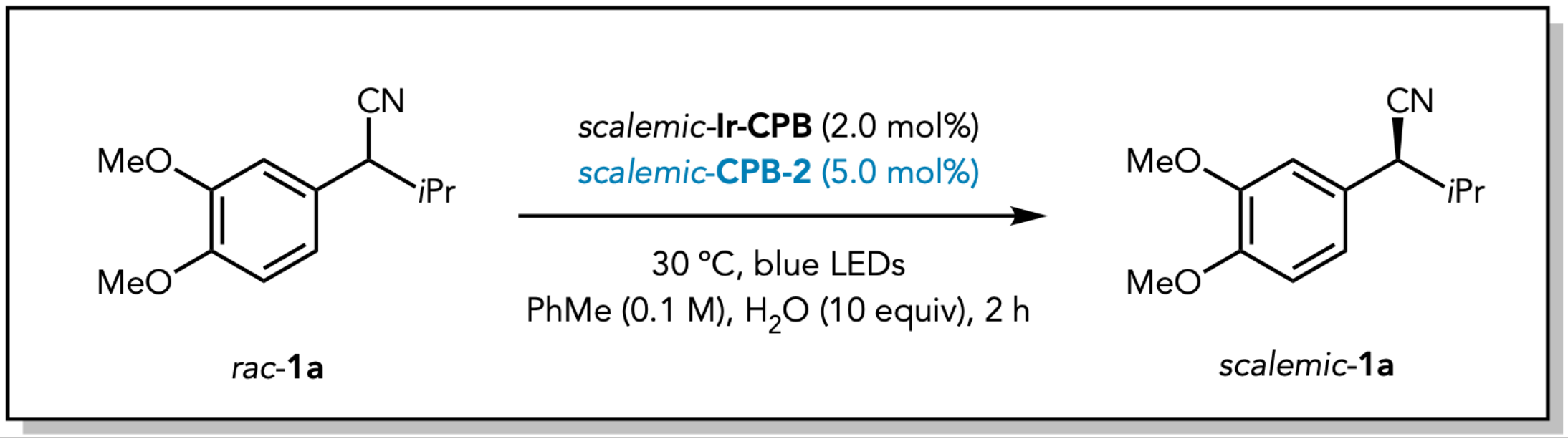

In an oven-dried volumetric flask, a stock solution of **Ir-CPB** (2.0 mM) and (*S*)-**CPB-2** (5.0 mM) in toluene was prepared. An analogous stock solution of the (*R*)-enantiomers of each component was prepared. An oven-dried 2-dram vial equipped with a magnetic stir bar was charged with *rac*-**1a** (11 mg, 0.05 mmol, 1.0 equiv). The vial was imported into an N_2 atmosphere glovebox and charged with the desired ratio of (*S*)- and (*R*)-stock solutions, totaling 500 µL, to afford scalemic catalyst of known ee. The vial was sealed with a Teflon septum cap and the junction between the cap and vial wrapped with vinyl electrical tape. The reaction mixture was exported from the glovebox and charged with degassed H_2O (10 µL, 10 equiv) via Hamilton syringe, then the top of the Teflon septum cap was taped over with vinyl electrical tape. The reaction was then placed in a photoreactor equipped with 456 nm Kessil lamps (PR160L) set to 100% intensity and irradiated for 2 h. Upon completion, the reaction mixture was opened to air and concentrated *in vacuo*. The crude material was redissolved in 500 µL of $CDCl_3$ with piperonal (0.1 M) as an internal standard. Half of the solution was characterized by quantitative ^1H NMR. The other half of the solution was purified by preparative thin layer chromatography (15% EtOAc/hexanes) and subsequently analyzed for enantiomeric excess via HPLC (AD-H, 10% isopropyl alcohol in hexanes, 15 min, 230 nm detection).

The following reactions were performed according to the general procedure. The enantiomeric excess of both chiral catalyst components is reported as a single value. Chiral shift quantitative ^{31}P NMR analysis of catalyst ee was omitted. Catalyst in the (*S*)-configuration is defined to have positive ee.

Table S13.2. ee of **1a** (%) vs. ee of catalyst (%) under modified conditions.

Entry	ee of **catalyst** (%)	ee of **1a** (%)	**1a** yield (%)
**A**	0	0	97
**B**	20	24	98
**C**	40	48	93
**D**	50	58	96
**E**	60	66	95
**F**	70	74	94
**G**	80	80	95
**H**	90	86	91
**A-S**	>99	92	92
**B-R**	<-99	-90	96

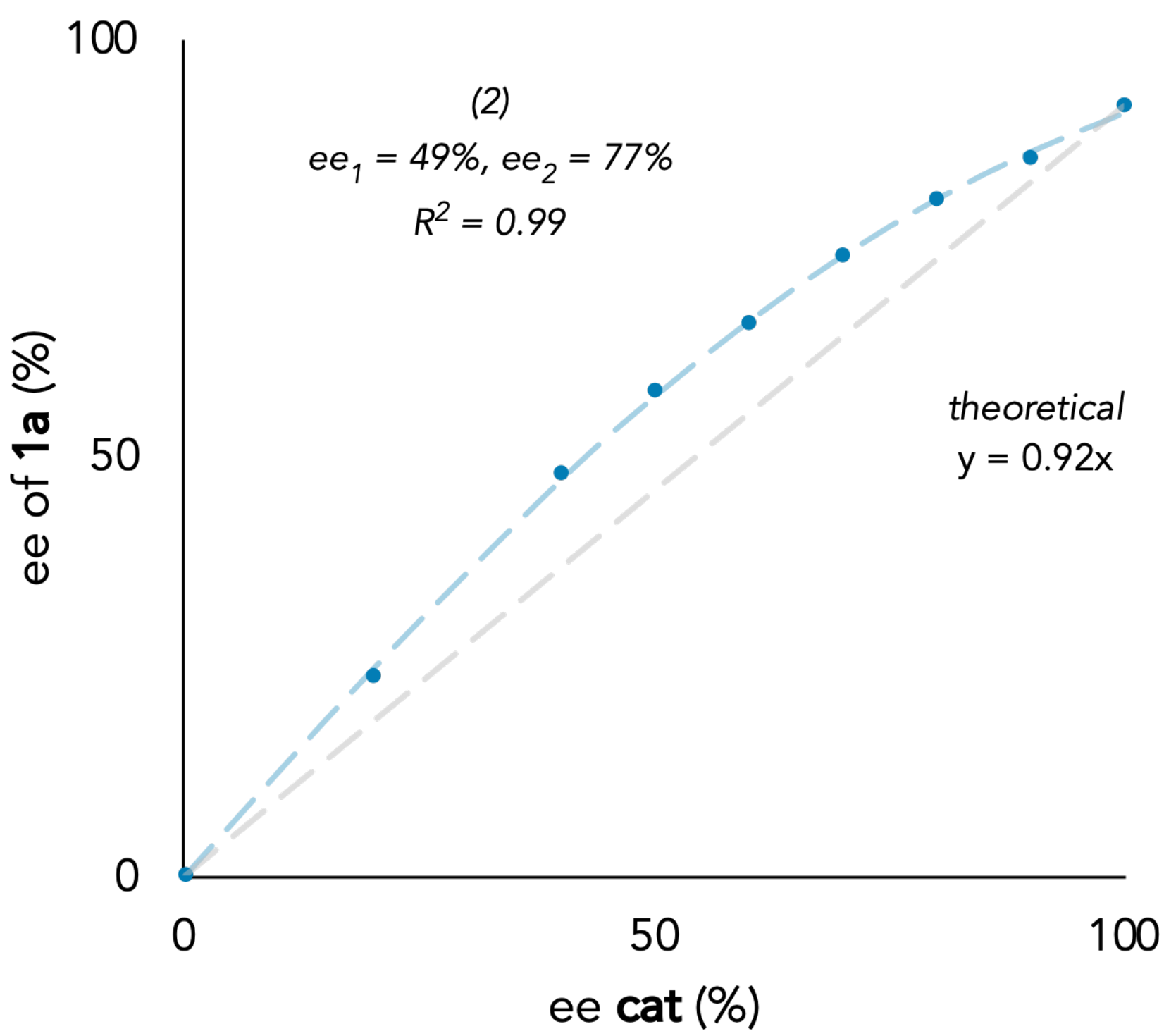

Figure S13.2. ee of **1a** (%) vs. ee of catalyst (%) under modified conditions.

Solver fit to equation (2) gives ee_1 = 49% and ee_2 = 77%, with an R^2 of 0.99 (see S13.5 for derivation), indicated by the dashed blue line in **Figure S13.2**.

13.3 Non-linear Effects with 1a and Ir-10

The non-linear effect with 1a was investigated with a more reducing photocatalyst **Ir-10**.

General Procedure

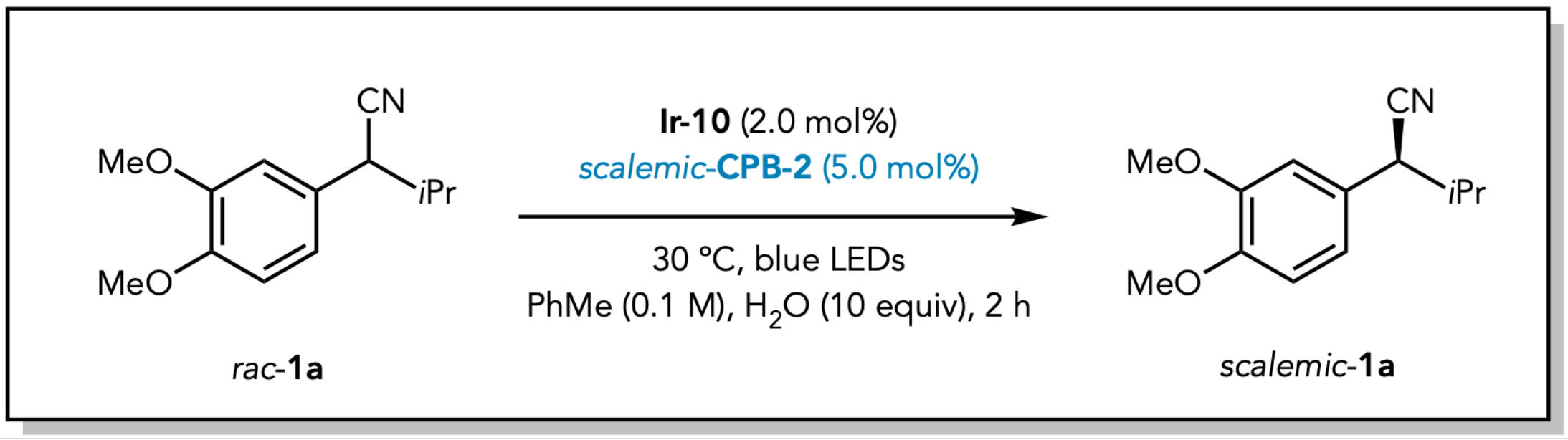

In an oven-dried volumetric flask, a stock solution of **Ir-10** (2.0 mM) and (*S*)-**CPB-2** (5.0 mM) in toluene was prepared. An analogous stock solution of **Ir-10** (2.0 mM) and (*R*)-**CPB-2** (5.0 mM) in toluene was prepared. An oven-dried 2-dram vial equipped with a magnetic stir bar was charged with *rac*-**1a** (11 mg, 0.05 mmol, 1.0 equiv). The vial was imported into an N_2 atmosphere glovebox and charged with the desired ratio of (*S*)- and (*R*)-stock solutions, totaling 500 µL, to afford scalemic catalyst of known ee. The vial was sealed with a Teflon septum cap and the junction between the cap and vial wrapped with vinyl electrical tape. The reaction mixture was exported from the glovebox and charged with degassed H_2O (10 µL, 10 equiv) via Hamilton syringe, then the top of the Teflon septum cap was taped over with vinyl electrical tape. The reaction was then placed in a photoreactor equipped with 456 nm Kessil lamps (PR160L) set to 100% intensity and irradiated for 2 h. Upon completion, the reaction mixture was opened to air and concentrated *in vacuo*. The crude material was redissolved in 500 µL of $CDCl_3$ with piperonal (0.1 M) as an internal standard. Half of the solution was characterized by quantitative ^{1}H NMR. The other half of the solution was purified by preparative thin layer chromatography (15% EtOAc/hexanes) and subsequently analyzed for enantiomeric excess via HPLC (AD-H, 10% isopropyl alcohol in hexanes, 15 min, 230 nm detection).

The following reactions were performed according to the general procedure.

Table S13.3. ee of **1a** (%) vs. ee of **CPB-2** (%) with **Ir-10** as photocatalyst.

Entry	ee of **catalyst** (%)	ee of **1a** (%)	**1a** yield (%)
**A**	0	0	93
**B**	25	18	89
**C**	50	34	93
**D**	75	48	92
**E**	>99	70	88

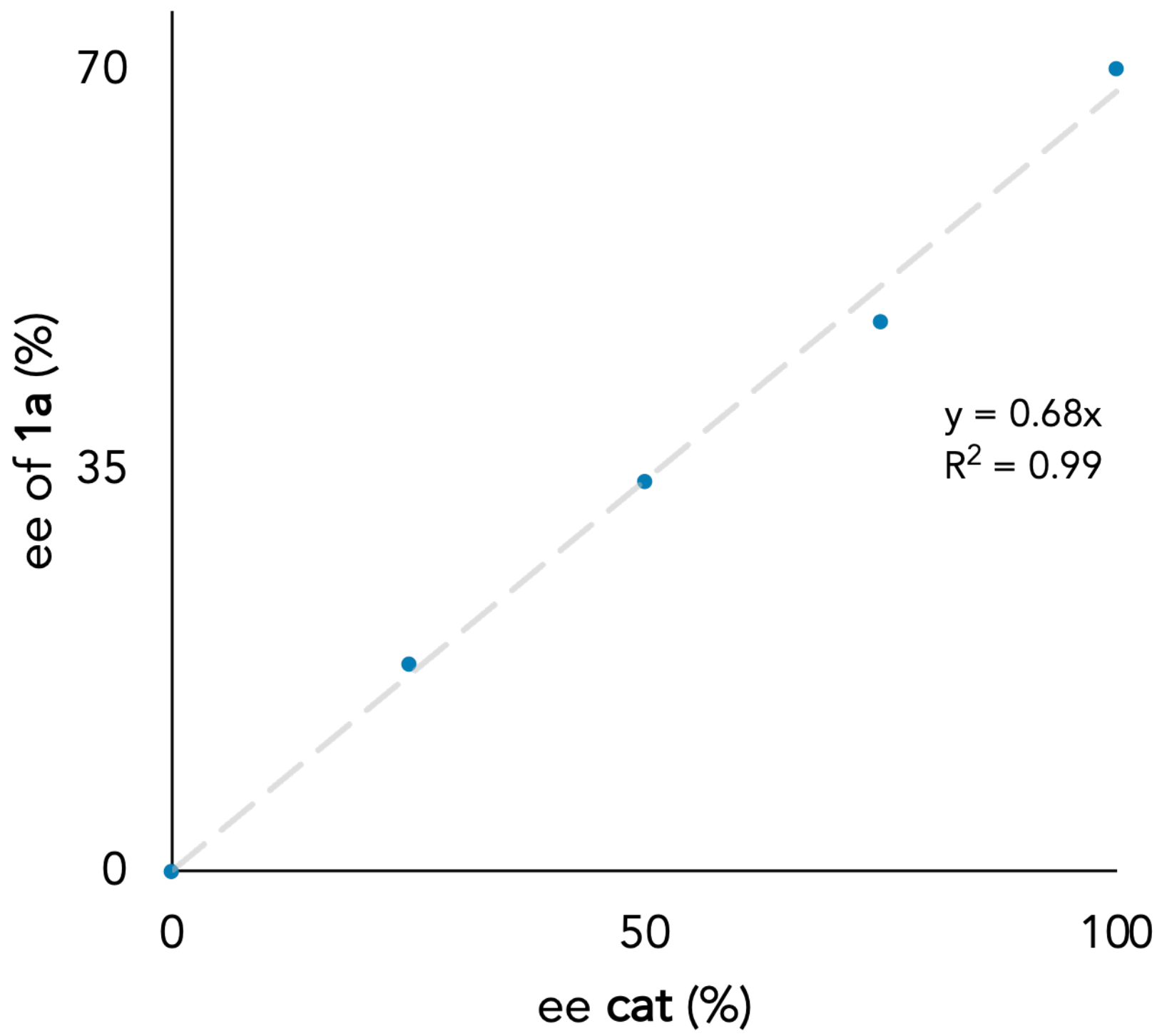

Figure S13.3 ee of **1a** (%) vs. ee of **CPB-2** (%) with **Ir-10** as photocatalyst.

13.4 Non-linear Effects with 2; Modified Conditions

The (non)-linear effect in C–H bond formation was investigated with mechanistic probe **2**.

General Procedure

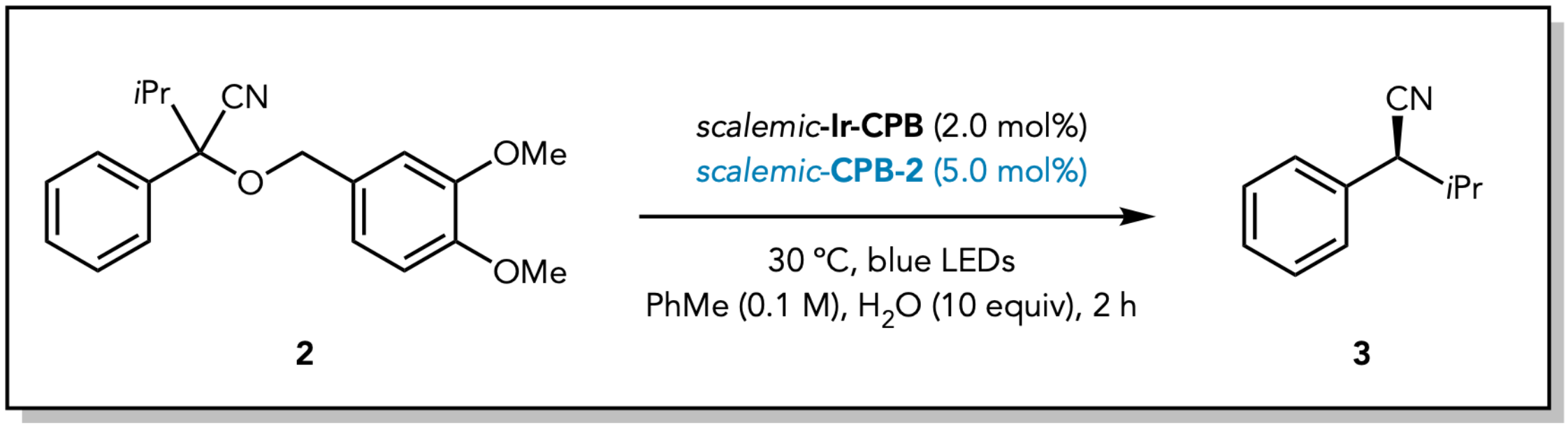

In an oven-dried volumetric flask, a stock solution of **Ir-CPB** (2.0 mM) and (*S*)-**CPB-2** (5.0 mM) in toluene was prepared. An analogous stock solution of the (*R*)-enantiomers of each component was prepared. An oven-dried 2-dram vial equipped with a magnetic stir bar was charged with **2** (16 mg, 0.05 mmol, 1.0 equiv). The vial was imported into an N_2 atmosphere glovebox and charged with the desired ratio of (*S*)- and (*R*)-stock solutions, totaling 500 µL, to afford scalemic catalyst of known ee. The vial was sealed with a Teflon septum cap and the junction between the cap and vial wrapped with vinyl electrical tape. The reaction mixture was exported from the glovebox and charged with degassed H_2O (10 µL, 10 equiv) via Hamilton syringe, then the top of the Teflon septum cap was taped over with vinyl electrical tape. The reaction was then placed in a photoreactor equipped with 456 nm Kessil lamps (PR160L) set to 100% intensity and irradiated for 2 h. Upon completion, the reaction mixture was opened to air and concentrated *in vacuo*. The crude material was redissolved in 500 µL of $CDCl_3$ with piperonal (0.1 M) as an internal standard. Half of the solution was characterized by quantitative ^{1}H NMR. The other half of the solution was purified by preparative thin layer chromatography (15% EtOAc/hexanes) and subsequently analyzed for enantiomeric excess via HPLC (OJ-H, 10% isopropyl alcohol in hexanes, 10 min, 210 nm detection).

The following reactions were performed according to the general procedure.

Table S13.4. ee of **3** (%) vs. ee of catalyst (%) under modified conditions.

Entry	ee of **catalyst** (%)	ee of **3** (%)	**3** yield (%)
**A**	0	0	97
**B**	25	17	96
**C**	50	30	95
**D**	75	45	89
**E**	>99	62	98

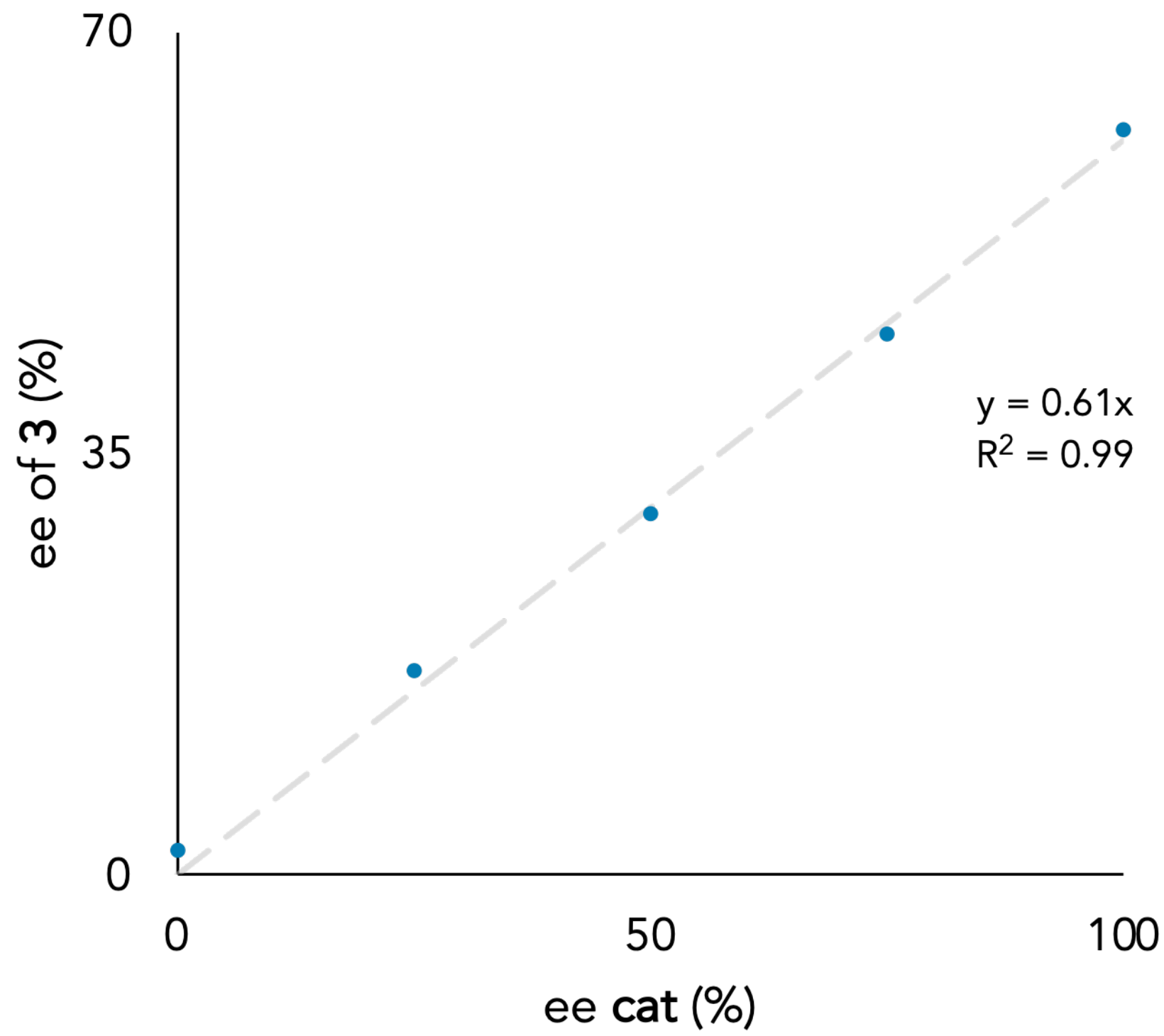

Figure S13.4. ee of **3** (%) vs. ee of catalyst (%) under modified conditions.

13.5 Derivation of a General Equation for the Non-linear Effect in Deracemization Reactions with Two Selective Steps

The following equation was derived to describe the observation of a non-linear effect in deracemization reactions with two selective steps in which the same chiral catalyst acts on the same stereocenter.

13.5.1 Definitions and Assumptions

The enantiomeric excess (ee) and enantiomer ratio (er) are related by,

$$ee = \frac{er - 1}{er + 1}$$

$$er = \frac{1 + ee}{1 - ee}$$

Let *i* be a single step. Assuming stepwise linearity under the NLE model,

$$ee_{NLE,i} = ee_i \cdot ee_{cat}$$

Therefore,

$$er_{NLE,i} = \frac{1 + ee_{NLE,i}}{1 - ee_{NLE,i}} = \frac{1 + ee_i \cdot ee_{cat}}{1 - ee_i \cdot ee_{cat}}$$

13.5.2 Overall er

The overall enantiomer ratio (er) of the run is the product of the stepwise er,

$$er_{NLE,f} = \prod_{i=1}^{n} er_{NLE,i}$$

In the case of two steps,

$$er_{NLE,f} = \frac{1 + ee_1 \cdot x}{1 - ee_1 \cdot x} \cdot \frac{1 + ee_2 \cdot x}{1 - ee_2 \cdot x}$$

where $x = ee_{cat}$. Expanding the numerator yields,

$$(1 + ee_1 \cdot x)(1 + ee_2 \cdot x) = 1 + (ee_1 + ee_2) \cdot x + ee_1 \cdot ee_2 \cdot x^2$$

Expanding the denominator,

$$(1 - ee_1 \cdot x)(1 - ee_2 \cdot x) = 1 - (ee_1 + ee_2) \cdot x + ee_1 \cdot ee_2 \cdot x^2$$

At this point it becomes convenient to make some algebraic simplifications. Define the shorthand,

$$S = ee_1 + ee_2,\ P = ee_1 \cdot ee_2$$

Where S is the sum of fractional ee and P is the product. Then,

$$er_{NLE,f} = \frac{1 + S \cdot x + P \cdot x^2}{1 - S \cdot x + P \cdot x^2}$$

13.5.3 Converting er to ee

The overall NLE ee is,

$$ee_{NLE,f} = \frac{er_{NLE,f} - 1}{er_{NLE,f} + 1}$$

We compute the numerator first,

$$er_{NLE,f} - 1 = \frac{1 + S \cdot x + P \cdot x^2}{1 - S \cdot x + P \cdot x^2} - 1$$

$$= \frac{2 \cdot S \cdot x}{1 - S \cdot x + P \cdot x^2}$$

We next compute the denominator,

$$er_{NLE,f} + 1 = \frac{1 + S \cdot x + P \cdot x^2}{1 - S \cdot x + P \cdot x^2} + 1$$

$$= \frac{2 + 2 \cdot P \cdot x^2}{1 - S \cdot x + P \cdot x^2}$$

Taking the quotient and cancelling like denominators yields,

$$ee_{NLE,f} = \frac{S \cdot x}{1 + P \cdot x^2}$$

Substituting S and P yields the final expression, equation (**2**)

$$ee_{NLE,f} = \frac{ee_{cat} \cdot (ee_1 + ee_2)}{1 + ee_1 . ee_2 \cdot ee_{cat}^2}$$

Blackmond and Fu have reported non-canonical NLEs in similar cases previously.[16, 17]

A second derivative test on eq (**2**) demonstrates negative curvature (positive NLE) in the matched case, where the selectivity of each individual step mediated by the chiral catalyst is biased towards formation of the same major enantiomer.

14. Determination of Initial Rates and Catalyst Order

Initial rates were determined by varying catalyst loadings in low-conversion experiments, starting from enantiopure (*R*)-**1a** and monitoring conversion to (*S*)-**1a** by LC-SFC.

General Procedure for Photocatalyst Initial Rates

An oven-dried 2-dram vial equipped with a magnetic stir bar was charged with (*R*)-**1a** (0.1 mmol, 1.0 equiv), **Ir-CPB** (0.5–2.0 μmol, 0.25–2.0 mol%, specified per experiment) and **CPB-2** (2.5 μmol, 2.5 mol%). The vial was imported into an N_2 atmosphere glovebox and charged with toluene (0.50 mL, 0.20 M). The vial was sealed with a Teflon septum cap and the junction between the cap and vial wrapped with vinyl electrical tape. The reaction mixture was exported from the glovebox and placed under positive N_2 pressure. Each vial was charged with degassed H_2O (20 μL, 10 equiv) via Hamilton syringe. The reaction was then placed in a photoreactor equipped with two rotary fans and 456 nm Kessil lamps (PR160L) set to 100% intensity and irradiated for four 3 minute intervals (total irradiation time: 12 minutes). After each interval, a 20 μL aliquot was removed from the reaction and the reaction vial was placed back under irradiation. The aliquot was dissolved in 1.0 mL acetonitrile and the optical purity was measured by 2D LC-SFC (**LC**: Agilent SB-C18 (1.8 μm particle size, 2.1 × 50 mm), 0–100% water/acetonitrile, 5.50 min, 1.0 mL/min flow rate, 210 nm detection, t_r **1a** = 3.12–3.25 min; collected onto the SFC; **SFC**: Daicel ChiralPak® AD-3 (3 μm particle size, 4.6 × 250 mm); 10% isopropyl alcohol:CO_2; 0.5 mL/min flow rate): t_R(major) = 6.11 min; t_R(minor) = 6.52 min).

General Procedure for Chiral Phosphate Base Initial Rates

An oven-dried 2-dram vial equipped with a magnetic stir bar was charged with (*R*)-**1a** (0.1 mmol, 1.0 equiv), **Ir-CPB** 1.0 μmol, 1.0 mol%) and (*S*)-**CPB-2** (0.625–6.25 μmol, 0.625–6.25 mol%). The vial was imported into an N_2 atmosphere glovebox and charged with toluene (0.50 mL, 0.20 M). The vial was sealed with a Teflon septum cap and the junction between the cap and vial wrapped with vinyl electrical tape. The reaction mixture was exported from the glovebox and placed under positive N_2 pressure. Each vial was charged with degassed H_2O (20 μL, 10 equiv) via Hamilton syringe. The reaction was then placed in a photoreactor equipped with two rotary fans and 456 nm Kessil lamps (PR160L) set to 100% intensity and irradiated for six 3 minute intervals (total irradiation time: 18 minutes). After each interval, a 20 μL aliquot was removed from the reaction and the reaction vial was placed back under irradiation. The aliquot was dissolved in 1.0 mL acetonitrile and the optical purity was measured by 2D LC-SFC (**LC**: Agilent SB-C18 (1.8 μm particle size, 2.1 × 50 mm), 0–100% water/acetonitrile, 5.50 min, 1.0 mL/min flow rate, 210 nm detection, t_r **1a** = 3.12–3.25 min; collected onto the SFC; **SFC**: Daicel ChiralPak® AD-3 (3 μm particle size, 4.6 × 250 mm); 10% isopropyl alcohol:CO_2; 0.5 mL/min flow rate): t_R(major) = 6.11 min; t_R(minor) = 6.52 min).

Note: Total number of irradiation intervals per catalyst were set such that experiments remained within low-conversion (linear conversion regimes; <20% average conversion) windows.

14.1 Kinetic order of Ir-CPB

Table S14.1. Results of the initial rates experiment to determine the kinetic order of **Ir-CPB**.

**Ir-CPB** loading	0.25 mol%<br>0.5 mM	0.5 mol%<br>1.0 mM	1 mol%<br>2.0 mM	2 mol%<br>4.0 mM
Time (min)	[(*S*)-**1a**] (M)	[(*S*)-**1a**] (M)	[(*S*)-**1a**] (M)	[(*S*)-**1a**] (M)
0	0.0000	0.0000	0.0000	0.0000
3	0.0050	0.0100	0.0200	0.0550
6	0.0066	0.0162	0.0332	0.0854
9	0.0096	0.0218	0.0432	0.1192
12	0.0100	0.0236	0.0616	0.1406
Initial rate (M/min)	0.0010	0.0022	0.0051	0.0127
R^2	0.9677	0.9425	0.9876	0.9728

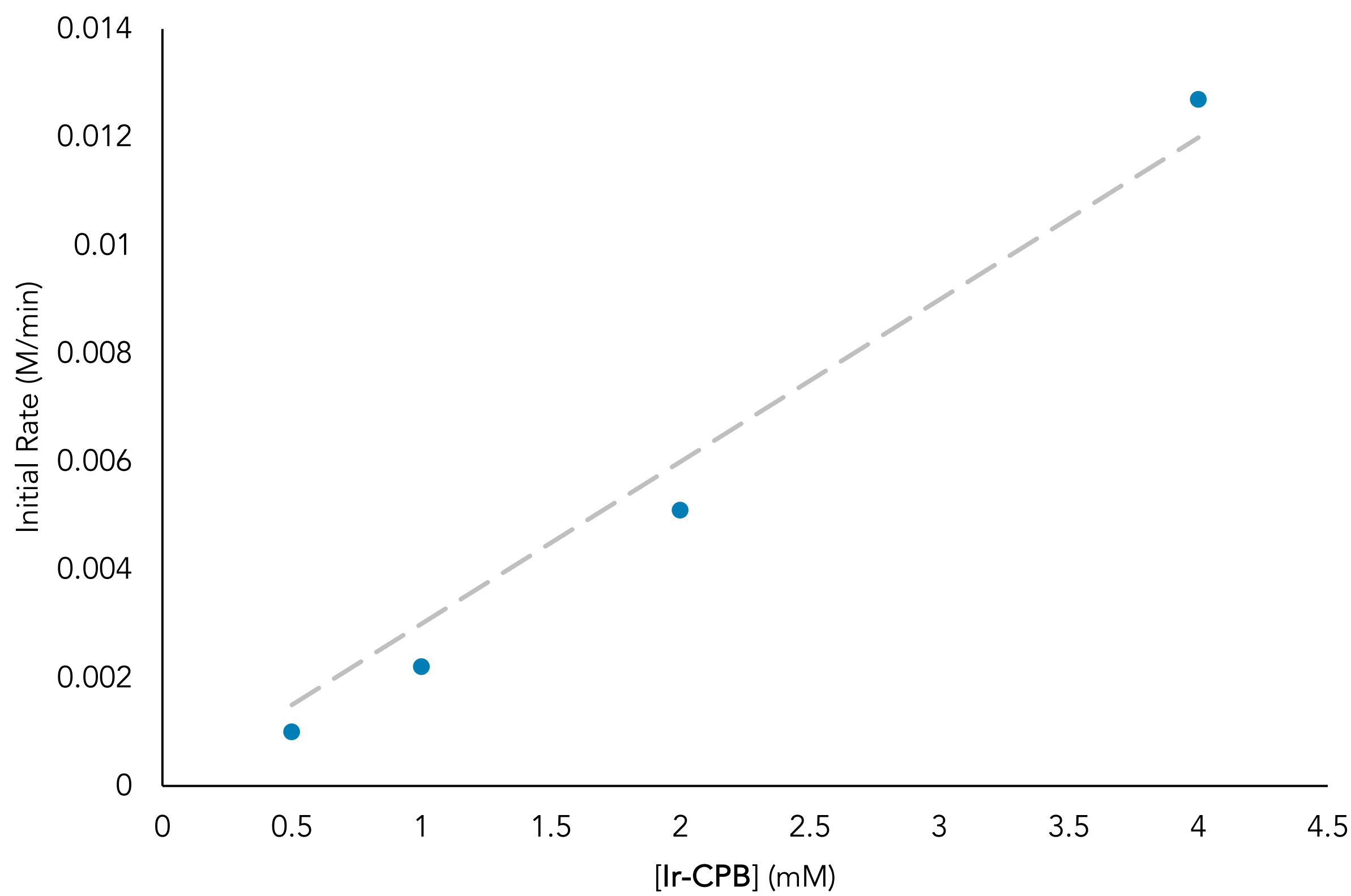

Figure S14.1. First-order dependence of the initial rate on the concentration of **Ir-CPB**.

14.2 Kinetic order of CPB-2

Table S14.2. Results of the initial rates experiment to determine the kinetic order of **CPB-2**.

**CPB-2** loading	0.625 mol% 1.25 mM	1.25 mol% 2.5 mM	2.5 mol% 5.0 mM	3.75 mol% 7.5 mM	5 mol% 10.0 mM	6.25 mol% 12.5 mM
Time (min)	[(*S*)-**1a**] (M)	[(*S*)-**1a**] (M)	[(*S*)-**1a**] (M)	[(*S*)-**1a**] (M)	[(*S*)-**1a**] (M)	[(*S*)-**1a**] (M)
0	0.0000	0.0000	0.0000	0.0000	0.0000	0.0000
3	0.0068	0.0068	0.0068	0.0068	0.0068	0.0068
6	0.0132	0.0122	0.0118	0.0106	0.0122	0.0146
9	0.0184	0.0158	0.0182	0.0182	0.0158	0.0216
12	0.0234	0.0226	0.0236	0.0250	0.0226	0.0274
15	0.0286	0.0284	0.0298	0.0300	0.0284	0.0346
18	0.0334	0.0316	0.0324	0.0352	0.0316	0.0400
Initial rate (M/min)	0.0019	0.0018	0.0018	0.0021	0.0020	0.0023
R^2	0.9957	0.9935	0.9928	0.9989	0.9958	0.9979

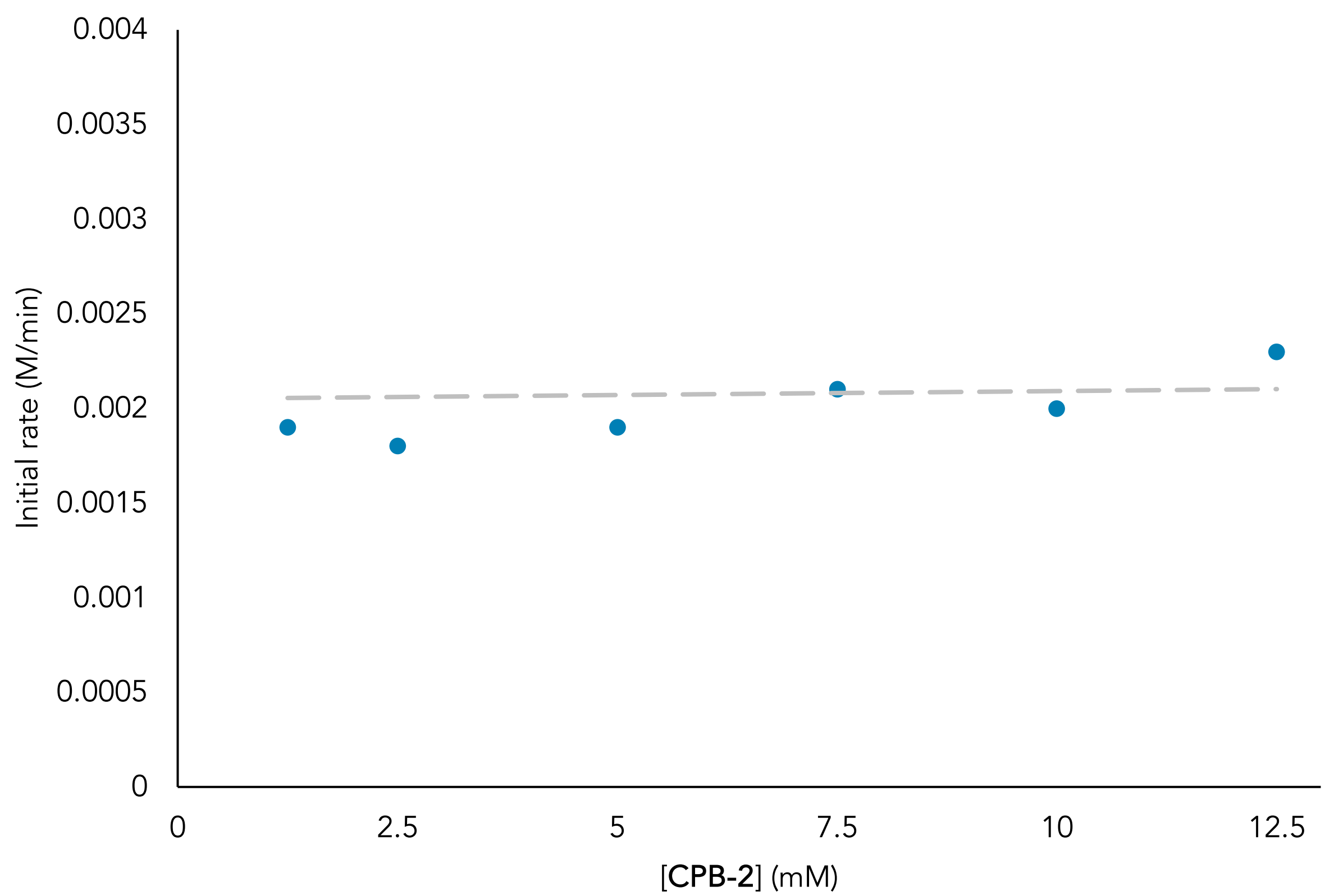

Figure S14.2. Zero-order dependence of the initial rate on the concentration of **CPB-2**.

14.3 Determination of the Effect of Water on Initial Rates

Procedure for Initial Rate Experiment Without Water

To an oven-dried 2-dram vial equipped with a magnetic stir bar was charged (*R*)-**1a** (0.1 mmol, 1.0 equiv), **Ir-CPB** (1.0 mol%) and **CPB-2** (2.5 mol%). The vial was imported into an N_2 atmosphere glovebox and charged with toluene (0.50 mL, 0.20 M). The vial was sealed with a Teflon septum cap and the junction between the cap and vial wrapped with vinyl electrical tape. The reaction mixture was exported from the glovebox and placed under positive N_2 pressure. The reaction was then placed in a photoreactor equipped with two rotary fans and 456 nm Kessil lamps (PR160L) set to 100% intensity, and irradiated for four 3 minute intervals (total irradiation time: 12 minutes). After each interval, a 20 µL aliquot was removed from the reaction and the reaction vial was placed back under irradiation. The aliquot was dissolved in 1.0 mL acetonitrile and the optical purity was measured by 2D LC-SFC (**LC**: Agilent SB-C18 (1.8 µm particle size, 2.1 × 50 mm), 0–100% water/acetonitrile, 5.50 min, 1.0 mL/min flow rate, 210 nm detection, t_r **1a** = 3.12–3.25 min; collected onto the SFC; **SFC**: Daicel ChiralPak® AD-3 (3 µm particle size, 4.6 × 250 mm); 10% isopropyl alcohol:CO_2; 0.5 mL/min flow rate): t_R(major) = 6.11 min; t_R(minor) = 6.52 min).

Table S14.3. Initial rate experiments to determine the effect of water on the reaction.

**water included?**	Yes[a]	No
Time (min)	[(*S*)-**1a**] (M)	[(*S*)-**1a**] (M)
0	0.0000	0.0000
3	0.0200	0.0090
6	0.0332	0.0136
9	0.0432	0.0186
12	0.0616	0.0260
Initial rate (M/min)	0.0051	0.0020
R^2	0.9876	0.9812

[a] Data from the first column was obtained from the set of experiments from which photocatalyst reaction order was determined.

15. Photocatalyst Excited-State Lifetime Measurements

The excited-state lifetimes of **Ir-1** and **Ir-CPB** were determined using time-correlated single photon counting (TCSPC). 2.5×10^{-5} M solutions of the photocatalysts were prepared in dioxane[3] and toluene, respectively, in quartz cuvettes under N_2 atmosphere. The lifetime of **Ir-1** was fitted with a single exponential while the lifetimes of **Ir-CPB** were fitted with a biexponential, presumably due to slight quenching of the iridium lifetime by the ion-paired phosphate base.[18]

The excited-state lifetime decay curves were measured using a Horiba Fluorolog-3 instrument. The sample was excited with unpolarized 445 nm light (NanoLED-450, Horiba; 1.2 ns pulse width) filtered through a vertical polarizer. The time-resolved emission was collected at 90° to the excitation beam under magic angle conditions with the monochromator centered at 550 nm (8-15 nm slit-width). The samples were kept at a temperature of 20°C. The instrument response function (IRF) was measured under the same settings, except that the monochromator was centered at 450 nm to collect light scattered from 20 nm diameter silica nanoparticles.

Separately, the IRF was fit to an empirical model $g(t)$and normalized to a one-photon probability density, $g^{(1)}(t)$. Then, $g^{(1)}(t)$ was convolved with a model mono- or bi-exponential decay model, $f^{(1)}(t)$, also normalized to a one-photon probability density. Parameters involved in the model decay were fit using MATLAB's built-in lsqnonlin() and MultiStart() functions. Time zero and the background before and after time zero were also fit during fitting of the decay model. The model time trace for comparison with experimental data was, $y(t) = N_{sig}\left(g^{(1)} * f^{(1)}\right)(t) + I_{bkg}(t)$, where N_{sig} is the total number of photons and $f^{(1)}(t)$ was either $f^{(1)}(t) = \frac{\exp\left(-\frac{t}{\tau}\right)}{\tau}$ or $f^{(1)}(t) = \frac{a_1 \exp\left(-\frac{t}{\tau_1}\right)}{\tau_1} + \frac{(1-a_1)\exp\left(-\frac{t}{\tau_2}\right)}{\tau_2}$.

The lifetime of **Ir-1** in dioxane was measured to be 1370 ns. The biexponential lifetime of **Ir-CPB** in toluene was measured to be 1183 ns (weighted average).

The lifetime of **Ir-1** was measured in dioxane for comparison and standardization to previous measurements. Additionally, **Ir-1** is sparingly soluble in toluene in the absence of **CPB**.

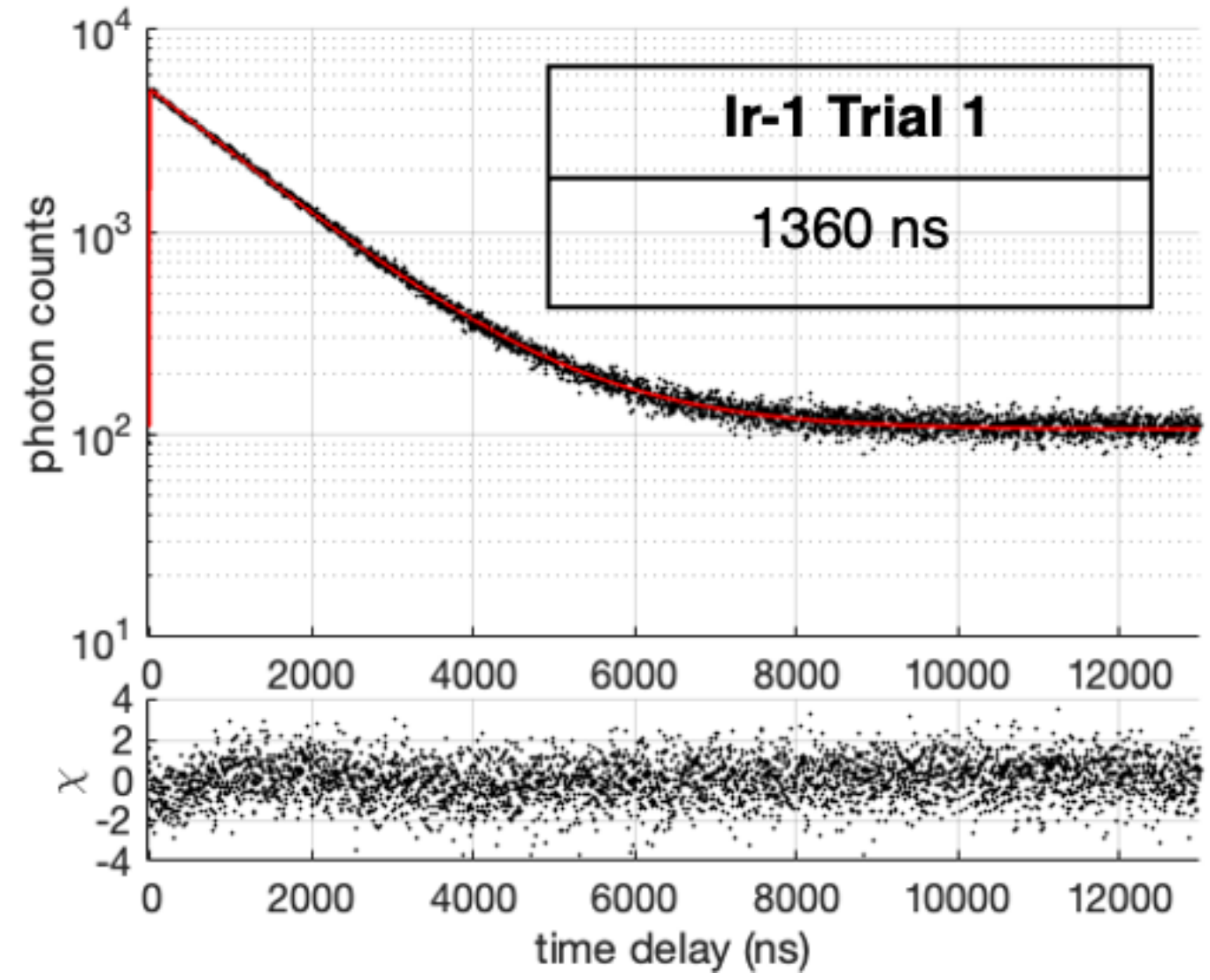

Figure S15.1 Monoexponential lifetime of **Ir-1** measured in dioxane – trial 1 of 2.

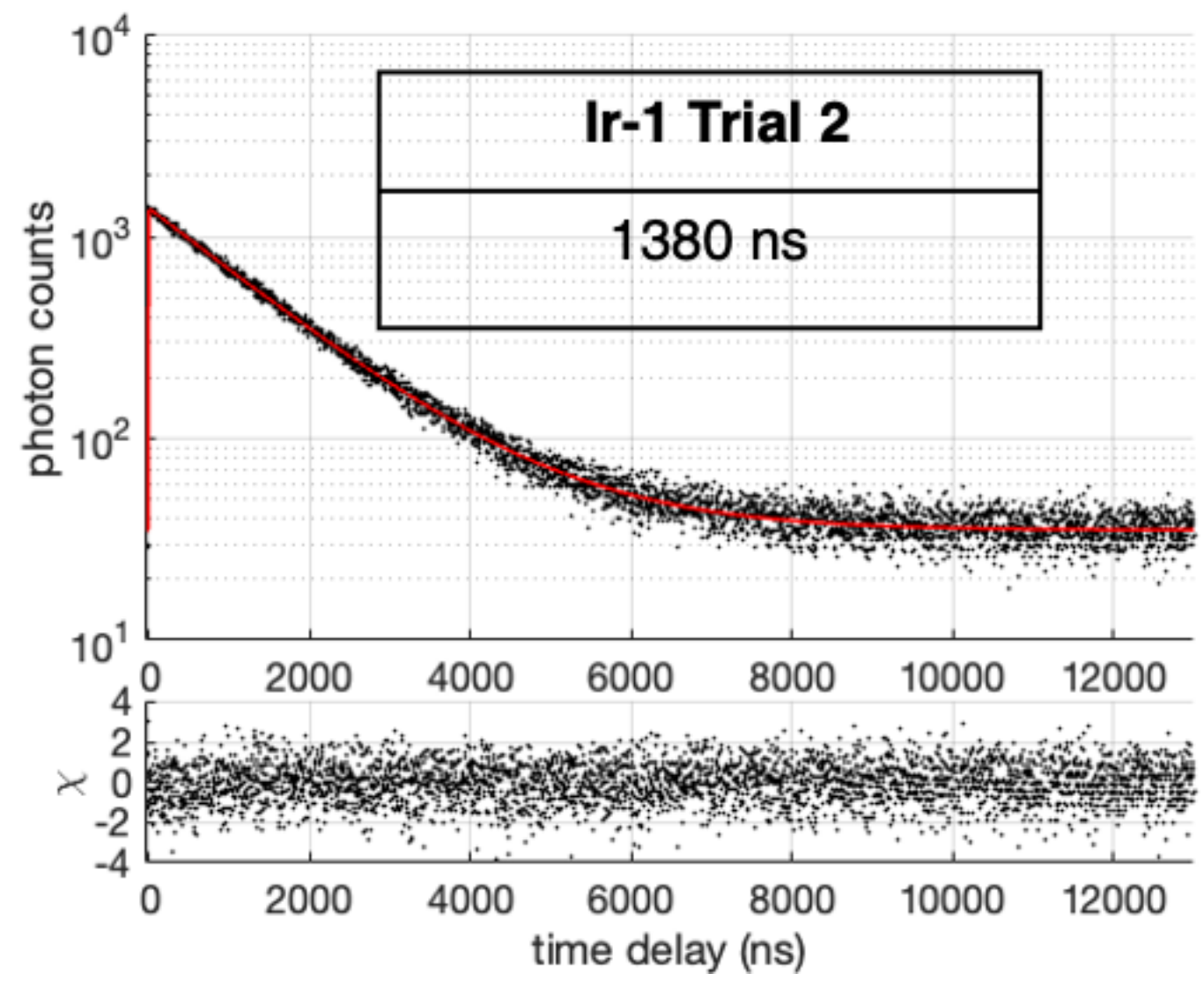

Figure S15.2 Monoexponential lifetime of **Ir-1** measured in dioxane – trial 2 of 2.

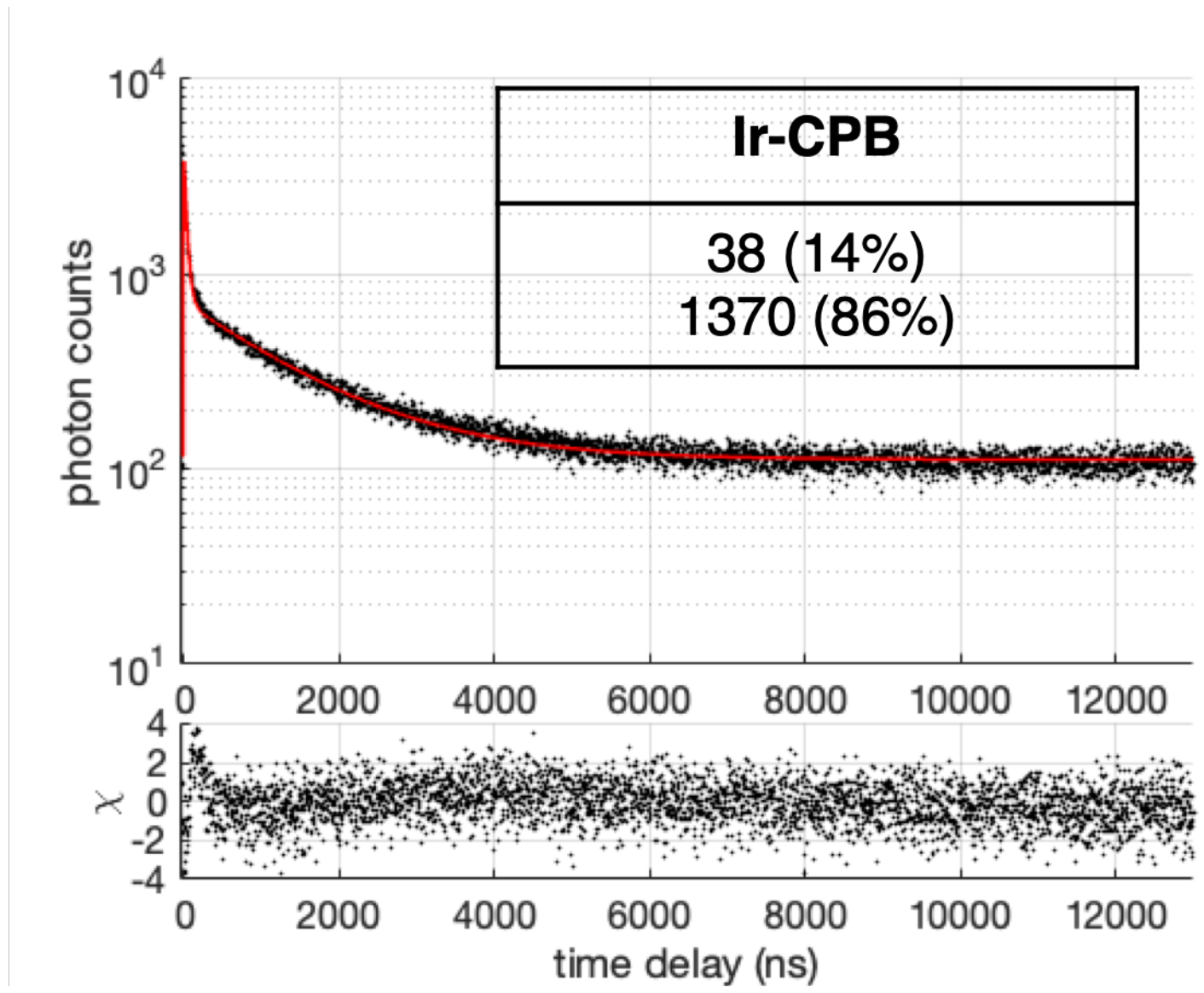

Figure S15.3 Biexponential lifetime of **Ir-CPB** measured in toluene.

16. Stern-Volmer Luminescence Quenching Experiments and Photocatalyst-Substrate Association

Steady state Stern–Volmer luminescence quenching experiments were conducted using an Edinburgh Instruments FLS1000 spectrometer with the Edinburgh Instruments Fluoracle® software. Quenching experiments were performed with freshly prepared, rigorously degassed 2.5 × 10^{-5} M solutions of **Ir-CPB** in toluene in quartz cuvettes at room temperature under nitrogen atmosphere. The solutions were irradiated at 456 nm, and luminescence was measured at 514 nm. The luminescence intensity was measured three times for each sample and averaged. I_0/I values per run were generated from the average of all three measurements and used to obtain K_{SV}. Linear regression of I_0/I as a function of quencher concentration to yield K_{SV} was performed using Microsoft Excel. Dynamic quenching of each photocatalyst with *rac*-**1a** was obfuscated by static quenching within steady state time scales. The ground state association constant between **Ir-CPB** and *rac*-**1a** was measured in C_6D_6. The data are tabulated below and graphically summarized.

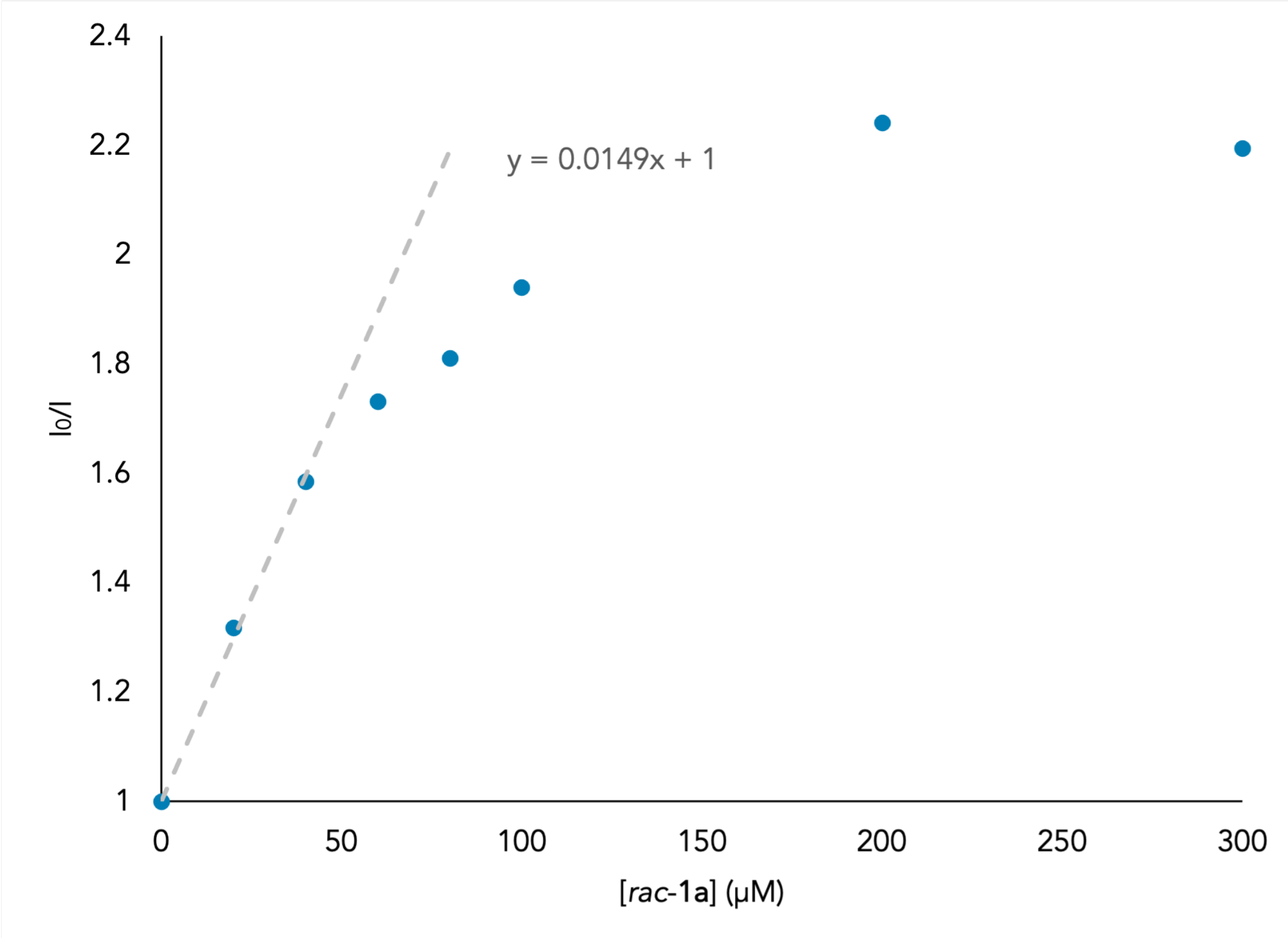

Figure S16.1. Stern-Volmer plot of **Ir-CPB** quenching with varying concentrations of *rac*-**1a**. The experiment demonstrates quenching but deviates from linearity.

$$\frac{I_0}{I} = 1 + \frac{k_q}{\tau}[Q]$$

The downward curvature of the plot indicates that besides dynamic quenching, there are other factors affecting luminescence. The K_{SV} of 0.0149 μM^{-1} or 14900 M^{-1} corresponds to a quenching rate $k_q = 1.09 \times 10^{10}$ M^{-1} s^{-1}, which is greater than diffusion-limited quenching. This led us to believe that static quenching was occurring, in which the photocatalyst and substrate could be forming a ground-state complex.

NMR Titrations of Ion-Paired Photocatalyst and 1a

$$\delta = \frac{[Ir^{+}, 1a] x\, \delta_{final} + ([Ir^{+}]_0 - [Ir^{+}, 1a]) x\, \delta_0}{[Ir^{+}]_0}$$

$$\Rightarrow [Ir^{+}, 1a] = \frac{[Ir^{+}]_0\, x\, (\delta - \delta_0)}{(\delta_{final} - \delta_0)}$$

$$K_{eq} = \frac{[Ir^{+}, 1a]}{([Ir^{+}]_0 - [Ir^{+}, 1a])\, x\, ([1a]_0 - [Ir^{+}, 1a])}$$

The equilibrium binding constant of **Ir-CPB** and **1a** were calculated by ^{1}H NMR from the downfield shift of the set of phenylpyridine protons (δ) relative to the chemical shift of the fully saturated complex (δ_{final}, 6.89 ppm) and the chemical shift without any **1a** (δ_0, 6.80 ppm). $[Ir^{+}]_0$ is the concentration of **Ir-CPB** added to solution, [**1a**] is the concentration of nitrile **1a** added to solution, and [Ir^{+}, **1a**] is the calculated concentration of Ir-nitrile formed *in situ*. The concentration of **Ir-CPB** in C_6D_6 was 3.0 mM and concentrations of **1a** were 0 mM, 30.0 mM, 60.0 mM, 90.0 mM, 120.0 mM, 150.0 mM. The equilibrium constant was calculated as the average of three different base concentrations using the equation for K_{eq} shown above, and averaged to arrive at the average equilibrium binding constant of 39.4.

Table S16.1 Catalyst **Ir-CPB** and **1a** association.

[**1a**] (mM)	ppm	[Ir^{+}, **1a**]	K_{eq}
0	6.7986	0	
30	6.8205	0.744	11.3
60	6.8418	1.468	16.4
90	6.8770	2.663	90.7
120	6.8863	2.980	1250
150	6.8869	3.000	—
		avg K_{eq} (of 3 expts)	39.4

K_{eq} was taken as the average of 3 experiments, in which [**1a**] is 30, 60, and 90 mM.

17. X-Ray Crystallography Data

(*S*)-**1t** – CCDC: 2566090

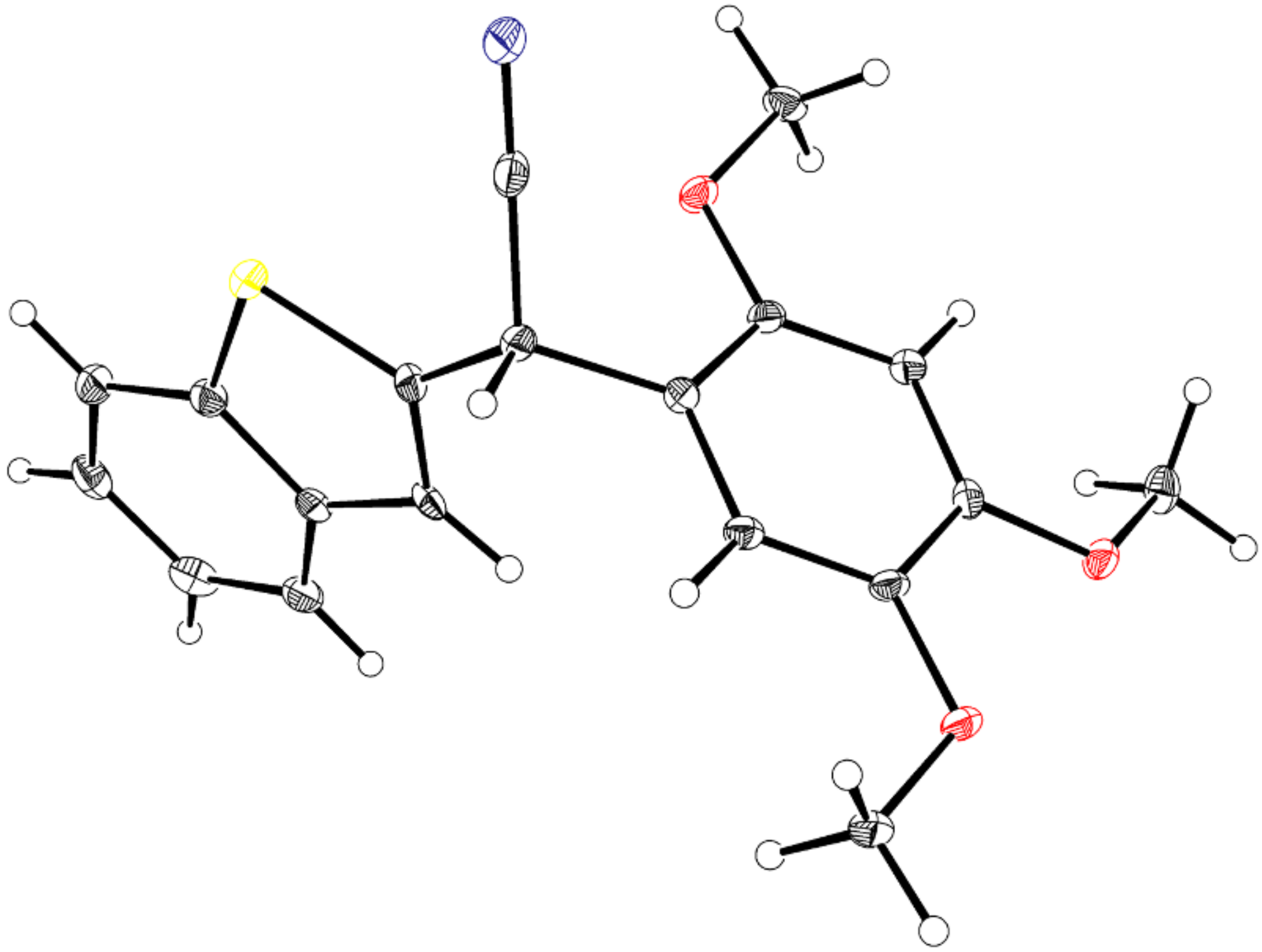

Figure S17.1. X-Ray Crystal Structure of (*S*)-**1t** at 30% probability ellipsoids. Equivalent molecule omitted for clarity. Clear, colorless needle-like crystals were prepared by slow evaporation from toluene.

Crystal data	
Chemical formula	$2(C_{19}H_{17}NO_3S)$
M_r	678.79
Crystal system, space group	Triclinic, *P*1
Temperature (K)	100
a, b, c (Å)	5.15042 (13), 10.8959 (4), 15.4474 (5)
α, β, γ (°)	107.395 (3), 93.078 (2), 93.046 (3)

V (Å^3)	823.86 (5)
Z	1
Radiation type	Cu *K*a
μ (mm^{-1})	1.89
Crystal size (mm)	0.48 × 0.21 × 0.17
Data collection	
Diffractometer	XtaLAB Synergy, Dualflex, HyPix-Arc 150
Absorption correction	Multi-scan *CrysAlis PRO* 1.171.44.128a (Rigaku Oxford Diffraction, 2025) Empirical absorption correction using spherical harmonics, implemented in SCALE3 ABSPACK scaling algorithm.
T_{min}, T_{max}	0.670, 1.000
No. of measured, independent and observed [$I > 2s(I)$] reflections	16154, 5186, 4837
R_{int}	0.048
(sin $\theta/\lambda)_{max}$ (Å^{-1})	0.628
Refinement	
$R[F^2 > 2s(F^2)]$, $wR(F^2)$, S	0.045, 0.120, 1.04
No. of reflections	5186
No. of parameters	439
No. of restraints	3
H-atom treatment	H-atom parameters constrained
$\Delta\rho_{max}$, $\Delta\rho_{min}$ (e Å^{-3})	0.23, -0.41

Absolute structure	Flack x determined using 1650 quotients [(I+)-(I-)]/[(I+)+(I-)] (Parsons, Flack and Wagner, Acta Cryst. B69 (2013) 249-259).
Absolute structure parameter	0.000 (16)

Computing details

Data collection: *CrysAlis PRO* 1.171.44.128a (Rigaku OD, 2025); cell refinement: *CrysAlis PRO* 1.171.44.128a (Rigaku OD, 2025); data reduction: *CrysAlis PRO* 1.171.44.128a (Rigaku OD, 2025); program(s) used to solve structure: SHELXT 2018/2 (Sheldrick, 2018); program(s) used to refine structure: *SHELXL* 2019/3 (Sheldrick, 2015); molecular graphics: Olex2 1.5 (Dolomanov *et al.*, 2009); software used to prepare material for publication: Olex2 1.5 (Dolomanov *et al.*, 2009).

(R)-**6** CCDC: 2566091

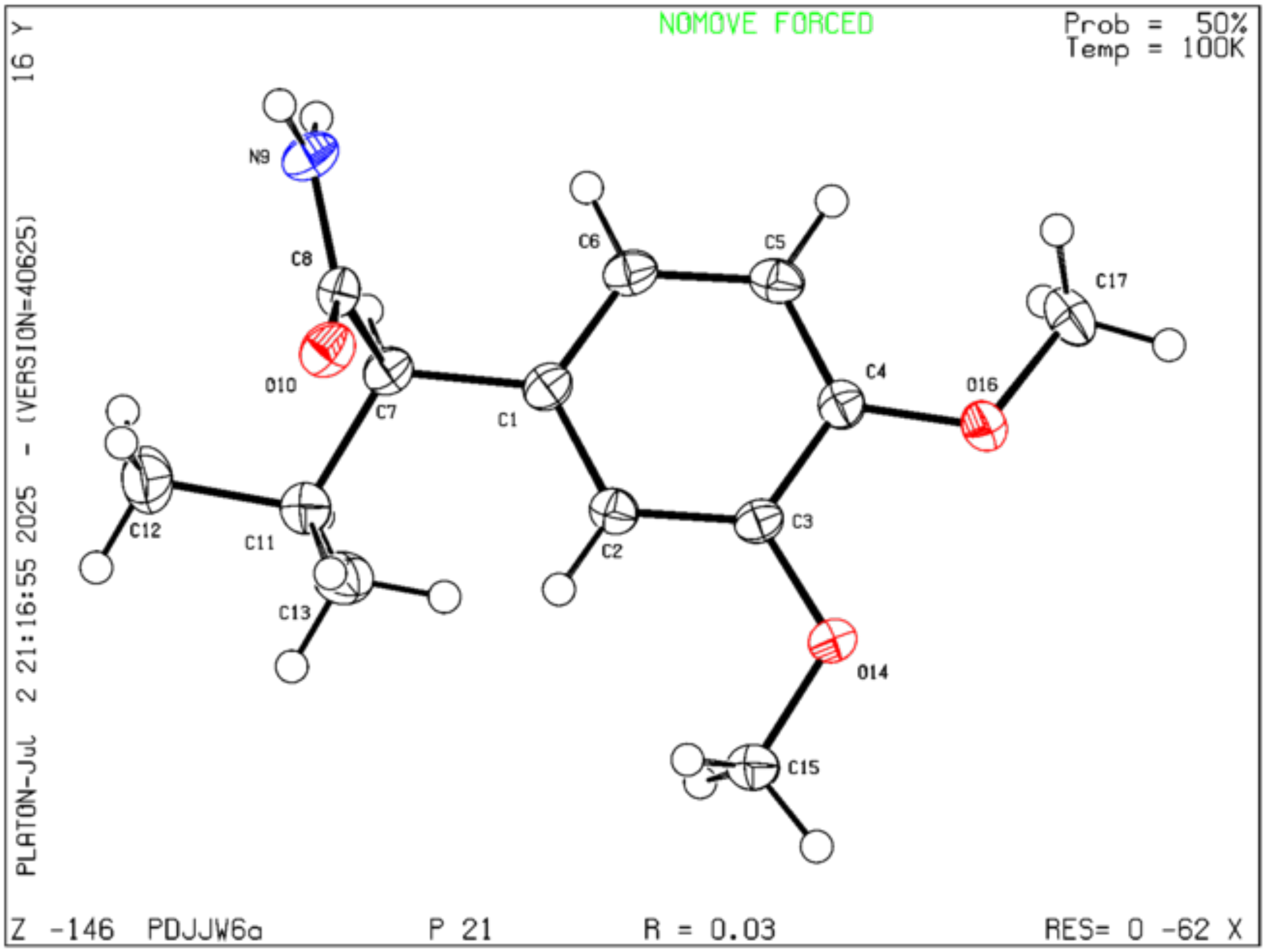

Figure S17.2. X-Ray Crystal Structure of (R)-**6** at 50% probability ellipsoids. Colorless prism-like crystals were prepared by slow evaporation from ethanol from a derivative of the *minor* enantiomer of the deracemization reaction with **1a**.

A colourless prism-like crystal of $C_{13}H_{19}NO_3$ with approximate dimensions of 0.320 mm x 0.160 mm x 0.100 mm was used for the X-ray crystallographic analysis. The X-ray intensity data were measured on a Rigaku Synergy-S system with a HyPix-150 detector equipped with a PhotonJet microfocus source (CuKα, λ = 1.54184 Å).

Crystal data	
Chemical formula	$C_{13}H_{19}NO_3$
M_r	237.29
Crystal system, space group	Monoclinic, *P21*
Temperature (K)	100

a, b, g (Å)	9.16770(8), 4.85304(5), 14.42457(11)
α, β, γ (°)	90, 90.1533(7), 90
V (Å^3)	641.765(9)
Z	2
Radiation type	Cu *K*a
μ (mm^{-1})	0.706 mm^{-1}
Crystal size (mm)	0.10 x 0.16 x 0.32
Data collection	
Diffractometer	XtaLAB Synergy, Dualflex, HyPix-Arc 150
Absorption correction	Gaussian
T_{min}, T_{max}	0.532, 1.000
No. of measured, independent and observed [I > 2s(I)] reflections	46621, 2333, 2312
R_{int}	0.0393
(sin θ/λ)$_{max}$ (Å^{-1})	0.793
Refinement	
R[F^2 > 2s(F^2)], wR(F^2), S	0.031, 0.0816, 1.088
No. of reflections	2333
No. of parameters	164
No. of restraints	2
$\Delta\rho_{max}$, $\Delta\rho_{min}$ (e Å^{-3})	+0.11, -0.17
Absolute structure	Flack x determined using 944 quotients [(I+)-(I-)]/[(I+)+(I-)]

	(Parsons, Flack and Wagner, Acta Cryst. B69 (2013) 249-259).
Absolute structure parameter	-0.16(9)

Computing details

Data collection: *CrysAlisPro 1.171.43.143a* (Rigaku OD, 2024); cell refinement: *CrysAlisPro 1.171.43.143a* (Rigaku OD, 2024); data reduction: *CrysAlisPro 1.171.43.143a* (Rigaku OD, 2024); program(s) used to solve structure: SHELXT 2018/2 (Sheldrick, 2018); program(s) used to refine structure: SHELXT 2018/2 (Sheldrick, 2018); molecular graphics: Platon; software used to prepare material for publication: Platon

18. Computational Details

18.1 Determination of Arene Radical Cation pK_a Values

pK_a calculations were performed using density functional theory as implemented in Gaussian 16.[19] Each compound was optimized with the B3LYP functional[20] along with GD3BJ dispersion.[21] The def2-TZVP basis set[22] was used for all atoms. Thermal corrections from geometry optimization were added to the energies found in single point energy calculations to find values for Gibbs free energy. The conductor-like polarizable continuum model (C-PCM) implicit solvation model for DMSO was used to account for solvation effects in all calculations.[23] Gibbs free energy was obtained at 1.0 M and 298.15 K (25 ºC) in DMSO. All geometries are fully optimized with stationary points characterized as local minima (zero imaginary frequencies) by analytical frequency calculations at the same level of theory.

The p*K*a of the arene radical cation intermediate implicated in the catalytic mechanism was benchmarked against the experimental nitrile pK_a value of phenylacetonitrile (pK_a = 21.9 in DMSO)[24] using the isodesmic reaction below.

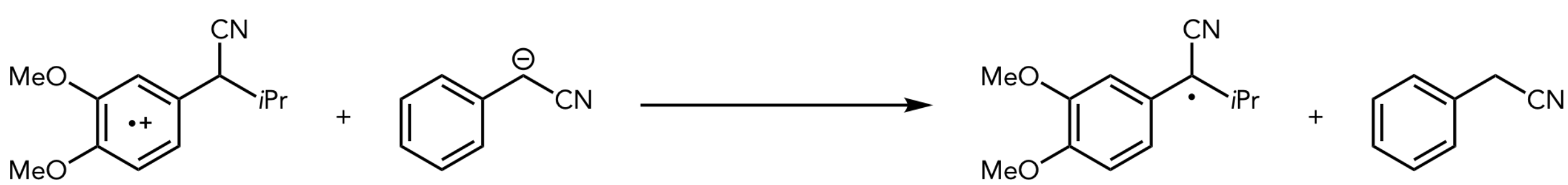

Figure S18.1 Isodesmic reaction employed for calculation of the pK_a of arene radical cation **1a**$^{\cdot+}$.

The pK_a values for the **1a**$^{\cdot+}$ in DMSO was determined using the following equation:

$$\Delta pK_a = [\Sigma G(\text{products})/1.36 - \Sigma G(\text{reactants})/1.36] \times 627.51\ (\text{kcal/mol})/\text{Hartree}$$

$$pK_a = \Delta pK_a + 21.9$$

The pK_a of **1a**$^{\cdot+}$ was calculated to be -11.5 in DMSO.

Phenylacetonitrile neutral

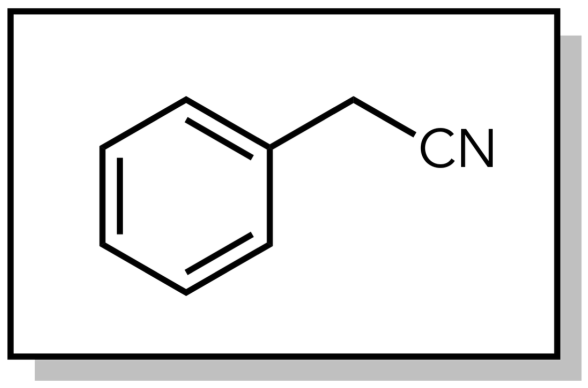

G(DMSO) = -363.884035

Charge = 0; Multiplicity = 1

C	2.3838360000	-0.8125390000	-0.0937170000
C	1.0284880000	-1.3394940000	-0.0593730000
H	-0.6945030000	1.7074370000	0.2152440000
C	2.6104200000	0.6216510000	-0.0454030000
C	-0.1238820000	-0.4355770000	0.0260660000
N	-3.7122030000	0.8069210000	-0.1659760000
C	-1.4877260000	-1.0609440000	0.1430970000
C	1.4786440000	1.5297370000	0.0384050000
H	3.2270510000	-1.4926820000	-0.1040710000
C	-2.6004120000	-0.1182630000	-0.0187770000
C	0.1215120000	1.0053700000	0.0997690000
H	0.8824520000	-2.4125270000	-0.0353760000
H	-1.5890710000	-1.8480180000	-0.6160960000
H	-1.5714060000	-1.5519980000	1.1190700000
H	1.6436480000	2.5994940000	0.0824920000
H	3.6219670000	1.0102000000	-0.0398330000

Phenylacetonitrile anion

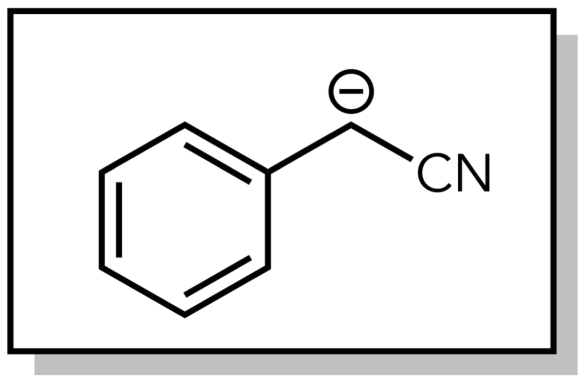

G(DMSO) = -363.400523

Charge = -1; Multiplicity = 1

C	-2.2451610000	-0.8385670000	0.0000320000
C	-0.9380820000	-1.2955360000	-0.0000770000
H	0.6571880000	1.7006570000	-0.0003050000
C	-2.5325230000	0.5280620000	0.0000950000
C	0.1676820000	-0.4057310000	0.0000550000
N	3.5562850000	0.6239660000	0.0000880000
C	1.5071470000	-0.9007920000	-0.0000850000
C	-1.4618940000	1.4235750000	-0.0000240000
H	-3.0558300000	-1.5589250000	-0.0000150000
C	2.6169150000	-0.0802210000	-0.0000160000
C	-0.1501910000	0.9775940000	-0.0000930000
H	-0.7460510000	-2.3630340000	-0.0002580000
H	-3.5549280000	0.8821400000	0.0002750000
H	1.6768830000	-1.9697730000	0.0004420000
H	-1.6546180000	2.4908690000	-0.0000770000

1a arene radical cation (**1a**$^{\bullet+}$)

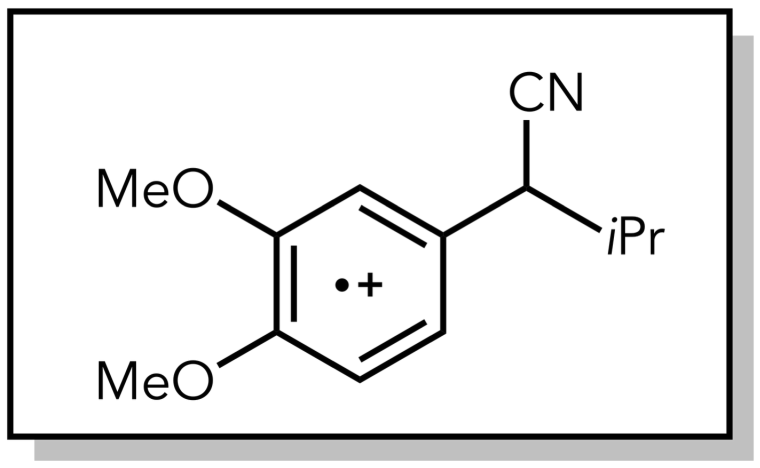

G(DMSO) = -710.689868

Charge = 1; Multiplicity = 2

C	1.2250520000	-2.0040380000	0.3221210000
C	-0.2057670000	-1.6676760000	-0.1405840000
H	0.1236770000	1.7763090000	0.3584760000
C	2.2200300000	-0.9356300000	-0.1574820000
C	-0.6628510000	-0.2580320000	0.3214610000
O	2.7929310000	1.4329320000	-0.0041600000
C	-2.0358210000	0.1902620000	-0.2676570000
C	1.8314440000	0.4357460000	0.4368840000
H	1.5192240000	-2.9877910000	-0.0511610000
O	3.5538200000	-1.3332730000	0.2374500000
C	0.4102170000	0.8038920000	-0.0414590000
H	-0.8748200000	-2.4370130000	0.2496520000
C	-3.1811550000	-0.8700150000	-0.1636270000
C	2.5452620000	2.8065490000	0.3759980000
C	-2.4678630000	1.5109710000	0.4178740000
C	-3.6529860000	-1.1037910000	1.2912280000
C	-4.3798640000	-0.4732290000	-1.0545650000
H	-2.8245280000	-1.8207730000	-0.5602000000
H	-4.8640890000	0.4367940000	-0.6941030000
H	-4.0531650000	-0.3052360000	-2.0836450000
H	-5.1261900000	-1.2715290000	-1.0676230000

H	-4.0967270000	-0.2006560000	1.7175830000
H	-4.4125100000	-1.8896930000	1.3205750000
H	-2.8224920000	-1.4139790000	1.9283710000
N	-2.9658780000	2.5113310000	-0.5402480000
H	3.4878350000	3.3585500000	0.3465170000
C	4.5744970000	-0.9957220000	-0.7309740000
H	1.8457250000	3.2502100000	-0.3381550000
H	4.2745930000	-1.3566570000	-1.7188860000
H	4.7140050000	0.0874710000	-0.7630610000
H	5.5144780000	-1.4700150000	-0.4389730000
H	2.1263160000	2.8486660000	1.3854900000
H	-1.8653560000	0.3830270000	-1.3307430000

1a benzylic radical (**1a•**)

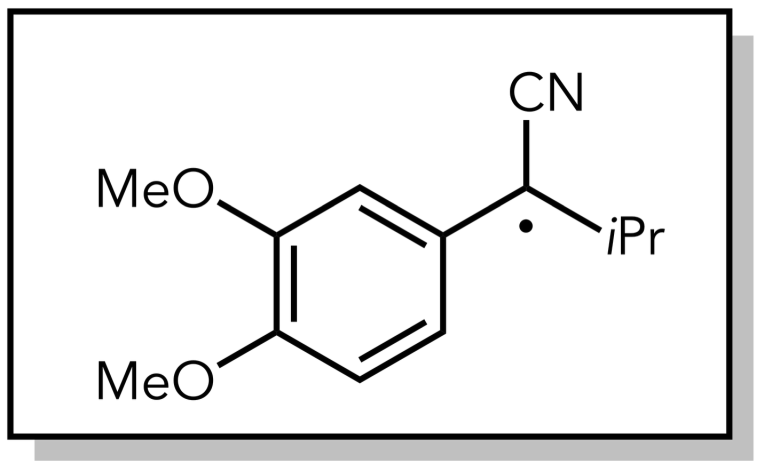

G(DMSO) = -710.278744

Charge = 0; Multiplicity = 2

C	1.1183450000	-1.9129220000	0.0924500000
C	-0.2191890000	-1.5766650000	-0.0021230000
H	0.1109740000	1.8062910000	0.0342690000
C	2.1067420000	-0.9387110000	0.1576540000
C	-0.6133200000	-0.2222570000	-0.0386590000
O	2.7446500000	1.3215230000	0.2646950000
C	-1.9885980000	0.1526760000	-0.1406920000
C	1.7338720000	0.4278350000	0.1468420000
H	1.4256220000	-2.9499670000	0.1161960000
O	3.4049490000	-1.3258850000	0.3023170000
C	0.3997770000	0.7667500000	0.0427260000
H	-0.9541470000	-2.3648230000	-0.0515910000
C	-3.1290460000	-0.8401080000	-0.1951180000
C	2.4229610000	2.7107190000	0.2601160000
C	-2.3221430000	1.5141550000	-0.1104230000
C	-3.5586010000	-1.2369840000	1.2275990000
C	-4.3215610000	-0.3256710000	-1.0043990000
H	-2.7552370000	-1.7335690000	-0.6969440000
H	-4.7733980000	0.5487800000	-0.5327620000
H	-4.0246470000	-0.0515840000	-2.0176110000
H	-5.0840400000	-1.1025350000	-1.0699310000

H	-3.9500820000	-0.3703180000	1.7640750000
H	-4.3431280000	-1.9944680000	1.1837750000
H	-2.7207560000	-1.6403030000	1.7966730000
H	1.7728900000	2.9646260000	1.1000470000
H	3.3695800000	3.2339130000	0.3595620000
C	4.2923240000	-1.0085800000	-0.7842760000
H	1.9407970000	2.9981630000	-0.6765950000
H	3.9469200000	-1.4941390000	-1.6996230000
H	4.3597890000	0.0672610000	-0.9354470000
H	5.2642290000	-1.4063650000	-0.5035410000
N	-2.6159340000	2.6375000000	-0.0881180000

18.2 Determination of Benzylic Radical Reduction Potentials

Reduction potential calculations were performed using density functional theory as implemented in Gaussian.[19] Each compound was optimized with the B3LYP functional[20] along with GD3BJ dispersion.[21] The 6-31+G(d,p) basis set[25] was used for all atoms. Thermal corrections from geometry optimization were added to the energies found in single point energy calculations to find values for Gibbs free energy. The conductor-like polarizable continuum model (C-PCM) implicit solvation model for acetonitrile was used to account for solvation effects in all calculations.[23] Gibbs free energy was obtained at 1.0 M and 298.15 K (25 ºC) in MeCN. All geometries are fully optimized with stationary points characterized as local minima (zero imaginary frequencies) by analytical frequency calculations at the same level of theory.

The reduction potential of the radical intermediate implicated in the catalytic mechanism was benchmarked against the experimental reduction potential value of 3-cyanotoluene (E^{o} = -1.51 V vs Fc^{+}/Fc in MeCN)[26] using the isodesmic reaction below.

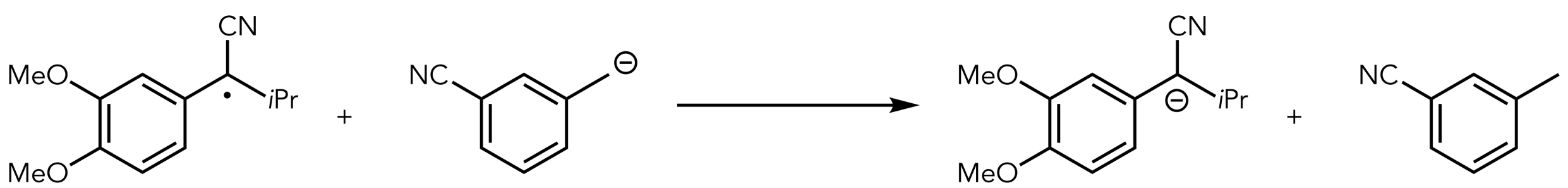

Figure S18.2 Isodesmic reaction employed for calculation of the E^{o} of benzylic radical **1a**$^{\cdot}$.

The E^{o} value for the radical derived from nitrile **1a** in MeCN was determined using the following equation:

$$\Delta E^{o} = [\Sigma G(\text{products})/23.06 - \Sigma G(\text{reactants})/23.06] \times 627.51 \text{ (kcal/mol)/Hartree}$$

$$E^{o} = \Delta E_{\text{red}} + 1.75$$

The E^{o} of **1a**$^{\cdot}$ was calculated to be –1.44 V vs Fc^{+}/Fc in MeCN.

1a benzylic radical (**1a**$^{\cdot}$)

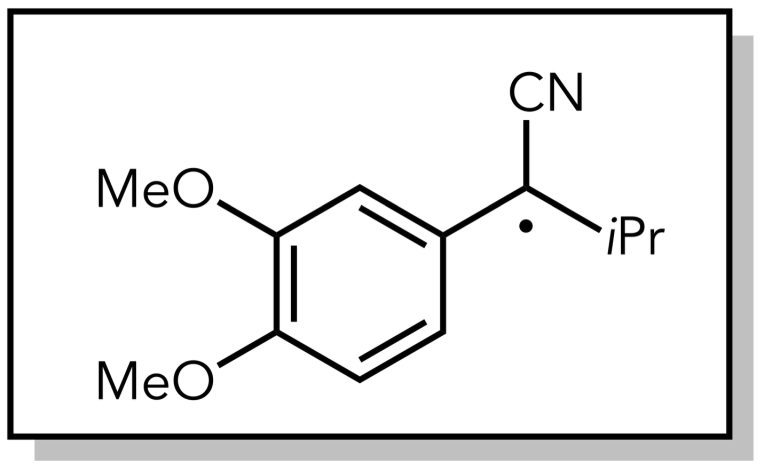

G(MeCN) = -710.278655

Charge = 0; Multiplicity = 2

C	-1.1182730000	-1.9130210000	-0.0923880000
C	0.2192590000	-1.5767470000	0.0019650000
H	-0.1109610000	1.8061560000	-0.0342780000
C	-2.1067010000	-0.9388530000	-0.1574180000
C	0.6133370000	-0.2223260000	0.0385140000
O	-2.7446660000	1.3213420000	-0.2647990000
C	1.9885970000	0.1527470000	0.1404510000
C	-1.7338790000	0.4277090000	-0.1468980000
H	-1.4255670000	-2.9500590000	-0.1161910000
O	-3.4048920000	-1.3260870000	-0.3018830000
C	-0.3998190000	0.7666250000	-0.0428470000
H	0.9542650000	-2.3648800000	0.0511840000
C	3.1290700000	-0.8400030000	0.1951980000
C	-2.4229980000	2.7105170000	-0.2605730000
C	2.3220120000	1.5142620000	0.1104820000
C	3.5586780000	-1.2373660000	-1.2273630000
C	4.3215900000	-0.3252040000	1.0042250000
H	2.7552580000	-1.7332740000	0.6973950000
H	4.7732500000	0.5491900000	0.5323200000
H	4.0247690000	-0.0508320000	2.0173900000
H	5.0841870000	-1.1019480000	1.0699200000

H	3.9502000000	-0.3708580000	-1.7640660000
H	4.3432020000	-1.9948430000	-1.1832740000
H	2.7208780000	-1.6408240000	-1.7964060000
N	2.6157860000	2.6376160000	0.0884390000
H	-3.3696260000	3.2337190000	-0.3599140000
C	-4.2923830000	-1.0080390000	0.7843790000
H	-1.9405640000	2.9981710000	0.6759410000
H	-3.9462790000	-1.4917510000	1.7004430000
H	-4.3610070000	0.0679730000	0.9338270000
H	-5.2638780000	-1.4073860000	0.5044320000
H	-1.7731110000	2.9642790000	-1.1007080000

1a keteniminate (**1a**$^-$)

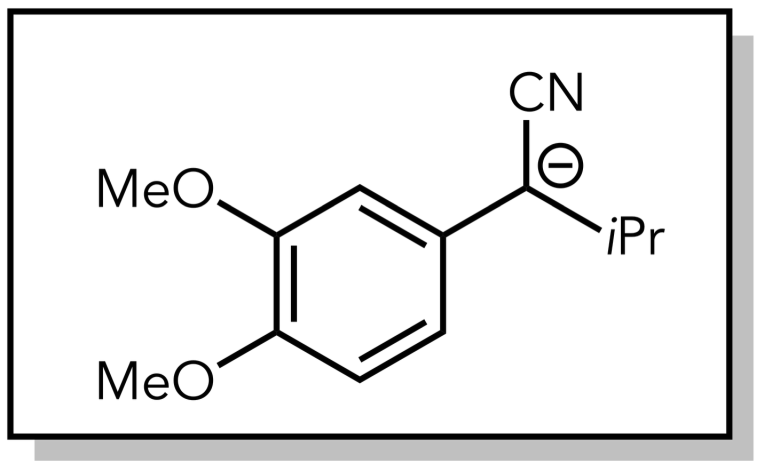

G(MeCN) = -710.408661

Charge = -1; Multiplicity = 1

C	1.1360710000	-1.8680850000	0.0964240000
C	-0.2099900000	-1.5352430000	0.0002930000
H	0.1198650000	1.8359870000	0.0541830000
C	2.1251160000	-0.9034320000	0.1863060000
C	-0.6316570000	-0.1825230000	-0.0304380000
O	2.7539730000	1.3611810000	0.2767970000
C	-2.0100430000	0.1947910000	-0.1557230000
C	1.7380510000	0.4504550000	0.1734460000
H	1.4367100000	-2.9096060000	0.1160240000
O	3.4546470000	-1.2668950000	0.3372980000
C	0.4013380000	0.7936940000	0.0669570000
H	-0.9359160000	-2.3333690000	-0.0516700000
C	-3.1195260000	-0.8418930000	-0.2195950000
C	2.4205790000	2.7412580000	0.2576930000
C	-2.3465110000	1.5346730000	-0.1284860000
C	-3.4640080000	-1.4157960000	1.1649520000
C	-4.3875420000	-0.3177930000	-0.8969090000
H	-2.7581330000	-1.6742880000	-0.8349170000
H	-4.8185840000	0.5133510000	-0.3337240000
H	-4.1779960000	0.0382530000	-1.9074500000
H	-5.1433520000	-1.1039130000	-0.9602400000

H	-3.9001470000	-0.6365380000	1.7959230000
H	-4.1851170000	-2.2361620000	1.0922130000
H	-2.5715630000	-1.7921530000	1.6670160000
N	-2.6239140000	2.6766330000	-0.1098800000
H	3.3615330000	3.2791300000	0.3456810000
C	4.2261270000	-1.1619010000	-0.8595300000
H	1.9281610000	3.0191510000	-0.6778730000
H	3.8225330000	-1.8178410000	-1.6370870000
H	4.2417620000	-0.1332450000	-1.2266310000
H	5.2402090000	-1.4745660000	-0.6130200000
H	1.7704470000	3.0058750000	1.0956520000

3-cyanotoluene radical

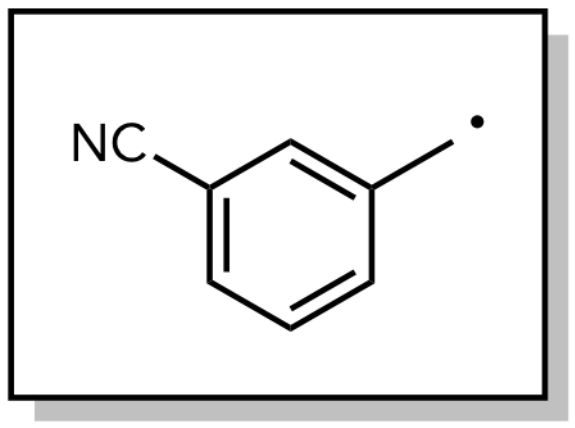

G(MeCN) = -710.02622

Charge = 0; Multiplicity = 2

C	0.7146690000	1.8642410000	0.1989360000
C	1.7608820000	0.8695900000	-0.2788180000
H	-0.1280130000	-1.9698560000	0.3384320000
C	-0.6737870000	1.4016840000	-0.2119590000
C	1.4798090000	-0.5164490000	0.2882480000
C	-2.3096160000	-0.4033840000	0.0591440000
H	2.3326090000	-2.5116460000	0.2081520000
C	-0.9520180000	0.0349170000	0.3957490000
H	0.9228070000	2.8641980000	-0.2348000000
C	2.5175220000	-1.5037110000	-0.2181690000
C	0.0776760000	-0.9708750000	-0.1014960000
H	2.7644330000	1.2167810000	0.0455400000
H	3.5344780000	-1.1786810000	0.0863280000
H	-1.4244000000	2.1415030000	0.1367360000
N	-3.3846770000	-0.7454830000	-0.1957430000

3-cyanotoluene anion

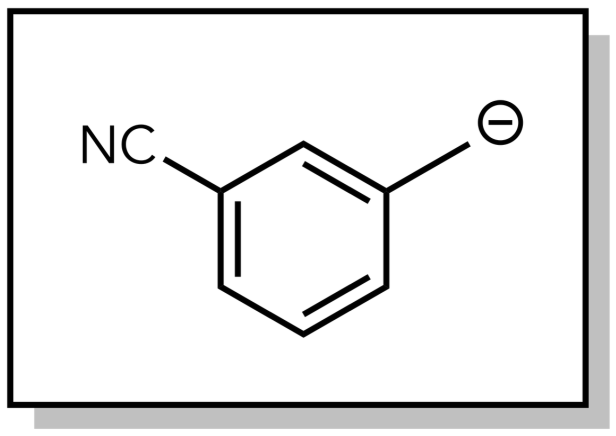

G(MeCN) = -363.257002

Charge = -1; Multiplicity = 1

C	0.7146690000	1.8642410000	0.1989360000
C	1.7608820000	0.8695900000	-0.2788180000
H	-0.1280130000	-1.9698560000	0.3384320000
C	-0.6737870000	1.4016840000	-0.2119590000
C	1.4798090000	-0.5164490000	0.2882480000
C	-2.3096160000	-0.4033840000	0.0591440000
H	2.3326090000	-2.5116460000	0.2081520000
C	-0.9520180000	0.0349170000	0.3957490000
H	0.9228070000	2.8641980000	-0.2348000000
C	2.5175220000	-1.5037110000	-0.2181690000
C	0.0776760000	-0.9708750000	-0.1014960000
H	2.7644330000	1.2167810000	0.0455400000
H	3.5344780000	-1.1786810000	0.0863280000
H	-1.4244000000	2.1415030000	0.1367360000
N	-3.3846770000	-0.7454830000	-0.1957430000

18.3 Determination of Reaction Coordinate for Ring Opening of 1e

Reaction coordinate calculations were performed using density functional theory as implemented in Gaussian 16.[19] Each compound was optimized with the UB3LYP functional[20] along with GD3BJ dispersion.[21] The 6-31++G(d,p) basis set[25] was used for all atoms. Thermal corrections from geometry optimization were added to the energies found in single point energy calculations to find values for Gibbs free energy. The conductor-like polarizable continuum model (C-PCM) implicit solvation model for toluene was used to account for solvation effects in all calculations.[23] Gibbs free energy was obtained at 1.0 M and 298.15 K (25 ºC) in toluene. All geometries are fully optimized with stationary points characterized as local minima (zero imaginary frequencies) by analytical frequency calculations at the same level of theory.

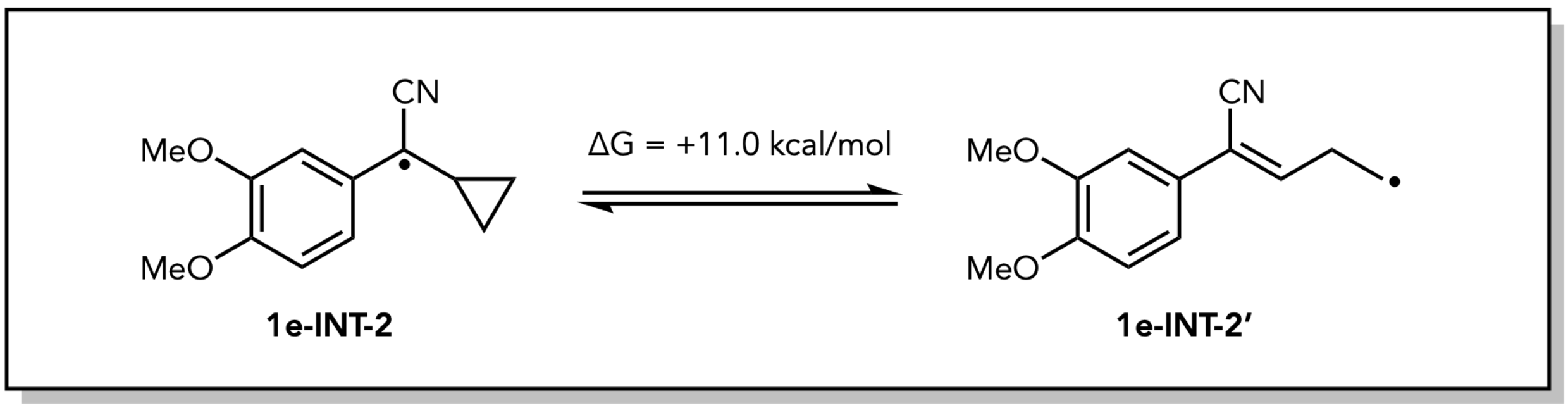

Figure S18.3 Ring opening free energy of **1e**.

1e-INT-2

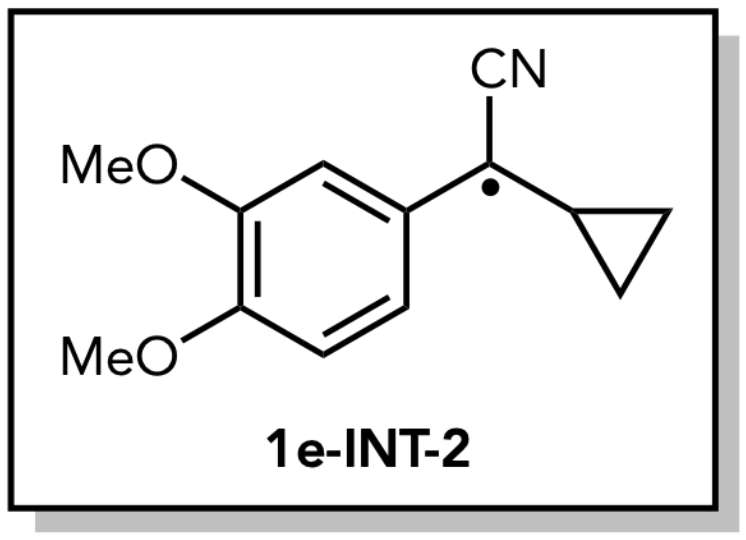

G(toluene) = -708.82198

Charge = 0; Multiplicity = 2

N	-2.65648	2.57917	0.15385
C	-2.36587	1.46832	0.04467
C	-2.02996	0.10679	-0.08088
C	-3.13647	-0.86649	-0.22342
C	-4.57458	-0.42454	-0.40386
C	-4.16497	-1.04916	0.89713
C	-0.64069	-0.26703	-0.10935
C	0.37308	0.72810	-0.05149
C	1.71973	0.39727	-0.10386
O	2.73055	1.30995	-0.13354
C	2.39268	2.69567	-0.06010
C	2.10535	-0.96910	-0.16390
O	3.40119	-1.37142	-0.31011
C	4.36960	-0.91699	0.65533
C	1.11217	-1.95004	-0.22322
C	-0.23685	-1.62039	-0.20470
H	-0.97158	-2.41390	-0.24922
H	-2.86402	-1.77616	-0.74604
H	-5.18064	-1.00574	-1.09133
H	-4.77457	0.64230	-0.41047
H	-4.48021	-2.06413	1.11848

H	-4.07256	-0.39822	1.76087
H	0.07621	1.76729	0.00035
H	3.33999	3.23175	-0.10970
H	1.88526	2.92674	0.88308
H	1.75793	2.99129	-0.90254
H	5.22455	-1.58600	0.54821
H	3.96118	-0.99836	1.66789
H	4.67178	0.11189	0.45567
H	1.42894	-2.98564	-0.28702

1e-INT-2'

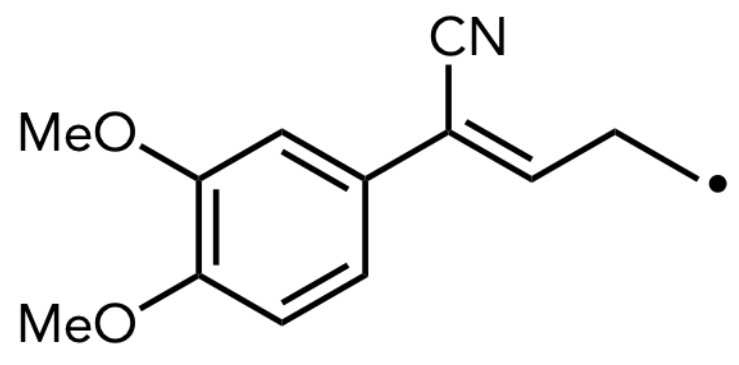

G(toluene) = -708.80428

Charge = 0; Multiplicity = 2

C	-4.11082	-2.12300	0.15415
C	-3.45053	-1.08223	1.00511
C	-3.26827	0.22114	0.28273
C	-2.10503	0.73188	-0.18451
C	-2.14972	2.03790	-0.78670
N	-2.15760	3.09806	-1.26894
C	-0.76225	0.09467	-0.12179
C	0.35396	0.87869	0.22194
C	1.62843	0.31566	0.29601
O	2.75029	0.99897	0.65476
C	2.62729	2.38966	0.95369
C	1.79984	-1.05595	-0.00402
O	3.02764	-1.66078	0.09817
C	3.99565	-1.26614	-0.88965
C	0.69412	-1.82333	-0.35540
C	-0.58313	-1.26100	-0.41772
H	-4.72098	-2.89706	0.60641
H	-3.92423	-2.16627	-0.91353
H	-1.42321	-1.87191	-0.72511
H	-2.47985	-1.44278	1.37940
H	-4.07030	-0.89814	1.89312

H	-4.17136	0.80670	0.12827
H	0.21013	1.92818	0.44564
H	3.63310	2.72698	1.20238
H	1.96359	2.55058	1.81058
H	2.25424	2.94777	0.08775
H	4.89300	-1.85010	-0.68132
H	4.21985	-0.19912	-0.81488
H	3.62636	-1.50321	-1.89426
H	0.85221	-2.87112	-0.58840

19. Unsuccessful Substrates

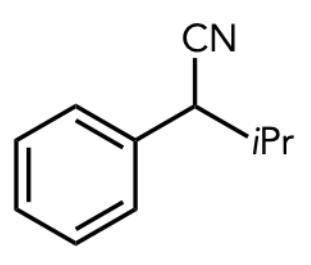
0% ee

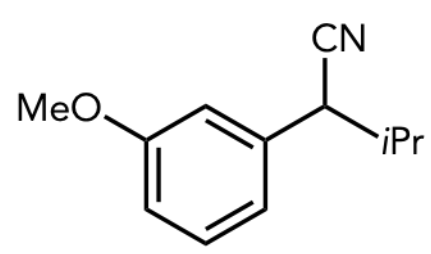
8% ee

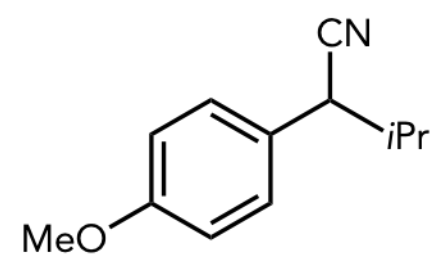
36% ee (8 h)

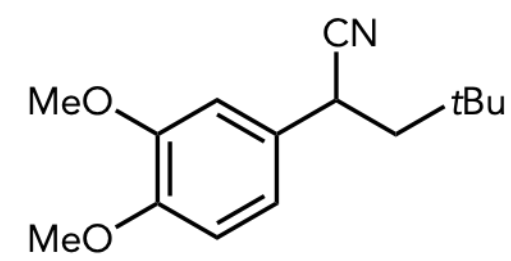
66% ee

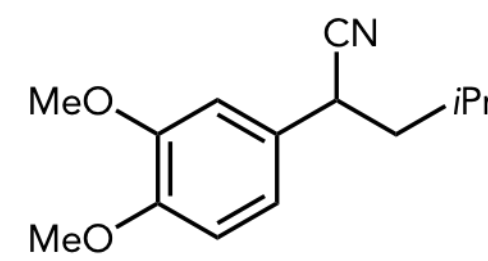
62% ee

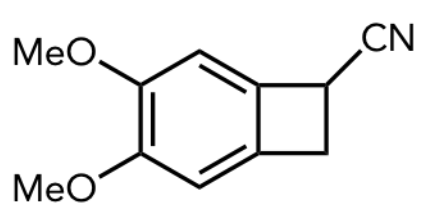
0% ee

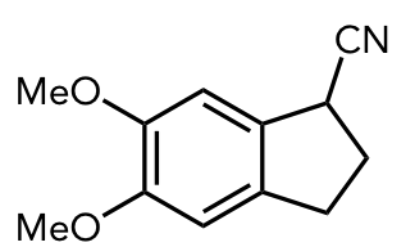
46% ee

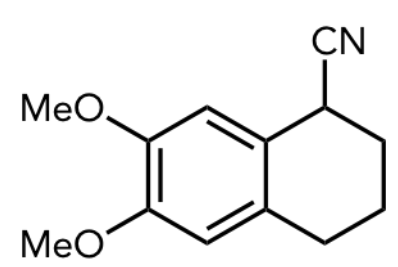
20% ee

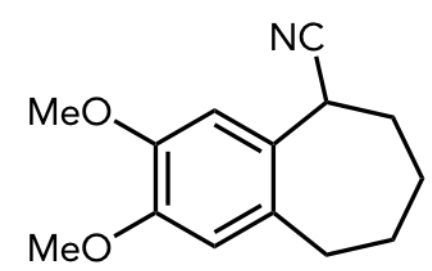
20% ee

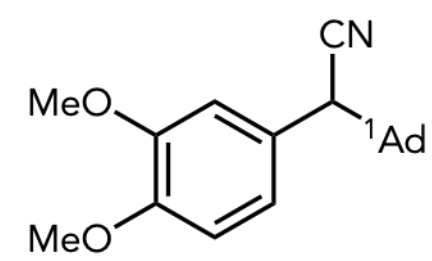
50% ee

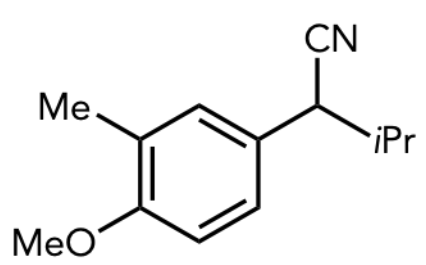
48% ee

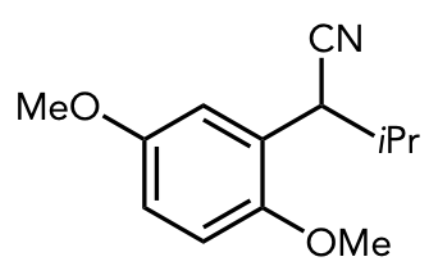
0% ee

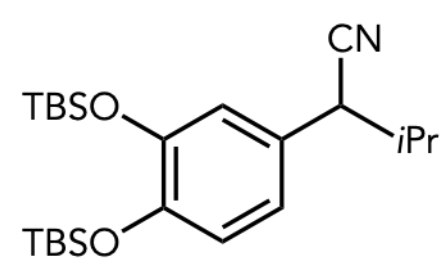
50% ee

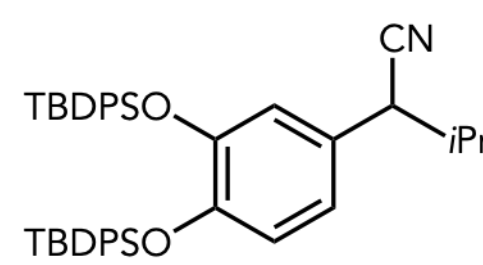
40% ee

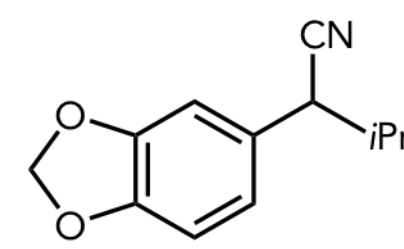
0% ee

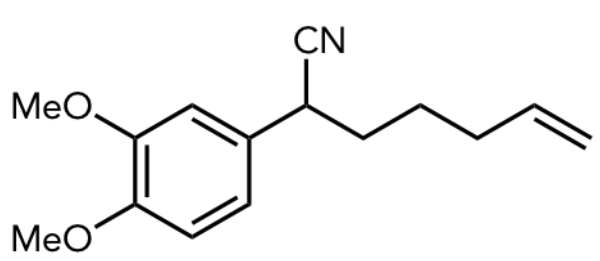
50% ee

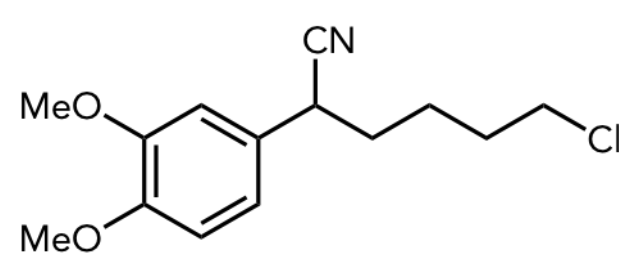
30% ee

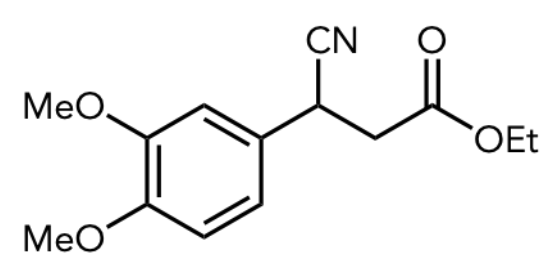
20% ee

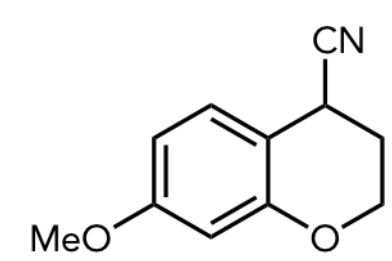
34% ee

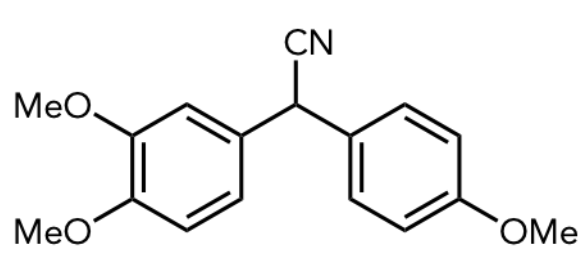
32% ee

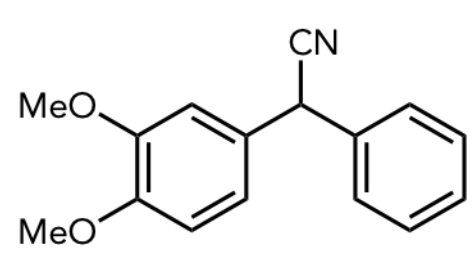
32% ee

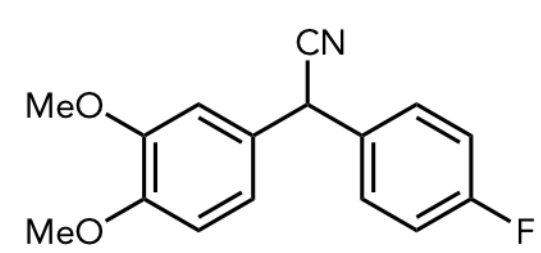
32% ee

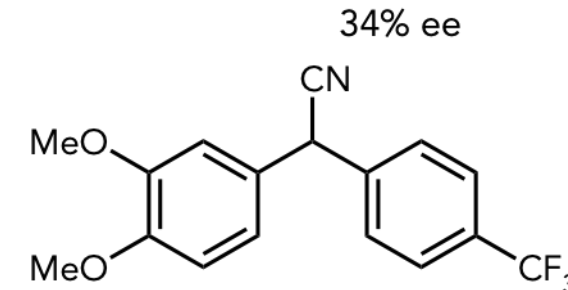
24% ee

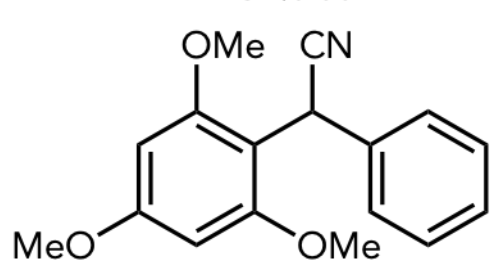
62% ee

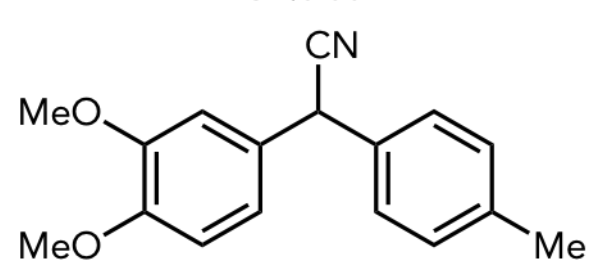
32% ee

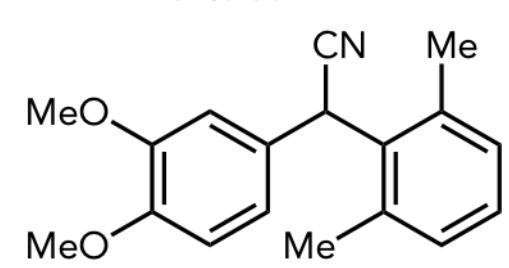
0% ee

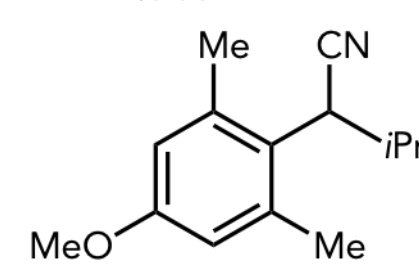
0% ee

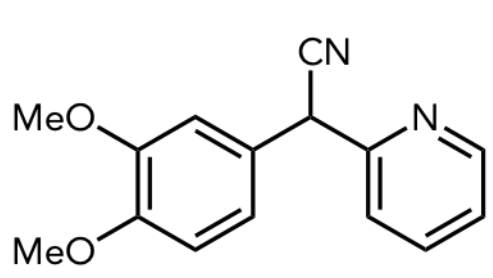
0% ee

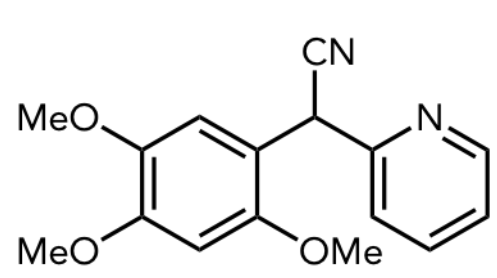
0% ee

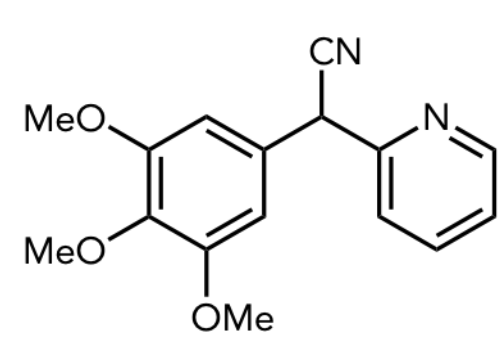
0% ee

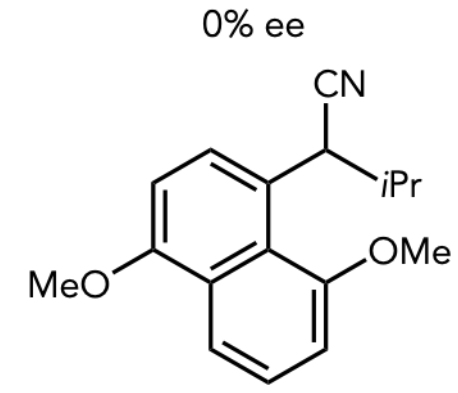
0% ee

20. NMR spectra of catalysts, substrates, and deracemized products

^{1}H NMR (500 MHz, $CDCl_3$), ^{13}C NMR (126 MHz, $CDCl_3$), ^{19}F NMR (470 MHz, $CDCl_3$) and ^{31}P NMR (203 MHz, $CDCl_3$) spectra for catalyst **Ir-CPB**

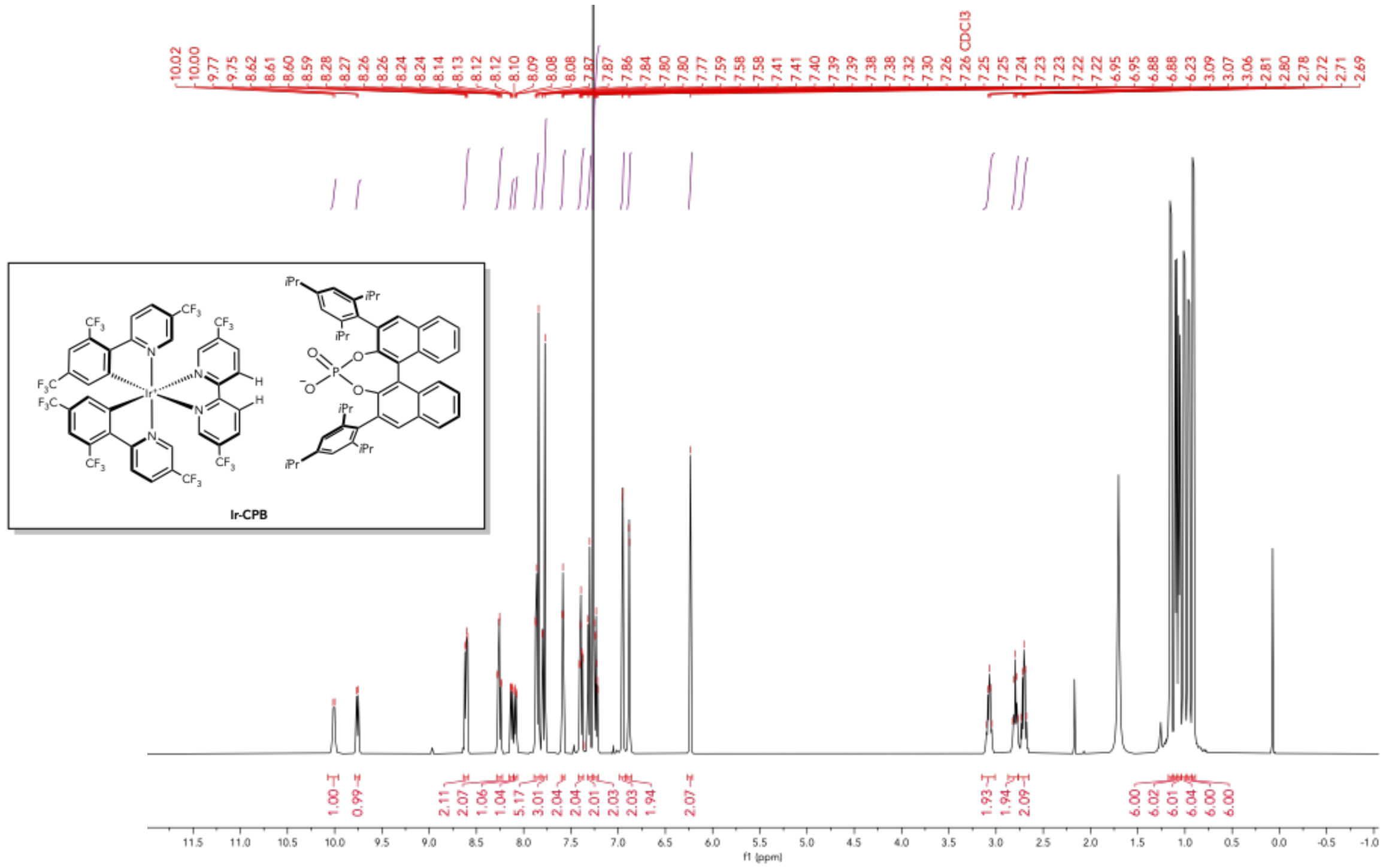

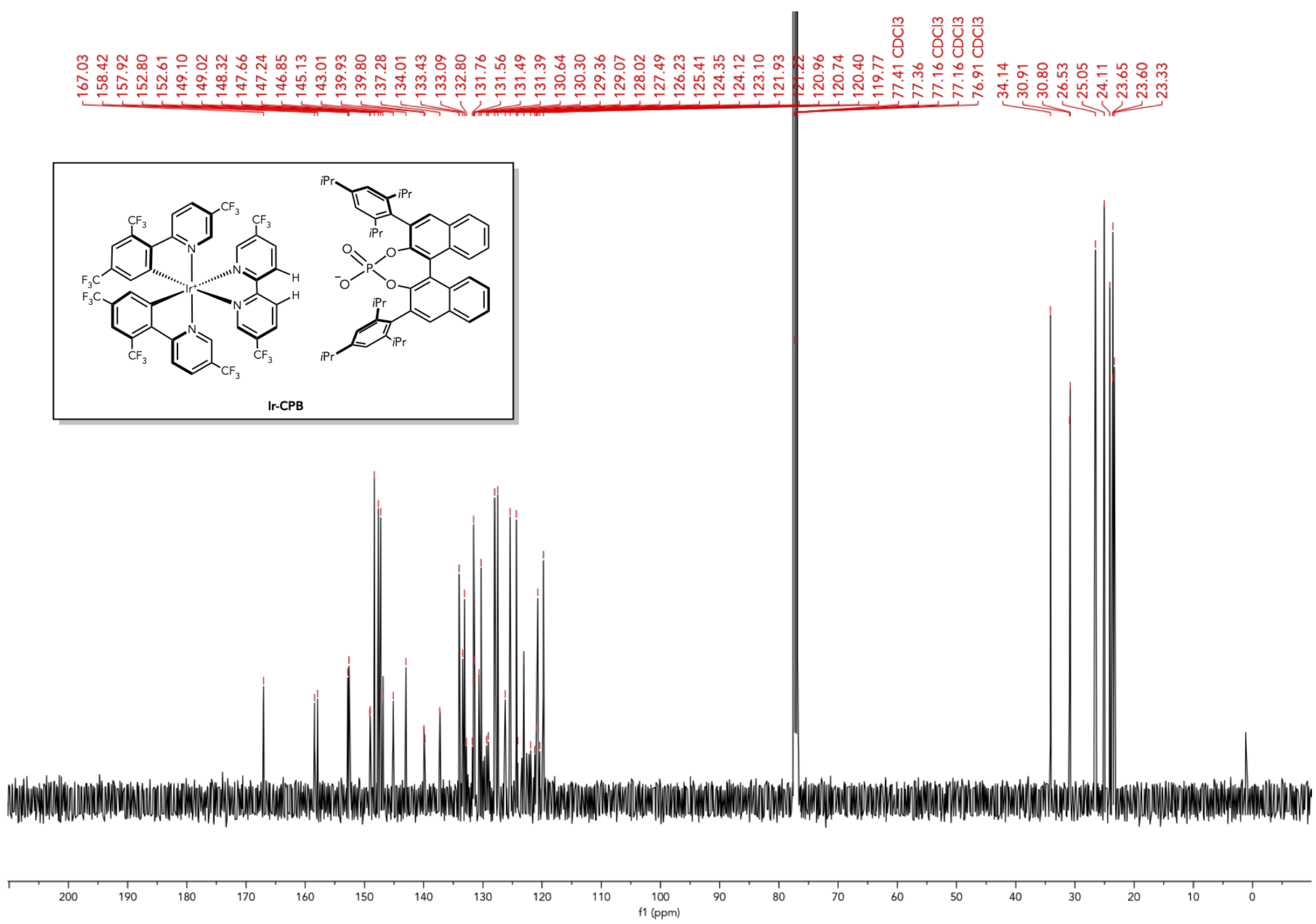

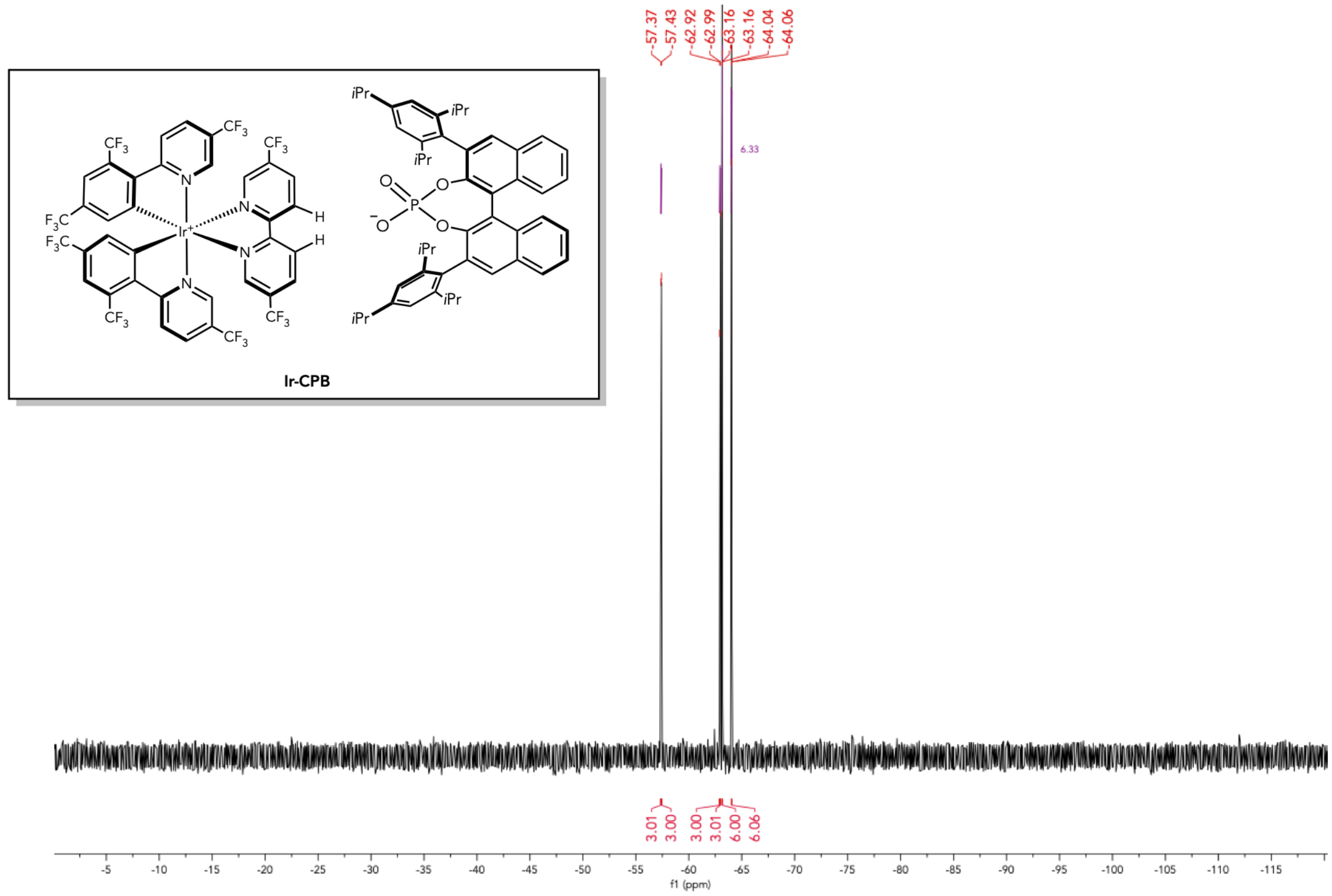

-57.37
-57.43
-62.92
-62.99
-63.16
-63.16
-64.04
-64.06
6.33
3.01
3.00
3.00
3.01
6.00
6.06
Ir-CPB
f1 (ppm)

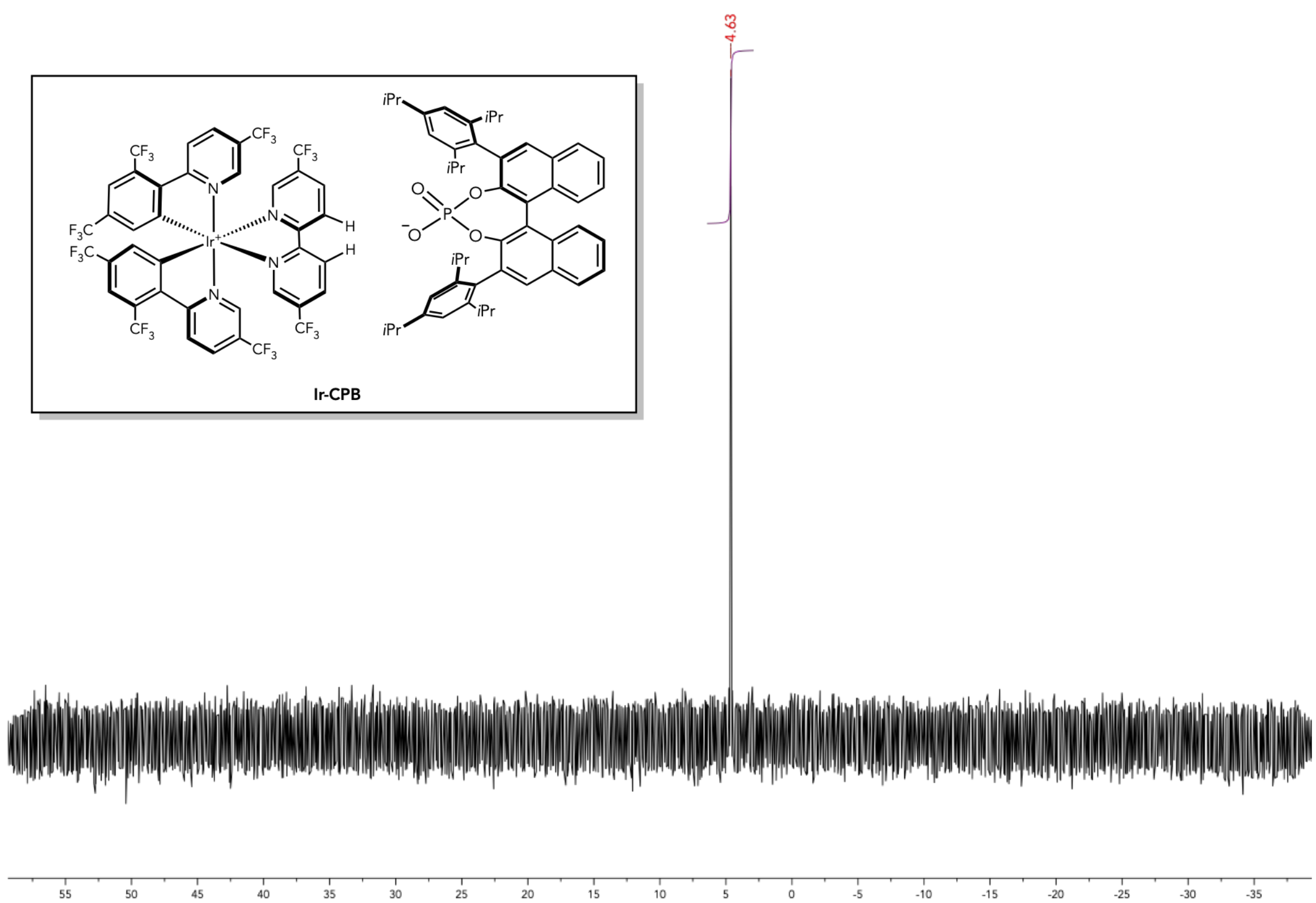

4.63
Ir-CPB
f1 (ppm)

^{1}H NMR (500 MHz, $CDCl_3$), ^{13}C NMR (126 MHz, $CDCl_3$), and ^{31}P NMR (162 MHz, $CDCl_3$) spectra for catalyst **CPB-2**

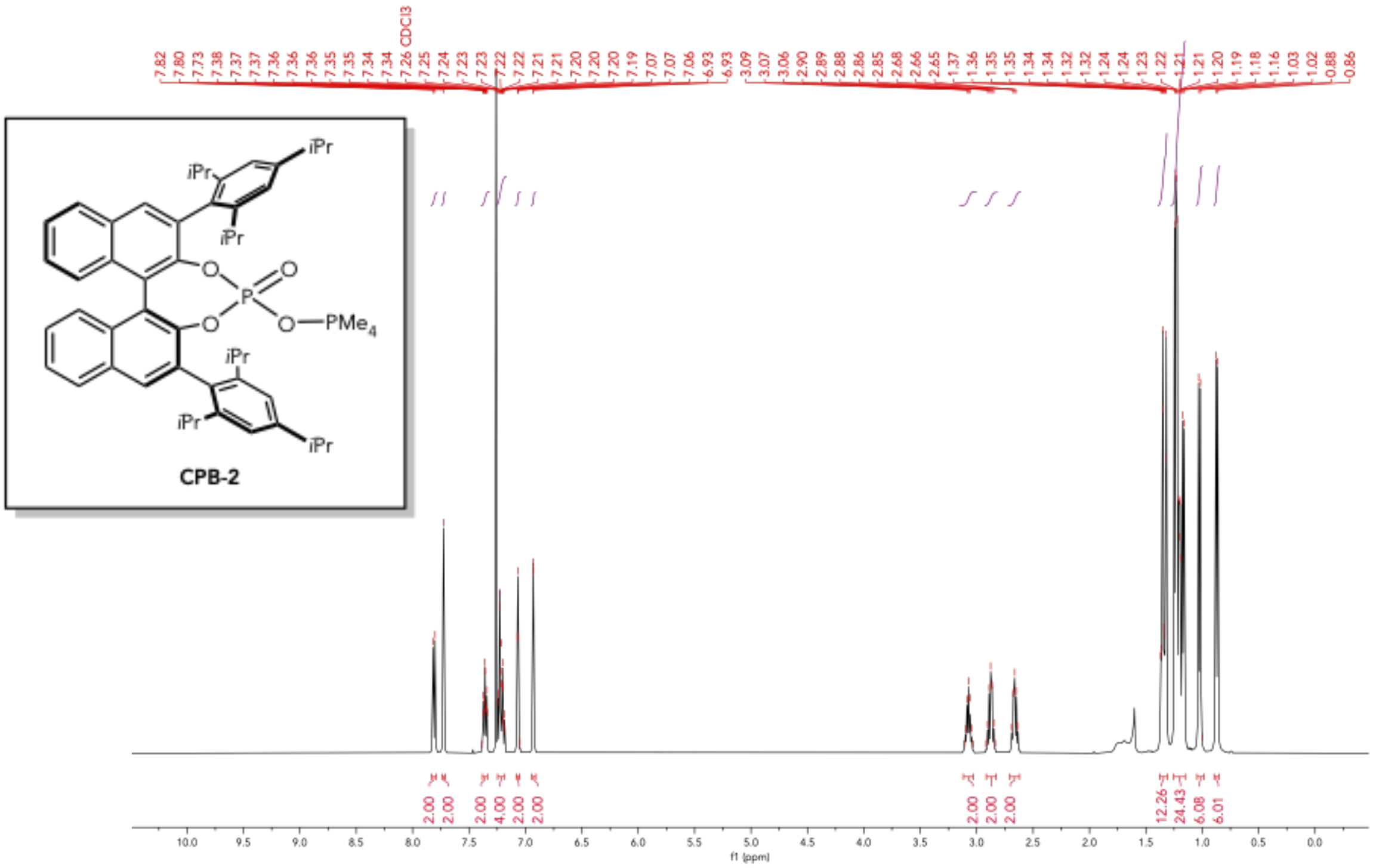

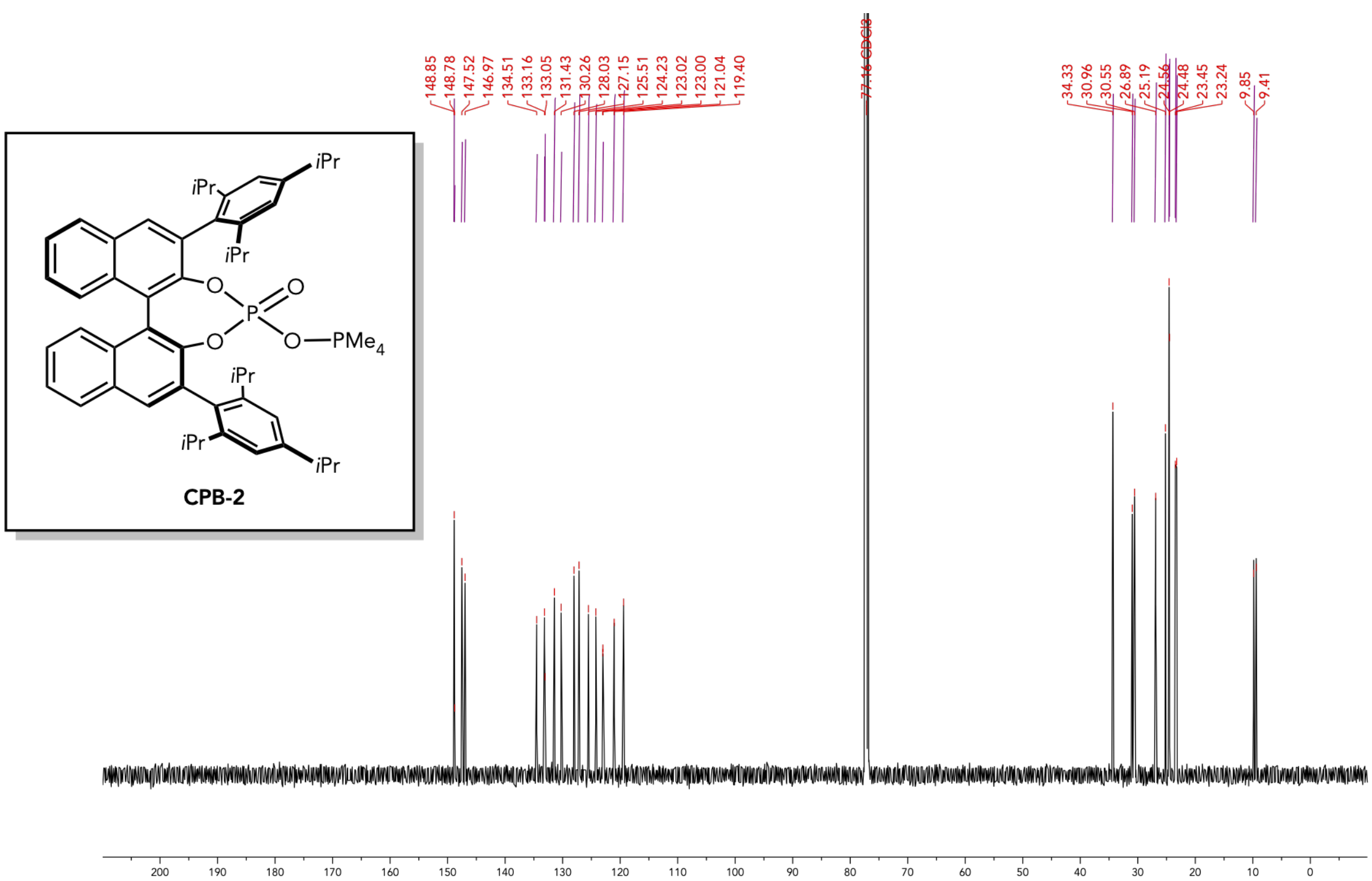

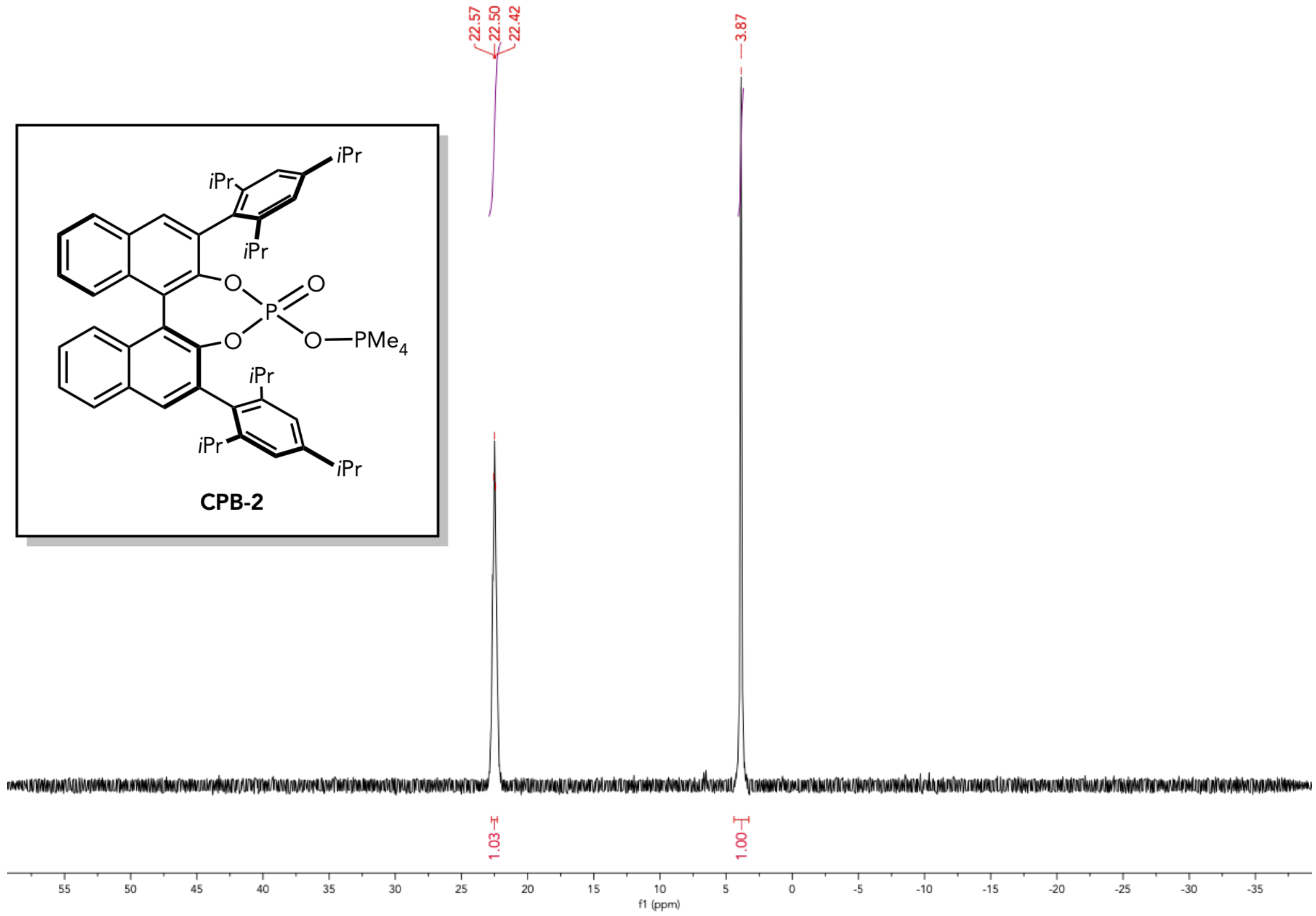
iPr
iPr
iPr
O
O
P
O
O
O
PMe4
iPr
iPr
iPr
CPB-2
22.57
22.50
22.42
3.87
1.03
1.00
55
50
45
40
35
30
25
20
15
10
5
0
-5
-10
-15
-20
-25
-30
-35
f1 (ppm)

^{1}H NMR (500 MHz, $CDCl_3$) and ^{13}C NMR (126 MHz, $CDCl_3$) spectra for compound *rac*-**1a**

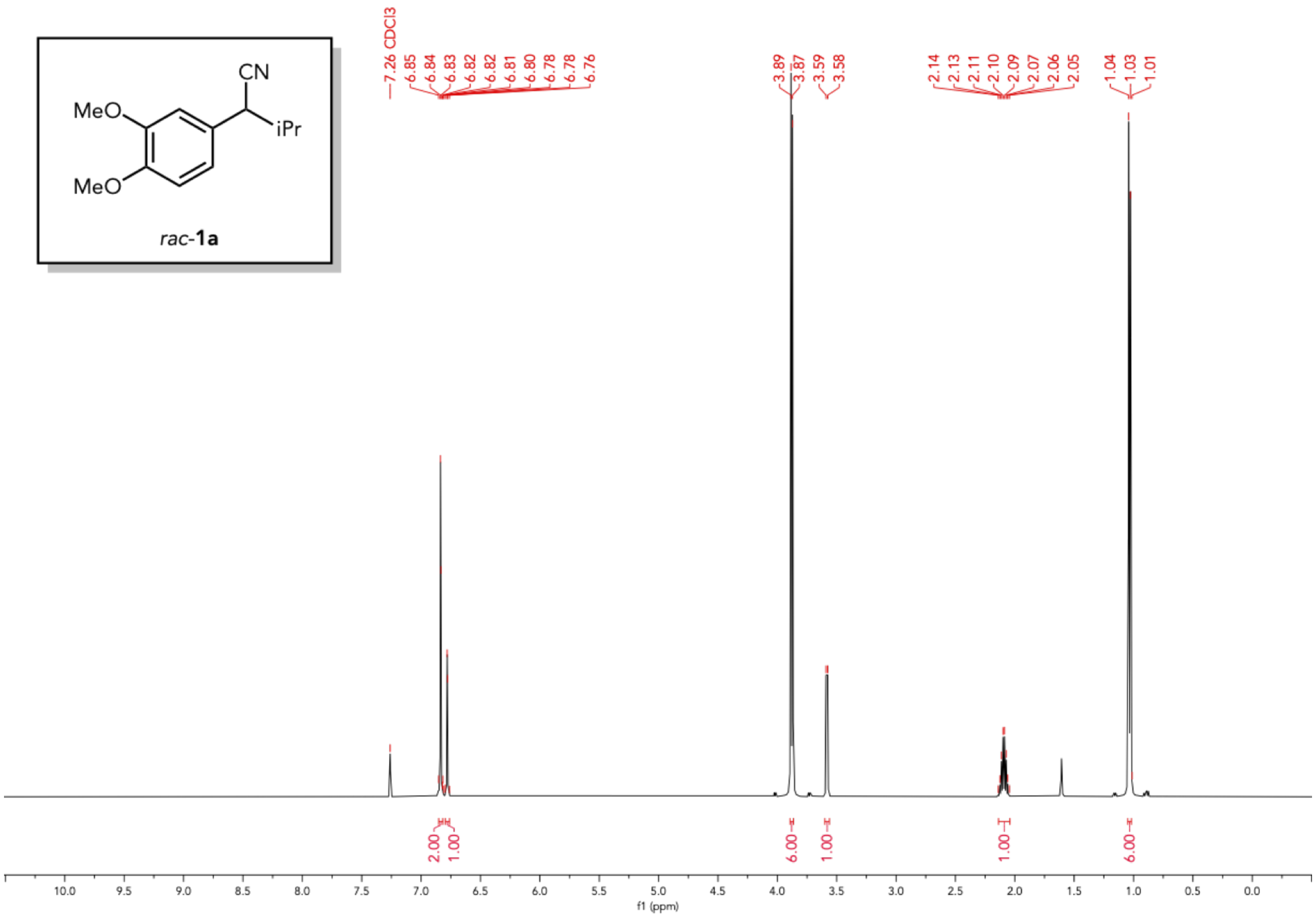

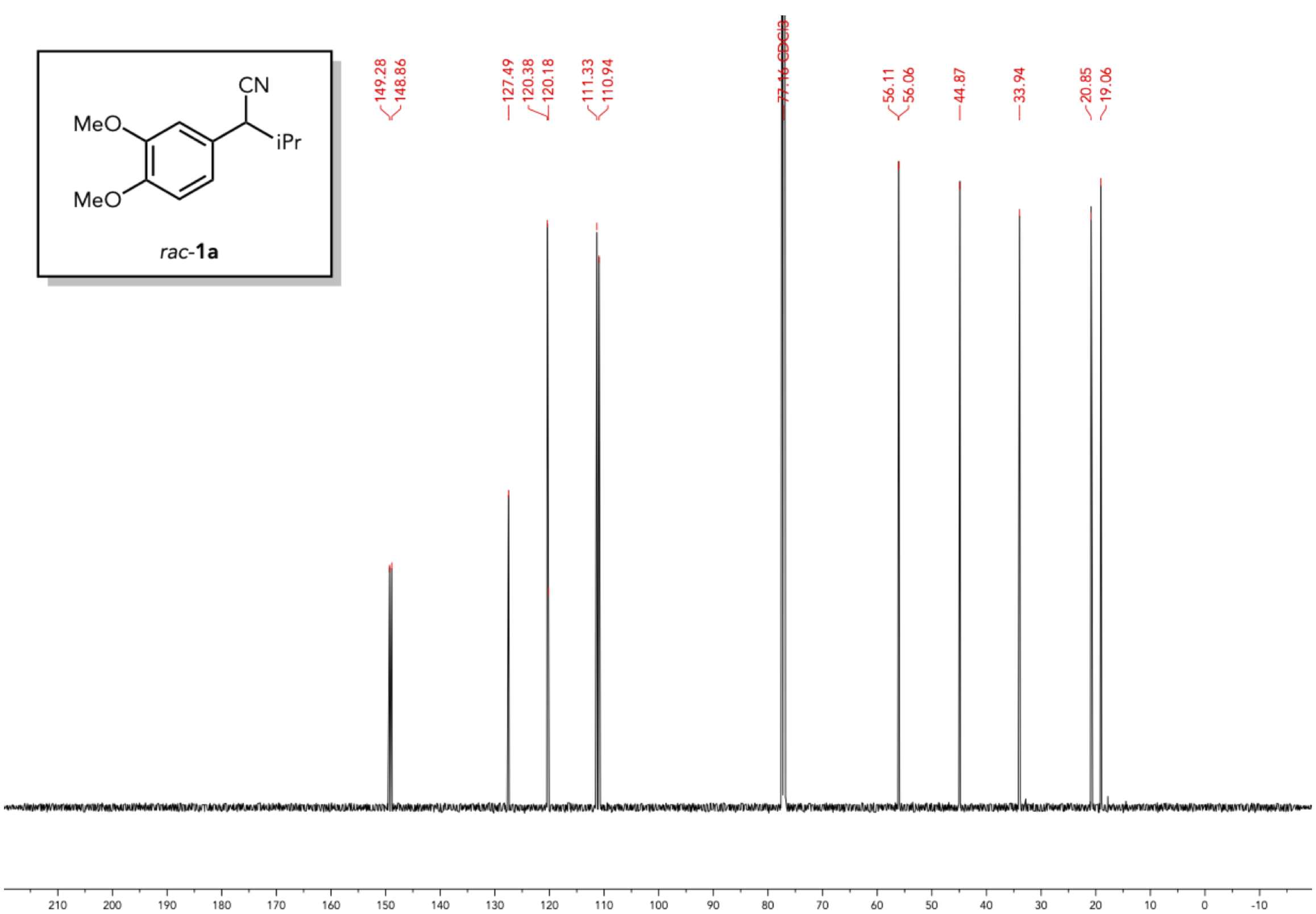

^{1}H NMR (500 MHz, $CDCl_3$) and ^{13}C NMR (126 MHz, $CDCl_3$) spectra for compound *rac*-**1b**

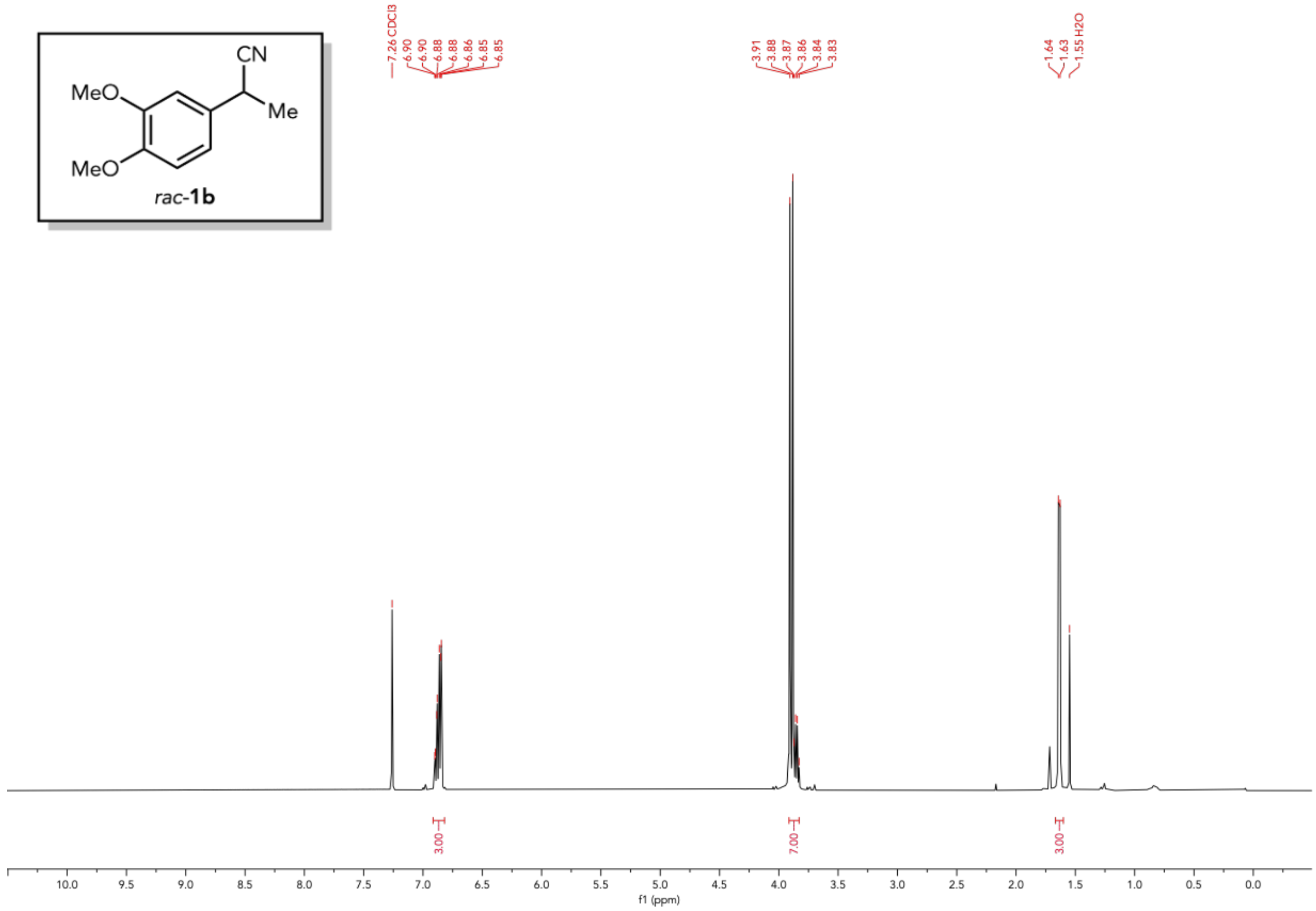

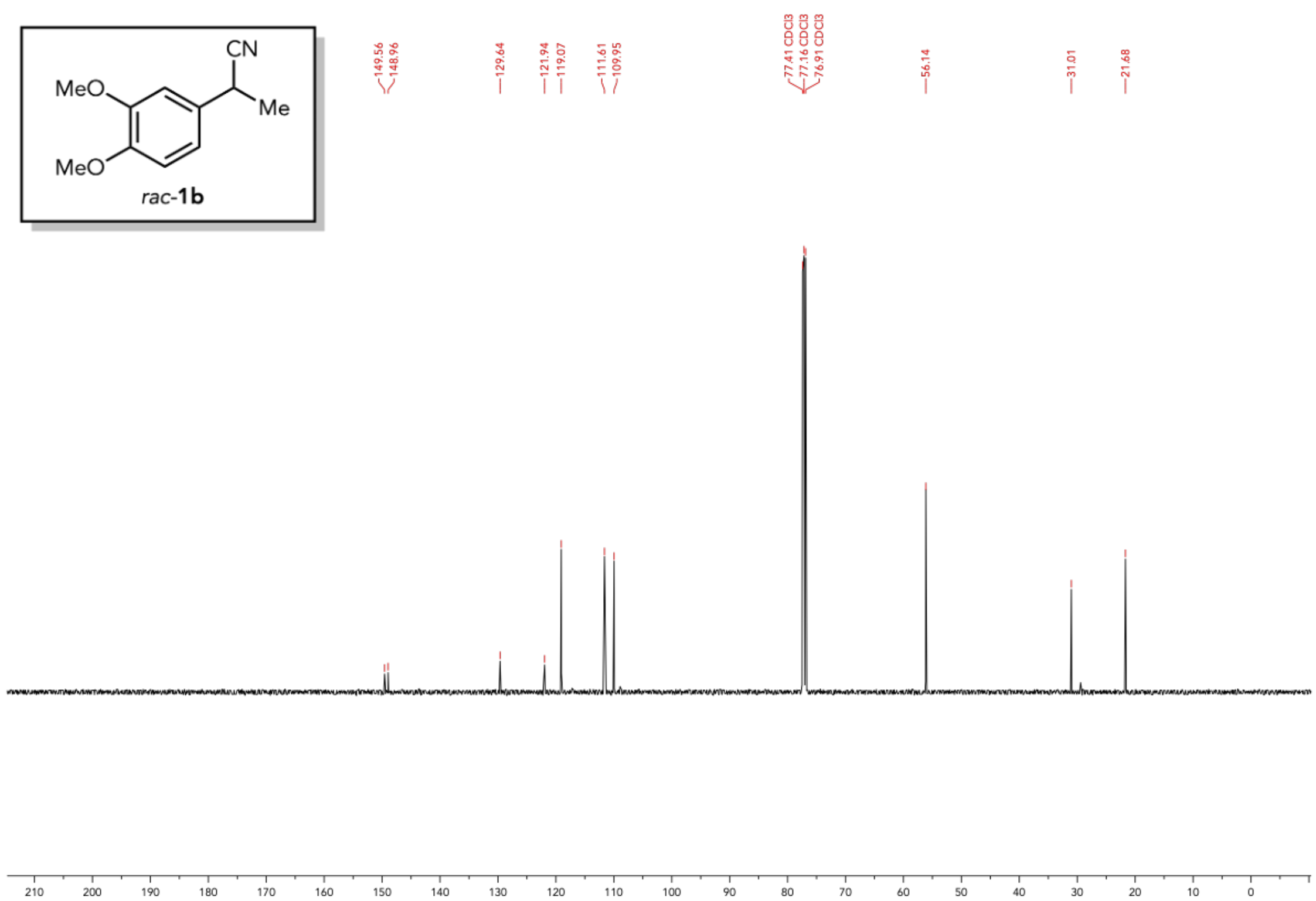

^{1}H NMR (500 MHz, $CDCl_3$) and ^{13}C NMR (126 MHz, $CDCl_3$) spectra for compound *rac*-**1c**

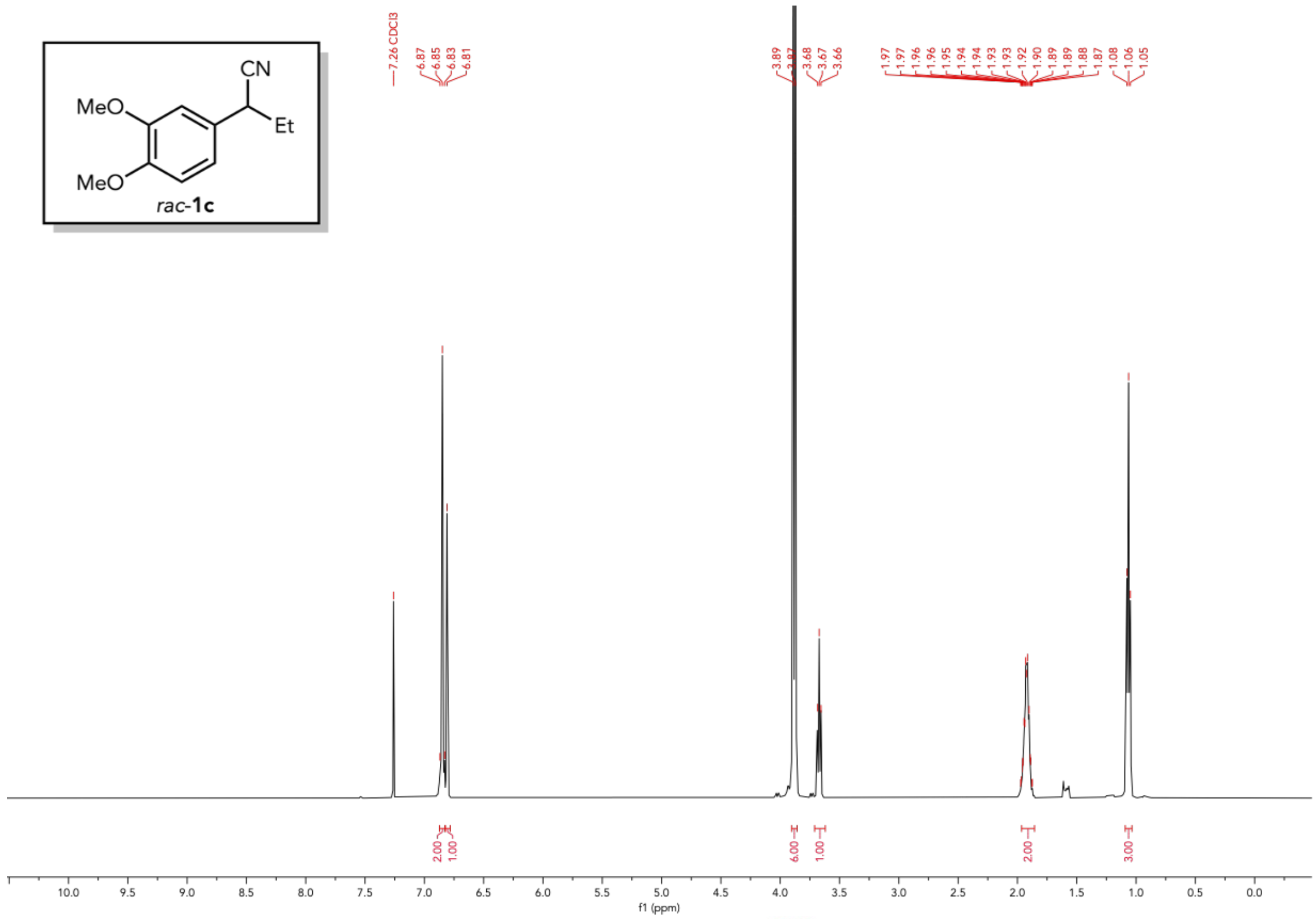

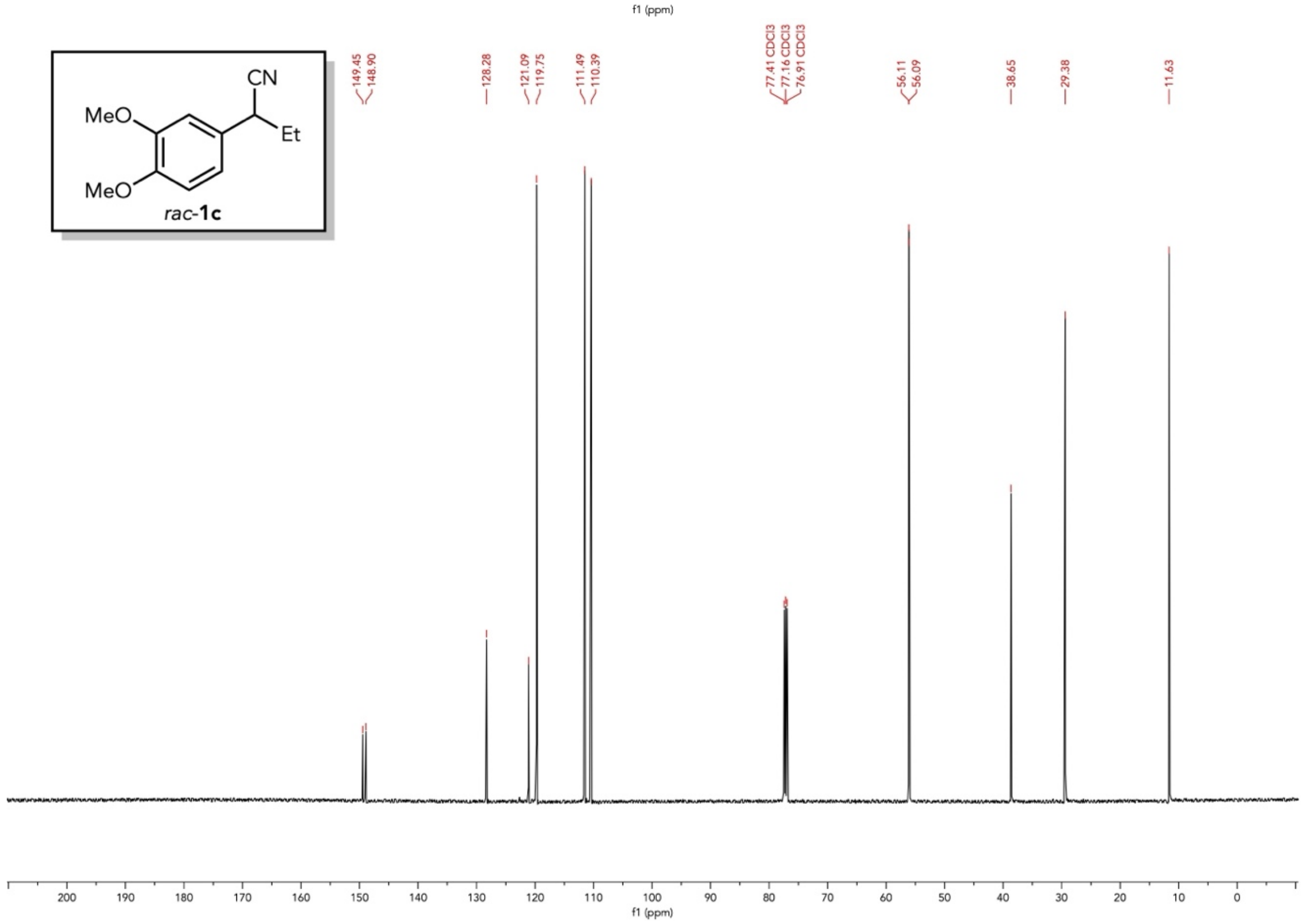

^{1}H NMR (500 MHz, $CDCl_3$) and ^{13}C NMR (126 MHz, $CDCl_3$) spectra for compound *rac*-**1d**

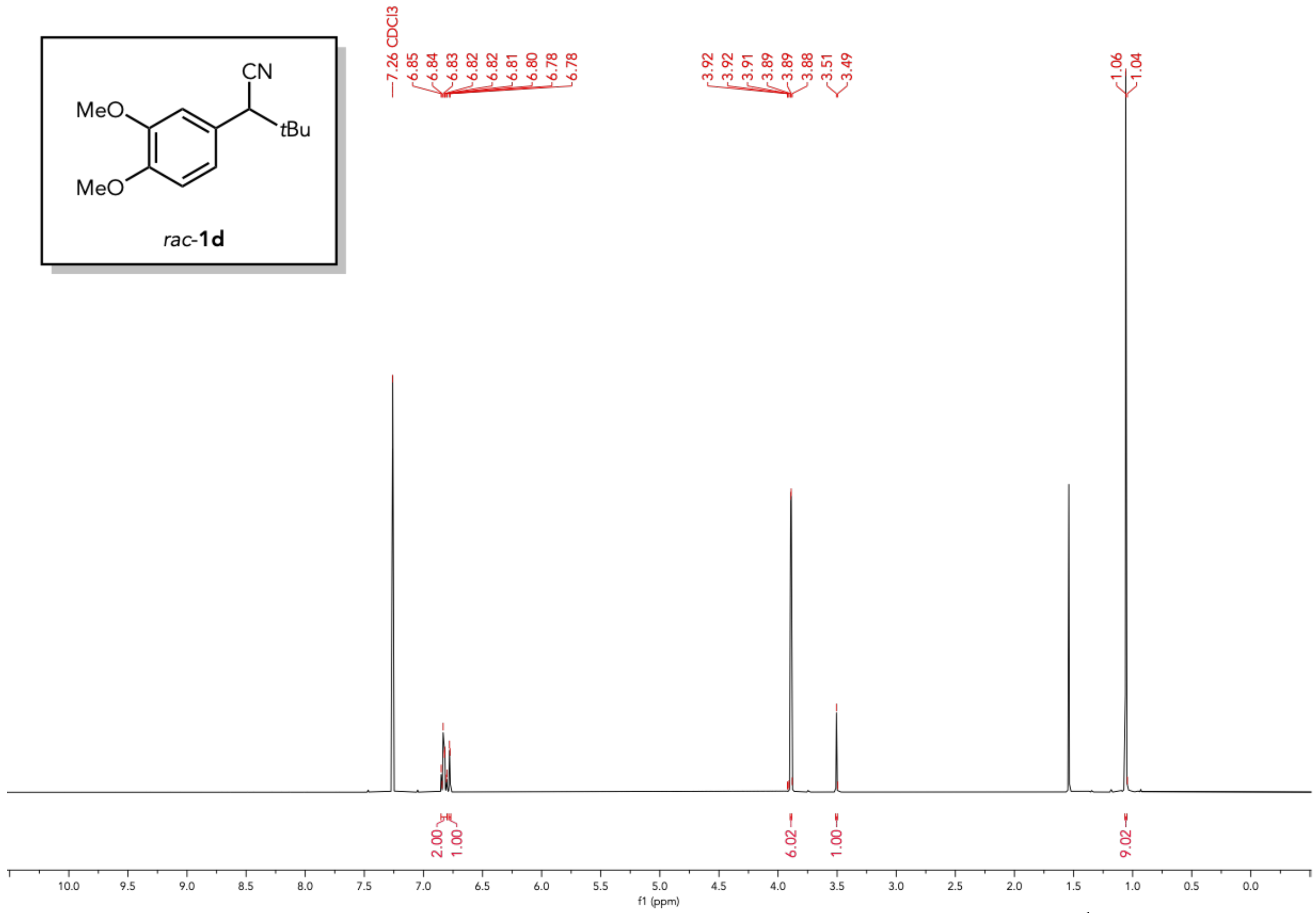

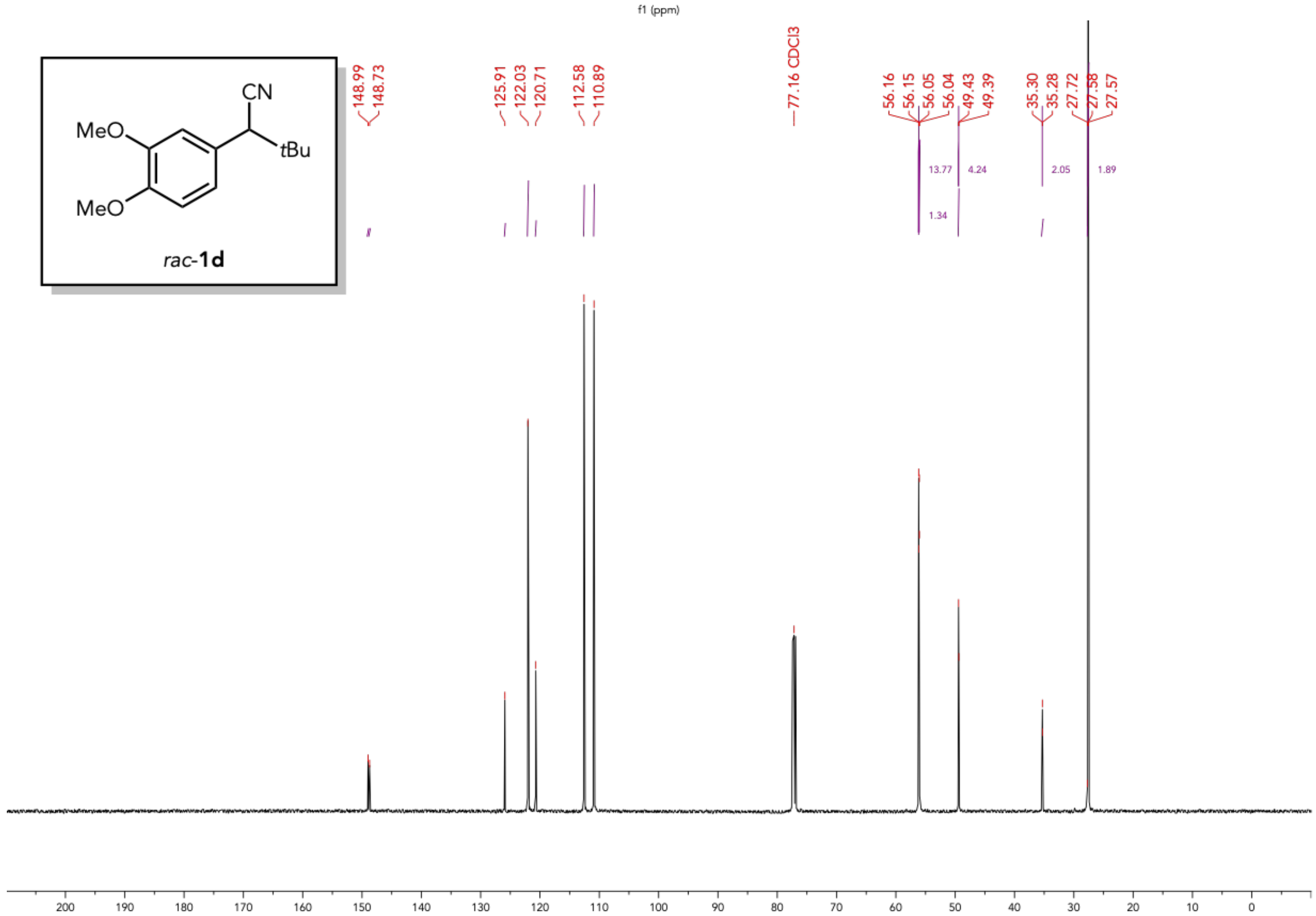

^{1}H NMR (500 MHz, $CDCl_3$) and ^{13}C NMR (126 MHz, $CDCl_3$) spectra for compound *rac*-**1e**

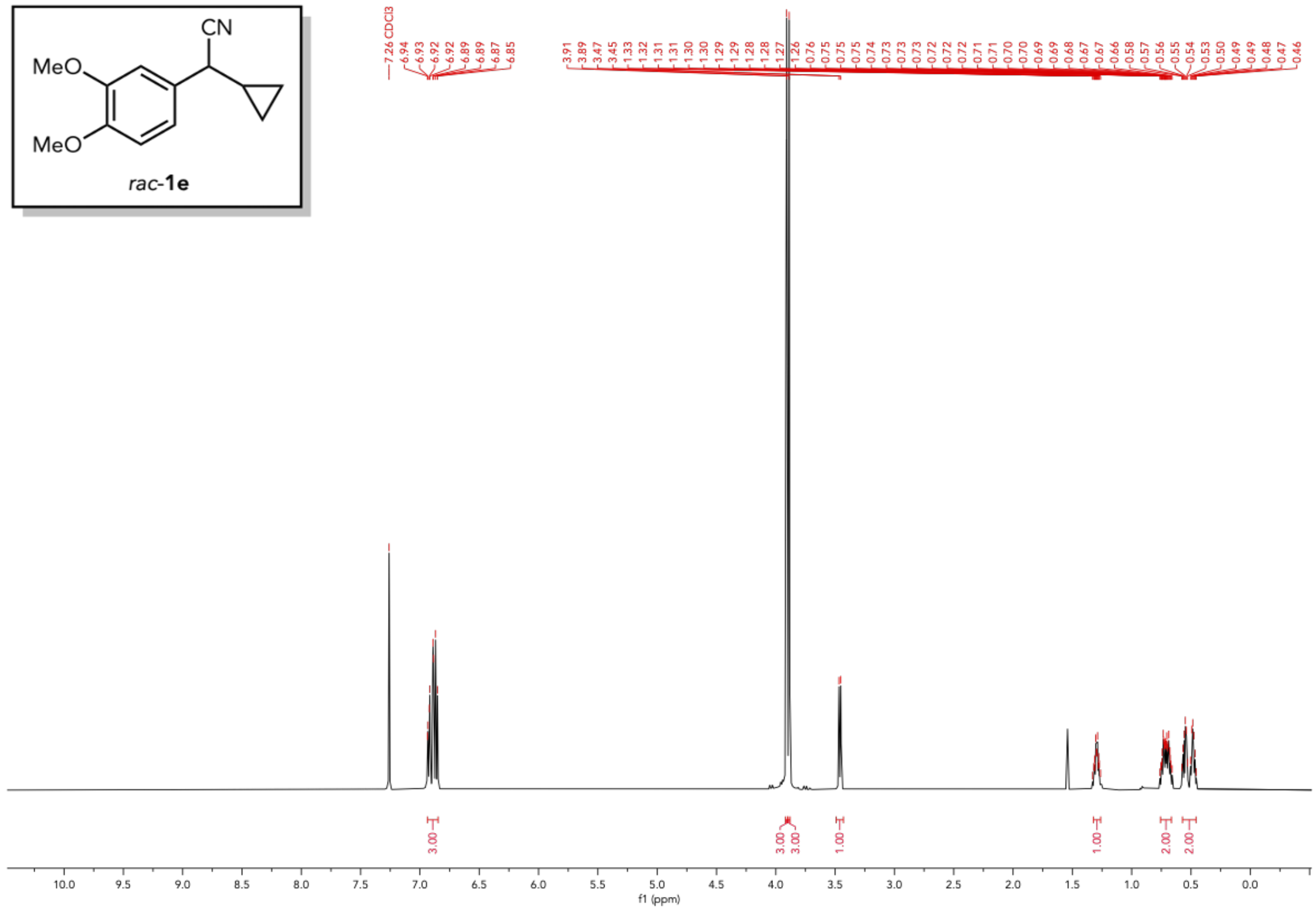

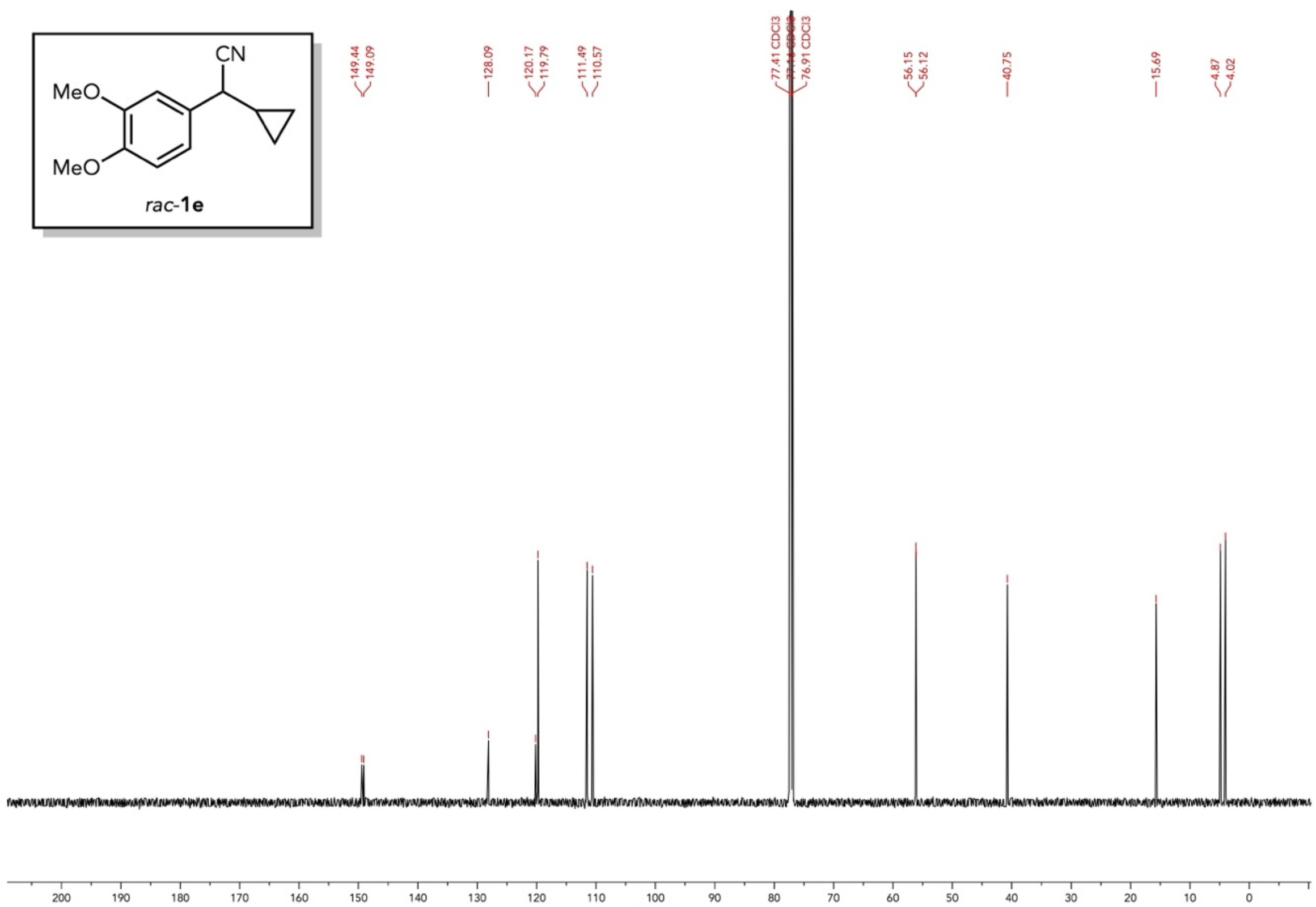

^{1}H NMR (500 MHz, $CDCl_3$) and ^{13}C NMR (126 MHz, $CDCl_3$) spectra for compound *rac*-**1f**

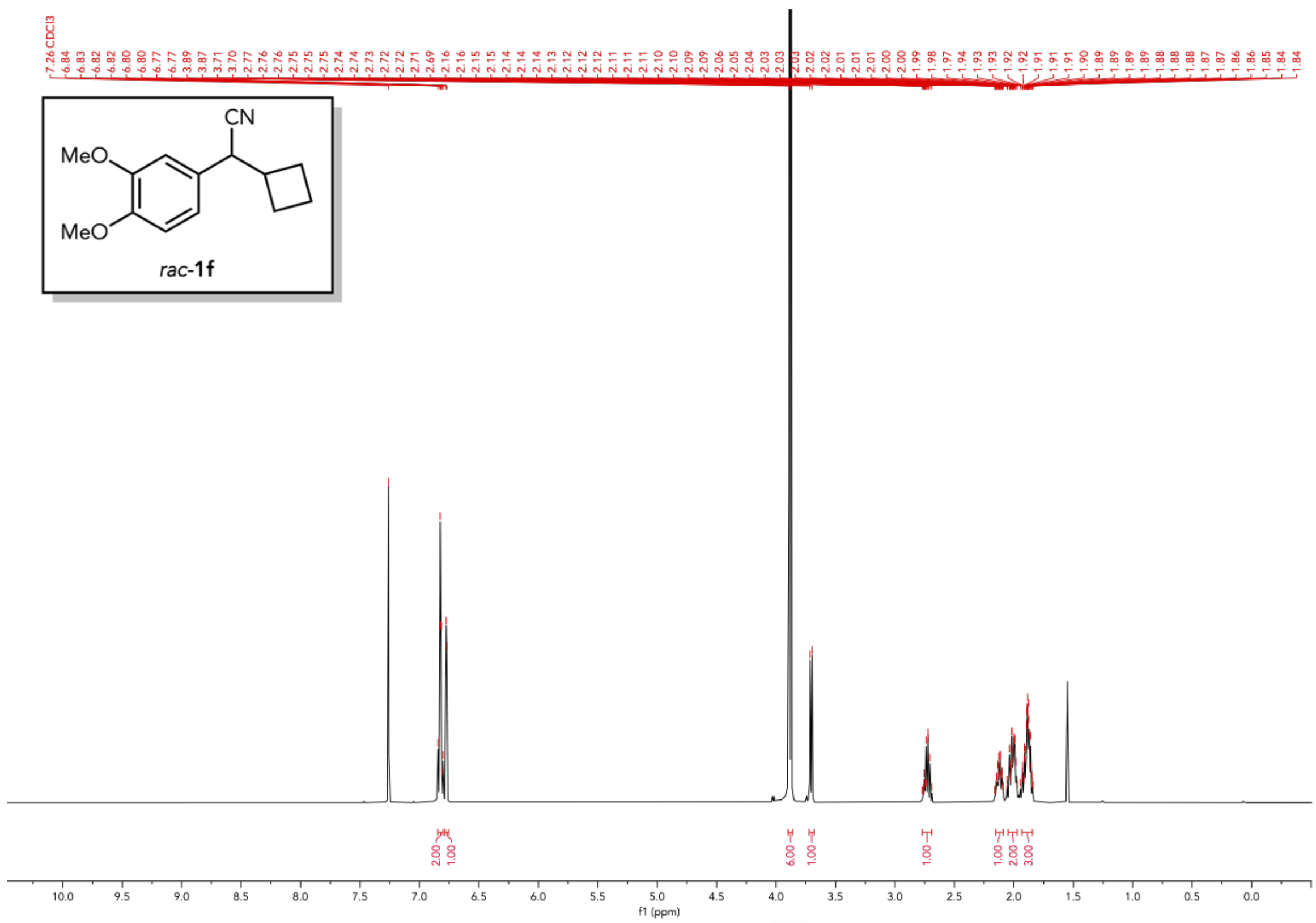

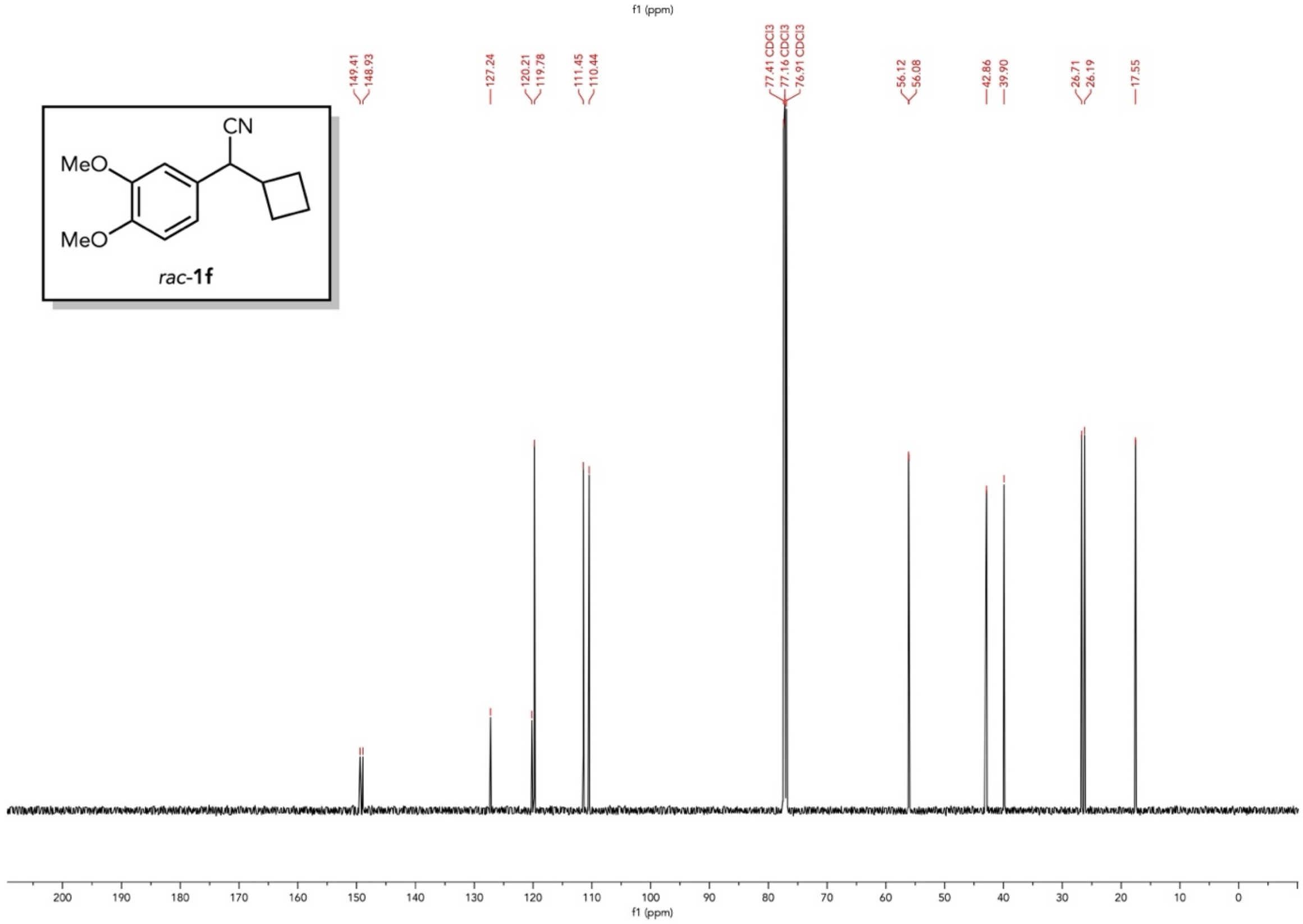

^{1}H NMR (500 MHz, $CDCl_3$) and ^{13}C NMR (126 MHz, $CDCl_3$) spectra for compound *rac*-**1g**

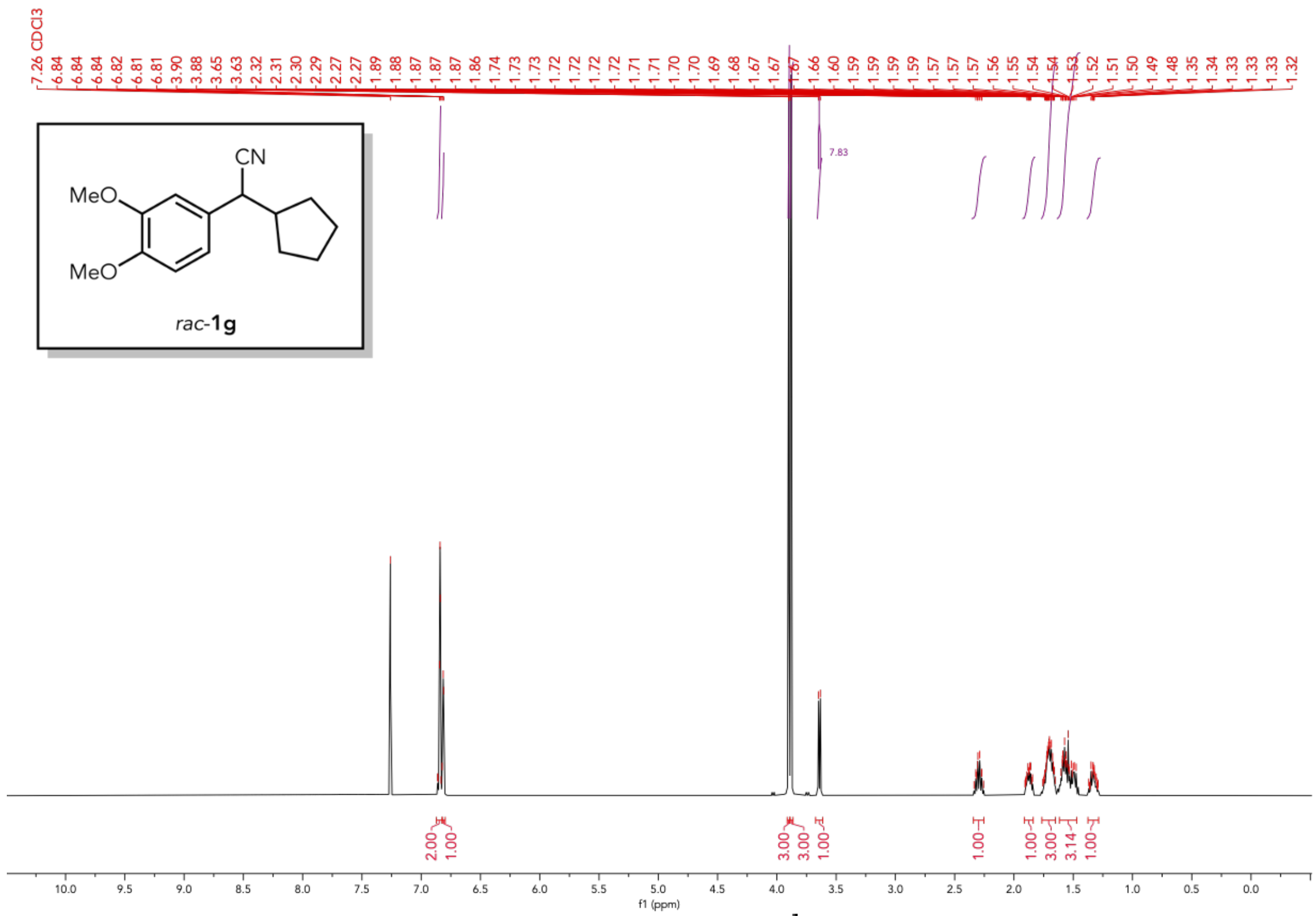

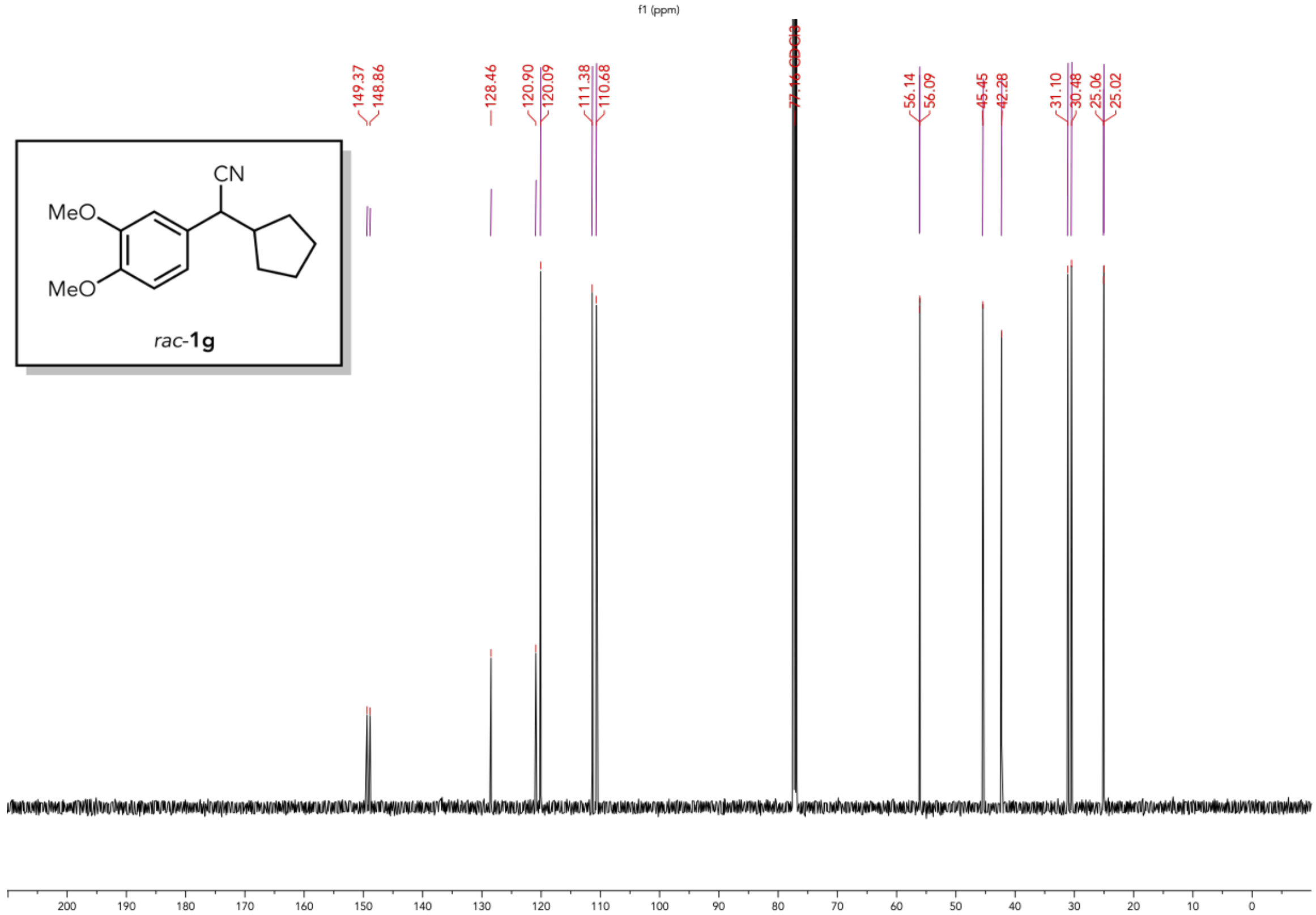

^{1}H NMR (500 MHz, $CDCl_3$) and ^{13}C NMR (126 MHz, $CDCl_3$) spectra for compound *rac*-**1h**

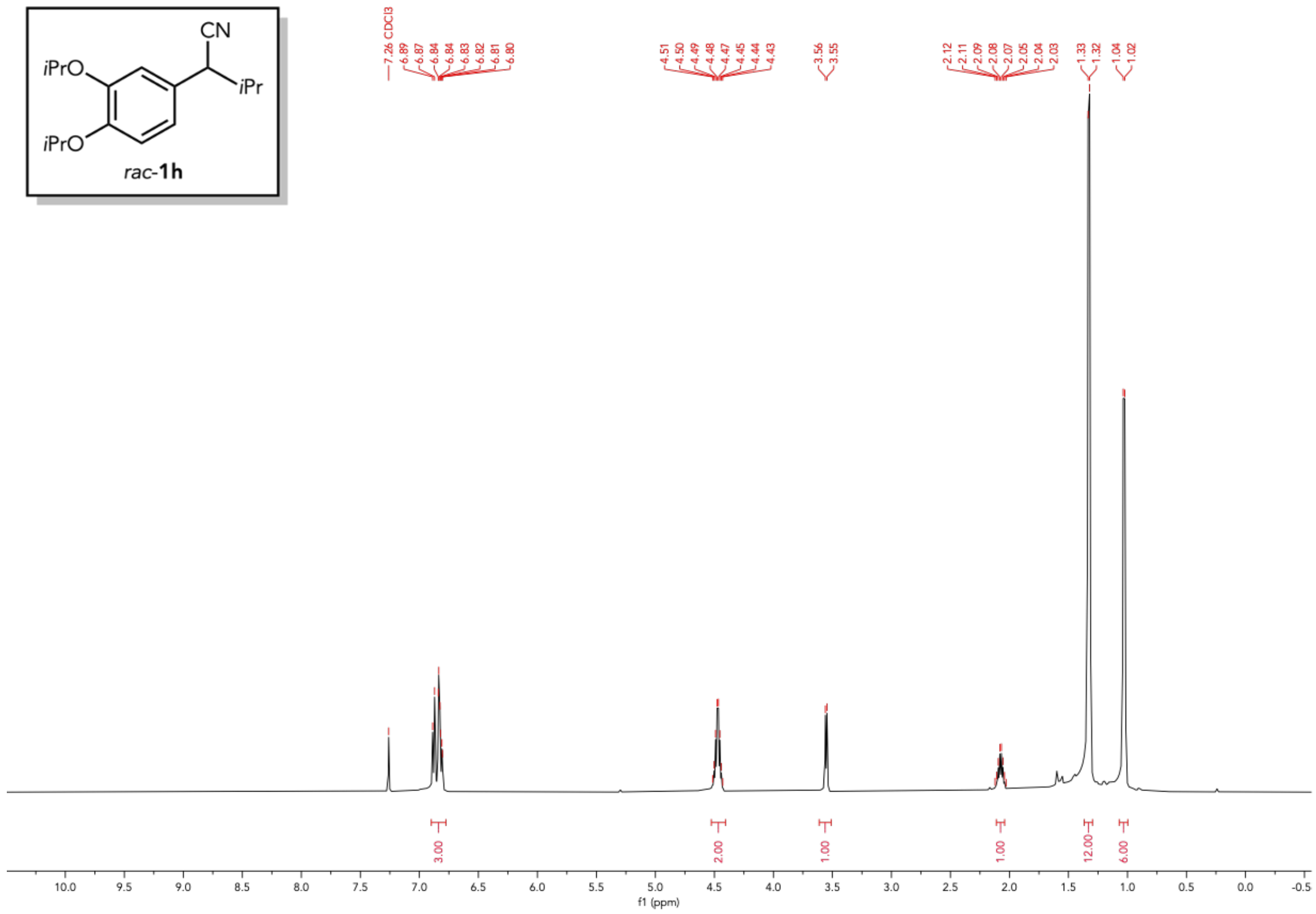

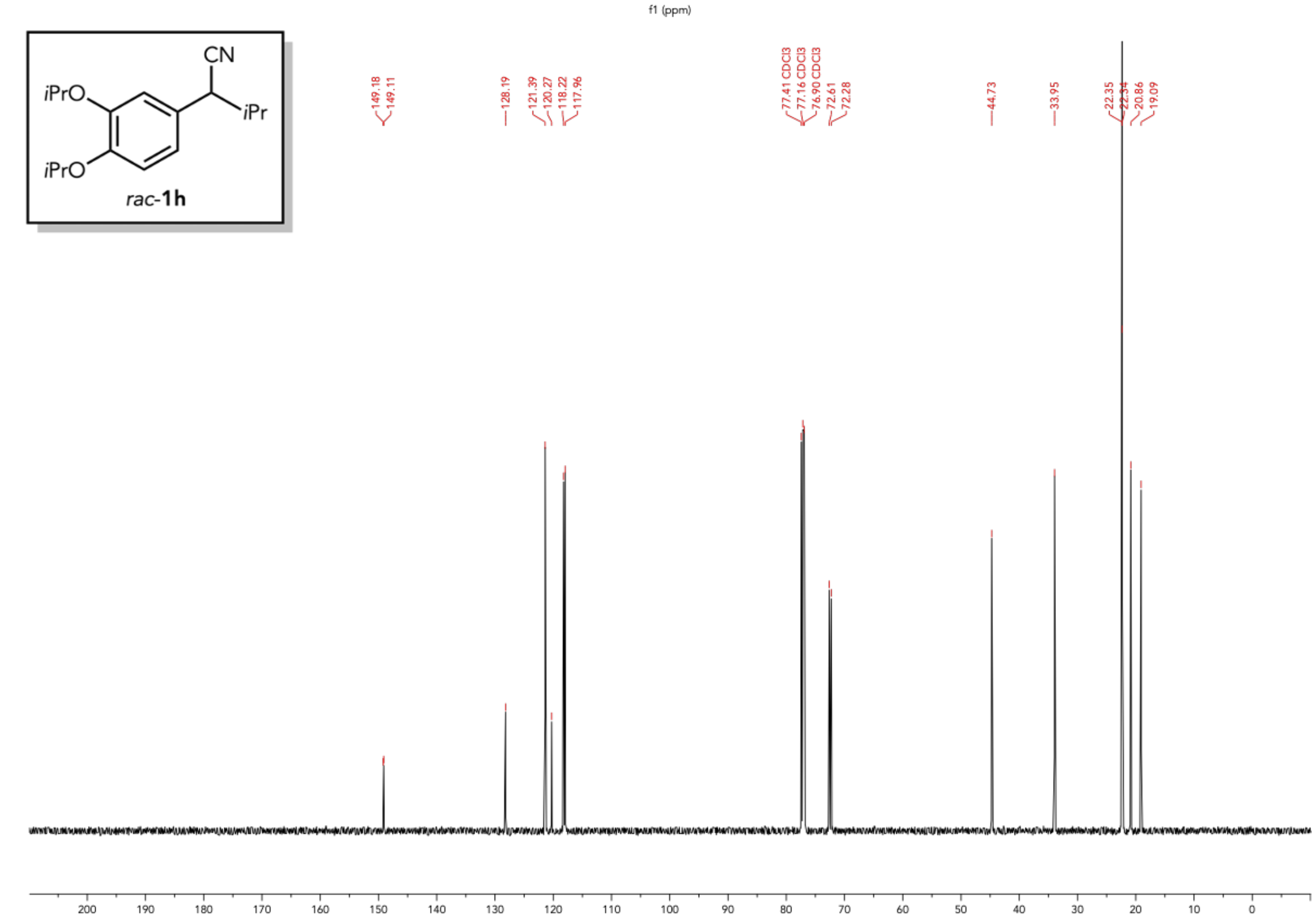

^{1}H NMR (500 MHz, $CDCl_3$) and ^{13}C NMR (126 MHz, $CDCl_3$) spectra for compound *rac*-**1i**

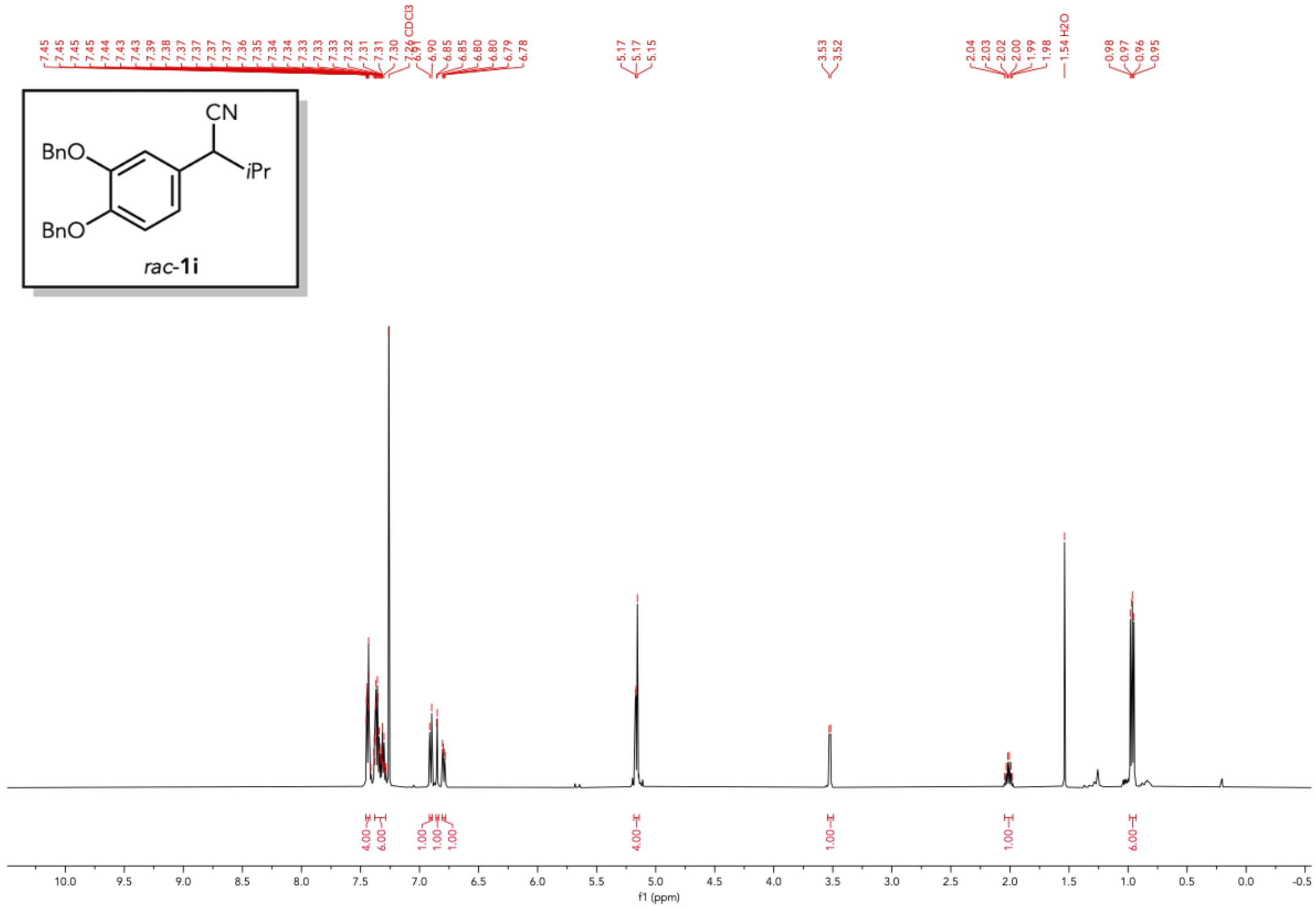

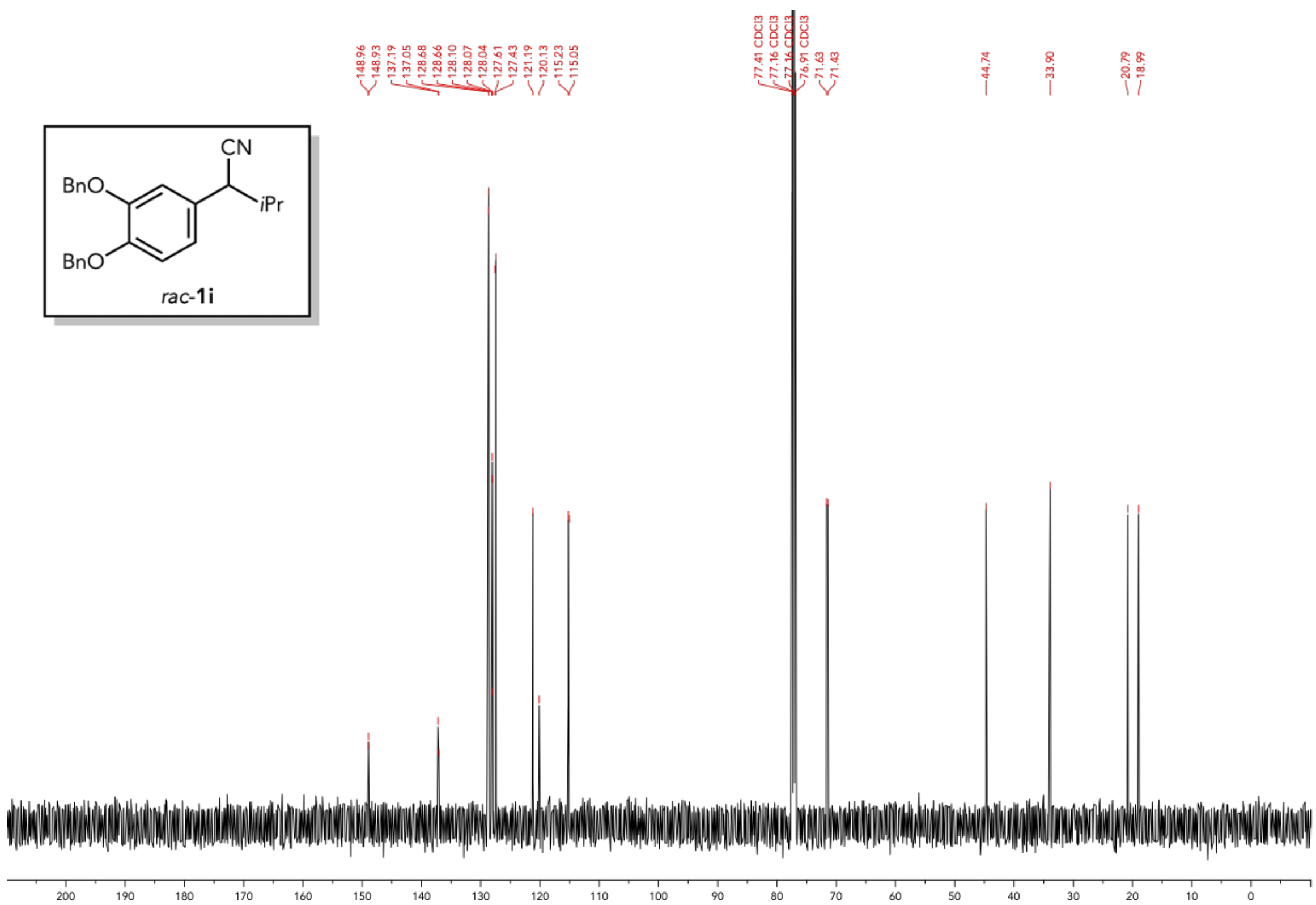

^{1}H NMR (500 MHz, $CDCl_3$) and ^{13}C NMR (126 MHz, $CDCl_3$) spectra for compound *rac*-**1j**

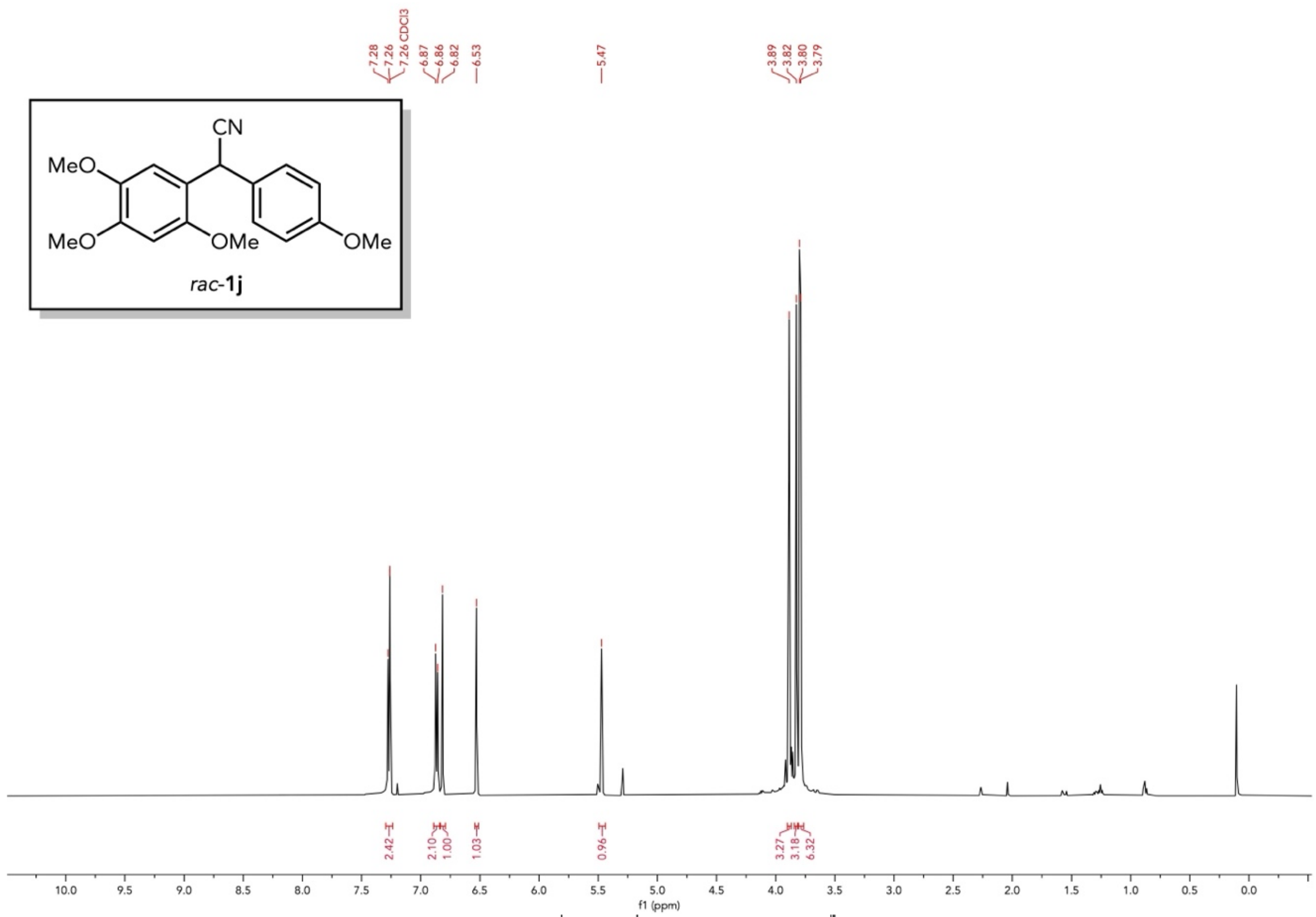

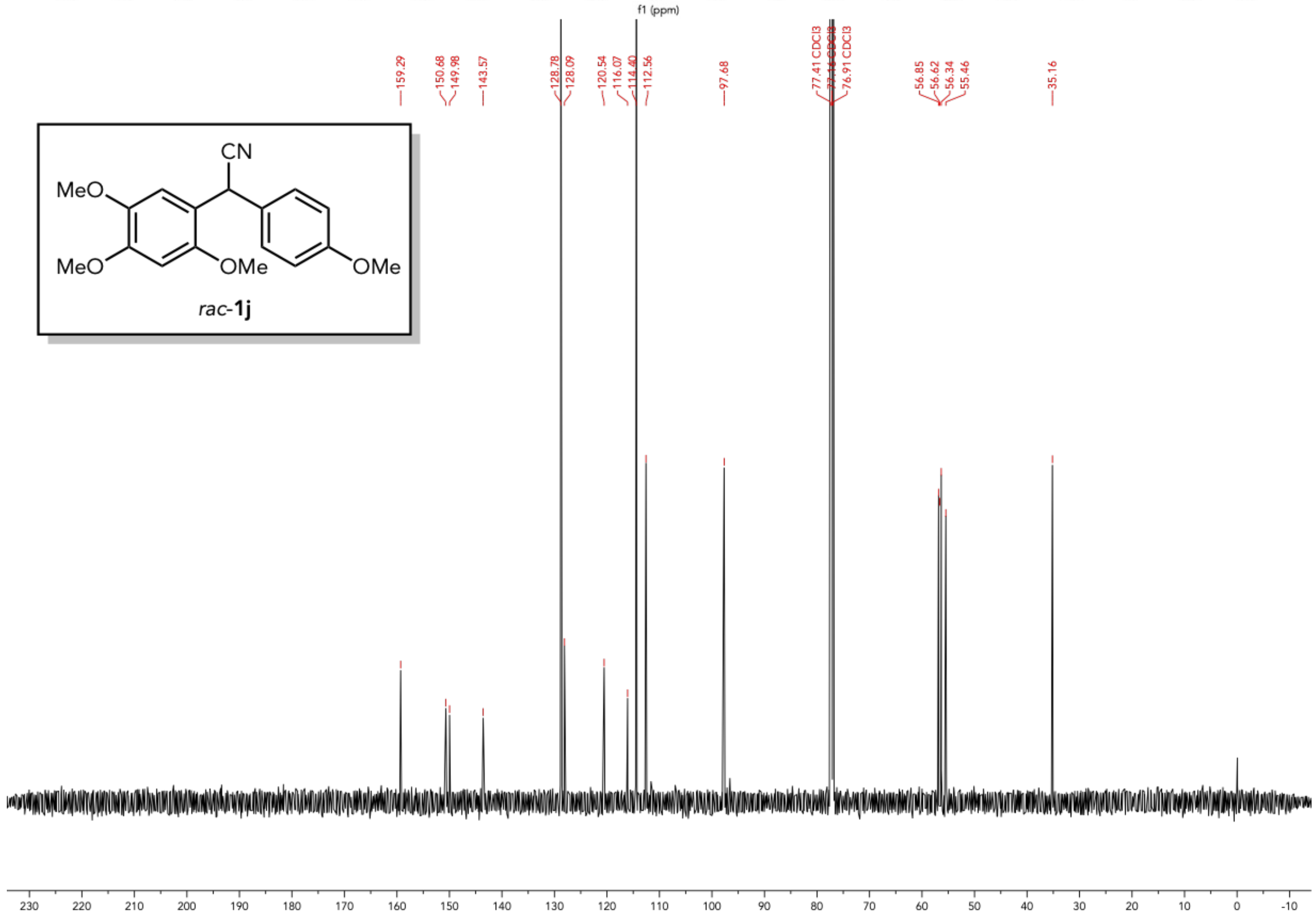

^{1}H NMR (500 MHz, $CDCl_3$) and ^{13}C NMR (126 MHz, $CDCl_3$) spectra for compound *rac*-**1k**

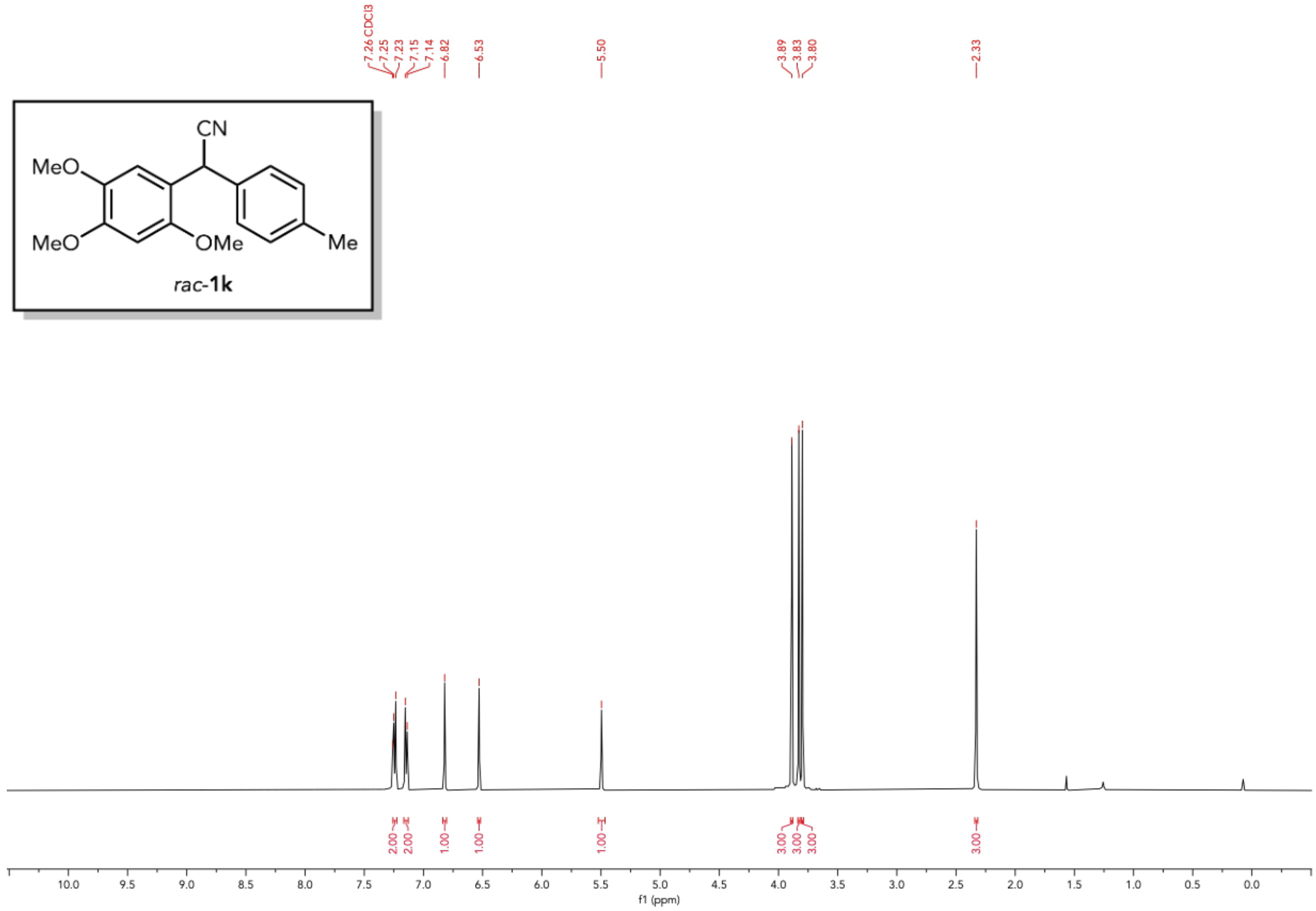

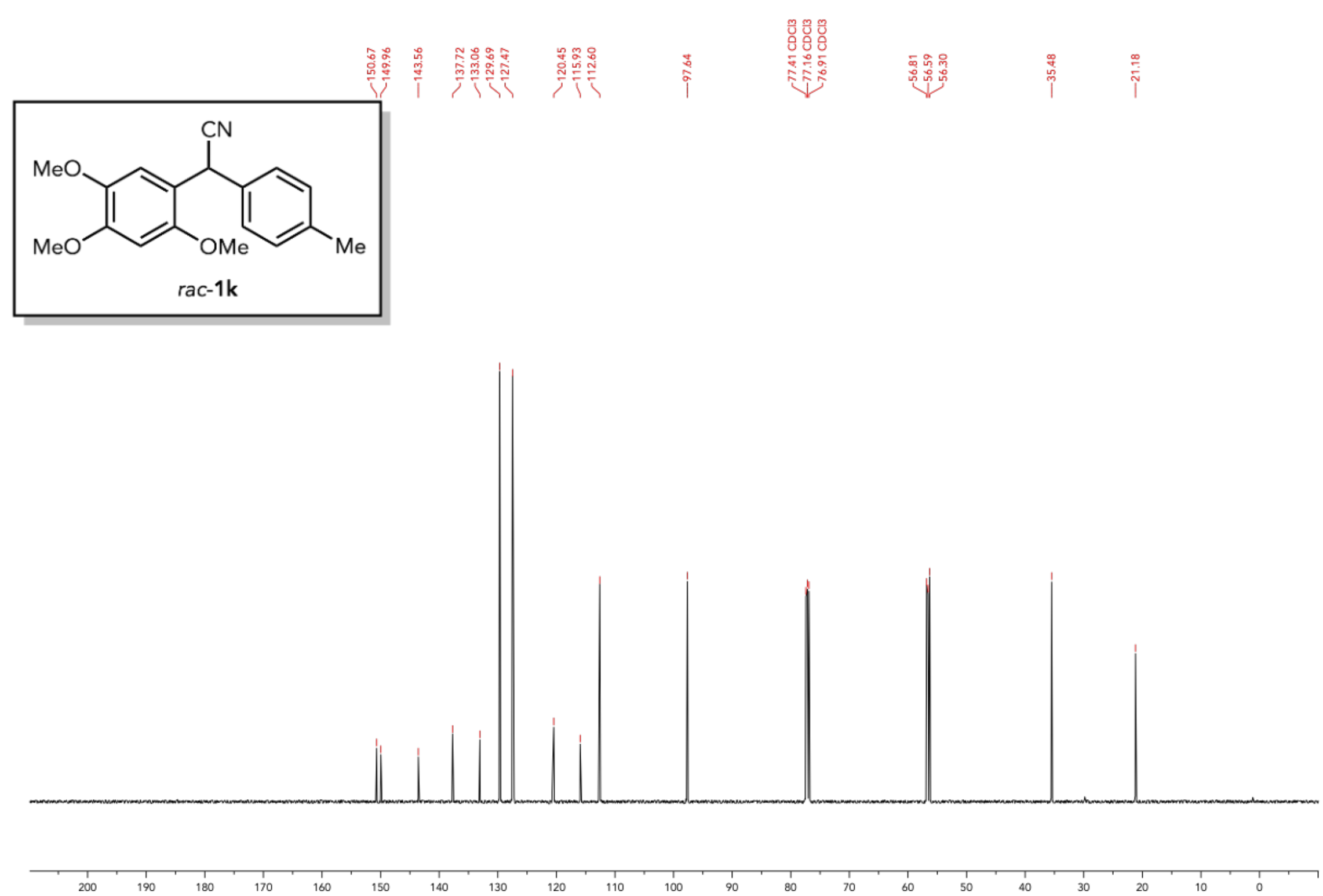

^{1}H NMR (500 MHz, $CDCl_3$) and ^{13}C NMR (126 MHz, $CDCl_3$) spectra for compound *rac*-**1l**

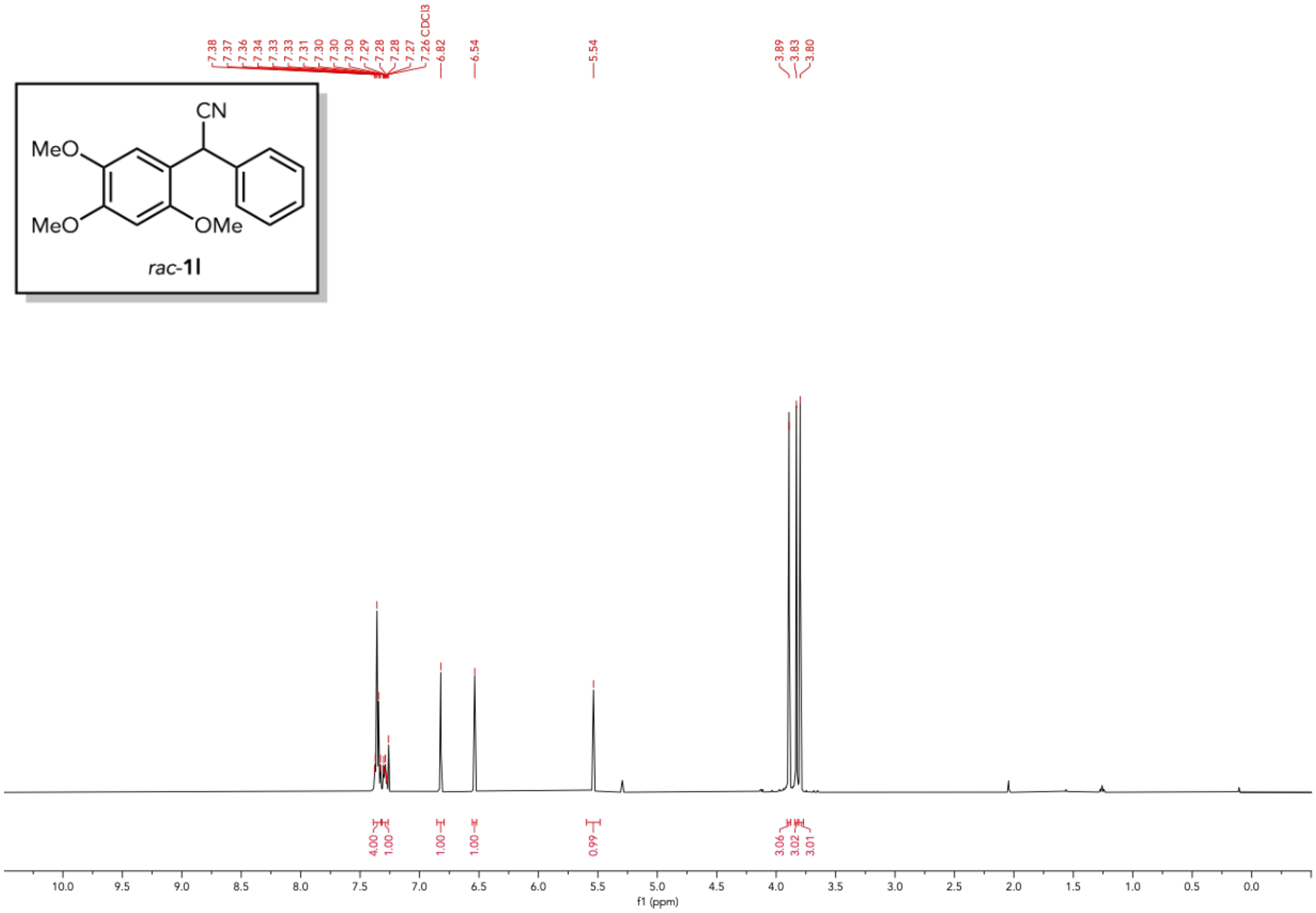

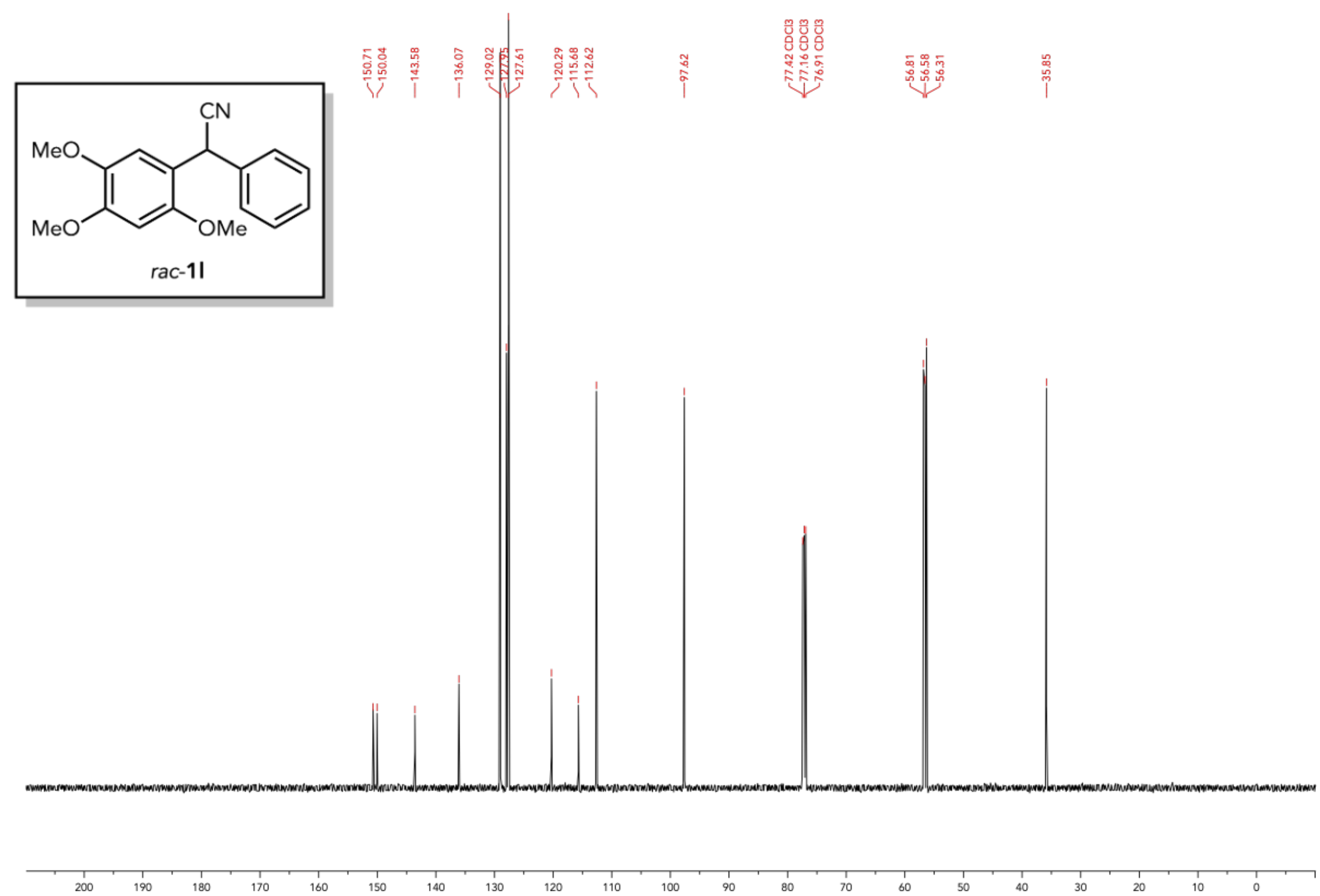

^{1}H NMR (500 MHz, $CDCl_3$) and ^{13}C NMR (126 MHz, $CDCl_3$) spectra for compound *rac*-**1m**

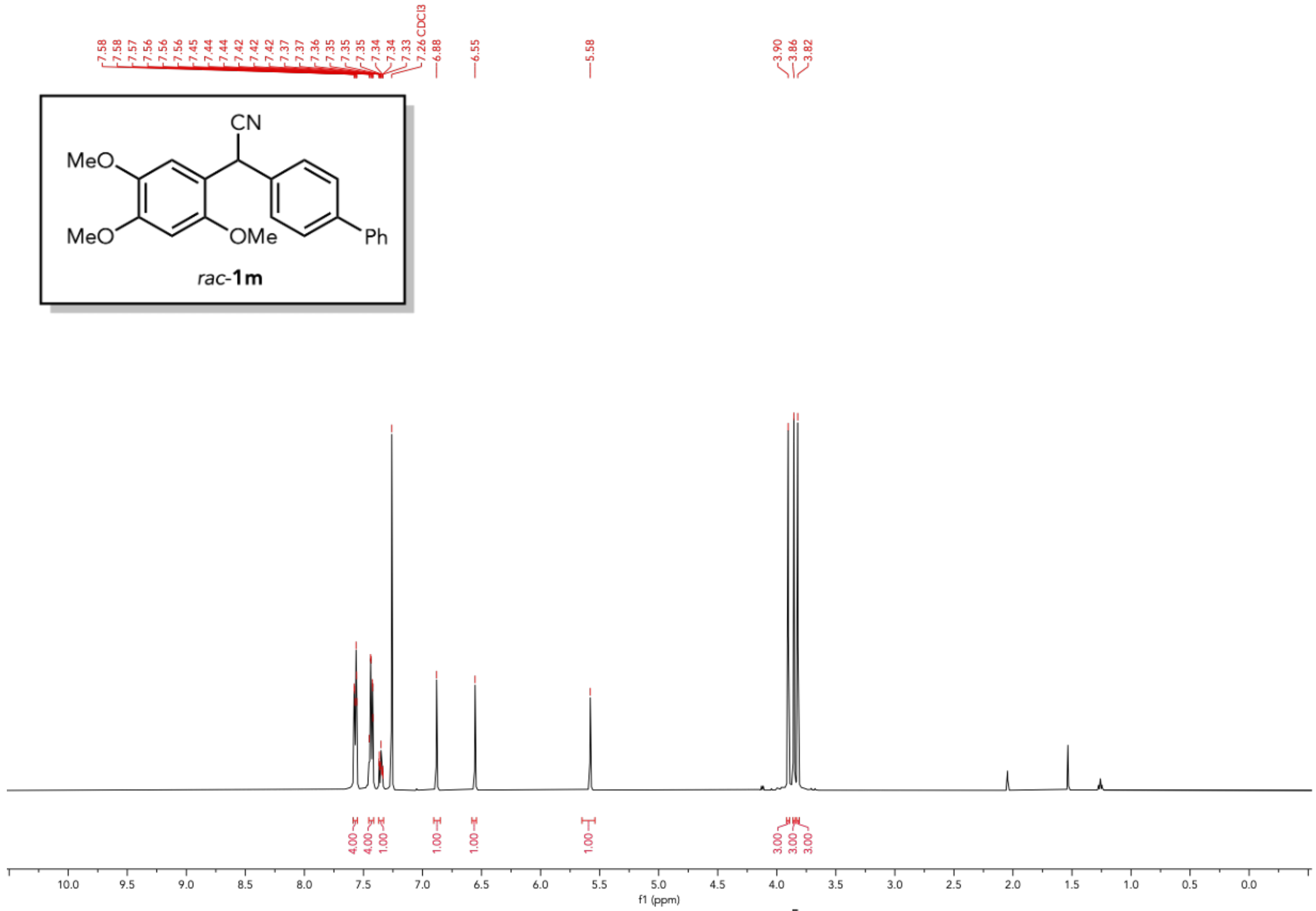

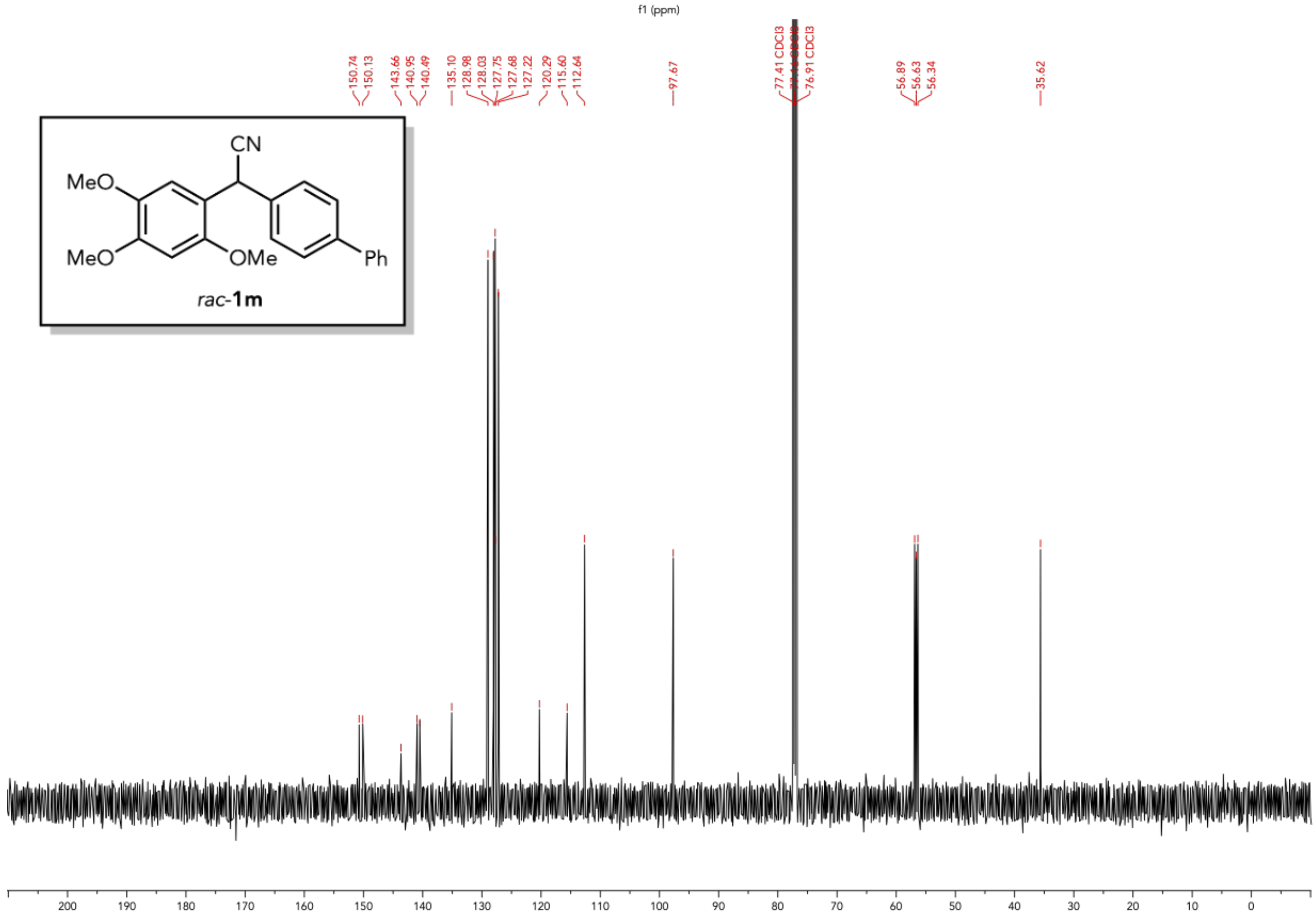

^{1}H NMR (500 MHz, $CDCl_3$) and ^{13}C NMR (126 MHz, $CDCl_3$) spectra for compound *rac*-**1n**

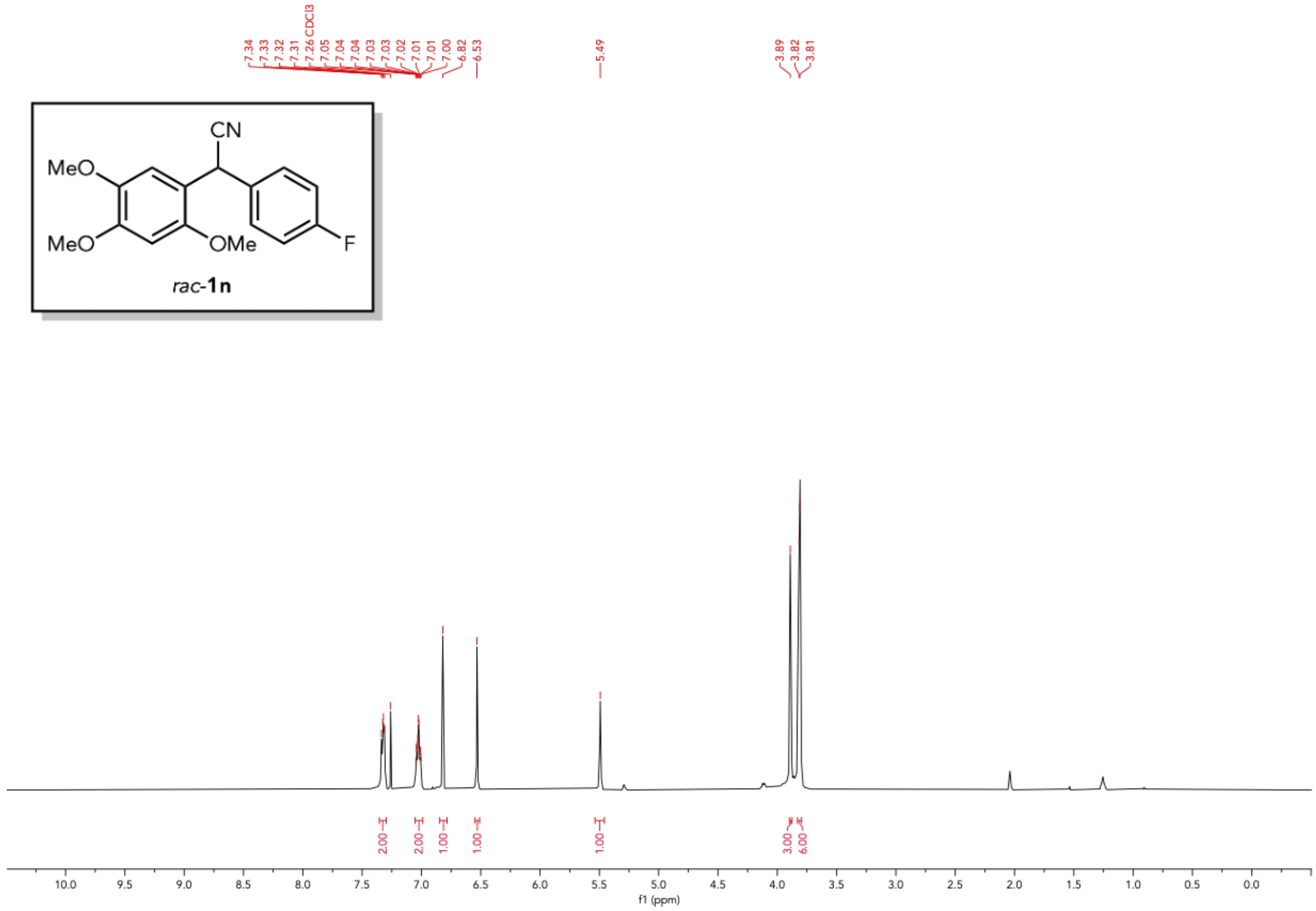

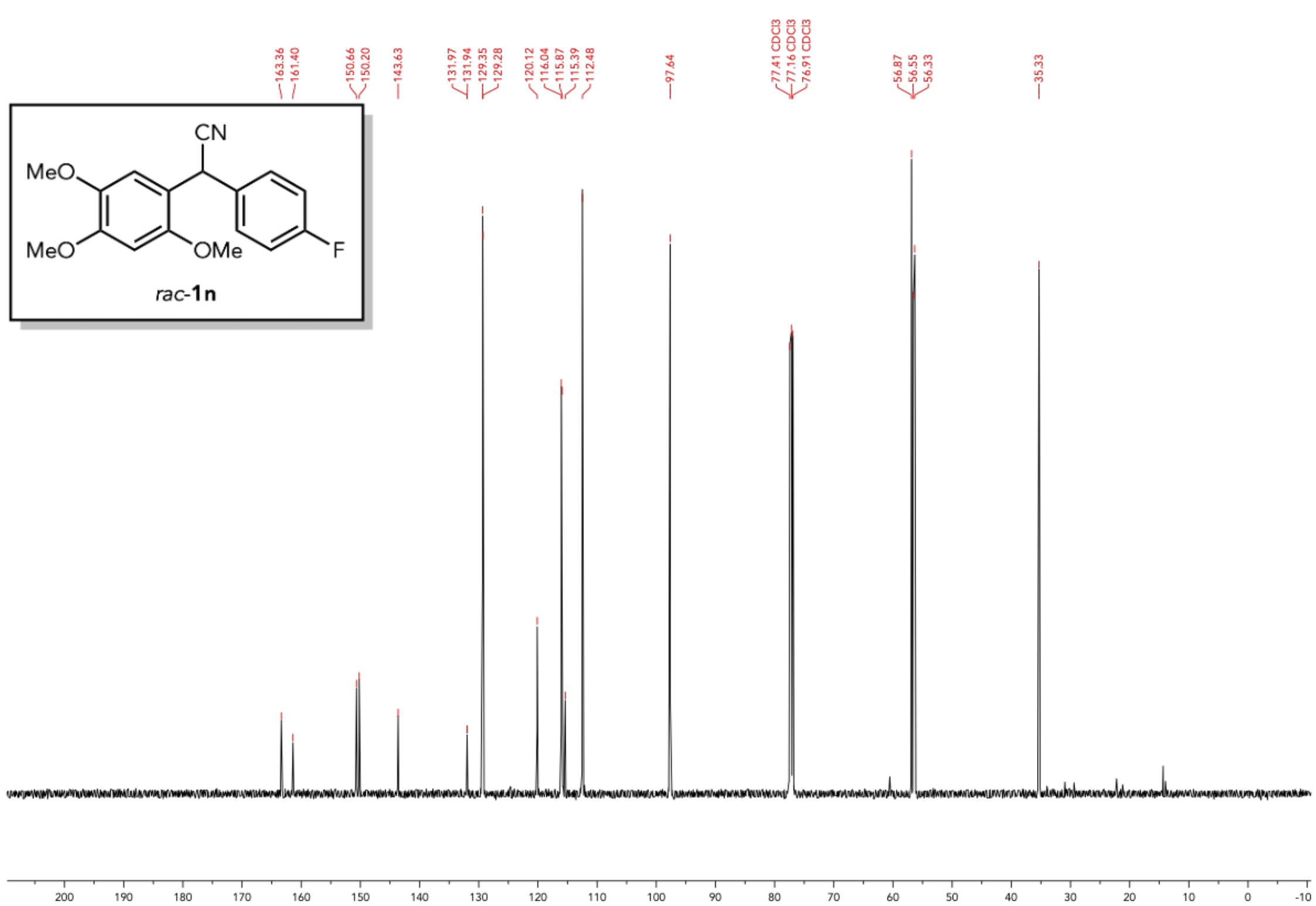

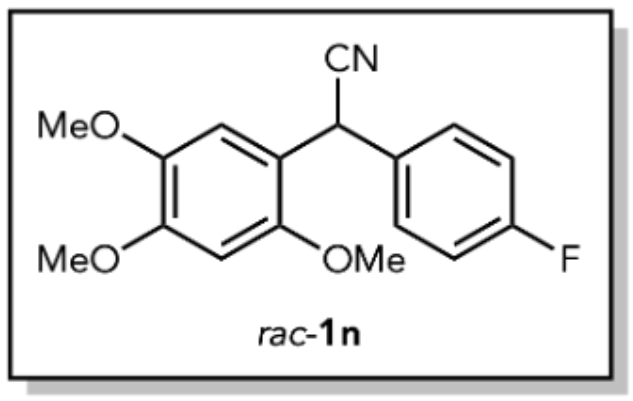
CN
MeO
MeO
OMe
F
rac-1n

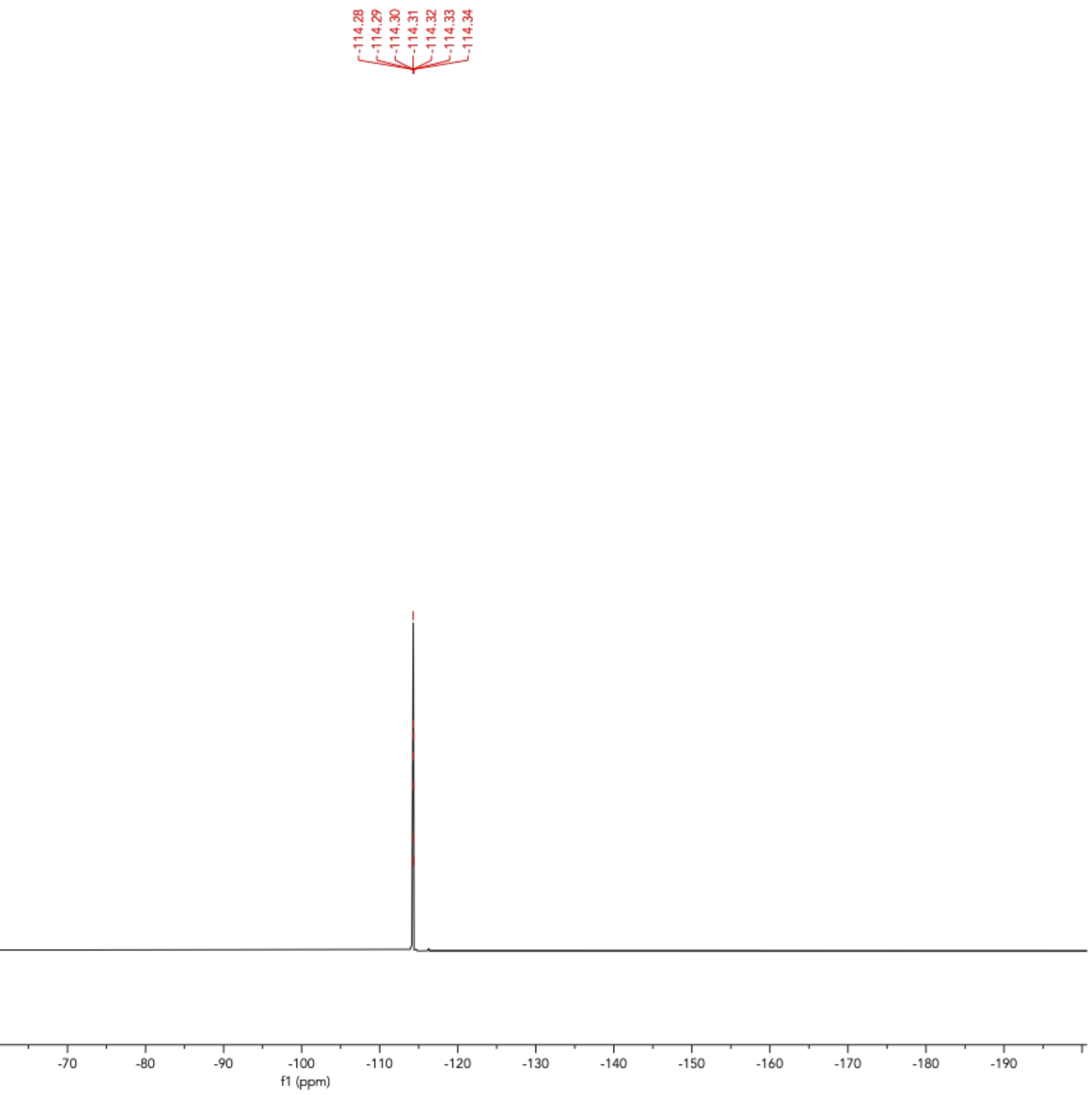
-114.28
-114.29
-114.30
-114.31
-114.32
-114.33
-114.34
-10
-20
-30
-40
-50
-60
-70
-80
-90
-100
-110
-120
-130
-140
-150
-160
-170
-180
-190
f1 (ppm)

^{1}H NMR (500 MHz, $CDCl_3$) and ^{13}C NMR (126 MHz, $CDCl_3$) spectra for compound *rac*-**1o**

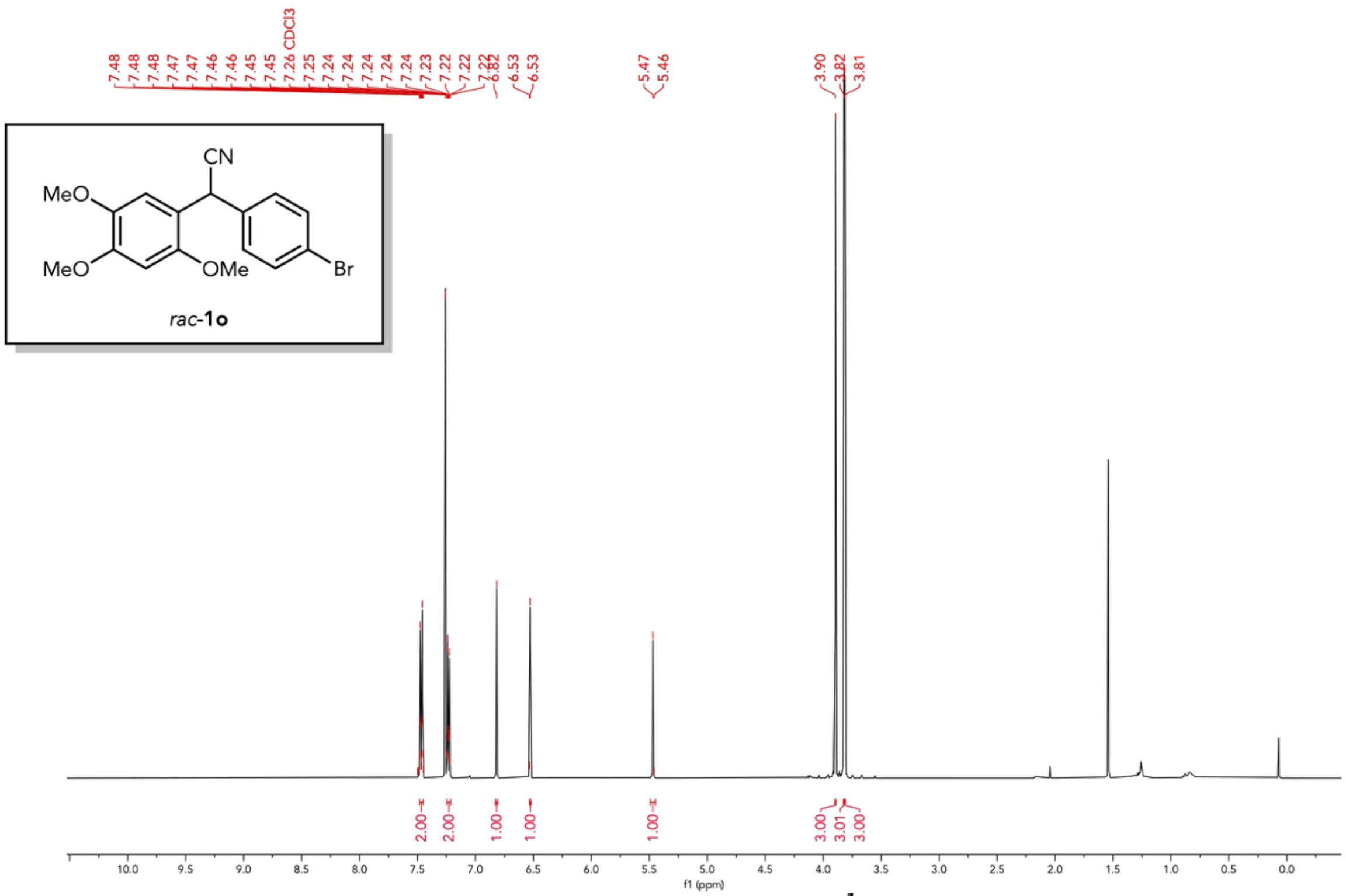

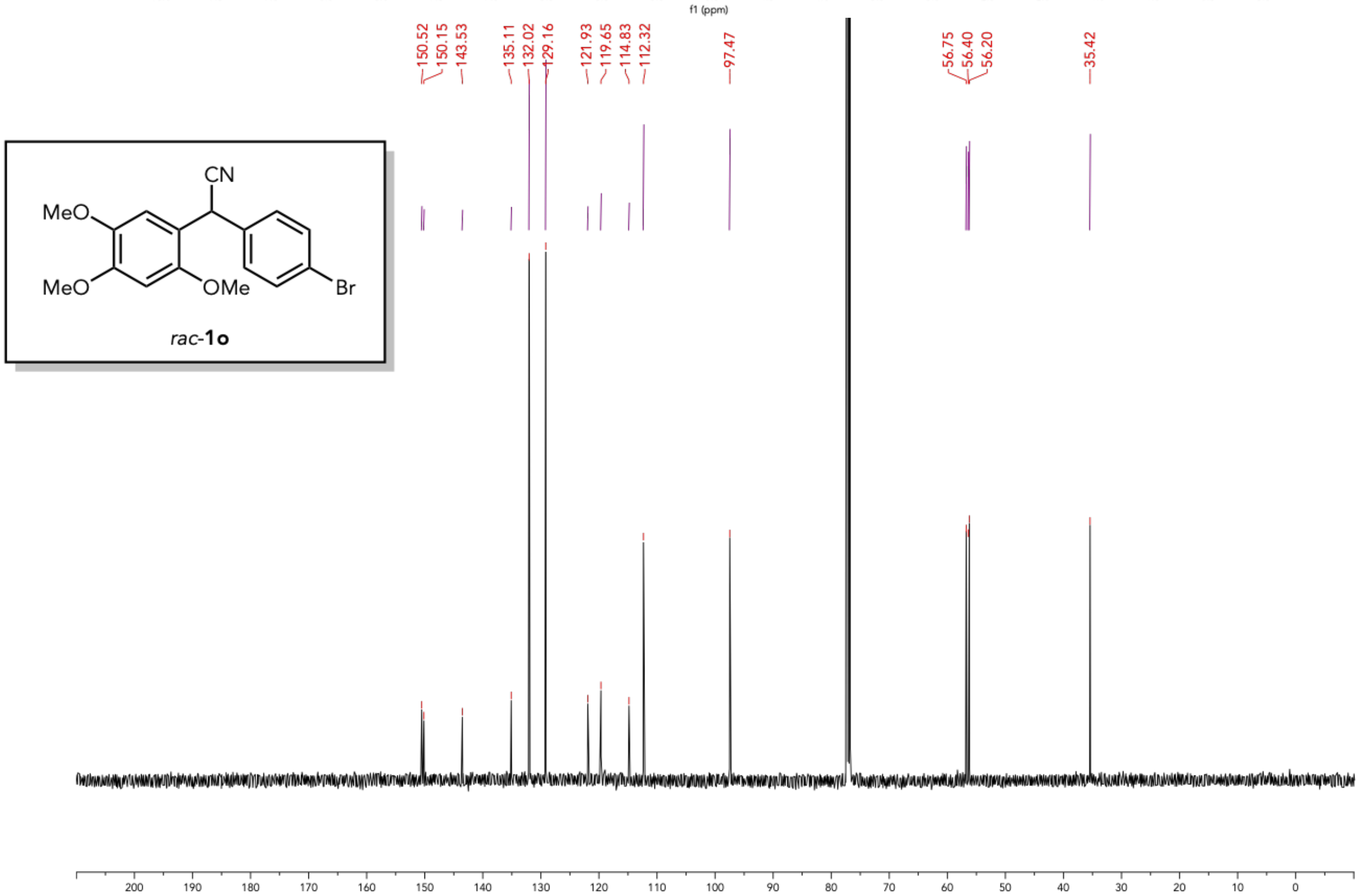

^{1}H NMR (500 MHz, $CDCl_3$) and ^{13}C NMR (126 MHz, $CDCl_3$) spectra for compound *rac*-**1p**

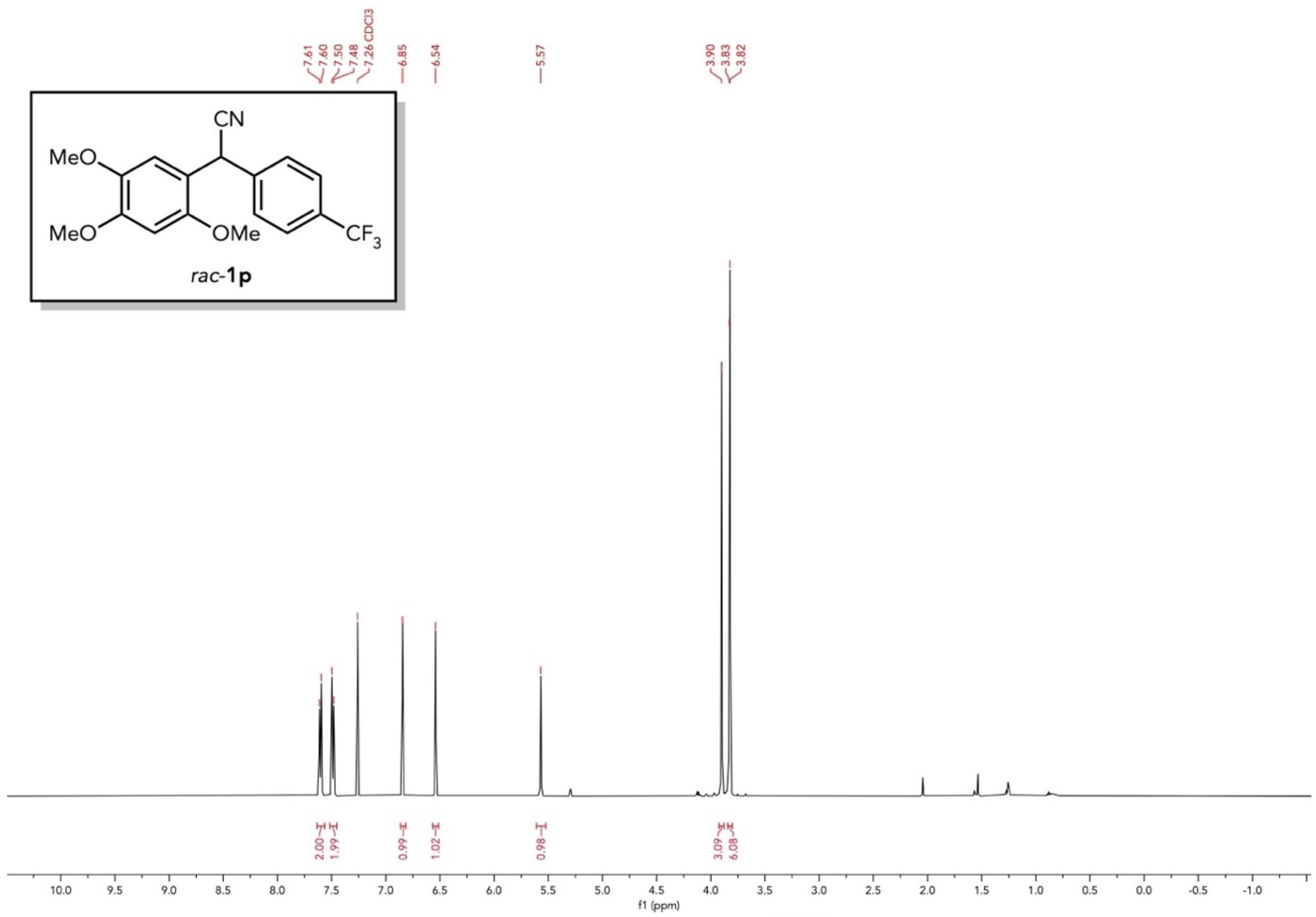

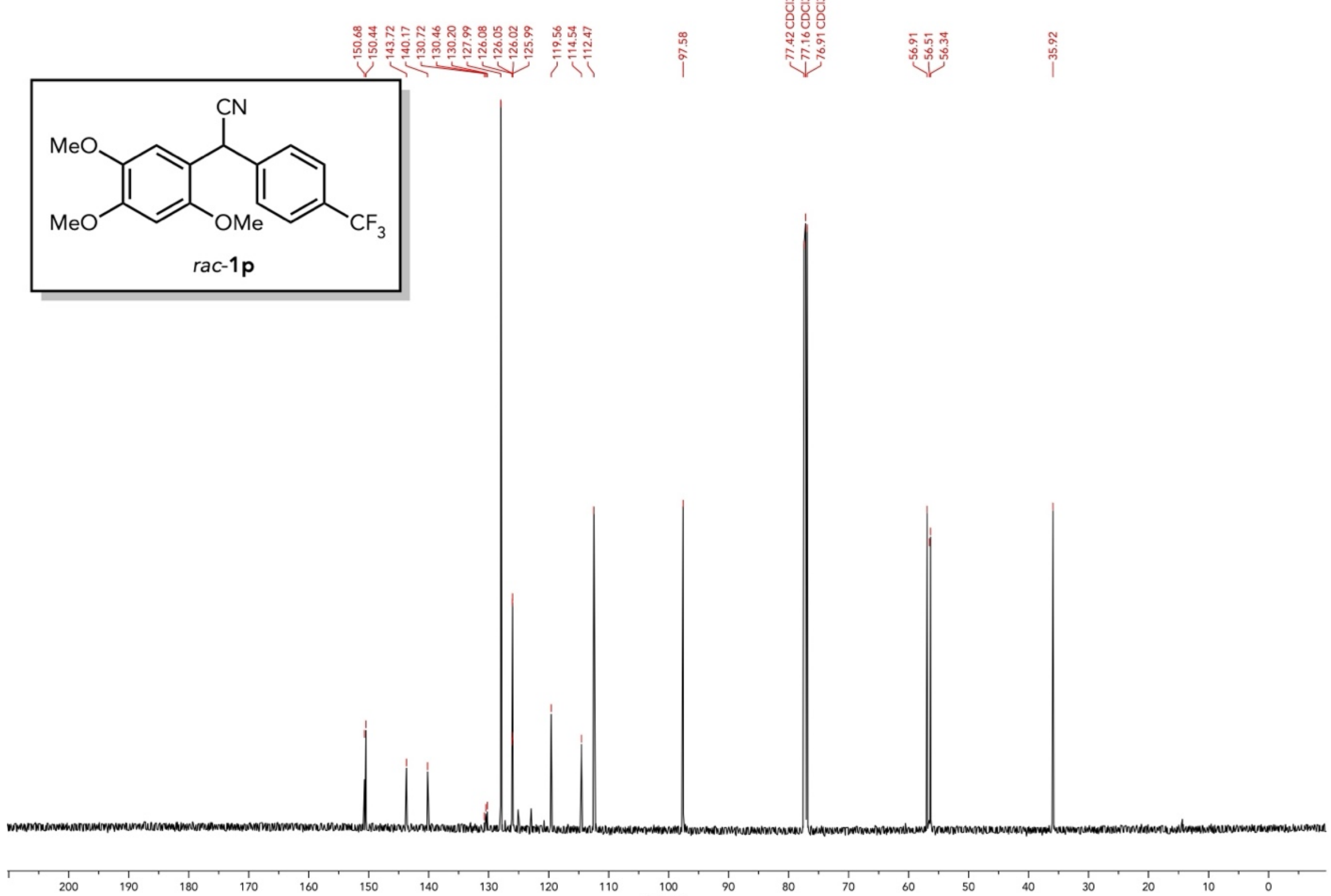

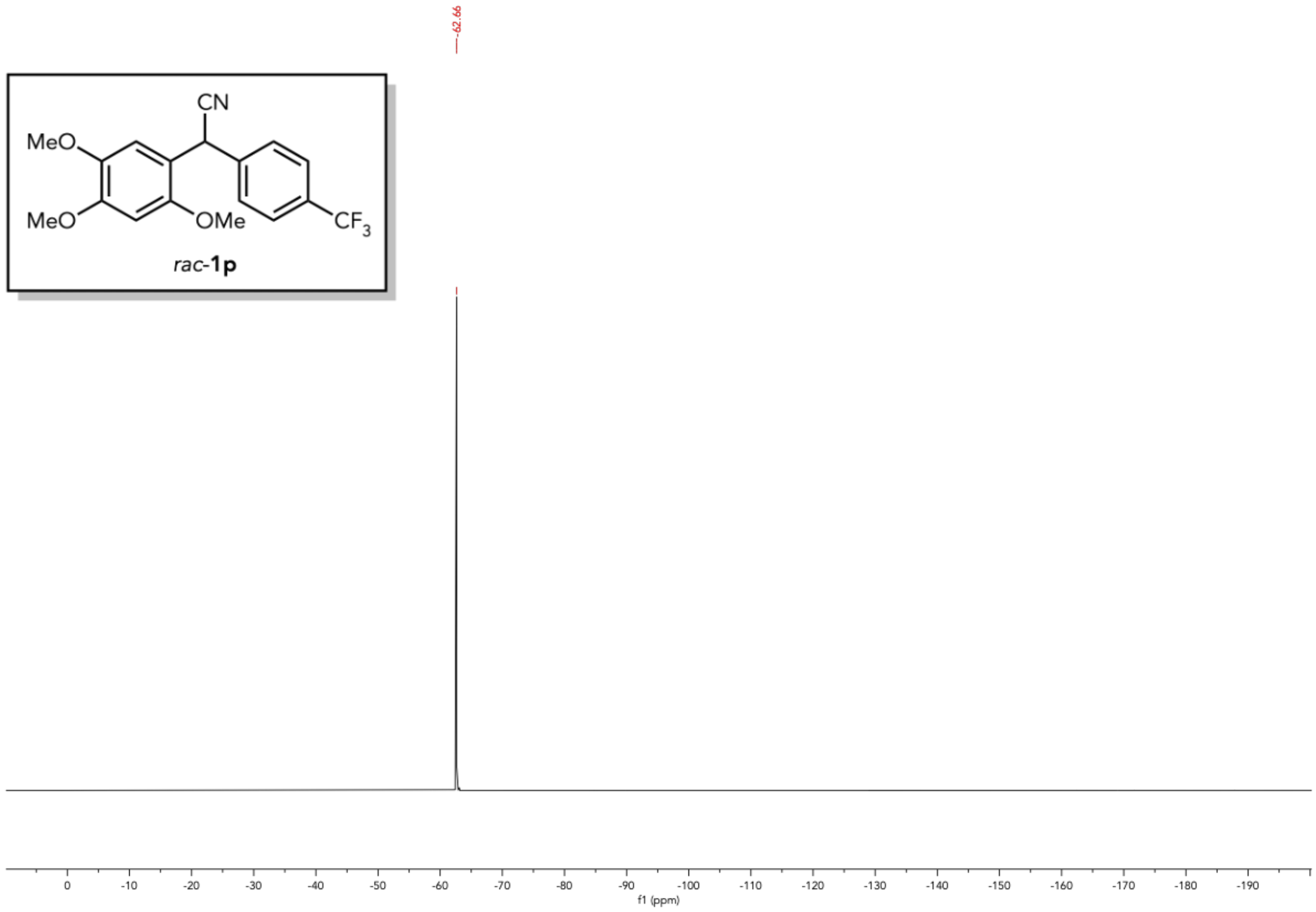
-62.66
CN
MeO
MeO
OMe
CF3
rac-1p
0
-10
-20
-30
-40
-50
-60
-70
-80
-90
-100
-110
-120
-130
-140
-150
-160
-170
-180
-190
f1 (ppm)

^{1}H NMR (500 MHz, $CDCl_3$) and ^{13}C NMR (126 MHz, $CDCl_3$) spectra for compound *rac*-**1q**

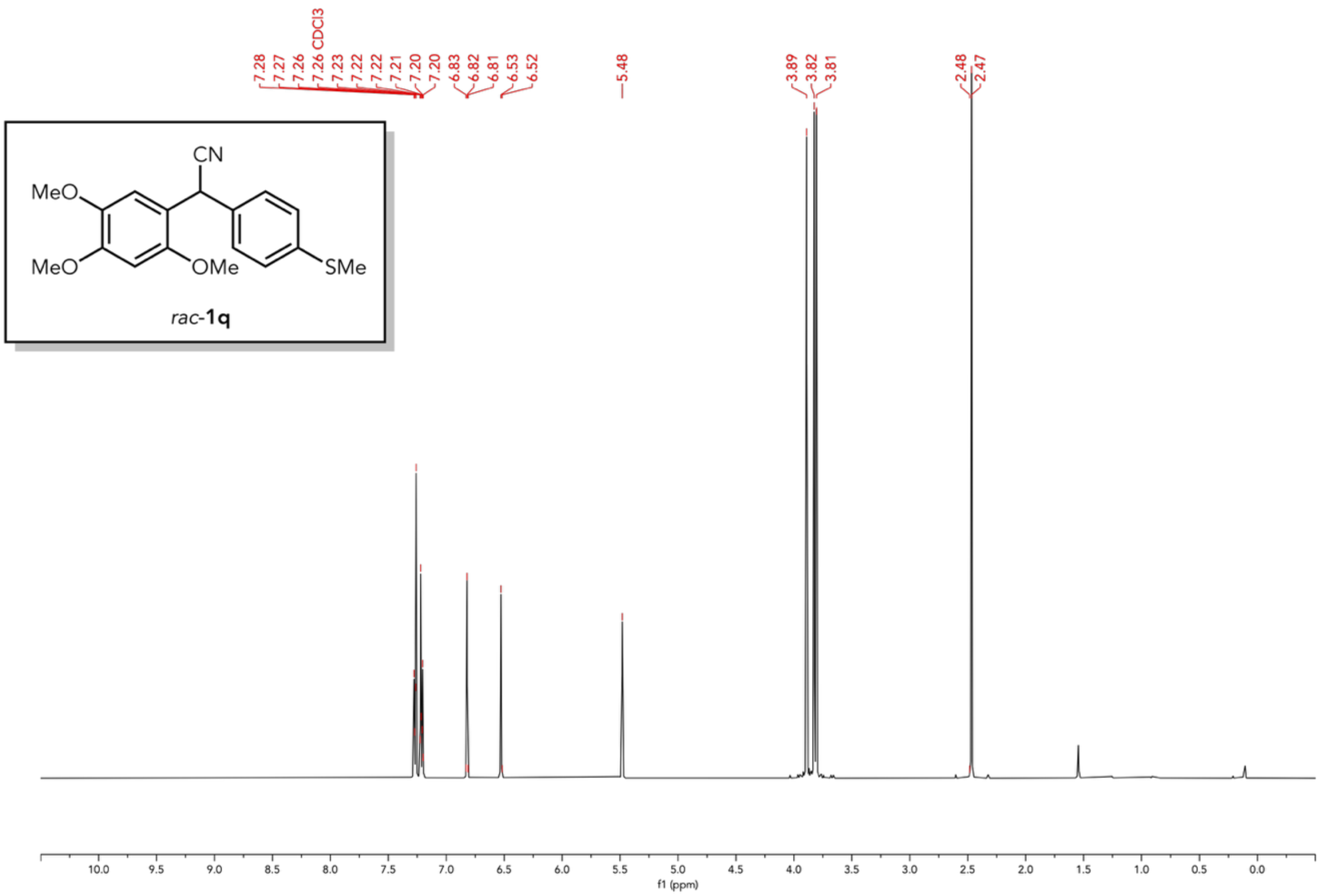

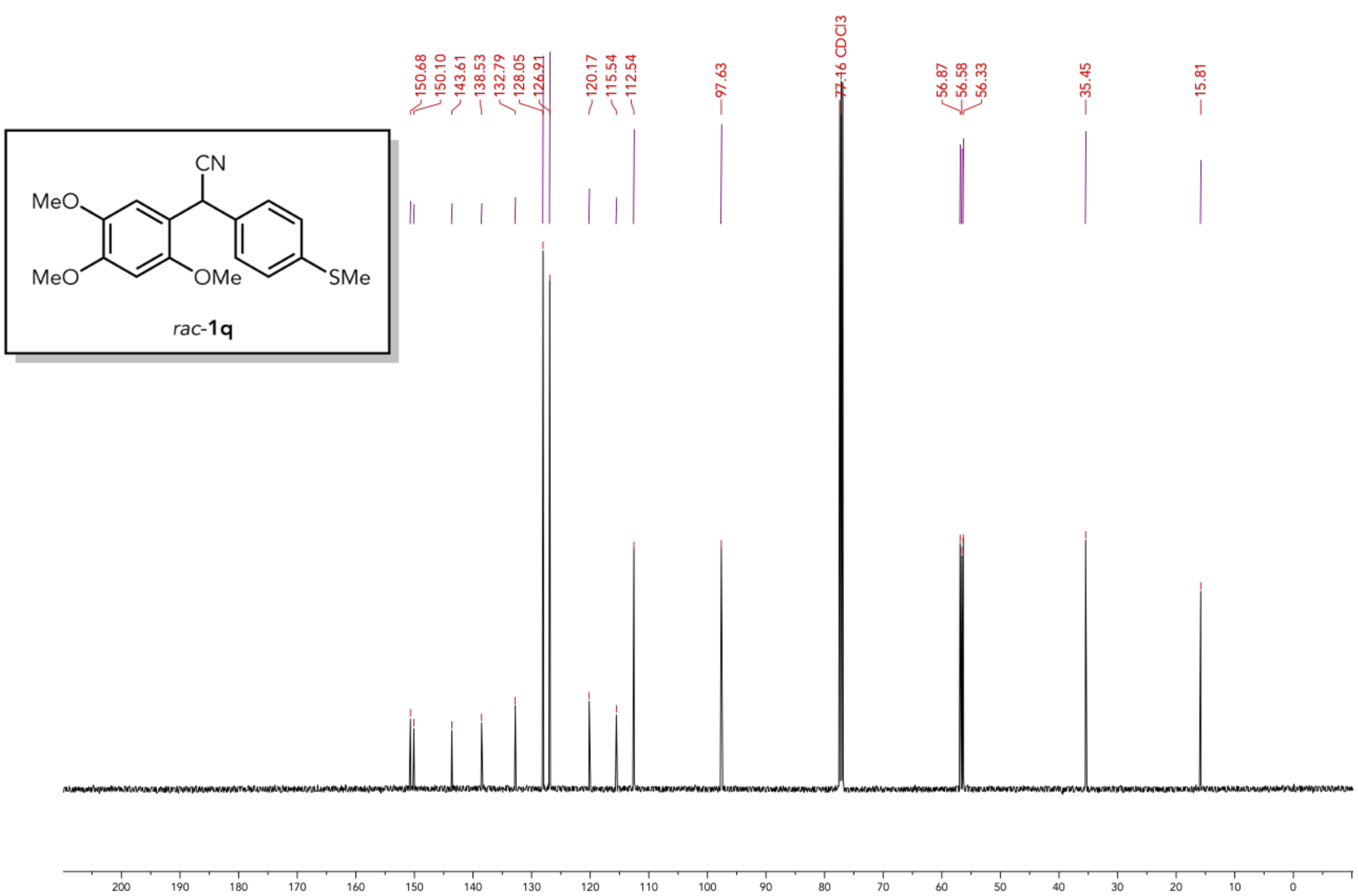

^{1}H NMR (500 MHz, $CDCl_3$) and ^{13}C NMR (126 MHz, $CDCl_3$) spectra for compound *rac*-**1r**

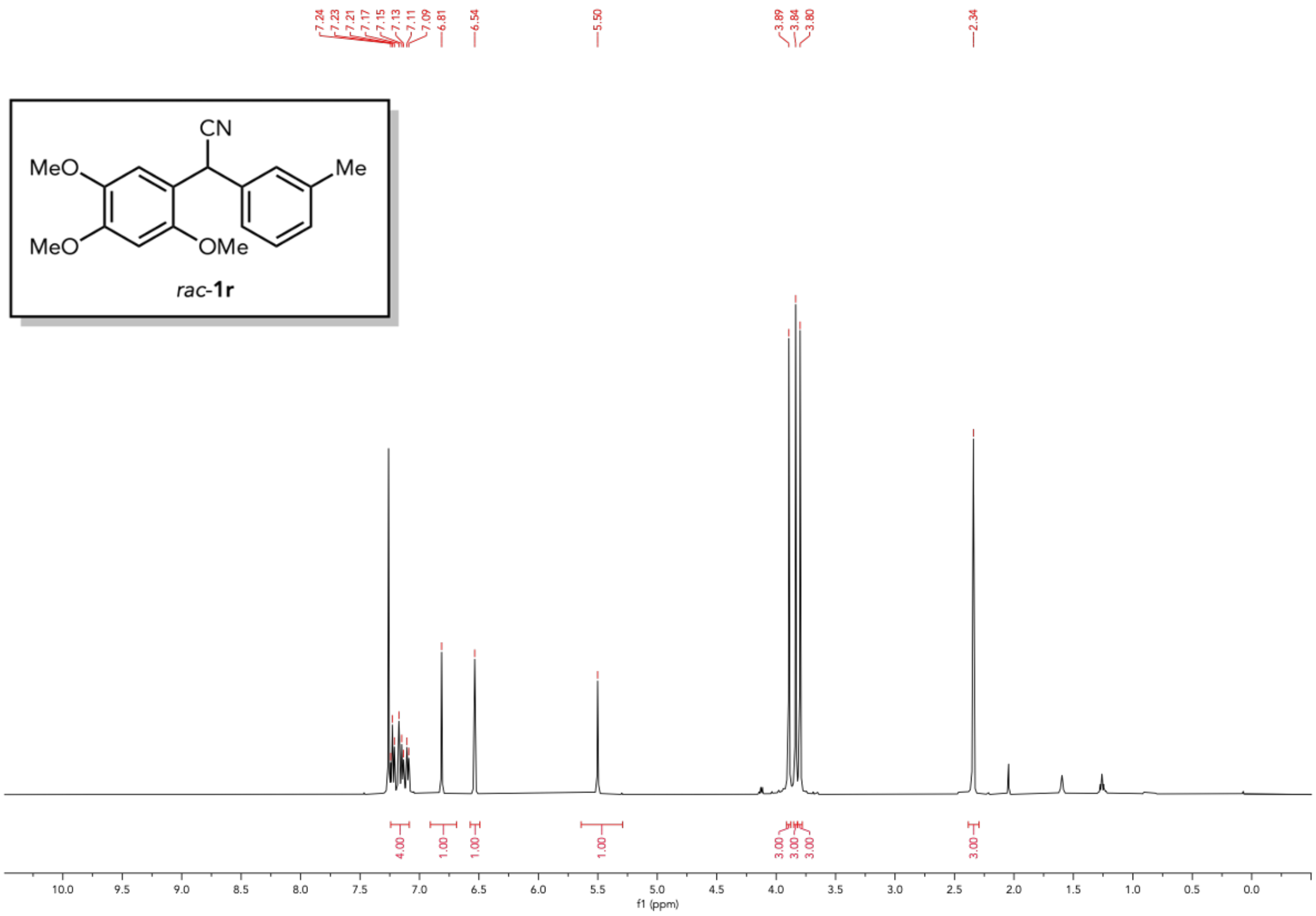

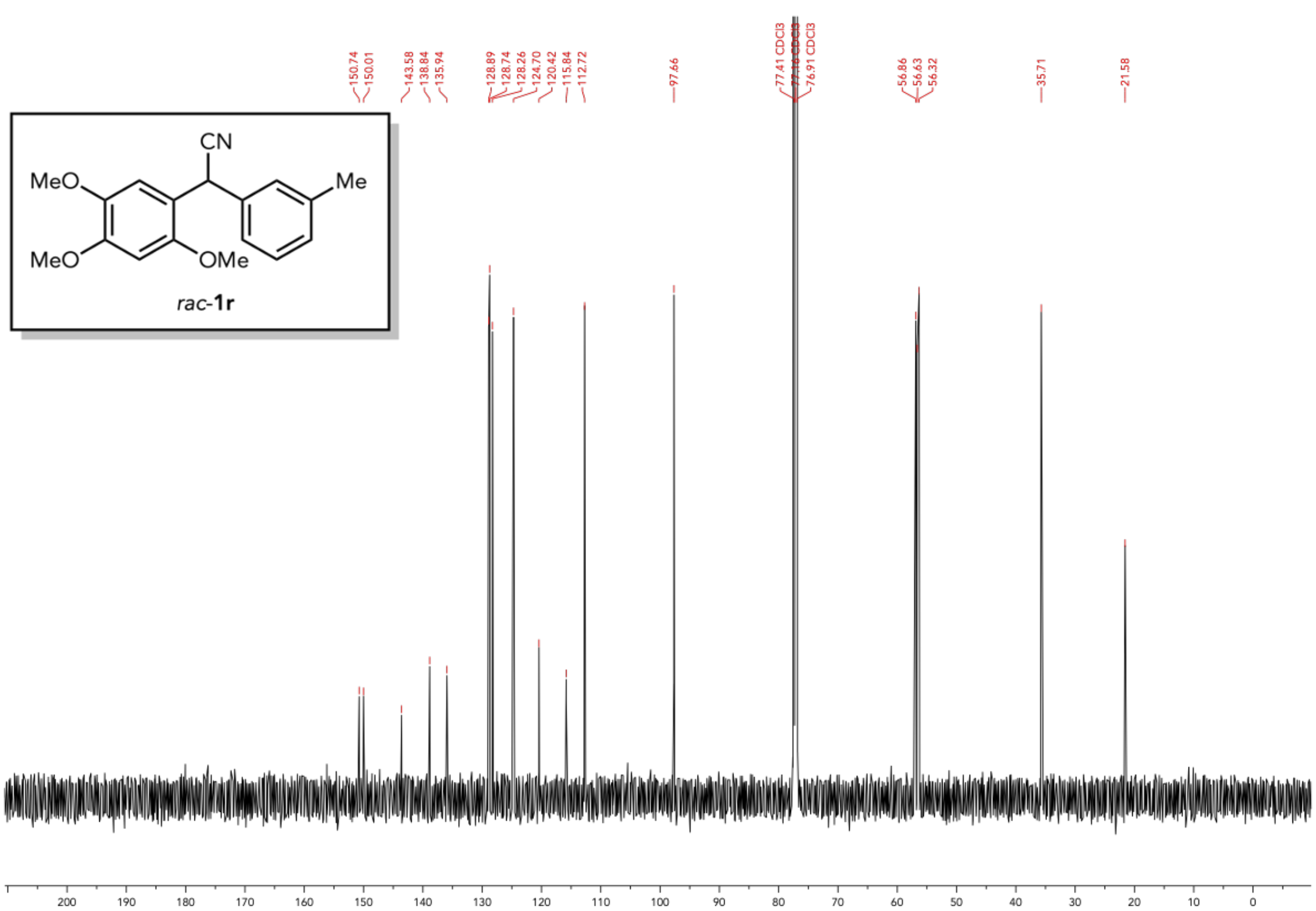

^{1}H NMR (500 MHz, $CDCl_3$) and ^{13}C NMR (126 MHz, $CDCl_3$) spectra for compound *rac*-**1s**

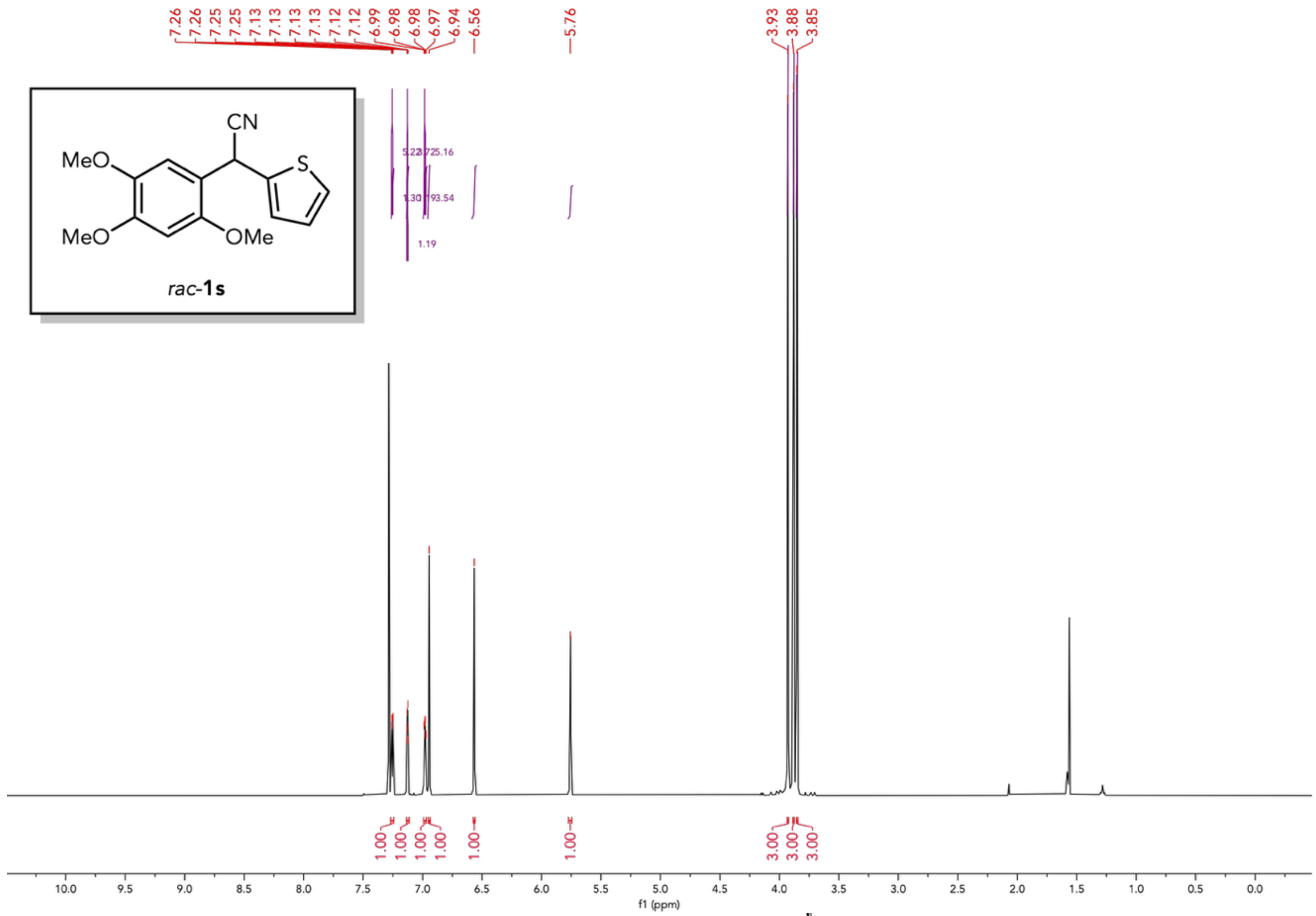

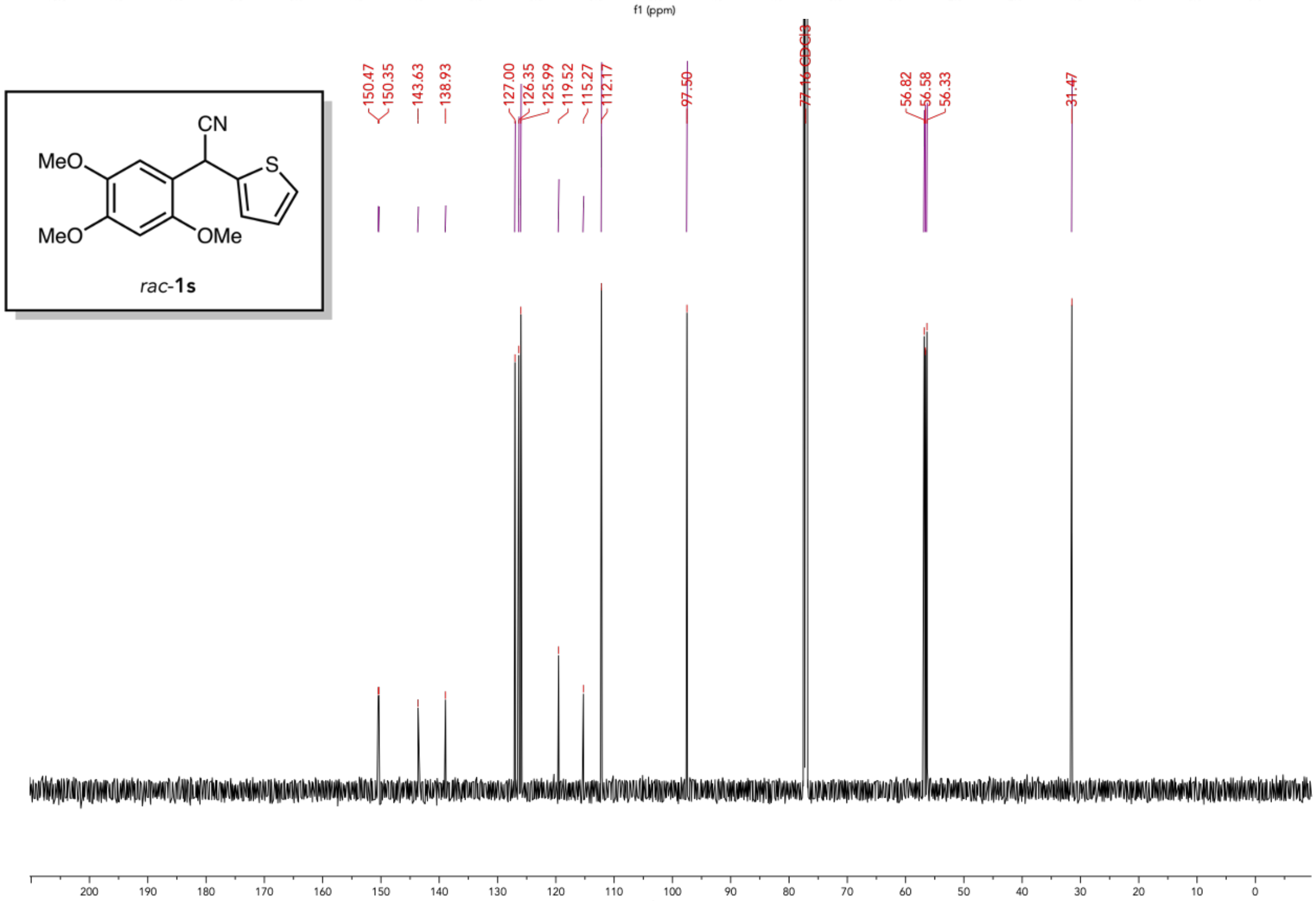

^{1}H NMR (500 MHz, $CDCl_3$) and ^{13}C NMR (126 MHz, $CDCl_3$) spectra for compound *rac*-**1t**

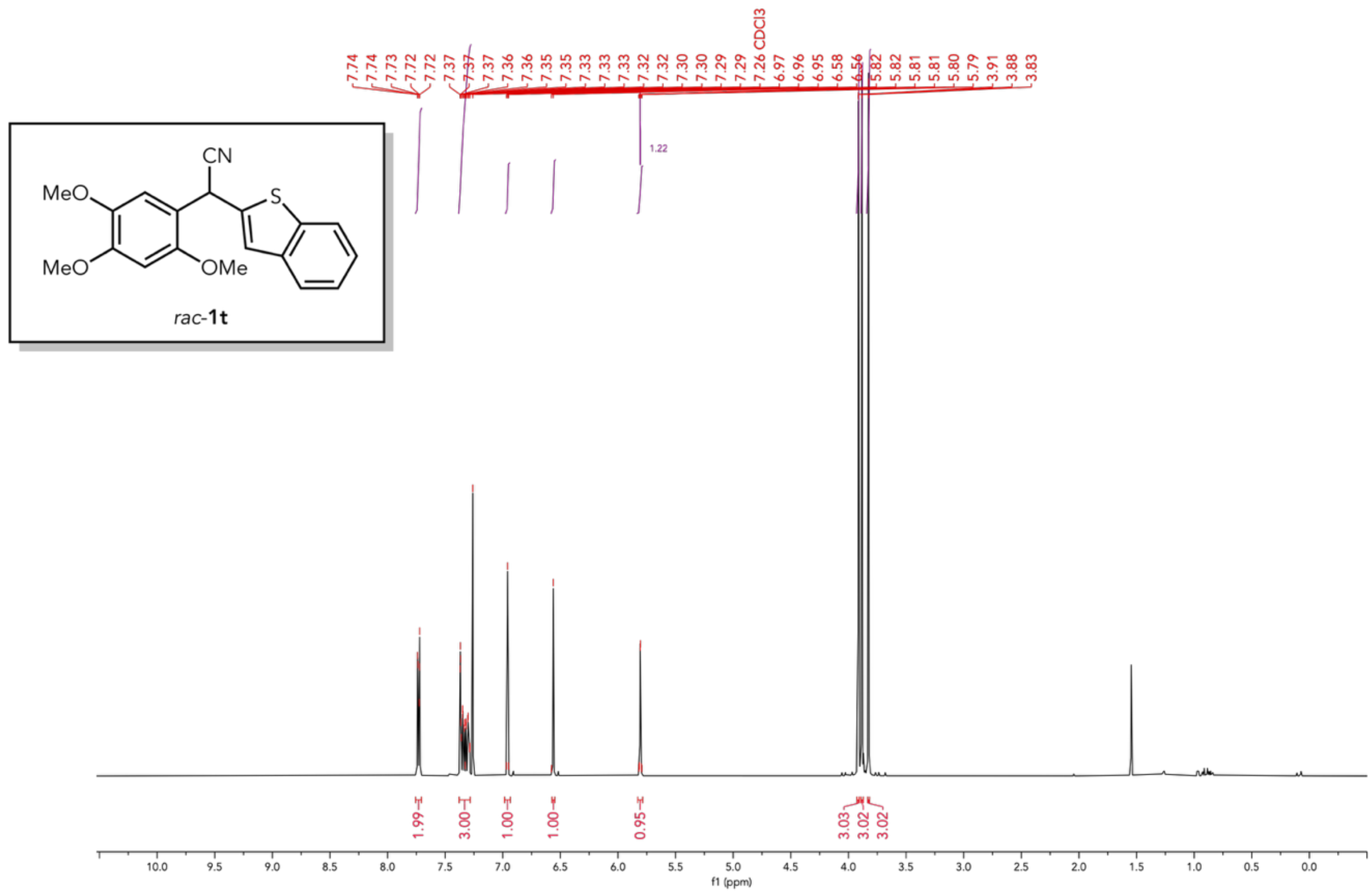

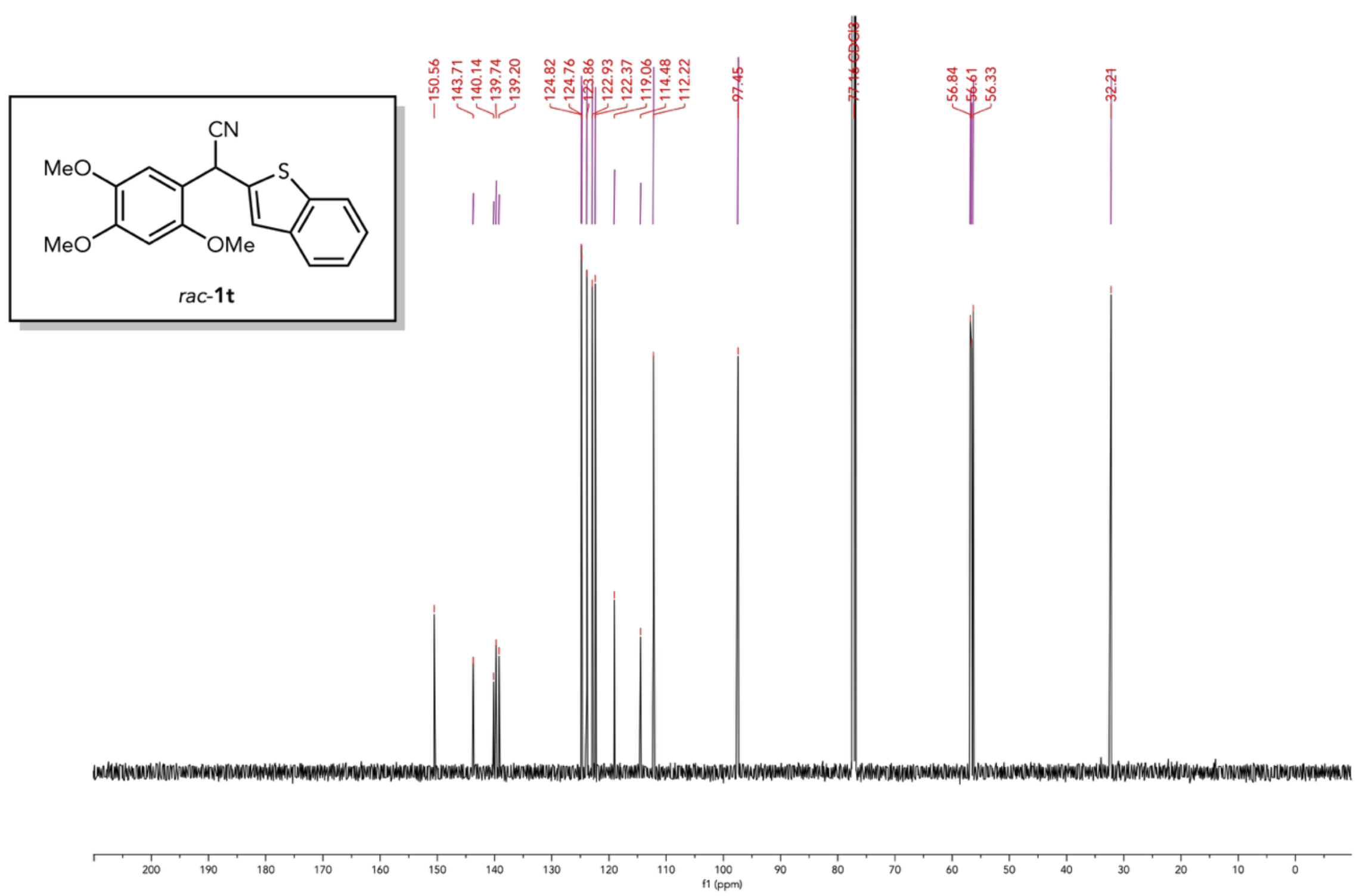

^{1}H NMR (500 MHz, $CDCl_3$) and ^{13}C NMR (126 MHz, $CDCl_3$) spectra for compound *rac*-**1u**

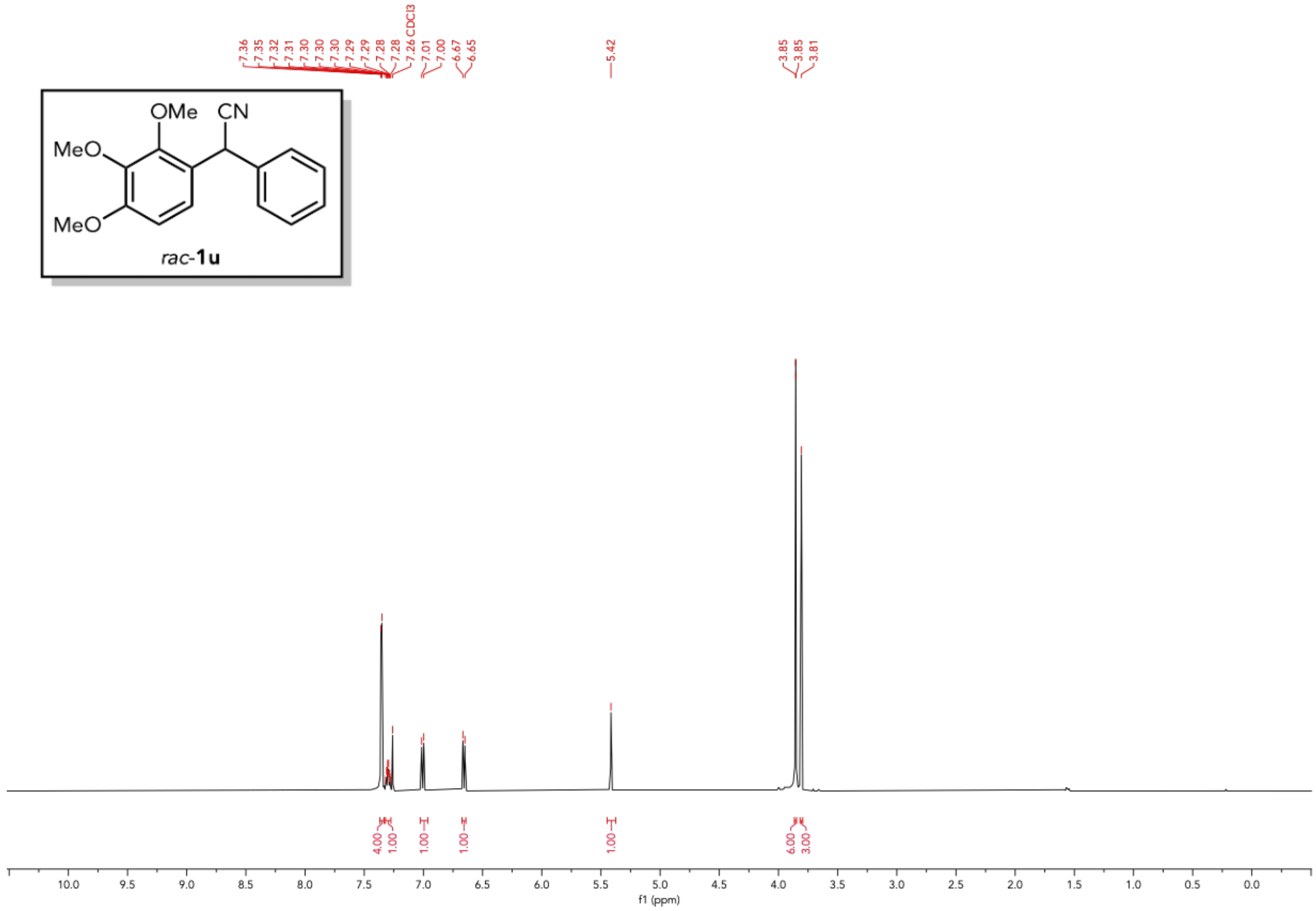

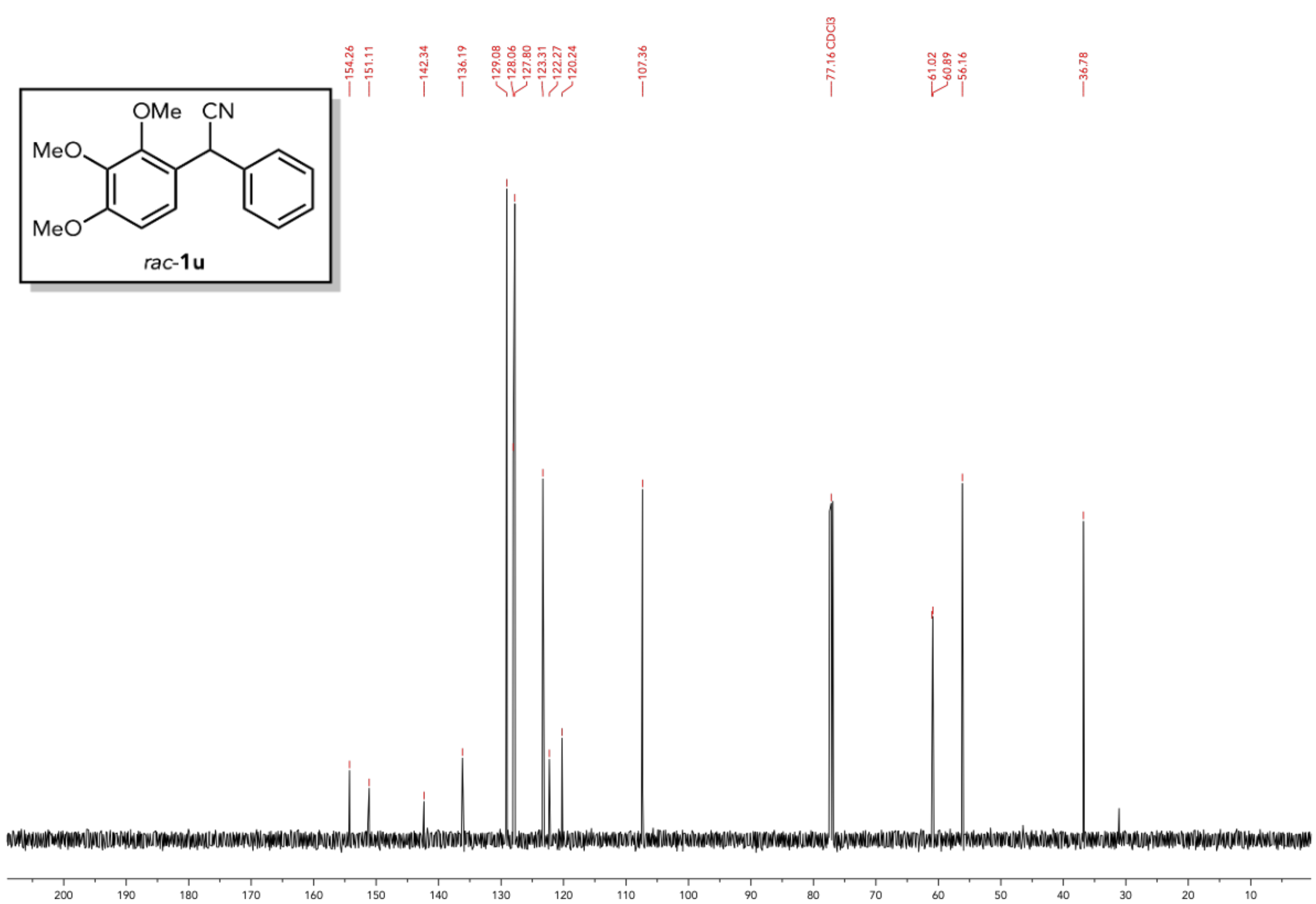

^{1}H NMR (500 MHz, $CDCl_3$) and ^{13}C NMR (126 MHz, $CDCl_3$) spectra for compound *rac*-**1v**

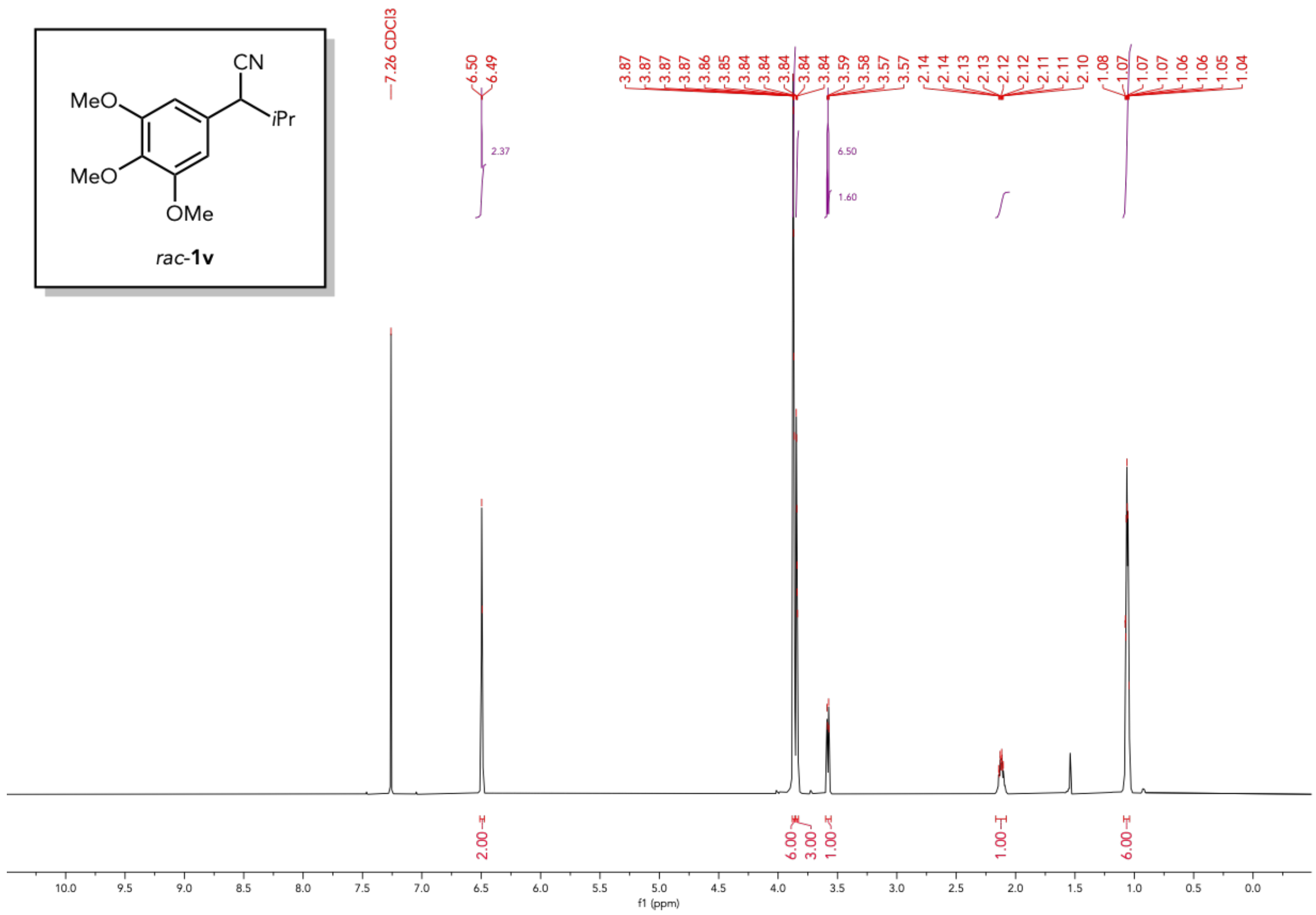

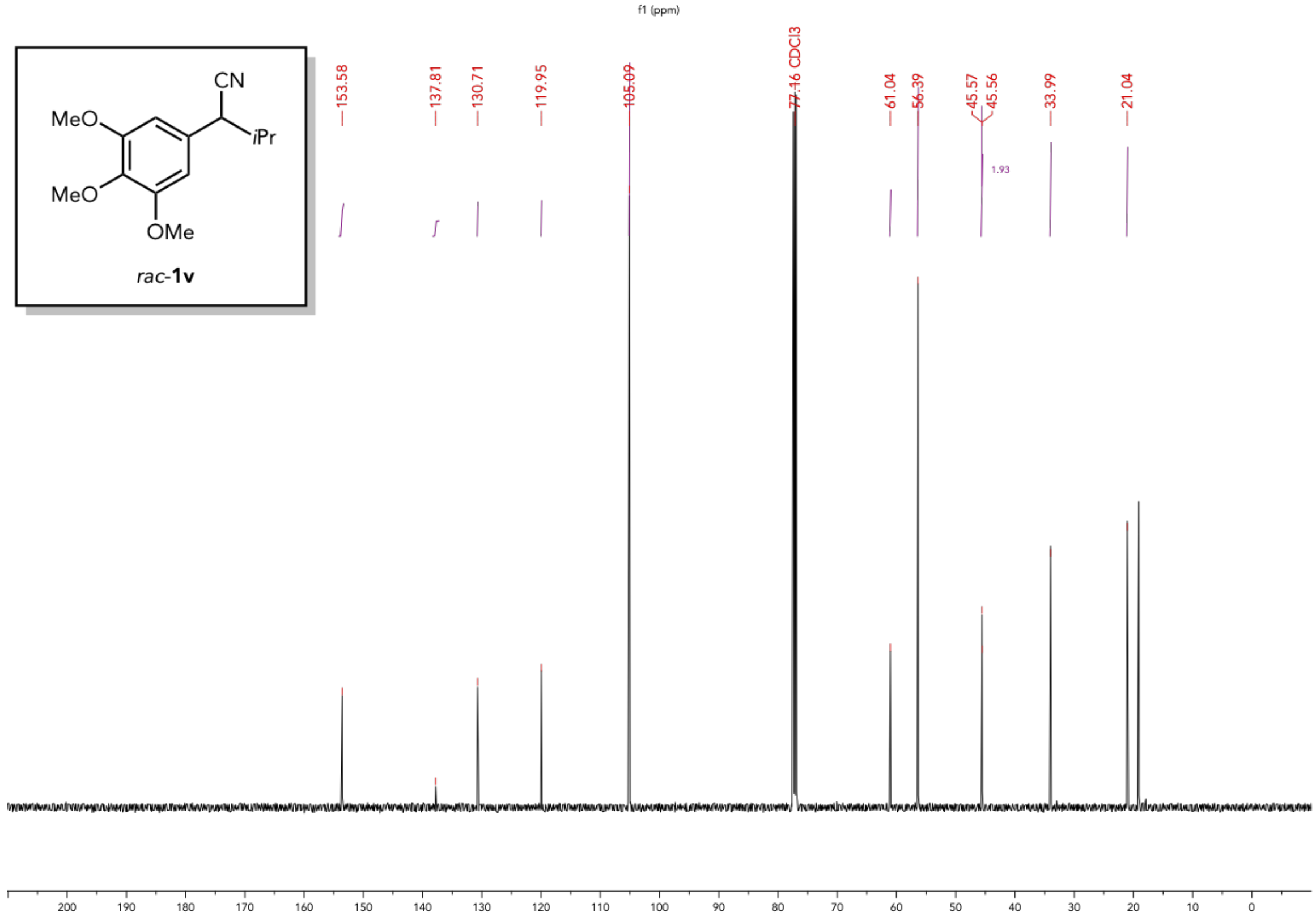

^{1}H NMR (500 MHz, $CDCl_3$) and ^{13}C NMR (126 MHz, $CDCl_3$) spectra for compound *rac*-**1w**

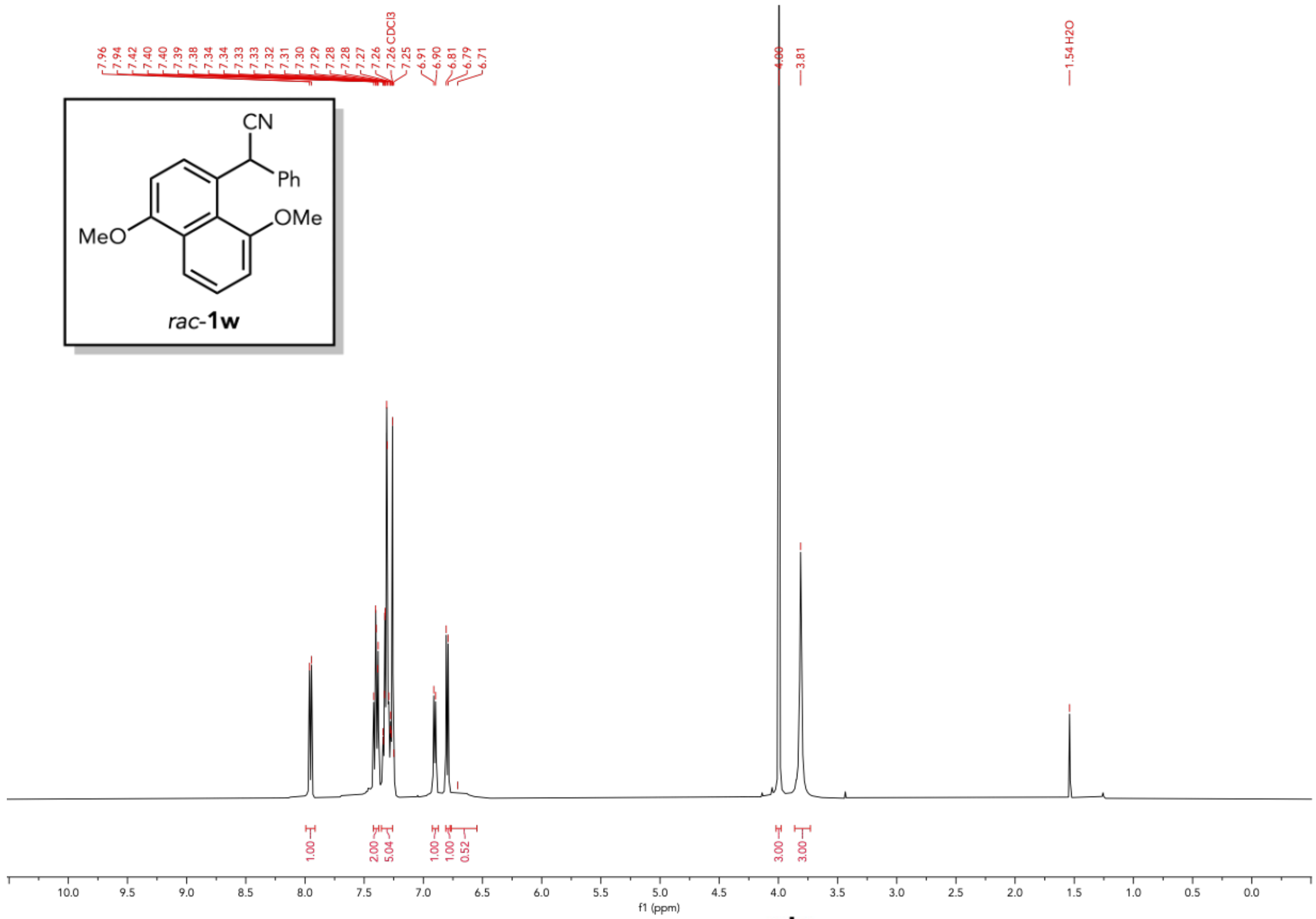

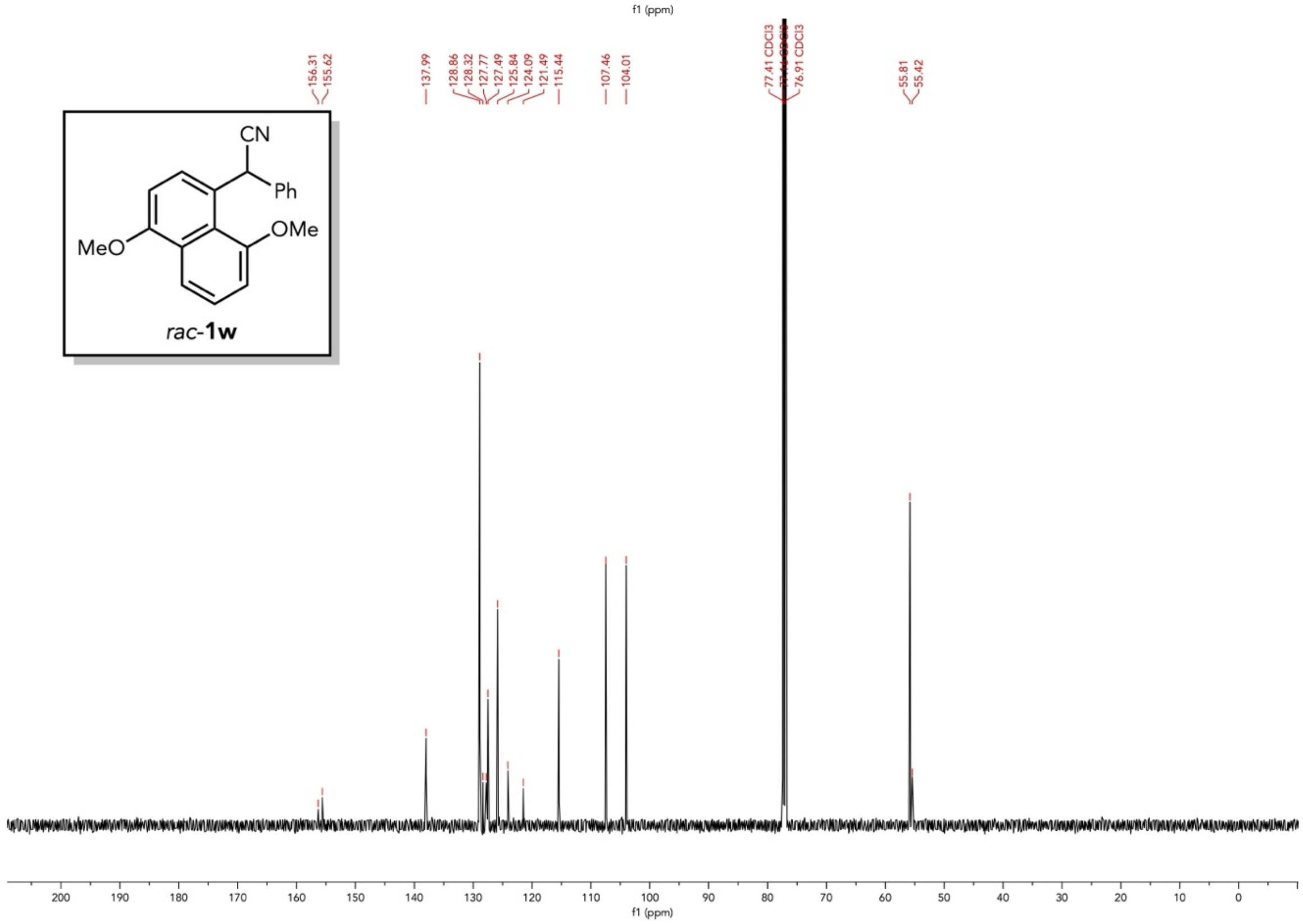

^{13}C NMR (126 MHz, DMSO-d_6) spectra for compound *rac*-**1w**

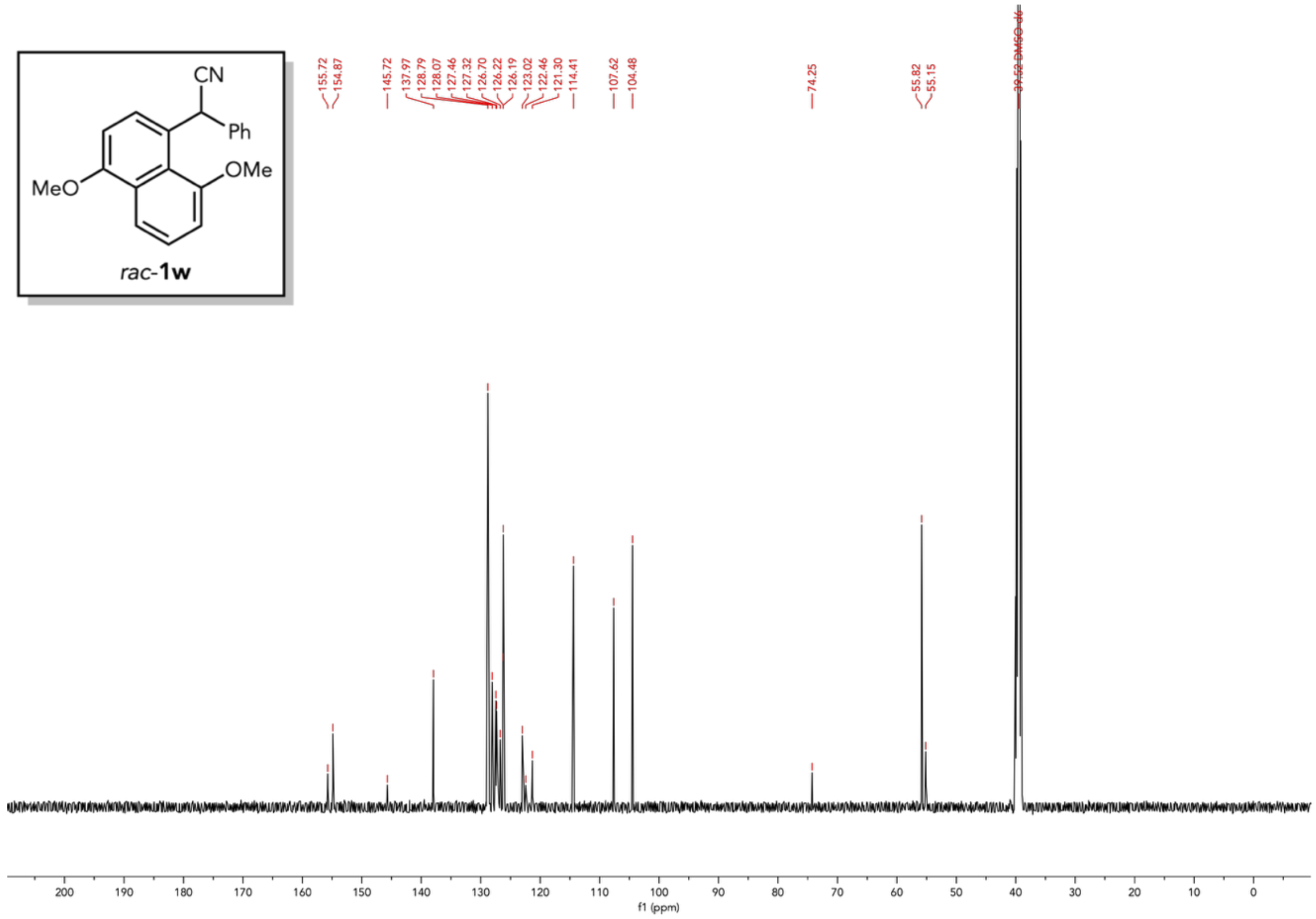

^{1}H NMR (500 MHz, $CDCl_3$) and ^{13}C NMR (126 MHz, $CDCl_3$) spectra for compound *rac*-**1x**

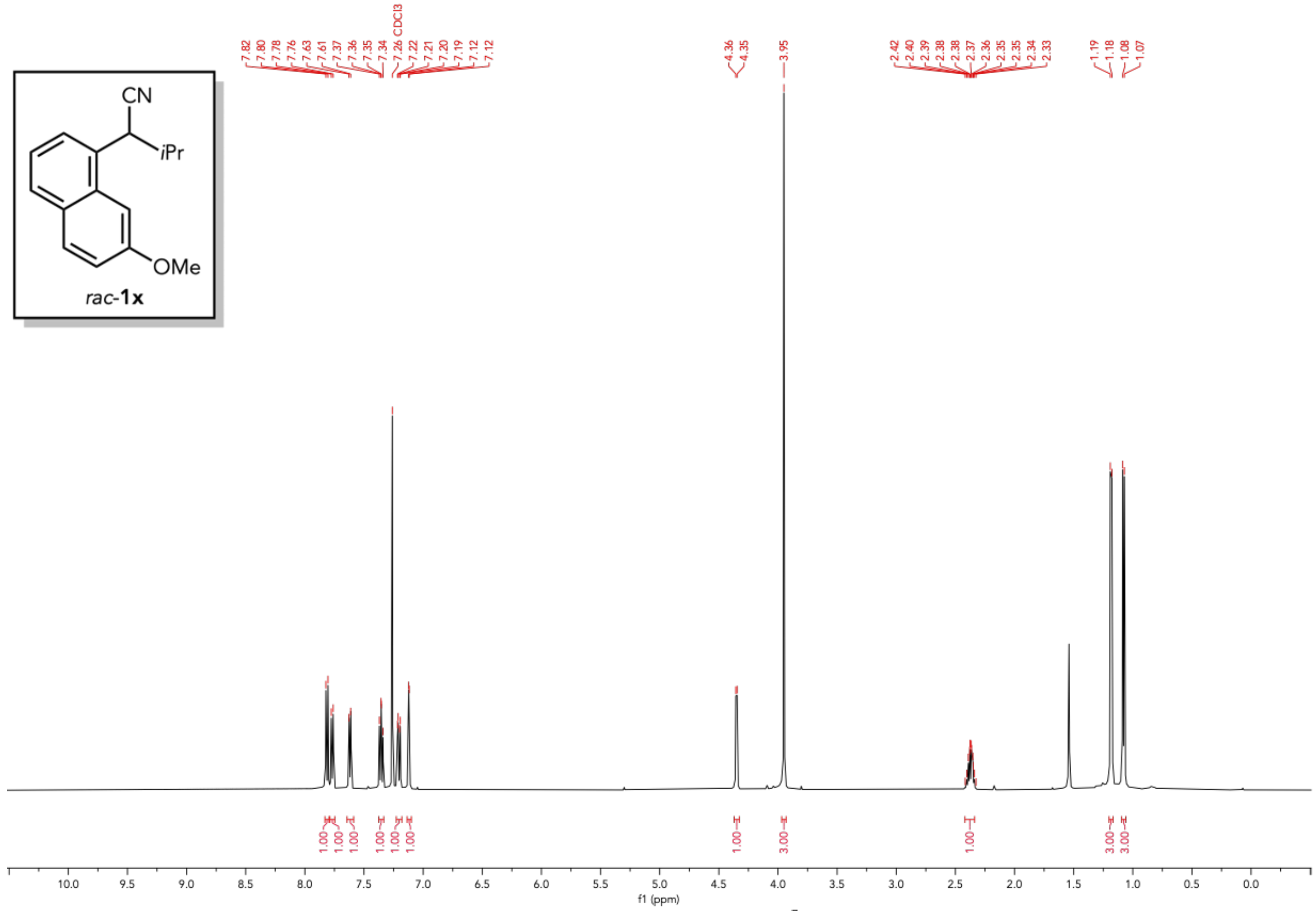

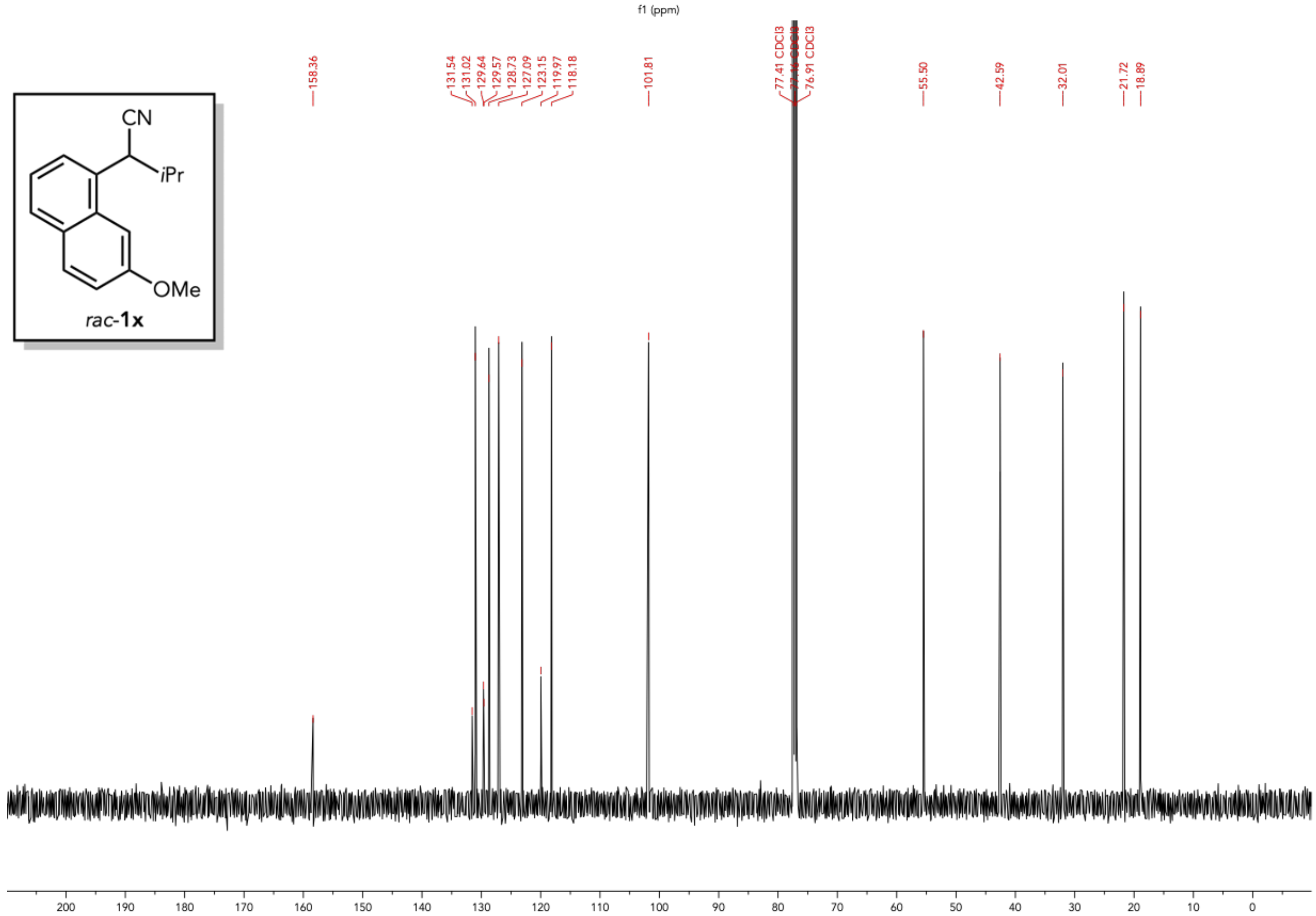

^{1}H NMR (500 MHz, $CDCl_3$) and ^{13}C NMR (126 MHz, $CDCl_3$) spectra for compound *rac*-**1y**

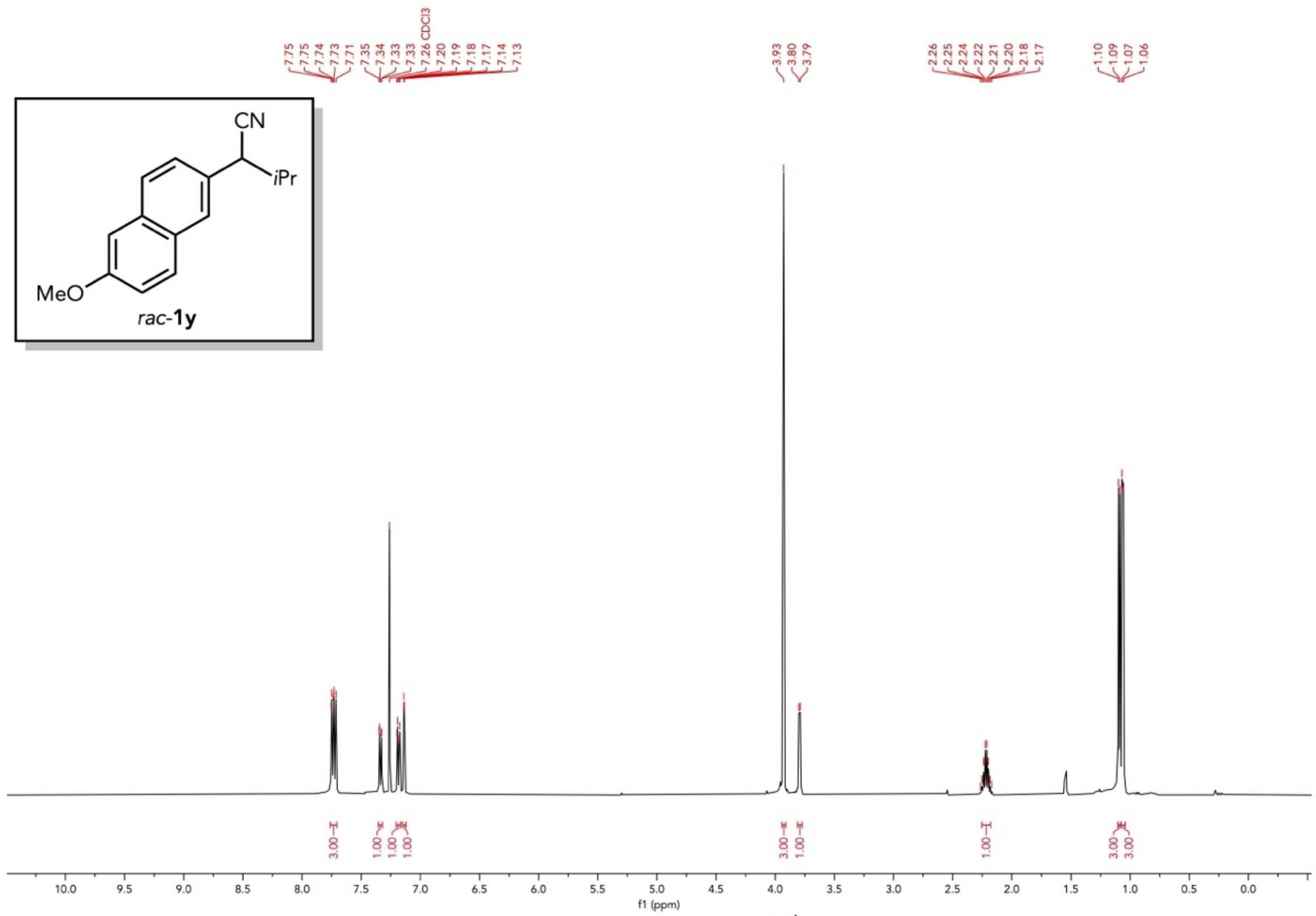

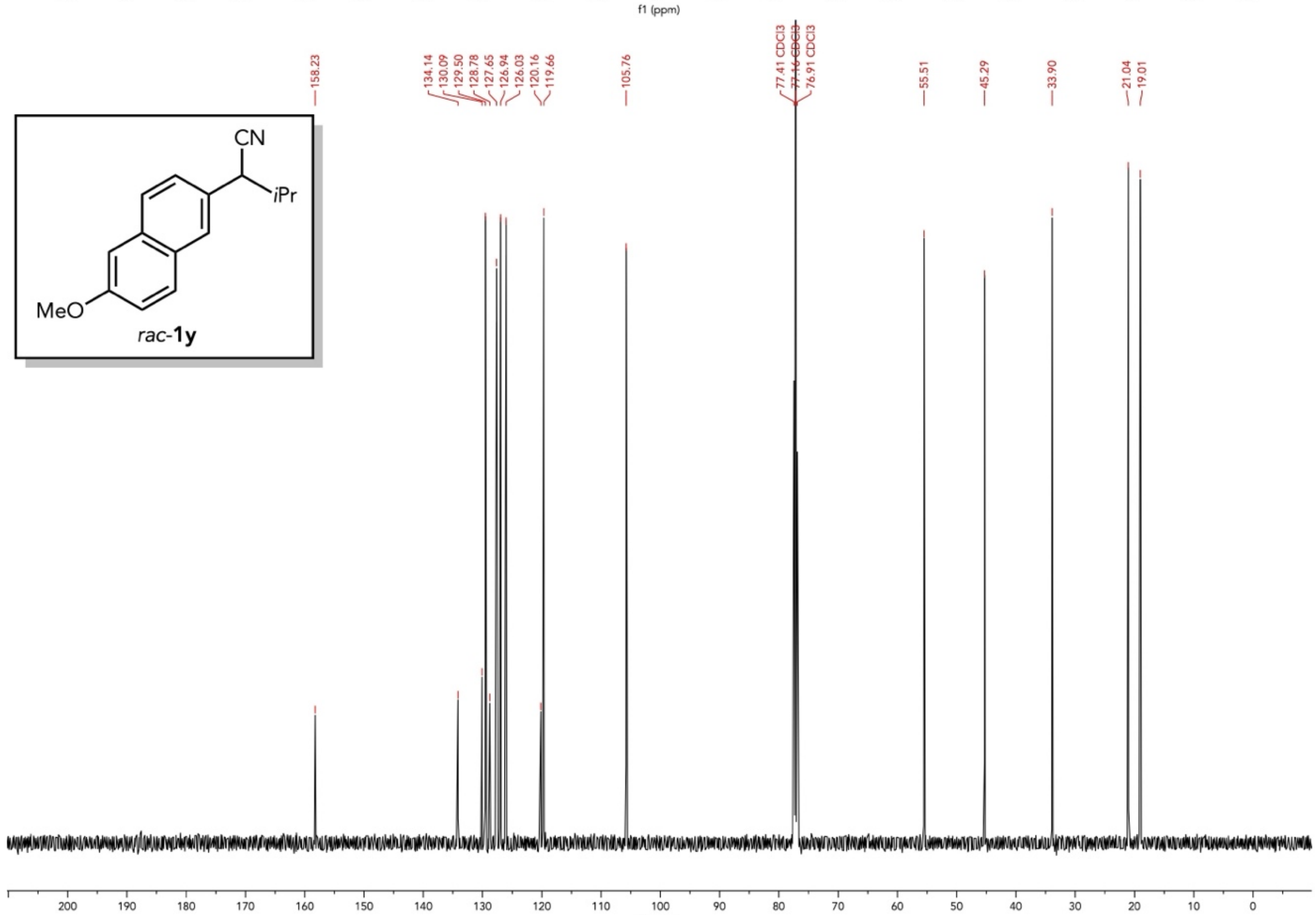

^{1}H NMR (500 MHz, $CDCl_3$) and ^{13}C NMR (126 MHz, $CDCl_3$) spectra for compound *rac*-**1z**

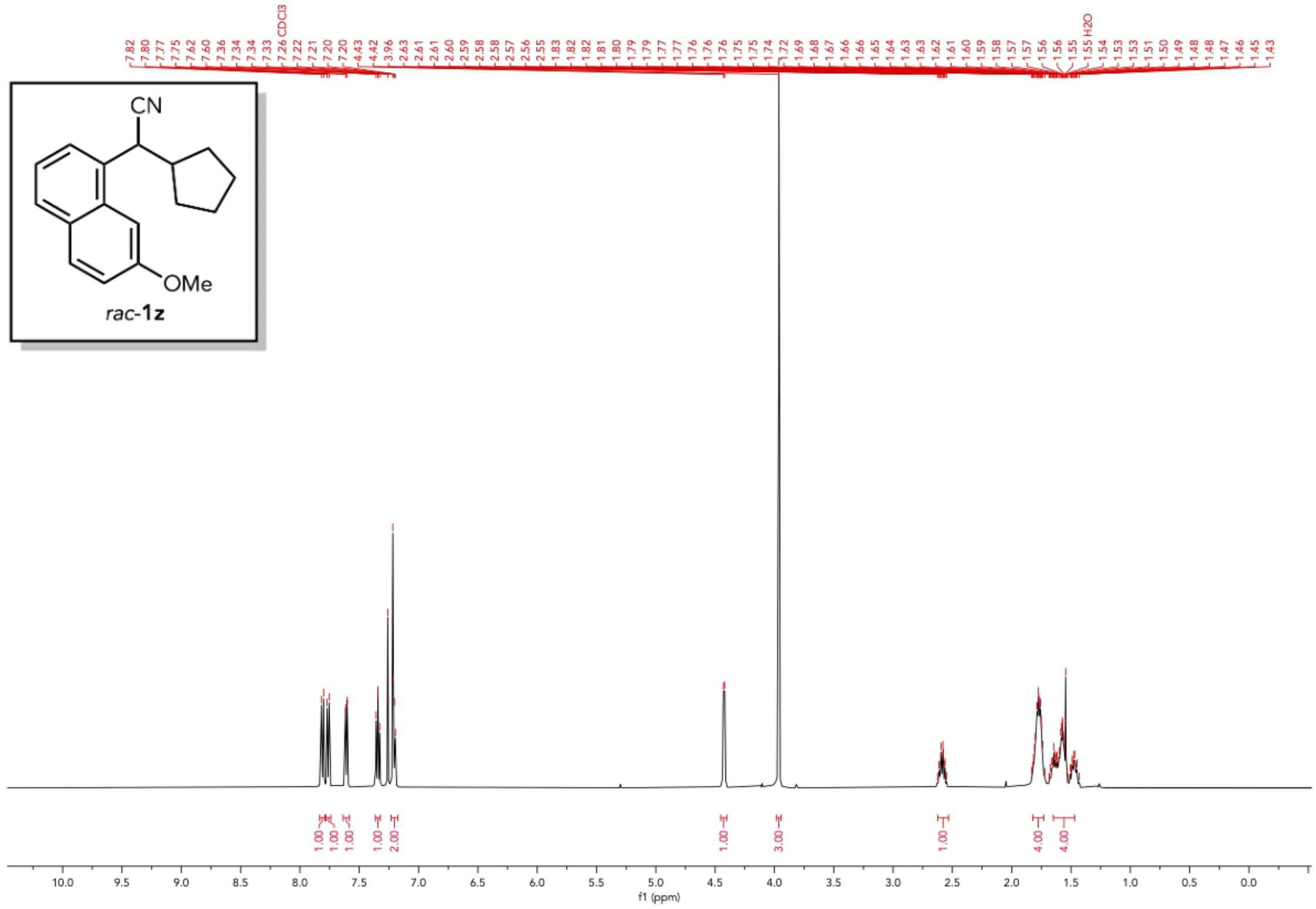

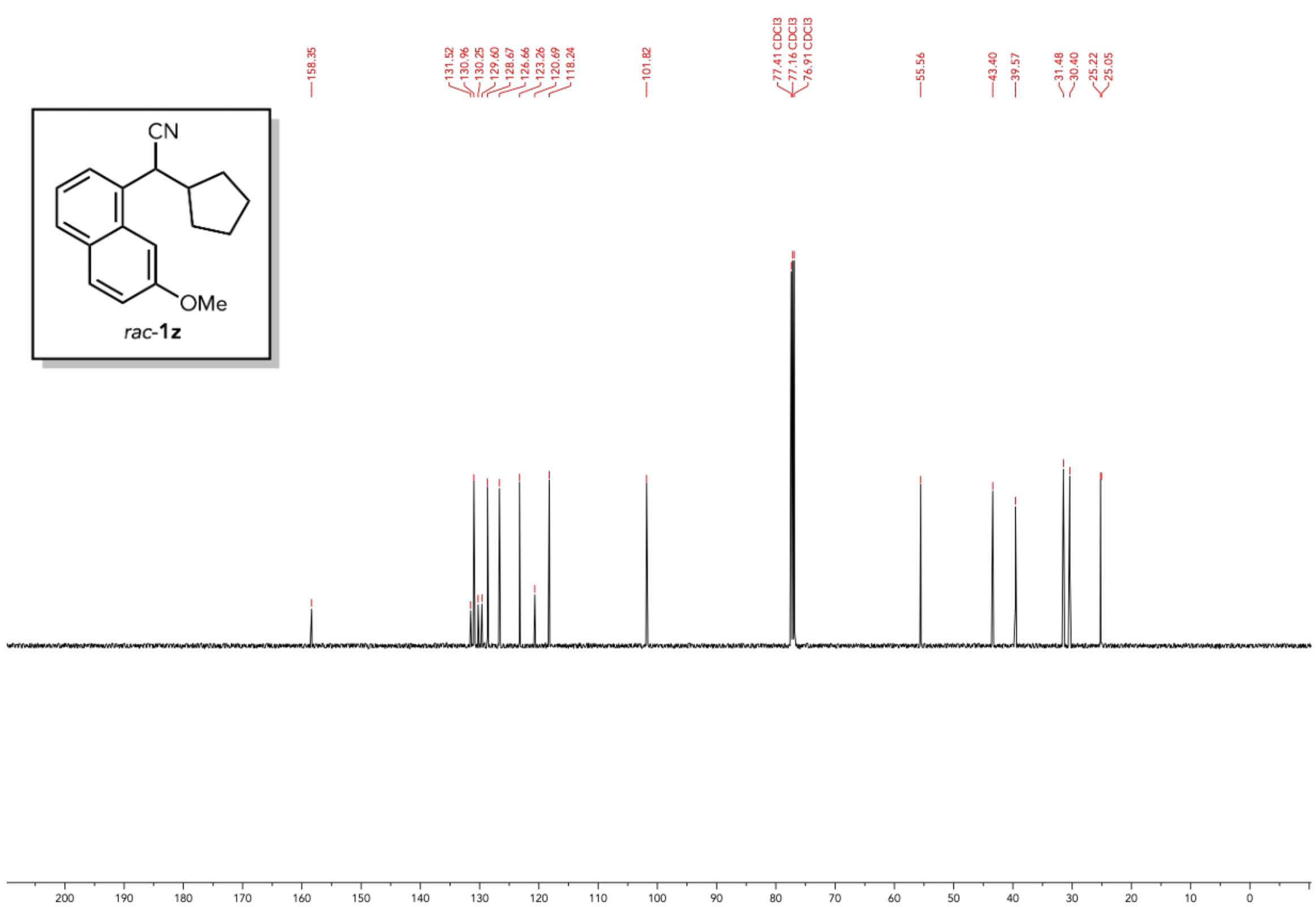

^{1}H NMR (500 MHz, $CDCl_3$) and ^{13}C NMR (126 MHz, $CDCl_3$) spectra for compound *rac*-**1aa**

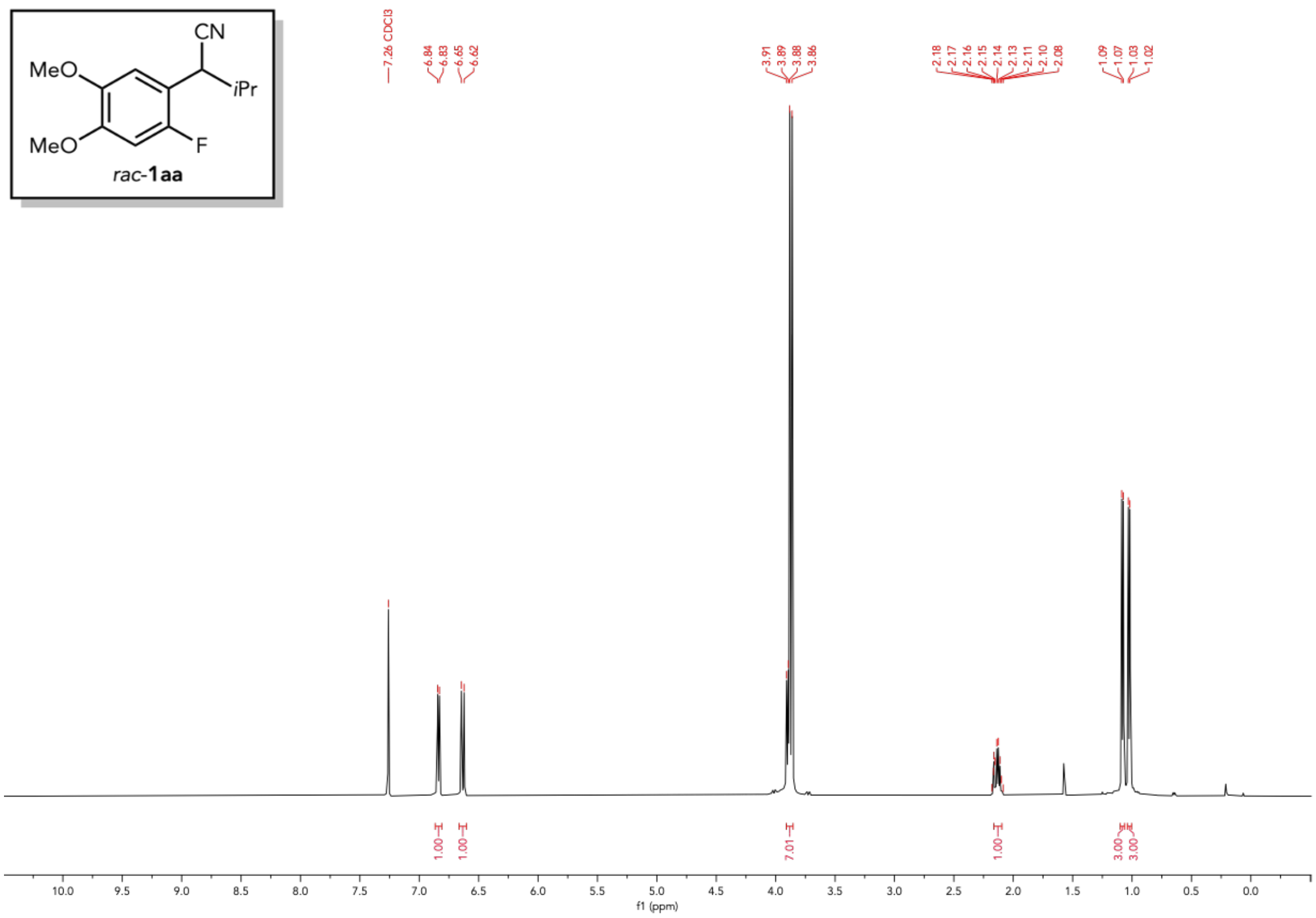

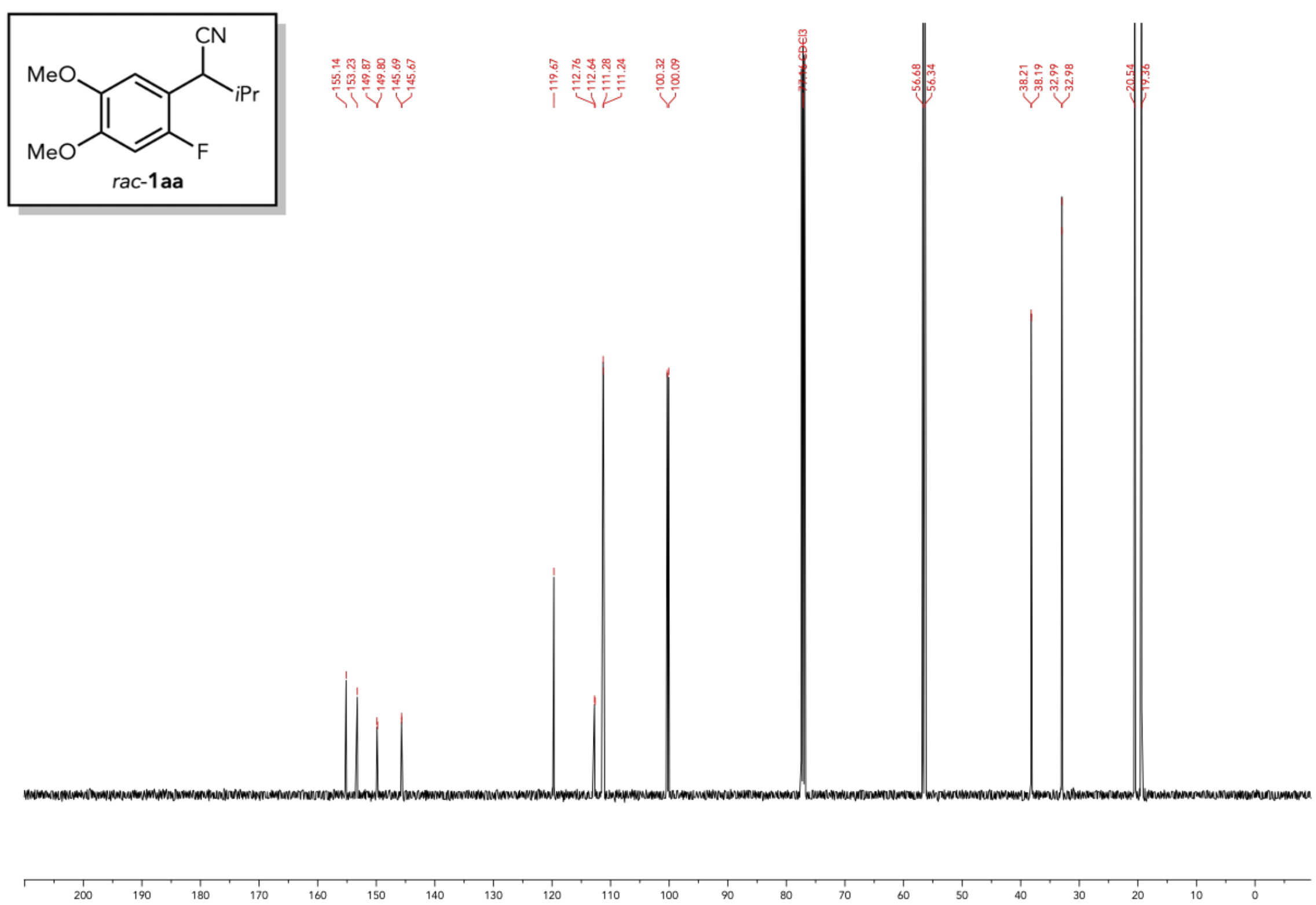

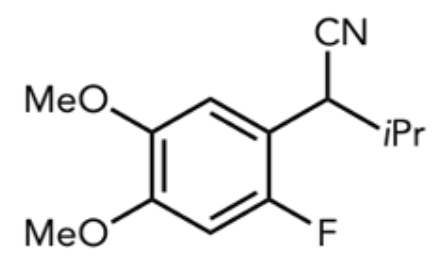

rac-**1aa**

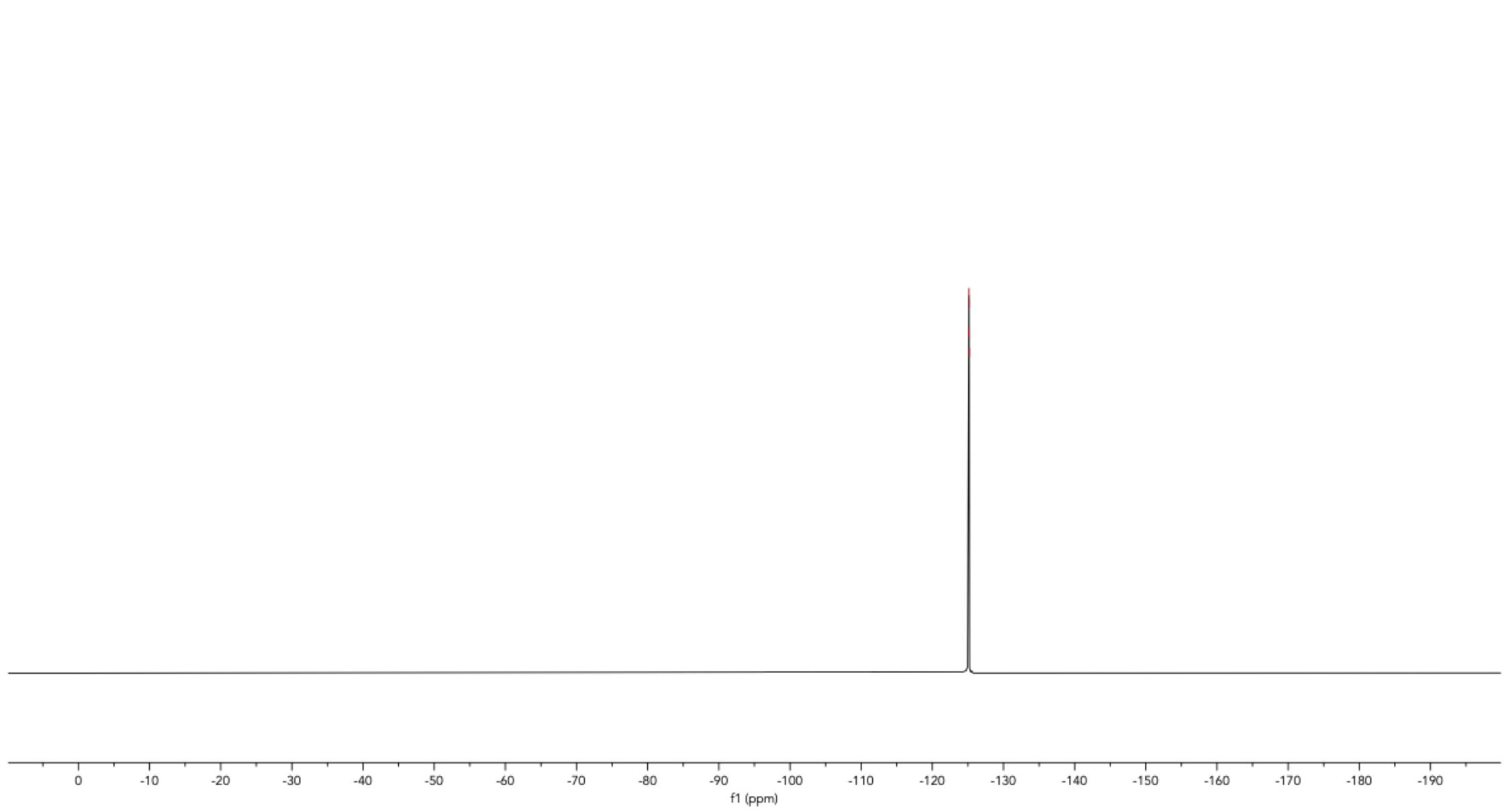

-125.14
-125.15
-125.16
-125.18
0 -10 -20 -30 -40 -50 -60 -70 -80 -90 -100 -110 -120 -130 -140 -150 -160 -170 -180 -190
f1 (ppm)

^{1}H NMR (500 MHz, $CDCl_3$) and ^{13}C NMR (126 MHz, $CDCl_3$) spectra for compound *rac*-**1ab**

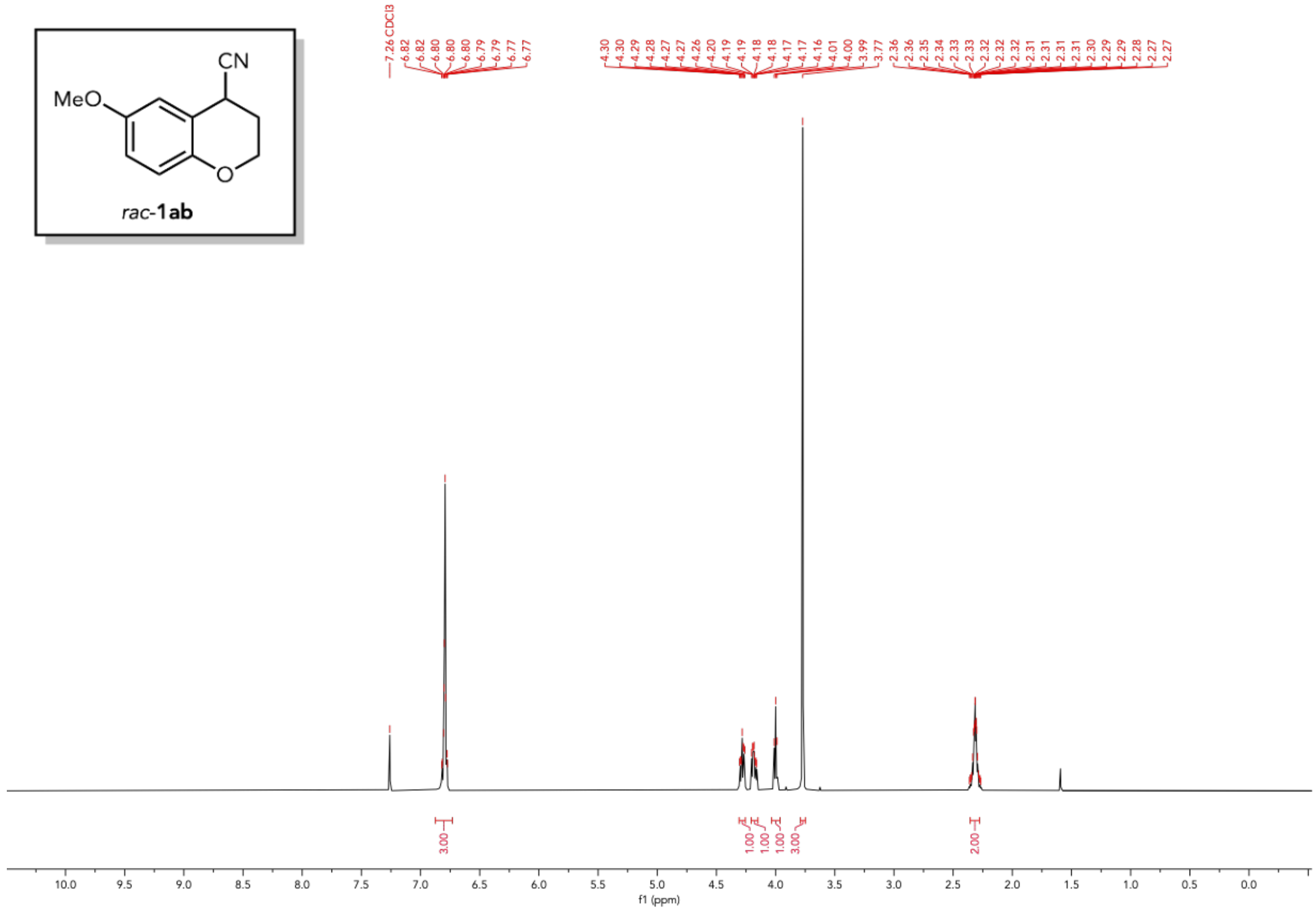

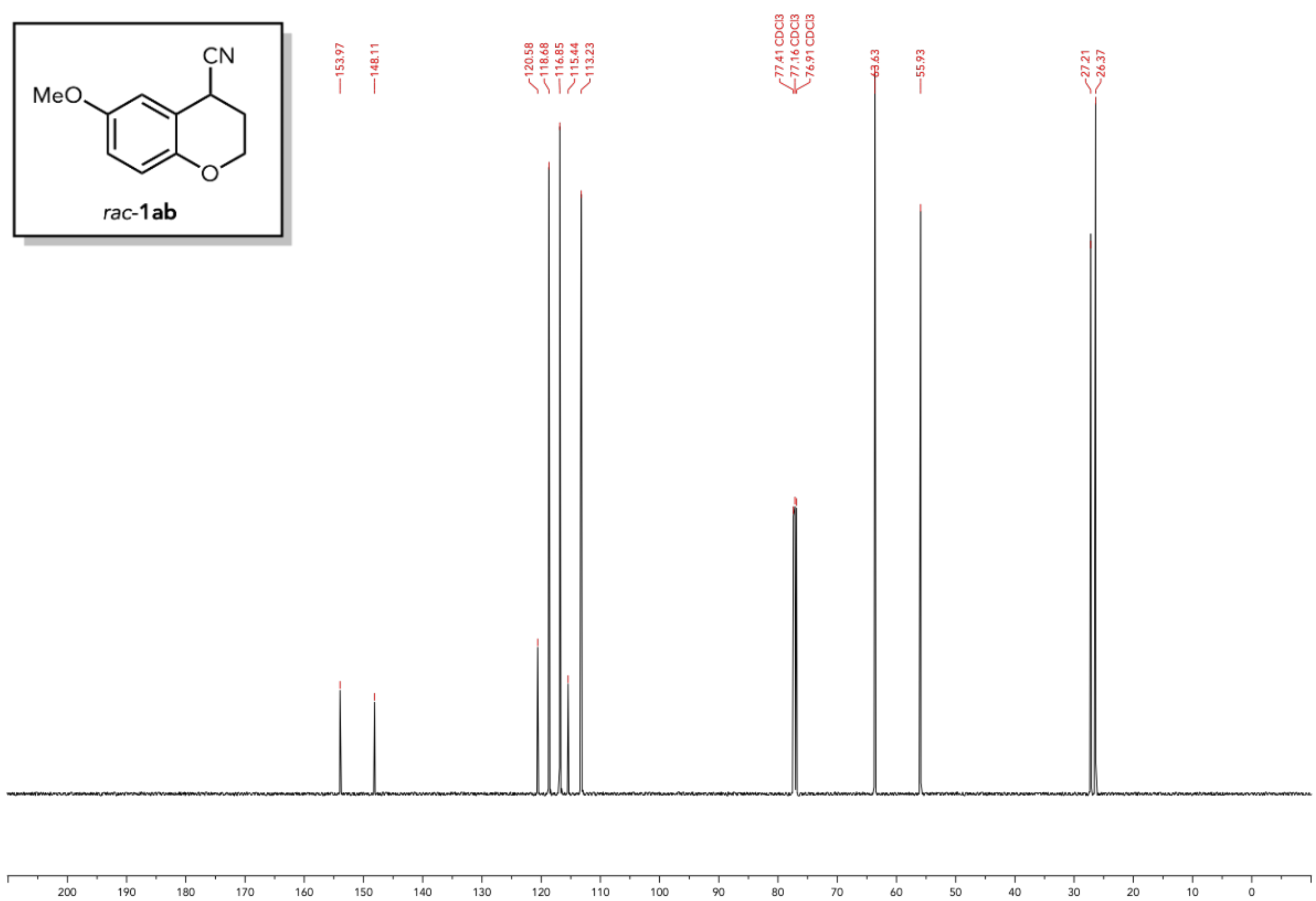

^{1}H NMR (500 MHz, $CDCl_3$) and ^{13}C NMR (126 MHz, $CDCl_3$) spectra for compound *rac*-**1ac**

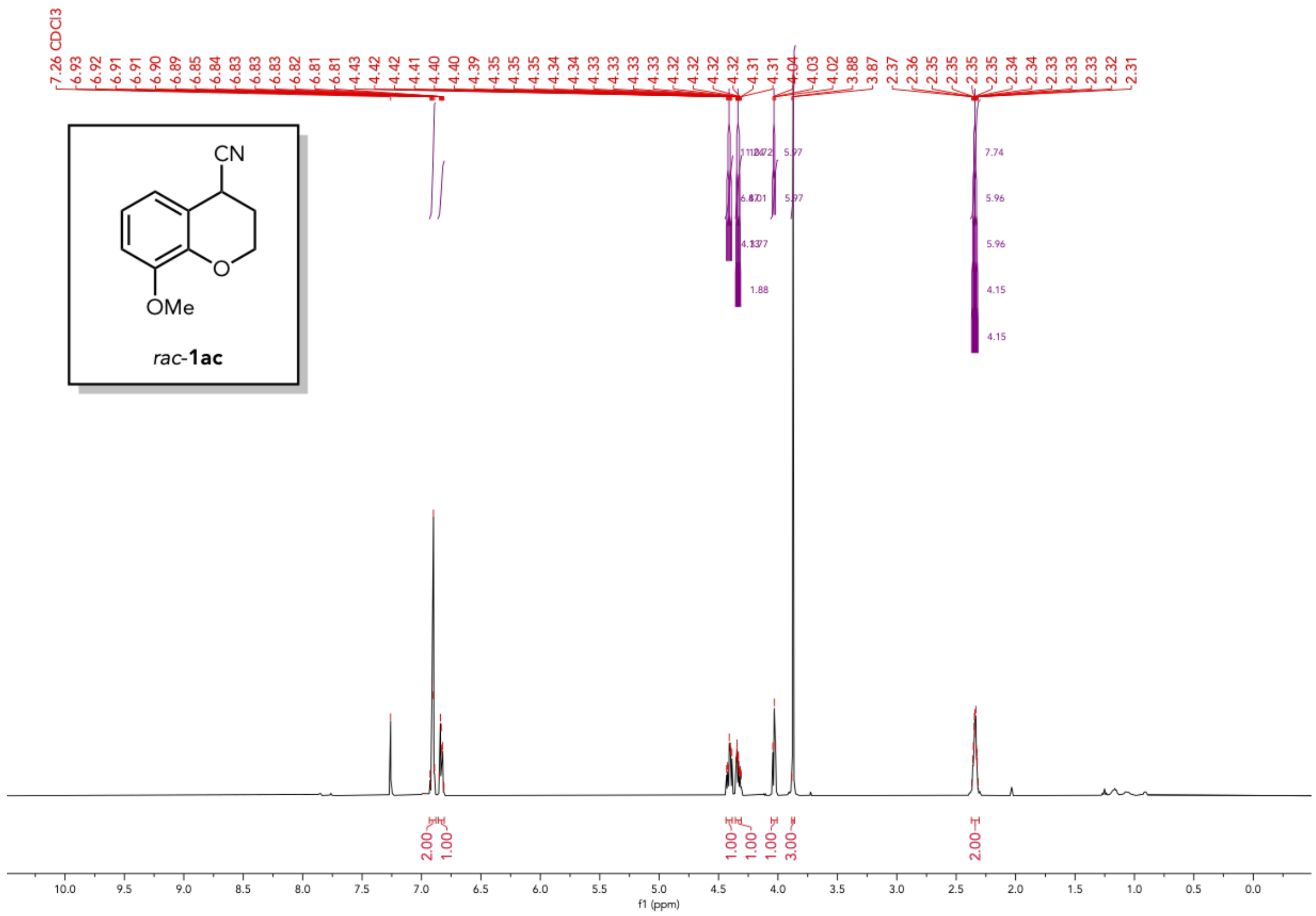

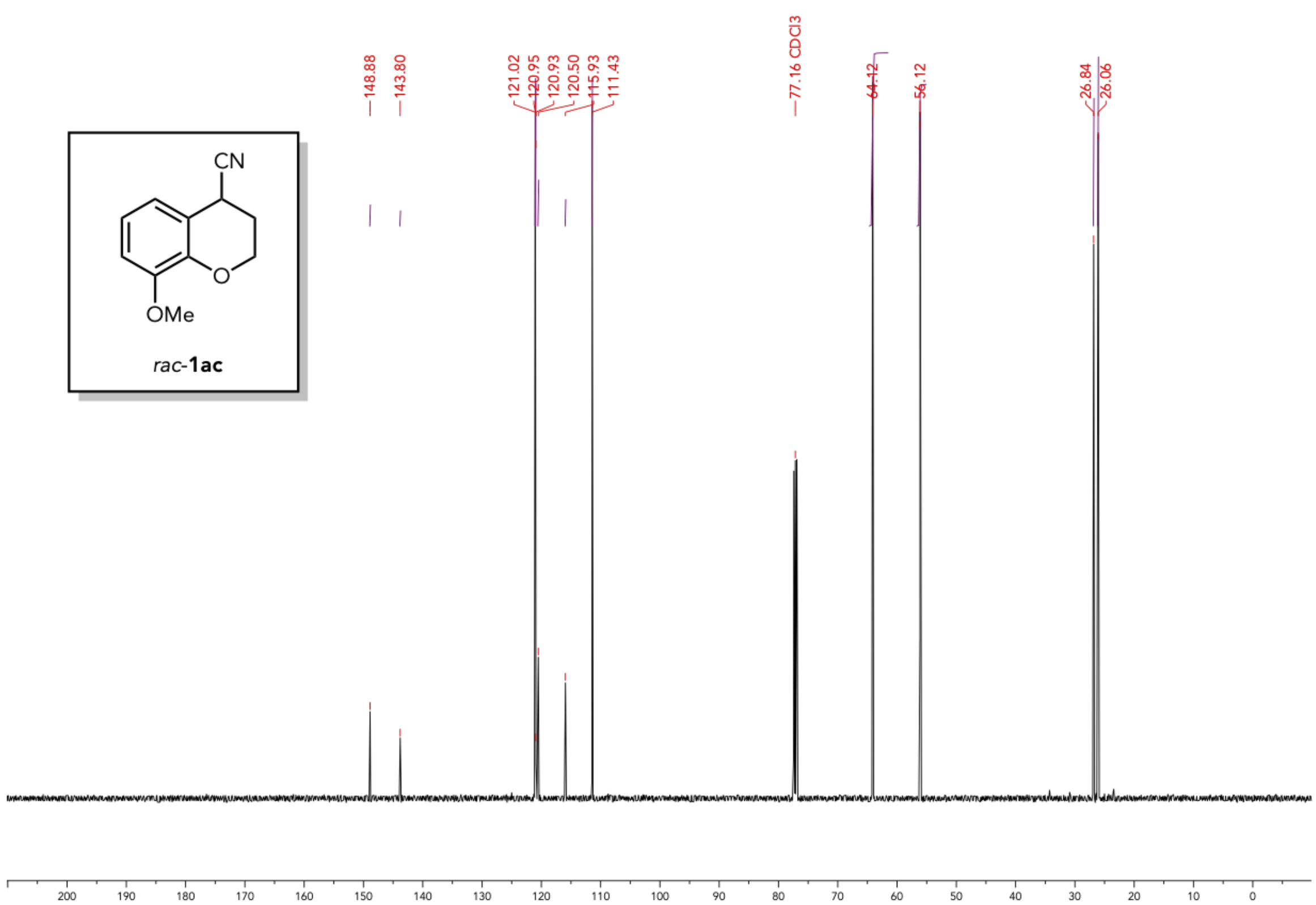

^{1}H NMR (500 MHz, $CDCl_3$) and ^{13}C NMR (126 MHz, $CDCl_3$) spectra for compound *rac*-**1ad**

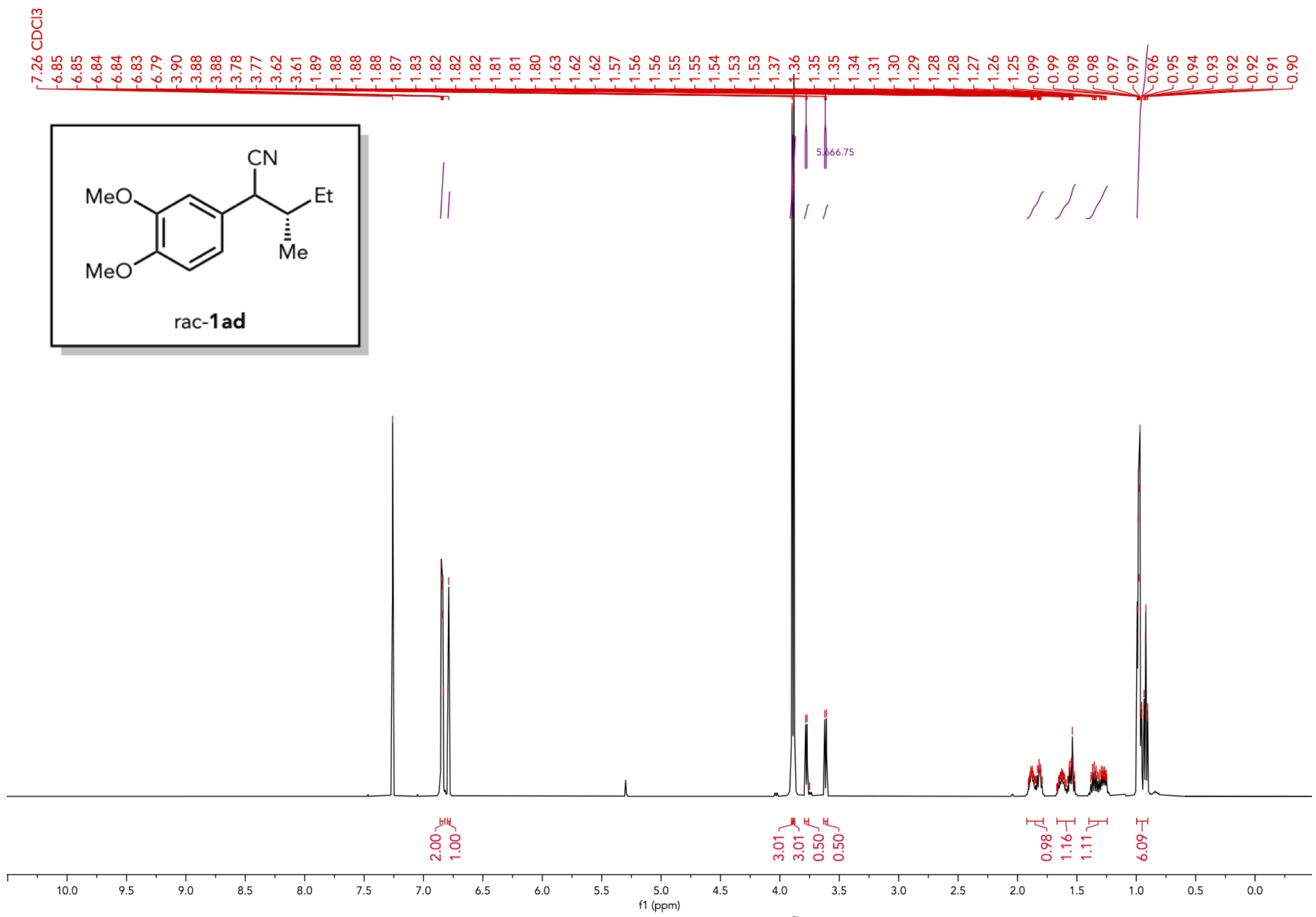

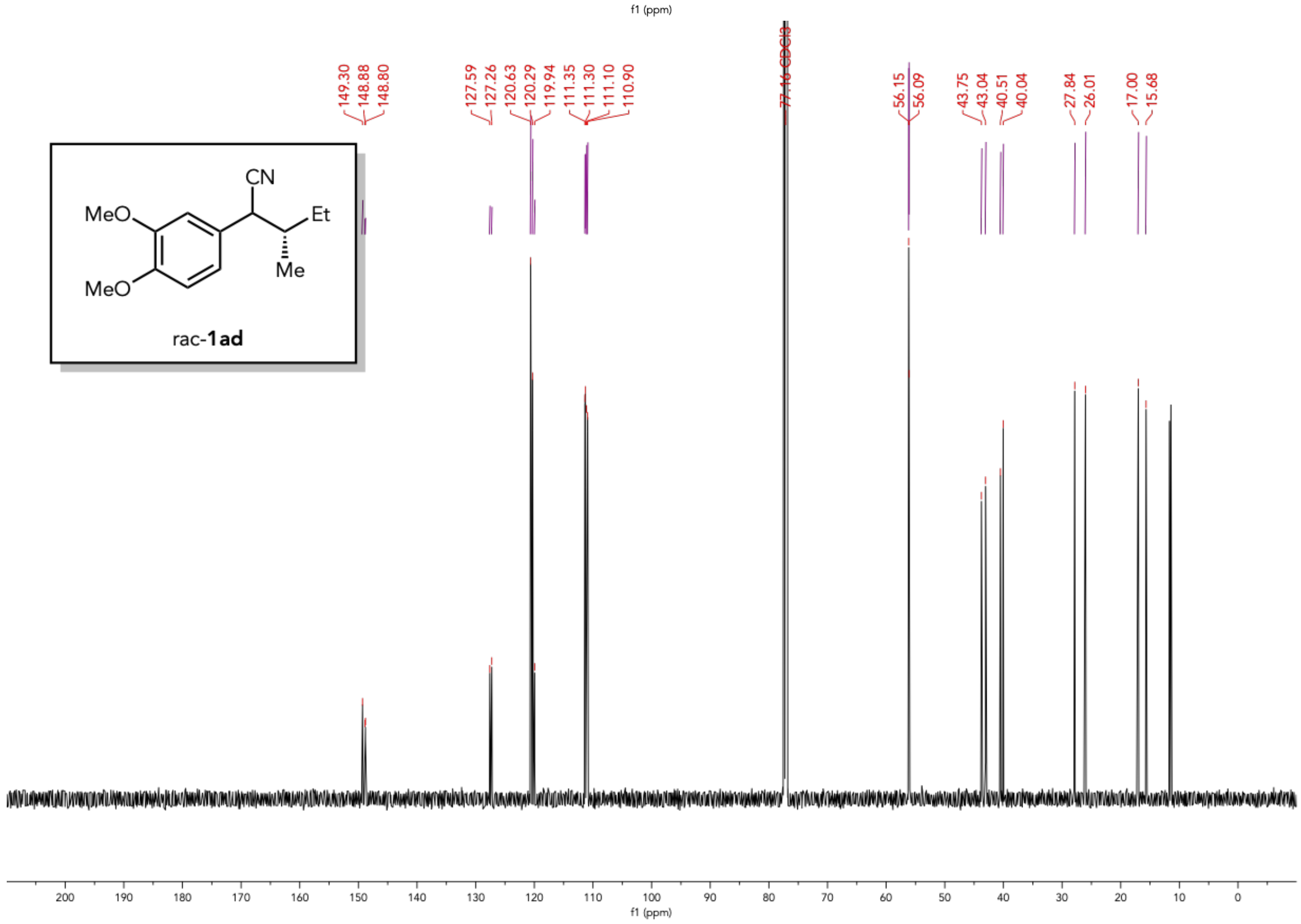

^{1}H NMR (500 MHz, $CDCl_3$) and ^{13}C NMR (126 MHz, $CDCl_3$) spectra for compound *rac*-**1ae**

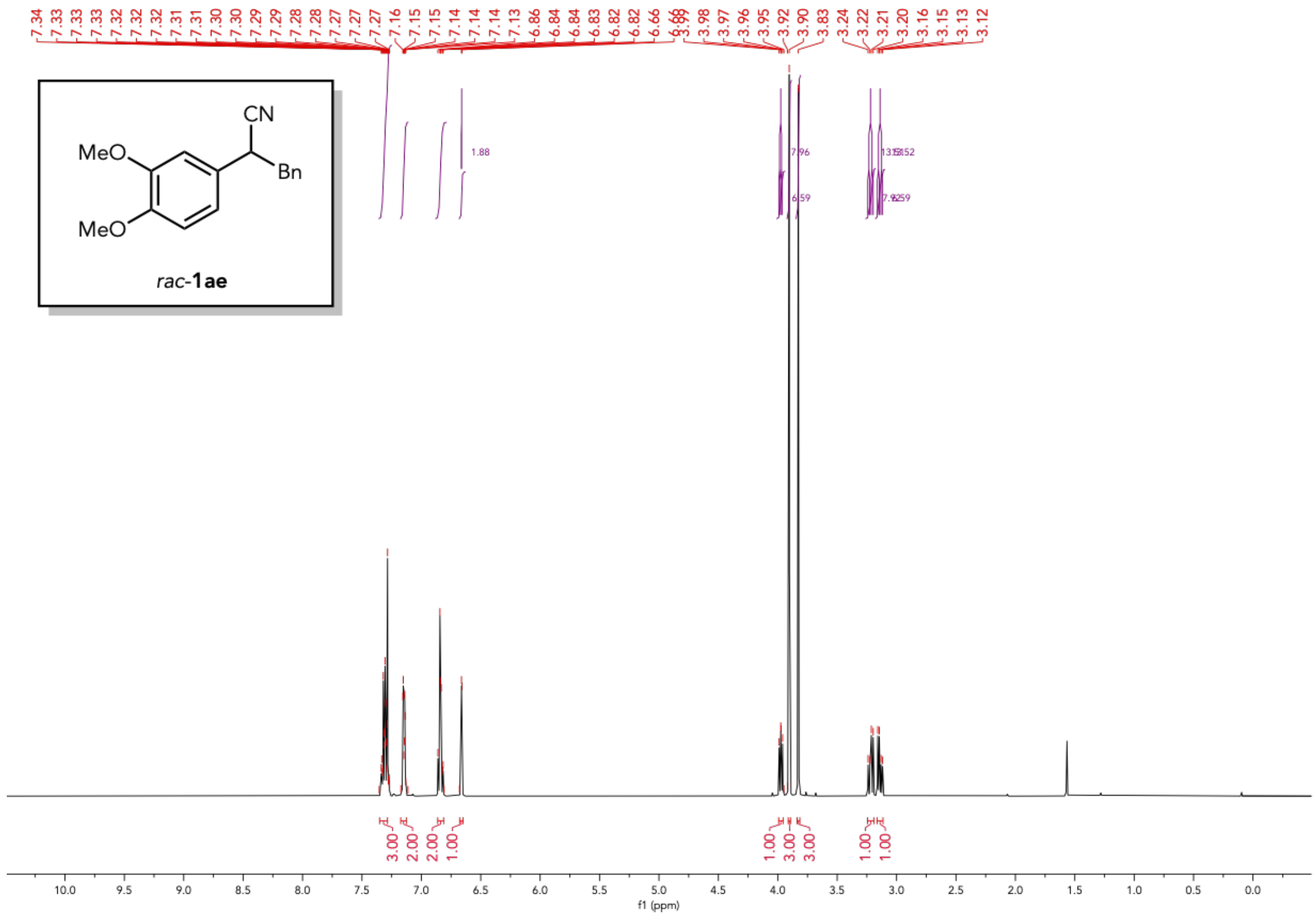

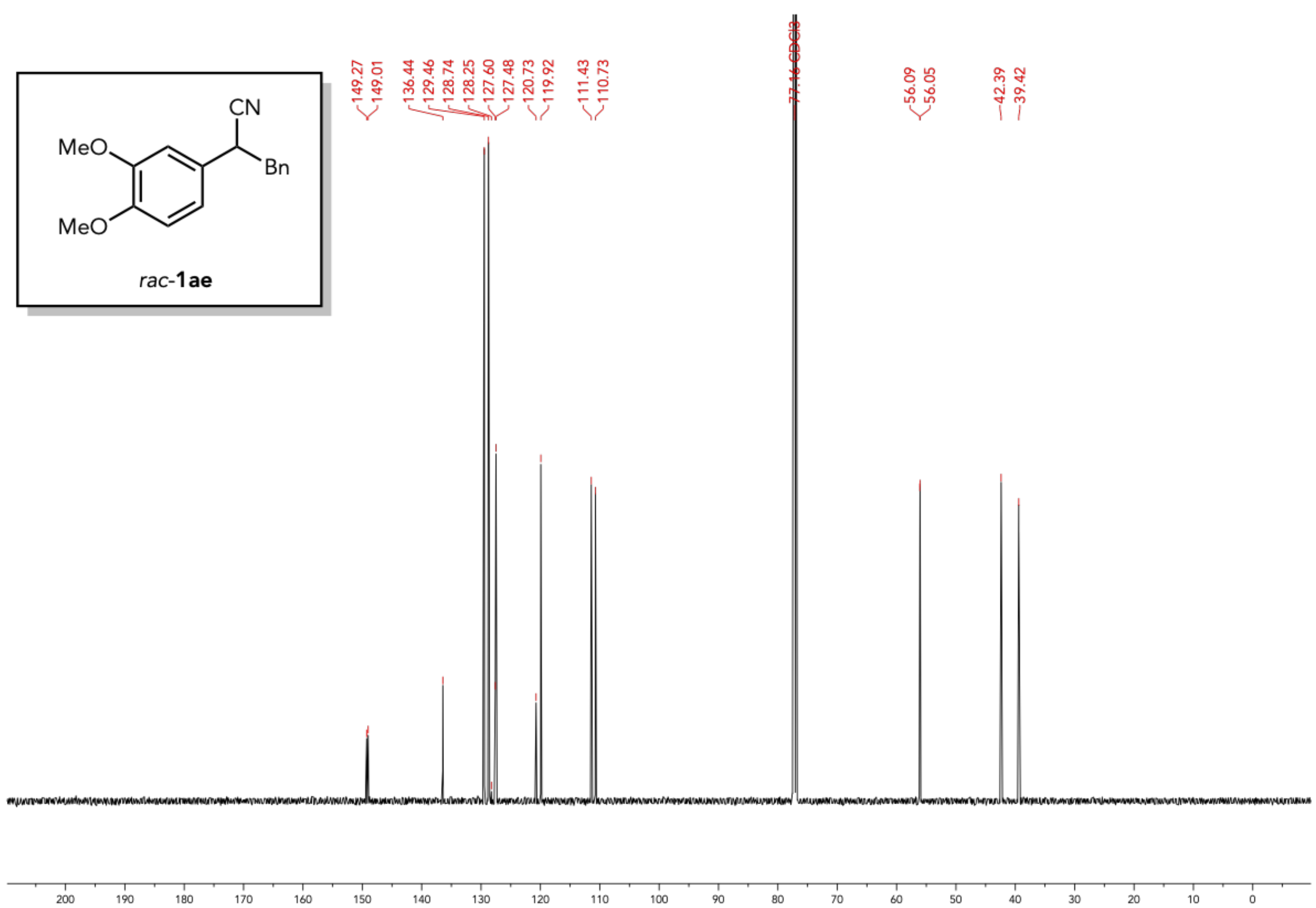

^1H NMR (500 MHz, $CDCl_3$) and ^{13}C NMR (126 MHz, $CDCl_3$) spectra for compound **2**

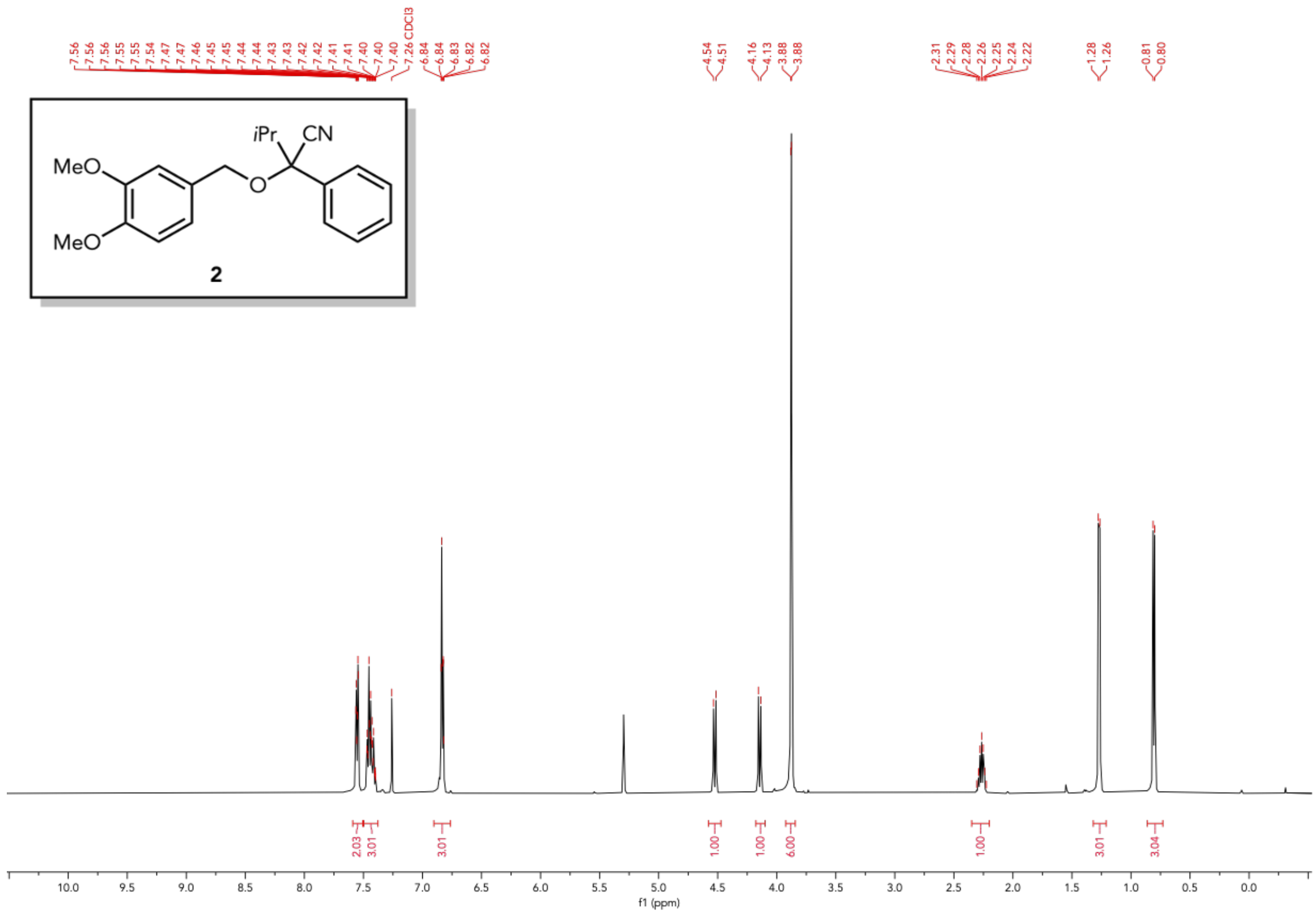

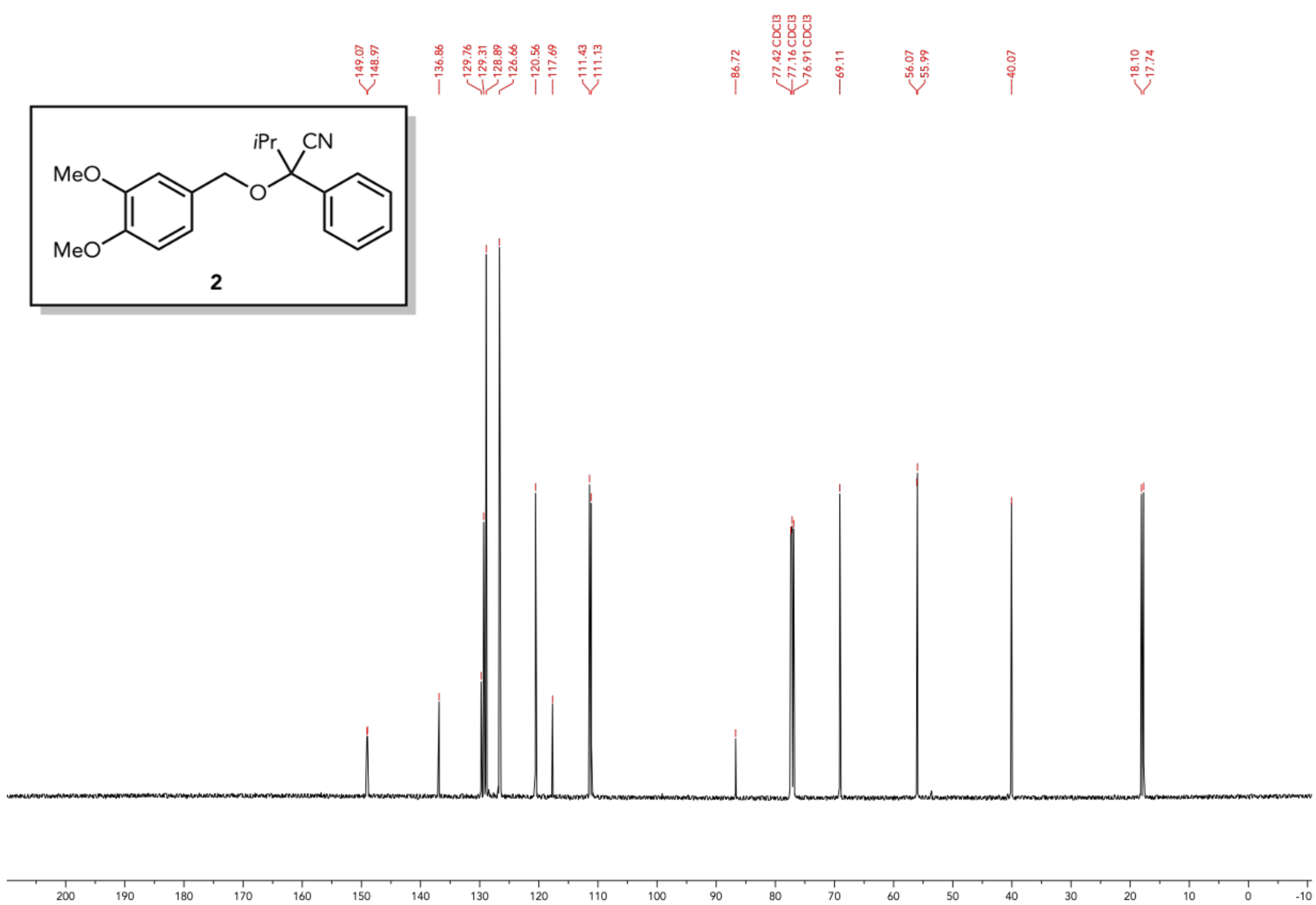

^{1}H NMR (500 MHz, $CDCl_3$) and ^{13}C NMR (126 MHz, $CDCl_3$) spectra for compound *rac*-**3**

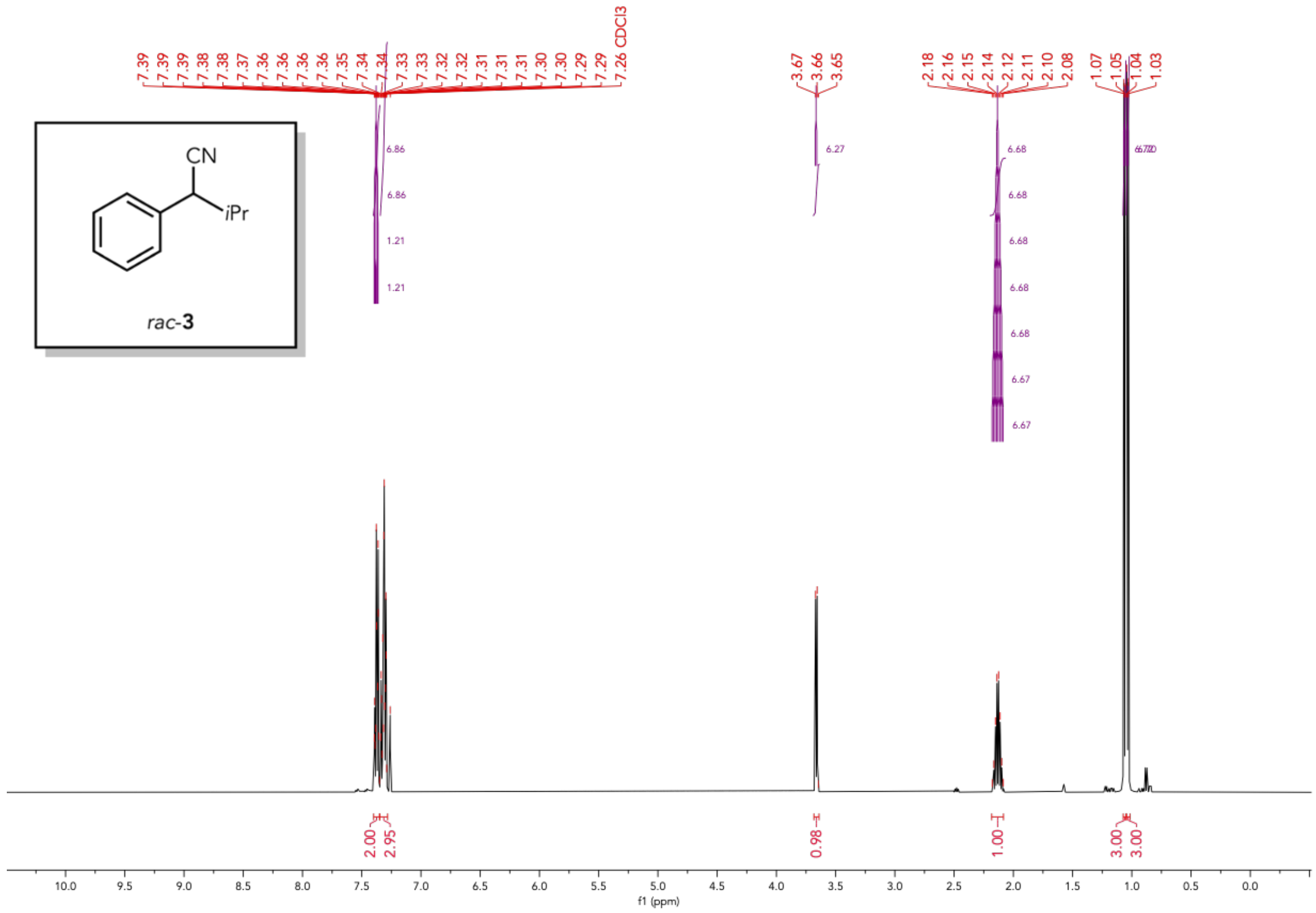

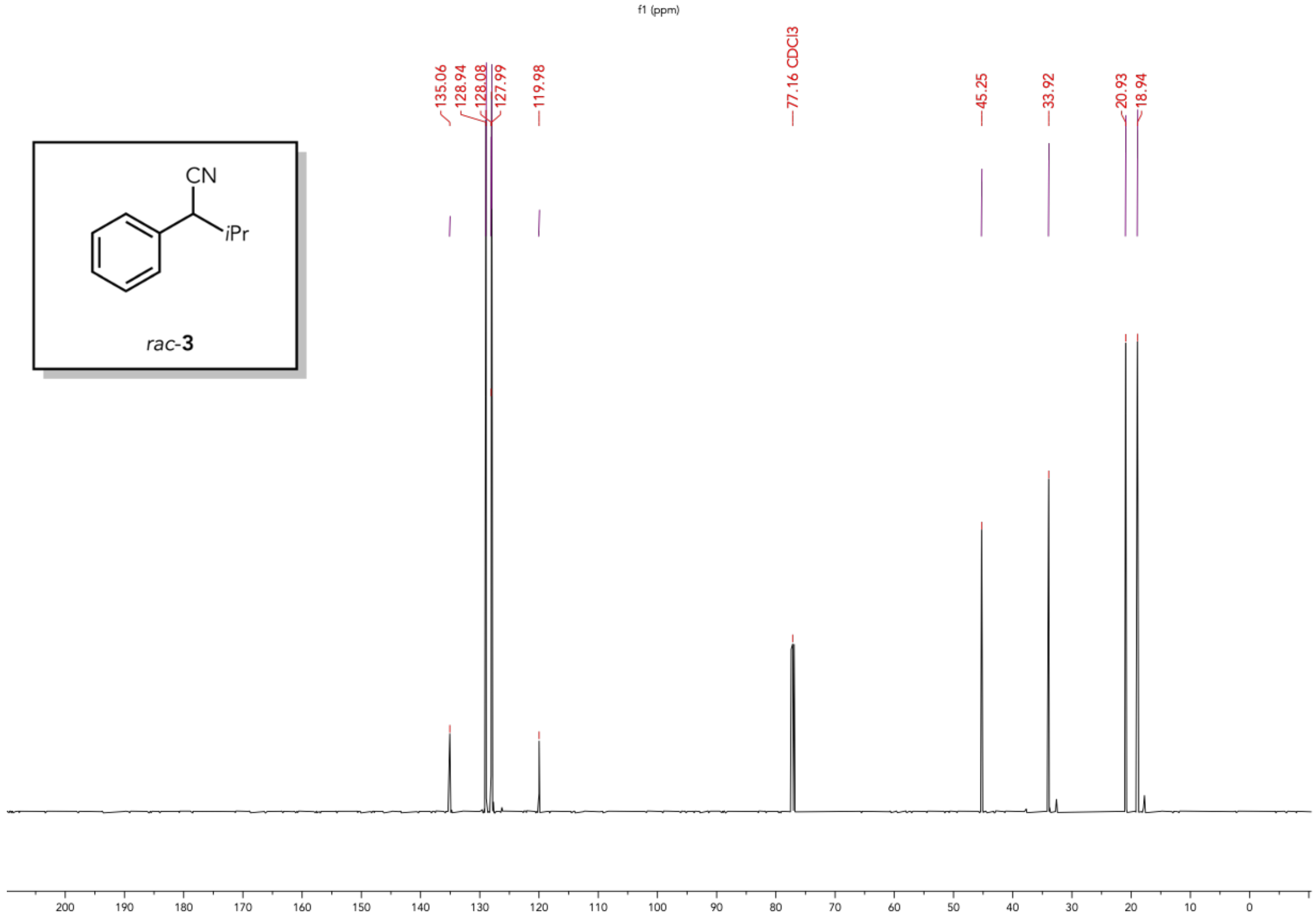

^{1}H NMR (500 MHz, $CDCl_3$) spectrum for compound **5**

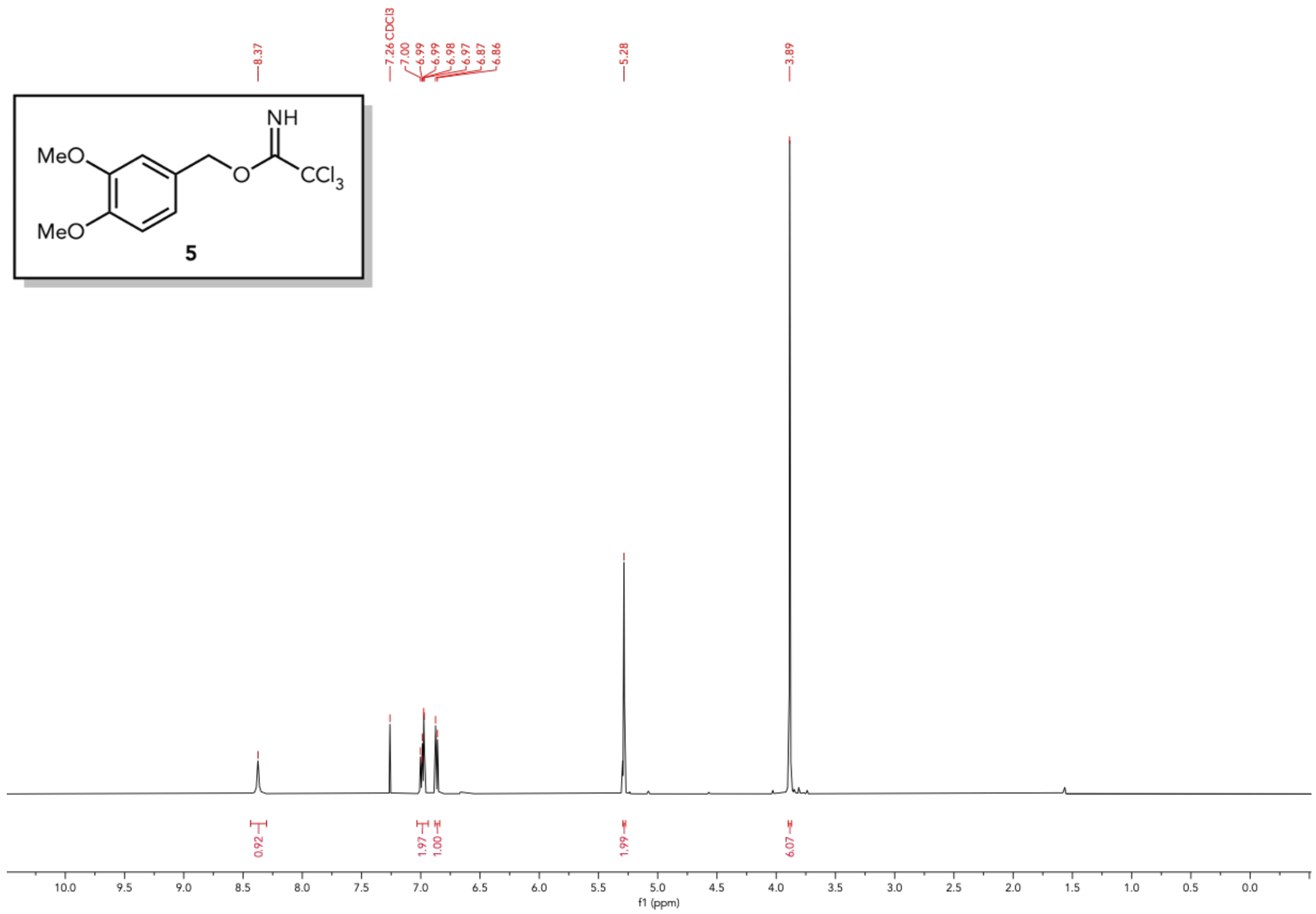

^{1}H NMR (500 MHz, $CDCl_3$) and ^{13}C NMR (126 MHz, $CDCl_3$) spectra for compound *rac*-**6**

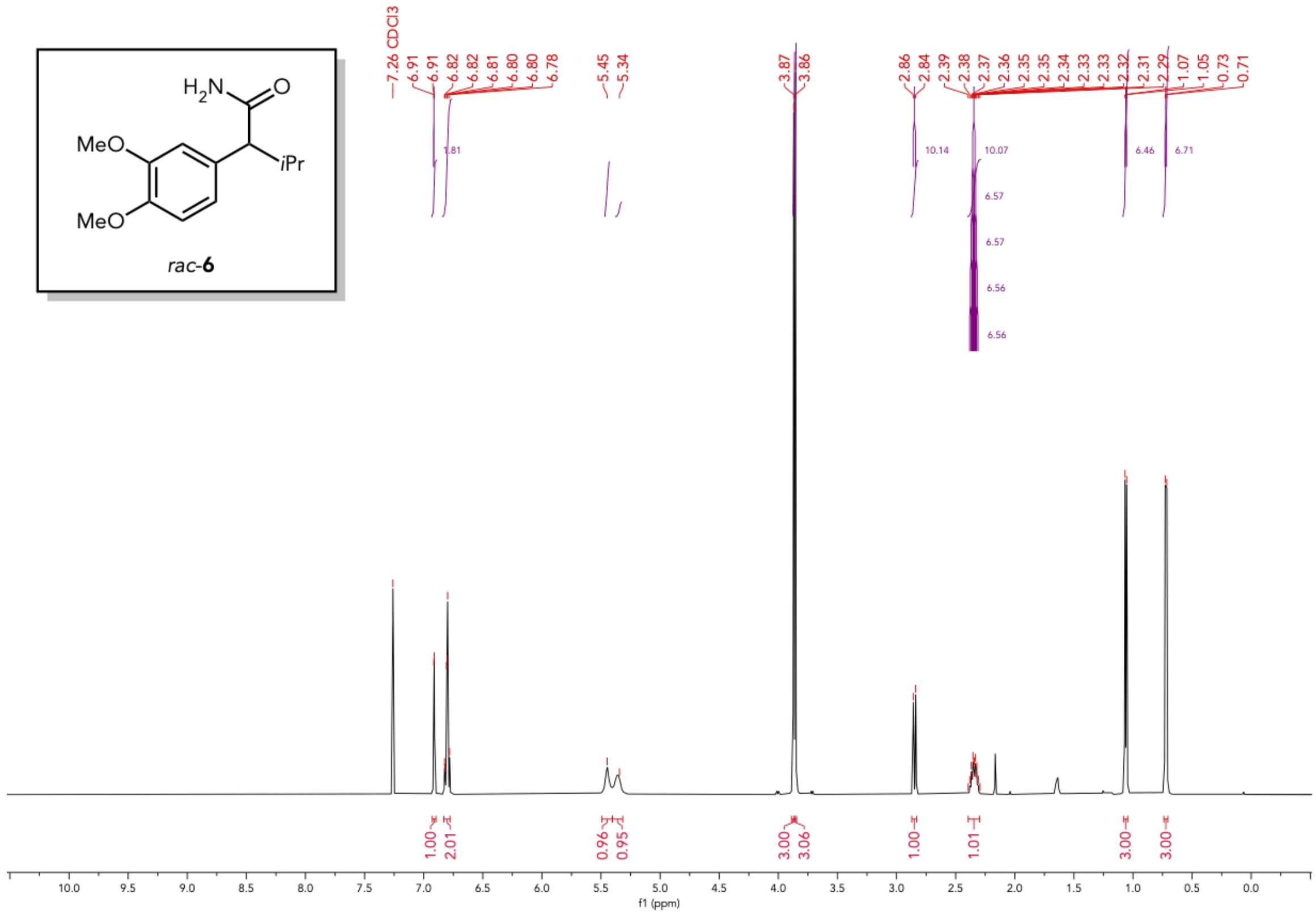

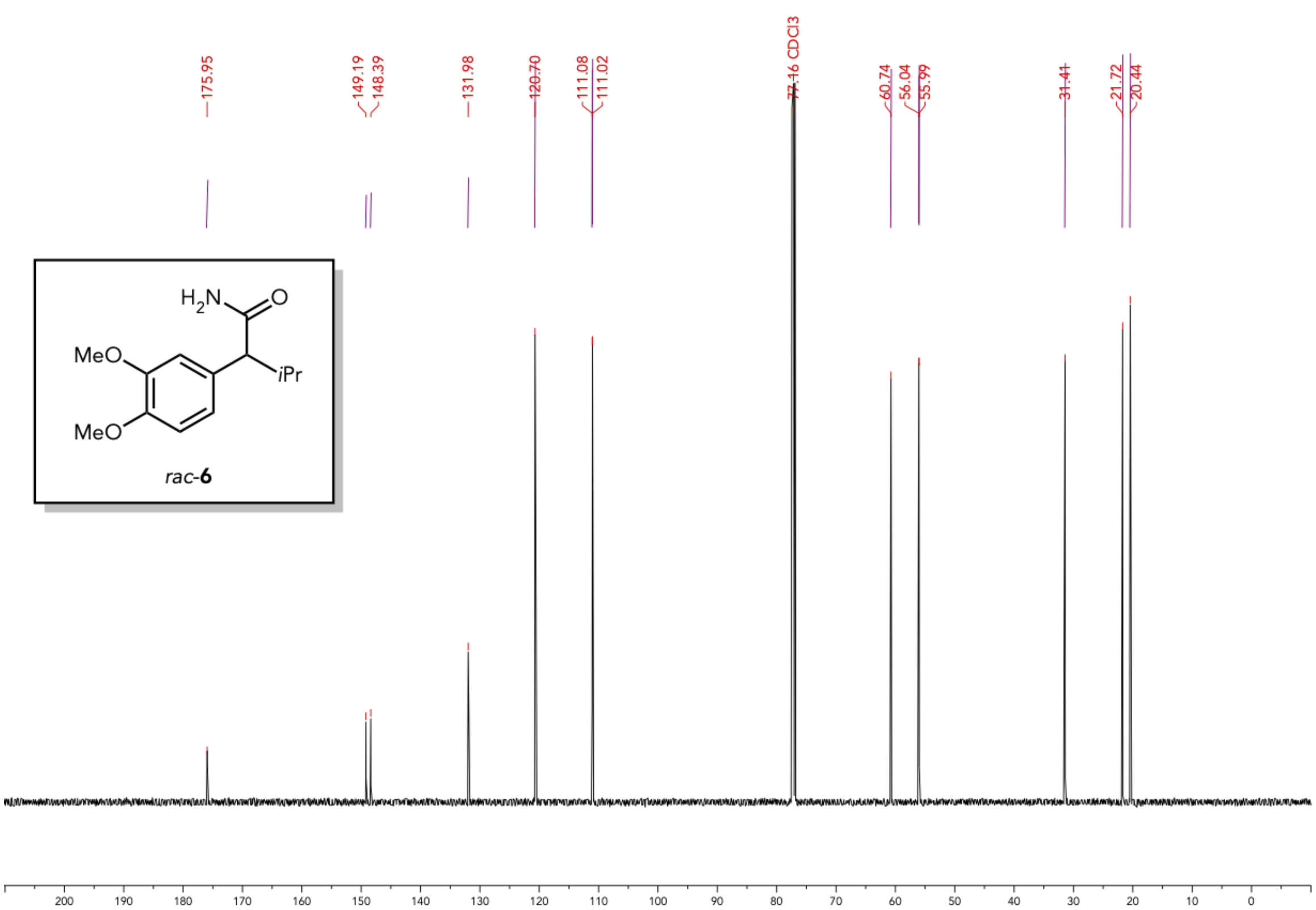

^{1}H and ^{13}C NMR spectra of deracemized substrates

^{1}H and ^{13}C spectra for compounds have previously been reported following racemic synthesis in the above section, and are reported herein following deracemization with ^{1}H only. Exceptions are (*S*)-**1a-*d*$_1$**, (2*S*)-**1ad**, (2*R*)-**1ad**, (*S)*-**1ae-*d*$_1$**, for which ^{13}C NMR are included.

^{1}H NMR (500 MHz, $CDCl_3$) spectrum for compound (*S*)-**1a**

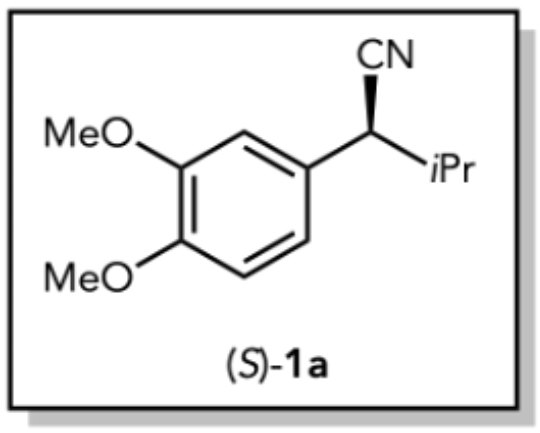

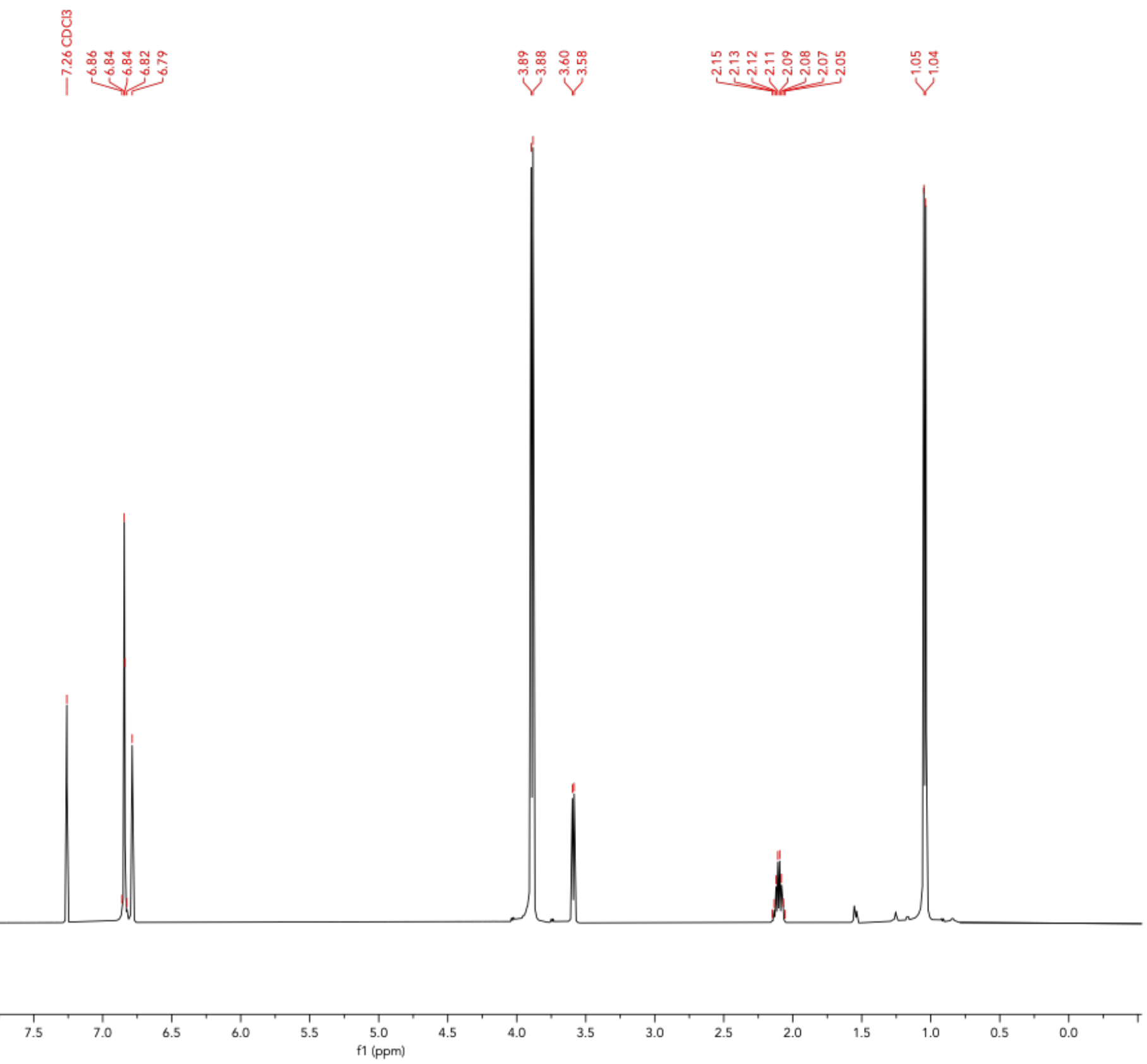

^{1}H NMR (500 MHz, $CDCl_3$) and ^{13}C NMR (126 MHz, $CDCl_3$) spectra for compound (*S*)-**1a-*d*₁**

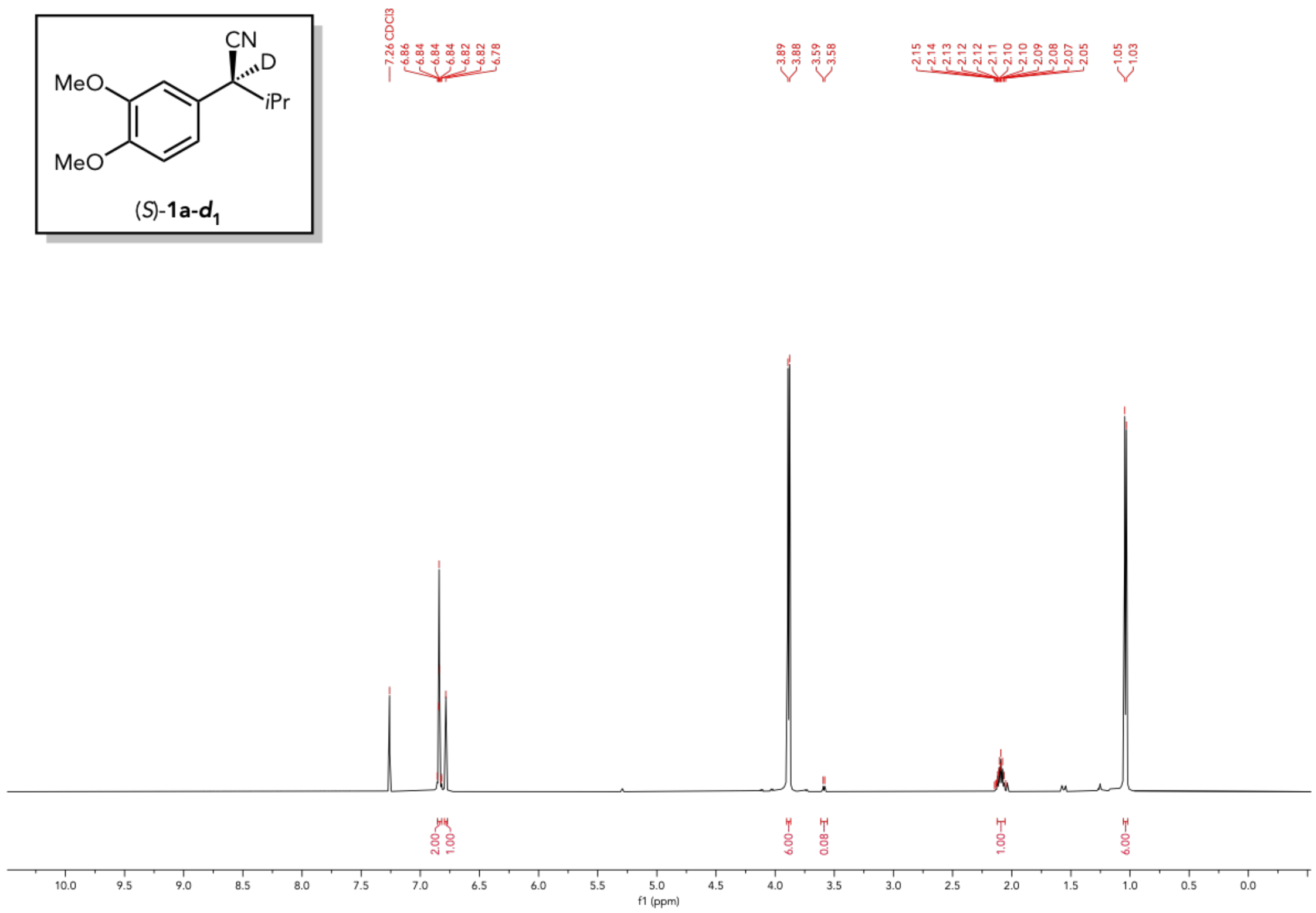

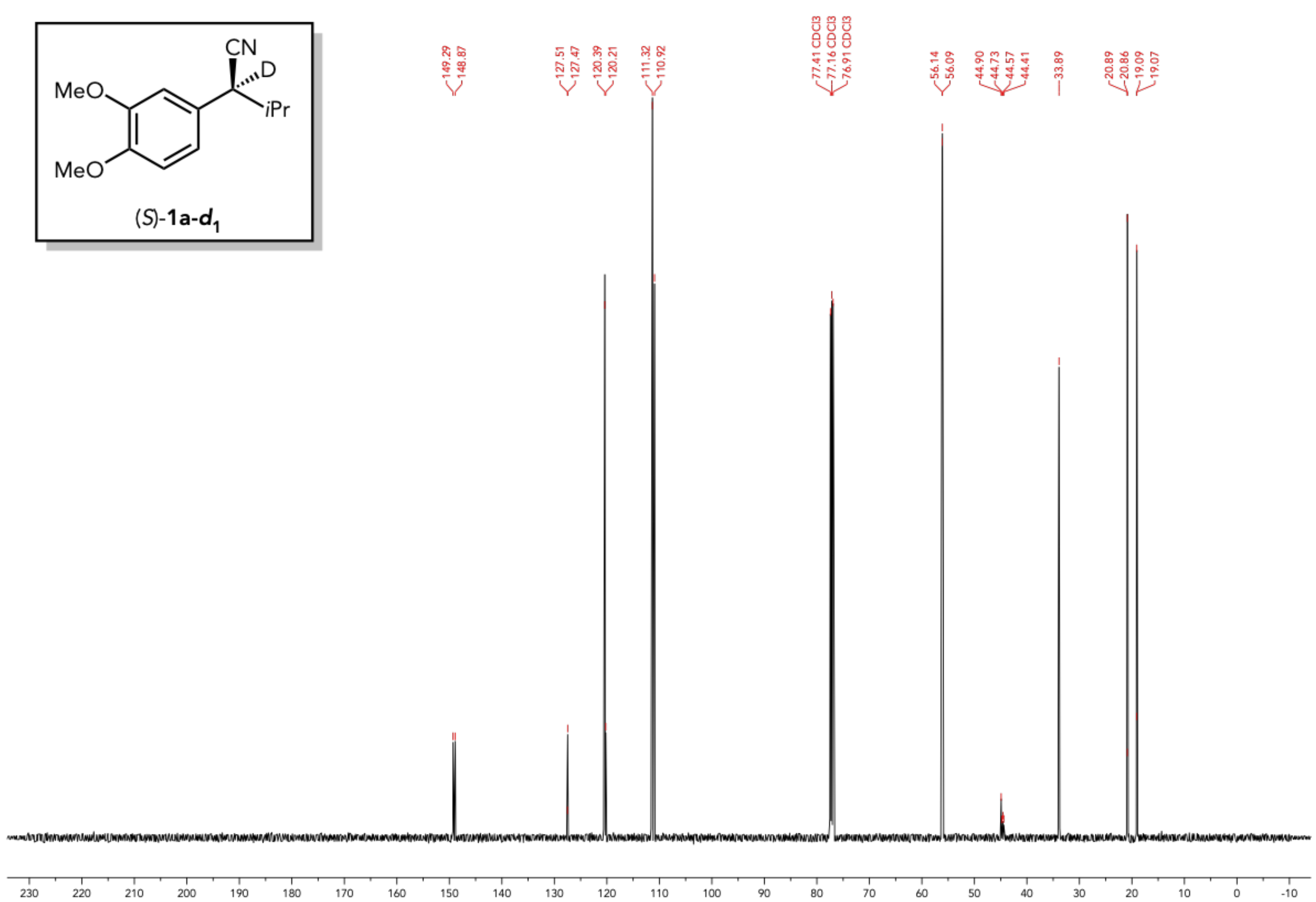

^{1}H NMR (500 MHz, $CDCl_3$) spectrum for compound (*S*)-**1b**

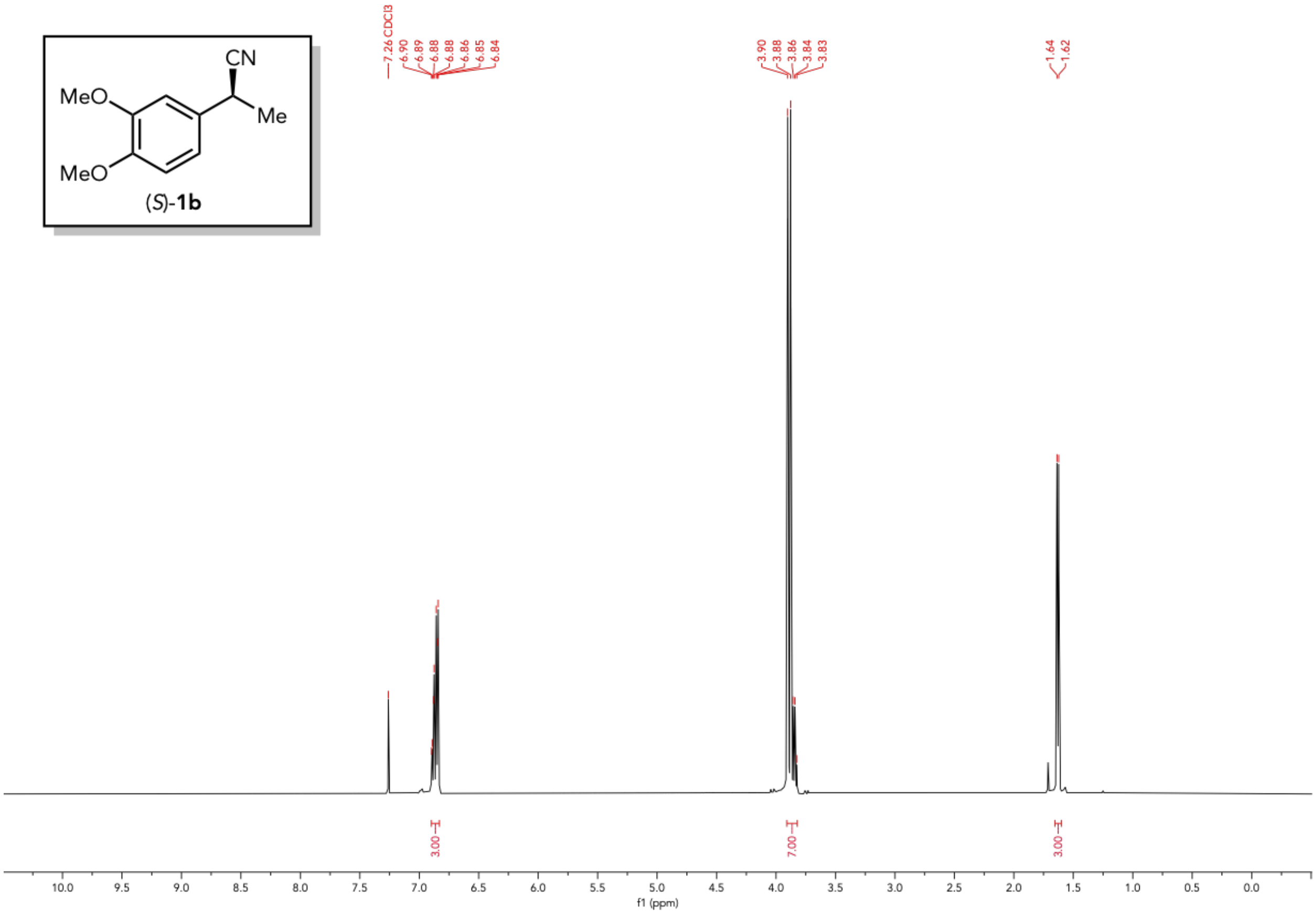

^{1}H NMR (500 MHz, $CDCl_3$) spectrum for compound (*S*)-**1c**

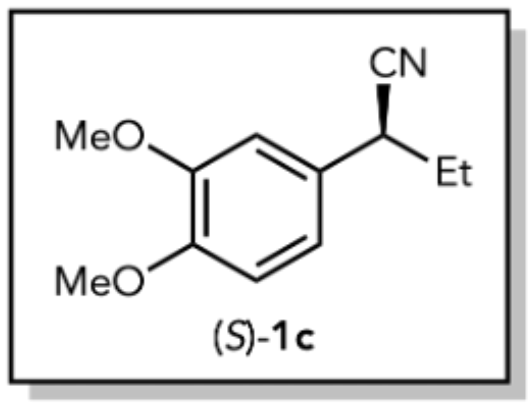

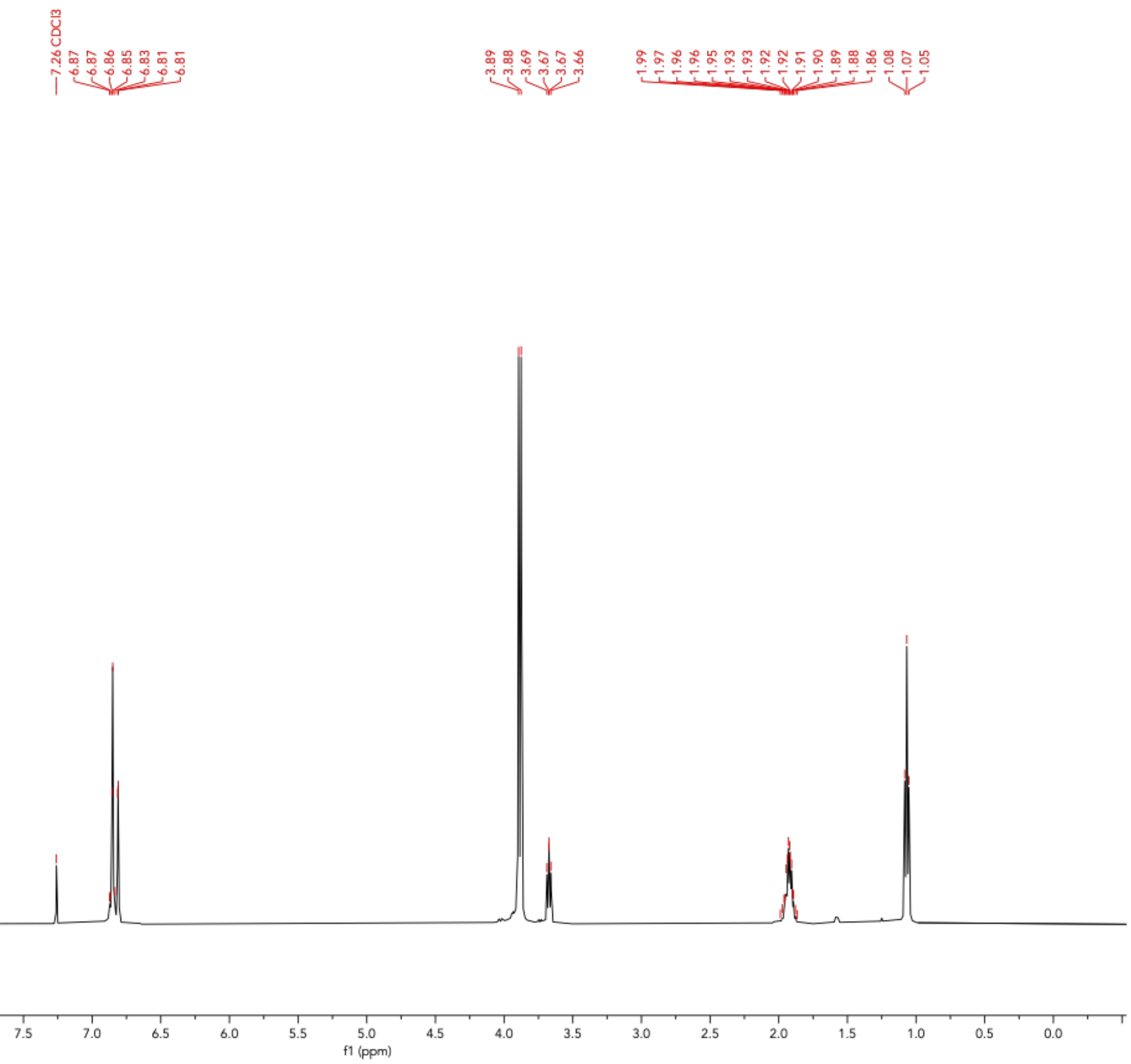

^{1}H NMR (500 MHz, $CDCl_3$) spectrum for compound (*S*)-**1d**

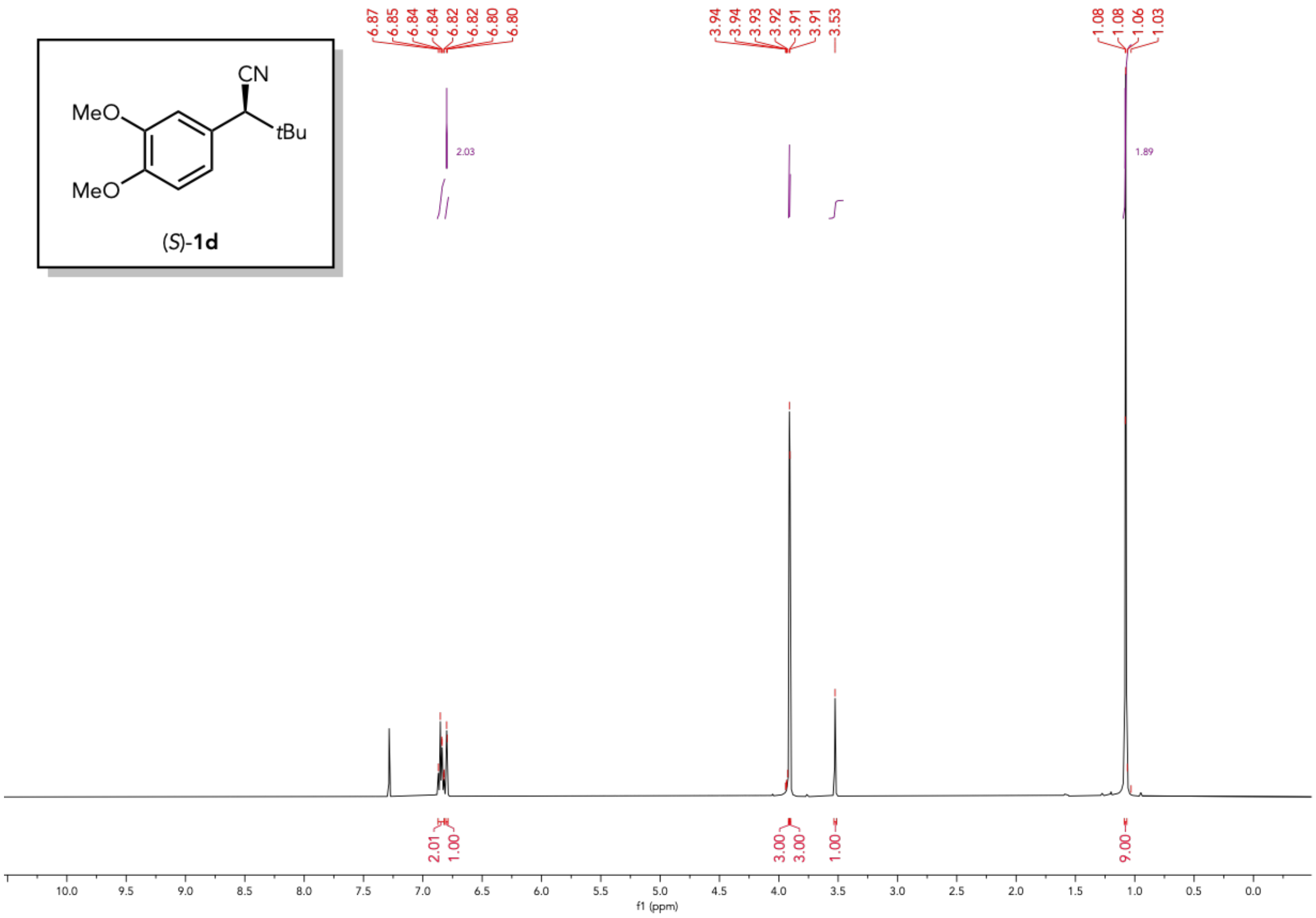

^{1}H NMR (500 MHz, $CDCl_3$) spectrum for compound (*S*)-**1e**

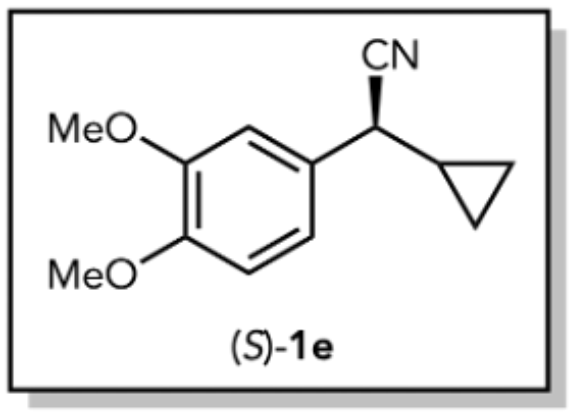

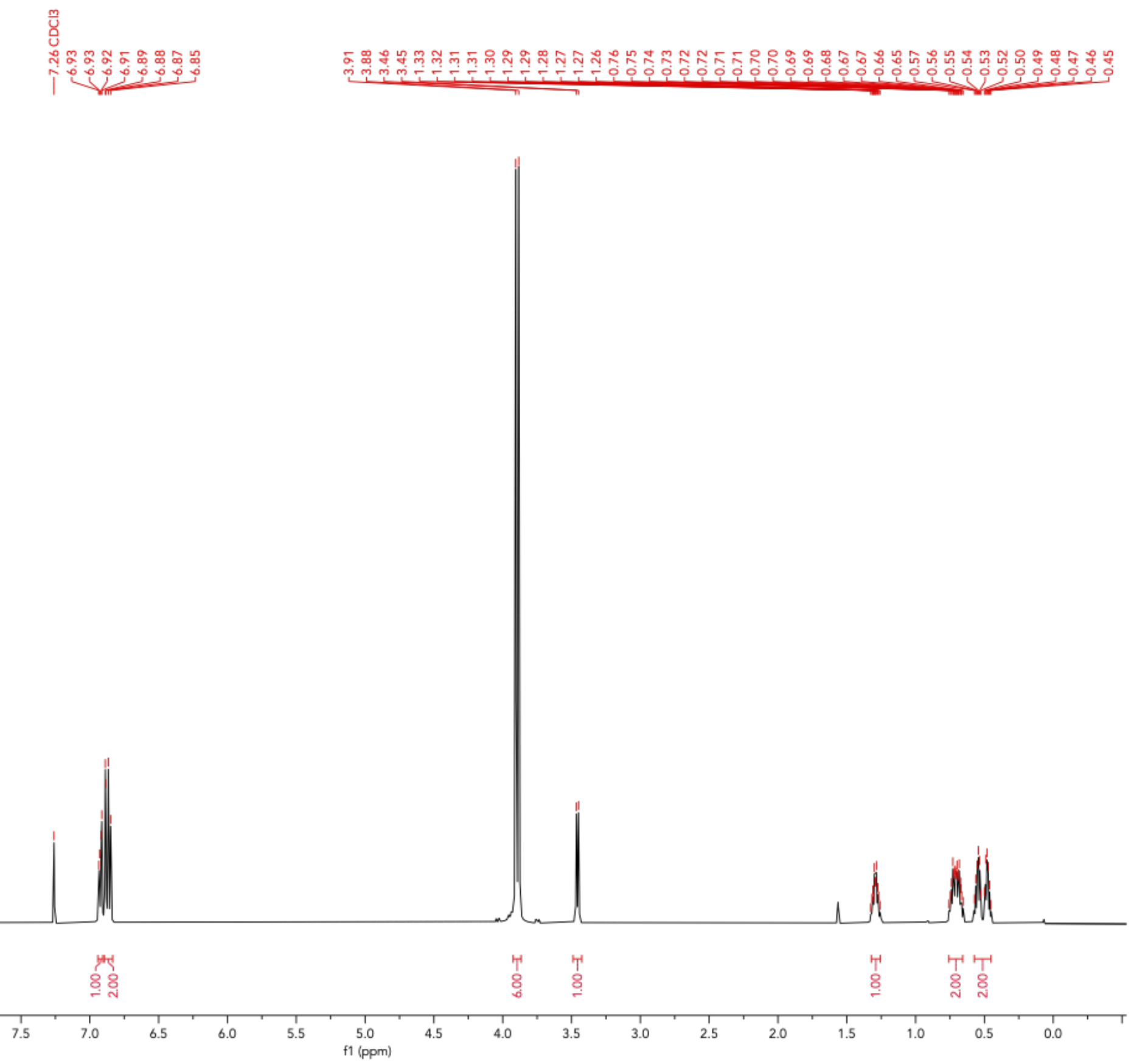

^{1}H NMR (500 MHz, $CDCl_3$) spectrum for compound (*S*)-**1f**

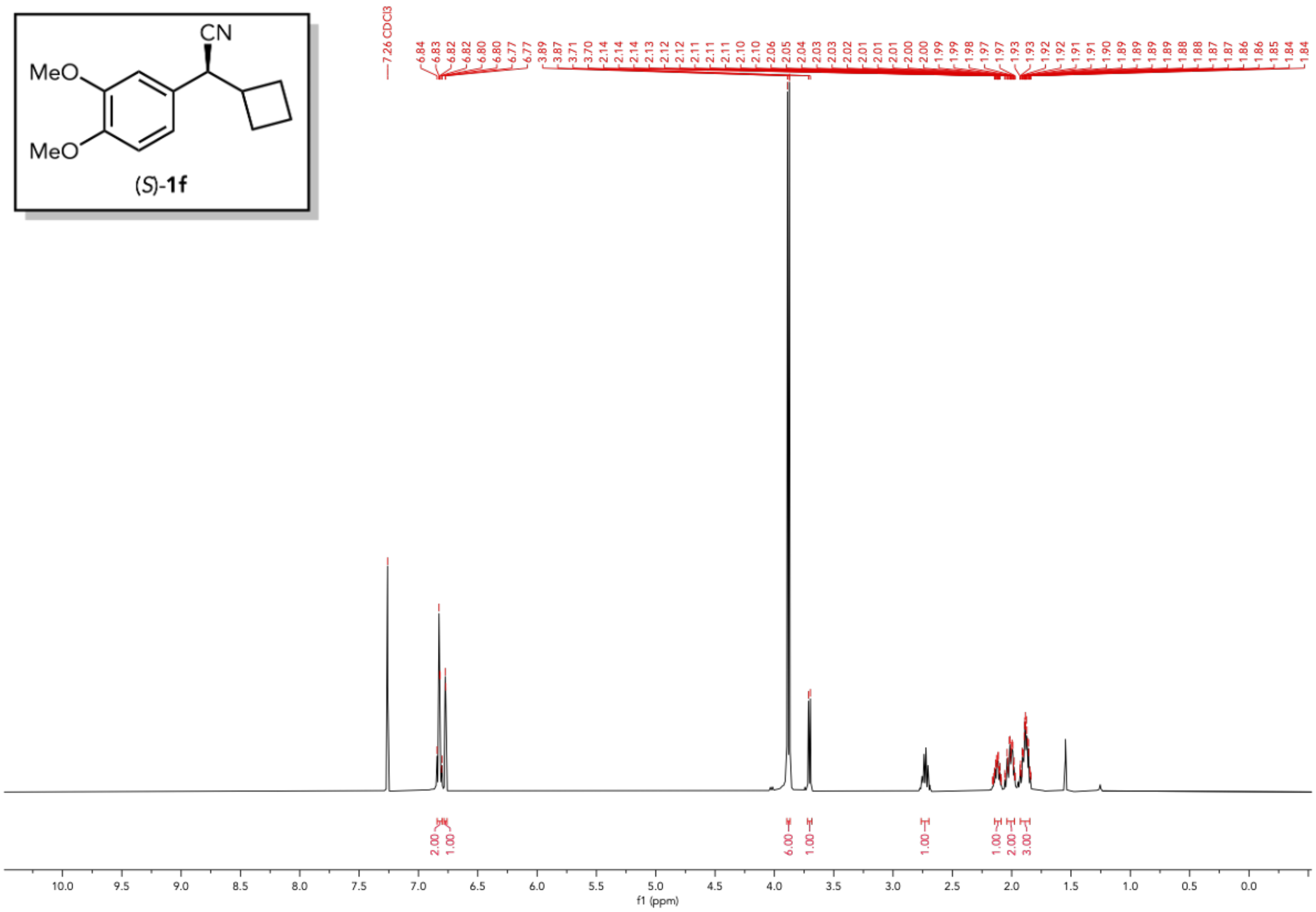

^{1}H NMR (500 MHz, $CDCl_3$) spectrum for compound (*S*)-**1g**

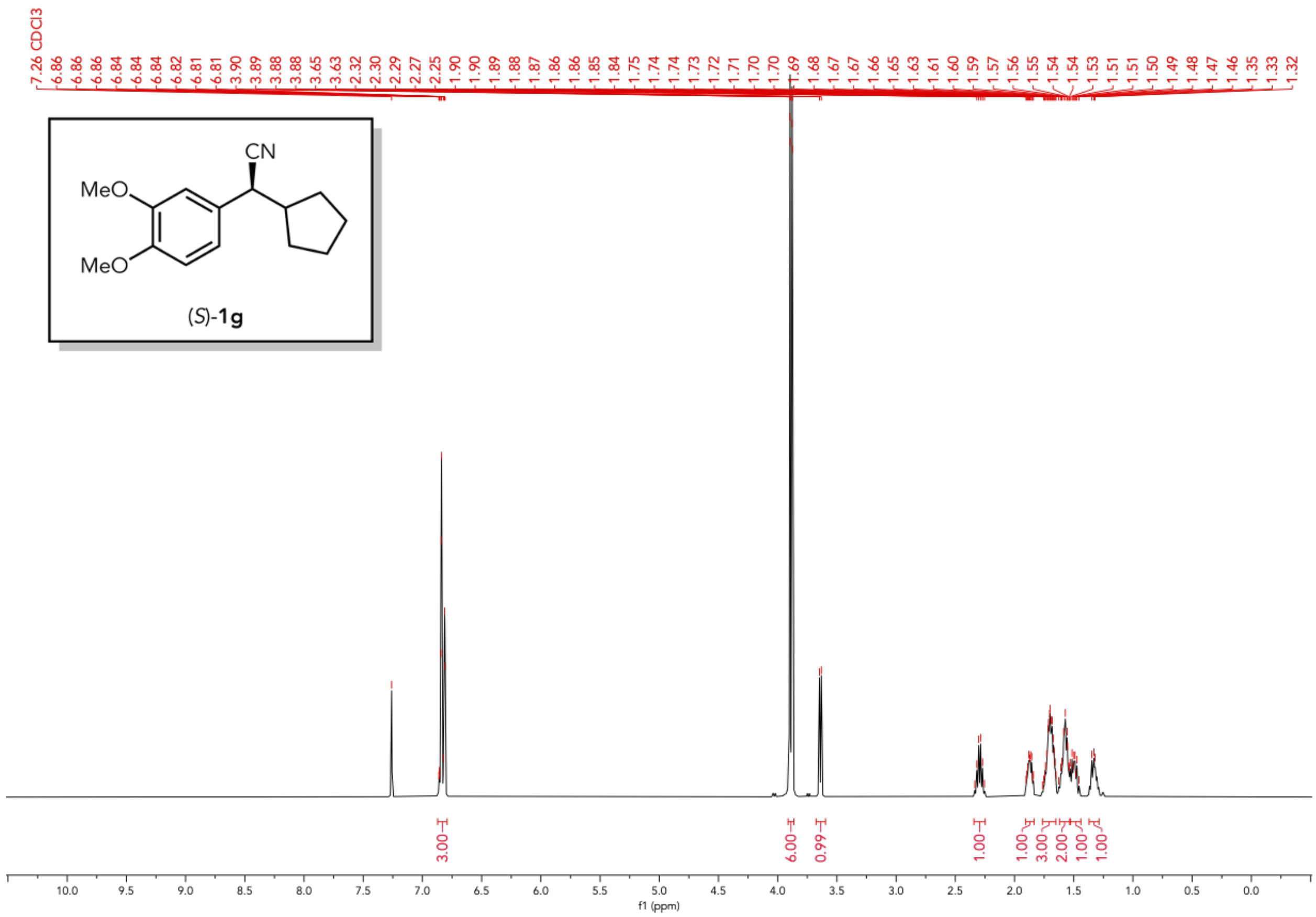

^{1}H NMR (500 MHz, $CDCl_3$) spectrum for compound (*S*)-**1h**

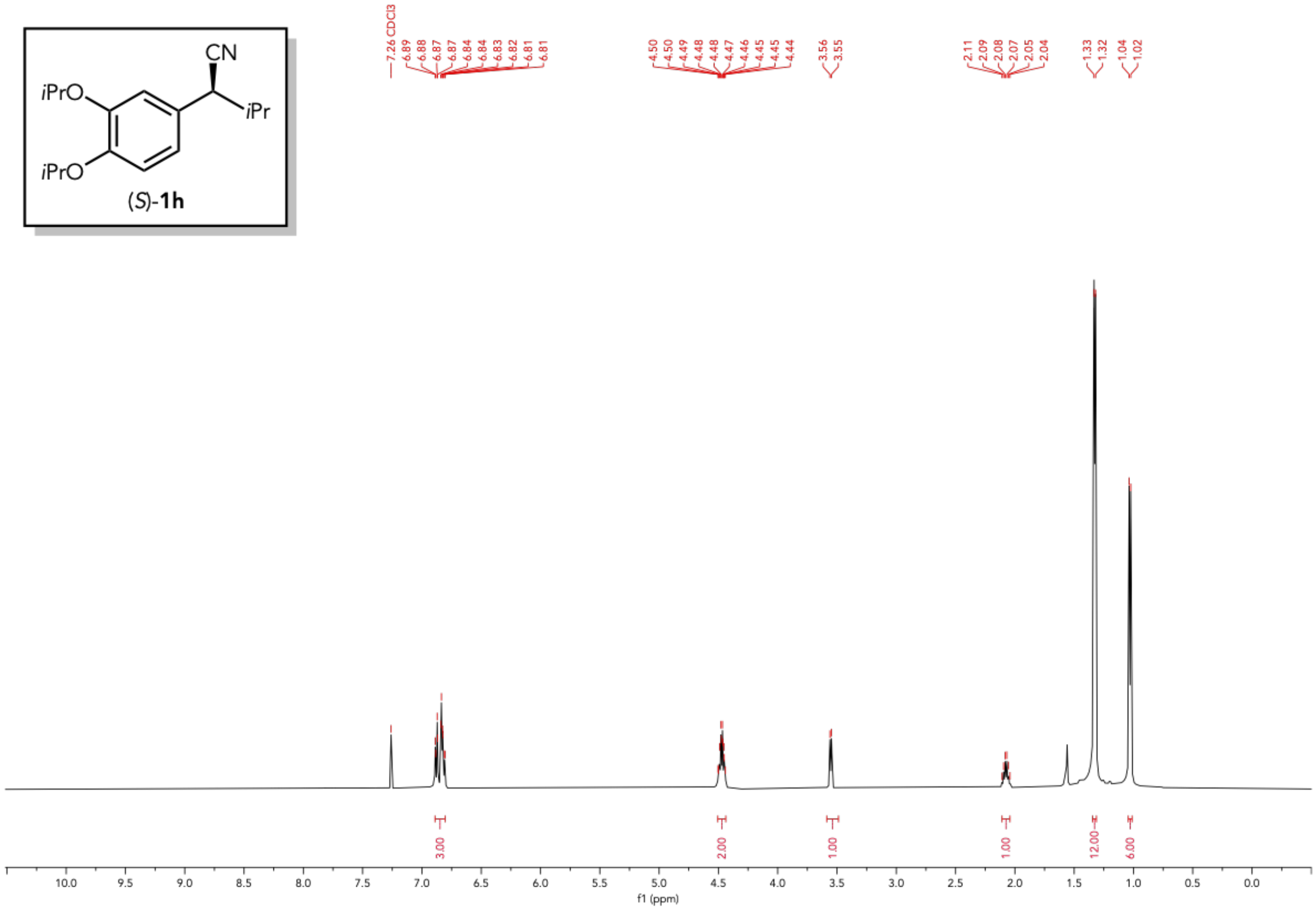

^{1}H NMR (500 MHz, $CDCl_3$) spectrum for compound (*S*)-**1i**

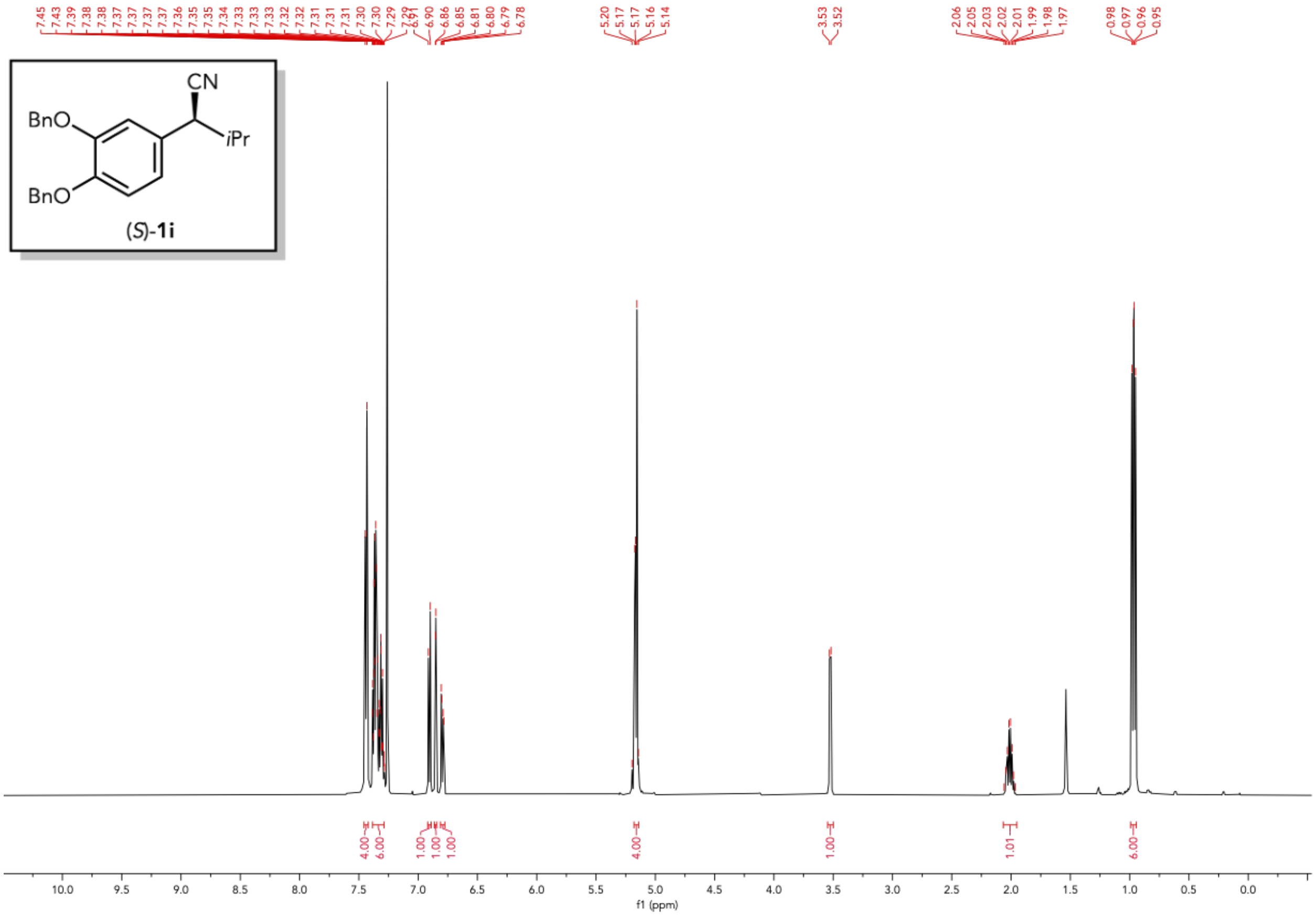

^{1}H NMR (500 MHz, $CDCl_3$) spectrum for compound (*S*)-**1j**

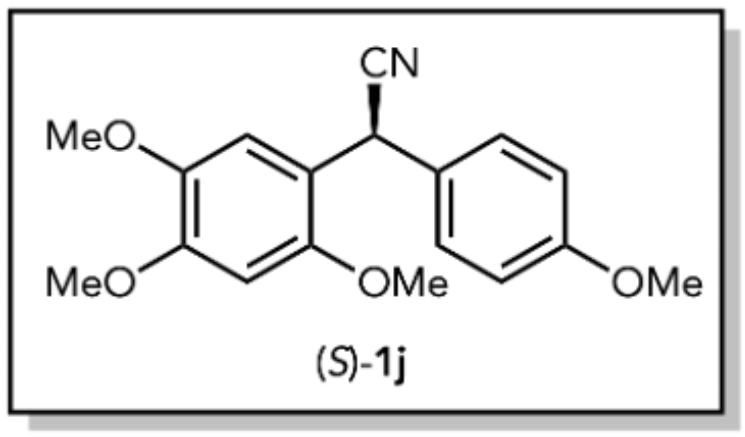

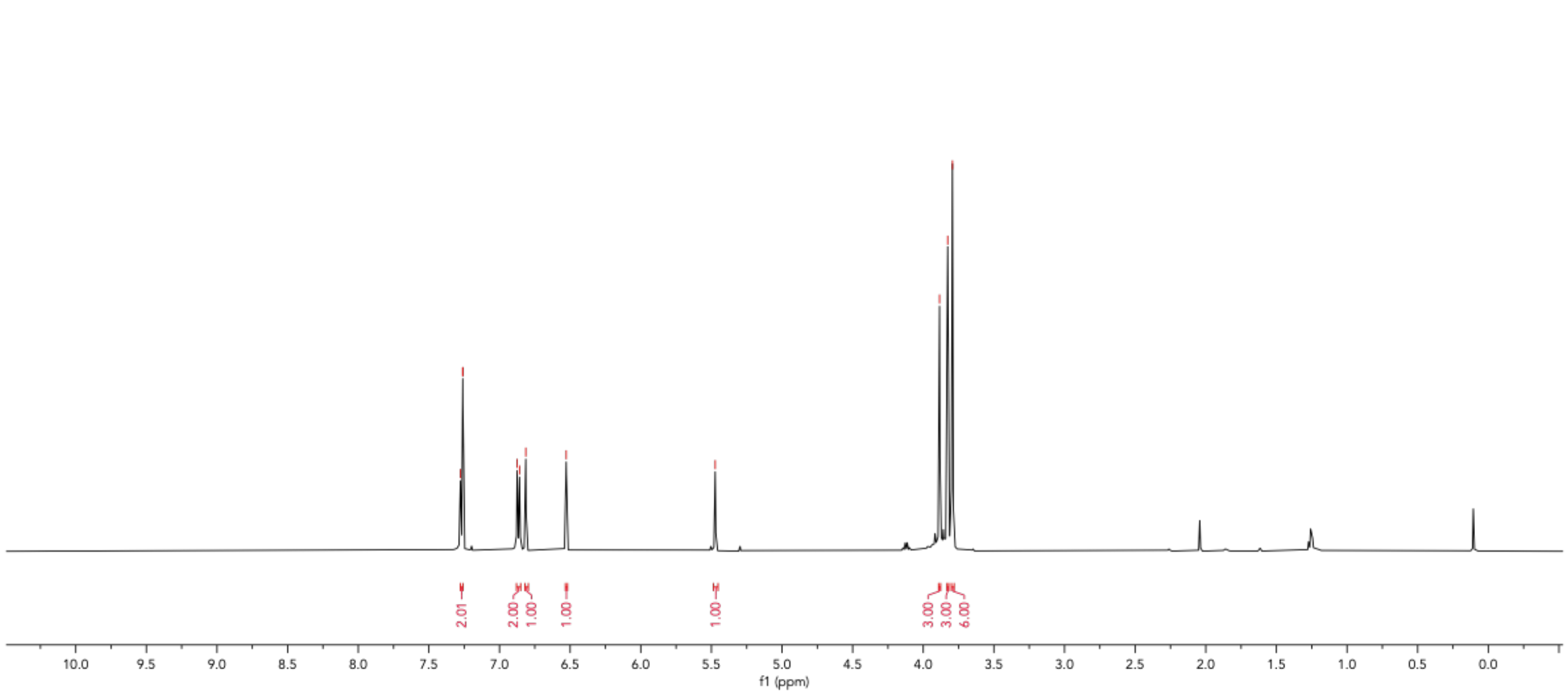

^{1}H NMR (500 MHz, $CDCl_3$) spectrum for compound (*S*)-**1k**

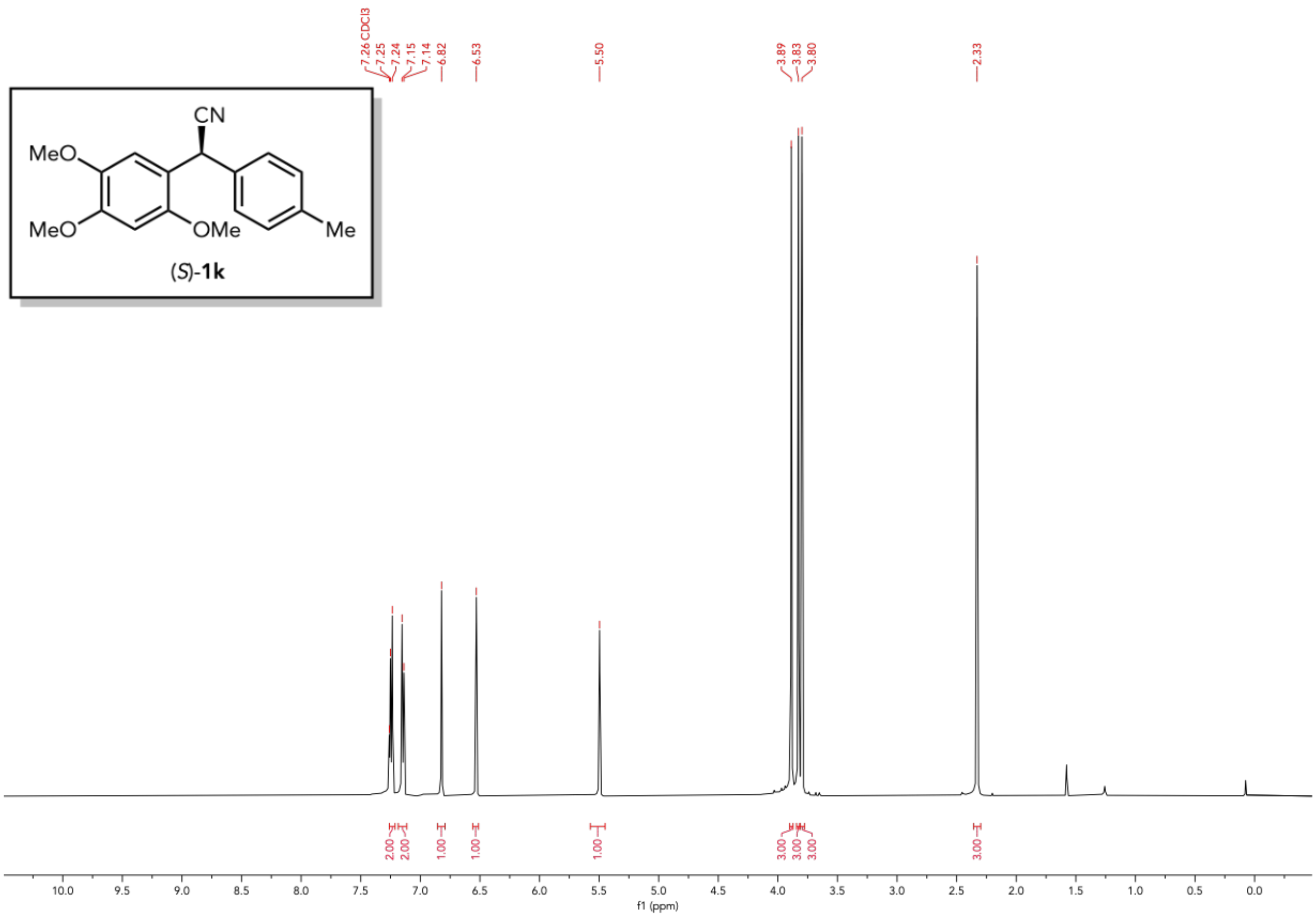

^{1}H NMR (500 MHz, $CDCl_3$) spectrum for compound (*S*)-**1l**

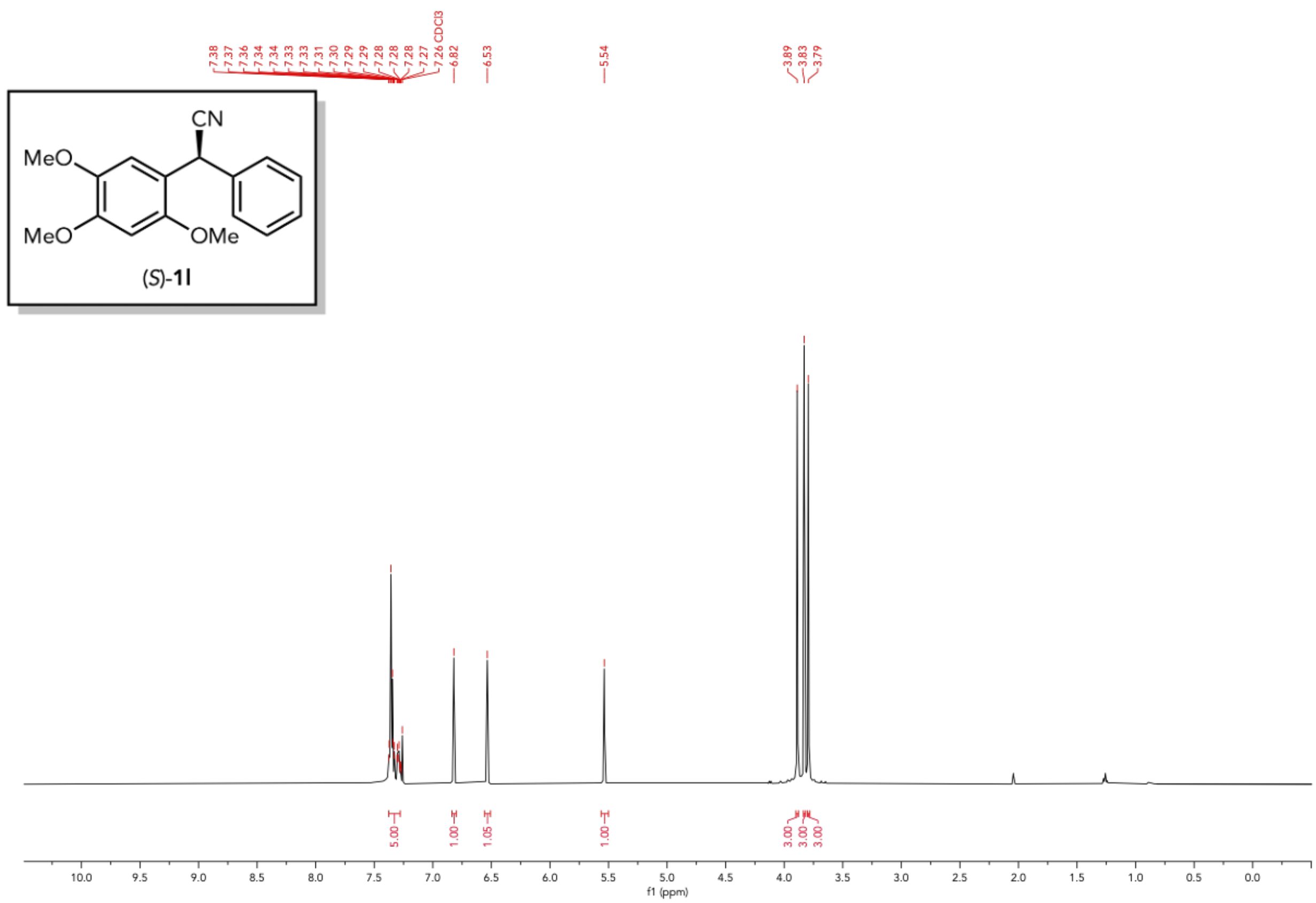

^{1}H NMR (500 MHz, $CDCl_3$) spectrum for compound (*S*)-**1m**

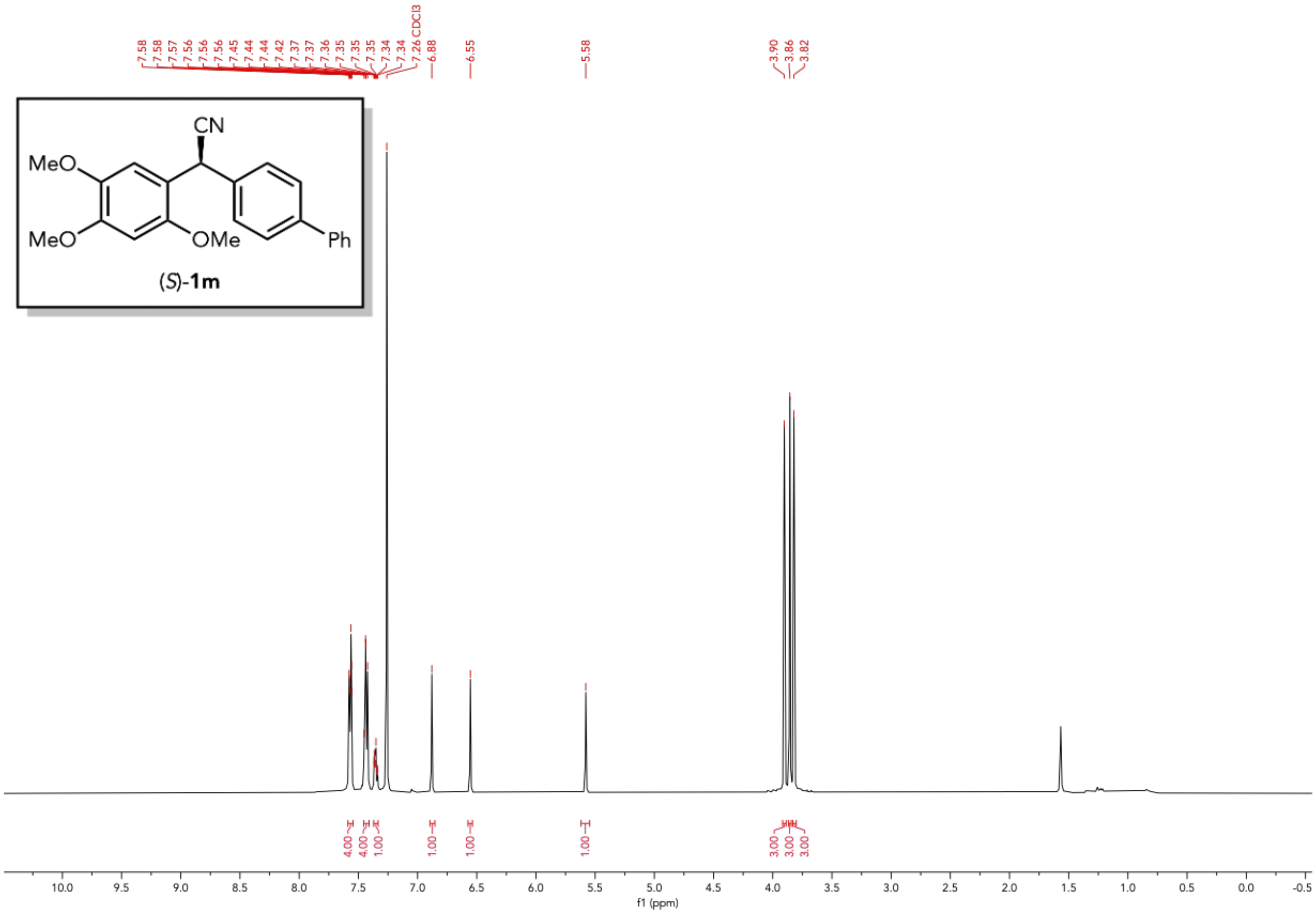

^{1}H NMR (500 MHz, CDCl₃) spectrum for compound (*S*)-**1n**

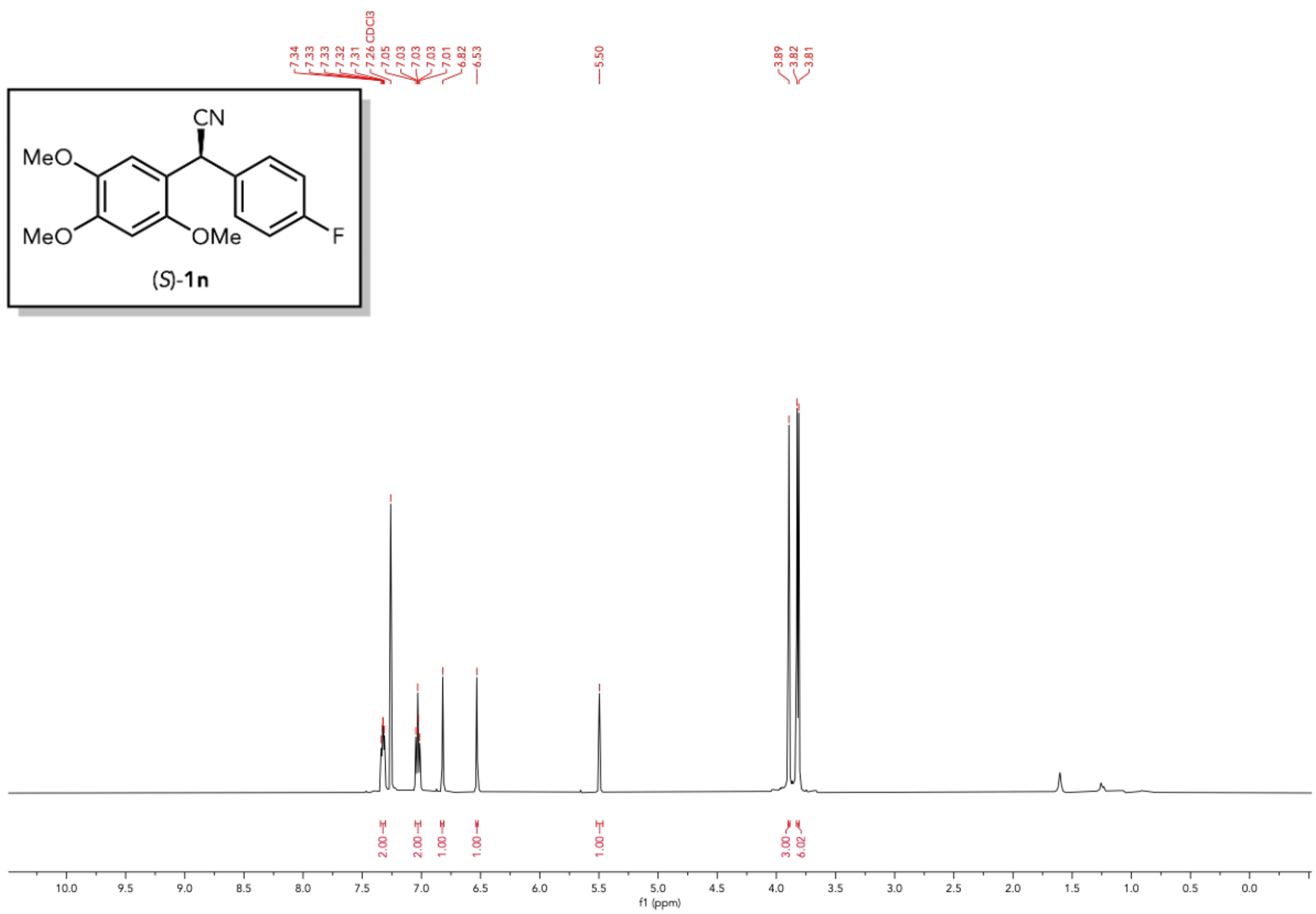

^{1}H NMR (500 MHz, $CDCl_3$) spectrum for compound (*S*)-**1o**

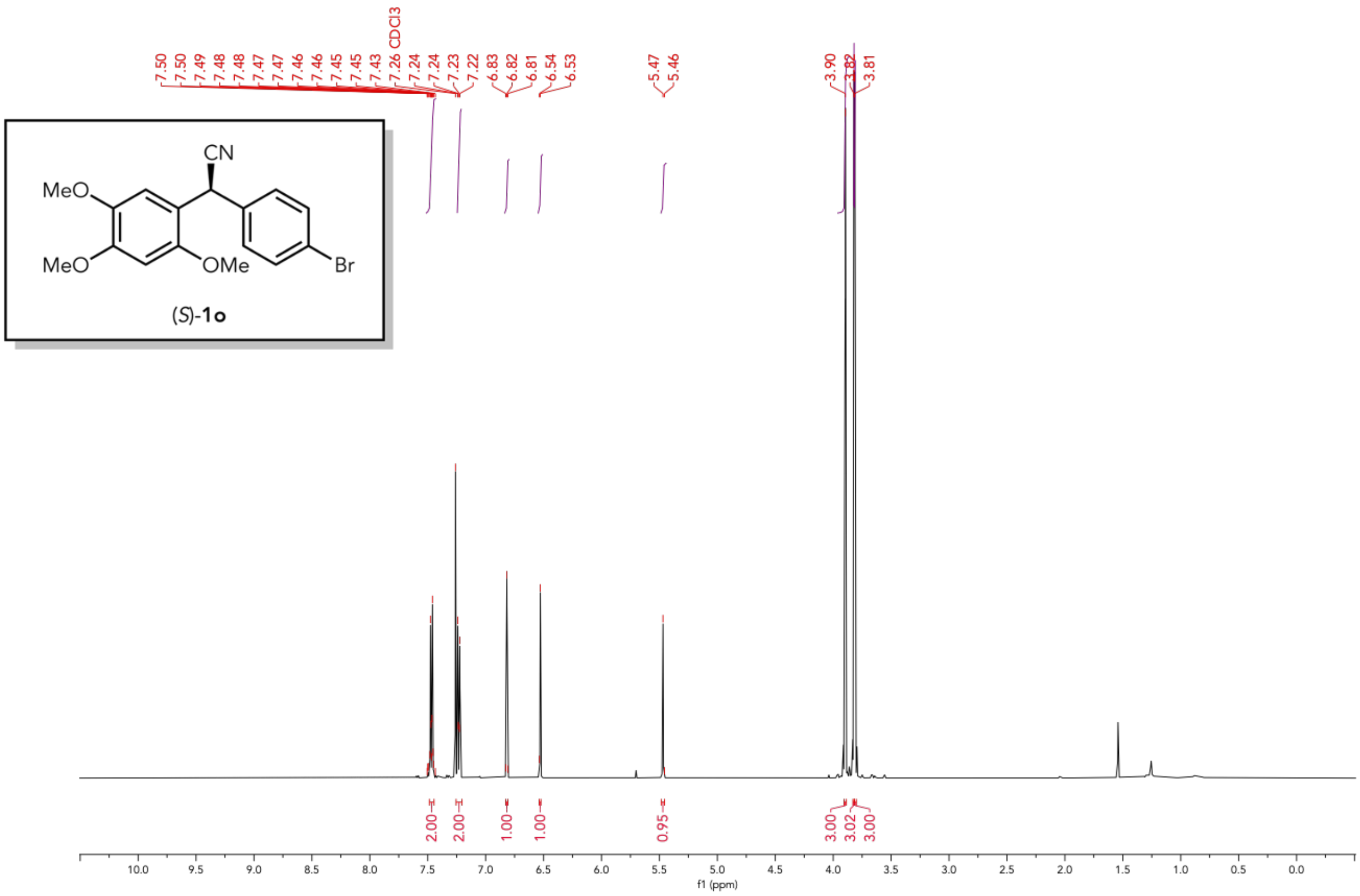

^{1}H NMR (500 MHz, $CDCl_3$) spectrum for compound (*S*)-**1p**

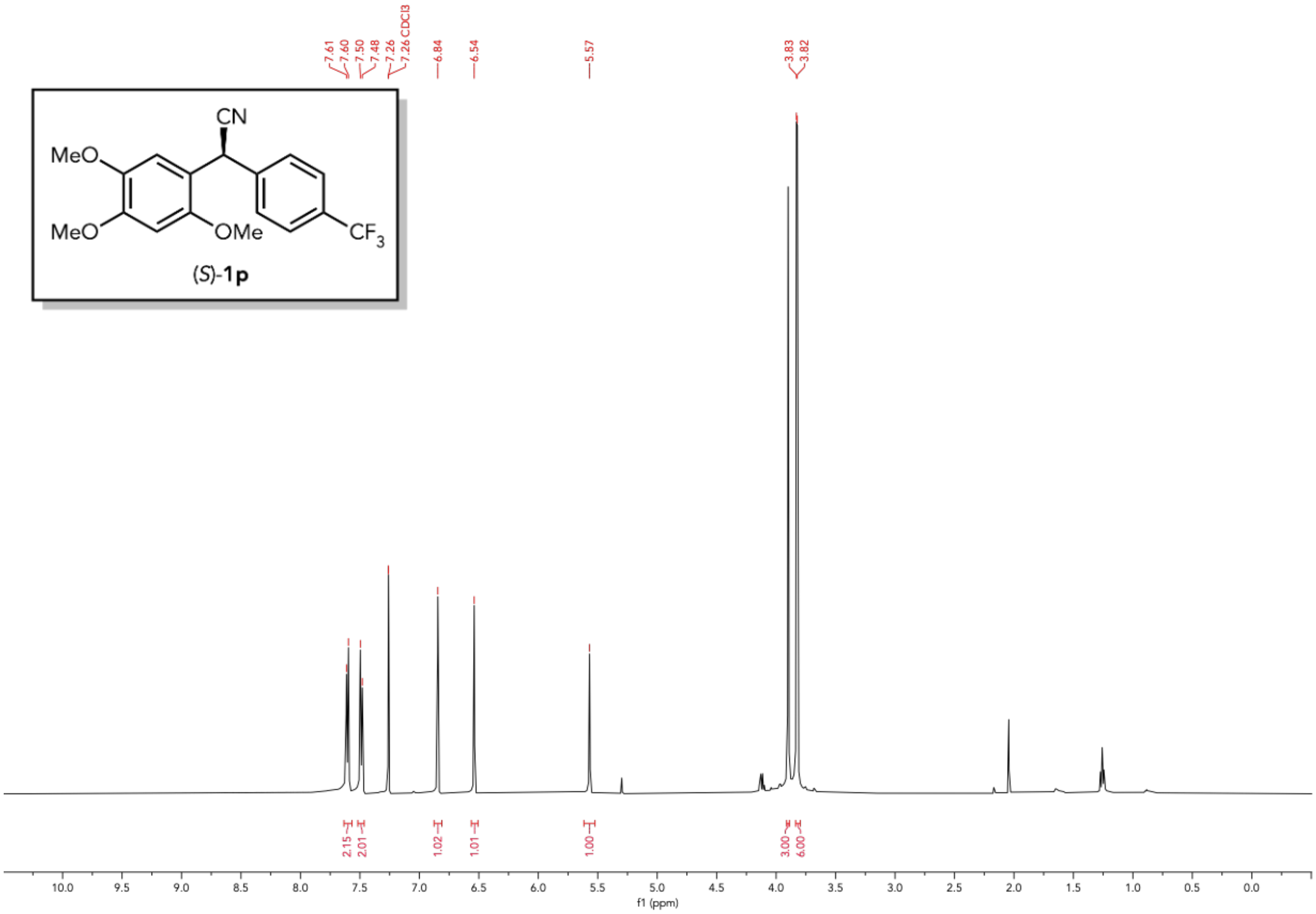

^{1}H NMR (500 MHz, $CDCl_3$) spectrum for compound (*S*)-**1q**

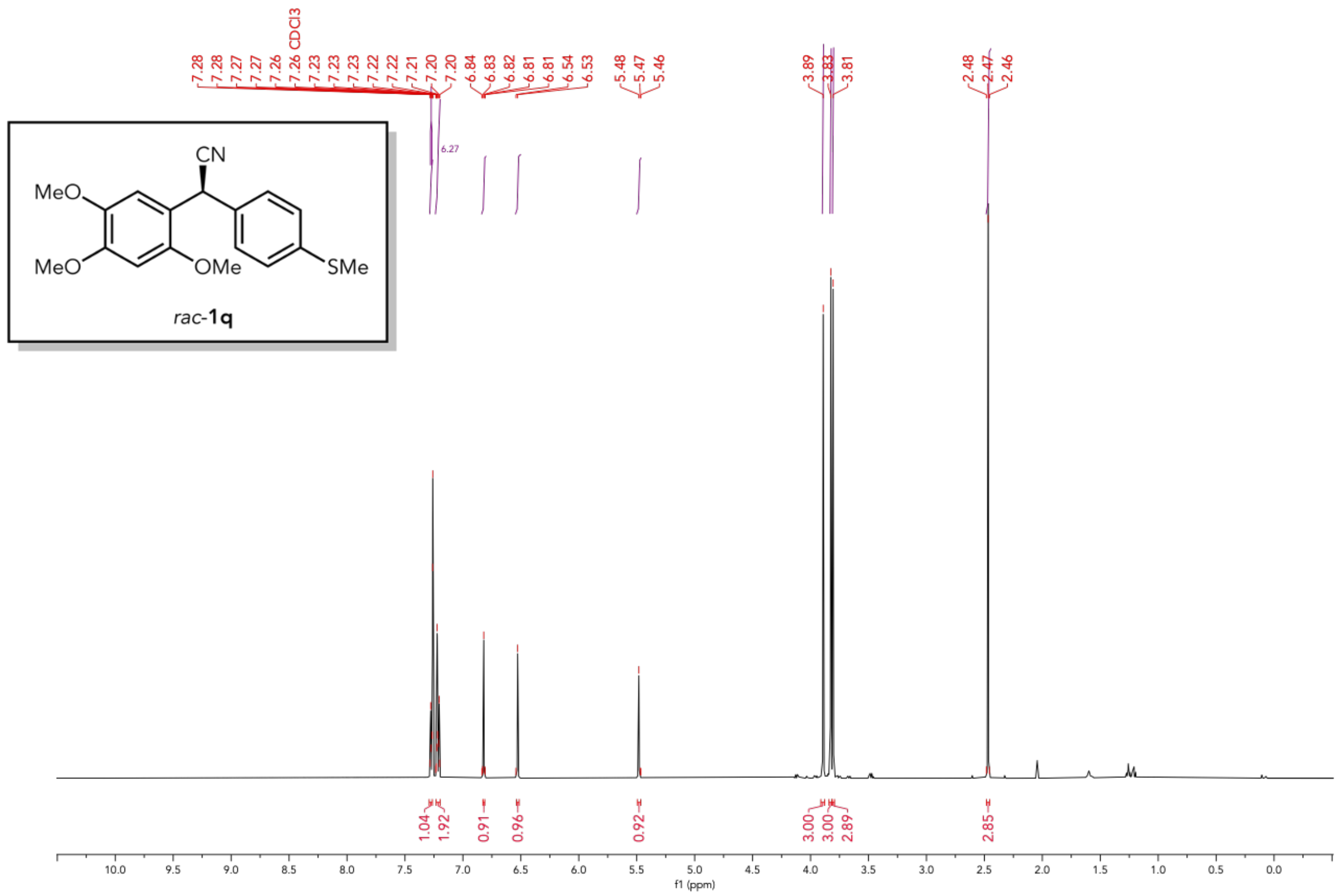

^{1}H NMR (500 MHz, $CDCl_3$) spectrum for compound (*S*)-**1r**

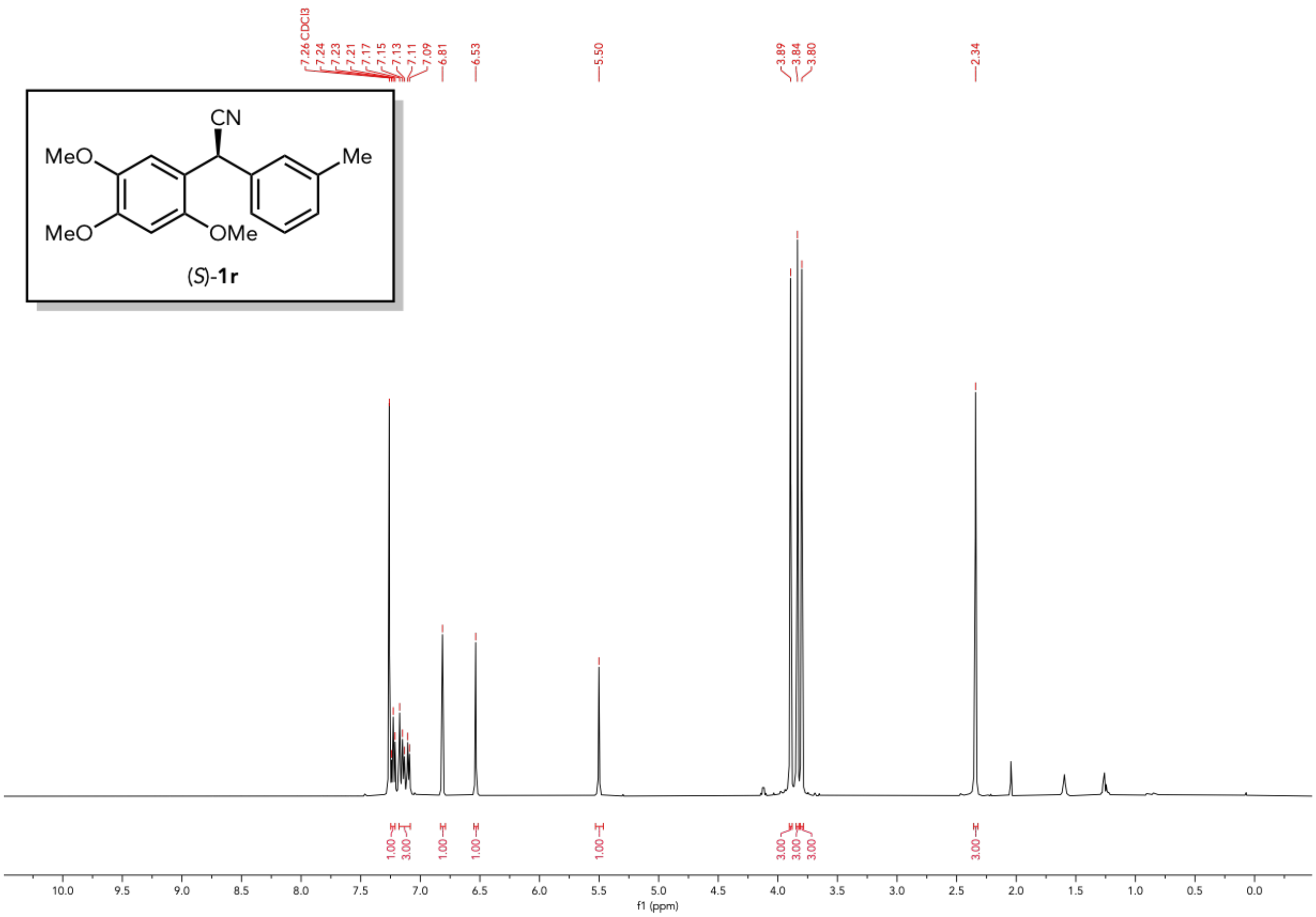

^{1}H NMR (500 MHz, CDCl$_3$) spectrum for compound (*S*)-**1s**

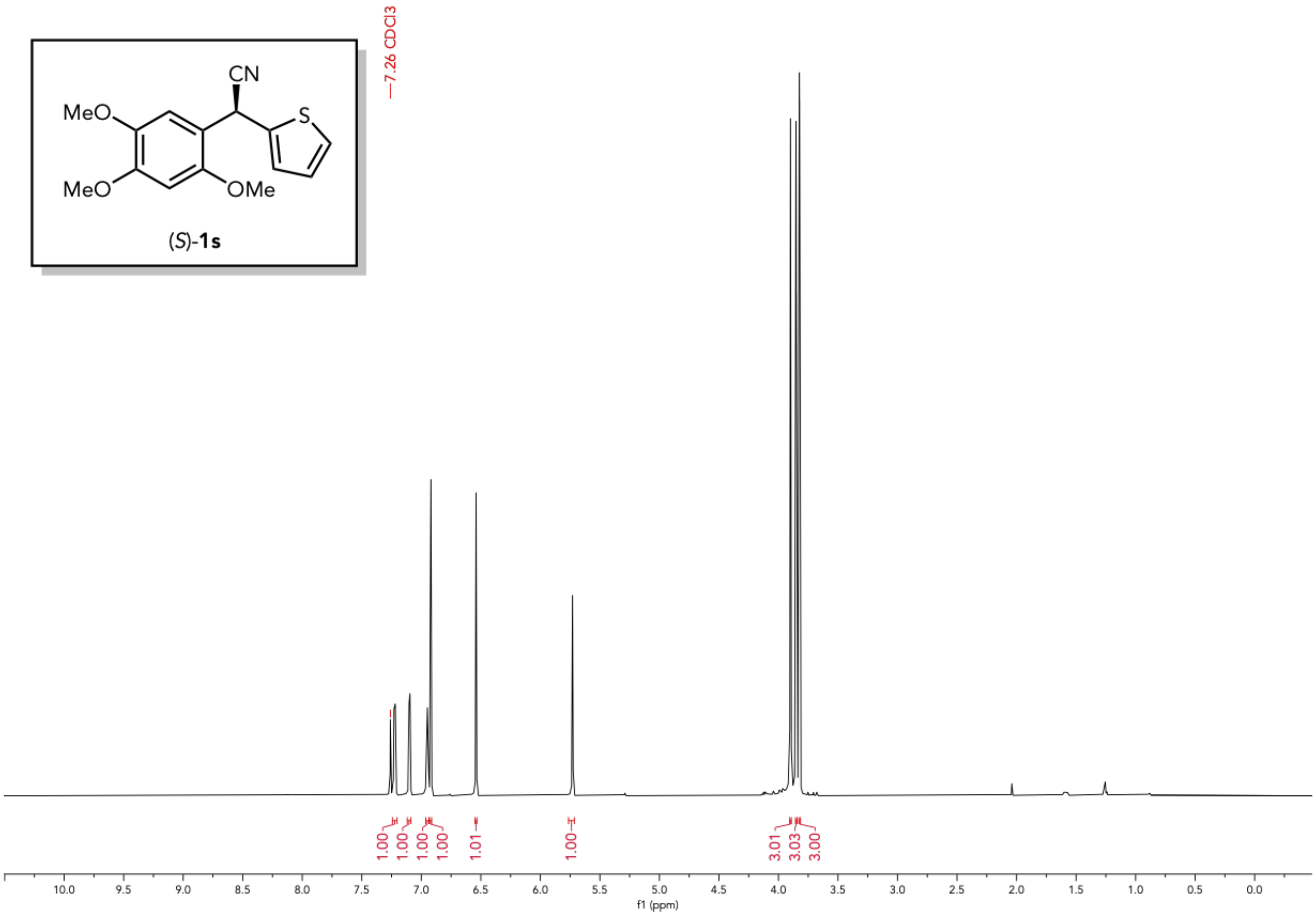

^{1}H NMR (500 MHz, $CDCl_3$) spectrum for compound (*S*)-**1t**

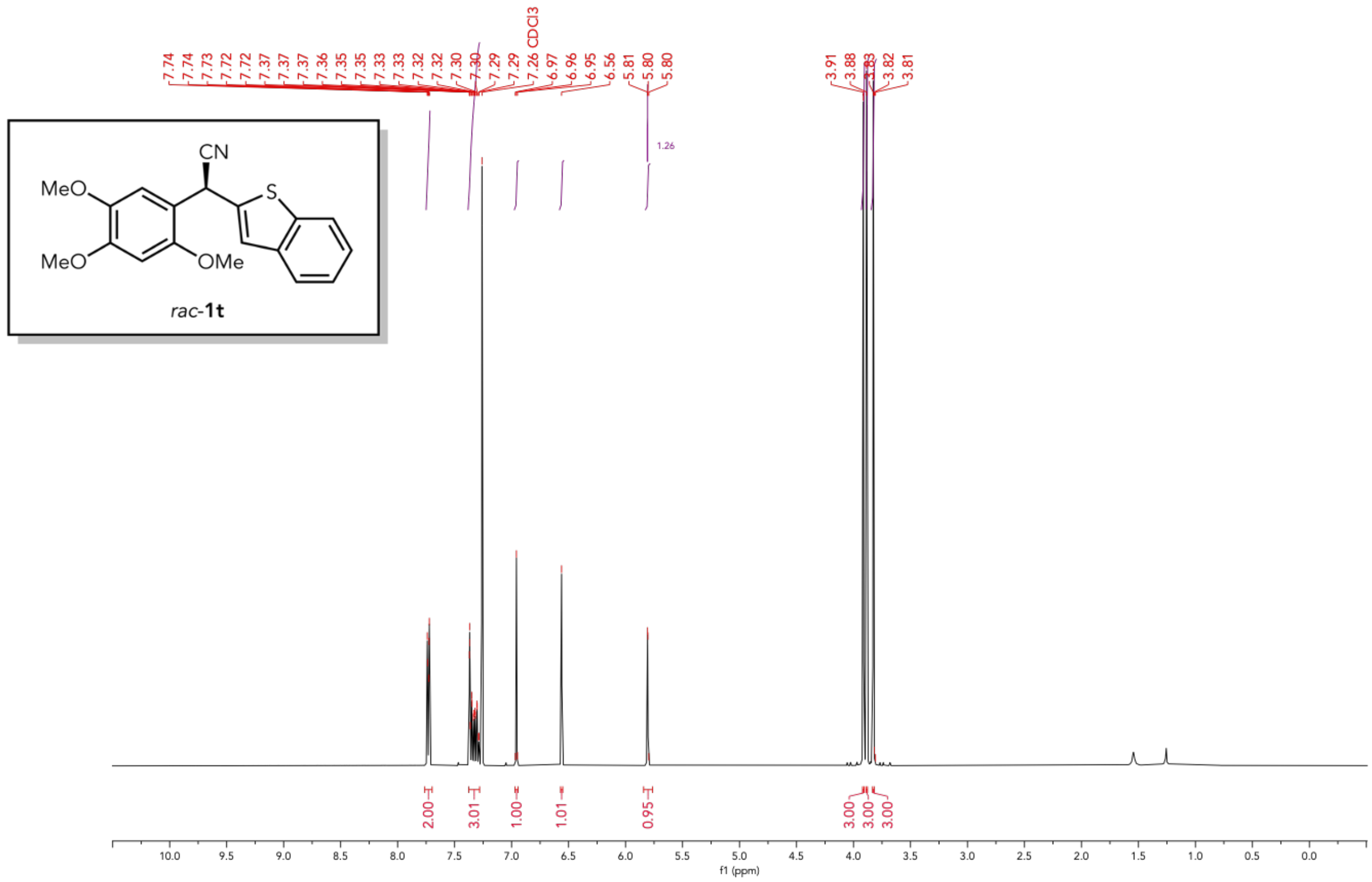

^{1}H NMR (500 MHz, $CDCl_3$) spectrum for compound (*S*)-**1u**

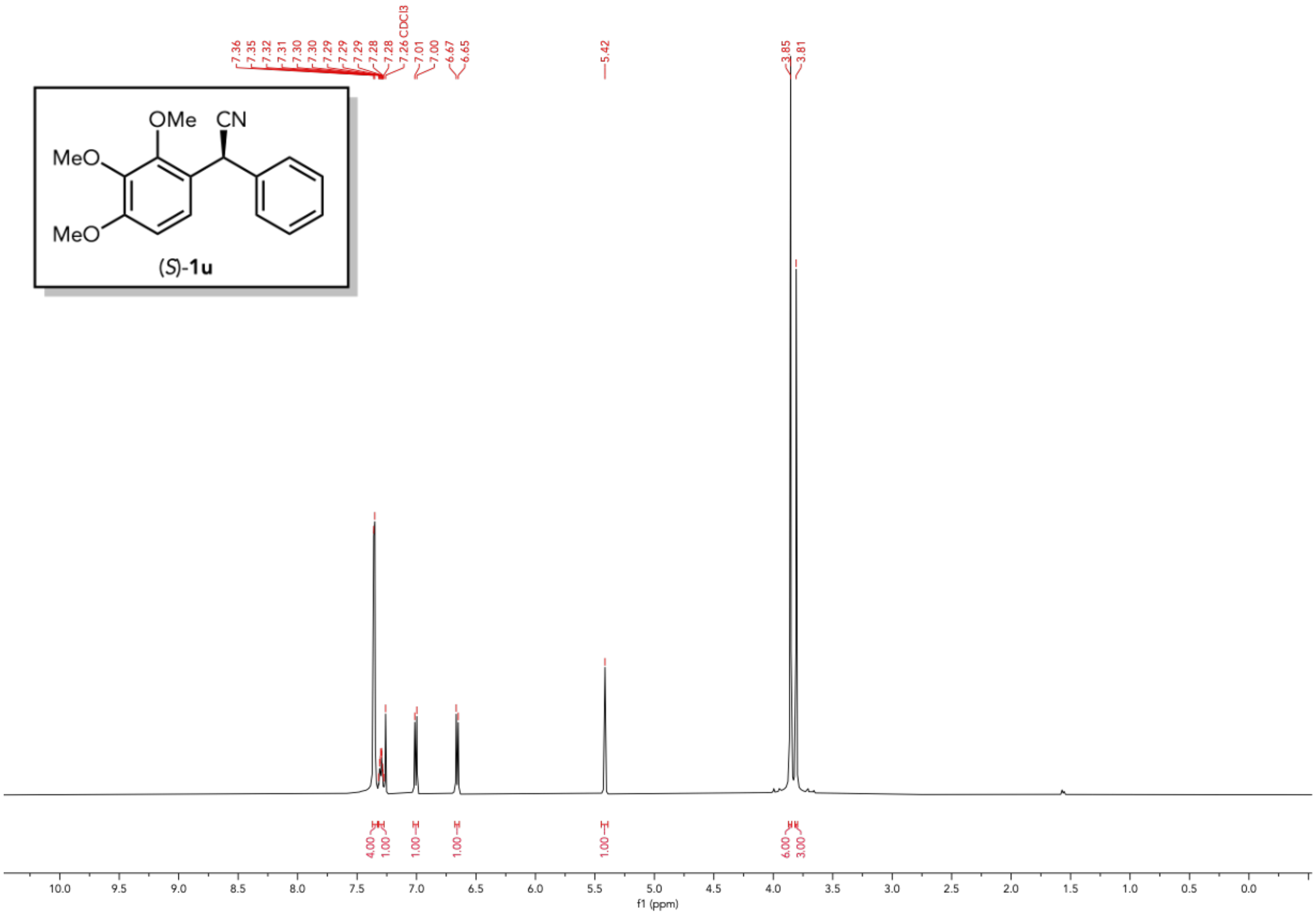

^{1}H NMR (500 MHz, $CDCl_3$) spectrum for compound (*S*)-**1v**

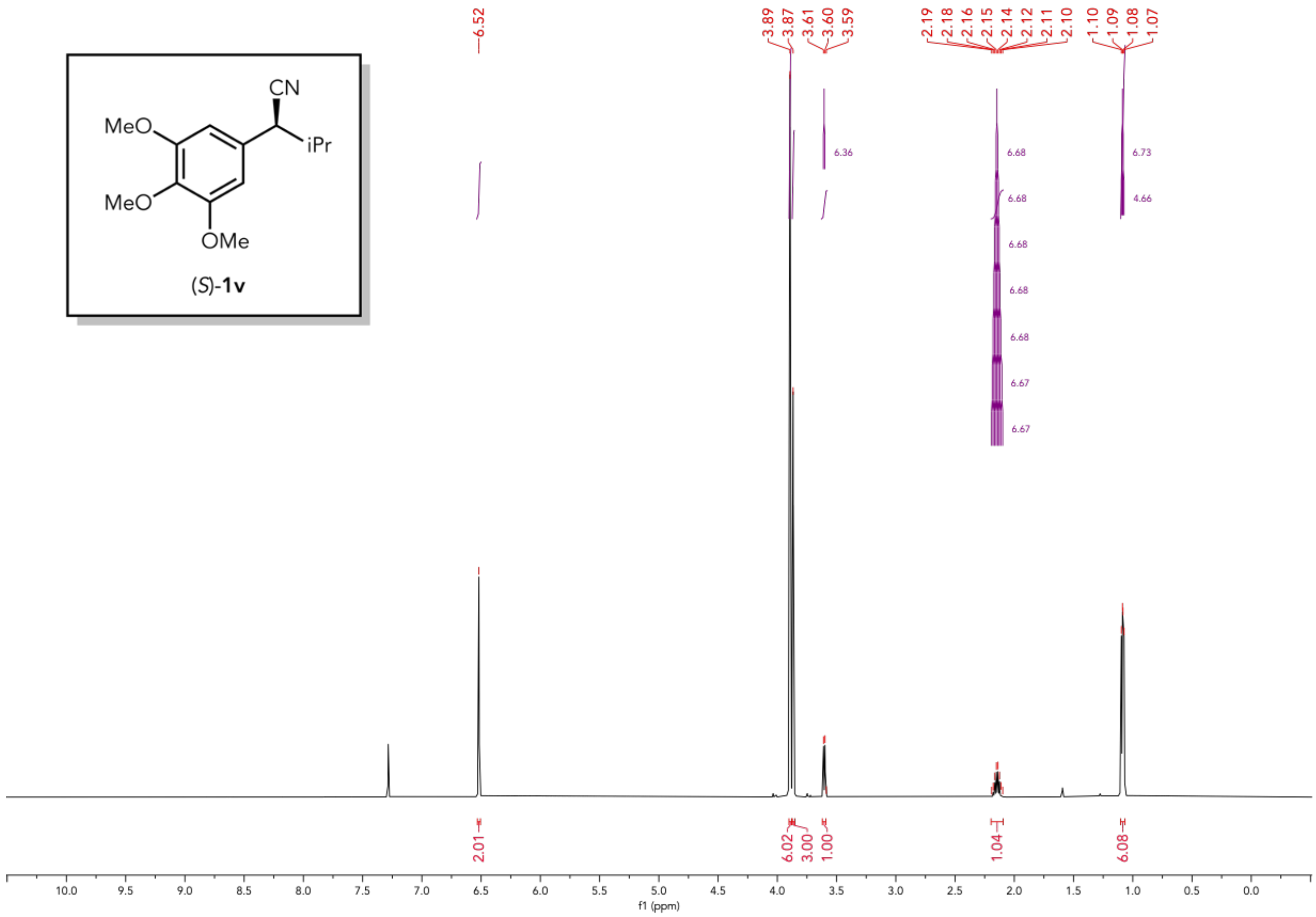

^{1}H NMR (500 MHz, $CDCl_3$) spectrum for compound (*S*)-**1w**

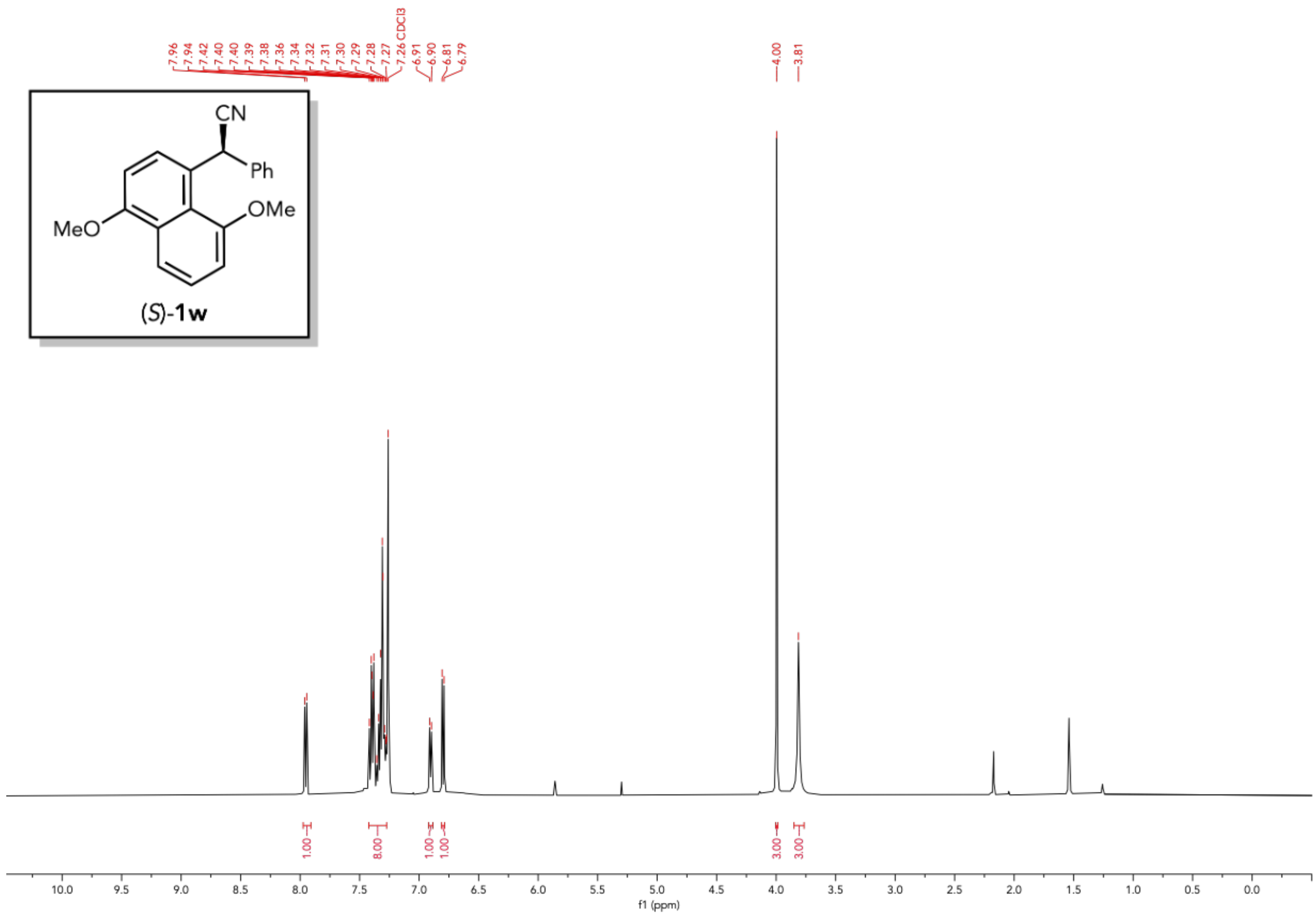

^{1}H NMR (500 MHz, $CDCl_3$) spectrum for compound (*S*)-**1x**

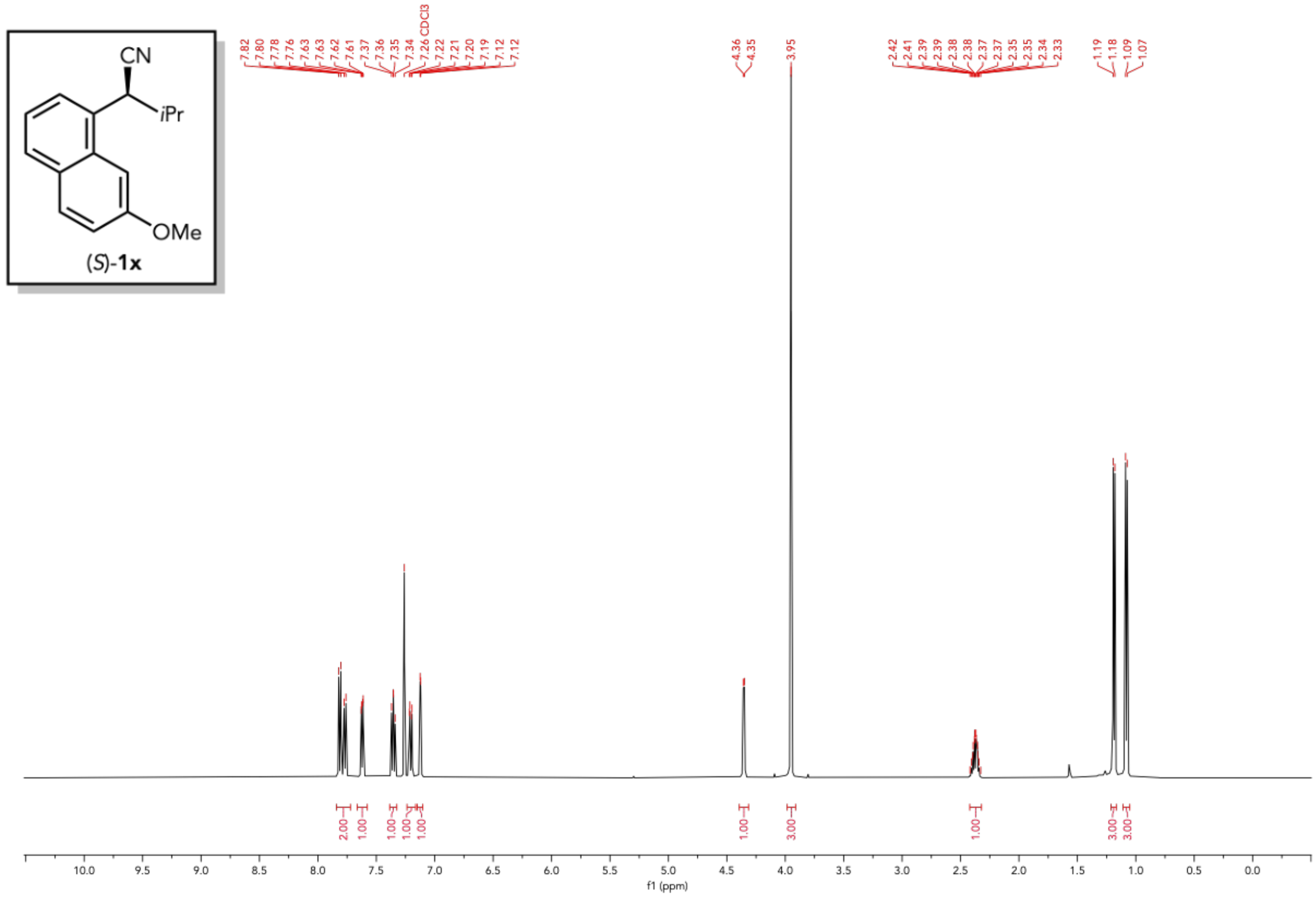

^{1}H NMR (500 MHz, $CDCl_3$) spectrum for compound (*S*)-**1y**

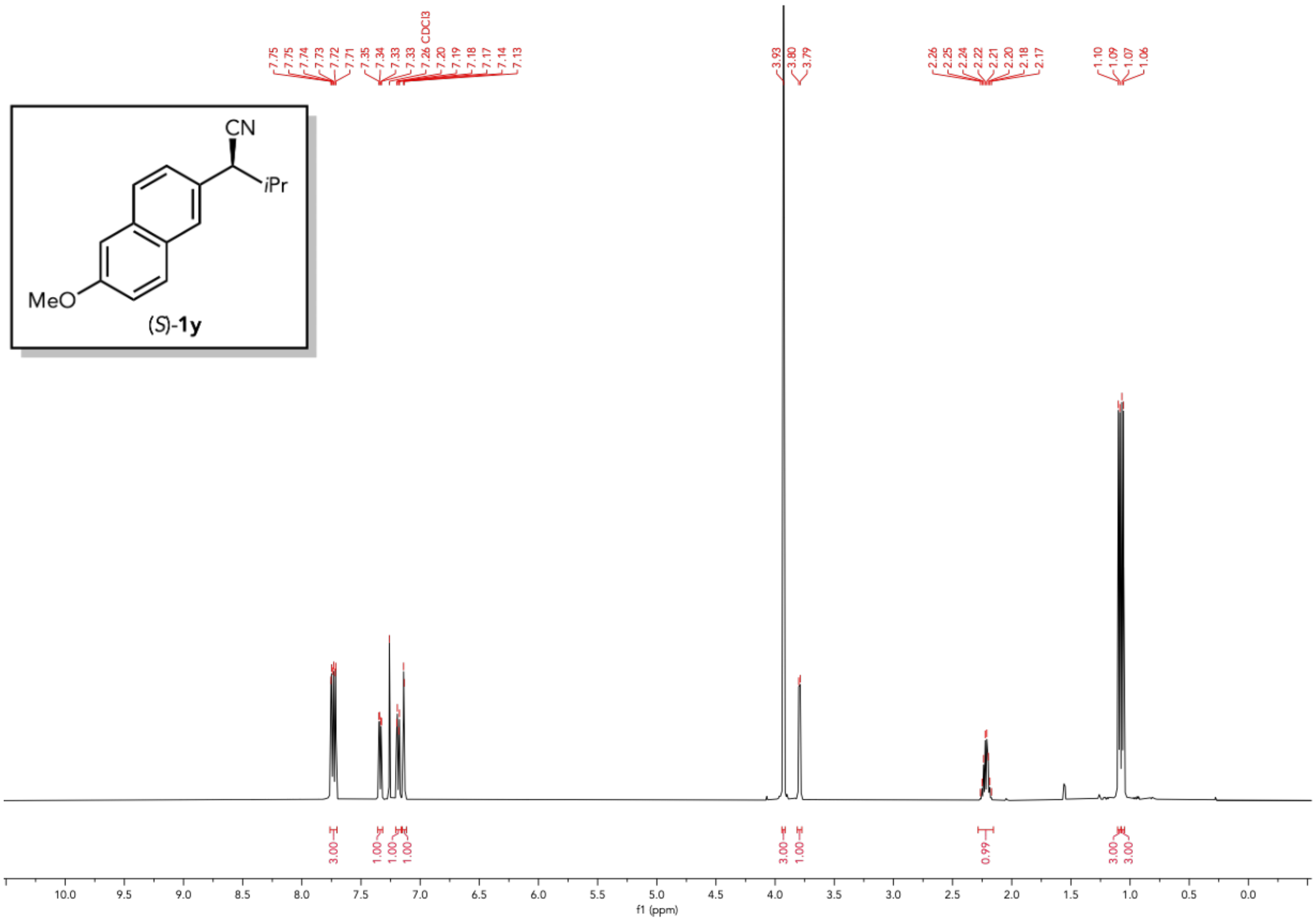

^{1}H NMR (500 MHz, $CDCl_3$) spectrum for compound (*S*)-**1z**

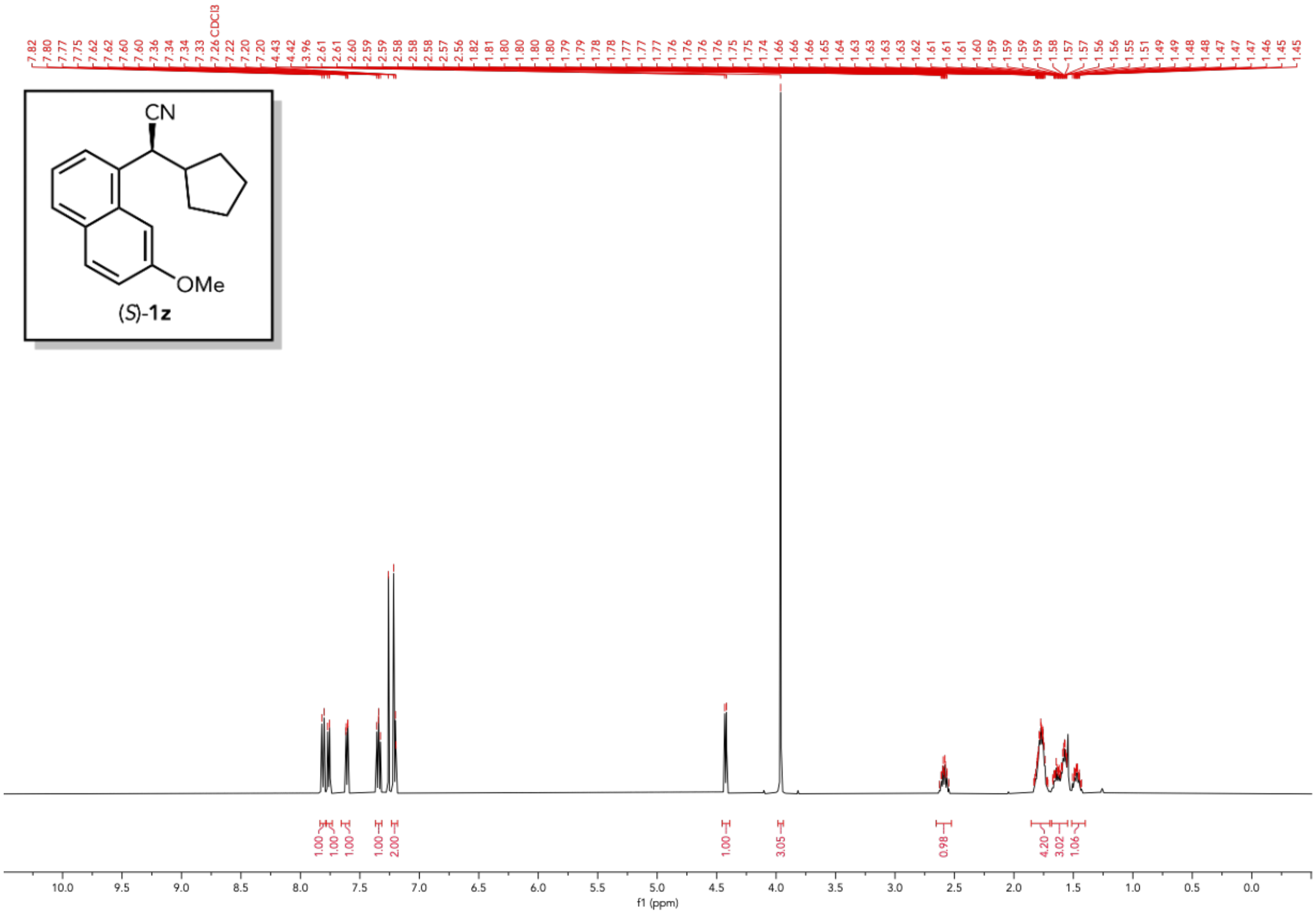

^{1}H NMR (500 MHz, $CDCl_3$) spectrum for compound (*S*)-**1aa**

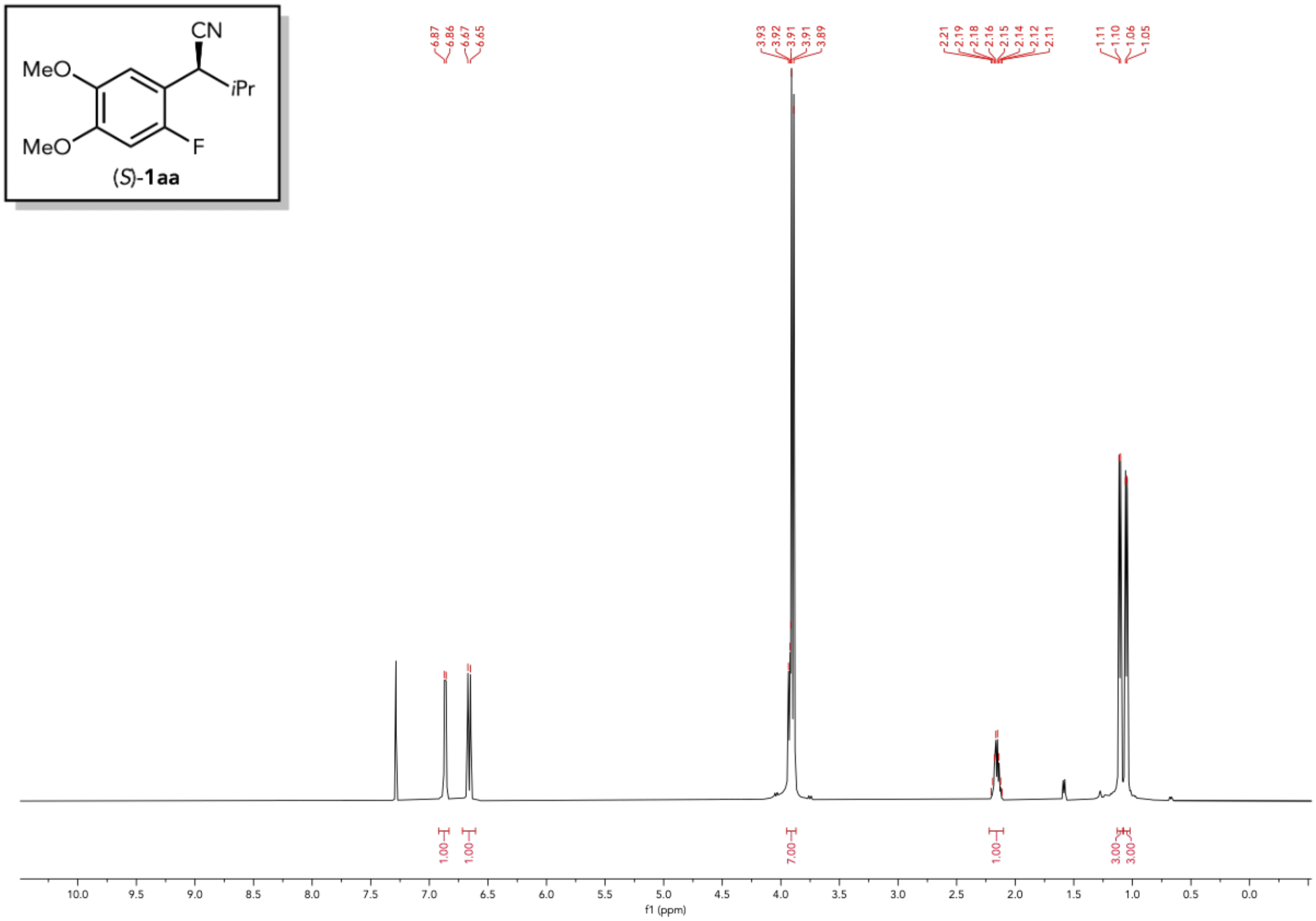

^{1}H NMR (500 MHz, $CDCl_3$) spectrum for compound (*S*)-**1ab**

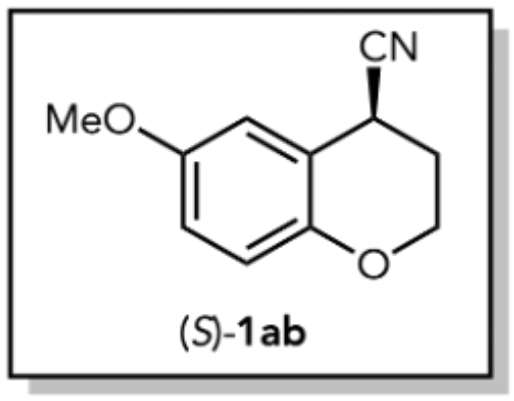

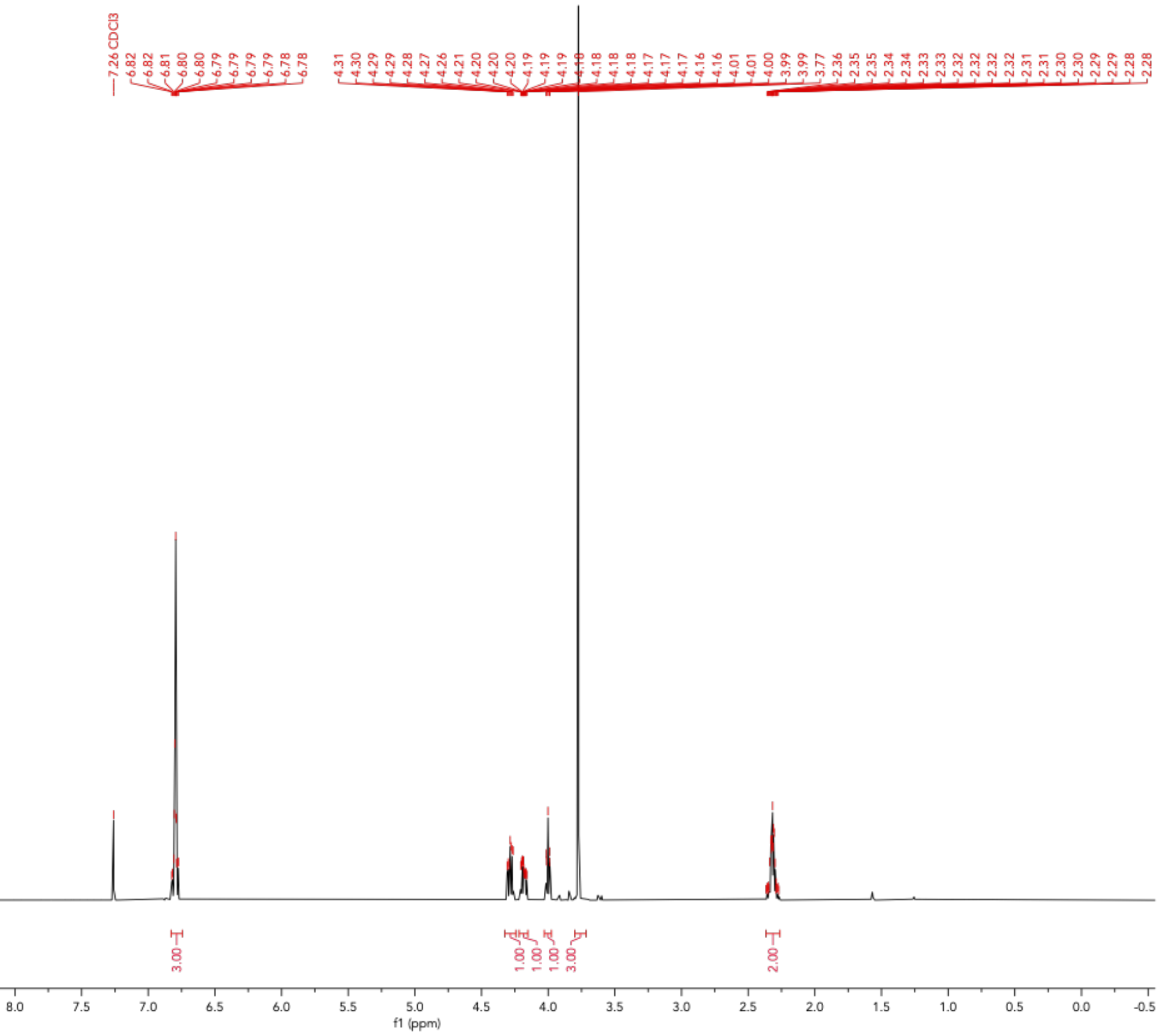

^{1}H NMR (500 MHz, $CDCl_3$) spectrum for compound (*S*)-**1ac**

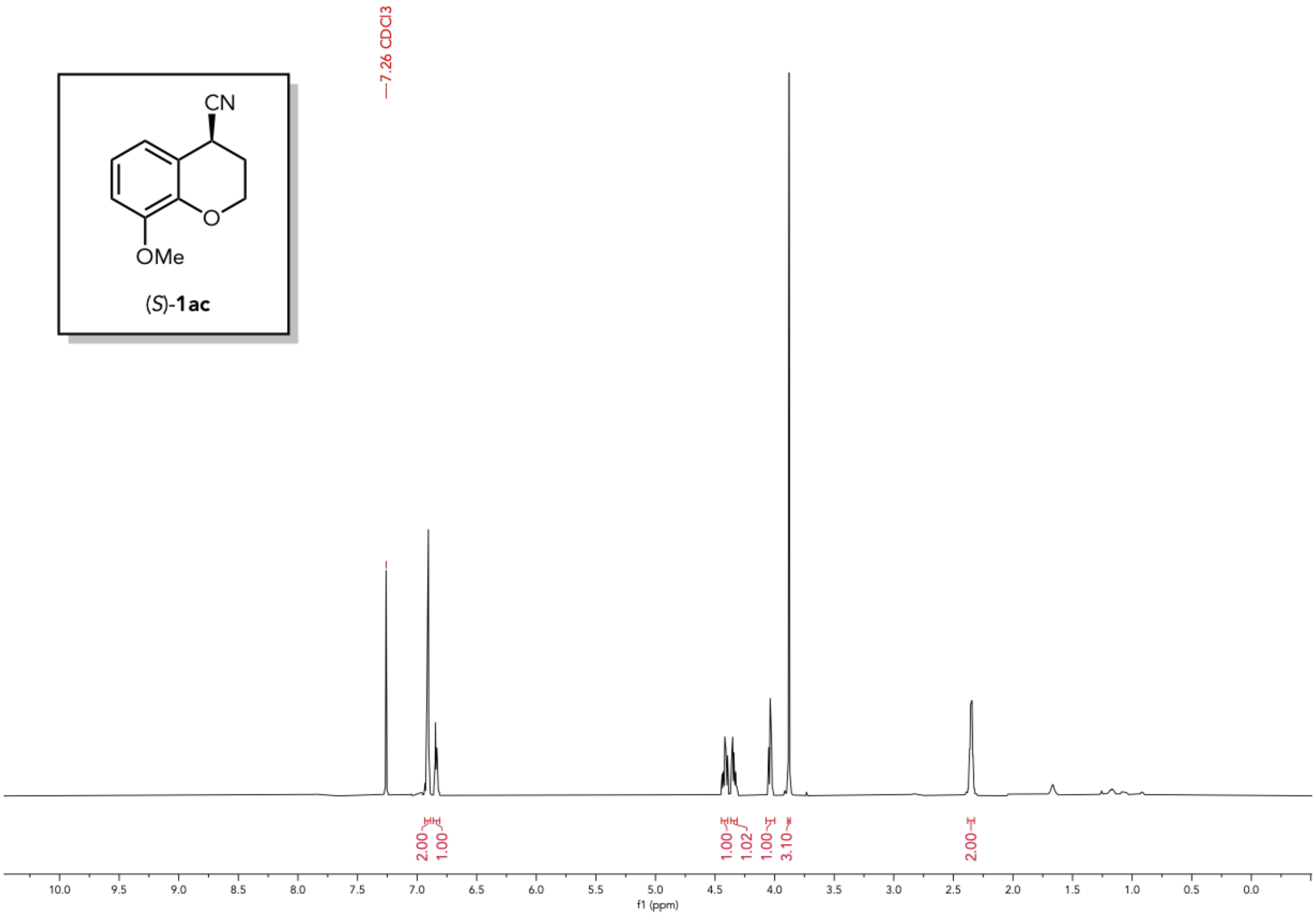

^{1}H NMR (500 MHz, $CDCl_3$) and ^{13}C NMR (126 MHz, $CDCl_3$) spectra for compound (2*S*)-**1ad**

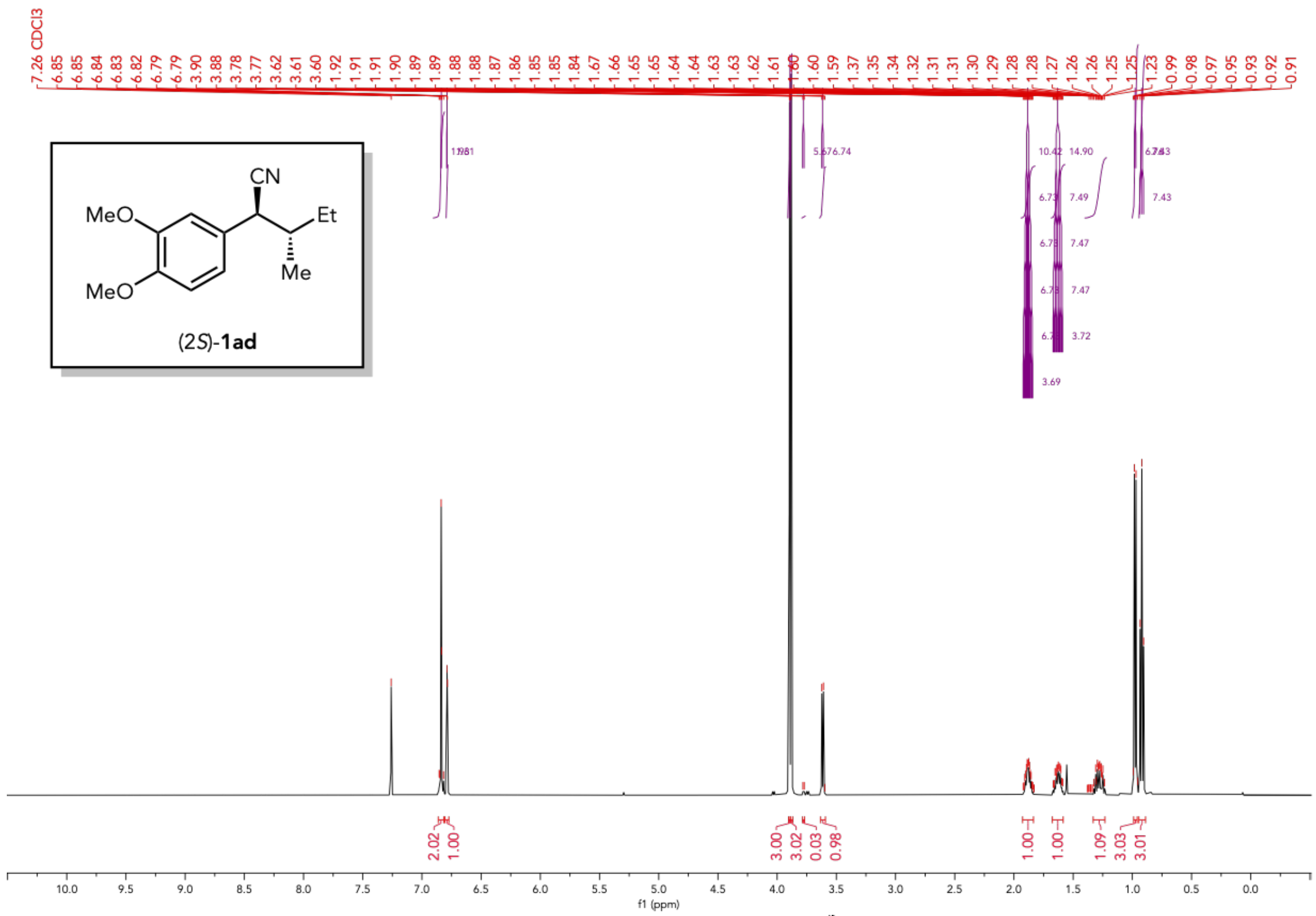

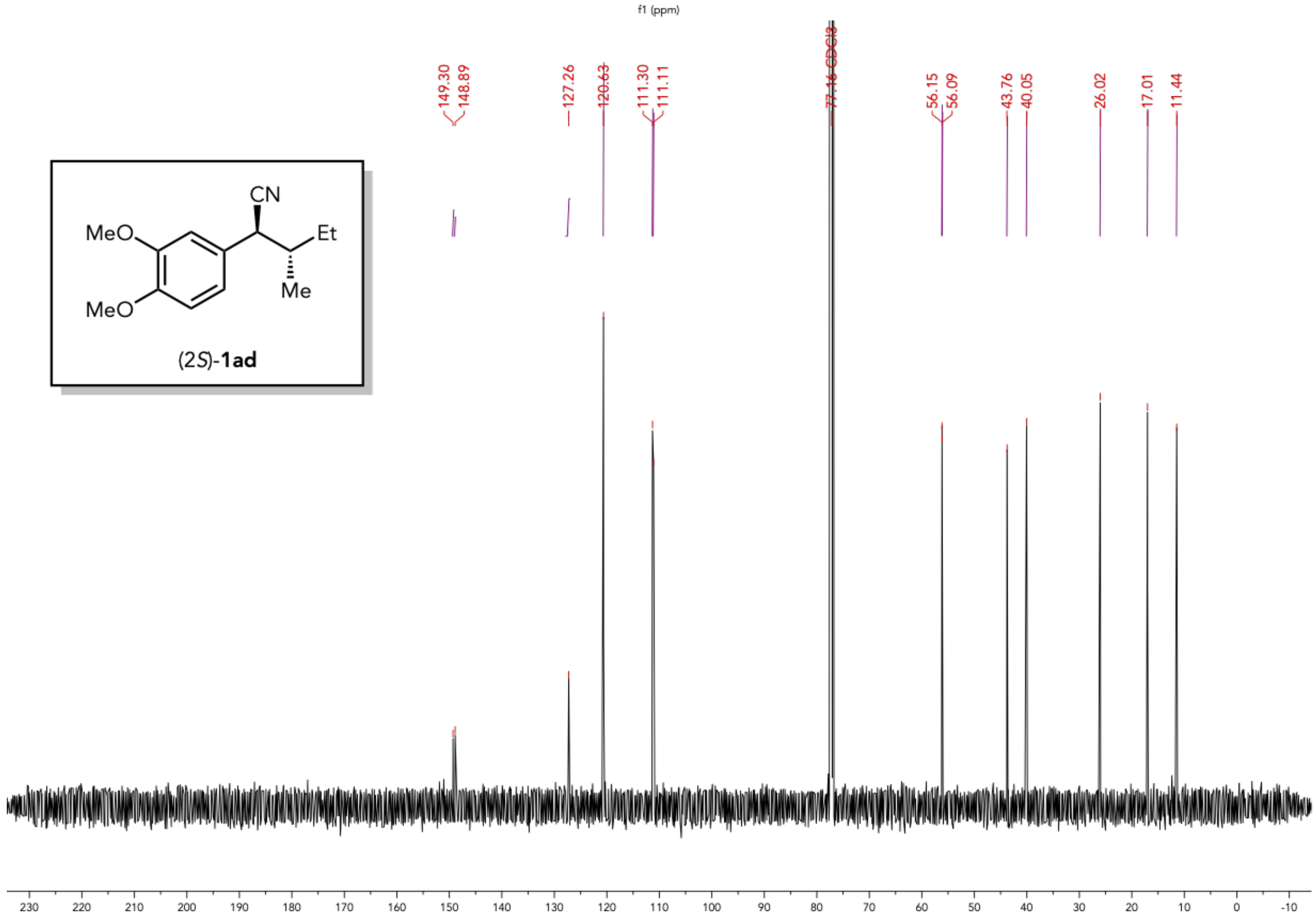

^{1}H NMR (500 MHz, $CDCl_3$) and ^{13}C NMR (126 MHz, $CDCl_3$) spectra for compound (2*R*)-**1ad**

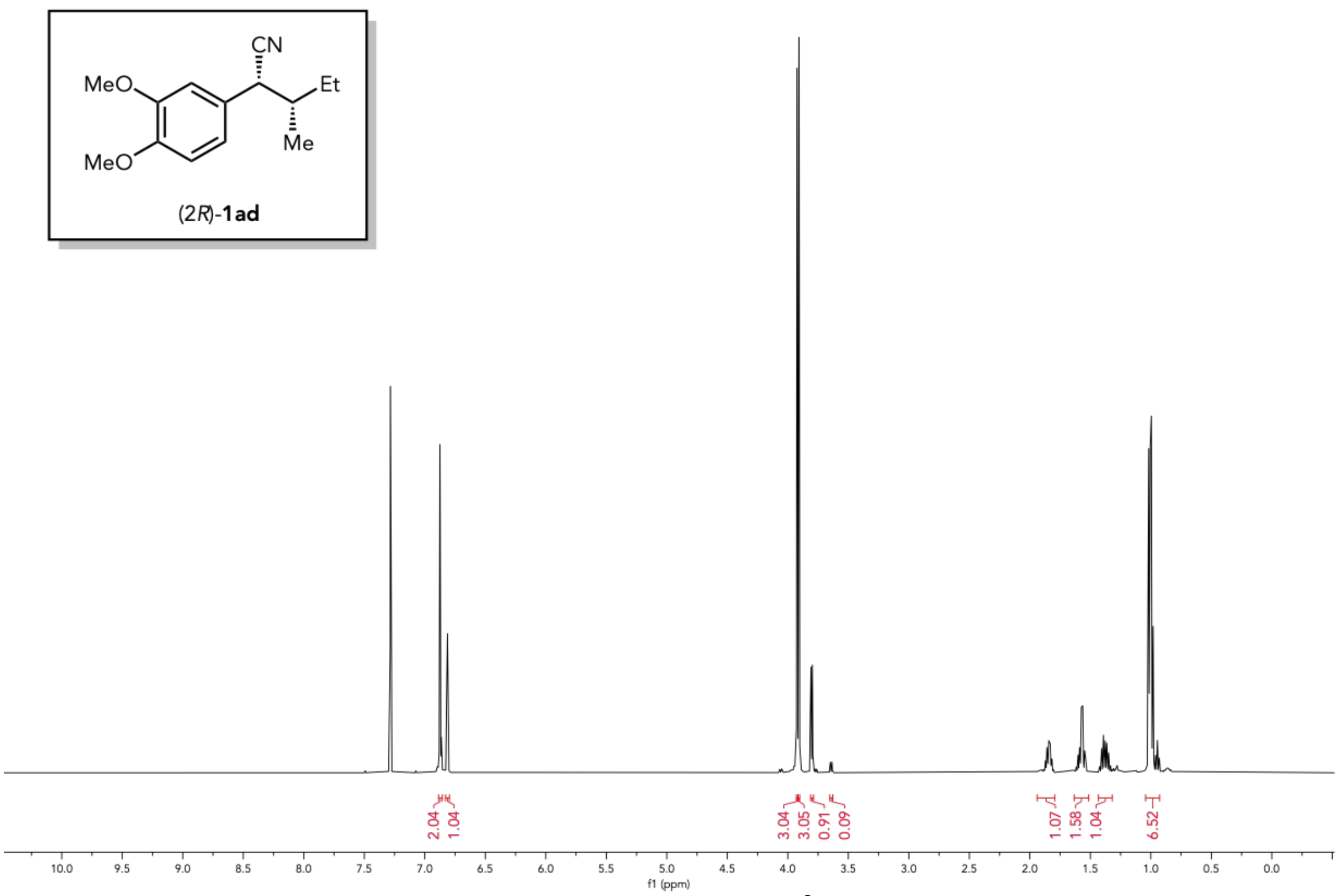

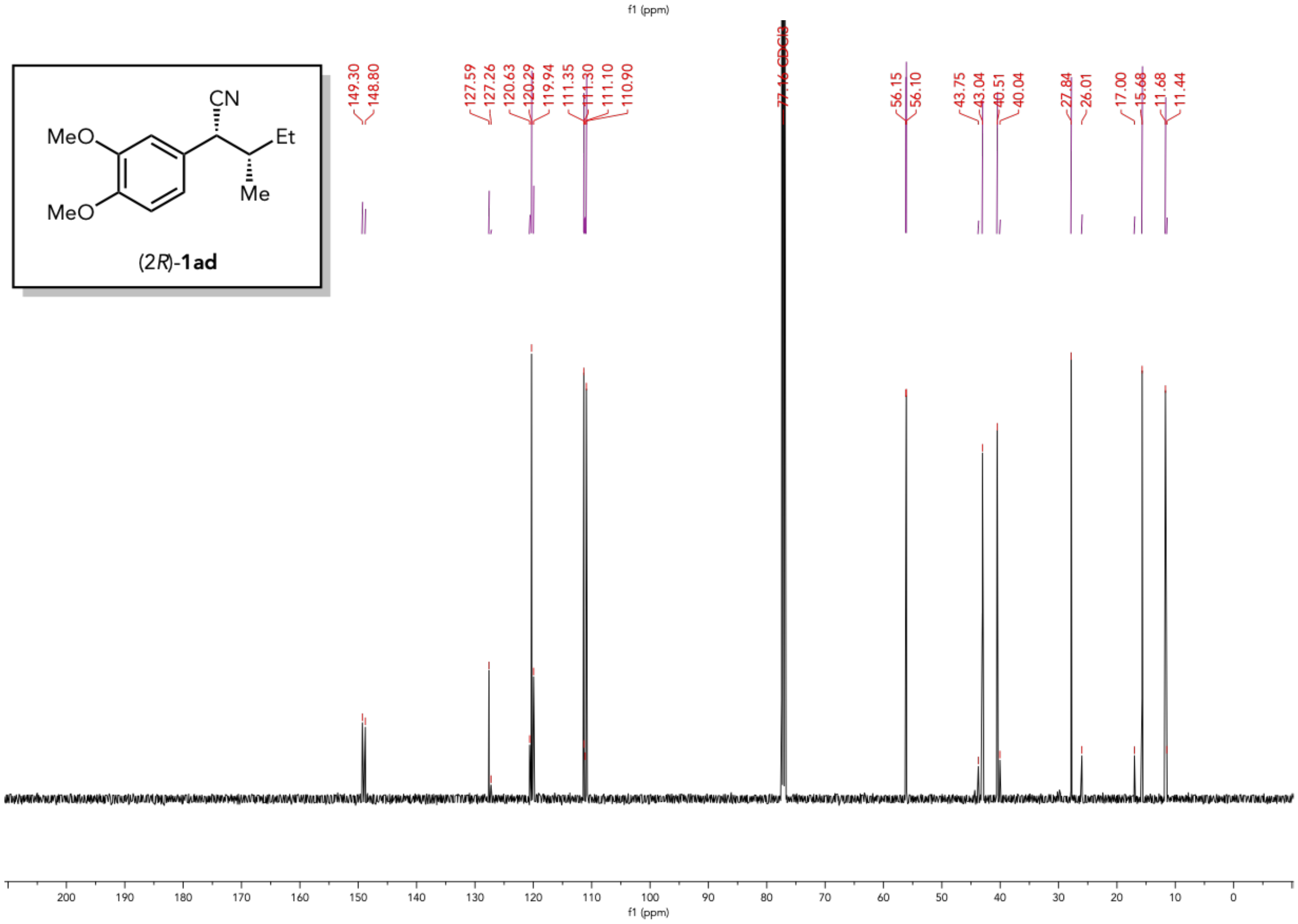

^{1}H NMR (500 MHz, $CDCl_3$) spectrum for compound (*S*)-**1ae**

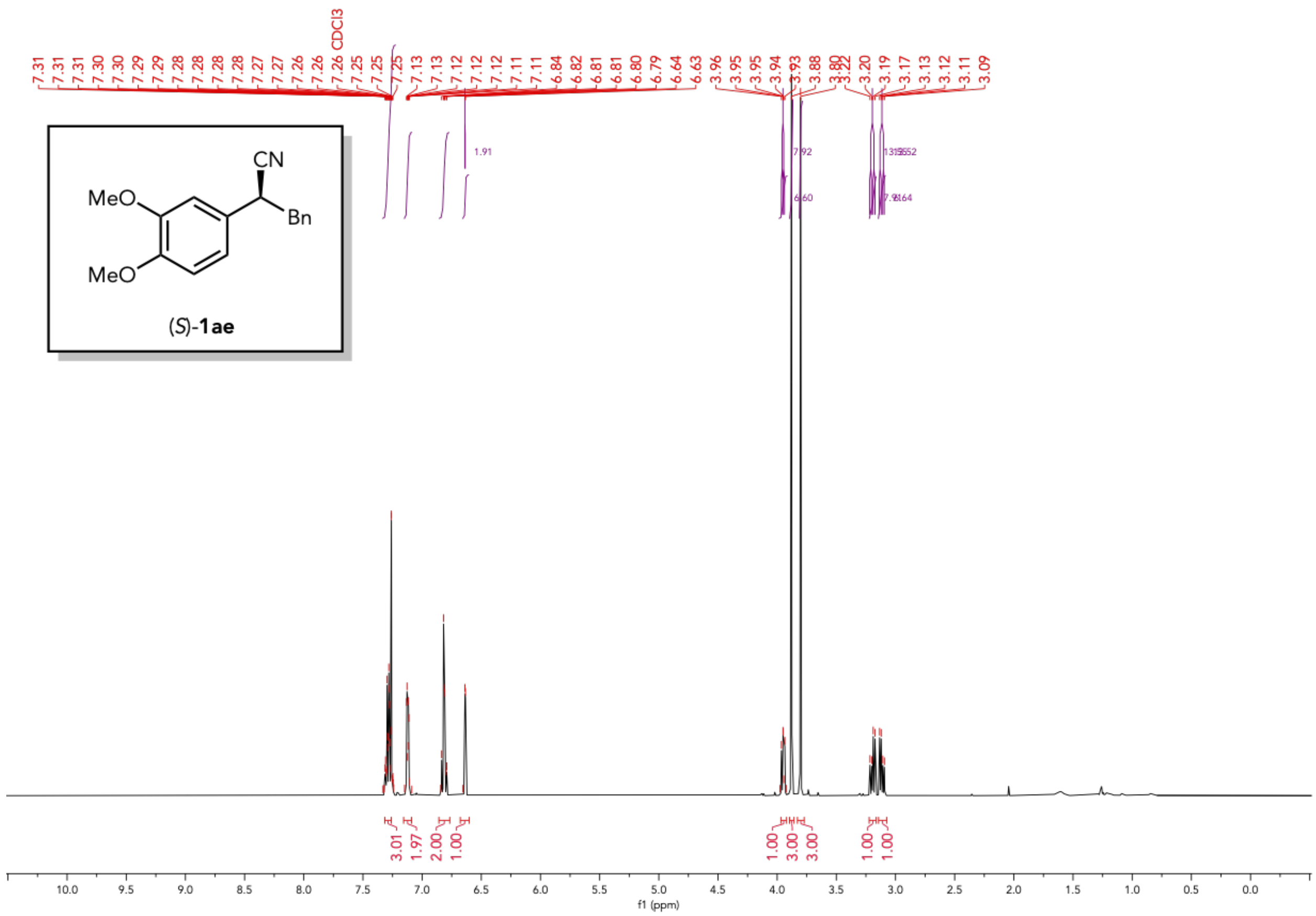

^{1}H NMR (500 MHz, $CDCl_3$) and ^{13}C NMR (126 MHz, $CDCl_3$) spectra for compound (*S*)-**1ae-*d₁***

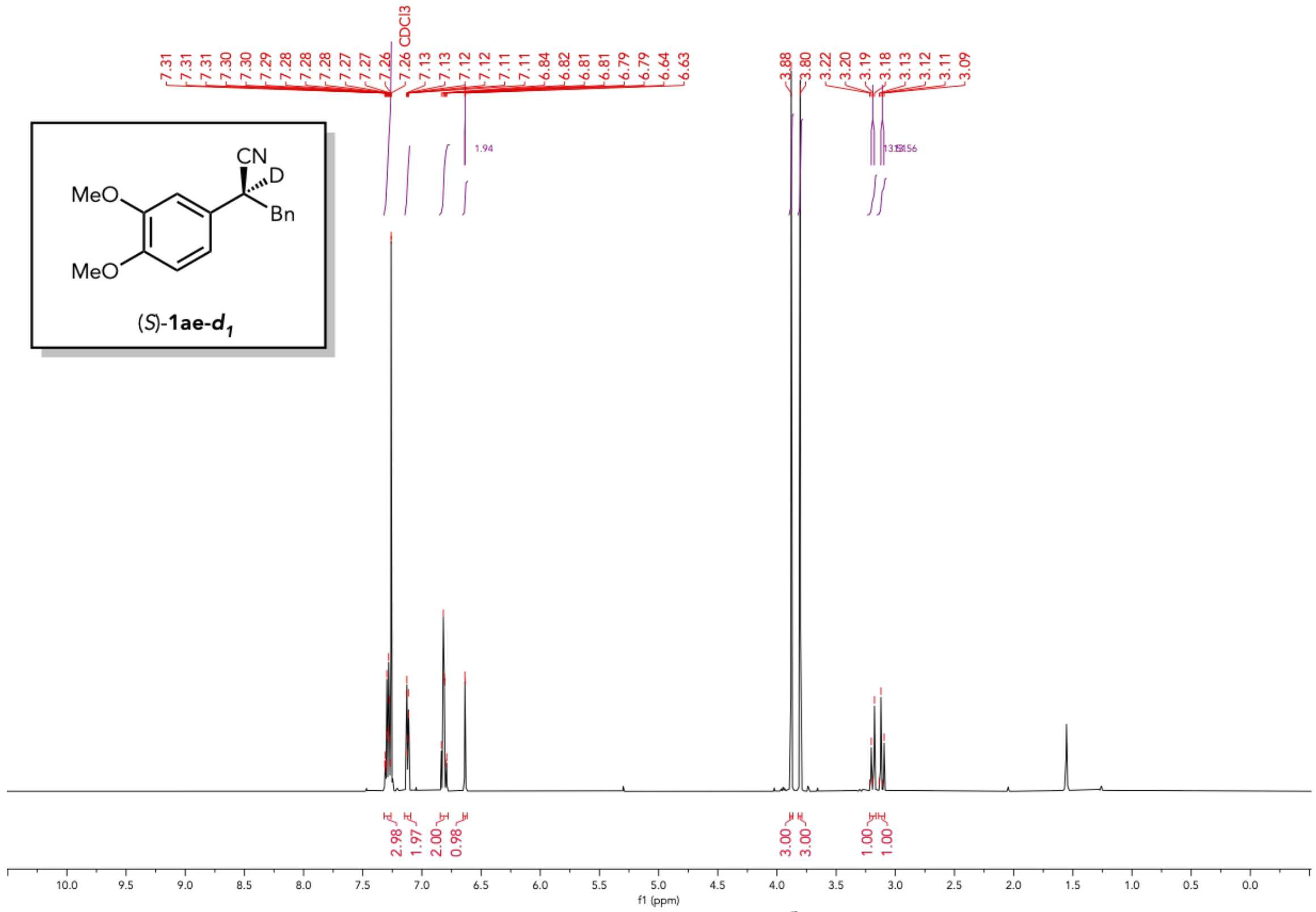

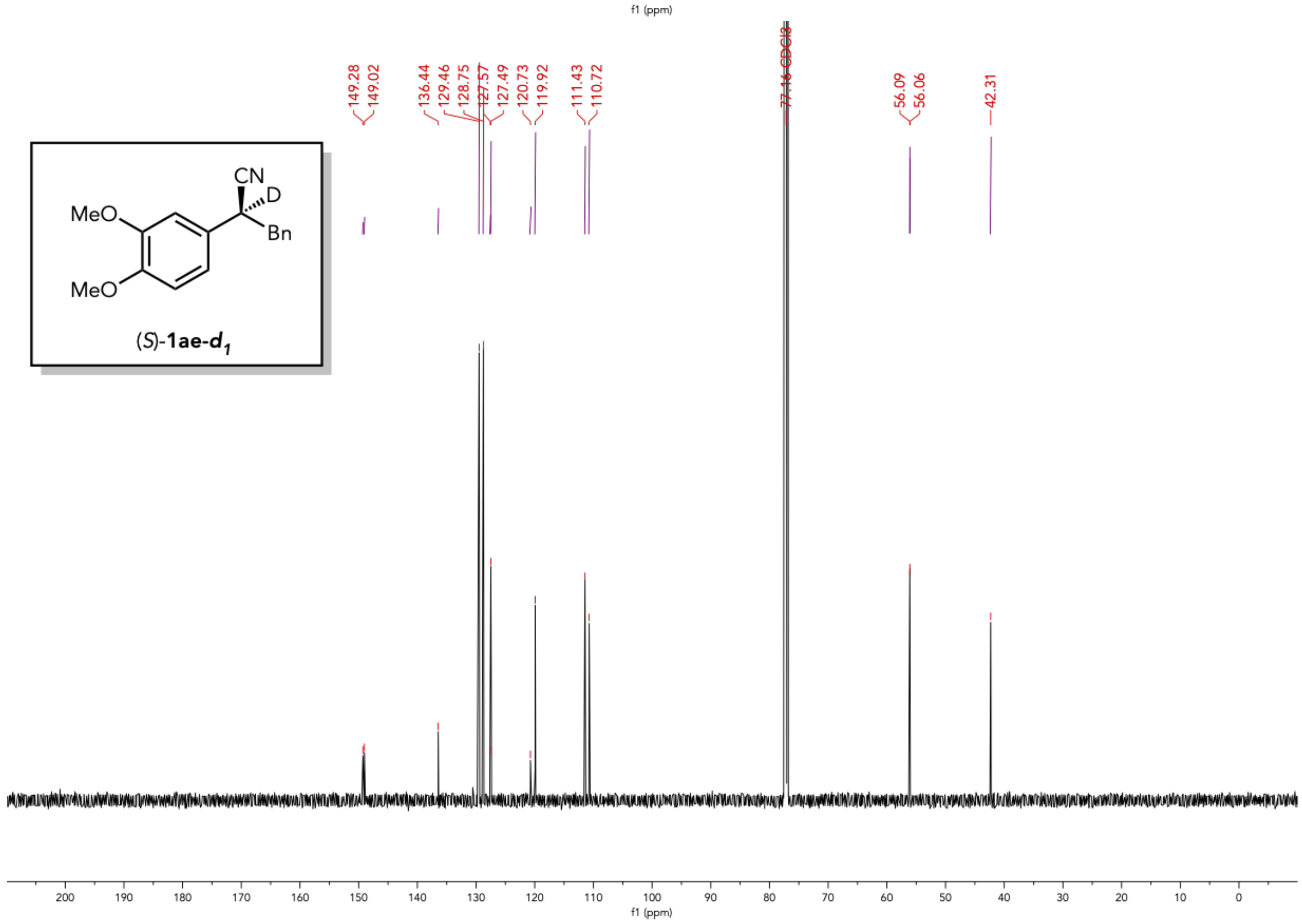

^1H NMR (500 MHz, $CDCl_3$) and ^{13}C NMR (126 MHz, $CDCl_3$) spectra for compound (*R*)-**6**

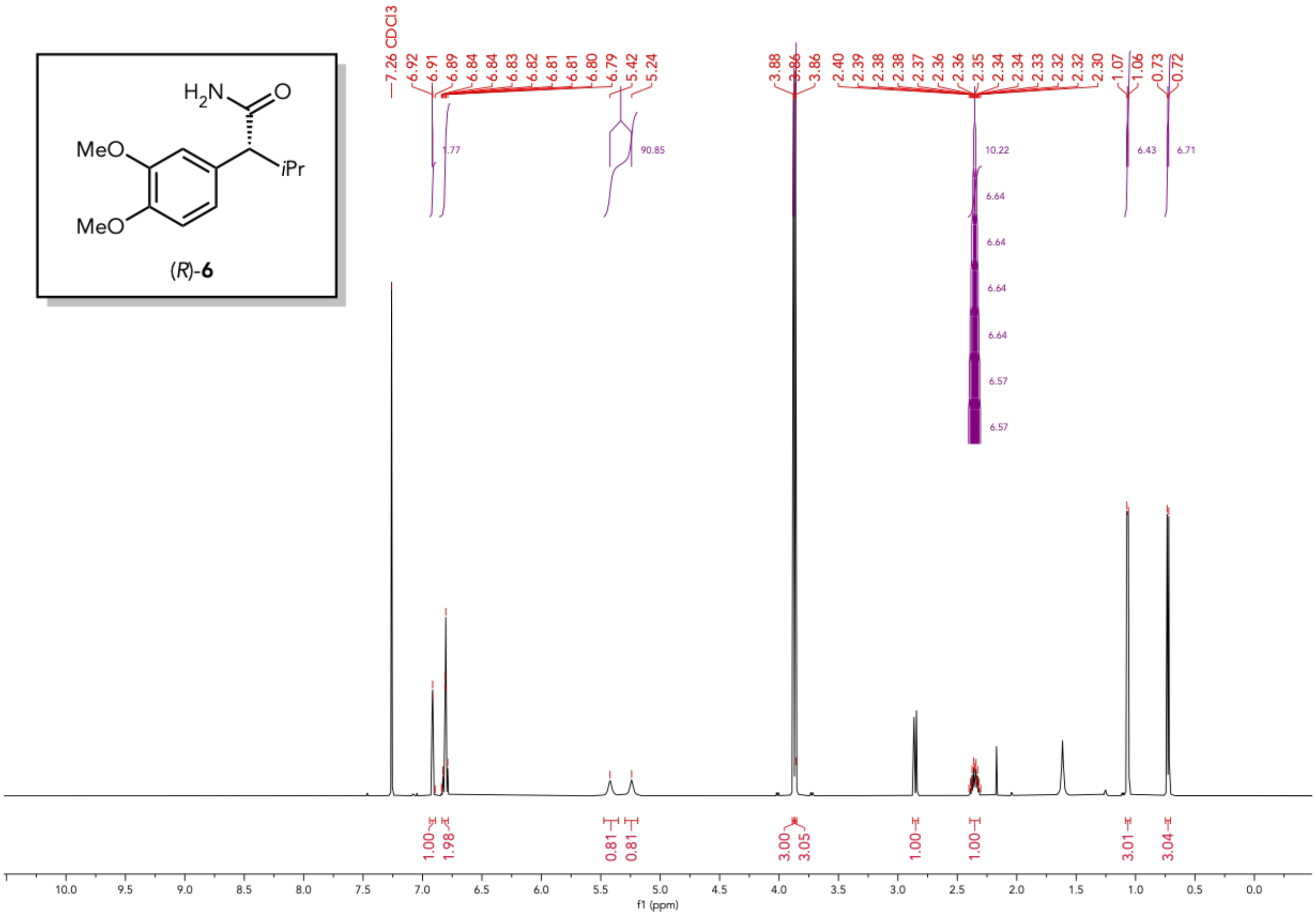

21. References

1. Pangborn, A. B.; Giardello, M. A.; Grubbs, R. H.; Rosen, R. K.; Timmers, F. J. Safe and Convenient Procedure for Solvent Purification. Organometallics 1996, 15, 1518–1520.
2. Still, W. C.; Kahn, M.; Mitra, A. Rapid chromatographic technique for preparative separations with moderate resolution. *J. Org. Chem*. **1978**, *43*, 2923–2925.
3. Harris, R. K.; Becker, E. D.; d. Menezes, S. M. C.; Granger, P.; Hoffman, R. E.; Zilm, K. W. Further conventions for NMR shielding and chemical shifts (IUPAC Recommendations 2008). *Pure Appl. Chem.* **2008**, *80*, 59–84.
4. Geunes, E.P.; Meinhardt, J.M.; Wu, E.J.; Knowles, R.R. Photocatalytic Anti-Markovnikov Hydroamination of Alkenes with Primary Heteroaryl Amines. *J. Am. Chem. Soc.* **2023**, *145*, 21738–21744.
5. Mamidala, R; Kommuri, C.; Paulose, J.; Aswath, H.; Pawar, L.; Arunachalampillai, A.; Cherney, A. H.; Tedrow, J. S.; Rötheli, A. R.; Ortiz, A.; Convenient, Large-Scale Synthesis of (*ß*)-TRIP Using Suzuki Cross-Coupling Conditions. *Org. Process Res. Dev*. **2022**, *26*, 165–173.
6. Wang, J.Y.; Villalona, E.; Knowles, R.R. Photocatalyst-Dependent Enantioselectivity in the Light-Driven Deracemization of Cyclic *α*-Aryl Ketones. *J. Am. Chem. Soc.* **2025**, *147*, 15307–15317.
7. Chen, G.; Wang, Z.; Wu, J.; Ding, K. Facile Preparation of *α*-Aryl Nitriles by Direct Cyanation of Alcohols with TMSCN Under the Catalysis of InX_3. *Org. Lett.* **2008**, *10*, 4573–4576.
8. Wu, G.; Deng, Y.; Wu, C.; Zhang, Y.; Wang, J. Synthesis of *α*-Aryl Esters and Nitriles: Deaminative Coupling of *α*-Aminoesters and *α*-Aminoacetonitriles with Arylboronic Acids. *Angew. Chem. Int. Ed*. **2014**, 53, 10510–10514.
9. Xing, X.; Xu, C.; Chen, B.; Li, C.; Virgil, S.C.; Grubbs, R.H. Highly Active Platinum Catalysts for Nitrile and Cyanohydrin Hydration: Catalyst Design and Ligand Screening via High-Throughput Techniques. *J. Am. Chem. Soc.* **2018**, *140*, 17782–17789.
10. Mahajani, N. S.; Chisholm, J. D.; Synthesis of 1,1'-Diarylethanes and Related Systems by Displacement of Trichloroacetimidates with Trimethylaluminum. *J. Org. Chem.* **2018**, *83*, 7, 4131–4139.
11. Litak, N.P.; Zheng, S.; Cui, D.; Betley, T.A. Synthesis of a Stable Tricobalt Carbide Cluster. *J. Am. Chem. Soc*. **2025**, *147*, 20056–20062.
12. Zhang, S.; Occhialini, G.; Carder, H.M.; de Kleijne, F.F.J.; Wendlandt, A.E. Selective epimerization of GlcNAc to GalNAc through steady-state tuning under kinetic network control. *Nat. Chem.* **2026**, *18*, 967–974.
13. Qiu, G.; Ni, C.; Knowles, R.R. Isotopic Fractionation as a Mechanistic Probe in Light-Driven C–H Bond Exchange Reactions. *J. Am. Chem. Soc.* **2023**, *145*, 11537–11543.
14. Tay, J.; Nagorny, P. ^{31}P NMR Based Method for Determining Enantiopurity of Chiral Phosphoric Acids and Its Application to the BINOL- and H_8-BINOL-Based Chiral Phosphoric Acid Thermal Racemization Studies. *Synlett*, **2016**, *27*, 551–554.
15. DeFord, J.; Chu, F.; Anslyn, E. V. Dimerization Constants for Phosphoric Acid Diesters. *Tet. Lett.* **1996**, *37*, 1925–1928.
16. Yu, J.; Blackmond, D.G. Nonclassical Nonlinear Effects (nc-NLEs) Provide Mechanistic Insights in Asymmetric Catalytic Cascade Reactions. *ACS Catal.* **2025**, *15*, 3890–3897.

17. Kalek, M.; Fu, G. Caution in the Use of Nonlinear Effects as a Mechanistic Tool for Catalytic Enantioconvergent Reactions: Intrinsic Negative Nonlinear Effects in the Absence of Higher-Order Species. *J. Am. Chem. Soc.* **2017**, *139*, 4225–4229.
18. Morton, C.A.; Zhu, Q.; Ripberger, H.; Troian-Gautier, L.; Toa, Z.S.D.; Knowles, R.R.; Alexanian, E.J. C–H alkylation via multisite-proton-coupled electron transfer of an aliphatic C–H bond. *J. Am. Chem. Soc.* **2019**, *141*, 13253–13260.
19. Gaussian 16, Revision C.01, Frisch, M. J.; Trucks, G. W.; Schlegel, H. B.; Scuseria, G. E.; Robb, M. A.; Cheeseman, J. R.; Scalmani, G.; Barone, V.; Petersson, G. A.; Nakatsuji, H.; Li, X.; Caricato, M.; Marenich, A. V.; Bloino, J.; Janesko, B. G.; Gomperts, R.; Mennucci, B.; Hratchian, H. P.; Ortiz, J. V.; Izmaylov, A. F.; Sonnenberg, J. L.; Williams-Young, D.; Ding, F.; Lipparini, F.; Egidi, F.; Goings, J.; Peng, B.; Petrone, A.; Henderson, T.; Ranasinghe, D.; Zakrzewski, V. G.; Gao, J.; Rega, N.; Zheng, G.; Liang, W.; Hada, M.; Ehara, M.; Toyota, K.; Fukuda, R.; Hasegawa, J.; Ishida, M.; Nakajima, T.; Honda, Y.; Kitao, O.; Nakai, H.; Vreven, T.; Throssell, K.; Montgomery, J. A., Jr.; Peralta, J. E.; Ogliaro, F.; Bearpark, M. J.; Heyd, J. J.; Brothers, E. N.; Kudin, K. N.; Staroverov, V. N.; Keith, T. A.; Kobayashi, R.; Normand, J.; Raghavachari, K.; Rendell, A. P.; Burant, J. C.; Iyengar, S. S.; Tomasi, J.; Cossi, M.; Millam, J. M.; Klene, M.; Adamo, C.; Cammi, R.; Ochterski, J. W.; Martin, R. L.; Morokuma, K.; Farkas, O.; Foresman, J. B.; Fox, D. J. Gaussian, Inc., Wallingford CT, 2016.
20. (a) Becke, A.D. *J. Chem. Phys.* **1993**, *98*, 5648–5652. (b) Lee, C.; Yang, W.; Parr, R. G. Development of the Colle-Salvetti correlation-energy formula into a functional of the electron density. *Phys. Rev. B.* **1988**, *37*, 785–789.; (c) Stephens, P.J.; Devlin, F.J.; Chabalowski, C.F.; Frisch, M.J. *J. Phys. Chem.* **1994**, *98*, 11623-11627
21. Grimme, S.; Antony, J.; Ehrlich, S.; Krieg, H. A consistent and accurate ab initio parametrization of density functional dispersion correction (DFT-D) for the 94 elements H-Pu. *J. Chem. Phys.* **2010**, *132*, 154104.
22. Weigend, F.; Ahlrichs, R. Balanced basis sets of split valence, triple zeta valence and quadruple zeta valence quality for H to Rn: Design and assessment of accuracy. *Phys. Chem. Chem. Phys.* **2005**, *7*, 3297–3305.
23. Cossi, M.; Rega, N.; Scalmani, G.; Barone, V. J. Energies, structures, and electronic properties of molecules in solution with the C-PCM solvation model. *Comput. Chem.* **2003**, *24*, 669–683.
24. Bordwell, F.G. Equilibrium acidities in dimethyl sulfoxide solution. *Acc. Chem. Res.* **1988**, *21*, 456–463.
25. Francl, M.M.; Pietro, W.J.; Hehre, W.J.; Binkley, J.S.; Gordon, M.S.; DeFrees, D.J.; Pople, J.A. Self-consistent molecular orbital methods. XXIII. A polarization-type basis set for second-row elements. *J. Chem. Phys.* **1982**, *77*, 3654–3665.
26. Sim, B.A.; Griller, D.; Wayner, D.D.M. Reduction potentials for substituted benzylic radicals: pK_a values for the corresponding toluenes. *J. Am. Chem. Soc.* **1989**, *111*, 754–755.
27. ^{13}C NMR chemical shifts are reported for *rac*-**1w** in $CDCl_3$ and DMSO-d_6 to resolve overlapping aromatic carbons.
